# Supplementary material for: A comprehensive review of randomized clinical trials in three medical journals reveals 396 medical reversals
Source: eLife. 2019 Jun 11;8:e45183. doi: 10.7554/eLife.45183 (PMC6559784; doi:10.7554/eLife.45183)
Supplement: Supplementary file 1. [file elife-45183-supp1.docx]

Supplementary File 1: Funding sources for articles in which there was a medical reversal identified

| **LANCET** | **Date** | **Title of article** | **funding body(ies)** | **funding category: 1)industry; 2)non-industry; 3)combination**  **4)non-industry plus insurance or banking institution** |
| --- | --- | --- | --- | --- |
| LANCET | 10/21/2017 | Effectiveness of household lockable pesticide storage to reduce pesticide self-poisoning in rural Asia: a community-based, cluster-randomised controlled trial | Wellcome Trust, with additional support from the American Foundation for Suicide Prevention, Lister Institute of Preventive Medicine, Chief Scientist Office of Scotland, University of Copenhagen, and NHMRC Australia. | 3 |
| LANCET | 8/5/2017 | Family-led rehabilitation after stroke in India (ATTEND): a randomised controlled trial | The National Health and Medical Research Council of Australia. | 2 |
| LANCET | 7/15/2017 | Intraoperative ketamine for prevention of postoperative delirium or pain after major surgery in older adults: an international, multicentre, double-blind, randomised clinical trial | National Institutes of Health and Cancer Center Support. | 2 |
| LANCET | 4/22/2017 | Prophylactic platelet transfusion plus supportive care versus supportive care alone in adults with dengue and thrombocytopenia: a multicentre, open-label, randomised, superiority trial | National Medical Research Council, Singapore. | 2 |
| LANCET | 4/8/2017 | Post-deployment screening for mental disorders and tailored advice about help-seeking in the UK military: a cluster randomised controlled trial | The US Army Medical Research and Materiel Command–Military Operational Medicine Research Program (USAMRMC–MOMRP). | 2 |
| LANCET | 4/1/2017 | Prophylactic hydration to protect renal function from intravascular iodinated contrast material in patients at high risk of contrast-induced nephropathy (AMACING): a prospective, randomised, phase 3, controlled, open-label, non-inferiority trial | Stichting de Weijerhorst. | 2 |
| LANCET | 3/4/2017 | High-flow warm humidified oxygen versus standard low-flow nasal cannula oxygen for moderate bronchiolitis (HFWHO RCT): an open, phase 4, randomised controlled trial | Hunter Children's Research Foundation, John Hunter Hospital Charitable Trust, and the University of Newcastle Priority Research Centre GrowUpWell. | 2 |
| LANCET | 11/19/2016 | Comparison of an everolimus-eluting bioresorbable scaffold with an everolimus-eluting metallic stent for the treatment of coronary artery stenosis (ABSORB II): a 3 year, randomised, controlled, single-blind, multicentre clinical trial | Abbott Vascular. | 1 |
| LANCET | 11/12/2016 | Comparison of stapled haemorrhoidopexy with traditional excisional surgery for haemorrhoidal disease (eTHoS): a pragmatic, multicentre, randomised controlled trial | National Institute for Health Research Health Technology Assessment programme. | 2 |
| LANCET | 11/5/2016 | Efficacy of infant simulator programmes to prevent teenage pregnancy: a school-based cluster randomised controlled trial in Western Australia | Western Australian Health Promotion Foundation (Healthway), Lotteries WA, the Western Australian Department of Education and Training, and the Western Australian Department of Health. | 2 |
| LANCET | 10/22/2016 | Platelet function monitoring to adjust antiplatelet therapy in elderly patients stented for an acute coronary syndrome (ANTARCTIC): an open-label, blinded-endpoint, randomised controlled superiority trial | Eli Lilly and Company, Daiichi Sankyo, Stentys, Accriva Diagnostics, Medtronic, and Fondation Coeur et Recherche. | 3 |
| LANCET | 10/22/2016 | Dexamethasone and supportive care with or without whole brain radiotherapy in treating patients with non-small cell lung cancer with brain metastases unsuitable for resection or stereotactic radiotherapy (QUARTZ): results from a phase 3, non-inferiority, randomised trial | Cancer Research UK, Medical Research Council Clinical Trials Unit at University College London, and the National Health and Medical Research Council in Australia. | 2 |
| LANCET | 9/10/2016 | Robot-assisted laparoscopic prostatectomy versus open radical retropubic prostatectomy: early outcomes from a randomised controlled phase 3 study | Cancer Council Queensland. | 2 |
| LANCET | 8/13/2016 | Immediate total-body CT scanning versus conventional imaging and selective CT scanning in patients with severe trauma (REACT-2): a randomised controlled trial | ZonMw, the Netherlands Organisation for Health Research and Development. | 2 |
| LANCET | 6/25/2016 | Platelet transfusion versus standard care after acute stroke due to spontaneous cerebral haemorrhage associated with antiplatelet therapy (PATCH): a randomised, open-label, phase 3 trial | The Netherlands Organisation for Health Research and Development, Sanquin Blood Supply, Chest Heart and Stoke Scotland, French Ministry of Health. | 2 |
| LANCET | 6/25/2016 | Hysteroscopy in recurrent in-vitro fertilisation failure (TROPHY): a multicentre, randomised controlled trial | European Society of Human Reproduction and Embryology, European Society for Gynaecological Endoscopy. | 2 |
| LANCET | 6/25/2016 | Hysteroscopy before in-vitro fertilisation (inSIGHT): a multicentre, randomised controlled trial | The Dutch Organisation for Health Research and Development (ZonMW). | 2 |
| LANCET | 1/30/2016 | Immediate delivery compared with expectant management after preterm pre-labour rupture of the membranes close to term (PPROMT trial): a randomised controlled trial | Australian National Health and Medical Research Council, the Women's and Children's Hospital Foundation, and The University of Sydney. | 2 |
| LANCET | 1/9/2016 | Effectiveness of a nurse-led intensive home-visitation programme for first-time teenage mothers (Building Blocks): a pragmatic randomised controlled trial | Department of Health Policy Research Programme. | 2 |
| LANCET | 1/9/2016 | Outcomes after thrombus aspiration for ST elevation myocardial infarction: 1-year follow-up of the prospective randomised TOTAL trial | Canadian Institutes of Health Research, Canadian Network and Centre for Trials Internationally, and Medtronic Inc. | 3 |
| LANCET | 11/7/2015 | Prophylactic antibiotics after acute stroke for reducing pneumonia in patients with dysphagia (STROKE-INF): a prospective, cluster-randomised, open-label, masked endpoint, controlled clinical trial | UK National Institute for Health Research. | 2 |
| LANCET | 11/7/2015 | Early combined immunosuppression for the management of Crohn's disease (REACT): a cluster randomised controlled trial | AbbVie Pharmaceuticals. | 1 |
| LANCET | 10/24/2015 | Percutaneous tibial nerve stimulation versus sham electrical stimulation for the treatment of faecal incontinence in adults (CONFIDeNT): a double-blind, multicentre, pragmatic, parallel-group, randomised controlled trial | National Institute for Health Research. | 2 |
| LANCET | 9/26/2015 | Methylprednisolone in patients undergoing cardiopulmonary bypass (SIRS): a randomised, double-blind, placebo-controlled trial | Canadian Institutes of Health Research. | 2 |
| LANCET | 7/25/2015 | Medical expulsive therapy in adults with ureteric colic: a multicentre, randomised, placebo-controlled trial | UK National Institute for Health Research Health Technology Assessment Programme. | 2 |
| LANCET | 7/4/2015 | Efficacy and safety of very early mobilisation within 24 h of stroke onset (AVERT): a randomised controlled trial | National Health and Medical Research Council, Singapore Health, Chest Heart and Stroke Scotland, Northern Ireland Chest Heart and Stroke, UK Stroke Association, National Institute of Health Research. | 2 |
| LANCET | 5/16/2015 | Automated, electronic alerts for acute kidney injury: a single-blind, parallel-group, randomised controlled trial | Penn Center for Healthcare Improvement and Patient Safety. | 2 |
| LANCET | 4/11/2015 | Efficacy of indoor residual spraying with dichlorodiphenyltrichloroethane against malaria in Gambian communities with high usage of long-lasting insecticidal mosquito nets: a cluster-randomised controlled trial | UK Medical Research Council. | 2 |
| LANCET | 4/4/2015 | Effect of early neonatal vitamin A supplementation on mortality during infancy in Ghana (Neovita): a randomised, double-blind, placebo-controlled trial | Bill & Melinda Gates Foundation grant to the WHO. | 2 |
| LANCET | 4/4/2015 | Effect of neonatal vitamin A supplementation on mortality in infants in Tanzania (Neovita): a randomised, double-blind, placebo-controlled trial | Bill & Melinda Gates Foundation to WHO. | 2 |
| LANCET | 2/14/2015 | A population-based, multifaceted strategy to implement antenatal corticosteroid treatment versus standard care for the reduction of neonatal mortality due to preterm birth in low-income and middle-income countries: the ACT cluster-randomised trial | Eunice Kennedy Shriver National Institute of Child Health and Human Development | 2 |
| LANCET | 11/8/2014 | Antepartum dalteparin versus no antepartum dalteparin for the prevention of pregnancy complications in pregnant women with thrombophilia (TIPPS): a multinational open-label randomised trial | Canadian Institutes of Health Research, Heart and Stroke Foundation of Canada, and Pharmacia and UpJohn. | 3 |
| LANCET | 11/1/2014 | Efficacy of paracetamol for acute low-back pain: a double-blind, randomised controlled trial | National Health and Medical Research Council of Australia and GlaxoSmithKline Australia. | 3 |
| LANCET | 9/20/2014 | Efficacy and cost of video-assisted thoracoscopic partial pleurectomy versus talc pleurodesis in patients with malignant pleural mesothelioma (MesoVATS): an open-label, randomised, controlled trial | BUPA Foundation. | 2 |
| LANCET | 8/9/2014 | High versus low positive end-expiratory pressure during general anaesthesia for open abdominal surgery (PROVHILO trial): a multicentre randomised controlled trial | Academic Medical Center (Amsterdam, Netherlands), European Society of Anaesthesiology. | 2 |
| LANCET | 7/19/2014 | Effect of gravity on volume of placental transfusion: a multicentre, randomised, non-inferiority trial | Foundation for Maternal and Child Health (FUNDASAMIN). | 2 |
| LANCET | 7/12/2014 | Comprehensive physiotherapy exercise programme or advice for chronic whiplash (PROMISE): a pragmatic randomised controlled trial | The National Health and Medical Research Council of Australia, Motor Accidents Authority of New South Wales, and Motor Accident Insurance Commission of Queensland. | 4 |
| LANCET | 3/8/2014 | Compression stockings to prevent post-thrombotic syndrome: a randomised placebo-controlled trial | Canadian Institutes of Health Research. | 2 |
| LANCET | 2/15/2014 | Medical management with or without interventional therapy for unruptured brain arteriovenous malformations (ARUBA): a multicentre, non-blinded, randomised trial | National Institutes of Health, National Institute of Neurological Disorders and Stroke. | 2 |
| LANCET | 1/18/2014 | Liverpool Care Pathway for patients with cancer in hospital: a cluster randomised trial | Italian Ministry of Health and Maruzza Lefebvre D'Ovidio Foundation-Onlus. | 2 |
| LANCET | 12/21/2013 | A structured training programme for caregivers of inpatients after stroke (TRACS): a cluster randomised controlled trial and cost-effectiveness analysis | Medical Research Council. | 2 |
| LANCET | 11/16/2013 | Intra-aortic balloon counterpulsation in acute myocardial infarction complicated by cardiogenic shock (IABP-SHOCK II): final 12 month results of a randomised, open-label trial | German Research Foundation; German Heart Research Foundation; German Cardiac Society; Arbeitsgemeinschaft Leitende Kardiologische Krankenhausärzte; University of Leipzig—Heart Centre; Maquet Cardiopulmonary; Teleflex Medical. | 3 |
| LANCET | 10/5/2013 | Effect of household and community interventions on the burden of tuberculosis in southern Africa: the ZAMSTAR community-randomised trial | Bill & Melinda Gates Foundation. | 2 |
| LANCET | 7/20/2013 | Screening and counselling in the primary care setting for women who have experienced intimate partner violence (WEAVE): a cluster randomised controlled trial | Australian National Health and Medical Research Council. | 2 |
| LANCET | 7/6/2013 | Exercise for depression in elderly residents of care homes: a cluster-randomised controlled trial | National Institute for Health Research Health Technology Assessment. | 2 |
| LANCET | 5/11/2013 | Community treatment orders for patients with psychosis (OCTET): a randomised controlled trial | National Institute of Health Research. | 2 |
| LANCET | 4/27/2013 | Population deworming every 6 months with albendazole in 1 million pre-school children in north India: DEVTA, a cluster-randomised trial | UK Medical Research Council, USAID, World Bank (albendazole donated by GlaxoSmithKline). | 3 |
| LANCET | 2/23/2013 | Biolimus-eluting biodegradable polymer-coated stent versus durable polymer-coated sirolimus-eluting stent in unselected patients receiving percutaneous coronary intervention (SORT OUT V): a randomised non-inferiority trial | Terumo and Cordis (Johnson & Johnson). | 1 |
| LANCET | 2/16/2013 | Emergency department treatments and physiotherapy for acute whiplash: a pragmatic, two-step, randomised controlled trial | NIHR Health Technology Assessment programme | 2 |
| LANCET | 12/1/2012 | Antimicrobial catheters for reduction of symptomatic urinary tract infection in adults requiring short-term catheterisation in hospital: a multicentre randomised controlled trial | UK National Institute for Health Research Health Technology Assessment Programme. | 2 |
| LANCET | 11/17/2012 | Screening for type 2 diabetes and population mortality over 10 years (ADDITION-Cambridge): a cluster-randomised controlled trial | Wellcome Trust; UK Medical Research Council; National Health Service research and development support; UK National Institute for Health Research; University of Aarhus, Denmark; Bio-Rad. | 3 |
| LANCET | 1/14/2012 | Comparison of annual versus twice-yearly mass azithromycin treatment for hyperendemic trachoma in Ethiopia: a cluster-randomised trial | National Institutes of Health (NEI U10 EY016214). | 2 |
| LANCET | 7/30/2011 | Sertraline or mirtazapine for depression in dementia (HTA-SADD): a randomised, multicentre, double-blind, placebo-controlled trial | UK National Institute of Health Research HTA Programme. | 2 |
| LANCET | 7/23/2011 | Prednisone versus tamoxifen in patients with idiopathic retroperitoneal fibrosis: an open-label randomised controlled trial | Parma University Hospital. | 2 |
| LANCET | 7/23/2011 | Urinary incontinence in men after formal one-to-one pelvic-floor muscle training following radical prostatectomy or transurethral resection of the prostate (MAPS): two parallel randomised controlled trials | National Institute of Health Research, Health Technology Assessment (NIHR HTA) Programme. | 2 |
| LANCET | 2/26/2011 | The angiotensin-receptor blocker candesartan for treatment of acute stroke (SCAST): a randomised, placebo-controlled, double-blind trial | South-Eastern Norway Regional Health Authority; Oslo University Hospital Ullevål; AstraZeneca; Takeda. | 3 |
| LANCET | 1/15/2011 | High-dose vitamin D3 during intensive-phase antimicrobial treatment of pulmonary tuberculosis: a double-blind randomised controlled trial | British Lung Foundation. | 2 |
| LANCET | 1/9/2010 | Umbilical vein oxytocin for the treatment of retained placenta (Release Study): a double-blind, randomised controlled trial | WHO, WellBeing of Women, Pakistan Higher Education Commission. | 2 |
| LANCET | 2/13/2010 | Comparative effectiveness of MRI in breast cancer (COMICE) trial: a randomised controlled trial | National Institute for Health Research's Health Technology Assessment Programme. | 2 |
| LANCET | 3/20/2010 | Carotid artery stenting compared with endarterectomy in patients with symptomatic carotid stenosis (International Carotid Stenting Study): an interim analysis of a randomised controlled trial | Medical Research Council, the Stroke Association, Sanofi-Synthélabo, European Union. | 3 |
| LANCET | 5/8/2010 | Effect of vitamin A supplementation in women of reproductive age on maternal survival in Ghana (ObaapaVitA): a cluster-randomised, placebo-controlled trial | UK Department for International Development, and USAID. | 2 |
| LANCET | 5/22/2010 | Misoprostol as an adjunct to standard uterotonics for treatment of post-partum haemorrhage: a multicentre, double-blind randomised trial | Bill & Melinda Gates Foundation, and UNDP/UNFPA/WHO/World Bank Special Programme of Research, Development and Research Training in Human Reproduction. | 2 |
| LANCET | 9/4/2010 | Effect of palliative oxygen versus room air in relief of breathlessness in patients with refractory dyspnoea: a double-blind, randomised controlled trial | US National Institutes of Health, Australian National Health and Medical Research Council, Duke Institute for Care at the End of Life, and Doris Duke Charitable Foundation. | 2 |
| LANCET | 10/2/2010 | Early versus delayed treatment of relapsed ovarian cancer (MRC OV05/EORTC 55955): a randomised trial | UK Medical Research Council and the European Organisation for Research and Treatment of Cancer. | 2 |
| LANCET | 1/10/2009 | Efficacy of systematic pelvic lymphadenectomy in endometrial cancer (MRC ASTEC trial): a randomised study | Medical Research Council and National Cancer Research Network. | 2 |
| LANCET | 2/14/2009 | Warfarin thromboprophylaxis in cancer patients with central venous catheters (WARP): an open-label randomised trial | Medical Research Council and Cancer Research UK. | 2 |
| LANCET | 6/6/2009 | Effectiveness of thigh-length graduated compression stockings to reduce the risk of deep vein thrombosis after stroke (CLOTS trial 1): a multicentre, randomised controlled trial | Medical Research Council (UK), Chief Scientist Office of Scottish Government, Chest Heart and Stroke Scotland, Tyco Healthcare (Covidien) USA, and UK Stroke Research Network. | 3 |
| LANCET | 7/18/2009 | Effect of interferon gamma-1b on survival in patients with idiopathic pulmonary fibrosis (INSPIRE): a multicentre, randomised, placebo-controlled trial | InterMune | 1 |
| LANCET | 11/14/2009 | Comparison of routine and on-demand prescription of chest radiographs in mechanically ventilated adults: a multicentre, cluster-randomised, two-period crossover study | Assistance Publique-Hôpitaux de Paris (Direction Régionale de la Recherche Clinique Ile de France). | 2 |
| LANCET | 12/5/2009 | Chlorhexidine maternal-vaginal and neonate body wipes in sepsis and vertical transmission of pathogenic bacteria in South Africa: a randomised, controlled trial | US Agency for International Development, National Vaccine Program Office and Centers for Disease Control's Antimicrobial Resistance Working Group, and Bill & Melinda Gates Foundation. | 2 |
| LANCET | 1/5/2008 | Risperidone, haloperidol, and placebo in the treatment of aggressive challenging behaviour in patients with intellectual disability: a randomised controlled trial | National Coordinating Centre for Health Technology Assessment (NCCHTA), Southampton UK | 2 |
| LANCET | 2/16/2008 | Multiple-dose activated charcoal in acute self-poisoning: a randomised controlled trial | Wellcome Trust's Tropical Interest Group | 2 |
| LANCET | 5/17/2008 | Active symptom control with or without chemotherapy in the treatment of patients with malignant pleural mesothelioma (MS01): a multicentre randomised trial | Cancer Research UK and the Medical Research Council (UK). | 2 |
| LANCET | 9/20/2008 | Management of asthma based on exhaled nitric oxide in addition to guideline-based treatment for inner-city adolescents and young adults: a randomised controlled trial | US National Institute of Allergy and Infectious Diseases, US National Institutes of Health. | 2 |
| LANCET | 10/4/2008 | Effect of rosuvastatin in patients with chronic heart failure (the GISSI-HF trial): a randomised, double-blind, placebo-controlled trial | Società Prodotti Antibiotici (SPA; Italy), Pfizer, Sigma Tau, and AstraZeneca. | 1 |
| LANCET | 11/15/2008 | Cardiovascular events associated with rofecoxib: final analysis of the APPROVe trial | Merck Research Laboratories. | 1 |
| LANCET | 3/17/2007 | Effect of zinc supplementation on mortality in children aged 1–48 months: a community-based randomised placebo-controlled trial | WHO | 2 |
| LANCET | 6/23/2007 | Effectiveness of an early supplementation scheme of high-dose vitamin A versus standard WHO protocol in Gambian mothers and infants: a randomised controlled trial | UK Medical Research Council | 2 |
| LANCET | 10/6/2007 | Effect of daily zinc supplementation on child mortality in southern Nepal: a community-based, cluster randomised, placebo-controlled trial | Eunice Kennedy Shriver National Institute of Child Health and Human Development (NICHD), United States Agency for International Development (USAID), Bill and Melinda Gates Foundation | 2 |
| LANCET | 11/10/2007 | Assessment of diclofenac or spinal manipulative therapy, or both, in addition to recommended first-line treatment for acute low back pain: a randomised controlled trial | Australia's National Health and Medical Research Council, (Active diclofenac donated by Alphapharm) | 3 |
| LANCET | 12/22/2007 | Mechanical bowel preparation for elective colorectal surgery: a multicentre randomised trial | Ikazia Hospital, Rotterdam | 2 |
| LANCET | 1/14/2006 | Effects of routine prophylactic supplementation with iron and folic acid on admission to hospital and mortality in preschool children in a high malaria transmission setting: community-based, randomised, placebo-controlled trial | WHO | 2 |
| LANCET | 5/13/2006 | Aminophylline in bradyasystolic cardiac arrest: a randomised placebo-controlled trial | Heart and Stroke Foundation of British Columbia and Yukon, Vancouver Coastal Health Research Institute | 2 |
| LANCET | 7/15/2006 | Intrauterine insemination with controlled ovarian hyperstimulation versus expectant management for couples with unexplained subfertility and an intermediate prognosis: a randomised clinical trial | Academic Medical Centre (AMC) (Netherlands) | 2 |
| LANCET | 7/29/2006 | Continuous venovenous haemodiafiltration versus intermittent haemodialysis for acute renal failure in patients with multiple-organ dysfunction syndrome: a multicentre randomised trial | Société de Réamination de Langue Francaise; Délégation à la Recherche Clinique de l’Assistance Publique-Hôpitaux de Paris; Hospal Inc | 2 |
| LANCET | 8/26/2006 | Secondary prevention of asthma by the use of Inhaled Fluticasone propionate in Wheezy INfants (IFWIN): double-blind, randomised, controlled study | GlaxoSmithKline | 1 |
| LANCET | 10/7/2006 | 30 day results from the SPACE trial of stent-protected angioplasty versus carotid endarterectomy in symptomatic patients: a randomised non-inferiority trial | Federal Ministry of Education and Research (BMBF: 01GI9918), German Research Foundation (DFG: HA 1394/4-2 and HA 1397/4-3), German Society of Neurology, German Society of Neuroradiology, German Radiological Society), Boston Scientific, Guidant, and Sanofi-Aventis. | 3 |
| LANCET | 10/21/2006 | Magnesium sulphate for treatment of severe tetanus: a randomised controlled trial | University of Oxford (UK) | 2 |
| LANCET | 12/9/2006 | Effect of mammographic screening from age 40 years on breast cancer mortality at 10 years' follow-up: a randomised controlled trial | Medical Research Council and Cancer Research UK, Department of Health and the US National Cancer Research Institute | 2 |
| LANCET | 1/29/2005 | Early surgery versus initial conservative treatment in patients with spontaneous supratentorial intracerebral haematomas in the International Surgical Trial in Intracerebral Haemorrhage (STICH): a randomised trial | UK Medical Research Council. | 2 |
| LANCET | 2/26/2005 | Routine oral nutritional supplementation for stroke patients in hospital (FOOD): a multicentre randomised controlled trial | Health Technology Assessment Board of NHS Research and Development in UK, the Stroke Association, the Chief Scientist Office of the Scottish Executive, and Chest, Heart and Stroke Scotland. The Royal Australasian College of Physicians supported the trial in Hawkes Bay, New Zealand | 2 |
| LANCET | 2/26/2005 | Effect of timing and method of enteral tube feeding for dysphagic stroke patients (FOOD): a multicentre randomised controlled trial | Health Technology Assessment Board of NHS Research and Development in UK, the Stroke Association, the Chief Scientist Office of the Scottish Executive, and Chest, Heart and Stroke Scotland. The Royal Australasian College of Physicians supported the trial in Hawkes Bay, New Zealand | 2 |
| LANCET | 4/30/2005 | Effects of N-acetylcysteine on outcomes in chronic obstructive pulmonary disease (Bronchitis Randomized on NAC Cost-Utility Study, BRONCUS): a randomised placebo-controlled trial | Zambon Group Spa | 1 |
| LANCET | 5/7/2005 | Oral vitamin D3 and calcium for secondary prevention of low-trauma fractures in elderly people (Randomised Evaluation of Calcium Or vitamin D, RECORD): a randomised placebo-controlled trial | The UK Medical Research Council, Shire Pharmaceuticals funded the drugs | 3 |
| LANCET | 6/18/2005 | Introduction of the medical emergency team (MET) system: a cluster-randomised controlled trial | Australian National Health and Medical Research Council, the Australian Council for Quality and Safety in Healthcare, and the Australian and New Zealand Intensive Care Foundation | 2 |
| LANCET | 6/25/2005 | Endovascular aneurysm repair versus open repair in patients with abdominal aortic aneurysm (EVAR trial 1): randomised controlled trial | National Health Service Research and Development Health Technology Assessment Programme | 2 |
| LANCET | 6/25/2005 | Endovascular aneurysm repair and outcome in patients unfit for open repair of abdominal aortic aneurysm (EVAR trial 2): randomised controlled trial | National Health Service Research and Development Health Technology Assessment Programme | 2 |
| LANCET | 7/2/2005 | Chloramphenicol treatment for acute infective conjunctivitis in children in primary care: a randomised double-blind placebo-controlled trial | Medical Research Council | 2 |
| LANCET | 8/6/2005 | Combination antibiotic susceptibility testing to treat exacerbations of cystic fibrosis associated with multiresistant bacteria: a randomised, double-blind, controlled clinical trial | Chiron Corporation for supplying TOBI (inhaled tobramycin) and placebo tobramycin to the Canadian study sites. This study was funded by grants from The Canadian Institutes of Health Research (C$319 225), The Australian CF Trust (A$138 000), Astra-Zeneca Canada Inc (unrestricted research grant, C$150 000), and The Canadian CF Foundation (C$98 526) | 3 |
| LANCET | 8/6/2005 | Assessment of the clinical effectiveness of pulmonary artery catheters in management of patients in intensive care (PAC-Man): a randomised controlled trial | UK NHS Research and Development Health Technology Assessment Programme | 2 |
| LANCET | 10/8/2005 | Effect of BCG revaccination on incidence of tuberculosis in school-aged children in Brazil: the BCG-REVAC cluster-randomised trial | Department of International Development (DFID), UKand the National Health Foundation (FUNASA), Brazil | 2 |
| LANCET | 1/24/2004 | Doubling the dose of inhaled corticosteroid to prevent asthma exacerbations: randomised controlled trial | NHS Executive | 2 |
| LANCET | 8/14/2004 | Oropharyngeal and nasopharyngeal suctioning of meconium-stained neonates before delivery of their shoulders: multicentre, randomised controlled trial | Pediatrics/American Heart Association Neonatal Resuscitation Program | 2 |
| LANCET | 10/9/2004 | Effect of intravenous corticosteroids on death within 14 days in 10 008 adults with clinically significant head injury (MRC CRASH trial): randomised placebo-controlled trial | UK Medical Research Council. Pharmacia and Upjohn (Pfizer from 2003) provided the MRC (without charge) the methylprednisolone needed for the trial, a grant-in-aid for preparation of the placebo, and support for collaborators' meetings | 3 |
| LANCET | 11/13/2004 | The United Kingdom Infantile Spasms Study comparing vigabatrin with prednisolone or tetracosactide at 14 days: a multicentre, randomised controlled trial | Bath Unit for Research in Paediatrics | 2 |
| LANCET | 2/1/2003 | Intrahepatic arterial versus intravenous fluorouracil and folinic acid for colorectal cancer liver metastases: a multicentre randomised trial | Medical Research Council and Cancer Research UK | 2 |
| LANCET | 2/8/2003 | Comparison of intermittent and continuous palliative chemotherapy for advanced colorectal cancer: a multicentre randomised trial | MRC grant. AstraZeneca provided free raltitrexed and a financial grant to support research costs of the centres participating | 3 |
| LANCET | 2/8/2003 | Admission cardiotocography: a randomised controlled trial | Research Committee of the National Maternity Hospital | 2 |
| LANCET | 2/22/2003 | Syndromic management of sexually-transmitted infections and behaviour change interventions on transmission of HIV-1 in rural Uganda: a community randomised trial | Medical Research Council and the UK Department for International Development with partial funding from initially the Global Programme on AIDS and later United Nations Programme on AIDS | 2 |
| LANCET | 4/12/2003 | Laparoscopic adhesiolysis in patients with chronic abdominal pain: a blinded randomised controlled multi-centre trial | NI | NI |
| LANCET | 6/28/2003 | Effect of conjugate pneumococcal vaccine followed by polysaccharide pneumococcal vaccine on recurrent acute otitis media: a randomised study | Netherlands Organisation for Health Research and Development ZonMw (grant number 002828480) and the Netherlands Health Insurance Company Zilveren Kruis-Achmea | 2 |
| LANCET | 8/16/2003 | Long-term effect of a watch and wait policy versus immediate systemic treatment for asymptomatic advanced-stage non-Hodgkin lymphoma: a randomised controlled trial | Lymphoma Research Trust | 2 |
| LANCET | 11/1/2003 | Efficacy of a short course of parent-initiated oral prednisolone for viral wheeze in children aged 1–5 years: randomised controlled trial | NHS National Research and Development Programme on Asthma Management | 2 |
| JAMA | 2/7/2017 | Video Laryngoscopy vs Direct Laryngoscopy on Successful First-Pass Orotracheal Intubation Among ICU Patients A Randomized Clinical Trial | Centre Hospitalier Département de la Vendée | 2 |
| JAMA | 2/14/2017 | Effect of 2 Years of Treatment With Sublingual Grass Pollen Immunotherapy on Nasal Response to Allergen Challenge at 3 Years Among Patients With Moderate to Severe Seasonal Allergic Rhinitis The GRASS Randomized Clinical Trial | National Institutes of Health; Denmark supplied Alutard SQ Grass Pollen, and Grazax, and matching placebos | 2 |
| JAMA | 2/21/2017 | Testosterone Treatment and Cognitive Function in Older Men With Low Testosterone and Age-Associated Memory Impairment | NIA/NIH, supplemented by funds from the National Heart, Lung, and Blood Institute; National Institute of Neurological Disorders and Stroke; and National Institute of Child Health and Human Development. AbbVie provided funding, AndroGel, and placebo gel. The Boston site was partially supported by the Claude D. Pepper Older Americans Independence Center (OAIC). The Yale Field Center was partially supported by Claude D. Pepper OAIC and the Yale Clinical and Translational Science Award. Additional support was from the Department of Veterans Affairs Puget Sound Health Care System; the Academic Leadership Award from the NIA; the Intramural Research Program of NIA/NIH National Institute for Diabetes and Digestive and Kidney Diseases of NIH to the University of Alabama at Birmingham Diabetes Research and Training Center; NIA/NIH; and the Boston Claude D. Pepper OAIC | 3 |
| JAMA | 2/21/2017 | Effect of Fibrinogen Concentrate on Intraoperative Blood Loss Among Patients With Intraoperative Bleeding During High-Risk Cardiac Surgery A Randomized Clinical Trial | CSL Behring | 1 |
| JAMA | 3/14/2017 | Effect of Inpatient Rehabilitation vs a Monitored Home-Based Program on Mobility in Patients With Total Knee Arthroplasty The HIHO Randomized Clinical Trial | HCF Research Foundation and was supported by the South Western Sydney Local Health District through the Whitlam Orthopaedic Research Centre and by HammondCare and the Ingham Institute | 2 |
| JAMA | 3/14/2017 | Effect of an Integrated Pest Management Intervention on Asthma Symptoms Among Mouse-Sensitized Children and Adolescents With Asthma A Randomized Clinical Trial | National Institute of Allergy and Infectious Diseases, National Institute of Environmental Health Sciences. The 3M Corporation donated air purifiers and CleanBrands LLC donated allergen-proof mattress and pillow encasements | 3 |
| JAMA | 4/4/2017 | Effect of Dexmedetomidine on Mortality and Ventilator-Free Days in Patients Requiring Mechanical Ventilation With Sepsis A Randomized Clinical Trial | Wakayama Medical University | 2 |
| JAMA | 5/16/2017 | Effect of Intra-articular Triamcinolone vs Saline on Knee Cartilage Volume and Pain in Patients With Knee Osteoarthritis A Randomized Clinical Trial | National Institute for Arthritis and Musculoskeletal Disorders and Skin Diseases (NIAMS) and National Center for Advancing Translational Sciences, National Institutes of Health | 2 |
| JAMA | 5/16/2017 | Association Between Long-Lasting Intravitreous Fluocinolone Acetonide Implant vs Systemic Anti-inflammatory Therapy and Visual Acuity at 7 Years Among Patients With Intermediate, Posterior, or Panuveitis | National Eye Institute Collaborative Agreements U10EY014656 (Dr Altaweel), U10EY014660 (Dr Holbrook), and U10EY014655 (Dr Jabs). Additional support was provided by Research to Prevent Blindness, the Paul and Evanina Mackall Foundation, and the Lois Pope Life Foundation. Bausch & Lomb provided support to the study in the form of donation of fluocinolone acetonide implants for patients randomized to implant therapy who were uninsured or otherwise unable to pay | 2 |
| JAMA | 6/27/2017 | Effect of Acupuncture and Clomiphene in Chinese Women With Polycystic Ovary Syndrome A Randomized Clinical Trial | National Public Welfare Projects for Chinese Medicine, JC200804 from the Heilongjiang Province Foundation for Outstanding Youths, 2011TD006 from the Intervention for PCOS Based on Traditional Chinese Medicine Theory–TianGui Disorder, and JDZX2012036 and 2015B009 from 2009 through 2016 at the First Affiliated Hospital, Heilongjiang University of Chinese Medicine from the National Clinical Trial Base in Chinese Medicine Special Projects, by the National Key Discipline of Chinese Medicine in Gynecology from 2009 through 2016, by the Heilongjiang Province “Longjiang Scholar” Program, and by the Chinese “Thousand Talents Plan” scholarship | 2 |
| JAMA | 7/4/2017 | Effect of Radiofrequency Denervation on Pain Intensity Among Patients With Chronic Low Back Pain The Mint Randomized Clinical Trials | Netherlands Organization for Health Research and Development, by the Dutch Society for Anesthesiology, and the Dutch health insurance companies | 4 |
| JAMA | 8/1/2017 | Effect of Oral Methylprednisolone on Clinical Outcomes in Patients With IgA Nephropathy The TESTING Randomized Clinical Trial | National Health and Medical Research Council of Australia, the Peking University Health Central Clinical Research Project, and the Canadian Institutes of Health Research. Study drug was provided by Pfizer Pharmaceuticals | 3 |
| JAMA | 8/1/2017 | Effect of Endovascular Contact Aspiration vs Stent Retriever on Revascularization in Patients With Acute Ischemic Stroke and Large Vessel Occlusion The ASTER Randomized Clinical Trial | Fondation Ophtalmologique Adolphe de Rothschild. An unrestricted research grant was provided by Penumbra, Alameda, California | 3 |
| JAMA | 8/8/2017 | Effect of Cerebral Embolic Protection Devices on CNS Infarction in Surgical Aortic Valve Replacement A Randomized Clinical Trial | National Institute of Neurological Disorders and Stroke, the National Heart, Lung, and Blood Institute, and the Canadian Institutes for Health Research. Additional support was provided by the National Institutes of Health. Training in use of the Embol-X (Edwards Lifesciences) and CardioGard (CardioGard) devices was provided by the companies but they did not provide financial support for the study. | 3 |
| JAMA | 8/8/2017 | Effect of Levosimendan on Low Cardiac Output Syndrome in Patients With Low Ejection Fraction Undergoing Coronary Artery Bypass Grafting With Cardiopulmonary Bypass The LICORN Randomized Clinical Trial | French Ministry of Health (Programme Hospitalier de Recherche Clinique national 2011, MIN02-07) and sponsored by Assistance Publique-Hôpitaux de Paris. Orion Pharma provided study drugs free of charge | 3 |
| JAMA | 8/22/2017 | Effect of Natriuretic Peptide–Guided Therapy on Hospitalization or Cardiovascular Mortality in High-Risk Patients With Heart Failure and Reduced Ejection Fraction A Randomized Clinical Trial | National Institutes of Health, and Roche Diagnostics provided support for NT-proBNP testing | 3 |
| JAMA | 9/12/2017 | Effect of Axillary Dissection vs No Axillary Dissection on 10-Year Overall Survival Among Women With Invasive Breast Cancer and Sentinel Node Metastasis The ACOSOG Z0011 (Alliance) Randomized Clinical Trial | National Cancer Institute | 2 |
| JAMA | 9/26/2017 | Effect of Routine Low-Dose Oxygen Supplementation on Death and Disability in Adults With Acute Stroke The Stroke Oxygen Study Randomized Clinical Trial | NIHR Health Technology Assessment Programme and the Research for Patient Benefit Programme | 2 |
| JAMA | 10/3/2017 | Effect of an Early Resuscitation Protocol on In-hospital Mortality Among Adults With Sepsis and Hypotension A Randomized Clinical Trial | National Institutes of Health, the Fogarty International Center, and the National Center for Advancing Translational Sciences | 2 |
| JAMA | 10/10/2017 | Effect of Lung Recruitment and Titrated Positive End-Expiratory Pressure (PEEP) vs Low PEEP on Mortality in Patients With Acute Respiratory Distress Syndrome A Randomized Clinical Trial | Brazilian Ministry of Health | 2 |
| JAMA | 10/24/2017 | Effect of Robotic-Assisted vs Conventional Laparoscopic Surgery on Risk of Conversion to Open Laparotomy Among Patients Undergoing Resection for Rectal Cancer The ROLARR Randomized Clinical Trial | Efficacy and Mechanism Evaluation Programme, a Medical Research Council and National Institute for Health Research partnership with contributions from the Chief Scientist Office in Scotland, the National Institute for Social Care and Health Research in Wales, and the Health and Social Care Research and Development Division, Public Health Agency in Northern Ireland | 2 |
| JAMA | 11/7/2017 | Effect of a Single Dose of Oral Opioid and Nonopioid Analgesics on Acute Extremity Pain in the Emergency DepartmentA Randomized Clinical Trial | National Institute on Aging, National Institutes of Health. | 2 |
| JAMA | 11/21/2017 | Effect of Sertraline on Depressive Symptoms in Patients With Chronic Kidney Disease Without Dialysis DependenceThe CAST Randomized Clinical Trial | National Institute of Diabetes and Digestive and Kidney Diseases (NIDDK) and Department of Veterans Affairs MERIT grant CX000217-01 (awarded to Dr Hedayati). Support was also provided by the University of Texas Southwestern (UTSW) Medical Center O’Brien Kidney Research Core Center via NIDDK grant P30DK079328, grant UL1TR001105 from the Center for Translational Medicine, National Center for Advancing Translational Sciences of the National Institutes of Health (NIH), the Center for Depression Research and Clinical Care at UTSW, and grant K23MH104768 from the National Institute of Mental Health | 2 |
| JAMA | 4/12/2016 | Effect of a Quality Improvement Intervention With Daily Round Checklists, Goal Setting, and Clinician Prompting on Mortality of Critically Ill PatientsA Randomized Clinical Trial | Brazilian Health Surveillance Agency (ANVISA), PROADI, and Brazilian Development Bank (BNDES). D’Or Institute for Research and Education also contributed with additional funding. | 4 |
| JAMA | 5/3/2016 | Effect of Chemoradiotherapy vs Chemotherapy on Survival in Patients With Locally Advanced Pancreatic Cancer Controlled After 4 Months of Gemcitabine With or Without ErlotinibThe LAP07 Randomized Clinical Trial | Roche and French National Institute of Cancer | 3 |
| JAMA | 6/28/2016 | Effect of Escitalopram on All-Cause Mortality and Hospitalization in Patients With Heart Failure and DepressionThe MOOD-HF Randomized Clinical Trial | German Ministry of Education and Research and Lundbeck AS Denmark; institute of Clinical Epidemiology and Biometry, University of Würzburg, and the Clinical Trial Centre, University of Leipzig, received funding for biometry, statistical analyses, and study management | 3 |
| JAMA | 7/5/2016 | Effect of Palliative Care–Led Meetings for Families of Patients With Chronic Critical Illness A Randomized Clinical Trial | National Institute of Nursing Research | 2 |
| JAMA | 7/21/2016 | Effect of Patient Navigation With or Without Financial Incentives on Viral Suppression Among Hospitalized Patients With HIV Infection and Substance UseA Randomized Clinical Trial | National Institute on Drug Abuse, University of Miami Center for AIDS Research, Emory University CFAR, Atlanta Clinical and Translational Science Institute, HIV Center for Clinical and Behavioral Studies at the New York State Psychiatric Institute/Columbia University Medical Center | 2 |
| JAMA | 7/26/2016 | Effect of Radiosurgery Alone vs Radiosurgery With Whole Brain Radiation Therapy on Cognitive Function in Patients With 1 to 3 Brain MetastasesA Randomized Clinical Trial | NCI | 2 |
| JAMA | 9/6/2016 | Effect of Topical Intranasal Therapy on Epistaxis Frequency in Patients With Hereditary Hemorrhagic TelangiectasiaA Randomized Clinical Trial | Cure HHT | 2 |
| JAMA | 9/20/2016 | Effect of Wearable Technology Combined With a Lifestyle Intervention on Long-term Weight LossThe IDEA Randomized Clinical Trial | National Institutes of Health and the National Heart, Lung, and Blood Institute | 2 |
| JAMA | 11/8/2016 | Effect of Cranberry Capsules on Bacteriuria Plus Pyuria Among Older Women in Nursing HomesA Randomized Clinical Trial | National Institute on Aging, National Institutes of Health. Cranberry and placebo capsules used in this study were manufactured and donated by Pharmatoka | 3 |
| JAMA | 1/27/2015 | Chlorhexidine Bathing and Health Care–Associated InfectionsA Randomized Clinical Trial | National Institutes of Health and through the Vanderbilt Institute for Clinical and Translational Research | 2 |
| JAMA | 3/3/2015 | Effect of Sedative Premedication on Patient Experience After General AnesthesiaA Randomized Clinical Trial | French Institutional Clinical Hospital Research Program, Ministry of Health | 2 |
| JAMA | 3/10/2015 | Surgical vs Nonsurgical Treatment of Adults With Displaced Fractures of the Proximal HumerusThe PROFHER Randomized Clinical Trial | National Institute for Health Research, Health Technology Assessment Programme | 2 |
| JAMA | 4/28/2015 | Effect of a Retrievable Inferior Vena Cava Filter Plus Anticoagulation vs Anticoagulation Alone on Risk of Recurrent Pulmonary EmbolismA Randomized Clinical Trial | Programme Hospitalier de Recherche Clinique (French Department of Health), Fondation de l'Avenir and Fondation de France. Filters were packaged and provided free of charge by ALN Implants Chirurgicaux. The study sponsor was the University Hospital of Saint-Etienne | 3 |
| JAMA | 8/25/2015 | Effect of a 24-Month Physical Activity Intervention vs Health Education on Cognitive Outcomes in Sedentary Older AdultsThe LIFE Randomized Trial | National Institutes of Health, National Institute on Aging and 3U01AG022376-05A2S from the National Heart, Lung, and Blood Institute; and sponsored in part by the Intramural Research Program, National Institute on Aging, National Institutes of Health | 2 |
| JAMA | 8/25/2015 | Effect of Omega-3 Fatty Acids, Lutein/Zeaxanthin, or Other Nutrient Supplementation on Cognitive FunctionThe AREDS2 Randomized Clinical Trial | NIH | 2 |
| JAMA | 10/6/2015 | Rehabilitation After Immobilization for Ankle FractureThe EXACT Randomized Clinical Trial | National Health and Medical Research Council, Australia | 2 |
| JAMA | 10/20/2015 | Naproxen With Cyclobenzaprine, Oxycodone/Acetaminophen, or Placebo for Treating Acute Low Back PainA Randomized Clinical Trial | Montefiore Medical Center (clinical trials website) | 2 |
| JAMA | 12/15/2015 | Autologous Hematopoetic Stem Cell Transplantation for Refractory Crohn DiseaseA Randomized Clinical Trial | European Group for Blood and Marrow Transplantation (EBMT) Autoimmune Diseases Working Party and the European Crohn and Colitis Organisation | 2 |
| JAMA | 1/1/2014 | Effect of Prehospital Induction of Mild Hypothermia on Survival and Neurological Status Among Adults With Cardiac ArrestA Randomized Clinical Trial | National Heart, Lung, and Blood Institute and with additional support from the Medic One Foundation | 2 |
| JAMA | 1/1/2014 | Mechanical Chest Compressions and Simultaneous Defibrillation vs Conventional Cardiopulmonary Resuscitation in Out-of-Hospital Cardiac ArrestThe LINC Randomized Trial | Uppsala University and by Physio-Control/Jolife AB | 3 |
| JAMA | 3/26/2014 | Web-Based Alcohol Screening and Brief Intervention for University StudentsA Randomized Trial | Alcohol Advisory Council | 2 |
| JAMA | 5/14/2014 | Effect of PET Before Liver Resection on Surgical Management for Colorectal Adenocarcinoma MetastasesA Randomized Clinical Trial | Ontario Ministry of Health and Long-term Care to the Ontario Clinical Oncology Group | 2 |
| JAMA | 5/21/2014 | Effect of Physical Therapy on Pain and Function in Patients With Hip OsteoarthritisA Randomized Clinical Trial | National Health and Medical Research Council | 2 |
| JAMA | 5/28/2014 | Effect of Endoscopic Sphincterotomy for Suspected Sphincter of Oddi Dysfunction on Pain-Related Disability Following CholecystectomyThe EPISOD Randomized Clinical Trial | National Institutes of Diabetes and Digestive and Kidney Diseases | 2 |
| JAMA | 7/9/2014 | Effect of Postoperative Antibiotic Administration on Postoperative Infection Following Cholecystectomy for Acute Calculous CholecystitisA Randomized Clinical Trial | French Ministry of Health's Programme Hospitalier de Recherche Clinique 2009 program | 2 |
| JAMA | 7/16/2014 | Effects of Hydroxychloroquine on Symptomatic Improvement in Primary Sjögren SyndromeThe JOQUER Randomized Clinical Trial | Assistance Publique-Hôpitaux de Paris, with a grant from the French Ministry of Research (Programme Hospitalier de Recherche Clinique National 2007 P070125). sanofi-aventis provided hydroxychloroquine and placebo | 2 |
| JAMA | 8/6/2014 | Brief Intervention for Problem Drug Use in Safety-Net Primary Care SettingsA Randomized Clinical Trial | National Institute on Drug Abuse | 2 |
| JAMA | 8/6/2014 | Screening and Brief Intervention for Drug Use in Primary CareThe ASPIRE Randomized Clinical Trial | National Institute on Drug Abuse (R01 DA025068), a portion of which was funded by the Center for Substance Abuse Treatment, SAMHSA. BNI counselors, training, and supervision were supported by a contract from the SAMHSA to the Massachusetts Department of Public Health Bureau of Substance Abuse Services, which contracted with Boston Medical Center to deliver BNI services. The study was also supported in part by the National Center for Research Resources | 2 |
| JAMA | 10/1/2014 | Acupuncture for Chronic Knee Pain A Randomized Clinical Trial | National Health and Medical Research Council | 2 |
| JAMA | 12/3/2014 | Effect of Screening for Coronary Artery Disease Using CT Angiography on Mortality and Cardiac Events in High-Risk Patients With DiabetesThe FACTOR-64 Randomized Clinical Trial | Intermountain Research and Medical Foundation, Intermountain Healthcare Urban Central Region, Salt Lake City, Utah, and the Intermountain Heart Institute Department of Cardiovascular Research, Toshiba Corporation and Bracco Corporation | 3 |
| JAMA | 12/3/2014 | Effect of Fenoldopam on Use of Renal Replacement Therapy Among Patients With Acute Kidney Injury After Cardiac SurgeryA Randomized Clinical Trial | Italian Ministry of Health | 2 |
| JAMA | 12/17/2014 | Low-Dose Aspirin for Primary Prevention of Cardiovascular Events in Japanese Patients 60 Years or Older With Atherosclerotic Risk FactorsA Randomized Clinical Trial | Japanese Ministry of Health, Labor, and Welfare and the Waksman Foundation of Japan. aspirin tablets were provided free of charge by Bayer Yakuhin | 3 |
| JAMA | 1/2/2013 | Effect of Maintenance Tocolysis With Nifedipine in Threatened Preterm Labor on Perinatal OutcomesA Randomized Controlled Trial | ZonMw, the Netherlands Organization for Health Research and Development Healthcare Efficiency Program | 2 |
| JAMA | 2/6/2013 | Effect of Corticosteroid Injection, Physiotherapy, or Both on Clinical Outcomes in Patients With Unilateral Lateral EpicondylalgiaA Randomized Controlled Trial | Australian National Health and Medical Research Council | 2 |
| JAMA | 5/22/2013 | Effect of Early vs Late Tracheostomy Placement on Survival in Patients Receiving Mechanical VentilationThe TracMan Randomized Trial | UK Intensive Care Society and the Medical Research Council | 2 |
| JAMA | 5/22/2013 | Early Parenteral Nutrition in Critically Ill Patients With Short-term Relative Contraindications to Early Enteral NutritionA Randomized Controlled Trial | Australian National Health and Medical Research Council (NH&MRC) and unrestricted academic grants from Fresenius Kabi Deutschland and Baxter Healthcare. Fresenius Kabi Australia supplied the study parenteral nutrition | 3 |
| JAMA | 7/10/2013 | Effect of Soy Protein Isolate Supplementation on Biochemical Recurrence of Prostate Cancer After Radical ProstatectomyA Randomized Trial | National Institute of Health grants, with minor support from the Prevent Cancer Foundation and the United Soybean Board. Solae LLC provided the intervention materials | 3 |
| JAMA | 10/2/2013 | Menopausal Hormone Therapy and Health Outcomes During the Intervention and Extended Poststopping Phases of the Women’s Health Initiative Randomized Trials | National Heart, Lung, and Blood Institute | 2 |
| JAMA | 10/16/2013 | Universal Glove and Gown Use and Acquisition of Antibiotic-Resistant Bacteria in the ICUA Randomized Trial | Agency for Healthcare Research and Quality (AHRQ), and the National Institutes of Health | 2 |
| JAMA | 10/23/2013 | Effect of Risk-Reduction Counseling With Rapid HIV Testing on Risk of Acquiring Sexually Transmitted InfectionsThe AWARE Randomized Clinical Trial | National Institute on Drug Abuse. The infrastructure of the National Drug Abuse Treatment Clinical Trials Network was used as a platform in conducting this trial. Support from the University of Miami Center for AIDS Research | 2 |
| JAMA | 11/6/2013 | Effects of Fluid Resuscitation With Colloids vs Crystalloids on Mortality in Critically Ill Patients Presenting With Hypovolemic ShockThe CRISTAL Randomized Trial | French Ministry of Health, Programme Hospitalier de Recherche Clinique | 2 |
| JAMA | 12/4/2013 | Effect of Communication Skills Training for Residents and Nurse Practitioners on Quality of Communication With Patients With Serious IllnessA Randomized Trial | National Institute of Nursing Research of the National Institutes of Health | 2 |
| JAMA | 12/25/2013 | Effect of Nortriptyline on Symptoms of Idiopathic GastroparesisThe NORIG Randomized Clinical Trial | National Institute of Diabetes and Digestive and Kidney Diseases | 2 |
| JAMA | 1/25/2012 | Lansoprazole for Children With Poorly Controlled Asthma A Randomized Controlled Trial | American Lung Association Asthma Clinical Research Centers Infrastructure Award and National Institutes of Health/National Heart, Lung, and Blood Institute. Lansoprazole and placebo were provided by Takeda Pharmaceuticals; albuterol was provided by GlaxoSmithKline | 2 |
| JAMA | 2/15/2012 | Amoxicillin for Acute Rhinosinusitis A Randomized Controlled Trial | National Institute of Allergy and Infectious Diseases | 2 |
| JAMA | 5/2/2012 | Intracoronary Abciximab and Aspiration Thrombectomy in Patients With Large Anterior Myocardial Infarction The INFUSE-AMI Randomized Trial | Atrium Medical. Atrium supplied the local drug delivery catheter. Aspiration catheters were provided at a discount by Medtronic. Bivalirudin was provided at no charge by The Medicines Company. | 1 |
| JAMA | 5/23/2012 | Effect of Continuous Positive Airway Pressure on the Incidence of Hypertension and Cardiovascular Events in Nonsleepy Patients With Obstructive Sleep Apnea A Randomized Controlled Trial | Instituto de Salud Carlos III (PI 04/0165) (Fondo de Investigaciones Sanitarios, Ministerio de Sanidad y Consumo, Spain), Spanish Respiratory Society (SEPAR) (Barcelona), Resmed (Bella Vista, Australia), Air Products–Carburos Metalicos (Barcelona), Respironics (Murrysville, Pennsylvania), and Breas Medical | 3 |
| JAMA | 7/18/2012 | Effect of Silymarin (Milk Thistle) on Liver Disease in Patients With Chronic Hepatitis C Unsuccessfully Treated With Interferon Therapy A Randomized Controlled Trial | National Institutes of Health (NIH) National Center for Complementary and Alternative Medicine, National Institute of Diabetes and Digestive and Kidney Diseases (NIDDK); and with support from the NIH Clinical & Translational Sciences Awards Division of Research Resources, Rottapharm Madaus (Monza, Italy, and Cologne, Germany) donated the silymarin study medication and matching placebo. Abbott Molecular Inc (Des Plaines, Illinois) donated the Abbott RealTime HCV assays | 3 |
| JAMA | 8/15/2012 | Effect of Screening for Partner Violence on Women's Quality of Life A Randomized Controlled Trial | Centers for Disease Control and Prevention (CDC), National Center for Injury Prevention and Control, Division of Violence Prevention | 2 |
| JAMA | 11/7/2012 | Multivitamins in the Prevention of Cardiovascular Disease in Men The Physicians' Health Study II Randomized Controlled Trial | NIH. BASF Corporation and Pfizer (formerly Wyeth, American Home Products, and Lederle); and DSM Nutritional Products Inc (formerly Roche Vitamins) | 3 |
| JAMA | 11/7/2012 | Intraoperative High-Dose Dexamethasone for Cardiac Surgery A Randomized Controlled Trial | Netherlands Organization for Health Research and Development (ZonMw) and 2007B125 from the Dutch Heart Foundation | 2 |
| JAMA | 11/21/2012 | Effect of Citicoline on Functional and Cognitive Status Among Patients With Traumatic Brain Injury Citicoline Brain Injury Treatment Trial (COBRIT) | National Institute of Child Health and Human Development grants. Ferrer Grupo provided the citicoline and identical placebo | 3 |
| JAMA | 11/21/2012 | Fish Oil and Postoperative Atrial Fibrillation The Omega-3 Fatty Acids for Prevention of Post-operative Atrial Fibrillation (OPERA) Randomized Trial | National Heart, Lung, and Blood Institute, National Institutes of Health (RC2-HL101816), GlaxoSmithKline, Sigma Tau, and Pronova BioPharma | 3 |
| JAMA | 1/12/2011 | Behavioral Therapy With or Without Biofeedback and Pelvic Floor Electrical Stimulation for Persistent Postprostatectomy Incontinence A Randomized Controlled Trial | National Institute of Diabetes and Digestive and Kidney Diseases and by the Department of Veterans Affairs Birmingham–Atlanta Geriatric Research, Education, and Clinical Center | 2 |
| JAMA | 8/3/2011 | Adjunctive Risperidone Treatment for Antidepressant-Resistant Symptoms of Chronic Military Service–Related PTSDA Randomized Trial | Cooperative Studies Program of the Department of Veterans Affairs Office of Research and Development. Risperidone and matching placebo were donated to the VA Cooperative Studies Program Clinical Research Pharmacy Coordinating Center (Albuquerque, New Mexico) by Ortho-McNeil Janssen Scientific Affairs. Ortho-McNeil Janssen Scientific Affairs also contributed a total of $606 219 to the Baltimore Research and Education Foundation on behalf of the VA Cooperative Studies Program | 3 |
| JAMA | 9/28/2011 | Effect of Increasing Doses of Saw Palmetto Extract on Lower Urinary Tract Symptoms A Randomized Trial | National Institutes of Health (NIH), National Institute of Diabetes and Digestive and Kidney Diseases. Support was also provided by the National Center for Complementary and Alternative Medicine and the Office of Dietary Supplements, NIH. Saw palmetto fruit extract and matching placebo were donated by Rottapharm/Madaus, Cologne, Germany | 3 |
| JAMA | 10/12/2011 | Vitamin E and the Risk of Prostate Cancer The Selenium and Vitamin E Cancer Prevention Trial (SELECT) | National Cancer Institute, National Institutes of Health, Department of Health and Human Services, and in part by the National Center for Complementary and Alternative Medicine (National Institutes of Health). Study agents and packaging were provided by Perrigo Co, Allegan, Michigan; Sabinsa Corp, Piscataway, New Jersey; Tishcon Corp, Westbury, New York; and DSM Nutritional Products Inc | 3 |
| JAMA | 10/12/2011 | Enteral Omega-3 Fatty Acid, γ-Linolenic Acid, and Antioxidant Supplementation in Acute Lung Injury | National Heart, Lung, and Blood Institute (NHLBI). Abbott Nutrition, Columbus, Ohio, provided the omega-3 fatty acid, γ-linolenic acid, antioxidant, and control supplements | 3 |
| JAMA | 1/13/2010 | Platelet-Rich Plasma Injection for Chronic Achilles Tendinopathy A Randomized Controlled Trial | Biomet Biologics LLC | 1 |
| JAMA | 1/27/2010 | Corticosteroid Treatment and Intensive Insulin Therapy for Septic Shock in Adults: A Randomized Controlled Trial | Assistance Publique–Hôpitaux de Paris | 2 |
| JAMA | 4/21/2010 | Early vs Late Tracheotomy for Prevention of Pneumonia in Mechanically Ventilated Adult ICU Patients: A Randomized Controlled Trial | Regione Piemonte Ricerca Sanitaria Finalizzata grant 03-08/ACR ASx44 | 2 |
| JAMA | 5/12/2010 | Annual High-Dose Oral Vitamin D and Falls and Fractures in Older Women: A Randomized Controlled Trial | National Health and Medical Research Council and by the Australian Government Department of Health and Ageing. | 2 |
| JAMA | 7/7/2010 | Effect of Glucosamine on Pain-Related Disability in Patients With Chronic Low Back Pain and Degenerative Lumbar Osteoarthritis: A Randomized Controlled Trial | Norwegian Foundation for Health and Rehabilitation through the Norwegian Low Back Pain Association, Norwegian Chiropractic Associations Research Fund, and Wilhelmsens Research Fund. The study medications (glucosamine and placebo) were produced by and purchased from Pharma Nord. Pharma Nord was chosen as the study agent supplier after all companies with marketing approvals in Norway were asked if they wanted to contribute with their product to the trial. | 2 |
| JAMA | 8/25/2010 | Elective Intra-aortic Balloon Counterpulsation During High-Risk Percutaneous Coronary Intervention: A Randomized Controlled Trial | British Cardiovascular Intervention Society, which in turn received funding from Maquet Cardiovascular (previously Datascope) (Mawah, New Jersey), Cordis, and Johnson & Johnson (Wokingham, United Kingdom) by way of unrestricted educational grants, and by Lilly (Hampshire, United Kingdom), which provided abciximab for use in the trial. | 3 |
| JAMA | 10/13/2010 | Transfusion Requirements After Cardiac SurgeryThe TRACS Randomized Controlled Trial | Instituto do Coracao | 2 |
| JAMA | 10/20/2010 | Effect of DHA Supplementation During Pregnancy on Maternal Depression and Neurodevelopment of Young Children: A Randomized Controlled Trial | Australian National Health and Medical Research Council | 2 |
| JAMA | 11/3/2010 | Docosahexaenoic Acid Supplementation and Cognitive Decline in Alzheimer Disease: A Randomized Trial | National Institute on Aging and Martek Biosciences | 3 |
| JAMA | 12/1/2010 | Efficacy and Safety of Prescription Omega-3 Fatty Acids for the Prevention of Recurrent Symptomatic Atrial Fibrillation: A Randomized Controlled Trial | GlaxoSmithKline | 1 |
| JAMA | 12/22/2010 | Weight Lifting for Women at Risk for Breast Cancer–Related Lymphedema: A Randomized Trial | National Cancer Institute, National Institutes of Health to the University of Pennsylvania, and BSN Medical | 3 |
| JAMA | 01/07/2009 | Effect of Selenium and Vitamin E on Risk of Prostate Cancer and Other Cancers The Selenium and Vitamin E Prevention Trial (SELECT) | National Cancer Institute, National Institutes of Health, Department of Health and Human Services, and in part by the National Center for Complementary and Alternative Medicine (National Institutes of Health), Perrigo Company, Sabinsa Corporation, Trishcon Corportaion, and DSM Nutritional Producs Inc | 3 |
| JAMA | 01/07/2009 | Vitamins E and C in the Prevention of Prostate and Total Cancer in MenThe Physicians' Health Study II Randomized Controlled Trial | National Institutes of Health, BASF Corporation, Wyeth Pharmaceuticals, and DSM Nutritional Producs Inc | 3 |
| JAMA | 04/15/2009 | Cardiac Outcomes After Screening for Asymptomatic Coronary Artery Disease in Patients With Type 2 Diabetes The DIAD Study: A Randomized Controlled Trial | National Institute of Health, Bristol Myers-Squibb Medical Imaging | 3 |
| JAMA | 09/02/2009 | Laparoscopic Uterosacral Nerve Ablation for Alleviating Chronic Pelvic Pain A Randomized Controlled Trial | Wellbeing for Women charity, Birmingham Women's Foundation NHS Trust Research and Development Committee, Birmingham Clinical Trials Unit, UK Department of Health | 2 |
| JAMA | 10/14/2009 | Effect of High Perioperative Oxygen Fraction on Surgical Site Infection and Pulmonary Complications After Abdominal Surgery The PROXI Randomized Clinical Trial | Danish Medical Research Council, Lundbeck Foundation, Rigshopitalet's Research Council, Novo Nordisk Foundation, Aase and Ejnar Danielsens Foundation, A.P. Moller Foundation, Danish Society of Anaesthesiology, Intensive Care Medicine's Research Initiative, Beckett-Foundation, Brodrene Hartmanns Foundation, Etly and Jordgen Stjerngrens Foundation | 3 |
| JAMA | 11/04/2009 | Surgical Mask vs N95 Respirator for Preventing Influenza Among Health Care Workers: A Randomized trial | Public Health Agency of Canada | 2 |
| JAMA | 11/11/2009 | Prone Positioning in Patients With Moderate and Severe Acute Respiratory Distress Syndrome: A Randomized Controlled Trial | Programme Hospitalier de Recherche Clinique National 2006 and 2010 of the French Ministry of Health | 2 |
| JAMA | 11/25/2009 | Intravenous Drug Administration During Out-of-Hospital Cardiac Arrest: A Randomized Trial | Eastern Norway Regional Health Authority, Oslo University Hospital, Norwegian Air Ambulance Foundation, Laerdal Foundation for Acute Medicine, and the Anders Jahres Fund | 2 |
| JAMA | 12/02/2009 | Effectiveness of Public Report Cards for Improving the Quality of Cardiac CareThe EFFECT Study: A Randomized Trial | Canadian Institutes of Health Research | 2 |
| JAMA | 12/23/2009 | Ginkgo biloba for Preventing Cognitive Decline in Older Adults: A Randomized Trial | National Institute on Aging, National Heart, Lung, and Blood Institute, University of Pittsburgh Alzheimer's Disease Research Center (grant P50AG05133), Roena Kulynych Center for Memory and Cognition Research, and National Institute of Neurological Disorders and Stroke, Schwabe Pharmaceuticals | 3 |
| JAMA | 1/2/2008 | Effect of Testosterone Supplementation on Functional Mobility, Cognition, and Other Parameters in Older Men A Randomized Controlled Trial | Netherlands Organization for Health Research and Development, Organon NV (Oss, the Netherlands) | 3 |
| JAMA | 04/09/2008 | Omega-3 Free Fatty Acids for the Maintenance of Remission in Crohn Disease: The EPIC Randomized Controlled Trials | Tillotts Pharma | 1 |
| JAMA | 05/28/2008 | Femoral vs Jugular Venous Catheterization and Risk of Nosocomial Events in Adults Requiring Acute Renal Replacement Therapy: A Randomized Controlled Trial | Centre Hospitalier Universitaire de Caen, French Health Ministry, MEDA Pharma | 3 |
| JAMA | 06/11/2008 | Hypericum perforatum (St John's Wort) for Attention-Deficit/Hyperactivity Disorder in Children and AdolescentsA Randomized Controlled Trial | National Center for Complementary and Alternative Medicine, Vital Nutrients Inc. | 3 |
| JAMA | 08/20/2008 | Mortality and Cardiovascular Events in Patients Treated With Homocysteine-Lowering B Vitamins After Coronary Angiography: A Randomized Controlled Trial | Norwegian Foundation for Health and Rehabilitation, the Norwegian Heart and Lung Patient Organisation, the Norwegian Ministry of Health and Care Services, the Western Norway Regional Health Authority, the Department of Heart Disease at Haukeland University Hospital, Locus for Homocysteine and Related Vitamins at the University of Bergen, Locus for Cardiac Research at the University of Bergen, the Foundation to Promote Research Into Functional Vitamin B12 Deficiency, Bergen, Norway, and Alpharma Inc | 2 |
| JAMA | 10/08/2008 | Serial 2-Point Ultrasonography Plus D-Dimer vs Whole-Leg Color-Coded Doppler Ultrasonography for Diagnosing Suspected Symptomatic Deep Vein Thrombosis A Randomized Controlled Trial | Società Italiana per lo Studio dell’Emostasi e della Trombosi | 2 |
| JAMA | 11/05/2008 | Effect of Combined Folic Acid, Vitamin B6, and Vitamin B12 on Cancer Risk in Women: A Randomized Trial | National Heart, Lung, and Blood Institute of the National Institutes of Health. | 2 |
| JAMA | 11/12/2008 | Low-Dose Aspirin for Primary Prevention of Atherosclerotic Events in Patients With Type 2 Diabetes:A Randomized Controlled Trial | Ministry of Health, Labour and Welfare of Japan | 2 |
| JAMA | 11/12/2008 | Vitamins E and C in the Prevention of Cardiovascular Disease in Men The Physicians' Health Study II Randomized Controlled Trial | National Institutes of Health,BASF Corporation, Wyeth Pharmaceuticals, DSM Nutritional Products Inc | 3 |
| JAMA | 11/19/2008 | Ginkgo biloba for Prevention of Dementia A Randomized Controlled Trial | National Center for Complementary and Alternative Medicine (NCCAM), National Institute on Aging; National Heart, Lung, and Blood Institute; University of Pittsburgh Alzheimer's Disease Research Center (P50AG05133); Roena Kulynych Center for Memory and Cognition Research; Wake Forest University School of Medicine; and National Institute of Neurological Disorders and Stroke. | 2 |
| JAMA | 12/17/2008 | Effect of Evidence-Based Feeding Guidelines on Mortality of Critically Ill Adults A Cluster Randomized Controlled Trial | Australian and New Zealand Intensive Care (ANZIC) Foundation, Novartis and Abbott Laboratories and significant support from Nutricia, Fresenius-Kabi, and Baxter | 3 |
| JAMA | 2/21/2007 | Cognitive and Cardiac Outcomes 5 Years After Off-Pump vs On-Pump Coronary Artery Bypass Graft Surgery | International Anesthesia Research Society (IARS). | 2 |
| JAMA | 05/02/2007 | Levosimendan vs Dobutamine for Patients With Acute Decompensated Heart Failure The SURVIVE Randomized Trial | Abbott and Orion Pharma | 1 |
| JAMA | 07/18/2007 | Influence of a Diet Very High in Vegetables, Fruit, and Fiber and Low in Fat on Prognosis Following Treatment for Breast Cancer The Women's Healthy Eating and Living (WHEL) Randomized Trial | Walton Family Foundation and National Cancer Institute | 2 |
| JAMA | 07/25/2007 | Efficacy of a Hip Protector to Prevent Hip Fracture in Nursing Home Residents The HIP PRO Randomized Controlled Trial | National Institutes of Health, National Institute on Aging, Lawrence J. and Anne Cable Rubenstein Charitable Foundation. | 2 |
| JAMA | 12/05/2007 | Antibiotics and Topical Nasal Steroid for Treatment of Acute Maxillary Sinusitis A Randomized Controlled Trial | UK Department of Health | 2 |
| JAMA | 02/08/2006 | Low-Fat Dietary Pattern and Risk of Colorectal Cancer The Women's Health Initiative Randomized Controlled Dietary Modification Trial | National Heart, Lung, and Blood Institute, US Department of Health and Human Services. | 2 |
| JAMA | 02/08/2006 | Low-Fat Dietary Pattern and Risk of Cardiovascular Disease The Women's Health Initiative Randomized Controlled Dietary Modification Trial | US Department of Health and Human Services | 2 |
| JAMA | 05/17/2006 | Effect of Policosanol on Lipid Levels Among Patients With Hypercholesterolemia or Combined Hyperlipidemia A Randomized Controlled Trial | Madaus AG | 1 |
| JAMA | 06/14/2006 | Fluoxetine After Weight Restoration in Anorexia Nervosa A Randomized Controlled Trial | National Institutes of Health, Eli Lilly | 3 |
| JAMA | 06/14/2006 | Manual Chest Compression vs Use of an Automated Chest Compression Device During Resuscitation Following Out-of-Hospital Cardiac Arrest: A Randomized Trial | Revivant Corporation | 1 |
| JAMA | 06/28/2006 | Cognitive Behavioral Therapy vs Zopiclone for Treatment of Chronic Primary Insomnia in Older Adults A Randomized Controlled Trial | University of Bergen, the Meltzer Fund, and the EXTRA funds from the Norwegian Foundation for Health and Rehabilitation | 2 |
| JAMA | 03/02/2005 | Distal Microcirculatory Protection During Percutaneous Coronary Intervention in Acute ST-Segment Elevation Myocardial Infarction A Randomized Controlled Trial | Medtronic Corp | 1 |
| JAMA | 03/16/2005 | Effects of Long-term Vitamin E Supplementation on Cardiovascular Events and Cancer A Randomized Controlled Trial | Medical Research Council of Canada, Hoechst-Marion Roussel, AstraZeneca, King Pharmaceuticals, Natural Source Vitamin E Association and Negma, and the Heart and Stroke Foundation of Ontario. Aventis Pharmaceuticals, King Pharmaceuticals, and the Natural Source Vitamin E Association | 3 |
| JAMA | 6/22/2005 | Information Leaflet and Antibiotic Prescribing Strategies for Acute Lower Respiratory Tract Infection A Randomized Controlled Trial | Medical Research Council | 2 |
| JAMA | 07/06/2005 | Vitamin E in the Primary Prevention of Cardiovascular Disease and CancerThe Women’s Health Study: A Randomized Controlled Trial | National Heart, Lung, and Blood Institute and the National Cancer Institute, Natural Source Vitamin E Association, Bayer Healthcare | 3 |
| JAMA | 08/24/2005 | Anesthesia-Assisted vs Buprenorphine- or Clonidine-Assisted Heroin Detoxification and Naltrexone Induction A Randomized Trial | National Institute on Drug Abuse (NIDA) and the National Institutes of Health (NIH). | 2 |
| JAMA | 10/05/2005 | Evaluation Study of Congestive Heart Failure and Pulmonary Artery Catheterization Effectiveness The ESCAPE Trial | National Heart, Lung, and Blood Institute to Duke University Medical Center | 2 |
| JAMA | 01/14/2004 | Electrocardiographic and Hemodynamic Effects of a Multicomponent Dietary Supplement Containing Ephedra and Caffeine A Randomized Controlled Trial |  | NI |
| JAMA | 03/17/2004 | Prehospital Hypertonic Saline Resuscitation of Patients With Hypotension and Severe Traumatic Brain Injury A Randomized Controlled Trial |  | NI |
| JAMA | 04/07/2004 | Low-Dose Inhaled Nitric Oxide in Patients With Acute Lung Injury A Randomized Controlled Trial |  | NI |
| JAMA | 04/14/2004 | Effects of Conjugated Equine Estrogen in Postmenopausal Women With Hysterectomy The Women's Health Initiative Randomized Controlled Trial | National Heart, Lung, and Blood Institute, US Department of Health and Human Services. | 2 |
| JAMA | 6/23/2004 | Conjugated Equine Estrogens and Incidence of Probable Dementia and Mild Cognitive Impairment in Postmenopausal WomenWomen's Health Initiative Memory Study | National Heart, Lung, and Blood Institute, US Department of Health and Human Services. | 2 |
| JAMA | 6/23/2004 | Conjugated Equine Estrogens and Global Cognitive Function in Postmenopausal Women Women's Health Initiative Memory Study | National Heart, Lung, and Blood Institute, US Department of Health and Human Services. | 2 |
| JAMA | 11/17/2004 | Effects of Systematic Prone Positioning in Hypoxemic Acute Respiratory Failure A Randomized Controlled Trial |  | NI |
| JAMA | 04/16/2003 | Prevention of Hip Fractures by External Hip Protectors A Randomized Controlled Trial |  | NI |
| JAMA | 05/07/2003 | Pacemaker Therapy for Prevention of Syncope in Patients With Recurrent Severe Vasovagal Syncope Second Vasovagal Pacemaker Study (VPS II): A Randomized Trial |  | NI |
| JAMA | 05/28/2003 | Estrogen Plus Progestin and the Incidence of Dementia and Mild Cognitive Impairment in Postmenopausal Women The Women's Health Initiative Memory Study: A Randomized Controlled Trial | National Heart, Lung, and Blood Institute, US Department of Health and Human Services. | 2 |
| JAMA | 05/28/2003 | Effect of Estrogen Plus Progestin on Global Cognitive Function in Postmenopausal Women The Women's Health Initiative Memory Study: A Randomized Controlled Trial | National Heart, Lung, and Blood Institute, US Department of Health and Human Services. | 2 |
| JAMA | 06/04/2003 | Rapid Magnetic Resonance Imaging vs Radiographs for Patients With Low Back Pain A Randomized Controlled Trial |  | NI |
| JAMA | 05/28/2003 | Effect of Estrogen Plus Progestin on Stroke in Postmenopausal Women The Women's Health Initiative: A Randomized Trial | National Heart, Lung, and Blood Institute, US Department of Health and Human Services. | 2 |
| JAMA | 06/25/2003 | Influence of Estrogen Plus Progestin on Breast Cancer and Mammography in Healthy Postmenopausal Women The Women's Health Initiative Randomized Trial | National Heart, Lung, and Blood Institute, US Department of Health and Human Services. | 2 |
| JAMA | 07/16/2003 | Effect of Behavioral Training With or Without Pelvic Floor Electrical Stimulation on Stress Incontinence in Women: A Randomized Controlled Trial |  | NI |
| JAMA | 7/23/2003 | Incidence of Cancer and Mortality Following α-Tocopherol and β-Carotene Supplementation A Postintervention Follow-up | U.S. National Cancer Institute (NCI) and the National Institute for Health and Welfare of Finland | 2 |
| JAMA | 08/13/2003 | Guggulipid for the Treatment of Hypercholesterolemia A Randomized Controlled Trial |  | NI |
| JAMA | 09/17/2003 | Effect of Magnetic vs Sham-Magnetic Insoles on Plantar Heel Pain: A Randomized Controlled Trial | "All insoles (active and inactive) were provided by the manufacturer to the investigators at no charge", but general funding not specified | NI |
| JAMA | 10/01/2003 | Effects of Estrogen Plus Progestin on Gynecologic Cancers and Associated Diagnostic ProceduresThe Women's Health Initiative Randomized Trial | National Heart, Lung, and Blood Institute, US Department of Health and Human Services. | 2 |
| JAMA | 10/8/2003 | Treatment of Corticosteroid-Responsive Autoimmune Inner Ear Disease With MethotrexateA Randomized Controlled Trial |  | NI |
| JAMA | 11/05/2003 | Fenoldopam Mesylate for the Prevention of Contrast-Induced Nephropathy A Randomized Controlled Trial |  | NI |
| JAMA | 11/26/2003 | Early Use of the Pulmonary Artery Catheter and Outcomes in Patients With Shock and Acute Respiratory Distress Syndrome A Randomized Controlled Trial |  | NI |
| JAMA | 12/03/2003 | Efficacy and Safety of Echinacea in Treating Upper Respiratory Tract Infections in Children A Randomized Controlled Trial |  | NI |
| JAMA | 12/10/2003 | Combined Levothyroxine Plus Liothyronine Compared With Levothyroxine Alone in Primary Hypothyroidism A Randomized Controlled Trial |  | NI |
| NEJM | 2/9/2017 | Thromboprophylaxis after Knee Arthroscopy and Lower-Leg Casting | Netherlands Organization for Health Research and Development | 2 |
| NEJM | 2/23/2017 | Tight Glycemic Control in Critically Ill Children | National Heart, Lung, and Blood Institute, National Institutes of Health, | 2 |
| NEJM | 3/2/2017 | Treatment of Subclinical Hypothyroidism or Hypothyroxinemia in Pregnancy | Eunice Kennedy Shriver National Institute of Child Health and Human Development and the National Institute of Neurological Disorders and Stroke | 2 |
| NEJM | 3/23/2017 | Trial of Pregabalin for Acute and Chronic Sciatica | National Health and Medical Research Council of Australia. | 2 |
| NEJM | 5/25/2017 | Levosimendan for Hemodynamic Support after Cardiac Surgery | Italian Ministry of Health | 2 |
| NEJM | 5/25/2017 | Levosimendan in Patients with Left Ventricular Dysfunction Undergoing Cardiac Surgery | Tenax Therapeutics | 1 |
| NEJM | 6/15/2017 | Bioresorbable Scaffolds versus Metallic Stents in Routine PCI | Abbott Vascular | 1 |
| NEJM | 6/29/2017 | Thyroid Hormone Therapy for Older Adults with Subclinical Hypothyroidism | European Union, Swiss National Science Foundation, Swiss Heart Foundation, Velux Stiftung | 2 |
| NEJM | 8/17/2017 | Five-Year Outcomes after On-Pump and Off-Pump Coronary-Artery Bypass | Department of Veterans Affairs (VA) Office of Research and Development Cooperative Studies Program (CSP) | 2 |
| NEJM | 9/21/2017 | Bivalirudin versus Heparin Monotherapy in Myocardial Infarction | Swedish Heart–Lung Foundation, the Swedish Research Council, AstraZeneca and the Medicines Company, and the Swedish Foundation for Strategic Research | 3 |
| NEJM | 9/28/2017 | Oxygen Therapy in Suspected Acute Myocardial Infarction | Swedish Heart–Lung Foundation, the Swedish Research Council, and the Swedish Foundation for Strategic Research | 3 |
| NEJM | 11/16/2017 | Lomustine and Bevacizumab in Progressive Glioblastoma | F. Hoffmann–La Roche and by the EORTC Cancer Research Fund | 3 |
| NEJM | 12/7/2017 | Pharmacomechanical Catheter-Directed Thrombolysis for Deep-Vein Thrombosis | National Heart, Lung, and Blood Institute (NHLBI) | 2 |
| NEJM | 12/14/2017 | Outcomes of a Coaching-Based WHO Safe Childbirth Checklist Program in India | Bill and Melinda Gates Foundation | 2 |
| NEJM | 12/21/2017 | PCI Strategies in Patients with Acute Myocardial Infarction and Cardiogenic Shock | European Union 7th Framework Program and by the German Heart Research Foundation and the German Cardiac Society | 2 |
| NEJM | 12/21/2017 | Delayed versus Immediate Cord Clamping in Preterm Infants | National Health and Medical Research Council (NHMRC) | 2 |
| NEJM | 2/4/2016 | Routine Amoxicillin for Uncomplicated Severe Acute Malnutrition in Children | Médecins sans Frontières Operational Center Paris | 2 |
| NEJM | 2/11/2016 | Adjunctive Dexamethasone in HIV-Associated Cryptococcal Meningitis | United Kingdom Department for International Development, the Wellcome Trust, and the Medical Research Council | 2 |
| NEJM | 3/17/2016 | A Randomized Trial of a Cervical Pessary to Prevent Preterm Singleton Birth | Fetal Medicine Foundation | 2 |
| NEJM | 3/24/2016 | Early versus Late Parenteral Nutrition in Critically Ill Children | Flemish Government, the European Research Council under the European Union’s Seventh Framework Program, a grant from Fonds NutsOhra, an Erasmus MC Cost-Effectiveness Research Grant to Dr. Verbruggen; and a grant from the Erasmus Trustfonds through Erasmus University Rotterdam | 2 |
| NEJM | 3/31/2016 | Randomized Trial of Longer-Term Therapy for Symptoms Attributed to Lyme Disease | Netherlands Organization for Health Research and Development ZonMw | 2 |
| NEJM | 4/14/2016 | A Randomized, Controlled Trial of Fusion Surgery for Lumbar Spinal Stenosis | Avtal om Läkarutbildning och Forskning, Johnson & Johnson, The National Swedish Register for Spine Surgery (Swespine) | 3 |
| NEJM | 4/14/2016 | Effect of Avoidance on Peanut Allergy after Early Peanut Consumption | National Institute of Allergy and Infectious Diseases of the National Institutes of Health | 2 |
| NEJM | 5/5/2016 | Amiodarone, Lidocaine, or Placebo in Out-of-Hospital Cardiac Arrest | NHLBI | 2 |
| NEJM | 5/5/2016 | Perioperative Rosuvastatin in Cardiac Surgery | British Heart Foundation, the European Network for Translational Research in Atrial Fibrillation of the European Commission Seventh Framework Program, the Oxford Biomedical Research Centre, and the U.K. Medical Research Council and by a small unrestricted grant from AstraZeneca. | 3 |
| NEJM | 5/19/2016 | Rate Control versus Rhythm Control for Atrial Fibrillation after Cardiac Surgery | National Heart, Lung, and Blood Institute and the National Institute of Neurological Disorders and Stroke of the National Institutes of Health, Bethesda, MD, and the Canadian Institutes of Health Research | 2 |
| NEJM | 5/19/2016 | Two-Year Outcomes of Surgical Treatment of Moderate Ischemic Mitral Regurgitation | National Heart, Lung, and Blood Institute and the National Institute of Neurological Disorders and Stroke of the National Institutes of Health and the Canadian Institutes of Health Research | 2 |
| NEJM | 5/19/2016 | Randomized Trial of a Lifestyle Program in Obese Infertile Women | Netherlands Organization for Health Research and Development | 2 |
| NEJM | 7/14/2016 | Initiation Strategies for Renal-Replacement Therapy in the Intensive Care Unit | French Ministry of Health. | 2 |
| NEJM | 8/11/2016 | Fresh versus Frozen Embryos for Infertility in the Polycystic Ovary Syndrome | National Basic Research Program of China | 2 |
| NEJM | 9/8/2016 | CPAP for Prevention of Cardiovascular Events in Obstructive Sleep Apnea | National Health and Medical Research Council (NHMRC) | 2 |
| NEJM | 10/27/2016 | A Randomized Trial of Long-Term Oxygen for COPD with Moderate Desaturation | National Heart, Lung, and Blood Institute, National Institutes of Health and Department of Health and Human Services | 2 |
| NEJM | 12/15/2016 | Five-Year Outcomes after Off-Pump or On-Pump Coronary-Artery Bypass Grafting | Canadian Institutes of Health Research | 2 |
| NEJM | 12/29/2016 | Randomized Trial of Bilateral versus Single Internal-Thoracic-Artery Grafts | British Heart Foundation, the U.K. Medical Research Council , and the National Institute of Health Research Efficacy and Mechanism Evaluation Programme | 2 |
| NEJM | 2/26/2015 | Randomized Trial of Peanut Consumption in Infants at Risk for Peanut Allergy | National Institute of Allergy and Infectious Diseases | 2 |
| NEJM | 4/9/2015 | Randomized Trial of Primary PCI with or without Routine Manual Thrombectomy | Medtronic and the Canadian Institutes of Health Research | 3 |
| NEJM | 4/2/2015 | Trial of Early, Goal-Directed Resuscitation for Septic Shock | United Kingdom National Institute for Health Research Health Technology Assessment Programme | 2 |
| NEJM | 4/23/2015 | Prednisolone or Pentoxifylline for Alcoholic Hepatitis | National Institute for Health Research (NIHR) Health Technology Assessment program. | 2 |
| NEJM | 5/7/2015 | Approaches to Catheter Ablation for Persistent Atrial Fibrillation | St. Jude Medical | 1 |
| NEJM | 5/14/2015 | Therapeutic Hypothermia after Out-of-Hospital Cardiac Arrest in Children | National Heart, Lung, and Blood Institute or the National Institutes of Health | 2 |
| NEJM | 8/13/2015 | A Randomized Trial of Intrapartum Fetal ECG ST-Segment Analysis | NICHD and by funding from Neoventa Medical | 3 |
| NEJM | 8/20/2015 | Screening for Occult Cancer in Unprovoked Venous Thromboembolism | Heart and Stroke Foundation of Canada | 2 |
| NEJM | 9/24/2015 | Letrozole, Gonadotropin, or Clomiphene for Unexplained Infertility | National Institutes of Health, the Eunice Kennedy Shriver National Institute of Child Health and Human Development | 2 |
| NEJM | 10/1/2015 | Randomized Trial of Benznidazole for Chronic Chagas’ Cardiomyopathy | Canadian Institute of Health Research | 2 |
| NEJM | 11/12/2015 | Effect of PCI on Long-Term Survival in Patients with Stable Ischemic Heart Disease | Department of Veterans Affairs Cooperative Studies Program, Canadian Institute of Halth Research, Merck, Pfizer, Bristol-Myers Squibb, Fujisawa, Kos Pharmaceuticals, Datascope, AstraZeneca, Key Pharmaceutical, Sanofi-Aventis, First Horizon, and GE Healthcare | 3 |
| NEJM | 12/3/2015 | Acetaminophen for Fever in Critically Ill Patients with Suspected Infection | Health Research Council of New Zealand, the Australian and New Zealand Intensive Care Foundation, and the Waikato Medical Research Foundation | 2 |
| NEJM | 12/10/2015 | Isosorbide Mononitrate in Heart Failure with Preserved Ejection Fraction | National Heart, Lung, and Blood Institute (NHLBI) | 2 |
| NEJM | 12/17/2015 | Hypothermia for Intracranial Hypertension after Traumatic Brain Injury | National Institute for Health Research Health Technology Assessment program | 2 |
| NEJM | 12/31/2015 | A Trial of Wound Irrigation in the Initial Management of Open Fracture Wounds | Canadian Institutes of Health Research | 2 |
| NEJM | 01/02/2014 | Stenting and Medical Therapy for Atherosclerotic Renal-Artery Stenosis | National Heart, Lung, and Blood Institute of the National Institutes of Health | 2 |
| NEJM | 1/23/2014 | A Trial of Mass Isoniazid Preventive Therapy for Tuberculosis Control | Bill and Melinda Gates Foundation; the South African Mine Health and Safety Council; the Foundation for Innovative New Diagnostics, Switzerland; the National Institutes of Health, National Institute of Allergy and Infectious Diseases; U.K. Department of Health, Sanofi-Aventis | 3 |
| NEJM | 2/20/2014 | A Randomized Trial of Bevacizumab for Newly Diagnosed Glioblastoma | National Cancer Institute and by an unrestricted educational grant from Genentech. | 3 |
| NEJM | 2/20/2014 | Bevacizumab plus Radiotherapy–Temozolomide for Newly Diagnosed Glioblastoma | F. Hoffmann–La Roche | 1 |
| NEJM | 04/10/2014 | Albumin Replacement in Patients with Severe Sepsis or Septic Shock | Italian Medicines Agency | 2 |
| NEJM | 04/17/2014 | Aspirin in Patients Undergoing Noncardiac Surgery | Canadian Institutes of Health Research, the National Health and Medical Research Council of Australia, and the Spanish Ministry of Health and Social Policy, Bayer Pharma | 3 |
| NEJM | 05/01/2014 | A Randomized Trial of Protocol-Based Care for Early Septic Shock | National Institute of General Medical Sciences | 2 |
| NEJM | 05/29/2014 | Randomized Trial of Acetylcysteine in Idiopathic Pulmonary Fibrosis | NHLBI | 2 |
| NEJM | 07/03/2014 | A Randomized Trial of Epidural Glucocorticoid Injections for Spinal Stenosis | Agency for Healthcare Research and Quality | 2 |
| NEJM | 07/17/2014 | Effects of Extended-Release Niacin with Laropiprant in High-Risk Patients | Merck, the U.K. Medical Research Council, the British Heart Foundation, and Cancer Research U.K. | 3 |
| NEJM | 09/18/2014 | Ultrasonography versus Computed Tomography for Suspected Nephrolithiasis | Agency of Healthcare Research and Quality through its Clinical and Health Outcomes Initiative in Comparative Effectiveness | 2 |
| NEJM | 09/18/2014 | Outcomes 1 Year after Thrombus Aspiration for Myocardial Infarction | Swedish Research Council | 2 |
| NEJM | 09/18/2014 | Prednisolone and Mycobacterium indicus pranii in Tuberculous Pericarditis | Canadian Institutes of Health Research, the Canadian Network and Centre for Trials Internationally, the Population Health Research Institute, the South African Medical Research Council, the Lily and Ernst Hausmann Research Trust, and Cadila Pharma, India | 3 |
| NEJM | 10/02/2014 | Introduction of Gluten, HLA Status, and the Risk of Celiac Disease in Children | Celiac Foundation (Fondazione Celiachia) of the Italian Celiac Society | 2 |
| NEJM | 10/02/2014 | Randomized Feeding Intervention in Infants at High Risk for Celiac Disease | European Commission, the Azrieli Foundation, Deutsche Zöliakie Gesellschaft, Eurospital, Fondazione Celiachia, Fria Bröd, Instituto de Salud Carlos III, Spanish Society for Pediatric Gastroenterology, Hepatology, and Nutrition, Komitet Badań Naukowych, Fundacja Nutricia, Hungarian Scientific Research Funds , Stichting Coeliakie Onderzoek Nederland, Thermo Fisher Scientific, and the European Society for Pediatric Gastroenterology, Hepatology, and Nutrition. | 3 |
| NEJM | 10/09/2014 | Lower versus Higher Hemoglobin Threshold for Transfusion in Septic Shock | Danish Strategic Research Council and by Copenhagen University Hospital, Rigshospitalet, the Scandinavian Society of Anaesthesiology and Intensive Care Medicine (ACTA Foundation), and Ehrenreich's Foundation | 2 |
| NEJM | 10/09/2014 | Follow-up of Blood-Pressure Lowering and Glucose Control in Type 2 Diabetes | National Health and Medical Research Council of Australia | 2 |
| NEJM | 10/16/2014 | Goal-Directed Resuscitation for Patients with Early Septic Shock | National Health and Medical Research Council of Australia | 2 |
| NEJM | 11/20/2014 | Early versus On-Demand Nasoenteric Tube Feeding in Acute Pancreatitis | Netherlands Organization for Health Research and Development, The ZonMw Health Care Efficiency Research Program, and Nutricia | 3 |
| NEJM | 12/04/2014 | Surgical Treatment of Moderate Ischemic Mitral Regurgitation | National Heart, Lung, and Blood Institute, including funding by the National Institute of Neurological Disorders and Stroke and the Canadian Institutes of Health Research | 2 |
| NEJM | 02/28/2013 | High-Frequency Oscillation in Early Acute Respiratory Distress Syndrome | Canadian Institutes of Health Reseach | 2 |
| NEJM | 02/28/2013 | High-Frequency Oscillation for Acute Respiratory Distress Syndrome | National Institute for Health Research Health Technology Assessment Programme | 2 |
| NEJM | 03/07/2013 | A Trial of Imaging Selection and Endovascular Treatment for Ischemic Stroke | NINDS, Concentric Medical | 3 |
| NEJM | 03/07/2013 | Endovascular Therapy after Intravenous t-PA versus t-PA Alone for Stroke | National Institues of Health and the NAtional Institue of Neurological Disorders and Stroke, Genentech, EKOS, Concentric Medial, Cordis Neurovascular, and Boehringer Ingelheim | 3 |
| NEJM | 03/07/2013 | Endovascular Treatment for Acute Ischemic Stroke | Italian Medicines Agency (AIFA), Boehringer Ingelheim Italia | 3 |
| NEJM | 03/21/2013 | Percutaneous Closure of Patent Foramen Ovale in Cryptogenic Embolism | St. Jude Medical | 1 |
| NEJM | 03/21/2013 | Closure of Patent Foramen Ovale versus Medical Therapy after Cryptogenic Stroke | St. Jude Medical | 1 |
| NEJM | 03/28/2013 | Effects of Off-Pump and On-Pump Coronary-Artery Bypass Grafting at 1 Year | Canadian Institutes of Health Research | 2 |
| NEJM | 03/28/2013 | Off-Pump versus On-Pump Coronary-Artery Bypass Grafting in Elderly Patients | Maquet | 1 |
| NEJM | 04/18/2013 | A Randomized Trial of Glutamine and Antioxidants in Critically Ill Patients | Canadian Institutes of Health Research | 2 |
| NEJM | 5/2/2013 | Surgery versus Physical Therapy for a Meniscal Tear and Osteoarthritis | National Institutes of Health | 2 |
| NEJM | 5/9/2013 | n–3 Fatty Acids in Patients with Multiple Cardiovascular Risk Factors | Società Prodotti Antibiotici, Pfizer, and Sigma-Tau | 1 |
| NEJM | 06/06/2013 | A Randomized Trial of Nighttime Physician Staffing in an Intensive Care Unit | National Institute on Aging, National Institues of Health | 2 |
| NEJM | 6/13/2013 | Racemic Adrenaline and Inhalation Strategies in Acute Bronchiolitis | Haukeland University Hospital | 2 |
| NEJM | 6/20/2013 | Rapid Blood-Pressure Lowering in Patients with Acute Intracerebral Hemorrhage | National Health and Medical Research Council (NHMRC) of Australia | 2 |
| NEJM | 6/20/2013 | Clopidogrel in Infants with Systemic-to-Pulmonary-Artery Shunts | Sanofi-Aventis and Bristol-Myers Squibb | 1 |
| NEJM | 7/11/2013 | Cardiovascular Effects of Intensive Lifestyle Intervention in Type 2 Diabetes | NIH, Johnson & Johnson, Nestle HealthCare Nutrition, Hoffmann-La Roche, Abbott Nutrition and Unilever North America | 3 |
| NEJM | 7/25/2013 | Therapies for Active Rheumatoid Arthritis after Methotrexate Failure | Canadian Institutes for Health Research | 2 |
| NEJM | 8/15/2013 | A Trial Comparing Noninvasive Ventilation Strategies in Preterm Infants | Canadian Institutes of Health Research | 2 |
| NEJM | 9/12/2013 | Pretreatment with Prasugrel in Non–ST-Segment Elevation Acute Coronary Syndromes | Daiichi Sankyo and Eli Lilly | 1 |
| NEJM | 10/03/2013 | A Randomized Trial of Planned Cesarean or Vaginal Delivery for Twin Pregnancy | Canadian Institutes of Health Research | 2 |
| NEJM | 10/03/2013 | Saxagliptin and Cardiovascular Outcomes in Patients with Type 2 Diabetes Mellitus | AstraZeneca and Bristol-Myers Squibb | 1 |
| NEJM | 10/24/2013 | Thrombus Aspiration during ST-Segment Elevation Myocardial Infarction | Swedish Research Council | 2 |
| NEJM | 12/5/2013 | Targeted Temperature Management at 33°C versus 36°C after Cardiac Arrest | Swedish Heart-Lung Foundation | 2 |
| NEJM | 12/12/2013 | A Pharmacogenetic versus a Clinical Algorithm for Warfarin Dosing | National Institutes of Health, Bristol-Myers Squibb, GenMark Diagnostics and AutoGenomics | 3 |
| NEJM | 12/12/2013 | A Randomized Trial of Genotype-Guided Dosing of Acenocoumarol and Phenprocoumon | European Commission Seventh Framework Programme, GlaxoSmithKline | 3 |
| NEJM | 12/26/2013 | Arthroscopic Partial Meniscectomy versus Sham Surgery for a Degenerative Meniscal Tear | Sigrid Juselius Foundation, the Competitive Research Fund of Pirkanmaa Hospital District, and the Academy of Finland. | 2 |
| NEJM | 01/12/2012 | Subclinical Atrial Fibrillation and the Risk of Stroke | St. Jude Medical | 1 |
| NEJM | 02/02/2012 | ABVD Alone versus Radiation-Based Therapy in Limited-Stage Hodgkin's Lymphoma | National Cancer Institute of Canada | 2 |
| NEJM | 02/09/2012 | Antenatal Thyroid Screening and Childhood Cognitive Function | Wellcome Trust and Compagnia di San Paulo | 2 |
| NEJM | 03/01/2012 | A Randomized Trial of Nicotine-Replacement Therapy Patches in Pregnancy | National Institute for Health Research (NIHR) | 2 |
| NEJM | 03/15/2012 | Closure of Patent Foramen Ovale versus Medical Therapy after Cryptogenic Stroke | St. Jude Medical | 1 |
| NEJM | 04/19/2012 | Off-Pump or On-Pump Coronary-Artery Bypass Grafting at 30 Days | Canadian Institutes of Health Research | 2 |
| NEJM | 05/17/2012 | Warfarin and Aspirin in Patients with Heart Failure and Sinus Rhythm | National Institute of Neurological Disorders and Stroke | 2 |
| NEJM | 05/24/2012 | Prednisone, Azathioprine, and N-Acetylcysteine for Pulmonary Fibrosis | NHLBI, Cowlin Family Fund, Zambon | 3 |
| NEJM | 05/31/2012 | Drotrecogin Alfa (Activated) in Adults with Septic Shock | Eli Lilly | 1 |
| NEJM | 07/12/2012 | Hydroxyethyl Starch 130/0.42 versus Ringer's Acetate in Severe Sepsis | Danish Rearch Couoncil | 2 |
| NEJM | 07/26/2012 | n–3 Fatty Acids and Cardiovascular Outcomes in Patients with Dysglycemia | Sanofi | 1 |
| NEJM | 9/27/2012 | Tight Glycemic Control versus Standard Care after Pediatric Cardiac Surgery | National Heart, Lung, and Blood Institute, National Institutes of Health | 1 |
| NEJM | 10/04/2012 | Intraaortic Balloon Support for Myocardial Infarction with Cardiogenic Shock | German Research Foundation, the German Heart Reseach Foundation, the German Cardia Society, Arbeitsgemainschaft Leitende Kardiologische Krankenhausarzte, and the University of Leipzig-Heart Center and Marqet Cardiopulmonary and Telefelx Medical | 2 |
| NEJM | 10/25/2012 | Radiofrequency Ablation as Initial Therapy in Paroxysmal Atrial Fibrillation | Danish Heart Foundation and Biosense Webster | 3 |
| NEJM | 11/15/2012 | Hydroxyethyl Starch or Saline for Fluid Resuscitation in Intensive Care | National Health and Medical Research Council of Australia | 2 |
| NEJM | 11/29/2012 | Bedside Monitoring to Adjust Antiplatelet Therapy for Coronary Stenting | Allies in Cardiovascular Trials Initiatives and Organized Networks, Assistance Publique Hopitaux de Paris, Fondation de France, Sanofi-Aventis, Cordis, Medtronic, Boston Scientific, and Fondation Scociete Generale Asset Management | 3 |
| NEJM | 12/13/2012 | Ultrafiltration in Decompensated Heart Failure with Cardiorenal Syndrome | National Heart, Lung, and Blood Institute | 2 |
| NEJM | 12/27/2012 | A Trial of Intracranial-Pressure Monitoring in Traumatic Brain Injury | National Institutes and Health and the Fogarty International Center, National Institute of Neurological Disorders and Stroke, Integra Life Sciences | 3 |
| NEJM | 12/27/2012 | Effect of Cinacalcet on Cardiovascular Disease in Patients Undergoing Dialysis | Amgen | 1 |
| NEJM | 3/3/2011 | Diuretic Strategies in Patients with Acute Decompensated Heart Failure | National Heart, Lung, and Blood Institute | 2 |
| NEJM | 3/3/2011 | Long-Acting Risperidone and Oral Antipsychotics in Unstable Schizophrenia | Veterans Affairs Cooperative Studies Program and an unrestricted grant from Ortho-McNeil Janssen Scientific Affairs | 3 |
| NEJM | 4/14/2011 | Intervention to Reduce Transmission of Resistant Bacteria in Intensive Care | National Institute of Allergy and Infectious Diseases to the Bacteriology and Mycology Study Group clinical research network and the Bacteriology and Mycology Statistical and Operations Unit data coordinating center; the National Center for Research Resources to the Mayo Clinic Center for Translational Science Activities and the General Clinical Research Center at Emory University; and by Merck, Elan Pharmaceuticals, Roche Diagnostics, and Kimberly Clark | 3 |
| NEJM | 4/21/2011 | Decompressive Craniectomy in Diffuse Traumatic Brain Injury | National Health and Medical Research Council of Australia | 2 |
| NEJM | 5/26/2011 | Body-Weight–Supported Treadmill Rehabilitation after Stroke | National Institute of Neurological Disorders and Stroke, National Center for Medical Rehabilitation Research, National Institutes of Health | 2 |
| NEJM | 6/9/2011 | Immediate versus Delayed IUD Insertion after Uterine Aspiration | Susan Thompson Buffett Foundation | 2 |
| NEJM | 6/30/2011 | Mortality after Fluid Bolus in African Children with Severe Infection | Medical Research Council | 2 |
| NEJM | 7/7/2011 | Primary Isoniazid Prophylaxis against Tuberculosis in HIV-Exposed Children | National Institute of Health, Bristol-Myers Squibb | 3 |
| NEJM | 7/7/2011 | Effect of Nesiritide in Patients with Acute Decompensated Heart Failure | Scios | 1 |
| NEJM | 8/11/2011 | Early versus Late Parenteral Nutrition in Critically Ill Adults | Methusalem program of the Flemish government, the Research Fund of the Catholic University of Leuven, the Research Foundation Flanders, Belgium, and the Clinical Research Fund of the University Hospitals Leuven, Belgium. In addition, the Catholic University of Leuven received an unconditional and nonrestrictive research grant for this trial from Baxter Healthcare | 3 |
| NEJM | 8/18/2011 | Prevention of Intraoperative Awareness in a High-Risk Surgical Population | Foundation for Anesthesia Education and Research and the American Society of Anesthesiologists and Winnipeg Regional Health Authority and University of Manitoba Department of Anesthesia | 2 |
| NEJM | 12/15/2011 | Niacin in Patients with Low HDL Cholesterol Levels Receiving Intensive Statin Therapy | National Heart, Lung, and Blood Institute, Abbott Laboratories | 3 |
| NEJM | 12/29/2011 | Liberal or Restrictive Transfusion in High-Risk Patients after Hip Surgery | National Heart, Lung, and Blood Institute | 2 |
| NEJM | 12/29/2011 | Low-Molecular-Weight Heparin and Mortality in Acutely Ill Medical Patients | Sanofi | 1 |

NI=not incicated
