# Supplementary material for: A comprehensive review of randomized clinical trials in three medical journals reveals 396 medical reversals
Source: eLife. 2019 Jun 11;8:e45183. doi: 10.7554/eLife.45183 (PMC6559784; doi:10.7554/eLife.45183)
Supplement: Supplementary file 2. [file elife-45183-supp2.docx]

Supplementary File 2: Reversal summaries identified in the top medical journals, by journal

|  | **Article and Author** | **Primary Medical Discipline** | **Date and Journal** | **Summary** | **Systematic Review** | **Search terms** |
| --- | --- | --- | --- | --- | --- | --- |
| 1 | Effectiveness of household lockable pesticide storage to reduce pesticide self-poisoning in rural Asia: a community-based, cluster-randomised controlled trial Pearson et al. | Public Health and General Preventive Medicine | 10/21/2017  Lancet | In rural Asia, pesticide self-poisoning is a common health issue. Restricting access to pesticides has been previously shown to reduce both method-specific and all-cause suicide rates in certain Asian countries. ^1 2^ The WHO advocates the use of locked boxes for storing pesticides in farming areas.^3^ In this study, lockable storage containers for pesticides were compared to usual practice in households (27,091 households to lockable storage containers and 26,291 households without) in rural areas of Sri Lanka. Lockable storage containers did not reduce the risk of pesticide self-poisoning (293/100,000 vs. 318/100,000 for intervention and control groups, respectively; p=0.33). This is a reversal of lockable storage containers to reduce self-poisoning. | None found | lock box self-poisoning (with and without "preventing") |
| 2 | Family-led rehabilitation after stroke in India (ATTEND): a randomised controlled trial ATTEND Collaborative Group; Lindley et al. | Neurology/Neurosurgery | 8/5/2017  Lancet | In low- to middle-income countries, community rehabilitation is thought of as a viable health-care delivery method to reduce disability. Results from multiple studies, included systematic reviews of early supported discharge (ESD) stroke services, have concluded that this type of rehabilitation can reduce death or dependency without placing adverse burden on family caregivers.^4^ The ATTEND trial found that family-led stroke rehabilitation in addition to routine rehabilitation did not reduce rates of death or dependency at six months, compared to routine rehabilitation, among patients (n=623 for the intervention group and 627 for the control group) who had a stroke within the past month and residual disability. The proportion of death or dependence was 47% (p=0.87) in both groups, suggesting that extra time and resources to implement a family-led rehabilitation program after stroke is not beneficial, and thus this practice is a reversal. | 2014. Cochrane review. “There is very low- to moderate-quality evidence that CME [caregiver-mediated exercises] may be a valuable intervention to augment the pallet of therapeutic options for stroke rehabilitation.”^4^ This review did not include this RCT, and “included studies were small, heterogeneous, and some trials had an unclear or high risk of bias.” | family led stroke rehabilitation |
| 3 | Intraoperative ketamine for prevention of postoperative delirium or pain after major surgery in older adults: an international, multicentre, double-blind, randomised clinical trial Avidan et al. | Anesthesiology | 7/15/2017  Lancet | Delirium and pain can often be co-occurring conditions in elderly people who have recently undergone surgery. Low-dose intraoperative ketamine has been associated with improved cognition after cardiac surgery and reduced pain, and it has been used by anesthesiologists around the world for over 50 years.^5^ In this study, ketamine did not decrease delirium in older adults after major surgery (19.4%; n=223 high-dose; n=227 in low-dose group), compared to placebo (19.8%; p=0.92; n=222), and even increased negative experiences such as hallucinations (p=0.01) and nightmares (p=0.03). This is a reversal of the practice of intraoperative ketamine for postoperative delirium or pain. | 2015. “Moderate to high-quality evidence supports the use of pharmacologic agents for the prevention of delirium, but results are based largely on one randomized controlled trial. The evidence for treating postcardiac surgery delirium with pharmacologic agents is inconclusive.”^6^ The study that carried most of the weight used dexamethasone in the intervention group. Only one included study in the meta-analysis had a ketamine intervention, and this study only included 58 men, which is far less than the 672 patients included in the Lancet study. | ketamine post-operative delirium |
| 4 | Prophylactic platelet transfusion plus supportive care versus supportive care alone in adults with dengue and thrombocytopenia: a multicentre, open-label, randomised, superiority trial Lye et al. | Infectious Disease | 4/22/2017  Lancet | Dengue is the most common vector-borne infection worldwide and is often associated with thrombocytopenia.^7^ Because of this, prophylactic platelet transfusion is often used, even though there has been no research investigating its efficacy and is sometimes not recommended.^8 9^ Results from this trial found that prophylactic platelet transfusion was no better than supportive care in preventing bleeding in those with dengue and thrombocytopenia (21% in platelet group vs. 26% in control group; RR=0.82; 95% CI=0.56-1.17; p=0.16; n=188 for transfusion and n=184 for the control group) and may even lead to adverse events, such as urticaria, other types of rashes, pruritus, chest pain, and anaphylaxis. This is a reversal of the practice of prophylactic platelet transfusion in patients with dengue and thrombocytopenia. | None found | Prophylactic platelet transfusion dengue |
| 5 | Post-deployment screening for mental disorders and tailored advice about help-seeking in the UK military: a cluster randomised controlled trial Rona et al. | Psychiatry | 4/8/2017  Lancet | A higher prevalence of mental health problems, including psychological distress and alcohol misuse, has been observed in personnel in direct combat roles deployed to Iraq and Afghanistan. To help in monitoring personnel, the US Armed Forces has implemented a post-deployment screening program for mental disorders.^10^ Canada, Australia, and the Netherlands also have post-deployment screening procedures for mental disorders.^11 12^ However, in this study, post-deployment screening with tailored help-seeking advice for mental disorders was no more effective at reducing prevalence of mental health disorders than receiving screening with general mental health advice (post-traumatic stress disorder OR=0.92; 95% CI=0.75-1.14; depression or anxiety OR=0.91; 95% CI=0.71-1.16; n=6350 in the screening group and n=3840 in the control group), nor did it increase help seeking for mental disorders (OR=0.92; 95% CI=0.78-1.08). This is a reversal of the practice of post-deployment screening for mental disorders. | None found | Post-deployment screening for mental disorders |
| 6 | Prophylactic hydration to protect renal function from intravascular iodinated contrast material in patients at high risk of contrast-induced nephropathy (AMACING): a prospective, randomised, phase 3, controlled, open-label, non-inferiority trial Nijssen et al. | Nephrology |  | Intravenous isotonic saline is recommended as prophylaxis for patients undergoing iodinated contrast procedures because contrast-induced nephropathy can often occur with this procedure and the infusion of isotonic saline is thought to prevent some of complications of nephropathy.^13 14^ In the present study of high-risk patients, it was found that contrast-induced nephropathy was no more common in those with no prophylaxis (2.6%; n=332) as compared to those with intravenous hydration (2.7%; p=0.47; n=328), and the cost savings was greater with no prophylaxis. This is a reversal of the practice of prophylactic hydration to protect renal function in high-risk patients undergoing iodinated contrast. | None found | Prophylactic saline hydration iodinated contrast material, no age restriction |
| 7 | High-flow warm humidified oxygen versus standard low-flow nasal cannula oxygen for moderate bronchiolitis (HFWHO RCT): an open, phase 4, randomised controlled trial Kepreotes et al. | Pulmonary disease | 3/4/2017  Lancet | High-flow warm humidified oxygen (HFWHO) is increasingly used for the treatment of respiratory infections in the pediatric population,^15-17^ but its efficacy has not been established in clinical trials. In this large randomized controlled trial, HFWHO did not reduce time on oxygen compared with cold wall oxygen (100%) therapy in children with moderate bronchiolitis. Median time to weaning was 24 hours for standard therapy (n=101) and 20 hours for HFWHO (HR=0.9; 95% CI=0.7-1.2; p=0.61; n=101). This is a reversal of the practice of HFWHO in children with moderate bronchiolitis. | 2017. “No difference in mortality or intubation was detected in patients with acute respiratory failure treated with high-flow nasal cannulae compared with usual care.” ^18^ This review did not include the RCT. | High-flow warm humidified oxygen and respiratory infection children |
| 8 | Comparison of an everolimus-eluting bioresorbable scaffold with an everolimus-eluting metallic stent for the treatment of coronary artery stenosis (ABSORB II): a 3 year, randomised, controlled, single-blind, multicentre clinical trial Serruys et al. | Cardiovascular Disease | 11/19/2016  Lancet | Bioresorbable scaffolds were developed as a way to promote revascularization to an area affected by obstructed coronary disease. These devices are thought to prevent restenosis after balloon angioplasty by delivering medications, but then will bioresorb after a time when the medications are no longer needed. These devices have received a CE mark and are widely used.^19 20^ In the ABSORB II Trial (335 patients in the scaffold group and 166 in the stent group), bioresorbable scaffolds were not superior to metallic stents, in regards to vasomotor reactivity (0.047 mm for scaffold vs. 0.056 mm for stent; p=0.49) and had larger late luminal loss (0.37 mm for scaffold vs. 0.25 mm for stent; p=0.78). This is a reversal of bioresorbable scaffolds for the treatment of coronary artery stenosis. | 2017. “Compared with everolimus-eluting stents, [bioresorbable vascular scaffolds] BVS is associated with increased risk of target lesion failure driven by the increased rates of target vessel myocardial infarction and ischemia-driven target lesion revascularization in these studies (mean follow-up, 25 months). The risk of definite or probable stent/scaffold thrombosis and very late stent/scaffold thrombosis seems to be higher with BVS. Further information from randomized trials is critical to evaluate clinical outcomes with BVS on complete resolution of the scaffold.”^21^ | GoogleScholar cited RCT |
| 9 | Comparison of stapled haemorrhoidopexy with traditional excisional surgery for haemorrhoidal disease (eTHoS): a pragmatic, multicentre, randomised controlled trial Watson et al. | Surgery | 11/12/2016  Lancet | Stapled hemorrhoidopexy and hemorrhoidal artery ligation are two newer, but commonly used, surgical interventions for severe hemorrhoids, although traditional excisional surgery is still used. A supposed advantage of the newer treatments is that they have less postoperative pain with similar symptom control.^22^ Numerous studies have been done, including 50 randomized controlled trials and several meta-analyses, comparing outcomes between stapled hemorrhoidopexy and traditional excisional surgery but these have had variable sample size and quality.^23-25^ To further examine and compare these outcomes, the eTHoS trial (389 received stapled haemorrhoidopexy and 388 received traditional excisional surgery) found that traditional excisional surgery led to more favorable pain scores, as measured by the EuroQol5 dimensions 3 level score, than stapled hemorrhoidopexy (1.62 vs. 1.56, respectively; p=0.03). This is a reversal of stapled hemorrhoidopexy for hemorrhoid disease. | None found | stapled hemorrhoidopexy with traditional excisional surgery |
| 10 | Efficacy of infant simulator programmes to prevent teenage pregnancy: a school-based cluster randomised controlled trial in Western Australia Brinkman et al. | Public Health and General Preventive Medicine | 11/5/2016  Lancet | The infant simulator is an example of persuasion technology or captology, where the use is intended to prevent teenage pregnancy.^26^ Their use is widespread in developed countries^27^ and is expanding into low-income and middle-income countries.^28^ However, in this study done in Australia, the infant simulator-based VIP program did not reduce teenage pregnancy. In fact, girls in the intervention group (n=1,267) were more likely to experience a birth (8% vs. 4%; HR=1.35; 95% CI=1.06-1.73; p=0.016) or an induced abortion (9% vs. 6%; HR=1.33 (1.00-1.78; p=0.049) than those in the control group (n=1,567) before they reached 20 years of age. This is a reversal of the practice of infant simulator programs to prevent teenage pregnancy. | None found | infant simulator program and teenage pregnancy |
| 11 | Platelet function monitoring to adjust antiplatelet therapy in elderly patients stented for an acute coronary syndrome (ANTARCTIC): an open-label, blinded-endpoint, randomised controlled superiority trial Cayla et al. | Cardiovascular Disease | 10/22/2016  Lancet | Platelet function monitoring may be one way to measure the platelet reactivity and adjust antiplatelet therapy to potentially improve clinical outcomes in patients with coronary artery disease, and was being used more frequently prior to this study.^29^ One example of this method of monitoring is the VerifyNow P2Y12 test, which measures aggregation with light transmittance to measure a patient’s response to antiplatelet therapy.^30^ Even though platelet function testing is still commonly used and international guidelines recommend platelet function testing in high-risk situations,^31 32^ this randomized controlled study (442 assigned to the monitoring group and 435 to the conventional group) does not support this practice in older adults with acute coronary syndrome. Rate of the composite outcome (cardiovascular death, myocardial infarction, stroke, stent thrombosis, urgent revascularization, and bleeding complications) was no better in the platelet testing group than in the control group ( HR=1.00; 95% CI=0.78-1.29). This is a reversal of the practice of platelet function monitoring to adjust antiplatelet therapy in elderly patients stented for acute coronary syndrome. | 2017. “Compared with traditional antiplatelet treatment, tailoring antiplatelet therapy according to  platelet reactivity testing failed to reduce all-cause mortality, MACE, and major bleeding events  in patients undergoing PCI..”^33^ | GoogleScholar cited RCT |
| 12 | Dexamethasone and supportive care with or without whole brain radiotherapy in treating patients with non-small cell lung cancer with brain metastases unsuitable for resection or stereotactic radiotherapy (QUARTZ): results from a phase 3, non-inferiority, randomised trial Mulvenna et al. | Oncology | 10/22/2016  Lancet | Whole brain radiotherapy (WBRT) in combination with steroids is a widely used approach in the management of patients with brain metastases, even though there is little evidence that it improves the quality of life or overall survival for the patient.^34 35^ With a sample size much bigger than other studies to date (N=538; 269 to each group), the QUARTZ Trial found that WBRT treatment did not result in better overall survival (HR=1.06; 95% CI=0.90-1.26) or quality of life (46.4 days for WBRT vs. 41.7 days for control) for patients with non-small cell lung cancer, and can be omitted from standard treatment. This is a reversal for WBRT in patients with NSCLC and brain metastases. | 2018. Cochrane review. There were only two studies, including the Mulvenna study, reporting on steroids alone versus steroids and WBRT. They were not able to pool the data because the other study did not include sufficient detail.^36^ | PubMed suggestion |
| 13 | Robot-assisted laparoscopic prostatectomy versus open radical retropubic prostatectomy: early outcomes from a randomised controlled phase 3 study Yaxley et al. | Oncology | 9/10/2016  Lancet | Surgery has traditionally been the main treatment of localized prostate cancer. However, complications with open radical retropubic prostatectomy has led to the search for less invasive treatments. Robot-assisted laparoscopic prostatectomy was introduced in 2001^37^ and has been rapidly adopted, increasing from 1.8% to 85% between 2003-2013. Robot-assisted laparoscopic prostatectomy is becoming the dominant surgical approach for prostatectomy in many countries.^38^ In this randomized trial, urinary and sexual function scores were no better in the robot-assisted laparoscopic prostatectomy group (83.8 and 35.0, respectively; n=163) than the radical retropubic prostatectomy group (82.5 and 38.9, respectively; n=163; p-values are 0.48 and 0.18). This is a reversal of the practice of robot-assisted laparoscopic prostatectomy for localized prostate cancer. | 2017. Cochrane review. “There is no high-quality evidence to inform the comparative effectiveness of LRP [laparoscopic prostatectomy] or RARP [radical retropubic prostatectomy] compared to ORP for oncological outcomes. Urinary and sexual quality of life-related outcomes appear similar.”^39^ | GoogleScholar cited RCT |
| 14 | Immediate total-body CT scanning versus conventional imaging and selective CT scanning in patients with severe trauma (REACT-2): a randomised controlled trial Sierink et al. | Critical Care Medicine | 8/13/2016  Lancet | Total-body CT scanning is increasingly used in the primary assessment of patients with trauma because it can provide a complete overview of life-threatening injuries faster than standard work-up and is sometimes advocated as the method of choice for injury screening.^40 41^ This study found that the use of an immediate total-body CT scan as part of trauma work-up did not reduce in-hospital mortality compared with the standard radiological work-up (total-body CT= 16% vs. standard work up 16%; p=0.92; 702 to immediate total-body CT and 701 to standard work-up). This is a reversal of the practice of total body scanning in patients with trauma, as standard radiological work-up was just as good. | 2017. A recent MA found that this was the only RCT on this topic, whereas other studies on this topic were observational.^42^ | total body ct scan vs. selective in trauma (selected the most recent |
| 15 | Platelet transfusion versus standard care after acute stroke due to spontaneous cerebral haemorrhage associated with antiplatelet therapy (PATCH): a randomised, open-label, phase 3 trial Baharoglu et al. | Neurology/ Neurosurgery | 6/25/2016  Lancet | Platelet transfusion is often given to patients in the emergency department, stroke units, and neurosurgical settings who have had a hemorrhagic stroke because, often, it is these patients who are also taking antiplatelet therapy.^43 44^ Additionally, multiple retrospective studies have been done in multiple locations, showing that this is a used practice^45^. In the PATCH Trial, the odds of death or dependence was actually higher, not lower, among patients receiving platelet transfusion treatment, compared to standard care. Survival was 68% in the platelet transfusion group (n=97) vs. 77% for the standard care group (n=93; OR=2.05; 95% CI=1.18-3.56; p=0.01). This is a reversal of the practice of platelet transfusion to patients with hemorrhagic stroke, especially since it leads to worse outcomes. | None found | platelet transfusion after stroke |
| 16 | Hysteroscopy in recurrent in-vitro fertilisation failure (TROPHY): a multicentre, randomised controlled trial El-Toukhy et al. | Obstetrics and Gynecology | 6/25/2016  Lancet | Up to 25% of women with infertility have abnormal intrauterine pathology.^46^ Outpatient hysteroscopy before starting in-vitro fertilization (IVF) may help to diagnose and treat abnormalities of the cervix and uterine cavity and possibly improve IVF outcomes, and is sometimes considered part of the initial evaluation for infertility. ^47 48^ However, in this randomized controlled trial of 350 women in the hysteroscopy group and 352 women in the control group, outpatient hysteroscopy before IVF did not improve the livebirth rate, compared to no hysteroscopy in women with a history of unsuccessful IVF cycles (29% in both groups; risk ratio 1.0; 95% CI=0.79-1.25; p=0.96). This is a reversal of the practice of hysteroscopy in women with in-vitro fertilization, as it does not lead to better patient outcomes. | 2015. Cochrane review. “More studies are needed before hysteroscopy can be proposed as a fertility-enhancing procedure in the general population of women having difficulty becoming pregnant.”^49^ The review did not include the RCT. | Hysteroscopy in "in-vitro fertilization" |
| 17 | Hysteroscopy before in-vitro fertilisation (inSIGHT): a multicentre, randomised controlled trial Smit et al. | Obstetrics and Gynecology | 6/25/2016  Lancet | Outpatient hysteroscopy before starting in-vitro fertilization (IVF) is a procedure commonly used, which may help to diagnose and treat abnormalities of the cervix and uterine cavity and possibly improve IVF outcomes.^47^ Similar to results of the TROPHY trial, routine hysteroscopy did not improve livebirth rates in infertile women with a normal transvaginal ultrasound of the uterine cavity, compared to immediately starting IVF treatment without hysteroscopy, in the inSIGHT trial (57% for hysteroscopy vs. 54% for immediate IVF; RR=1.06; 95% CI=0.93-1.20; p=0.41; n=373 in hysteroscopy group and n=377 in the immediate IVF group). This is a reversal of the practice of hysteroscopy in women with in-vitro fertilization, as it does not lead to better patient outcomes. | 2015. Cochrane review. “More studies are needed before hysteroscopy can be proposed as a fertility-enhancing procedure in the general population of women having difficulty becoming pregnant.”^49^ | Hysteroscopy in "in-vitro fertilization" |
| 18 | Immediate delivery compared with expectant management after preterm pre-labour rupture of the membranes close to term (PPROMT trial): a randomised controlled trial Morris et al. | Obstetrics and Gynecology | 1/30/2016  Lancet | Both the American College of Obstetricians and Gynecologists and Royal College of Obstetrics and Gynaecology support and/or recommend immediate delivery for women with ruptured membranes who are 34 weeks or greater.^50^ Neonatal infection is a major concern in when there has been a ruptured membrane, especially in premature infants.^51^ In this trial, participants assigned to the expectant management group did not have any worse outcomes regarding the primary outcomes of neonatal sepsis (2%; n=924 in the immediate birth arm vs. 3%; n=915 in the expectant management arm; RR=0.8; 95% CI=0.5-1.3; p=0.37) or neonatal morbidity and mortality (8% vs. 7%; p=0.32) than those assigned to immediate delivery, and had less respiratory distress (p=0.008) and need for mechanical ventilation (p=0.02). This is a reversal of the practice of immediate delivery in women with preterm, pre-labor rupture of the membranes, as it does not lead to less neonatal sepsis. | 2017. Cochrane review. “We found no clinically important difference in the incidence of neonatal sepsis between women who birth immediately and those managed expectantly in PPROM prior to 37 weeks' gestation. Early planned birth was associated with an increase in the incidence of neonatal RDS, need for ventilation, neonatal mortality, endometritis, admission to neonatal intensive care, and the likelihood of birth by caesarean section, but a decreased incidence of chorioamnionitis.”^52^ | GoogleScholar cited RCT |
| 19 | Effectiveness of a nurse-led intensive home-visitation programme for first-time teenage mothers (Building Blocks): a pragmatic randomised controlled trial Robling et al. | Public Health and General Preventive Medicine | 1/9/2016  Lancet | Children born to young mothers or into impoverished circumtances are at higher risk for numerous adverse health outcomes. Programs such as the Family Nurse Partnership (FNP) have been designed to address maternal and birth outcomes, and improve cognitive and socioeconomic development.^53^ FNP has been offered outside of research settings since 1996.^54^ After several randomized trials showed this program to be successful in US cities, the UK implemented this program into a few selected areas.^53^ After implementation to a selected number of partnerships, the number of partnerships in the UK was expanded and a randomized trial, in pregnant women less than 20 years of age, testing the effectiveness of the program began (823 women assigned to FNP plus usual care and 822 received usual care only). This trial reported no differences in number of mothers who smoked (56% vs 56%; p=0.51) or in mean birthweight (3217 grams vs. 3198 grams; p=0.50). The practice of FNP in addition to usual care provided no key benefits and should be reversed. | None found |  |
| 20 | Outcomes after thrombus aspiration for ST elevation myocardial infarction: 1-year follow-up of the prospective randomised TOTAL trial Jolly et al. | Cardiovascular Disease | 1/9/2016  Lancet | In treating patients with ST elevation myocardial infarction, thrombus aspiration during percutaneous coronary intervention (PCI) is thought to reduce distal embolization and improve microvascular perfusion. Multiple studies show that this is an established practice,^55-58^ and the 2009 American College of Cardiology Foundation/American Heart Association Task Force on Practice Guidelines had recently included this as part of their recommendations.^59^ A dozen devices have been developed for manual thrombectomy.^60^ However, in this study, routine thrombus aspiration did not reduce the composite of cardiovascular death, myocardial infarction, shock, or heart failure compared with PCI alone. The primary outcome (a composite of CV death, MI, cardiogenic shock, or heart failure) occurred 8% in both groups (HR=1.00; 95%CI=0.87-1.15; thrombectomy followed by PCI n=5,371 and PCI alone n=5,360). This is a reversal of the practice of thrombus aspiration for ST elevation myocardial infarction. | 2017. “Routine aspiration thrombectomy prior to primary PCI was not associated with a reduction in long-term mortality or clinical outcomes.”^61^ | GoogleScholar cited RCT |
| 21 | Prophylactic antibiotics after acute stroke for reducing pneumonia in patients with dysphagia (STROKE-INF): a prospective, cluster-randomised, open-label, masked endpoint, controlled clinical trial Kalra et al. | Neurology/Neurosurgery | 11/7/2015  Lancet | Post-stroke pneumonia can occur after an acute stroke. Prophylactic antibiotics might decrease the risk of post-stroke pneumonia, mortality, and disability in these patients. Prophylactic antibiotics was standard of care in the 1990s and is still commonly used, even though current guidelines recommend against using them.^62 63^  Antibiotic prophylaxis (n=615) did not lead to lower rates of pneumonia in stroke patients, compared to standard stroke unit care (n=602; 13% vs. 10%, respectively; OR=1.21; 95% CI=0.71-2.08; p=0.49). This is a reversal of prophylactic antibiotics for pneumonia after acute stroke. | 2018. Cochrane review. “Preventive antibiotics had no effect on functional outcome or mortality, but significantly reduced the risk of 'overall' infections. This reduction was driven mainly by prevention of urinary tract infection; no effect for pneumonia was found.”^64^ | PubMed suggestion |
| 22 | Early combined immunosuppression for the management of Crohn's disease (REACT): a cluster randomised controlled trial Khanna et al. | Gastroenterology/Hepatology | 11/7/2015  Lancet | Early combined immunosuppression (ECI) emerged as a treatment strategy for Crohn's disease in an attempt to overcome issues of traditional step-care approach, such as delaying in effective therapy for those who do not respond to therapy initially. Several studies have already reported on the benefits of this type of approach.^65 66^ The European Crohn's and Colitis Organization recommend treatment with azathioprine or methotrexate for any patient who relapses early (<3 months) and supports the use of early therapy for patients with moderately active disease^67 68^ However, in this study, ECI (n=1084) was not more effective than conventional management (n=806) for inducing corticosteroid-free remission in patients with Crohn’s disease (66% vs. 61.9%; p=0.52). This is a practice of ECI for the management of Crohn’s disease. | None found | early immunosuppression in crohn's disease |
| 23 | Percutaneous tibial nerve stimulation versus sham electrical stimulation for the treatment of faecal incontinence in adults (CONFIDeNT): a double-blind, multicentre, pragmatic, parallel-group, randomised controlled trial Knowles et al. | Gastroenterology/Hepatology | 10/24/2015  Lancet | Stimulation of the sacral nerve is the first-line surgical intervention for those who do not respond to non-surgical treatments for fecal incontinence. Stimulation of the tibial nerve is non-invasive and may produce similar results because of the shared sacral segmental innervation. Several studies, including observational studies, have been done to evaluate this intervention.^69 70^ Stimulation of the tibial nerve has been proposed as a second line treatment for this condition.^71^ In this study, percutaneous tibial nerve stimulation (PTNS) did not lead to fewer episodes of fecal incontinence, compared to sham electrical stimulation (38% vs. 31% with 50% or greater reduction in the number of episodes of fecal incontinence per week; aOR=1.28; 95% CI=0.72-2.28; p=0.40; 115 assigned to PTNS and 112 to sham stimulation). This is a reversal fo percutaneous tibial nerve stimulation for fecal incontinence in adults. | Only one SR/MA was found on this specific topic. The authors conclude that percutaneous tibial nerve stimulation are associated with improvement in fecal incontinence, however, all but one of the studies used in their review were prospective case series.^72^ | Percutaneous tibial nerve stimulation and fecal incontinence |
| 24 | Methylprednisolone in patients undergoing cardiopulmonary bypass (SIRS): a randomised, double-blind, placebo-controlled trial Whitlock et al. | Cardiovascular disease | 9/26/2015  Lancet | Steroids suppress inflammatory responses that often occur in patients with cardiopulmonary bypass, and, therefore, might improve outcomes in patients at high risk of morbidity and mortality.^73^  This practice has been used routinely since the 1970s/1980s.^74^ In the SIRS study that included 3,655 patients in the methylprednisolone group and 3,752 to the placebo group, methylprednisolone did not lead to reductions in mortality (4% vs. 5%; RR=0.87; 95% CI=0.70-1.07; p=0.19) or major morbidity (24% vs. 24%; RR=1.03; 95% CI=0.95-1.11; p=0.52) after cardiopulmonary bypass. This is a reversal of methylprednisolone in patients undergoing cardiopulmonary bypass surgery. | A 2015 SR/MA that did not include this study, concluded that while corticosteroids decreased atrial fibrillation and length of hospital stay, they did not reduce mortality, and increased length of time spent on ventillation.^75^ | steroids for cardiopulmonary bypass |
| 25 | Medical expulsive therapy in adults with ureteric colic: a multicentre, randomised, placebo-controlled trial Pickard et al. | Urology | 7/25/2015  Lancet | Smooth muscle relaxant drugs are used for the treatment of ureteric colic as a kidney stone passes down the ureter, because they are thought to relax the ureteric smooth muscle. Guidelines by European Association of Urology (EAU) and the American Urologic Association (AUA) joint Guideline for the Management of Ureteral Calculi suggest this as a treatment option.^76 77^ Despite recommendations supporting their use, this study found that neither tamsulosin (81%; p=0.73; n=378) or nifedipine (80%; p=0.88; n=379) were more effective than placebo (80%; n=379) at decreasing the need for further treatment to achieve stone clearance (size 10mm or less) for patients with expectantly managed ureteric colic. This is a reversal of the practice of using either tamsulosin or nifedipine for expulsive therapy in patients with ureteric colic (stone size 10mm or less). | One recent SR/MA comparing only tamsulosin and nifedipine (the two active interventions in the study) found that nifedipine was better than tamsulosin but this SR/MA did not compare these interventions to a control arm.^78^ In another SR/MA of alpha blockers (tamsulosin) compared with placebo or other control, alpha blockers were associated with a higher likelihood of stone passage than placebo or other type of control, but this association was mainly for larger stones.^79^ | GoogleScholar cited RCT |
| 26 | Efficacy and safety of very early mobilisation within 24 h of stroke onset (AVERT): a randomised controlled trial AVERT Trail Collaboration Group; Bernhardt et al. | Neurology/Neurosurgery | 7/4/2015  Lancet | Early mobilization after stroke is recommended in many guidelines because of potential benefits to the musculoskeletal, cardiovascular, and respiratory systems during a window of brain plasticity and repair following stroke, with most recommending rehabilitation beginning as soon as the diagnosis of stroke is established and life threatening issues are under control or within 24 hours.^80^ Previous studies have generally found positive findings with this type of intervention, but in this randomized controlled trial, very early mobilization protocol (within 24 hours) led to fewer people achieving a high score on the modified Rankin Scale (46% [n=480] vs. 50% [n=525]; aOR=0.73; 95%CI=0.59-0.90; p=0.004), which is used to measure disability after a stroke, compared to usual care. This is a reversal of the practice of very early mobilization after a stroke. | 2017. Pooled data from RCTs concluded that VEM [very early mobilization] is not associated with beneficial effects when carried out in patients 24 or 48 hours after the onset of a stroke.”^81^ | GoogleScholar cited RCT |
| 27 | Automated, electronic alerts for acute kidney injury: a single-blind, parallel-group, randomised controlled trial Wilson et al. | Nephrology | 5/16/2015  Lancet | Automated alerts are used for a wide range of clinical settings, and may be especially helpful for monitoring kidney injury, which can be complex and time sensitive to treat.^82-85^ Because of this, consensus statements have been made recommending the use of tailored early treatment, and clinician notification has been rapidly adopted as a way to tailor the patient’s treatment^86-88^  However, in this trial, the use of an electronic alert system did not improve clinical outcomes (creatinine, dialysis, and death) in patients with acute kidney injury (11.1% [n=1207] vs. 11.6% [n=1192]; p=0.88). This is a reversal of the practice of automated, electronic alerts for acute kidney injury in hospitalized patients. | 2017. “The benefit of electronic alerting systems for acute kidney injury has this far not been supported by randomized studies..”^89^ This review only found and included two randomized studies. | GoogleScholar cited RCT |
| 28 | Efficacy of indoor residual spraying with dichlorodiphenyltrichloroethane against malaria in Gambian communities with high usage of long-lasting insecticidal mosquito nets: a cluster-randomised controlled trial Pinder et al. | Public Health and General Preventive Medicine | 4/11/2015  Lancet | Insecticidal nets, indoor residual spraying, and artemisinin-based therapies have helped in reducing malaria in sub-Saharan Africa. Survey data from 17 African countries showed that the combination use of long-lasting insecticidal nets and indoor residual spraying has been used and could be an effective combination to prevent even more cases and deaths from malaria.^90-92^ In this study, the use of indoor residual spraying in addition to insecticidal nets did not result in any improvement in clinical malaria or vector density than insecticidal nets only in Gambian village homes. The incidence rate was 0.047 per child-month in the control arm (nets; n=3,622) and 0.044 per child-month in the group with residual spraying, in addition to nets (n=3,777; rate ratio=1.08; 95% CI=0.80-1.46). This is a reversal of the practice of indoor residual spraying to prevent malaria. | No MA were found since the publication of this study. There was only was SR in the gray literature (BA thesis) that found positive results for this intervention but most of the studies were observational and not randomized.^93^ | GoogleScholar cited RCT |
| 29 | Effect of early neonatal vitamin A supplementation on mortality during infancy in Ghana (Neovita): a randomised, double-blind, placebo-controlled trial Edmond et al. | Pediatrics | 4/4/2015  Lancet | Vitamin A deficiency is a public health issue in low-income countries. While multiple trials have been performed, in addition to a Cochrane review, on the effectiveness of vitamin A supplementation in infants in low-income countries, the WHO stated that there was insufficient evidence to make a recommendation on its usage.^94-96^ The International Vitamin A Consultative Group (IVACG) supports the use of 50,000 IUs for infants <6 months of age.^97^ In this trial based in Ghana, vitamin A supplementation did not lead to a lower mortality rate compared to placebo (24.5/1,000 [n=11,474] vs. 21.8/1,000 [n=11,481] supplemented infants; RR1.12; 95% CI=0.95-1.33; p=0.18), in newborn infants. This is a reversal of the practice of vitamin A supplementation during the early neonatal period in Africa, as it does not improve mortality. | 2017. Cochrane review. “Evidence provided in this review does not indicate a potential beneficial effect of vitamin A supplementation among neonates at birth in reducing mortality during the first six months or 12 months of life.”^98^ | "vitamin A" supplementation infant mortality (in Cochrane reviews)/ Pubmed suggestion |
| 30 | Effect of neonatal vitamin A supplementation on mortality in infants in Tanzania (Neovita): a randomised, double-blind, placebo-controlled trial Masanja et al. | Pediatrics | 4/4/2015  Lancet | Vitamin A deficiency is a public health issue in low-income countries. While multiple trials, including a Cochrane review, have been performed on the effectiveness of vitamin A supplementation in infants in low-income countries, the WHO stated that there was insufficient evidence to make a recommendation on its usage.^94-96^ The International Vitamin A Consultative Group (IVACG) supports the use of 50,000 IUs for infants <6 months of age.^97^ In this trial based in Tanzania, vitamin A supplementation did not lead to a lower mortality rate (26/1,000 [n=15,995] vs. 24/1,000 [n=16,004] livebirths; risk ratio=1.10; 95%CI=0.95-1.26) compared to placebo, in newborn infants. This is a reversal of the practice of neonatal vitamin A supplementation to reduce mortality in infants in Africa. | 2017. Cochrane review. “Evidence provided in this review does not indicate a potential beneficial effect of vitamin A supplementation among neonates at birth in reducing mortality during the first six months or 12 months of life.”^98^ | "vitamin A" supplementation infant mortality (in Cochrane reviews)/ Pubmed suggestion |
| 31 | A population-based, multifaceted strategy to implement antenatal corticosteroid treatment versus standard care for the reduction of neonatal mortality due to preterm birth in low-income and middle-income countries: the ACT cluster-randomised trial Althabe et al. | Obstetrics and Gynecology | 2/14/2015  Lancet | Antenatal corticosteroids are effective at reducing preterm birth among women at risk. Even though it is recommended for high-risk women by the WHO,^99^ many women in low-income countries may not receive this potentially beneficial intervention. Multiple groups have studied and promoted the use of antenatal corticosteroid scale-up programs.^100 101^ When an antenatal scale-up program was implemented in low-income countries (dexamethasone administered from 24-36 weeks gestation) 28-day neonatal mortality in preterm infants did not decrease. Conversely, a higher mortality rate was seen in babies born 37 weeks gestation, compared to standard care (27.4/1,000 [n=48,219] vs. 23.9/1,000 [51,523 livebirths]; RR=1.12; 95% CI=1.02-1.22; p=0.013). While corticosteroids are beneficial for preventing preterm births in women with access to adequate health care, in low to middle income countries, this practice did not reduce neonatal mortality. This is a reversal of the practice of using corticosteroids for the reduction of neonatal mortality due to preterm births in women residing in low-income and middle-income countries. | None found | "corticosteroid at risk of preterm birth low income"; "scale up programs for corticosteroid" |
| 32 | Antepartum dalteparin versus no antepartum dalteparin for the prevention of pregnancy complications in pregnant women with thrombophilia (TIPPS): a multinational open-label randomised trial Rodger et al. | Obstetrics and Gynecology | 11/28/2014  Lancet | Women with genetic thrombophilias are at higher risk of adverse pregnancy outcomes.^102^ This observation, combined with results from small trials showing a benefit of low-molecular-weight heparin on pregnancy outcomes in women with thrombophilias, led to the adoption of this therapy by clinicians and guideline committees.^103-105^ However, in this study, antepartum prophylactic heparin did not reduce the occurrence of thromboembolism, pregnancy loss, or placenta-mediated pregnancy complications in pregnant women with thrombophilia (17.1% [n=146] vs. 18.9% [n=143] for the composite outcome; p=0.70). This is a reversal of the practice of antepartum prophylactic heparin administration in pregnant women with thrombophilia. | 2016. “We found no difference in preventing future pregnancy loss with LMWH [low-molecular-weight heparin] when compared with no LMWH in women with inherited thrombophilia and prior late or recurrent early pregnancy loss.”^106^ The study was published only a few months after the SR/MA was conducted, and therefore not included in the SR/MA. | GoogleScholar-cited RCT |
| 33 | Efficacy of paracetamol for acute low-back pain: a double-blind, randomised controlled trial Williams et al. | Orthopedic | 11/1/2014  Lancet | Paracetamol is the recommended first-line analgesic for acute low-back pain, despite the lack of high-quality evidence to support this recommendation. ^107 108^ In this study, time to recovery for patients with acute low-back pain was no different between regular or as-needed paracetamol, compared to placebo, with 85% [n=550], 83% [n=549], and 84% [547] of participants in the regular, as-needed, and placebo (control arm), respectively, reaching sustained recovery (p=0.79). Paracetamol is ineffective for acute low-back pain. This is a reversal of using paracetamol for acute low-back pain. | 2015. “Paracetamol is ineffective in the treatment of low back pain ^109^ The conclusions were based mainly on the results of this one study. | GoogleScholar cited RCT |
| 34 | Efficacy and cost of video-assisted thoracoscopic partial pleurectomy versus talc pleurodesis in patients with malignant pleural mesothelioma (MesoVATS): an open-label, randomised, controlled trial Rintoul et al. | Oncology | 9/20/2014  Lancet | Standard treatment for pleural effusion in patients with mesothelioma was once talc pleurodesis. .^110-112^ Video-assisted thoracic surgery was “supported with fervor” in the early 1990’s by pulmonologists and oncologists.^113^ In this study, video-assisted thoracoscopic partial pleurectomy, which is a more invasive intervention, did not lead to better survival in patients with mesothelioma (52% [n=87] vs. 57% [n=88] in the VATS and talc pleurodesis groups, respectively), and surgical complications were more common, when compared to the standard talc pleurodesis. This is a reversal of the practice of VATS in patients with malignant pleural mesothelioma. | 2016. Cochrane review. Using network analysis, the authors concluded that talc was a better method of pleurodesis than other used methods. While this review did cite this study, it was the only one included in the review that compared talc with partial pleurectomy.^114^ | suggested SR by PubMed. |
| 35 | High versus low positive end-expiratory pressure during general anaesthesia for open abdominal surgery (PROVHILO trial): a multicentre randomised controlled trial PROVE Network Investigators, Hemmes et al. | Anesthesiology | 8/9/2014  Lancet | Mechanical ventilation can help to minimize post-operative pulmonary complications, but this procedure can lead to its own set of complications. Positive end-expiratory pressure (PEEP) is commonly used in mechanical ventilation, and studies have shown that high-levels of PEEP can be safely applied.^115-117^ High PEEP was used frequently in the 1970’s and is now sometimes part of open lung and protective lung ventilation strategies.^118^ Postoperative complications in this study were no different between those with high and lower PEEP (40% [n=447] vs. 39% [n=453]; RR=1.01; 95%*=*0.86-1.20; p=0.86), and high PEEP led to more intraoperative hypotension and needed more vasoactive drugs. This is a reversal of high PEEP for patients receiving open abdominal surgery. | 2014. Cochrane review. “Evidence is currently insufficient to permit conclusions about whether intraoperative PEEP alters risks of postoperative mortality and respiratory complications among undifferentiated surgical patients.”^119^ The Cochrane review did not include this RCT. | GoogleScholar cited RCT |
| 36 | Effect of gravity on volume of placental transfusion: a multicentre, randomised, non-inferiority trial Vain et al. | Obstetrics and Gynecology | 7/19/2014  Lancet | It is believed that delaying cord clamping after a baby is delivered and holding the baby at or below the level of the vagina increases the beneficial passage of blood from the placenta to the baby. Because of this belief, recommendations have been made supporting this practice, even though it can be cumbersome and interferes with the mother/baby bonding.^120-122^ The authors of a Cochrane review advocate that mothers should be supported in their decision of baby placement after birth.^123^ A survey of nurse-midwives reported that the majority of respondents placed the baby on the mother's abdomen and not at a lower level. ^124^ This study showed that keeping the baby below the level of the vagina, as compared to placing the baby on the abdomen, did not lead to better weight gain (as a proxy for placental transfusion volume; 56 g [n=274] vs. 53 g [272]; p=0.45), and babies can be safely placed in the arms of their mothers immediately after birth without affecting weight gain. This is a reversal of the practice of keeping the baby at or below the level of the mother’s vagina immediately after birth. | A Cochrane review from 2010 was not able to make a conclusion because of the lack of studies evaluating practice (no RCTs). ^123^ | gravity and cord clamping (Cochrane.org - no SR/MA were found using GoogleScholar) |
| 37 | Comprehensive physiotherapy exercise programme or advice for chronic whiplash (PROMISE): a pragmatic randomised controlled trial Michaleff et al. | Orthopedic | 7/12/2014  Lancet | Clinical practice guidelines support the use of conservative treatment approaches such as physiotherapy exercise programs for whiplash-associated disorders but acknowledge that there has been little research on the effectiveness of this type of intervention.^125-127^ This study shows that a comprehensive exercise program (n=86) was no better in reducing pain for patients with chronic whiplash-associated disorder than was a one-time 30-minute consultation with a physiotherapist (n=86). Differences in pain at 14 weeks, 6 months, and 12 months were 0.0, 0.2, and -0.1 on a scale, where 2 is a clinically worthwhile effect. This is a reversal of the practice of a comprehensive physiotherapy exercise program for patients who have experienced whiplash. | A SR/MA that was presented (conference brief only) a few months before this study, concluded that conservative treatment (physiotherapy, behavioral approaches) was effective at reducing pain in patients with whiplash associated disorder.^128^ This RCT was not included in the review. | GoogleScholar cited RCT |
| 38 | Compression stockings to prevent post-thrombotic syndrome: a randomised placebo-controlled trial Kahn et al. | Cardiovascular Disease | 3/8/2014  Lancet | Post-thrombotic syndrome can often develop after deep venous thrombosis. Elastic compression stockings are often used and recommended because of their potential to reduce venous hypertension and reflux.^129-131^ In one survey of university-based physicians in Canada, 68% reported prescribing compression stockings if the patients had venous symptoms.^132^ In the SOX trial, elastic compression stockings did not prevent post-thrombotic syndrome in patients with an initial deep vein thrombosis (14.2% in the compression stocking group [n=410] and 12.7% in the placebo group [n=396]; HR=1.13; 95% CI=0.73-1.76; p=0.58). This is a reversal of using compression stocking to prevent post-thrombotic syndrome in patients who experienced deep venous thrombosis. | 2017. Cochrane review. “Low-quality evidence suggests that elastic compression stockings may reduce the occurrence of PTS after DVT. We downgraded the quality of evidence owing to considerable heterogeneity between studies and lack of or unclear risk of blinding due to clinical assessment scores... Large randomised controlled trials are needed to confirm these findings because of current lack of high-quality evidence and considerable heterogeneity.” ^133^ | PubMed suggestion |
| 39 | Medical management with or without interventional therapy for unruptured brain arteriovenous malformations (ARUBA): a multicentre, non-blinded, randomised trial Mohr et al. | Neurology/Neurosurgery | 2/15/2014  Lancet | Brain arteriovenous malformations in patients confer a small but higher risk of brain hemorrhage. There is debate about whether to treat these malformations before they rupture. Currently, a conservative approach is recommended but microsurgical resection, stereotactic radiotherapy, and endovascular embolization are commonly used.^134-136^ The Stroke Council of the American Heart Association guidelines at the time of this study strongly recommended the consideration of interventional therapy for larger aneurysms (>9mm).^137^ In this study, medical management alone led to less death or stroke than did medical management with interventional therapy (10.1% vs. 30.7%, respectively) in patients with unruptured brain arteriovenous malformations of various sizes. This is a reversal of the practice of interventional therapy in patients with unruptured brain arteriovenous malformations and who receive medical management. | None found | "unruptured brain arteriovenous malformations medical management:, "unruptured brain arteriovenous malformations treatment" (no date restriction) |
| 40 | Liverpool Care Pathway for patients with cancer in hospital: a cluster randomised trial Constantini et al. | Oncology | 1/18/2014  Lancet | The Liverpool Care Pathway (LCP) program for dying patients was developed in the late 1990s in an attempt to improve end-of life care by transferring hospice practices of end-of-life care to hospitals. Broad uptake of this program occurred before its effectiveness could be thoroughly studied.^138-141^ Results from this study show that scores measuring the quality of end-of-life care were no different between hospitals who implemented LCP and those with standard health-care practice (score 70.5/100 [n=147] vs. 63.0/100 [161]; p=0.19). This is a reversal of using the LCP in hospitalized cancer patients. | 2017. Cochrane review. “There is limited available evidence concerning the clinical, physical, psychological or emotional effectiveness of end-of-life care pathways.”^142^ | PubMed suggestion |
| 41 | A structured training programme for caregivers of inpatients after stroke (TRACS): a cluster randomised controlled trial and cost-effectiveness analysis Forster et al. | Neurology/Neurosurgery | 12/21/2013  Lancet | Stroke can lead to disability, which can place added burden on family members who care for these patients on a daily basis. Interventions have been developed and implemented to reduce the burden on caregivers.^143 144^ In this study, caregivers who received training for how to care for stroke patients did not have better scores on the caregiver burden scale (45.5 [n=450] vs. 45.0 [n=478]; p=0.67), nor did patients have any better functional scores (27.4 vs. 27.6; p=0.87) than did caregivers or patients who received usual care. This is a reversal of training programs for caregivers immediately after stroke. | On recent SR/MA found that transitional care interventions were generally effective at reducing mortality, but when looking only at educational interventions, which this study was considered, there was no beneficial effect on mortality.^145^ This RCT was the only multi-center study with a primary education intervention and had the largest sample size. | GoogleScholar cited RCT |
| 42 | Intra-aortic balloon counterpulsation in acute myocardial infarction complicated by cardiogenic shock (IABP-SHOCK II): final 12 month results of a randomised, open-label trial Thiele et al. | Cardiovascular Disease | 11/16/2013  Lancet | Mortality in acute myocardial infarction is high even though beneficial treatment strategies have been developed. The Intra-aortic balloon pump (IABP) has been the most frequently used mechanical cardiac assist device for almost 50 years.^146^ However, IABP did not lead to lower mortality in this study of patients undergoing early revascularization for myocardial infarction (52% [n=301] vs. 51% [n=299]; RR=1.01; 95% CI=0.86-1.18; p=0.91). This is a reversal of IABP in acute myocardial infarction complicated by cardiogenic shock. | 2016. “In patients undergoing high-risk coronary revascularization, IABP did not significantly decrease mortality. But high-risk CABG patients may be benefit from IABP. Rigorous criteria should be applied to the use of IABPs.”^147^ | GoogleScholar cited RCT |
| 43 | Effect of household and community interventions on the burden of tuberculosis in southern Africa: the ZAMSTAR community-randomised trial Ayles et al. | Pulmonary Disease | 10/5/2013  Lancet | Enhanced case finding of tuberculosis has been done with various methods since the 1960s.^148^ Recommendations current to the time of this study recommended the use of media campaigns and outreach at schools and workplaces, in addition to active case finding.^148^ In the ZAMSTAR trial, neither enhanced case finding (adjusted prevalence ratio=1.04; 95% CI=0.72-1.51; p=0.81) nor household interventions (adjusted prevalence ratio=0.78; 95% CI=0.54-1.12; p=0.16) led to reductions in tuberculosis, compared to standard community clinic practices. This is a reversal of the practice of enhanced case findings and household interventions in Africa to reduce tuberculosis incidence. | 2017. Cochrane review. “The available evidence demonstrates that when used in appropriate settings, active case-finding approaches may result in increase in tuberculosis case detection in the short term. The effect of active case finding on treatment outcome needs to be further evaluated in sufficiently powered studies.”^149^ While the case detection was better in this review, there did not appear to be an improvement in treatment success, mortality, or TB prevalence in the community with these interventions. |  |
| 44 | Screening and counselling in the primary care setting for women who have experienced intimate partner violence (WEAVE): a cluster randomised controlled trial Hegarty et al. | Public Health and General Preventive Medicine | 7/20/2013  Lancet | The World Health Organization endorses early intervention in the primary care setting for intimate partner violence (IPV),^150^ but there is often a lack of structured interventions for physicians to use for the issue of IPV. Evidence for the effectiveness of IPV screening instruments has guided recommendations by the United States Preventive Service Task Force (USPSTF) in use of this intervention, but do not make specific recommendations on types of counseling for those who screen positive.^151^ The WEAVE study randomized physicians to provide patients care with either the Healthy Relationships Training Program in addition to basic IPV education or routine care with basic IPV education. In this study, there were no differences in quality of life (63.5 [n=96] vs. 62.2 [n=100]; p=0.50), or mental health SF-12 (47% vs. 52%; p=0.52) between those assigned to the intervention group and those assigned to the control group in women who screened positive for fear of a partner. This is a reversal of the practice of counselling with the use of the Healthy Relationships Training Program in the primary care setting for women who have experience intimate partner violence. | None found |  |
| 45 | Exercise for depression in elderly residents of care homes: a cluster-randomised controlled trial Underwood et al. | Psychiatry | 7/6/2013  Lancet | Depression is common among residents of care homes. Many studies have evaluated different types of exercise interventions in different older adult populations, with varying degrees of efficacy in treating depression.^152-155^ The 2008 Physical Activity Guidelines for Americans has found strong evidence that physical activity helps with depression in adults and older adults, but these guidelines were for the older adult population, in general, and not for specific subgroups of older adults.^156^ In this study, the addition of a moderately intense exercise program to adults 65 years and older did not reduce depressive symptoms in residents of care homes compared to a depression awareness training alone. While exercise may be beneficial for other reasons, this is a reversal of the practice of exercise for alleviating depression in older adults residing in resident care homes. | None found. Because of the results of this trial, updates to the Cochrane review will include strata-specific results to account for heterogeneity in the older adult population.^157^ | exercise and depression in older adults |
| 46 | Community treatment orders for patients with psychosis (OCTET): a randomised controlled trial Burns et al. | Psychiatry | 5/11/2013  Lancet | Community treatment orders (CTOs) are legal orders that are part of 17A of the Mental Health Act. They were introduced in England and Wales in 2008, and require patients to accept clinical monitoring and treatment while living in the community.^158 159^ In this study, the imposition of compulsory supervision did not reduce the number of readmission of psychotic patients (36% [n=166] vs. 36% [n=167]; RR=1.0; 95% CI=0.75-1.33). This is a reversal of the practice of community treatment orders for patients with psychosis. | 2017. Cochrane review. “Compulsory community treatment results in no significant difference in service use, social functioning or quality of life compared with standard care.”^160^ | PubMed suggestion |
| 47 | Population deworming every 6 months with albendazole in 1 million pre-school children in north India: DEVTA, a cluster-randomised trial Awasthi et al. | Public Health and General Preventive Medicine | 4/27/2013  Lancet | Worm infection in children can lead to various health conditions, including vitamin deficiencies and cognitive impairment. Results from a Cochrane review on whether deworming is an effective intervention are mixed.^161-163^ The World Health Organization recommends deworming strategies in the pre-school and school-age populations.^164^ Community deworming in this trial did not lead to lower mortality rates in India (deaths per child-care center: 3.00 [albendazole] vs. 3.16 [control]). This is a reversal of the practice of population deworming every 6 months with albendazole for pre-school children in India. | 2017. “Deworming did not show consistent benefits for indicators of mortality, anemia, or growth in children younger than five or women of reproductive age. We do not recommend including the effect of deworming in the LiST model.”^165^ | GoogleScholar cited RCT |
| 48 | Biolimus-eluting biodegradable polymer-coated stent versus durable polymer-coated sirolimus-eluting stent in unselected patients receiving percutaneous coronary intervention (SORT OUT V): a randomised non-inferiority trial Christiansen et al. | Cardiovascular Disease | 2/23/2013  Lancet | The biolimus-eluting stent (Nobori) was developed to reduce the risk of restenosis with a controlled release of antiproliferative drugs with a degradation of the polymer. They were approved by the European Economic Area in 2008.^166 167^ Not only was the biolimus-eluting stent found to not be non-inferior compared to a sirolimus-eluting stent in this trial, there were more incidences of stent thrombosis among those with biolimus-eluting stents (4.1% [n=1,229] vs. 3.8% [n=1,239]; p(non-inferiority)=0.06). Further, these stents did not improve rates of cardiac death, myocardial infarction, or definite stent thrombosis. This is a reversal of the practice of biolimus-eluting stents in patients receiving percutaneous coronary intervention. | A SR/MA of RCTs concluded that biolimus-eluting stents were not better than sirolimus-eluting stents at reducing mortality, myocardial infarction, or major adverse cardiac events.^168^ The RCT was not included in this review. | GoogleScholar cited RCT |
| 49 | Emergency department treatments and physiotherapy for acute whiplash: a pragmatic, two-step, randomised controlled trial Lamb et al. | Orthopedic | 2/16/2013  Lancet | The chronic symptoms that can result from whiplash can be economically burdensome in terms of treatment and loss of work productivity. Guidelines recommend physiotherapy even though this intervention is not supported by evidence.^169-171^ The Whiplash Book was developed to educate patients on managing whiplash.^171^ This book, published in 2004, has been studied in multiple populations and can now be found on Amazon.com.^172-174^ In this two-step trial, neck disability scores were no different between patients who received the Whiplash Book (n=2,253) and those who did not receive the book (n=1,598; difference at 12 months 0.5, 95% CI=-1.5 to 2.5). This is a reversal of active management with the use of the Whiplash Book in patients who present at the emergency department for acute whiplash. | In one SR/MA of RCTs that did not include this study, the authors conclude that therapeutic exercise was beneficial in improving short-term and intermediate-term pain and disablilty.^175^ A Cochrane review concluded that there was insufficient high quality evidence to make a conclusion about the effectiveness of exercise for neck pain.^176^ However, this review also did not include this study since the intervention was a "multimodal treatment". | GoogleScholar cited RCT |
| 50 | Antimicrobial catheters for reduction of symptomatic urinary tract infection in adults requiring short-term catheterisation in hospital: a multicentre randomised controlled trial Pickard et al. | Urology | 12/1/2012  Lancet | Urinary tract infections are common among patients with catheters. Several antimicrobial catheters are widely available, with the intent of reducing catheter-associated infections. These include silver alloy-coated and nitrofural-impregnated ones, silver agents being the most commonly used for catheters.^177^ A Cochrane Review has reported that while multiple studies have examined the effectiveness of these devices, the results are equivocal.^178^ Compared with traditional polytetrafluoroethylene catheters, silver alloy-coated catheters did not reduce symptomatic urinary tract infections in this trial (12.5% [n=2,097] vs. 12.6% [n=2,144]). This is a reversal of the practice of silver alloy-coated catheters for preventing urinary tract infection in patients requiring short-term catheterization. | None found | antimicrobial catheters and urinary tract infection |
| 51 | Screening for type 2 diabetes and population mortality over 10 years (ADDITION-Cambridge): a cluster-randomised controlled trial Simmons et al. | Public Health and General Preventive Medicine | 11/17/2012  Lancet | Thirty-five percent of people in England have prediabetes^179^ and many of these individuals will progress to type 2 diabetes. The current UK National Health Service (NHS) Health Checks program includes an assessment of diabetes risk for all individuals 40–74 years of age.^180 181^ All-cause, cardiovascular, or diabetes-related mortality was not decreased with the implementation of diabetes screening programs in this study. The mortality hazard ratio was 1.06 (95% CI=0.90-1.25). This is a reversal of the practice of screening all individuals 40-74 years of age for diabetes. | 2015. United States Preventive Services Task Force. “Screening for diabetes did not improve mortality rates after 10 years of follow-up.”^182^ | PubMed suggestion |
| 52 | Comparison of annual versus twice-yearly mass azithromycin treatment for hyperendemic trachoma in Ethiopia: a cluster-randomised trial Gebre et al. | Infectious Disease | 1/14/2012  Lancet | Azithromycin is the standard treatment for people with *Chlamydia trachomatis* of the eye. The WHO supports an annual mass treatment of chlamydia infection with azithromycin but for children in hyperendemic areas (>10% of children 1-9 years of age infected), three treatment cycles should before reassessment.^183-186^ Some have recommended and used biannual treatment of trachoma in hyperendemic areas.^187-189^ In this trial, ocular chlamydia infection was no different in those treated once yearly as compared to those who received treatment twice yearly (p>0.99). This is a reversal of the practice of the additional mass azithromycin treatment for trachoma, as a once-yearly treatment is adequate. | None found | azithromycin for endemic trachoma |
| 53 | Sertraline or mirtazapine for depression in dementia (HTA-SADD): a randomised, multicentre, double-blind, placebo-controlled trial Banerjee et al. | Psychiatry | 7/30/2011  Lancet | Sertraline and mirtazapine are commonly prescribed for depression in older adults, and mirtazapine is recommended as a first-line treatment for depression in clinical guidelines, regardless of age.^190-193^ The results from this trial show that neither sertraline (n=107; mean difference=1.17; 95% CI=-0.23 to 2.58; p=0.10) nor mirtazapine (n=108; mean difference=0.01; 95% CI=-1.37 to 1.38; p=0.99) improved rates of depression over placebo (n=111) in those with Alzheimer's disease. This is a reversal of the practice of using traditional treatments for depression, such as sertraline or mirtazapine, in patients with Alzheimer’s, as depression in this population may have different mechanisms than that of the general population. | 2017. “We found no significant drug-placebo difference for depressive symptoms. Overall quality of the evidence was moderate because of methodological limitations in studies and the small number of trials.”^194^ | GoogleScholar - cited this RCT |
| 54 | Prednisone versus tamoxifen in patients with idiopathic retroperitoneal fibrosis: an open-label randomised controlled trial Vaglio et al. | Nephrology | 7/23/2011  Lancet | Idiopathic retroperitoneal fibrosis is a rare disease with no established treatment. Several treatments, including glucocorticoids, immunosuppressive drugs, and tamoxifen, have been used anecdotally, but none of these treatments have been tested for efficacy. The most commonly used treatment at the time of the study was glucocorticoid therapy but tamoxifen therapy had also become a generally accepted treatment.^195-197^ In this RCT, relapse in idiopathic retroperitoneal fibrosis was more common in the tamoxifen compared to prednisone in this trial (39% vs. 6%; p=0.04; n=20 in each group), even though an equal amount of patients in each group achieved remission initially. This is a reversal of tamoxifen treatment for idiopathic retroperitoneal fibrosis. | None found | treatment for idiopathic retroperitoneal fibrosis |
| 55 | Urinary incontinence in men after formal one-to-one pelvic-floor muscle training following radical prostatectomy or transurethral resection of the prostate (MAPS): two parallel randomised controlled trials Glazener et al. | Urology | 7/23/2011  Lancet | Urinary incontinence is a common side effect of radical prostatectomy for the treatment of localized prostate cancer. Pelvic-floor muscle training is effective for urinary incontinence in women and is well established. Although less established in men, this type of therapy is also recommended for men with urinary incontinence.^198 199^ In this trial, pelvic floor exercises, compared to standard care, did not improve urinary incontinence in men who had been treated for prostate cancer or benign prostatic enlargement (radical prostatectomy trial; 76% [n=196] vs. 77% [n=195]; p=0.64; transurethral resection of the prostate trial: 65% [n=194] vs. 62% [n=203]; p=0.47). This is a reversal of the practice of pelvic floor muscle training to prevent urinary incontinence in men who have undergone radical prostatectomy. | 2015. Cochrane review. “There was no evidence from eight trials that pelvic floor muscle training with or without biofeedback was better than control for men who had urinary incontinence up to 12 months after radical prostatectomy.”^200^ | PubMed suggestion |
| 56 | The angiotensin-receptor blocker candesartan for treatment of acute stroke (SCAST): a randomised, placebo-controlled, double-blind trial Sandset et al. | Neurology/Neurosurgery | 2/26/2011  Lancet | At the time of this study, there was considerable debate as to whether or not to treat hypertension immediately after a stroke. Guidelines leaned toward not treating hypertensive stroke victims, unless they are very hypertensive, because of the lack of evidence, but also suggest that starting of antihypertensive therapy within 24 hours is "relatively safe".^201^ Evidence showed that adverse outcomes were associated with both high and low blood pressure immediately after a stroke, and multiple studies have examined the effects of antihypertensive therapy.^202 203^ Candesartan is a commonly used drug for hypertension that was approved by the FDA in 2000.^204^ Even though blood pressure was reduced in the candesartan group in this study, it did not lead to better cardiovascular outcomes (composite of vascular death, MI, or stroke) compared to placebo (aHR=1.09; 95 % CI=0.84-1.41; p=0.52). This is a reversal of the routine practice of improving blood pressure with candesartan immediately after a stroke. | 2014. Cochrane review. “There is insufficient evidence that lowering blood pressure during the acute phase of stroke improves functional outcome.”^205^ Another recent SR/MA concluded that early blood pressure lowering may increase death after acute stroke, and the authors do not support the use of this practice.^206^ | PubMed suggestion |
| 57 | High-dose vitamin D3 during intensive-phase antimicrobial treatment of pulmonary tuberculosis: a double-blind randomised controlled trial Martineau et al. | Pulmonary Disease | 1/15/2011  Lancet | High doses of vitamin D have been used in the treatment of tuberculosis before antibacterial chemotherapy in the 1950's because of the observation that patients who took cod liver oil had improvements in their tuberculosis disease status.^207^ Better understanding of the biologic mechanisms of vitamin D has led to a revived interest in this as a treatment for tuberculosis.^208-210^ In this study, vitamin D, compared to placebo, did not improve time to sputum culture conversion among patients (62 assigned to intervention and 64 assigned to placebo) with pulmonary tuberculosis (aHR=1.39; 95% CI=0.90-2.16). This is a reversal of the practice of high-dose vitamin D3 during antimicrobial treatment of tuberculosis. | 2016. Cochrane review. “Although blood levels of some vitamins may be low in people starting treatment for active tuberculosis, there is currently no reliable evidence that routinely supplementing above recommended daily amounts has clinical benefits.”^211^ | PubMed suggestion |

1. Hawton K, Ratnayeke L, Simkin S, et al. Evaluation of acceptability and use of lockable storage devices for pesticides in Sri Lanka that might assist in prevention of self-poisoning. *BMC public health* 2009;9(1):69.

2. Organization WH. Safer access to pesticides: experiences from community interventions, 2016.

3. Organization WH. Guns, knives, and pesticides: reducing access to lethal means. 2009

4. Vloothuis JD, van Wegen EE, Veerbeek JM, et al. Caregiver-mediated exercises for improving outcomes after stroke. *Cochrane Database Syst Rev* 2014

5. Hudetz J, Iqbal Z, Gandhi S, et al. Ketamine attenuates post‐operative cognitive dysfunction after cardiac surgery. *Acta Anaesthesiologica Scandinavica* 2009;53(7):864-72.

6. Mu JL, Lee A, Joynt GM. Pharmacologic agents for the prevention and treatment of delirium in patients undergoing cardiac surgery: systematic review and metaanalysis. *Critical care medicine* 2015;43(1):194-204.

7. Bhatt S, Gething PW, Brady OJ, et al. The global distribution and burden of dengue. *Nature* 2013;496(7446):504.

8. Lee T-H, Wong JG, Leo Y-S, et al. Potential harm of prophylactic platelet transfusion in adult dengue patients. *PLoS neglected tropical diseases* 2016;10(3):e0004576.

9. Whitehorn J, Roche RR, Guzman MG, et al. Prophylactic platelets in dengue: survey responses highlight lack of an evidence base. *PLoS neglected tropical diseases* 2012;6(6):e1716.

10. Lee DJ, Warner CH, Hoge CW. Advances and controversies in military posttraumatic stress disorder screening. *Current psychiatry reports* 2014;16(9):467. doi: 10.1007/s11920-014-0467-7 [published Online First: 2014/07/16]

11. Zamorski MA, Rusu C, Garber BG. Prevalence and correlates of mental health problems in canadian forces personnel who deployed in support of the mission in Afghanistan: Findings from postdeployment Screenings, 2009–2012. *The Canadian Journal of Psychiatry* 2014;59(6):319-26.

12. Vermetten E, Greenberg N, Boeschoten MA, et al. Deployment-related mental health support: comparative analysis of NATO and allied ISAF partners. *European Journal of Psychotraumatology* 2014;5(1):23732.

13. Thomsen HS. European Society of Urogenital Radiology guidelines on contrast media application. *Current opinion in urology* 2007;17(1):70-76.

14. National CGCU. Acute Kidney Injury: Prevention, Detection and Management Up to the Point of Renal Replacement Therapy. 2013

15. Mayfield S, Bogossian F, O'malley L, et al. High‐flow nasal cannula oxygen therapy for infants with bronchiolitis: Pilot study. *Journal of paediatrics and child health* 2014;50(5):373-78.

16. Hough JL, Shearman AD, Jardine LA, et al. Humidified high flow nasal cannulae: current practice in Australasian nurseries, a survey. *Journal of paediatrics and child health* 2012;48(2):106-13.

17. Roehr CC, Yoder BA, Davis PG, et al. Evidence Support and guidelines for using heated, humidified, high-flow nasal cannulae in neonatology. *Clinics in perinatology* 2016;43(4):693-705.

18. Monro-Somerville T, Sim M, Ruddy J, et al. The effect of high-flow nasal cannula oxygen therapy on mortality and intubation rate in acute respiratory failure: a systematic review and meta-analysis. *Critical care medicine* 2017;45(4):e449-e56.

19. Serruys PW, Chevalier B, Sotomi Y, et al. Comparison of an everolimus-eluting bioresorbable scaffold with an everolimus-eluting metallic stent for the treatment of coronary artery stenosis (ABSORB II): a 3 year, randomised, controlled, single-blind, multicentre clinical trial. *The Lancet* 2016;388(10059):2479-91.

20. Onuma Y, Ormiston J, Serruys PW. Bioresorbable scaffold technologies. *Circulation Journal* 2011;75(3):509-20.

21. Mahmoud AN, Barakat AF, Elgendy AY, et al. Long-term efficacy and safety of everolimus-eluting bioresorbable vascular scaffolds versus everolimus-eluting metallic stents: a meta-analysis of randomized trials. *Circulation: Cardiovascular Interventions* 2017;10(5):e005286.

22. Lacerda-Filho A, Silva RGd. Stapled hemorrhoidectomy: present status. *Arquivos de gastroenterologia* 2005;42(3):191-94.

23. Nisar PJ, Acheson AG, Neal KR, et al. Stapled hemorrhoidopexy compared with conventional hemorrhoidectomy: systematic review of randomized, controlled trials. *Diseases of the colon & rectum* 2004;47(11):1837-45.

24. Lumb KJ, Colquhoun PH, Malthaner R, et al. Stapled versus conventional surgery for hemorrhoids. *The Cochrane Library* 2006

25. Burch J, Epstein D, Baba-Akbari A, et al. Stapled haemorrhoidectomy (haemorrhoidopexy) for the treatment of haemorrhoids: a systematic review and economic evaluation. *HEALTH TECHNOLOGY ASSESSMENT-SOUTHAMPTON-* 2008;12(8)

26. Fogg B, Cuellar G, Danielson D. Motivating, influencing, and persuading users: An introduction to captology. *Human Computer Interaction Fundamentals* 2009:109-22.

27. Site HIS. Working to Find and advance solutions to environment, health, poverty, and development problems. Realityworks infant simulator and RealCare Parenting Program 2011 [Available from: <http://www.solutions-site.org/node/222>.

28. it RLiL. Live it Learn it Experiential Learning Technology. 2012 [Available from: <https://www.realityworks.com/news-events/8-Realityworks-Releases-New-Version-of-RealCare-Baby>.

29. Pollack A. For some, aspirin may not help hearts. *New York Times* 2004

30. Gurbel PA, Becker RC, Mann KG, et al. Platelet function monitoring in patients with coronary artery disease. *Journal of the American College of Cardiology* 2007;50(19):1822-34.

31. Aradi D, Storey RF, Komócsi A, et al. Expert position paper on the role of platelet function testing in patients undergoing percutaneous coronary intervention. *European heart journal* 2014;35(4):209.

32. Choi J-L, Li S, Han J-Y. Platelet function tests: a review of progresses in clinical application. *BioMed research international* 2014;2014

33. Xing Z, Tang L, Zhu Z, et al. Platelet reactivity-adjusted antiplatelet therapy in patients with percutaneous coronary intervention: a meta-analysis of randomized controlled trials. *Platelets* 2017:1-7.

34. Chao JH, Phillips R, Nickson JJ. Roentgen‐ray therapy of cerebral metastases. *Cancer* 1954;7(4):682-89.

35. Order SE, Hellmän S, Von Essen CF, et al. Improvement in quality of survival following whole-brain irradiation for brain metastasis. *Radiology* 1968;91(1):149-53.

36. Tsao MN, Xu W, Wong RK, et al. Whole brain radiotherapy for the treatment of newly diagnosed multiple brain metastases. *The Cochrane Library* 2018

37. Binder J, Kramer W. Robotically‐assisted laparoscopic radical prostatectomy. *BJU international* 2001;87(4):408-10.

38. Yaxley JW, Coughlin GD, Chambers SK, et al. Robot-assisted laparoscopic prostatectomy versus open radical retropubic prostatectomy: early outcomes from a randomised controlled phase 3 study. *The Lancet* 2016;388(10049):1057-66.

39. Ilic D, Evans SM, Allan CA, et al. Laparoscopic and robot‐assisted vs open radical prostatectomy for the treatment of localized prostate cancer: a Cochrane systematic review. *BJU international* 2017

40. Peytel E, Menegaux F, Cluzel P, et al. Initial imaging assessment of severe blunt trauma. *Intensive care medicine* 2001;27(11):1756-61.

41. Leidner B, Beckman M. Standardized whole-body computed tomography as a screening tool in blunt multitrauma patients. *Emergency Radiology* 2001;8(1):20-28.

42. Chidambaram S, Goh EL, Khan MA. A meta-analysis of the efficacy of whole-body computed tomography imaging in the management of trauma and injury. *Injury* 2017

43. Lovelock C, Molyneux A, Rothwell P. Change in incidence and aetiology of intracerebral haemorrhage in Oxfordshire, UK, between 1981 and 2006: a population-based study. *The Lancet Neurology* 2007;6(6):487-93.

44. Baharoglu MI, Cordonnier C, Salman RA-S, et al. Platelet transfusion versus standard care after acute stroke due to spontaneous cerebral haemorrhage associated with antiplatelet therapy (PATCH): a randomised, open-label, phase 3 trial. *The Lancet* 2016;387(10038):2605-13.

45. Leong LB, David TKP. Is platelet transfusion effective in patients taking antiplatelet agents who suffer an intracranial hemorrhage? *The Journal of emergency medicine* 2015;49(4):561-72.

46. El-Toukhy T, Campo R, Khalaf Y, et al. Hysteroscopy in recurrent in-vitro fertilisation failure (TROPHY): a multicentre, randomised controlled trial. *The Lancet* 2016;387(10038):2614-21.

47. El-Toukhy T, Sunkara SK, Coomarasamy A, et al. Outpatient hysteroscopy and subsequent IVF cycle outcome: a systematic review and meta-analysis. *Reproductive biomedicine online* 2008;16(5):712-19.

48. Medicine PCotASfR. Diagnostic evaluation of the infertile female: a committee opinion. *Fertility and sterility* 2012;98(2):302-07.

49. Bosteels J, Kasius J, Weyers S, et al. Hysteroscopy for treating subfertility associated with suspected major uterine cavity abnormalities. *The Cochrane Library* 2015

50. Morris JM, Roberts CL, Bowen JR, et al. Immediate delivery compared with expectant management after preterm pre-labour rupture of the membranes close to term (PPROMT trial): a randomised controlled trial. *The Lancet*;387(10017):444-52. doi: 10.1016/S0140-6736(15)00724-2

51. Premature rupture of the membranes: neonatal consequences. Seminars in perinatology; 1996. Elsevier.

52. Bond DM, Middleton P, Levett KM, et al. Planned early birth versus expectant management for women with preterm prelabour rupture of membranes prior to 37 weeks' gestation for improving pregnancy outcome. *The Cochrane Library* 2017

53. Robling M, Bekkers M-J, Bell K, et al. Effectiveness of a nurse-led intensive home-visitation programme for first-time teenage mothers (Building Blocks): a pragmatic randomised controlled trial. 2016;387(10014):146-55.

54. Olds DL. The nurse-family partnership: An evidence-based preventive intervention. *Infant mental health journal* 2006;27(1):5-25. doi: 10.1002/imhj.20077 [published Online First: 2006/01/01]

55. Svilaas T, Vlaar PJ, van der Horst IC, et al. Thrombus aspiration during primary percutaneous coronary intervention. *New England Journal of Medicine* 2008;358(6):557-67.

56. Vlaar PJ, Svilaas T, van der Horst IC, et al. Cardiac death and reinfarction after 1 year in the Thrombus Aspiration during Percutaneous coronary intervention in Acute myocardial infarction Study (TAPAS): a 1-year follow-up study. *The Lancet* 2008;371(9628):1915-20.

57. Fröbert O, Lagerqvist B, Olivecrona GK, et al. Thrombus aspiration during ST-segment elevation myocardial infarction. *New England journal of medicine* 2013;369(17):1587-97.

58. Lagerqvist B, Fröbert O, Olivecrona GK, et al. Outcomes 1 year after thrombus aspiration for myocardial infarction. *New England Journal of Medicine* 2014;371(12):1111-20.

59. Kushner FG, Hand M, Smith SC, et al. 2009 Focused Updates: ACC/AHA Guidelines for the Management of Patients With ST‐Elevation Myocardial Infarction (Updating the 2004 Guideline and 2007 Focused Update) and ACC/AHA/SCAI Guidelines on Percutaneous Coronary Intervention (Updating the 2005 Guideline and 2007 Focused Update). *Catheterization and Cardiovascular Interventions* 2009;74(7)

60. Costopoulos C, Gorog DA, Di Mario C, et al. Use of thrombectomy devices in primary percutaneous coronary intervention: a systematic review and meta-analysis. *International journal of cardiology* 2013;163(3):229-41.

61. Elgendy AY, Elgendy IY, Mahmoud AN, et al. Long‐term outcomes with aspiration thrombectomy for patients undergoing primary percutaneous coronary intervention: A meta‐analysis of randomized trials. *Clinical Cardiology* 2017

62. Marik PE. Aspiration pneumonitis and aspiration pneumonia. *New England Journal of Medicine* 2001;344(9):665-71.

63. Westendorp WF, Vermeij JD, Vermeij F, et al. Antibiotic therapy for preventing infections in patients with acute stroke. *The Cochrane Library* 2012

64. Vermeij JD, Westendorp WF, Dippel DW, et al. Antibiotic therapy for preventing infections in people with acute stroke. *The Cochrane Library* 2018

65. D'Haens G, Baert F, Van Assche G, et al. Early combined immunosuppression or conventional management in patients with newly diagnosed Crohn's disease: an open randomised trial. *The Lancet* 2008;371(9613):660-67.

66. Colombel JF, Sandborn WJ, Reinisch W, et al. Infliximab, azathioprine, or combination therapy for Crohn's disease. *New England Journal of Medicine* 2010;362(15):1383-95.

67. Travis S, Stange E, Lemann M, et al. European evidence based consensus on the diagnosis and management of Crohn’s disease: current management. *Gut* 2006;55(suppl 1):i16-i35.

68. Dignass A, Van Assche G, Lindsay J, et al. The second European evidence-based consensus on the diagnosis and management of Crohn's disease: current management. *Journal of Crohn's and Colitis* 2010;4(1):28-62.

69. Horrocks E, Thin N, Thaha M, et al. Systematic review of tibial nerve stimulation to treat faecal incontinence. *British Journal of Surgery* 2014;101(5):457-68.

70. Leroi A, Siproudhis L, Etienney I, et al. Transcutaneous electrical tibial nerve stimulation in the treatment of fecal incontinence: a randomized trial (CONSORT 1a). *The American journal of gastroenterology* 2012;107(12):1888-96.

71. Hotouras A, Thaha M, Allison M, et al. Percutaneous tibial nerve stimulation (PTNS) in females with faecal incontinence: the impact of sphincter morphology and rectal sensation on the clinical outcome. *International journal of Colorectal disease* 2012;27(7):927-30.

72. Edenfield AL, Amundsen CL, Wu JM, et al. Posterior tibial nerve stimulation for the treatment of fecal incontinence: a systematic evidence review. *Obstetrical & gynecological survey* 2015;70(5):329-41.

73. Sauerland S, Nagelschmidt M, Mallmann P, et al. Risks and benefits of preoperative high dose methylprednisolone in surgical patients. *Drug Safety* 2000;23(5):449-61.

74. Chaney MA. Corticosteroids and cardiopulmonary bypass: a review of clinical investigations. *Chest* 2002;121(3):921-31.

75. Ali-Hassan-Sayegh S, Mirhosseini SJ, Haddad F, et al. Protective effects of corticosteroids in coronary artery bypass graft surgery alone or combined with valvular surgery: an updated and comprehensive meta-analysis and systematic review. *Interactive cardiovascular and thoracic surgery* 2015;20(6):825-36.

76. Campschroer T, Zhu Y, Duijvesz D, et al. Alpha‐blockers as medical expulsive therapy for ureteral stones. *The Cochrane Library* 2014

77. Seitz C, Liatsikos E, Porpiglia F, et al. Medical therapy to facilitate the passage of stones: what is the evidence? *European urology* 2009;56(3):455-71.

78. Wang H, Man LB, Huang GL, et al. Comparative efficacy of tamsulosin versus nifedipine for distal ureteral calculi: a meta-analysis. *Drug design, development and therapy* 2016;10:1257.

79. Hollingsworth JM, Canales BK, Rogers MA, et al. Alpha blockers for treatment of ureteric stones: systematic review and meta-analysis. *bmj* 2016;355:i6112.

80. Bernhardt J, Langhorne P, Lindley RI, et al. Efficacy and safety of very early mobilisation within 24 h of stroke onset (AVERT): a randomised controlled trial. 2015;386(9988):46-55.

81. Xu T, Yu X, Ou S, et al. Efficacy and Safety of Very Early Mobilization in Patients with Acute Stroke: A Systematic Review and Meta-analysis. *Scientific reports* 2017;7(1):6550.

82. Strom BL, Schinnar R, Aberra F, et al. Unintended effects of a computerized physician order entry nearly hard-stop alert to prevent a drug interaction: a randomized controlled trial. *Archives of internal medicine* 2010;170(17):1578-83.

83. Strom BL, Schinnar R, Bilker W, et al. Randomized clinical trial of a customized electronic alert requiring an affirmative response compared to a control group receiving a commercial passive CPOE alert: NSAID—warfarin co-prescribing as a test case. *Journal of the American Medical Informatics Association* 2010;17(4):411-15.

84. Kucher N, Koo S, Quiroz R, et al. Electronic alerts to prevent venous thromboembolism among hospitalized patients. *N Engl j Med* 2005;2005(352):969-77.

85. Dexter PR, Perkins S, Overhage JM, et al. A computerized reminder system to increase the use of preventive care for hospitalized patients. *New England Journal of Medicine* 2001;345(13):965-70.

86. Selby NM, Crowley L, Fluck RJ, et al. Use of electronic results reporting to diagnose and monitor AKI in hospitalized patients. *Clinical Journal of the American Society of Nephrology* 2012;7(4):533-40.

87. Porter CJ, Juurlink I, Bisset LH, et al. A real-time electronic alert to improve detection of acute kidney injury in a large teaching hospital. *Nephrology Dialysis Transplantation* 2014;29(10):1888-93.

88. Colpaert K, Hoste EA, Steurbaut K, et al. Impact of real-time electronic alerting of acute kidney injury on therapeutic intervention and progression of RIFLE class. *Critical care medicine* 2012;40(4):1164-70.

89. Haase M, Kribben A, Zidek W, et al. Electronic Alerts for Acute Kidney Injury: A Systematic Review. *Deutsches Ärzteblatt International* 2017;114(1-2):1.

90. Okumu FO, Mbeyela E, Lingamba G, et al. Comparative field evaluation of combinations of long-lasting insecticide treated nets and indoor residual spraying, relative to either method alone, for malaria prevention in an area where the main vector is Anopheles arabiensis. *Parasites & vectors* 2013;6(1):46.

91. Fullman N, Burstein R, Lim SS, et al. Nets, spray or both? The effectiveness of insecticide-treated nets and indoor residual spraying in reducing malaria morbidity and child mortality in sub-Saharan Africa. *Malaria journal* 2013;12(1):62.

92. Kleinschmidt I, Schwabe C, Shiva M, et al. Combining indoor residual spraying and insecticide-treated net interventions. *The American journal of tropical medicine and hygiene* 2009;81(3):519-24.

93. Feng J. The Effect of Combining Insecticide Treated Nets and Indoor Residual Spraying for Vector Control of Malaria: A Systematic Review, 2017.

94. Imdad A, Ahmed Z, Bhutta ZA. Vitamin A supplementation for the prevention of morbidity and mortality in infants one to six months of age. *The Cochrane Library* 2016

95. Gogia S, Sachdev HS. Vitamin A supplementation for the prevention of morbidity and mortality in infants six months of age or less. *Cochrane Database Syst Rev* 2011;10

96. Organization WH. Report of the WHO technical consultation on neonatal vitamin A supplementation research priorities, Geneva, Switzerland, 4-5 December 2008, 2009.

97. Ross DA. Recommendations for vitamin A supplementation. *The Journal of nutrition* 2002;132(9):2902S-06S.

98. Haider BA, Sharma R, Bhutta ZA. Neonatal vitamin A supplementation for the prevention of mortality and morbidity in term neonates in low and middle income countries. *The Cochrane Library* 2017

99. World Health Organization G. The selection and use of essential medicines: report of the WHO Expert Committee, 2013 (including the 18th WHO Model List of Essential Medicines and the 4th WHO Model List of Essentials Medicines for Children). , 2013.

100. Costello A, Azad K. Scaling up antenatal corticosteroids in low-resource settings? *The Lancet* 2015;385(9968):585-87.

101. Lawn J, Segrè J, Buekens P, et al. Antenatal corticosteroids for the reduction of deaths in preterm babies. *A case study prepared for the United Nation’s Commission on Commodities for Women’s and children’s health* 2012

102. Kupferminc MJ, Eldor A, Steinman N, et al. Increased frequency of genetic thrombophilia in women with complications of pregnancy. *New England Journal of Medicine* 1999;340(1):9-13.

103. Bates SM, Greer IA, Hirsh J, et al. Use of antithrombotic agents during pregnancy: the Seventh ACCP Conference on Antithrombotic and Thrombolytic Therapy. *Chest Journal* 2004;126(3_suppl):627S-44S.

104. Gris J-C, Mercier E, Quéré I, et al. Low-molecular-weight heparin versus low-dose aspirin in women with one fetal loss and a constitutional thrombophilic disorder. *Blood* 2004;103(10):3695-99.

105. Brenner B, Hoffman R, Carp H, et al. Efficacy and safety of two doses of enoxaparin in women with thrombophilia and recurrent pregnancy loss: the LIVE‐ENOX study. *Journal of Thrombosis and Haemostasis* 2005;3(2):227-29.

106. Skeith L, Carrier M, Kaaja R, et al. A meta-analysis of low-molecular-weight heparin to prevent pregnancy loss in women with inherited thrombophilia. *Blood* 2016:blood-2015-12-626739.

107. Chou R, Qaseem A, Snow V, et al. Diagnosis and Treatment of Low Back Pain: A Joint Clinical Practice Guideline from the American College of Physicians and the American Pain SocietyDiagnosis and Treatment of Low Back Pain. *Annals of internal medicine* 2007;147(7):478-91.

108. Koes BW, van Tulder M, Lin C-WC, et al. An updated overview of clinical guidelines for the management of non-specific low back pain in primary care. *European Spine Journal* 2010;19(12):2075-94.

109. Machado GC, Maher CG, Ferreira PH, et al. Efficacy and safety of paracetamol for spinal pain and osteoarthritis: systematic review and meta-analysis of randomised placebo controlled trials. *bmj* 2015;350:h1225.

110. Lee YG. Surgical resection of mesothelioma: an evidence-free practice. *The Lancet* 2014;384(9948):1080-81.

111. Nakas A, Martin Ucar AE, Edwards JG, et al. The role of video assisted thoracoscopic pleurectomy/decortication in the therapeutic management of malignant pleural mesothelioma. *European Journal of Cardio-Thoracic Surgery* 2008;33(1):83-88.

112. Halstead J, Lim E, Venkateswaran R, et al. Improved survival with VATS pleurectomy-decortication in advanced malignant mesothelioma. *European Journal of Surgical Oncology (EJSO)* 2005;31(3):314-20.

113. Lewis RJ, Caccavale RJ, Sisler GE, et al. One hundred consecutive patients undergoing video-assisted thoracic operations. *The Annals of thoracic surgery* 1992;54(3):421-26.

114. Clive AO, Jones HE, Bhatnagar R, et al. Interventions for the management of malignant pleural effusions: a network meta‐analysis. *The Cochrane Library* 2016

115. Wiesen J, Ornstein M, Tonelli AR, et al. State of the evidence: mechanical ventilation with PEEP in patients with cardiogenic shock. *Heart* 2013;99(24):1812-17.

116. Duggan M, Kavanagh BP. Pulmonary AtelectasisA Pathogenic Perioperative Entity. *The Journal of the American Society of Anesthesiologists* 2005;102(4):838-54.

117. Halter JM, Steinberg JM, Schiller HJ, et al. Positive end-expiratory pressure after a recruitment maneuver prevents both alveolar collapse and recruitment/derecruitment. *American journal of respiratory and critical care medicine* 2003;167(12):1620-26.

118. Zaky A, Lang JD. The use of intraoperative positive end expiratory pressure. *J Anesthe Clinic Res* 2011;4(308):2.

119. Barbosa FT, Castro AA, de Sousa‐Rodrigues CF. Positive end‐expiratory pressure (PEEP) during anaesthesia for prevention of mortality and postoperative pulmonary complications. *The Cochrane Library* 2014

120. Van Rheenen P. Delayed cord clamping and improved infant outcomes: British Medical Journal Publishing Group, 2011.

121. Barrios JZ, Albornoz G, Musante G, et al. Timing of umbilical cord clamping of term infants in a large maternity center in Buenos Aires. *Journal of Perinatal Medicine* 2011;39

122. Committee SA. Clamping of the umbilical cord and placental transfusion. *London: RCOG* 2009

123. Airey RJ, Farrar D, Duley L. Alternative positions for the baby at birth before clamping the umbilical cord. *Cochrane Database Syst Rev* 2010(10):Cd007555. doi: 10.1002/14651858.CD007555.pub2 [published Online First: 2010/10/12]

124. Mercer JS, Nelson CC, Skovgaard RL. Umbilical cord clamping: Beliefs and practices of American nurse‐midwives. *Journal of Midwifery & Women’s Health* 2000;45(1):58-66.

125. Sterling M. Clinical guidelines for best practice management of acute and chronic whiplash-associated disorders. 2008

126. Stewart MJ, Maher CG, Refshauge KM, et al. Randomized controlled trial of exercise for chronic whiplash-associated disorders. *Pain* 2007;128(1):59-68.

127. Lamb SE, Gates S, Williams MA, et al. Emergency department treatments and physiotherapy for acute whiplash: a pragmatic, two-step, randomised controlled trial. *The Lancet* 2013;381(9866):546-56.

128. Wiangkham T, Duda J, Haque M, et al. Effectiveness of conservative treatment in acute whiplash associated disorder (WAD) II: a systematic review and meta-analysis of randomised controlled trials. *Physiotherapy* 2015;101:e1623-e24.

129. Kearon C, Akl EA, Comerota AJ, et al. Antithrombotic therapy for VTE disease: antithrombotic therapy and prevention of thrombosis: American College of Chest Physicians evidence-based clinical practice guidelines. *Chest Journal* 2012;141(2_suppl):e419S-e94S.

130. Brandjes DP, Büller HR, Heijboer H, et al. Randomised trial of effect of compression stockings in patients with symptomatic proximal-vein thrombosis. *The Lancet* 1997;349(9054):759-62.

131. Prandoni P, Lensing AW, Prins MH, et al. Below-Knee Elastic Compression Stockings To Prevent the Post-Thrombotic SyndromeA Randomized, Controlled Trial. *Annals of Internal Medicine* 2004;141(4):249-56.

132. Kahn S, Elman E, Rodger M, et al. Use of elastic compression stockings after deep venous thrombosis: a comparison of practices and perceptions of thrombosis physicians and patients. *Journal of Thrombosis and Haemostasis* 2003;1(3):500-06.

133. Appelen D, van Loo E, Prins MH, et al. Compression therapy for prevention of post‐thrombotic syndrome. *The Cochrane Library* 2017

134. Ajiboye N, Chalouhi N, Starke RM, et al. Cerebral arteriovenous malformations: evaluation and management. *The Scientific World Journal* 2014;2014

135. van Beijnum J, van der Worp HB, Buis DR, et al. Treatment of brain arteriovenous malformations: a systematic review and meta-analysis. *Jama* 2011;306(18)

136. Hartmann A, Mast H, Mohr JP, et al. Determinants of staged endovascular and surgical treatment outcome of brain arteriovenous malformations. *Stroke* 2005;36(11):2431-35.

137. Bederson JB, Awad IA, Wiebers DO, et al. Recommendations for the management of patients with unruptured intracranial aneurysms. *Stroke* 2000;31(11):2742-50.

138. Currow DC, Abernethy AP. Lessons from the Liverpool Care Pathway--evidence is key. *The Lancet* 2014;383(9913):192.

139. Jack BA, Gambles M, Murphy D, et al. Nurses' perceptions of the Liverpool Care Pathway for the dying patient in the acute hospital setting. *International journal of palliative nursing* 2003;9(9)

140. Di Leo S, Beccaro M, Finelli S, et al. Expectations about and impact of the Liverpool Care Pathway for the dying patient in an Italian hospital. *Palliative medicine* 2011;25(4):293-303.

141. Veerbeek L, van Zuylen L, Swart SJ, et al. The effect of the Liverpool Care Pathway for the dying: a multi-centre study. *Palliative Medicine* 2008;22(2):145-51.

142. Chan R, Webster J. End-of-life care pathways for improving outcomes in caring for the dying. *Cochrane Database Syst Rev* 2010;1

143. Kalra L, Evans A, Perez I, et al. Training carers of stroke patients: randomised controlled trial. *Bmj* 2004;328(7448):1099.

144. Stevenson D. Training informal caregivers of patients with stroke improved patient and caregiver quality of life and reduced costs. *Evidence-based nursing* 2004;7(4):118.

145. Wang Y, Yang F, Shi H, et al. What Type of Transitional Care Effectively Reduced Mortality and Improved ADL of Stroke Patients? A Meta-Analysis. *International journal of environmental research and public health* 2017;14(5):510.

146. Thiele H, Allam B, Chatellier G, et al. Shock in acute myocardial infarction: the Cape Horn for trials? 2010;31(15):1828-35.

147. Wan Y-D, Sun T-W, Kan Q-C, et al. The effects of intra-aortic balloon pumps on mortality in patients undergoing high-risk coronary revascularization: a meta-analysis of randomized controlled trials of coronary artery bypass grafting and stenting era. *PloS one* 2016;11(1):e0147291.

148. Golub J, Mohan C, Comstock G, et al. Active case finding of tuberculosis: historical perspective and future prospects. *The International Journal of Tuberculosis and Lung Disease* 2005;9(11):1183-203.

149. Mhimbira FA, Cuevas LE, Dacombe R, et al. Interventions to increase tuberculosis case detection at primary healthcare or community‐level services. *The Cochrane Library* 2017

150. Organization WH. WHO Multi-Country Study on Women’s Health and Domestic Violence: Domestic Violence Module. *Geneva: Author Retrieved November* 2005;29:2006.

151. Nelson HD, Bougatsos C, Blazina I. Screening women for intimate partner violence: a systematic review to update the US Preventive Services Task Force recommendation. *Annals of internal medicine* 2012;156(11):796-808.

152. Bridle C, Spanjers K, Patel S, et al. Effect of exercise on depression severity in older people: systematic review and meta-analysis of randomised controlled trials. *The British Journal of Psychiatry* 2012;201(3):180-85.

153. Craft LL, Perna FM. The benefits of exercise for the clinically depressed. *Primary care companion to the Journal of clinical psychiatry* 2004;6(3):104.

154. Potter R, Ellard D, Rees K, et al. A systematic review of the effects of physical activity on physical functioning, quality of life and depression in older people with dementia. *International journal of geriatric psychiatry* 2011;26(10):1000-11.

155. Blumenthal JA, Babyak MA, Moore KA, et al. Effects of exercise training on older patients with major depression. *Archives of internal medicine* 1999;159(19):2349-56.

156. Fulton JE, Kohl HW. 2008 physical activity guidelines for Americans; be active, healthy, and happy! 2008

157. Ekkekakis P. Honey, I shrunk the pooled SMD! Guide to critical appraisal of systematic reviews and meta-analyses using the Cochrane review on exercise for depression as example. *Mental health and physical activity* 2015;8:21-36.

158. Steadman HJ, Gounis K, Dennis D, et al. Assessing the New York City involuntary outpatient commitment pilot program. *Psychiatric Services* 2001;52(3):330-36.

159. Swartz MS, Swanson JW, Wagner HR, et al. Can involuntary outpatient commitment reduce hospital recidivism?: findings from a randomized trial with severely mentally ill individuals. *American Journal of Psychiatry* 1999;156(12):1968-75.

160. Kisely SR, Campbell LA, Preston NJ. Compulsory community and involuntary outpatient treatment for people with severe mental disorders. *Cochrane Database of Systematic Reviews* 2005(3 Article No: CD004408):1-25.

161. Taylor-Robinson DC, Maayan N, Soares-Weiser K, et al. Deworming drugs for soil-transmitted intestinal worms in children: effects on nutritional indicators, haemoglobin and school performance. *Cochrane Database Syst Rev* 2012;11(7)

162. Taylor-Robinson D, Jones A, Garner P. Does deworming improve growth and school performance in children? *PLoS neglected tropical diseases* 2009;3(1):e358.

163. Bundy DA, Kremer M, Bleakley H, et al. Deworming and development: asking the right questions, asking the questions right. *PLoS Neglected Tropical Diseases* 2009;3(1):e362.

164. Organization WH. Prevention and control of schistosomiasis and soil-transmitted helminthiasis: report of a WHO expert committee. 2002

165. Thayer WM, Clermont A, Walker N. Effects of deworming on child and maternal health: a literature review and meta-analysis. *BMC public health* 2017;17(4):830.

166. Windecker S, Serruys PW, Wandel S, et al. Biolimus-eluting stent with biodegradable polymer versus sirolimus-eluting stent with durable polymer for coronary revascularisation (LEADERS): a randomised non-inferiority trial. *The Lancet* 2008;372(9644):1163-73.

167. Systems TI. Terumo receives CE Certificate for Nobori™ Drug-Eluting Stent 2008 [Available from: <https://www.terumo-europe.com/en-emea/news/terumo-receives-ce-certificate-for-nobori%E2%84%A2-drug-eluting-stent> accessed 01/22/2018.

168. Zhang Y-J, Zhu L-L, Bourantas CV, et al. The impact of everolimus versus other rapamycin derivative-eluting stents on clinical outcomes in patients with coronary artery disease: A meta-analysis of 16 randomized trials. *Journal of cardiology* 2014;64(3):185-93.

169. Hurwitz EL, Carragee EJ, van der Velde G, et al. Treatment of neck pain: noninvasive interventions: results of the Bone and Joint Decade 2000-2010 Task Force on Neck Pain and Its Associated Disorders. *Spine* 2008;33(4 Suppl):S123-52. doi: 10.1097/BRS.0b013e3181644b1d [published Online First: 2008/02/07]

170. Verhagen AP, Scholten-Peeters GG, van Wijngaarden S, et al. Conservative treatments for whiplash. *Cochrane Database Syst Rev* 2007(2):Cd003338. doi: 10.1002/14651858.CD003338.pub3 [published Online First: 2007/04/20]

171. McClune T, Burton AK, Waddell G. Evaluation of an evidence based patient educational booklet for management of whiplash associated disorders. *Emergency medicine journal : EMJ* 2003;20(6):514-7. [published Online First: 2003/11/19]

172. Amazon. 2018 [1/22/2018]. Available from: <https://www.amazon.com/Whiplash-Book-US-Canada-copies/dp/0117033251>.

173. Ferrari R, Rowe BH, Majumdar SR, et al. Simple educational intervention to improve the recovery from acute whiplash: results of a randomized, controlled trial. *Academic emergency medicine* 2005;12(8):699-706.

174. Could a simple educational intervention modify beliefs about whiplash? A preliminary study among professionals working in a rehabilitation ward. Annales de réadaptation et de médecine physique; 2007. Elsevier.

175. Bertozzi L, Gardenghi I, Turoni F, et al. Effect of therapeutic exercise on pain and disability in the management of chronic nonspecific neck pain: systematic review and meta-analysis of randomized trials. *Physical therapy* 2013;93(8):1026-36.

176. Gross A, Kay TM, Paquin JP, et al. Exercises for mechanical neck disorders. *The Cochrane Library* 2015

177. Brosnahan J, Jull A, Tracy C. Types of urethral catheters for management of short‐term voiding problems in hospitalised adults. *The Cochrane Library* 2004

178. Schumm K, Lam T. Types of urethral catheters for management of short-term voiding problems in hospitalised adults. *Cochrane Database Syst Rev* 2008;2(2)

179. Mainous AG, Tanner RJ, Baker R, et al. Prevalence of prediabetes in England from 2003 to 2011: population-based, cross-sectional study. *BMJ open* 2014;4(6):e005002.

180. Echouffo-Tcheugui JB, Simmons RK, Williams KM, et al. The ADDITION-Cambridge trial protocol: a cluster–randomised controlled trial of screening for type 2 diabetes and intensive treatment for screen-detected patients. *BMC public health* 2009;9(1):136.

181. Autier P, Boniol M, Gavin A, et al. Breast cancer mortality in neighbouring European countries with different levels of screening but similar access to treatment: trend analysis of WHO mortality database. *Bmj* 2011;343:d4411.

182. Selph S, Dana T, Blazina I, et al. Screening for Type 2 Diabetes Mellitus: A Systematic Review for the US Preventive Services Task ForceScreening for Type 2 Diabetes Mellitus. *Annals of internal medicine* 2015;162(11):765-76.

183. World Health Organization G. Report of the Eighth Meeting of the WHO Alliance for the Global Elimination of Blinding Trachoma Geneva 29−30 March, 2004. GLOBAL ELIMINATION OF BLINDING TRACHOMA BY THE YEAR 2020 WORLD HEALTH ORGANIZATION Prevention of Blindness and Deafness WHO/PBD/GET/042

2004.

184. West SK, Munoz B, Mkocha H, et al. Infection with Chlamydia trachomatis after mass treatment of a trachoma hyperendemic community in Tanzania: a longitudinal study. *The Lancet* 2005;366(9493):1296-300.

185. Solomon AW, Holland MJ, Alexander ND, et al. Mass treatment with single-dose azithromycin for trachoma. *New England Journal of Medicine* 2004;351(19):1962-71.

186. Melese M, Alemayehu W, Lakew T, et al. Comparison of annual and biannual mass antibiotic administration for elimination of infectious trachoma. *Jama* 2008;299(7):778-84.

187. Lietman T, Porco T, Dawson C, et al. Global elimination of trachoma: how frequently should we administer mass chemotherapy? *Nature medicine* 1999;5(5):572.

188. Gill DA, Lakew T, Alemayehu W, et al. Complete elimination is a difficult goal for trachoma programs in severely affected communities. *Clinical infectious diseases* 2008;46(4):564-66.

189. Yang JL, Hong KC, Schachter J, et al. Detection of Chlamydia trachomatis ocular infection in trachoma-endemic communities by rRNA amplification. *Investigative ophthalmology & visual science* 2009;50(1):90-94.

190. Nelson JC, Delucchi K, Schneider LS. Efficacy of second generation antidepressants in late-life depression: a meta-analysis of the evidence. *The American Journal of Geriatric Psychiatry* 2008;16(7):558-67.

191. Doody R, Stevens J, Beck C, et al. Practice parameter: Management of dementia (an evidence-based review) Report of the Quality Standards Subcommittee of the American Academy of Neurology. *Neurology* 2001;56(9):1154-66.

192. Eccles M, Clarke J, Livingston M, et al. North of England evidence based guidelines development project: guideline for the primary care management of dementia. *BMJ: British Medical Journal* 1998;317(7161):802.

193. National Collaborating Centre for Mental H. National Institute for Health and Clinical Excellence: Guidance. Dementia: A NICE-SCIE Guideline on Supporting People With Dementia and Their Carers in Health and Social Care. Leicester (UK): British Psychological Society

The British Psychological Society & The Royal College of Psychiatrists. 2007.

194. Orgeta V, Tabet N, Nilforooshan R, et al. Efficacy of Antidepressants for Depression in Alzheimer's Disease: Systematic Review and Meta-Analysis. *Journal of Alzheimer's disease : JAD* 2017;58(3):725-33. doi: 10.3233/jad-161247 [published Online First: 2017/05/17]

195. Bourouma R, Chevet D, Michel F, et al. Treatment of idiopathic retroperitoneal fibrosis with tamoxifen. *Nephrology, dialysis, transplantation: official publication of the European Dialysis and Transplant Association-European Renal Association* 1997;12(11):2407-10.

196. Clark C, Vanderpool D, Preskitt J. The response of retroperitoneal fibrosis to tamoxifen. *Surgery* 1991;109(4):502-06.

197. Dedeoglu F, Rose CD, Athreya BH, et al. Successful treatment of retroperitoneal fibrosis with tamoxifen in a child. *The Journal of rheumatology* 2001;28(7):1693-95.

198. Wallace SA, Roe B, Williams K, et al. Bladder training for urinary incontinence in adults. *The Cochrane Library* 2004

199. Abrams P, Andersson K-E, Birder L, et al. Fourth International Consultation on Incontinence Recommendations of the International Scientific Committee: Evaluation and treatment of urinary incontinence, pelvic organ prolapse, and fecal incontinence. *Neurourology and urodynamics* 2010;29(1):213-40.

200. Anderson CA, Omar MI, Campbell SE, et al. Conservative management for postprostatectomy urinary incontinence. *The Cochrane Library* 2015

201. Adams HP, Del Zoppo G, Alberts MJ, et al. Guidelines for the early management of adults with ischemic stroke. *Circulation* 2007;115(20):e478-e534.

202. Geeganage C, Bath P, Geeganage C, et al. Interventions for deliberately altering blood pressure in acute stroke. *Cochrane Database Syst Rev* 2008;4(4)

203. Schrader J, Lüders S, Kulschewski A, et al. The ACCESS study. *Stroke* 2003;34(7):1699-703.

204. CenterWatch. Atacand (candesartan cilexetil) 2018 [Available from: <http://www.centerwatch.com/drug-information/fda-approved-drugs/drug/646/atacand-candesartan-cilexetil> accessed 1/22/2018.

205. Bath PM, Krishnan K. Interventions for deliberately altering blood pressure in acute stroke. *The Cochrane Library* 2014

206. Wang H, Tang Y, Rong X, et al. Effects of early blood pressure lowering on early and long-term outcomes after acute stroke: an updated meta-analysis. *PloS one* 2014;9(5):e97917.

207. Dini C, Bianchi A. The potential role of vitamin D for prevention and treatment of tuberculosis and infectious diseases. *Annali dell'Istituto superiore di sanita* 2012;48(3):319-27.

208. Martineau AR, Honecker FU, Wilkinson RJ, et al. Vitamin D in the treatment of pulmonary tuberculosis. *The Journal of steroid biochemistry and molecular biology* 2007;103(3):793-98.

209. Gwinup G, Randazzo G, Elias A. The influence of vitamin D intake on serum calcium in tuberculosis. *Acta endocrinologica* 1981;97(1):114-17.

210. Morcos M, Gabr A, Samuel S, et al. Vitamin D administration to tuberculous children and its value. *Bollettino chimico farmaceutico* 1998;137(5):157-64.

211. Grobler L, Nagpal S, Sudarsanam TD, et al. Nutritional supplements for people being treated for active tuberculosis. *The Cochrane Library* 2016

| **#** | **Article and Author** | **Primary Medical Discipline** | **Date and Journal** | **Summary** | **Systematic Review** | **Search terms** |
| --- | --- | --- | --- | --- | --- | --- |
| 58 | Early versus delayed treatment of relapsed ovarian cancer (MRC OV05/EORTC 55955): a randomised trial Rustin et al. | Oncology | 10/2/2010  LANCET | The serum tumor marker CA125 is often used for initial diagnosis of ovarian cancer and for the monitoring of response to chemotherapy for epithelial ovarian cancer. CA125 often rises before the recurrence of ovarian cancer, and as such, guidelines recommend the testing for CA125 as part of follow-up care.^1^ The 1442 subjects in this trial were women with complete ovarian cancer remission after first-line platinum-based chemotherapy and a normal CA125 concentration. If the CA125 concentration exceeded twice the upper limit of normal (n=436), patients were randomly assigned to early (n=265) or delayed (n=264) chemotherapy. They found no difference in overall survival between the early (186 deaths) and delayed (184 deaths). This is a reversal of the use of CA125 as a tool for determining timing of chemotherapy in women with ovarian cancer who have been in remission. | 2016. Cochrane review. “Limited evidence from a single trial suggests that routine surveillance with CA125 in asymptomatic patients and treatment at CA125 relapse does not seem to offer survival advantage when compared to treatment at symptomatic relapse. RCTs are needed to compare different types of follow-up, looking at survival, QoL [quality of life], cost and psychological effects as outcomes.”^2^ | pubMed suggestion |
| 59 | Effect of palliative oxygen versus room air in relief of breathlessness in patients with refractory dyspnoea: a double-blind, randomised controlled trial Abernethy et al. | Pulmonary Disease | 9/4/2010  LANCET | Patients with various neurological conditions can sometimes become breathless.^3 4^ Oxygen therapy is often used to manage breathlessness. However, although there is accepted evidence for using oxygen in patients with COPD and hypoxemia,^5^ palliative oxygen is often used in patients with advanced life-limiting illness, irrespective of the partial pressure of oxygen in arterial blood (PaO2). This study randomized 239 subjects that had life-limiting illness, refractory dyspnea, and partial pressure of oxygen in arterial blood more than 7.3 kPa to either receive oxygen (n=120) or room air (n=119) via a concentrator through a nasal cannula at 2 L per minutes for 7 days. They found that oxygen delivered by cannula provides no additional symptomatic benefit for relief of refractory dyspnea. From baseline to day 6, mean morning breathlessness change by -0.9 points (95% CI: -1.3 to -0.5) in the oxygen group and -0.7 points (-1.2 to -0.2) in the room air group (p = 0.504). This is a reversal of the use of palliative oxygen as a way to relieve breathlessness in patients with refractory dyspnea. | 2016. Cochrane review. “Most evidence pertains to acute effects during exercise tests, and no evidence indicates that oxygen decreases breathlessness in the daily life setting. Findings show that oxygen does not affect health-related quality of life.”^6^ | pubMed suggestion |
| 60 | Misoprostol as an adjunct to standard uterotonics for treatment of post-partum haemorrhage: a multicentre, double-blind randomised trial Widmer et al. | Obstetrics and Gynecology | 5/22/2010  LANCET | Hemorrhage is the leading cause of maternal mortality in low-resource settings.^7^ Misoprostol is a prostaglandin E1 analogue that is widely marketed for the prevention and treatment of peptic ulcer disease. Although misoprostol is less effective than oxytocin for prevention of post-partum hemorrhage, it has been promoted widely because it is thermostable, orally administered, and inexpensitve.^8^ A trial on misoprostol administration in rural India found misoprostol is more effective than placebo in decreasing rates of post-partum hemorrhage, further supporting its use.^9^ This study randomized 1422 women with postpartum hemorrhage? to receive either 600 μg misoprostol (n=705) or matching placebo (n=717) sublingually. Both groups were also given routine injectable uterotonics. They found that the proportion of women in the misoprostol group who lost 500 mL of blood or more within 60 minutes was similar to the placebo group (100[14%] vs 100[14%], relative risk 1.02 [95% CI 0.79 – 1.32]). This is a reversal of the use of misoprostol as an adjunct to uterotonics for the treatment of post-partum hemorrhage. | 2014. Cochrane review. “Four RCTs (1881 participants) compared misoprostol with placebo given in addition to conventional uterotonics. Adjunctive use of misoprostol (in the dose of 600 to 1000 mcg) with simultaneous administration of additional uterotonics did not provide additional benefit for our primary outcomes including maternal mortality (risk ratio (RR) 6.16, 95% confidence interval (CI) 0.75 to 50.85), serious maternal morbidity (RR 0.34, 95% CI 0.01 to 8.31), admission to intensive care (RR 0.79, 95% CI 0.30 to 2.11) or hysterectomy (RR 0.93, 95% CI 0.16 to 5.41).  ^10^ | pubMed suggestion |
| 61 | Effect of vitamin A supplementation in women of reproductive age on maternal survival in Ghana (ObaapaVitA): a cluster-randomised, placebo-controlled trial Kirkwood et al | Obstetrics and Gynecology | 5/8/2010  LANCET | Vitamin A deficiency is a well-recognized nutritional problem and the leading cause of preventable childhood blindness. Vitamin A supplementation is safe (including during pregnancy), inexpensive, and potentially deliverable at community level, even in the absence of strong health systems advocated in existing safe motherhood strategies.^11^ This study randomized subjects in Ghana to receive a vitamin A supplements (25000 IU retinol equivalents) (544 clusters of 104 484 women) or placebo (542 clusters of 103 297 women). They found that women supplemented with vitamin A did not have significantly lower rates of pregnancy-related deaths than the placebo group (348 deaths per 100 000 pregnancies vs 377 per 100 000 pregnancies, adjusted odds ratio; 0.92 [95% CI 0.73-1.18], p=0.51). This is a reversal of vitamin A supplementation for reduction of pregnancy-related deaths in African countries. | 2015. Cochrane review. “The pooled results of three large trials in Nepal, Ghana and Bangladesh (with over 153,500 women) do not currently suggest a role for antenatal vitamin A supplementation to reduce maternal or perinatal mortality”^12^ | PubMed suggestion |
| 62 | Carotid artery stenting compared with endarterectomy in patients with symptomatic carotid stenosis (International Carotid Stenting Study): an interim analysis of a randomised controlled trial  Ederle et al. | Cardiovascular Disease | 3/20/2010  LANCET | Stenting for carotid artery stenosis gained popularity during the 1990s, and by 1999 stenting was used in almost 85% of percutaneous coronary intervention procedures.^13^ The potential benefit of endovascular treatment (angioplasty with or without stenting) as an alternative to carotid endarterectomy was first highlighted by the Carotid and Vertebral Artery Transluminal Angioplasty Study (CAVATAS).^14^ Two large randomized trials comparing use of carotid stenting with endarterectomy for symptomatic stenosis have subsequently published short-term outcomes and longer term results.^15^ This multicenter, international study randomized 1713 subjects with recent symptomatic carotid artery stenosis to receive either carotid artery stenting (n=855) or carotid endarterectomy (n=858). They found the stenting group had significantly higher incidence of stroke, death or procedural myocardial infarction (8.5% vs 5.2%, HR 1.69 [95% CI 1.16 – 2.45 p = 0.006]), risk of any stroke (HR 1.92 [1.27 – 2.89]), and all-cause death (HR 2.76 [1.16 – 6.56]). This is a reversal of the use of carotid artery stenting in patients with symptomatic carotid artery stenosis over carotid endarterectomy. | 2012. Cochrane review. “Endovascular treatment is associated with an increased risk of peri-procedural stroke or death compared with endarterectomy. However, this excess risk appears to be limited to older patients. The longer term efficacy of endovascular treatment and the risk of restenosis are unclear and require further follow-up of existing trials.”^16^  2017. Journal of American college of Cardiology “CAS [carotid artery stenting] and CEA [carotid artery endarterectomy] were associated with similar rates of a composite of periprocedural death, stroke, MI, or nonperiprocedural ipsilateral stroke. The risk of long-term overall stroke was significantly higher with CAS, and was mostly attributed to periprocedural minor stroke. CAS was associated with lower rates of periprocedural MI and cranial nerve palsy than CEA.”^17^ | PubMed suggestion |
| 63 | Comparative effectiveness of MRI in breast cancer (COMICE) trial: a randomised controlled trial  Turnbill et al. | Oncology | 2/13/2010  LANCET | Malignant lesions are difficult to detect in the mammographically dense breast. There is good evidence that MRIs detect malignancies, and may be better than X-ray mammography. Findings from two observational studies^18^ on the role of dynamic contrast-enhanced MRI in clinical management of patients scheduled for breast-conservation surgery have shown management to be altered in 14–18% of patients because of detection of disease that was more extensive than was first diagnosed, although neither study reported factors predictive of alteration in outcome. The COMICE study randomized 1623 women aged 18 years or older with biopsy-proven primary breast cancer who were scheduled for wide local excision after clinical, radiological (X-ray mammography and ultrasound), and pathological (fine-needle aspiration cytology or core biopsy) assessment. Patients were assigned to either MRI (n=816) or no further imaging (n=807). They found that addition of MRI to conventional triple assessment was not associated with a reduction of reoperation rate, with 153 (19%) needing reoperation in the MRI group and 156 (19%) in the no MRI group (Odds ratio 0.96 [95% CI 0.75-1.24], p=0.77). This is a reversal of the use of MRI after triple assessment in women with breast cancer. | 2013. “Our summary of the evidence showed that MRI significantly increased mastectomy rates and suggests an unfavorable harm-benefit ratio for routine use of preoperative MRI in BC. We found weak evidence that MRI reduced re-excision surgery in patients with [Invasive lobular cancer] ILC -although this was at the expense of increased mastectomies-and overall patient benefit from MRI in ILC is not clear from this study.”^19^ | PubMed suggestion |
| 64 | Umbilical vein oxytocin for the treatment of retained placenta (Release Study): a double-blind, randomised controlled trial Weeks et al. | Obstetrics and Gynecology | 1/9/2010  LANCET | Retained placenta complicates 0.1 – 2% of deliveries. The rate has increased in Europe since the 1920’s, and is nearly ten times that of resource-poor settings. Oxytocin injection into the umbilical cord began as early as the 1980s to treat retained placenta^20^ an alternative to manual removal, which is invasive and carries risk of infection and trauma.^21^ This study randomizes 577 hemodynamically stable women with a retained placenta for more than 30 minutes to receive 30 mL saline containing either 50 IU oxytocin (n=292) or 5 mL water (n=285). This study found that umbilical oxytocin has no clinically significant effect on the need for manual removal for women with retained placenta (oxytocin 179/292 [61.3%] vs placebo 177/285 [62.1%]). This is a reversal of the use of umbilical vein oxytocin for treatment of retained placenta on hemodynamically stable women. | 2012. Cochrane review. “… high-quality randomized trials show that the use of oxytocin has little or no effects [on reduction in manual removal of the placenta].”^22^ | PubMed suggestion |
| 65 | Chlorhexidine maternal  vaginal and neonate body wipes in sepsis and vertical  transmission of pathogenic  bacteria in South Africa: a  randomised, controlled trial Cutland et al. | Obstetrics and Gynecology | 12/5/2009  LANCET | Chlorhexidine wipes have been developed to help prevent the spread of bacterial infections from mother to newborn baby. Some countries in Europe have already implemented the practice of vaginal disinfection with these wipes as preventive measures.^23^ Neonatal and maternal mortality and morbidity due to bacterial infections are high in places like Africa, and some countries, like Malawi, have tested this type of intervention.^24^ In this study 8011 women aged 12-51 years were randomly assigned to chlorhexidine vaginal wipes (n=4072) or external genitalia water wipes (4057) during active labor. They showed that there was no difference between chlorhexidine and control groups in preventing neonatal sepsis or vertical acquisition of potentially pathogenic bacteria (chlorhexidine 141 [3%] of 4072 *vs* control 148 [4%] of 4057; p=0.6518). This is a reversal of the practice of using chlorhexidine vaginal wipes to reduce neonatal sepsis and vertical acquisition of potentially pathogenic bacteria in neonates. | 2015. Cochrane review. “Maternal vaginal chlorhexidine compared to usual care probably leads to no difference in neonatal mortality in hospital settings. Maternal vaginal chlorhexidine compared to usual care results in no difference in the risk of infections in hospital settings.”^25^ | PubMed suggestion |
| 66 | Comparison of routine and on-demand prescription of chest radiographs in mechanically ventilated adults: a multicentre, cluster-randomised, two-period crossover study Hejblum et al. | Critical Care Medicine | 11/14/2009  LANCET | The American College of Radiology recommends routine chest radiographs for patients who are mechanically ventilated, in part because this can help diagnose life-threatening situations.^26^ However, because of the extra radiation and often unnecessary expense, the practice of routine chest radiographs is questioned.^26^ A survey of physicians showed that 63% of physicians used a daily-routine strategy for chest radiographs.^27^ This RCT randomly assigned 21 ICUs at 18 hospitals in France to use a routine (n=424) or on demand (n=425) strategy. They showed that an on-demand strategy reduced the number of chest radiographs(32% [95% CI 25 – 38]) without significant differences in mortality, length of hospital stay, or days of mechanical ventilation compared to routine daily chest radiographs. This is a reversal of the routine daily use of chest radiographs in mechanically ventilated patients. | 2012. “This meta-analysis did not detect any harm associated with a restrictive chest radiograph strategy.”^28^ Not all studies included in the review were randomized. | PubMed suggestion |
| 67 | Effect of interferon gamma-1b on survival in patients with idiopathic pulmonary fibrosis (INSPIRE): a multicentre, randomised, placebo-controlled trial King et al | Pulmonary Disease | 7/18/2009 | Idiopathic pulmonary fibrosis (IPF) is a disease where there is fibroblast proliferation and excess extracellular connective tissue matrix protein, which leads to deterioration of lung function.^29^ Treatment options are poor, as the few that are recommended show little to moderate benefit. Interferon gamma-1b has been shown to reduce fibroblast proliferation and extracellular matrix deposition, and these findings led to the thought that it might also be an effective treatment option for patients with IPF.^29^ These findings, combined with poor treatment options led to the use of interferon gamma1-b before randomized trials could be conducted to test the effectiveness. | 2010. Cochrane review. “From the studies in this review, interferon gamma‐1beta has not been shown to affect survival.” |  |
| 68 | Effectiveness of thigh-length graduated compression stockings to reduce the risk of deep vein thrombosis after stroke (CLOTS trial 1): a multicentre, randomised controlled trial CLOTS Trials Collaboration, Dennis et al. | Cardiovascular Disease | 6/9/2009  LANCET | Compression therapy was first used by German physicians in the late 19^th^ century when they noticed that superficial vein thromboses disappeared after the use of compression bandages.^30^ Compression stockings were used as early as the 1930s but became widely used after the results of a trial were published in 2000.^30^ National stroke guidelines recommend use of graduated compression stockings (GCS) to reduce risk of deep vein thrombosis (DVT) and pulmonary embolism^31^ although there is a lack of clinical trials investigating its use in an acute stroke population. This study compared routine care plus GCS (n=1265) with routine care plus avoidance of GCS (n=1262) in patients within 1 week of an acute stroke. The study found that there was no difference in occurrence of symptomatic or asymptomatic DVT between groups (126 [10%] in the GCS group vs 133 [10.5%] in the control group) and more adverse events (64 [5%] vs 16 [1%]) in the GCS group. This is a reversal of the use of thigh-length graduated compression stockings to reduce the risk of deep vein thrombosis after stroke. | Cochrane. 2010. “Evidence from randomised trials does not support the routine use of GCS to reduce the risk of DVT after acute stroke.”^32^ However, this RCT was not included in the review. | PubMed suggestion |
| 69 | Warfarin thromboprophylaxis in cancer patients with central venous catheters (WARP): an open-label randomised trial Young et al. | Oncology | 2/14/2009  LANCET | The use of long-term central venous catheters (CVC) for infusional chemotherapy is common in patients with cancer. However, their use has been associated with upper-limb deep-vein thrombosis (DVT). Earlier clinical trials found that patients treated with CVC chemotherapy benefited from prophylactic anticoagulation, but later trials have shown contradictory findings.^33^ A majority of clinicians in the UK have been found to administer warfarin for thromboprophylaxis in their patients receiving CVC chemotherapy, even with the uncertainty of its efficacy.^34 35^ This study compared the effect of warfarin (n=408) vs no warfarin (n=404) on rate of radiologically proven, symptomatic catheter-related thrombosis and found no significant difference in symptomatic catheter-related or other thromboses in patients with cancer. Compared with no warfarin, warfarin did not reduce the rate of catheter related thromboses (24 [6%] vs 24 [6%], relative risk 0.99, [95% CI 0.57 – 1.72], p=0.98). This is a reversal of the use of warfarin to reduce catheter related thromboses. | 2014. Cochrane review. “Compared with no anticoagulation, we found a statistically significant reduction of symptomatic DVT with heparin and asymptomatic DVT with VKA. Heparin was associated with a higher risk of thrombocytopenia and asymptomatic DVT when compared with VKA. However, the findings did not rule out other clinically important benefits and harms. People with cancer with CVCs considering anticoagulation should balance the possible benefit of reduced thromboembolic complications with the possible harms and burden of anticoagulants.”^36^ | pubMed suggestion |
| 70 | Efficacy of systematic pelvic lymphadenectomy in endometrial cancer (MRC ASTEC trial): a randomised study  Kitchener et al. | Oncology | 1/10/2009  LANCET | Endometrial cancer is a common gynecological cancer that is generally treated through hysterectomy and bilateral salpingo-oophorectomy (BSO) for stage I tumors. Some patients with stage 1 endometrial cancer are still at risk of metastasis after surgery, and adjuvant radiotherapy is typically added to treatment in high risk women.^37^ To prevent unnecessary adjuvant radiotherapy, guidelines have implemented systemic pelvic and para-aortic lymphadenectomy to properly stage the endometrial cancer.^38^ Case series and non-randomized studies have shown an association between lymphadenectomy and increased survival,^39 40^ but other observational studies have not found benefit to the procedure.^41^ From 85 centers in four countries, 1408 women with histologically proven endometrial carcinoma thought preoperatively to be confined to the corpus were randomized to either standard surgery (n=704) or standard surgery plus lymphadenectomy (n=704). This study found that the addition of lymphadenectomy to standard therapy did not affect overall or recurrence-free survival in women with early endometrial cancer (88 deaths in the standard surgery group vs 103 in lymphadenectomy, HR: 1.16 [95% CI: 0.87 – 1.54; p = 0.31]). This is a reversal of the guidelines recommending lymphadenectomy to properly stage endometrial cancer. | 2017. Cochrane review. “This review found no evidence that lymphadenectomy decreases risk of death or disease recurrence compared with no lymphadenectomy in women with presumed stage I disease.”^42^ | pubMed suggestion |
| 71 | Cardiovascular events associated with rofecoxib: final analysis of the APPROVe trial Baron et al. | Cardiovascular Disease | 11/15/2008  LANCET | Rofecoxib was approved by the FDA in 1999 to treat osteoarthritis, acute pain, and dysmenorrhea.^43^ Because of the anti-inflammatory effect, rofecoxib was also thought to prevent colorectal polyps and cancer. By 2004, 80 million patients had taken this drug, and annual sales were more than $2.5 billion.^43^ The APPROVe study randomized 2587 patients with a history of colorectal adenomas to either 25 mg rofecoxib (n=1287) or placebo (n=1300). They assessed the effects of rofecoxib on recurrence of adenomatous polyps in the large bowel and found that rofecoxib was associated with an increased combined incidence of non-fatal myocardial infarction, non-fatal stroke, and death from cardiovascular, hemorrhagic, and unknown causes (59 vs 34; hazard ratio 1.79 [95% CI 1.17 – 2.73], p=0.006). The trial was terminated early on recommendation of its data safety and monitoring board due to concerns about cardiovascular toxicity. This is a reversal of the use of rofecoxib to prevent colorectal polyps and cancer due to harms. | 2011. “Compared with placebo, rofecoxib was associated with the highest risk of myocardial infarction.”^44^ | GoogleScholar - cited this RCT |
| 72 | Effect of rosuvastatin in patients with chronic heart failure (the GISSI-HF trial): a randomised, double-blind, placebo-controlled trial Tavazzi et al. | Cardiovascular Disease | 10/4/2008  LANCET | Statins are known to be effective for atherothrombosis prevention, but are also under investigation for other indications such as their potential anti-inflammatory, antihypertrophic, antifibrotic, and antioxidant effects.^45^ Large observational studies and meta-analyses have indicated that statins can contribute to lowered cardiovascular mortality.^45^ Patients aged 18 years or older with chronic heart failure of New York Association class II-IV, irrespective of cause and left ventricular ejection fraction were randomized to rosuvastatin (10 mg daily, n=2285) or placebo (n=2289). This study found that rosuvastatin had no effect on time to death or admission to hospital for cardiovascular reasons (1305 [57%]) compared to placebo (1283 [56%]; adjusted HR 1.01 [99% CI 0.908 – 1.112], p=0.903). This is a reversal of the use of statins for lowered cardiovascular mortality in patients with chronic heart failure. | 2010. National Clinical Guideline for Diagnosis and Management in Primary and Secondary Care. “These trials (GISSI-HF and CORONA) randomized 4574 patients with heart failure and 5011 patients over the age of 60 years with systolic heart failure of ischaemic origin, respectively, to have 10 mg rosuvastatin or placebo. The statin did not have an impact on any of the trials’ outcomes other than reducing hospitalisation in the CORONA study. Therefore, it is unlikely that statins would be beneficial in heart failure. The Guideline Development Group (GDG) felt that in the light of this evidence, the recommendation on statin use from the 2003 guideline should be deleted.” | PubMed suggestion |
| 73 | Management of asthma based on exhaled nitric oxide in addition to guideline-based treatment for inner-city adolescents and young adults: a randomised controlled trial Szefler et al. | Pulmonary Disease | 9/20/2008  LANCET | International guidelines recommend a range of clinical tests to confirm the diagnosis and manage symptoms of asthma. Included in this is exhaled nitric oxide.^46^ This study randomized 546 patients who adhered to treatment during this run-in period were then randomly assigned to 46 weeks of either standard treatment, based on guidelines of the National Asthma Education and Prevention Program, or standard treatment modified on the basis of measurements of fraction of exhaled NO. They found that conventional asthma management resulted in good control of symptoms, while the addition of exhaled nitric oxide resulted in higher doses of inhaled corticosteroids without any clinically important improvements in asthma symptoms or management (difference in mean number of days with asthma symptoms between groups; 0.04 [95% CI -0.22 to 0.29], p=0.78) in patients with persistent asthma. This is a reversal of the universal management of asthma based on exhaled nitric oxide. | 2016. Cochrane review. “Therefore, the use of FeNO to guide asthma therapy in children may be beneficial in a subset of children (had at least one exacerbation during the study period), it cannot be universally recommended for all children with asthma.”^47^ | PubMed suggestion |
| 74 | Active symptom control with or without chemotherapy in the treatment of patients with malignant pleural mesothelioma (MS01): a multicentre randomised trial Muers et al. | Oncology | 5/17/2008  LANCET | Treatment recommendations for malignant mesothelioma have been primarily through active symptom control (ASC)^48^ and because chemotherapy therapies do not appear to provide benefit for patients with mesothelioma. Despite this, chemotherapy has been used,^49^ although no specific regimen appears to be more favorable than others. 409 patients with malignant pleural mesothelioma were randomly assigned to ASC alone (n=136), to ASC plus MVP (four cycles of mitomycin 6 mg/m^2^, vinblastine 6 mg/m^2^, and cisplatin 50 mg/m^2^ every 3 weeks, n=137), of ASC plus vinorelbine (one injection of vinorelbine 30 mg/m^2^ every week for 12 weeks, n=136). This study found that the addition of chemotherapy to ACS did not increase overall survival (393 [96%] deaths overall, ASC 132 [97%], ASC plus MVP 132 [96%], ASC plus vinorelbine 129 [95%]) or quality of life compared to ACS alone in patients with malignant pleural mesothelioma. This is a reversal of the addition of chemotherapy, specifically MVP and vinblastine, to ASC in patients with MS01. Since the publication of this trial, there have been positive RCTs on this condition. This is a reversal of the practice at the time of this study. | None found | active symptom control chemotherapy in malignant pleural mesothelioma |
| 75 | Multiple-dose activated charcoal in acute self-poisoning: a randomised controlled trial Eddleston et al. | Public Health and General Preventive Medicine | 2/16/2008  LANCET | In rural developing countries, self-poisoning is often a result of ingesting toxic pesticides and plants rather than pharmaceuticals that are more often used in the developed world. Activated charcoal is a widely used substance to treat self-poisoning in some parts of the world.^50^ 4632 patients were randomized to receive no charcoal (n=1554), one dose of charcoal (n=1545), or six doses of charcoal (n=1533). This study found that activated charcoal did not reduce risk of mortality (97 [6.3%]) compared to no charcoal (105 [6.8%]) in patients experiencing self-poisoning in 3 Sri Lankan hospitals. This is a reversal of the use of activated charcoal in cases of self-poisoning in developing countries. | None found | activated charcoal and poisoning |
| 76 | Risperidone, haloperidol, and placebo in the treatment of aggressive challenging behaviour in patients with intellectual disability: a randomised controlled trial Tyrer et al. | Psychiatry | 1/5/2008  LANCET | Adults with intellectual disability can often times express aggressive, challenging behavior. Antipsychotic drugs are often used in this population, even if the patient with intellectual disability does not have any underlying psychiatric illness.^51^ The NACHBID study randomized 86 non-psychotic patients presenting with aggressive challenging behavior from ten centers in England, Wales, and one in Australia to either haloperidol (n=28), risperidone (n=29) or placebo (n=29). They found that haloperidol, risperidone, and placebo all decreased aggression after 4 weeks in non-psychotic patients presenting with aggressive behavior, with placebo showing the greatest change (median decrease in MOAS score after 4 weeks=9 [95% CI 5–14] for placebo, 79% from baseline; 7 [4–14] for risperidone, 58% from baseline; 6.5 [5–14] for haloperidol, 65% from baseline; p=0·06). This is a reversal of the administration of risperidone or haloperidol to patients with intellectual disabilities expressing aggressive challenging behavior who did not have any underlying psychiatric illness. | A 2016 review concluded that there was insufficient evidence to support the use of psychotherapy, biological, or system level interventions for adults with mild to moderate intellectual disabilities. This review did not include the RCT in the in its analysis.^52^ | GoogleScholar-cited RCT |
| 77 | Mechanical bowel preparation for elective colorectal surgery: a multicentre randomised trial Contant et al. | Surgery | 12/22/2007  LANCET | Preoperative bowel preparation has been used since the 1950s to reduce intestinal mass, decrease risk of infection from pathogens in the colon, and improve post-surgical healing.^53^ This procedure was commonly used at the beginning of this study.^53^ 1431 patients who were going to have elective colorectal surgery were randomly assigned to either mechanical bowel preparation (n=670) or not (n=684). This study found that mechanical bowel preparation did not lower the rate of anastomotic leakage compared to no preparation (difference 0.6%, 95% CI −1.7% to 2.9%, p=0.69). This is a reversal of the use of mechanical bowel preparation for elective colorectal surgery. | 2011. Cochrane review. “Despite the inclusion of more studies with a total of 5805 participants, there is no statistically significant evidence that patients benefit from mechanical bowel preparation, nor the use of rectal enemas. In colonic surgery the bowel cleansing can be safely omitted and induces no lower complication rate.”^54^ | PubMed suggestion |
| 78 | Assessment of diclofenac or spinal manipulative therapy, or both, in addition to recommended first-line treatment for acute low back pain: a randomised controlled trial Hancook et al. | Orthopedic | 11/10/2007  LANCET | Current guidelines for first line treatment for acute lower back pain recommend physicians give advice (remain active, avoid bed rest, and reassurance of favorable prognosis) and paracetamol. Non-steroidal anti-inflammatory drugs (NSAIDs) and spinal manipulative therapy are recommended as second-line management options for patients who have slow recovery.^55^ 240 patients with acute low back pain who had seen their general practitioner and had been given advice and paracetamol were randomized to one of four groups: diclofenac 50 mg twice daily and placebo manipulative therapy (n=60); diclofenac 50 mg twice daily and spinal manipulative therapy (n=60); spinal manipulative therapy and placebo drug (n=60); or double placebo (n=60). This study found that patients do not recover more quickly with the addition of either diclofenac or spinal manipulative therapy (spinal manipulative therapy hazard ratio 1.01, [95% CI 0.77–1.31], p=0.955). This is a double reversal of the use of spinal manipulative therapy and diclofenac for treatment of acute lower back pain. | A 2017 SR/MA found spinal manipulation to be beneficial for patients with acute low back pain, but not when comparing spinal manipulation to sham controls.^56^ |  |
| 79 | Effect of daily zinc supplementation on child mortality in southern Nepal: a community-based, cluster randomised, placebo-controlled trial Tielsch et al. | Public Health and General Preventive Medicine | 10/6/2007  LANCET | Zinc is a micronutrient that may lower the risk of morbidity and mortality when provided to populations that are deficient. Several methods have been used to increase the availability and intake of zinc in these populations, including supplementation and fortification.^57^ A community based, cluster-randomized trial randomized 41276 children aged 1-35 months into a zinc group (n=20968) or placebo (n=20308). They found no reduction in mortality between the two groups (Hazard ratio 0.92 [95% CI 0.75 – 1.12]). This is a reversal of the supplementation the individual nutrient of zinc in developing countries to reduce mortality. | 2014. Cochrane.  This review found that there was a non-significant reduction in risk of death with zinc supplementation (RR 0.95 [95% CI 0.86 – 10.5]), and a small increase in average height in the zinc group. They concede that a “size of this effect might not be clinically important”, however.^58^ | PubMed suggestion |
| 80 | Effectiveness of an early supplementation scheme of high-dose vitamin A versus standard WHO protocol in Gambian mothers and infants: a randomised controlled trial Darboe et al. | Public Health and General Preventive Medicine | 6/23/2007  LANCET | Many developing countries have adopted the WHO recommended dose for vitamin A supplementation.^59^ The Vitamin A Consultative Group recommended a doubling of vitamin A recommendations for developing countries, out of concern for low serum concentrations of retinol in populations living in these countries. ^60^ 197 infants were randomized to either high dose (n=99) or the WHO recommended dose (n=98) and followed for 12 months. This study determined that a high-dose regimen of vitamin A supplementation for mother-infant pairs in rural Gambia did not affect levels of infant plasma vitamin A, and rates of H pylori infection, pneumococcal carriage, and gut epithelial integrity compared to the WHO recommended dose. This is a reversal of vitamin A supplementation at levels recommended by the Vitamin A Consultative Group, which are higher than World Health Organization, in developing countries. | 2016. Cochrane review. “There was no evidence of benefit from different doses of vitamin A supplementation for postpartum women on maternal and infant mortality and morbidity, compared with other doses or placebo.”^61^ | PubMed suggestion |
| 81 | Effect of zinc supplementation on mortality in children aged 1–48 months: a community-based randomised placebo-controlled trial Sazawal  et al. | Public Health and General Preventive Medicine | 3/17/2007  LANCET | Zinc is a micronutrient that may lower the risk of morbidity and mortality when provided to populations that are deficient, and cross-sectional studies have shown an inverse association between zinc status and certain types of malaria.^62^ Several methods have been used to increase the availability and intake of zinc in these populations, including supplementation and fortification.^57^ This study compared daily supplementation with zinc (10 mg, 5 mg in children younger than 12 months; n=21274) compared to placebo (n=21272) in children aged 1-36 months residing in an area with a high-frequency of malaria. They found no significant reduction in overall mortality (7% difference [95% CI -6% - 19%], p=0.29) between the group supplemented with zinc and placebo. This is a reversal of zinc supplementation in areas with high incidence of malaria to reduce mortality. | 2014. Cochrane.  This review found that there was a non-significant reduction in risk of death with zinc supplementation (RR 0.95 [95% CI 0.86 – 10.5]), and a small increase in average height in the zinc group. They concede that a “size of this effect might not be clinically important”, however.^58^ | PubMed suggestion |

1. Pepin K, del Carmen M, Brown A, et al. CA 125 and epithelial ovarian cancer: role in screening, diagnosis, and surveillance. *American Journal of Hematology/Oncology®* 2014;10(6)

2. Kew F, Galaal K, Bryant A, et al. Evaluation of follow-up strategies for patients with epithelial ovarian cancer following completion of primary treatment. *Cochrane Database Syst Rev* 2011;6

3. Higginson IJ, Hart S, Silber E, et al. Symptom prevalence and severity in people severely affected by multiple sclerosis. *Journal of palliative care* 2006;22(3):158.

4. Saleem T, Leigh PN, Higginson IJ. Symptom prevalence among people affected by advanced and progressive neurological conditions-a systematic review. *Journal of palliative care* 2007;23(4):291.

5. Wilt TJ, Niewoehner D, MacDonald R, et al. Management of stable chronic obstructive pulmonary disease: a systematic review for a clinical practice guideline. *Annals of internal medicine* 2007;147(9):639-53.

6. Ekström M, Ahmadi Z, Bornefalk‐Hermansson A, et al. Oxygen for breathlessness in patients with chronic obstructive pulmonary disease who do not qualify for home oxygen therapy. *The Cochrane Library* 2016

7. Khan KS, Wojdyla D, Say L, et al. WHO analysis of causes of maternal death: a systematic review. *Lancet* 2006;367(9516):1066-74. doi: 10.1016/s0140-6736(06)68397-9 [published Online First: 2006/04/04]

8. Prata N, Sreenivas A, Vahidnia F, et al. Saving maternal lives in resource-poor settings: facing reality. *Health policy* 2009;89(2):131-48.

9. Derman RJ, Kodkany BS, Goudar SS, et al. Oral misoprostol in preventing postpartum haemorrhage in resource-poor communities: a randomised controlled trial. *The Lancet* 2006;368(9543):1248-53. doi: <https://doi.org/10.1016/S0140-6736(06)69522-6>

10. Mousa HA, Alfirevic Z. Treatment for primary postpartum haemorrhage. *Cochrane database syst Rev* 2003;1

11. Starrs AM. Delivering for women. *The Lancet* 2007;370(9595):1285-87.

12. Van Den Broek N, Dou L, Othman M, et al. Vitamin A supplementation during pregnancy for maternal and newborn outcomes. *Cochrane Database of Systematic Reviews* 2010(11)

13. Newsome LT, Kutcher MA, Gandhi SK, et al. A protocol for the perioperative management of patients with intracoronary drug-eluting stents. *Anesthesia Patient Safety Foundation APSF Newsletter* 2006;4:81-2.

14. Brown M, Rogers J, Bland J. Endovascular versus surgical treatment in patients with carotid stenosis in the Carotid and Vertebral Artery Transluminal Angioplasty Study (CAVATAS): a randomised trial. *The Lancet* 2001;357(9270):1729-37.

15. Mas J-L, Chatellier G, Beyssen B, et al. Endarterectomy versus stenting in patients with symptomatic severe carotid stenosis. *New England Journal of Medicine* 2006;355(16):1660-71.

16. Bonati LH, Lyrer P, Ederle J, et al. Percutaneous transluminal balloon angioplasty and stenting for carotid artery stenosis. *The Cochrane Library* 2012

17. Sardar P, Chatterjee S, Aronow HD, et al. Carotid artery stenting versus endarterectomy for stroke prevention: a meta-analysis of clinical trials. *Journal of the American College of Cardiology* 2017;69(18):2266-75.

18. Fischer U, Kopka L, Grabbe E. Breast carcinoma: effect of preoperative contrast-enhanced MR imaging on the therapeutic approach. *Radiology* 1999;213(3):881-88.

19. Houssami N, Turner R, Morrow M. Preoperative magnetic resonance imaging in breast cancer: meta-analysis of surgical outcomes. *Annals of surgery* 2013;257(2):249-55.

20. Carroli G, Bergel E. Umbilical vein injection for management of retained placenta. *Cochrane Database of Systematic Reviews* 2001(4) doi: 10.1002/14651858.CD001337

21. Sivalingam N, Surinder S. Is there a place for intra-umbilical oxytocin for the management of retained placenta? *The Medical journal of Malaysia* 2001;56(4):451-9. [published Online First: 2002/05/17]

22. Nardin JM, Weeks A, Carroli G. Umbilical vein injection for management of retained placenta. *The Cochrane Library* 2011

23. Schrag SJ, Schuchat A. Easing the burden: characterizing the disease burden of neonatal group B streptococcal disease to motivate prevention: The University of Chicago Press, 2004.

24. Taha TE, Biggar RJ, Broadhead RL, et al. Effect of cleansing the birth canal with antiseptic solution on maternal and newborn morbidity and mortality in Malawi: clinical trial. *Bmj* 1997;315(7102):216-20.

25. Sinha A, Sazawal S, Pradhan A, et al. Chlorhexidine skin or cord care for prevention of mortality and infections in neonates. *The Cochrane Library* 2015

26. Hejblum G, Chalumeau-Lemoine L, Ioos V, et al. Comparison of routine and on-demand prescription of chest radiographs in mechanically ventilated adults: a multicentre, cluster-randomised, two-period crossover study. *The Lancet* 2009;374(9702):1687-93.

27. Graat ME, Hendrikse KA, Spronk PE, et al. Chest radiography practice in critically ill patients: a postal survey in the Netherlands. *BMC medical imaging* 2006;6(1):8.

28. Ganapathy A, Adhikari NK, Spiegelman J, et al. Routine chest x-rays in intensive care units: a systematic review and meta-analysis. *Critical Care* 2012;16(2):R68.

29. Interferon gamma-1b therapy for advanced idiopathic pulmonary fibrosis. Mayo Clinic proceedings; 2003. Elsevier.

30. Galanaud JP, Laroche JP, Righini M. The history and historical treatments of deep vein thrombosis. *Journal of Thrombosis and Haemostasis* 2013;11(3):402-11.

31. Adams HP, Jr., del Zoppo G, Alberts MJ, et al. Guidelines for the early management of adults with ischemic stroke: a guideline from the American Heart Association/American Stroke Association Stroke Council, Clinical Cardiology Council, Cardiovascular Radiology and Intervention Council, and the Atherosclerotic Peripheral Vascular Disease and Quality of Care Outcomes in Research Interdisciplinary Working Groups: The American Academy of Neurology affirms the value of this guideline as an educational tool for neurologists. *Circulation* 2007;115(20):e478-534. doi: 10.1161/circulationaha.107.181486 [published Online First: 2007/05/23]

32. Naccarato M, Chiodo Grandi F, Dennis M, et al. Physical methods for preventing deep vein thrombosis in stroke. *Cochrane Database Syst Rev* 2010(8):Cd001922. doi: 10.1002/14651858.CD001922.pub3 [published Online First: 2010/08/06]

33. Couban S, Goodyear M, Burnell M, et al. Randomized placebo-controlled study of low-dose warfarin for the prevention of central venous catheter-associated thrombosis in patients with cancer. *Journal of clinical oncology : official journal of the American Society of Clinical Oncology* 2005;23(18):4063-9. doi: 10.1200/jco.2005.10.192 [published Online First: 2005/03/16]

34. Monreal M, Alastrue A, Rull M, et al. Upper extremity deep venous thrombosis in cancer patients with venous access devices--prophylaxis with a low molecular weight heparin (Fragmin). *Thrombosis and haemostasis* 1996;75(2):251-53.

35. Bern MM, Lokich JJ, Wallach SR, et al. Very low doses of warfarin can prevent thrombosis in central venous catheters: a randomized prospective trial. *Annals of internal medicine* 1990;112(6):423-28.

36. Akl EA, Ramly EP, Kahale LA, et al. Anticoagulation for people with cancer and central venous catheters. *The Cochrane Library* 2014

37. Kong A, Simera I, Collingwood M, et al. Adjuvant radiotherapy for stage I endometrial cancer: systematic review and meta-analysis. *Annals of Oncology* 2007;18(10):1595-604. doi: 10.1093/annonc/mdm066

38. Shepherd JH. Revised FIGO staging for gynaecological cancer. *BJOG: An International Journal of Obstetrics & Gynaecology* 1989;96(8):889-92.

39. Creasman WT, Morrow CP, Bundy BN, et al. Surgical pathologic spread patterns of endometrial cancer: A gynecologic oncology group study. *Cancer* 1987;60(S8):2035-41. doi: doi:10.1002/1097-0142(19901015)60:8+<2035::AID-CNCR2820601515>3.0.CO;2-8

40. Morrow CP, Bundy BN, Kurman RJ, et al. Relationship between surgical-pathological risk factors and outcome in clinical stage I and II carcinoma of the endometrium: a Gynecologic Oncology Group study. *Gynecol Oncol* 1991;40(1):55-65. [published Online First: 1991/01/01]

41. Trimble EL, Kosary C, Park RC. Lymph node sampling and survival in endometrial cancer. *Gynecol Oncol* 1998;71(3):340-3. doi: 10.1006/gyno.1998.5254 [published Online First: 1999/01/15]

42. Frost JA, Webster KE, Bryant A, et al. Lymphadenectomy for the management of endometrial cancer. *Cochrane Database Syst Rev* 2017;10:Cd007585. doi: 10.1002/14651858.CD007585.pub4 [published Online First: 2017/10/03]

43. Topol EJ. Failing the public health—rofecoxib, Merck, and the FDA. *New England Journal of Medicine* 2004;351(17):1707-09.

44. Trelle S, Reichenbach S, Wandel S, et al. Cardiovascular safety of non-steroidal anti-inflammatory drugs: network meta-analysis. *Bmj* 2011;342:c7086.

45. Levy WC. Observational studies of statins in systolic heart failure. *Heart failure clinics* 2008;4(2):201-08.

46. Global initiative for asthma: global strategy for asthma management and prevention. NHLBI/WHO workshop report NIH publication; 1995.

47. Petsky HL, Kew KM, Kynaston JA, et al. Exhaled nitric oxide levels to guide treatment for children with asthma. *Cochrane Database of Systematic Reviews* 2015

48. Committee BTSSoC. Statement on malignant mesothelioma in the United Kingdom. *Thorax* 2001;56(4):250-65.

49. Fizazi K, Caliandro R, Soulie P, et al. Combination raltitrexed (Tomudex®)–oxaliplatin: a step forward in the struggle against mesothelioma? The Institut Gustave Roussy experience with chemotherapy and chemo-immunotherapy in mesothelioma. *European Journal of Cancer* 2000;36(12):1514-21.

50. Ardagh M, Flood D, Tait C. Limiting the use of gastrointestinal decontamination does not worsen the outcome from deliberate self-poisoning. *The New Zealand Medical Journal* 2001;114(1140):423-25.

51. Branford D. A study of the prescribing for people with learning disabilities living in the community and in National Health Service care. *Journal of Intellectual Disability Research* 1994;38(6):577-86.

52. Koslowski N, Klein K, Arnold K, et al. Effectiveness of interventions for adults with mild to moderate intellectual disabilities and mental health problems: systematic review and meta-analysis. *The British Journal of Psychiatry* 2016:bjp. bp. 114.162313.

53. Oliveira L, Wexner SD, Daniel N, et al. Mechanical bowel preparation for elective colorectal surgery. *Diseases of the colon & rectum* 1997;40(5):585-91.

54. Guenaga K, Matos D, Wille-Jorgensen P. Mechanical bowel preparation for elective colorectal surgery. *Cochrane Database Syst Rev* 2009;1(1)

55. van Tulder M, Becker A, Bekkering T, et al. Chapter 3. European guidelines for the management of acute nonspecific low back pain in primary care. *European spine journal : official publication of the European Spine Society, the European Spinal Deformity Society, and the European Section of the Cervical Spine Research Society* 2006;15 Suppl 2:S169-91. doi: 10.1007/s00586-006-1071-2 [published Online First: 2006/03/22]

56. Paige NM, Miake-Lye IM, Booth MS, et al. Association of Spinal Manipulative Therapy With Clinical Benefit and Harm for Acute Low Back Pain: Systematic Review and Meta-analysis. *Jama* 2017;317(14):1451-60. doi: 10.1001/jama.2017.3086 [published Online First: 2017/04/12]

57. Gibson RS, Ferguson EL. Nutrition intervention strategies to combat zinc deficiency in developing countries. *Nutrition Research Reviews* 1998;11(1):115-31.

58. Mayo‐Wilson E, Junior JA, Imdad A, et al. Zinc supplementation for preventing mortality, morbidity, and growth failure in children aged 6 months to 12 years of age. *The Cochrane Library* 2014

59. Force WUIT, Group IVAC, UNICEF., et al. Vitamin A supplements: a guide to their use in the treatment and prevention of vitamin A deficiency and xerophthalmia: World Health Organization 1997.

60. Sommer A, Davidson FR. Assessment and control of vitamin A deficiency: the Annecy Accords. *The Journal of nutrition* 2002;132(9):2845S-50S.

61. Oliveira JM, Allert R, East CE. Vitamin A supplementation for postpartum women. *The Cochrane Library* 2016

62. Shankar AH. Nutritional modulation of malaria morbidity and mortality. *The Journal of infectious diseases* 2000;182(Supplement_1):S37-S53.

| **#** | **Article and Author** | **Primary Medical Discipline** | **Date and Journal** | **Summary** | **Systematic Review** | **Systematic Review Search Terms** |
| --- | --- | --- | --- | --- | --- | --- |
| 243 | Video Laryngoscopy vs Direct Laryngoscopy on Successful First-Pass Orotracheal Intubation Among ICU Patients A Randomized Clinical Trial  Lascarrou et al. | Critical care | 2/7/2017  JAMA | Given the inherent risk - such as cardiac arrest - that comes with intubating patients, indirect video laryngoscopy (VL) was developed to assist with the intubation process and reduce morbidity and mortality.^1^ Several types of these devices were commercially available in 2014.^2^ However, in a randomized control study of 371 patients in the ICU, it was found that, compared to direct laryngoscopy (DL, n=185), VL (n=186) was not beneficial in improving first-pass intubation success rates (67.7% for VL vs 70.3% for direct; absolute difference, -2.5%; 95% CI, -11.9% to 6.95; P=.60). Furthermore, VL was associated with increased rates of severe life-threatening complications compared to DL (absolute difference, 6.7%; 95% CI, 1.8% to 11.6%; P=.01). This is a reversal of video laryngoscopy for first-pass orotracheal intubation in ICU patients. | 2017. “The VL technique did not increase the first-attempt success rate during EI [endotracheal intubation] in ICU patients compared with DL. These findings do not support routine use of VL in ICU patients.”^3^ | GoogleScholar _ cited RCT |
| 244 | Effect of 2 Years of Treatment With Sublingual Grass Pollen Immunotherapy on Nasal Response to Allergen Challenge at 3 Years Among Patients With Moderate to Severe Seasonal Allergic Rhinitis The GRASS Randomized Clinical Trial  Scadding et al. | Allergy and immunology | 2/14/2017  JAMA | Rhinitis due to allergens is highly prevalent, and various treatment approaches exist. One option for treatment has been the use of sublingual immunotherapy in the form of droplets or tablets to increase the patient's exposure to the allergen, thereby increasing tolerance and improving the patient's symptoms.^4^ This randomized controlled trial compared sublingual immunotherapy (n=30), subcutaneous immunotherapy (positive control, n=31), and placebo (n=31) and their effect in patients with moderate to severe seasonal allergic rhinitis after 2 years of treatment. At 3 year follow up, the between-group difference was -0.18 (95% CI, -1.25 to 0.90; P=.75). Sublingual immunotherapy did not improve nasal response compared to placebo. This is a reversal of sublingual grass pollen immunotherapy for improving seasonal allergy symptoms. | 2017. “AIT [allergen immunotherapy] did not result in a statistically significant reduction in the risk of developing a first allergic disease.”^5^ The review did not include the RCT. | immunotherapy and allergic rhinitis |
| 245 | Testosterone Treatment and Cognitive Function in Older Men With Low Testosterone and Age-Associated Memory Impairment  Resnick et al. | Endocrinology, Diabetes, and Metabolism | 2/21/2017  JAMA | Some studies have suggested that testosterone therapy may improve cognitive function as people age, because aging is associated with declines in both testosterone levels and cognitive function. ^6 7^ Sales of testosterone increased 500% between 1993 and 2001 to treat symptoms of hypogonadism, including cognitive function.^8^ This randomized control trial investigated the cognitive effects of testosterone treatment in men 65 years or older with low testosterone, hypogonadism symptoms, and age-associated memory impairment. The study found that men in the testosterone group (n=247) did not show improved delayed paragraph recall compared to men in the placebo group (n=245) from baseline to 6 and 12 months (adjusted estimated difference, -0.07; 95% CI, -0.92 to 0.79; P=.88). This is a reversal of testosterone treatment for improving cognitive function in older men with low testosterone and age-associated memory impairment. | None found | testosterone and cognitive |
| 246 | Effect of Fibrinogen Concentrate on Intraoperative Blood Loss Among Patients With Intraoperative Bleeding During High-Risk Cardiac Surgery A Randomized Clinical Trial  Bilecen et al. | Surgery | 2/21/2017  JAMA | Due to the likelihood of excessive intraoperative bleeding during high-risk cardiac surgery, it is common to administer fibrinogen concentrate (FC) in order to maintain homeostasis during surgery. ^9 10^ Despite its common usage to control bleeding, it is unknown whether FC's use in this manner is effective. A randomized control trial enrolling 120 patients who underwent cardiac surgery found that, among the patients who experienced intraoperative bleeding, there was no difference in intraoperative bleeding when comparing the fibrinogen group (n=58) with those receiving placebo (n=57). The fibrinogen group had a median blood loss of 50 mL vs 70 mL in the placebo group (P=.19; absolute difference, 20 mL; 95% CI, -13 to 35 mL). This is a reversal of FC to control bleeding during high-risk cardiac surgery. | 2016. “In surgical patients, FC was associated with reduced bleeding and a lower number of red blood cell units transfused, and it also might reduce mortality. However, none of the analyzed trials was powered for estimation of survival and adverse events with FC use.”^11^ This review did not include the RCT. | fibrinogen concentrate during surgery |
| 247 | Effect of Inpatient Rehabilitation vs a Monitored Home-Based Program on Mobility in Patients With Total Knee Arthroplasty The HIHO Randomized Clinical Trial  Buhagiar et al. | Orthopedic | 3/14/2017  JAMA | Total knee arthroplasties are performed frequently, and debate over the best form of rehabilitation persists. Some advocate for formal inpatient rehabilitation programs, while others hold that monitored home-based programs are sufficient to rehabilitate mobility and function and improve quality of life.^12^ The United States offers formal inpatient rehabilitation after knee surgery at higher rates than countries such as Canada or the UK, suggesting potential overuse or underuse.^13^ This multicenter, randomized control trial compared mobility improvement through inpatient rehabilitation (n=81) vs home programs (n=84) in patients with osteoarthritis undergoing primary total knee arthroplasty. The study found no significant difference in 6-minute walk test at 26 weeks after surgery between inpatient and home program groups (mean difference, -1.01; 95% CI, -25.56 to 23.55). This is a reversal of inpatient rehabilitation in place of monitored home-based programs `for improving mobility in patients with total knee arthroplasties. | 2017. “Home-based rehabilitation after primary TKA was comparable to hospital-based rehabilitation and thus is a significant alternative for patients.“^14^ This SR/MA did not include this study. | inpatient rehabilitation vs home program knee arthroplasty |
| 248 | Effect of an Integrated Pest Management Intervention on Asthma Symptoms Among Mouse-Sensitized Children and Adolescents With Asthma A Randomized Clinical Trial  Matsui et al. | Pediatrics | 3/14/2017  JAMA | It is a widely held belief that pests, such as mice, cockroaches, and other critters, can lead to asthma attacks,^15 16^ which are increasingly prevalent in our society.^17^ Professional pest management companies include an integrated pest management service, which has been advocated for by public and private organizations for reducing asthma in children.^15 16^ In this trial, an integrated pest management service with education (IPM, n=166) was no better than education alone (n=168) for reducing asthma symptoms in children. Maximal symptom days did not differ between groups at 6, 9, and 12 months (median max symptom days, 2.0 in IPM group vs 2.7 in education alone group; P=.16). This is a reversal of integrated pest management interventions for improving asthma symptoms in children and adolescents with asthma. | None found |  |
| 249 | Effect of Dexmedetomidine on Mortality and Ventilator-Free Days in Patients Requiring Mechanical Ventilation With Sepsis A Randomized Clinical Trial  Kawazoe et al. | Critical care | 4/4/2017  JAMA | Dexmedetomidine, a sedative agent, became increasingly popular for the sedation of patients in the intensive care unit due to its ability to promote “cooperative sedation”, where patients are awake, aware, and able to communicate pain to health care providers while remaining calm and lightly sedated.^18^ In this randomized controlled trial, the use of dexmedetomidine (n=100) vs no dexmedetomidine (n=101) did not lead to improvements in mortality and ventilator-free days over a 28-day duration. Mortality in the dexmedetomide was 19 patients (22.8%) vs 28 patients (30.8%) in the control group (HR, 0.69; 95% CI, 0.38-1.22; P=0.20). Median ventilator-free days was 20 days vs 18 days (P=0.20). This is a reversal of dexmedetomidine for sedation of patients requiring mechanical ventilation with sepsis. | None found | sedation and mechanical ventilation in sepsis |
| 250 | Effect of Intra-articular Triamcinolone vs Saline on Knee Cartilage Volume and Pain in Patients With Knee Osteoarthritis A Randomized Clinical Trial  McAlindon et al. | Orthopedic | 5/16/2017  JAMA | Osteoarthritis of the knee is a common condition, and it is often recommended that patients with osteoarthritis be treated with corticosteroid injections.^19^ However, despite the prevalence of its use in this population,^20^ there is debate regarding the association with corticosteroid injections and joint damage. A 2-year, randomized, placebo-controlled, double-blind trial found that, when compared with a placebo injection (n=70), patients with symptomatic knee osteoarthritis who underwent intra-articular triamcinolone injections (n=70) experienced significantly higher rates of cartilage volume loss (triamcinolone vs saline, -0.21 mm vs -0.10 mm; between-group difference, -0.11 mm; 95% CI, -0.20 to -0.03 mm; P=.01) and no improved knee pain (-1.2 vs -1.9; between-group difference, -0.6; 95% CI, -1.6 to 0.3). This is a reversal of corticoid injections for patients with osteoarthritis of the knee. | None found | corticosteroids and osteoarthritis |
| 251 | Association Between Long-Lasting Intravitreous Fluocinolone Acetonide Implant vs Systemic Anti-inflammatory Therapy and Visual Acuity at 7 Years Among Patients With Intermediate, Posterior, or Panuveitis  MUST trial group | Ophthalmology | 5/16/2017  JAMA | Noninfectious intraocular inflammation, or uveitis, can lead to visual impairment. Currently, there are two treatments commonly used for uveitis; the first approach is through systemic corticosteroids and corticosteroid-sparing immunosuppressive drugs.^21^ The other, more recent approach was approved by the FDA in 2005 and involves surgically implanting fluocinolone acetonide implants. ^22^ When systemic therapy (n=126) and intravitreous implants (n=129) approaches were compared with one another in a randomized control trial, it was found that after seven years of follow up, those that were randomized to receive implants had poorer visual acuity than the group who were treated with systemic therapy. Change in mean visual acuity from baseline through 7 years was 1.15 in the systemic therapy group and -5.96 in the implant group (between-group difference, -7.12; 95% CI, -12.4 to -2.14; P=.006). This is a reversal of intravitreous fluocinolone acetonide implants for uveitis. | None found | fluocinolone acetonide implant and panuveitis |
| 252 | Effect of Acupuncture and Clomiphene in Chinese Women With Polycystic Ovary Syndrome A Randomized Clinical Trial  Wu et al. | Obstetrics and gynecology | 6/27/2017  JAMA | Women with polycystic ovary syndrome (PCOS) often struggle with infertility. The first-line treatment for women in this category is clomiphene citrate, a medication meant to induce ovulation, even though it has a high failure rate at 23.4% after five months use.^23^ Given the lack of success with this approach alone, adjuvant treatments, such as acupuncture, are often recommended in addition to clomiphene to increase fertility.^24^ The PCOS Acupuncture and Clomiphene Trial found that there was no increase in live birth rates from acupuncture, with (n=235; 29.4%) or without clomiphene (n=223; 13.9%), compared to clomiphene with control acupuncture (n=236; 28.0%; P=.39). This is a reversal of acupuncture for improving live birth rates in women with PCOS. | 2016. A recent SR/MA of RCTs found that acupuncture was not associated with better pregnancy outcomes (clinical pregnancy, live birth) in women with polycystic ovary syndrome, although the number of studies was small and did not include this study.^25^ | acupuncture and polycystic ovary syndrome |
| 253 | Effect of Radiofrequency Denervation on Pain Intensity Among Patients With Chronic Low Back Pain The Mint Randomized Clinical Trials  Juch et al. | Orthopedic | 7/4/2017  JAMA | Radiofrequency denervation is often recommended for people suffering from chronic low back pain, despite conflicting evidence to its efficacy.^26^ The treatment aims to damage the pain-conducting nerve to reduce sensations of pain and is minimally invasive, and therefore anesthesiologists support its use in patients with chronic low back pain.^26^ Three multicenter, nonblinded randomized control trials compared the effects of radiofrequency denervation plus standard care versus standard care alone in patients with chronic low back pain, a positive diagnostic block at the facet joints (facet joint trial, 251 participants), sacroiliac joints (sacroiliac joint trial, 228 participants), or a combination of facet joints, sacroiliac joints, or intervertebral disks (combination trial, 202 participants) and were unresponsive to conservative care. The studies found no difference in clinical improvement between patients who underwent radiofrequency denervation and those who underwent a standardized exercise program alone. The mean differences in pain intensity between denervation and standard care groups was −0.18 (95% CI, −0.76 to 0.40) in the facet joint trial; −0.71 (95% CI, −1.35 to −0.06) in the sacroiliac joint trial; and −0.99 (95% CI, −1.73 to −0.25) in the combination trial. This is a reversal of radiofrequency denervation to reduce pain in patients with chronic low back pain. | 2014. A SR/MA of RCTs and quasi-RCTs concluded that facet joint radiofrequency was more effective than placebo or steroid injection in pain control. However, the quality of these studies was low and did not use an active control group, such as exercise.^27^ | radiofrequency denervation and back pain |
| 254 | Effect of Oral Methylprednisolone on Clinical Outcomes in Patients With IgA Nephropathy The TESTING Randomized Clinical Trial  Jicheng et al. | Nephrology | 8/1/2017 | Immunoglobulin A nephropathy often leads to end-stage kidney disease and other kidney problems. Corticosteroid therapy is often recommended as treatment, despite conflicting evidence about its efficacy and safety.^28^ In a multicenter, double-blind, randomized study that set out to establish the safety and efficacy of methylprednisolone (full-dose corticosteroid therapy; n=134) compared to placebo (n=126), it found that methylprednisolone was associated with a greater incidence of serious adverse events (14.7% in methylprednisone group vs 3.2% in placebo group; P=.001; risk difference, 11.5%, 95% CI, 4.8%-18.2%), so much so that the trial was discontinued. This is a reversal of administering oral methylprednisolone to patients with IgA nephropathy. | 2015. Cochrane review. “The optimal management of IgAN remains uncertain although corticosteroid therapy may lower the risks of kidney disease progression and need for dialysis or transplantation. Evidence for treatment effects of immunosuppressive agents on mortality, infection, and cancer is generally sparse or low-quality and insufficient to guide clinical practice.” ^29^ This review did not include this RCT. | PubMed |
| 255 | Effect of Endovascular Contact Aspiration vs Stent Retriever on Revascularization in Patients With Acute Ischemic Stroke and Large Vessel Occlusion  The ASTER Randomized Clinical Trial  Lapergue et al. | Neurology | 8/1/2017  JAMA | Mechanical thrombectomy has become the new standard of care for patients with acute ischemic stroke and large vessel occlusion, but debate remains about the appropriate technique for endovascular revascularization - specifically between contact aspiration and stent retriever technique, with contact aspiration gaining popularity.^30^ The Contact Aspiration vs Stent Retriever for Successful Revascularization (ASTER) trial assessed the two techniques and found that among patients with ischemic stroke in the anterior circulation undergoing thrombectomy, there was no additional benefit among patients who underwent contact aspiration compared to stent retriever. The proportion of patients with successful revascularization in the contact aspiration group was 85.4% (n = 164) vs 83.1% (n = 157) in the stent retriever group (OR, 1.20; 95% CI, 0.68-2.10; P = .53; difference, 2.4%; 95% CI, −5.4% to 9.7%). | 2017. “In a separate network of seven RCTs (MR-CLEAN, ESCAPE, EXTEND-IA, SWIFT-PRIME, REVASCAT, THERAPY, ASTER; 1737 patients), first-line stent retriever was associated with a higher top rank probability of functional independence than aspiration (95% vs 54%), with comparable safety outcomes..”^31^ | Google Scholar - MA cited RCT |
| 256 | Effect of Cerebral Embolic Protection Devices on CNS Infarction in Surgical Aortic Valve Replacement A Randomized Clinical Trial  Mack et al. | Neurology | 8/8/2017  JAMA | After patients undergo surgical aortic valve replacement or transcatheter aortic valve replacement (TAVR), they are at increased risk for central nervous system infarction. As such, cerebral embolic protection devices have been developed, namely the Embol-X (Edwards Lifesciences) intra-aortic filtration and CardioGard (CardioGard) devices.^32^ A trial evaluating the efficacy and adverse effects of such devices found that, when comparing a suction-based extraction device (n=118) and an intra-aortic filtration device (n=133) with a standard aortic cannula (n=132), there was no significant reduction in risk of central nervous system (CNS) injury in patients undergoing TAVR. The absolute difference in rate of freedom from CNS infarction at 7 days compared to control was 1.3 (95% CI, -11.2 to 13.8; P=84) for suction-based extraction and 6.9 (95% CI, -4.2 to 17.9%; P=.22) with intra-aortic filtration. This is a reversal of embolic protection devices for protecting against infarction during surgical aortic valve replacement. | 2016. “Use of EP [embolic protection] seems to be associated with reductions in imaging markers of cerebral infarction and early clinical neurological effectiveness in patients undergoing TAVR.”^33^ The RCT was not included as part of this review. A total of 252 participants from 4 studies were included in this meta-analysis compared to the 380 participants in the Mack et al study. | cerebral embolic protection devices and aortic valve replacement |
| 257 | Effect of Levosimendan on Low Cardiac Output Syndrome in Patients With Low Ejection Fraction Undergoing Coronary Artery Bypass Grafting With Cardiopulmonary Bypass The LICORN Randomized Clinical Trial  Cholley et al. | Cardiovascular | 8/8/2017  JAMA | Low cardiac output syndrome after coronary bypass grafting surgery is associated with a higher risk of complications, such as pulmonary impairment, myocardial infarction, stroke, and renal failure.^34^ Treatment for low cardiac output syndrome had typically involved inotropic agents and mechanical assist devices, yet current inotropic agents have increased risk of morbidity and mortality after surgery. Levosimendan is a calcium sensitizing inotropic agent used in patients undergoing cardiac surgery that was thought to have fewer adverse events.^35 36^ This study found that, compared to placebo (n=156), levosimendan (n=158) did not reduce the incidence of the composite end point of prolonged catecholamine infusion, use of left ventricular mechanical assist device, or renal replacement therapy (occurred in 52% of levosimendan group vs 61% placebo group) in patients with low ejection fraction who underwent coronary artery bypass grafting (absolute risk difference taking into account center effect, −7%; 95% CI, −17% to 3%; P = .15). This is a reversal of levosimendan in treating low cardiac output syndrome in patients with low ejection fraction undergoing coronary artery bypass grafting with cardiopulmonary bypass. | 2018. “Pooled analysis of 5 low risk of bias trials (1910 patients) showed no association between levosimendan and mortality (OR 0.86 [95% CI, 0.62, 1.18], p=0.34, TSA inconclusive), acute kidney injury, need of renal replacement therapy, myocardial infarction, ventricular arrhythmias, and serious adverse events, but an association with higher incidence of supraventricular arrhythmias (RR 1.11 [95% CI, 1.00, 1.24], p=0.05, TSA inconclusive) and hypotension (RR 1.15 [95% CI, 1.01, 1.30], p=0.04, TSA inconclusive).” ^37^ | levosimendan low cardiac output syndrome "Coronary Artery Bypass" |
| 258 | Effect of Natriuretic Peptide–Guided Therapy on Hospitalization or Cardiovascular Mortality in High-Risk Patients With Heart Failure and Reduced Ejection Fraction A Randomized Clinical Trial  Felker et al. | Cardiovascular | 8/22/2017  JAMA | Patients with heart failure (HF) were shown to have significantly improved outcomes when administered evidence-based therapies targeting neurohormonal activation. Yet many patients are not always treated with these agents or are treated with inappropriate doses in clinical practice.^38 39^ Natriuretic peptides are biomarkers that are associated with adverse outcomes in heart failure and have shown to decline in response to recommended therapies.^40^ While not the standard of care, natriuretic peptide-guided therapy for heart failure has been mentioned in AACF/AHA guidelines and has been studied in clinical trials over the last 2 decades.^41^ This study found that biomarker-guided therapy (n=446) was no more effective than a usual care strategy (n=448) for preventing a composite of HF hospitalization or cardiovascular mortality (37% in biomarker-guided group vs 37% in usual care group; adjusted HR. 0.98; 95% CI, 0.65-1.37; P=.75) in high-risk patients with HF and reduced ejection fraction. This is a reversal of natriuretic peptide-guided therapy for high-risk patients with HF and reduced ejection fraction. | None found | BNP guided therapy and heart failure |
| 259 | Effect of Axillary Dissection vs No Axillary Dissection on 10-Year Overall Survival Among Women With Invasive Breast Cancer and Sentinel Node Metastasis The ACOSOG Z0011 (Alliance) Randomized Clinical Trial  Guiliano et al. | Oncology | 9/12/2017  JAMA | Axillary lymph node dissection (ALND) and sentinel lymph node dissection (SLND) have both been used in the treatment of breast cancer. ALND is a more invasive procedure and was the considered standard of care in the 1990s. By the end of the 1990s, ALND had fallen out of practice while SLND, a less invasive therapy, became more popular.^42-45^ This study compared ALND (n=420) to SLND (n=436) in patients with cT1-2N0 breast cancer and metastases to 1 or 2 sentinel lymph nodes and found that 10-year overall survival was 86.3% in the SLND alone group and 83.6% in the ALND group (HR, 0.85; 1-sided 95% CI, 0-1.16; noninferiority P = .02). SLND was noninferior to ALND. | 2015. “ALND appears to positively impact on overall and recurrence-free survival from breast cancer. These data highlight the enduring benefits of ALND in an era where adjuvant therapies are being promoted to manage regionally advanced/metastatic disease.”^46^ This review did not include the RCT and included observational studies. | axillary dissection with invasive breast cancer |
| 260 | Effect of Routine Low-Dose Oxygen Supplementation on Death and Disability in Adults With Acute Stroke The Stroke Oxygen Study Randomized Clinical Trial  Roffe et al. | Neurology | 9/26/2017  JAMA | Stroke patients often experience hypoxia, which can lead to neurologic problems and higher mortality rates. As such, several guidelines have advocated the use of oxygen in stroke patients to prevent hypoxia and secondary brain damage.^47 48^ This study compared continuous oxygen (n=2567), nocturnal oxygen (2561), and control (oxygen only if clinically indicated n=2549) in patients with acute stroke and found that there was no difference in disability between the oxygen groups and control group (OR, 0.97; 95% CI, 0.89-1.05; P=.47). This is a reversal of routine low-dose oxygen supplementation to prevent disability in patients with acute stroke. | None found | oxygen supplementation in stroke |
| 261 | Effect of an Early Resuscitation Protocol on In-hospital Mortality Among Adults With Sepsis and Hypotension A Randomized Clinical Trial  Andrews et al. | Critical care | 10/3/2017 | A decline in rates of mortality from sepsis has been seen in developed countries,^49^ and many accredit the decline to sepsis protocols that call for early resuscitation with intravenous (IV) fluid boluses and vasopressors.^50^ ^51^ In developing countries, where sepsis mortality continues to be a pressing issue, many guidelines do not call for early resuscitation with IV fluid and vasopressors. Attempts to implement these sepsis guidelines have led to mixed results.^52-54^ In this study, an early resuscitation protocol for sepsis (n=106) compared to usual care (n=103) lead to increased in-hospital mortality rates in Zambian adults with sepsis and hypotension (48.1% in sepsis protocol group vs 33.0% in usual care group; RR, 1.46; 95% CI, 1.04-2.05; P=.03). This is a reversal of early resuscitation protocol for sepsis in resource-limited settings. | 2015. “EGDT [early goal-directed therapy] is not superior to usual care for ED patients with septic shock but is associated with increased utilisation of ICU resources.”^55^ This review did not include the RCT. | early resuscitation protocol and sepsis and hypotension |
| 262 | Effect of Lung Recruitment and Titrated Positive End-Expiratory Pressure (PEEP) vs Low PEEP on Mortality in Patients With Acute Respiratory Distress Syndrome A Randomized Clinical Trial  ART Investigators | Critical care | 10/10/2017  JAMA | Use of mechanical ventilation for patients with acute respiratory distress (ARDS) can, in some cases, lead to lung injury. Positive end-expiratory pressure (PEEP) has been used to prevent injury, but whether to use low or high PEEP has been questioned.^56-59^ One meta-analysis, based on results from several trials, suggested found in a sub analysis the use of titration that higher levels in PEEP is associated with improved survival in patients with ARDS.^58^ High PEEP was used frequently in the 1970’s and is now sometimes part of open lung and protective lung ventilation strategies.^60^ This trial found that lung recruitment and titrated PEEP (n=501), when compared to low PEEP (n=509), led to higher 28-day all-cause mortality (HR, 1.20; 95% CI, 1.01 to 1.42; P=0.41). This is a reversal of titrated PEEP for patients with ARDS. | 2017. “Use of higher PEEP is unlikely to improve clinical outcomes among unselected patients with ARDS.”^61^ This review did not include the RCT. | titrated positive end-expiratory pressure vs. low peep ARDS |
| 263 | Effect of Robotic-Assisted vs Conventional Laparoscopic Surgery on Risk of Conversion to Open Laparotomy Among Patients Undergoing Resection for Rectal Cancer The ROLARR Randomized Clinical Trial  Jayne et al. | Oncology | 10/24-31/2017  JAMA | Robotic-assisted laparoscopic surgery was first used for rectal cancer in 2007,^62^ and its use has increased in the past decade because of better visualization of the surgical site and improved dexterity.^63^ In this trial, patients undergoing surgery for rectal adenocarcinoma did not have reduced risk of conversion to open laparotomy with the use of robotic-assisted laparoscopic surgery (n=235), compared to those who received conventional laparoscopic surgery (n=224). Rate of conversion to open laparotomy was 8.1% in the robotic-assisted laparoscopic group and 12.2% in the conventional laparoscopic group (unadjusted risk difference = 4.1%; 95% CI, −1.4% to 9.6%; adjusted OR = 0.61; 95% CI, 0.31 to 1.21; P = .16). This is a reversal of robotic-assisted laparoscopic surgery for patients undergoing resection for rectal cancer. | None found | robotic-assisted vs conventional laparoscopic surgery rectal cancer resection |
| 264 | Effect of a Single Dose of Oral Opioid and Nonopioid Analgesics on Acute Extremity Pain in the Emergency Department A Randomized Clinical Trial  Chang et al. | Public health/Preventive medicine | 11/7/2017  JAMA | Opioids are commonly prescribed for pain in the emergency department and are the first-line pain relievers that are administered, but with the increasing number of people becoming dependent on them, alternative treatments should be evaluated.^64^ Ibuprofen and acetaminophen are nonopioid alternatives that are commonly used together for the treatment of pain.^65^ This study randomized patients with moderate to severe acute extremity pain admitted to the ER to one of four groups, which were administered acetaminophen plus: (1) ibuprofen (n=101), (2) oxycodone (n=104), (3) hydrocodone (n=103), or (4) codeine (n=103). The study found that opioid-based analgesics did not lead to better pain control than a combination of ibuprofen and acetaminophen. Two hours after ingestion, decline in pain scores in the ibuprofen group was 4.3, oxycodone was 4.4, hydrocodone was 3.5, and codeine was 3.9 (P=.053). The greatest difference pain score decline was between the oxycodone and hydrocodone groups (0.9; 99.2% CI, −0.1 to 1.8), which was less than the minimum clinically important difference in pain score of 1.3. This is a reversal of opioid-based analgesics for acute extremity pain the emergency department. | There were several MA on pain management but none were found that were in the emergency room and only one was found that directly compared opioids and nonopioids, but this was written in German. | opioid vs nonopioid analgesics in emergency department |
| 265 | Effect of Sertraline on Depressive Symptoms in Patients With Chronic Kidney Disease Without Dialysis Dependence The CAST Randomized Clinical Trial  Hedayati et al. | Psychiatry | 11/21/2017  JAMA | Up to 25% of patients with chronic kidney disease have depression, which is 4 times higher than in the general population.^66^ Because of safety concerns, people with chronic kidney disease are not always included in depression medication trials, although these medications, including sertraline, are recommended for patients with depression, regardless of chronic kidney disease status. ^67 68^ In this study, sertraline (n=97) was no better than placebo (n=96) in treating depression in patients with non-dialysis-dependent chronic kidney disease. Difference in depressive symptom severity scores from baseline to 12 weeks was -4.1 in the sertraline group and -4.2 in the placebo group (between-group difference, 0.1; 95% CI, -1.1 to 1.3; P=.82). This is a reversal of sertraline for treating depressive symptoms in patients with chronic kidney disease. | None found | sertraline depression chronic kidney disease (also antidepressants or SSRI in place of sertraline) |
| 266 | Effect of a Quality Improvement Intervention With Daily Round Checklists, Goal Setting, and Clinician Prompting on Mortality of Critically Ill Patients A Randomized Clinical Trial  CHECKLIST-ICU Investigators | Critical care | 4/12/2016  JAMA | Recently, it has been proposed that checklists be used to ensure that healthcare providers do not omit critical elements of care in places such as the intensive care unit (ICU).^69^ Various studies suggest that such checklists lead to improved quality of care and patient outcomes.^70 71^ However, evidence is lacking to support this hypothesis. A randomized controlled trial in Brazil found that the use of a multifaceted quality improvement intervention with daily checklists (n of patients= 3327; n of ICUs=59) did not reduce mortality rates in ICUs compared to routine care (n of patients= 3434; n of ICUs=59). In-hospital mortality at 60 days was similar between groups, with 1096 deaths (32.9%) in the intervention group and 1196 deaths (34.8%) in the routine care group (OR, 1.02; 95% CI, 0.82-1.26; P=.88). This is a reversal of multifaceted quality improvement intervention with daily checklists in ICUs. | None found | quality improvement (with our without checklist) intervention in critically ill |
| 267 | Effect of Chemoradiotherapy vs Chemotherapy on Survival in Patients With Locally Advanced Pancreatic Cancer Controlled After 4 Months of Gemcitabine With or Without Erlotinib The LAP07 Randomized Clinical Trial  Hammel et al. | Oncology | 5/3/2016  JAMA | Administering chemotherapy plus adjuvant radiotherapy in the treatment of locally advanced prostate cancer (LAPC) is commonly practiced,^72^ although the addition of radiotherapy is controversial and not recommended outside of clinical trials for this patient population.^73^ A 2003 national study found that between 1991 and 1996, 24% of patients with LAPC received chemoradiation.^72^ This randomized controlled trial investigated the effects of chemotherapy (n=136) vs chemoradiotherapy (n=133) in patients with LAPC with disease controlled after 4 months of induction chemotherapy. Median overall survival was not significantly different between chemotherapy at 16.5 months (95% CI, 14.5-18.5 months) and chemoradiotherapy at 15.2 months (95% CI, 13.9-17.3 months; HR, 1.03; 95% CI, 0.79-1.34; P = .83). This is a reversal of chemoradiotherapy versus chemotherapy alone for patients with locally advanced pancreatic cancer. | 2016. “This meta-analysis showed that CRT [chemoradiotherapy] showed no significant effect on OS [overall survival] and PFS [progression-free survival] when compared to non-CRT. Neoadjuvant CRT showed no significant effect over postoperative adjuvant CRT.”^74^ This review did not include the RCT. | chemotherapy vs chemoradiotherapy in pancreatic cancer |
| 268 | Effect of Escitalopram on All-Cause Mortality and Hospitalization in Patients with Heart Failure and Depression The MOOD-HF Randomized Clinical Trial  Angermann et al. | Psychiatry | 6/28/2016  JAMA | Depression after heart failure is common and has been associated with poor health outcomes.^75^ Psychotherapy and pharmacological medicine are generally used to treat patients with heart failure and depression,^76^ but there is not strong evidence for their efficacy in this population in reducing depression or cardiovascular morbidities.^77^ This study compared the effect of escitalopram (n= 185), a selective serotonin reuptake inhibitor, versus placebo (n=187) on a composite of time to all-cause death or hospitalization in patients with chronic heart failure with reduced ejection fraction and depression and found no benefit to the drug (HR, 0.99; 95% CI, 0.76-1.27; P=.92). Furthermore, escitalopram did not improve depression compared to placebo (mean depression rating score, 21.4 vs 12.5; between-group difference, -0.9; 95% CI, -2.6 to 0.7; P=.26). This is a reversal of escitalopram to reduce incidence of death or hospitalization and for reducing depressive symptoms in patients with heart failure and depression. | None found |  |
| 269 | Effect of Palliative Care–Led Meetings for Families of Patients With Chronic Critical Illness A Randomized Clinical Trial  Carson et al. | Critical care | 7/5/2017  JAMA | Family members of patients with chronic critical illness often experience emotional distress, including anxiety, depression, and post-traumatic stress disorder (PTSD).^78^ Support and communication of expected outcomes of patients with chronic critical illness is often insufficient in helping families to create goals of care.^79^ While various interventions to facilitate communication about prognosis and goals of care in the ICU have been investigated, their results are mixed.^80 81^ The 2008 American Thoracic Society Clinical Policy Statement recommends that palliative care specialists offer support for families in planning and providing care.^82^ This study found that among families of patients with chronic critical illness, palliative care-led support meetings (n=130) did not reduce anxiety or depression compared to usual care (n=126) and may have increased PTSD (P=.0495). Hospital Anxiety and Depression Scale symptom scores for intervention and placebo at 3 months were 12.2 vs 11.4, respectively (between-group difference, 0.8; 95% CI, -0.9 to 2.6; P=.34). Palliative care support meetings in this population did not show benefit. This is a reversal of palliative care-led meetings for families of patients with chronic critical illness for reducing PTSD severity. | 2017. “Despite the existence of consensus-based family meeting guidelines, there is a paucity of evidence to support family meetings in the inpatient palliative care setting.”^83^ This review did not include this RCT. | palliative care and meetings for families of patients |
| 270 | Effect of Patient Navigation With or Without Financial Incentives on Viral Suppression Among Hospitalized Patients With HIV Infection and Substance Use A Randomized Clinical Trial  Metsch et al. | Infectious | 7/21/2016  JAMA | Patient navigation was first used in 2007 to help patients in underserved populations receive the best available care in order to improve health outcomes.^84^ Financial incentives to encourage people to engage in healthy habits have been used in the US for at least several decades, although use in the field of HIV has been more recent.^85^ In this study, patient navigation with (n=263) or without (n=255) financial incentives did not result in better HIV viral suppression or death, compared to usual care (n=256). The usual care group experienced treatment success in 34.1% of patients, compared to navigation-only at 35.7% (treatment difference, 1.6%; 95% CI, -6.8 to 10.0%; P=.80) and compared to navigation plus incentives at 38.6% (treatment difference, 4.5%, 95% CI, -4.0% to 12.8%: P=.68). This is a reversal of patient navigation and financial incentives for patients with HIV infections. | None found | cash (or financial) incentives and hiv |
| 271 | Effect of Radiosurgery Alone vs Radiosurgery With Whole Brain Radiation Therapy on Cognitive Function in Patients With 1 to 3 Brain Metastases A Randomized Clinical Trial  Brown et al. | Oncology | 7/26/2016  JAMA | Stereotactic radiosurgery (SRS) is the mainline treatment for patients with brain metastases, but this treatment alone is often insufficient to stop intracranial tumor progression. As such, whole brain radiotherapy (WBRT) is often recommended afterwards, despite conflicting evidence that this may lead to cognitive deterioration and decreased quality of life.^86 87^ A multi-institutional randomized control trial found that - among patients with 1 to 3 brain metastases - SRS alone (n=63) resulted in fewer patients with cognitive deterioration at three months than did the group that underwent SRS and adjuvant WBRT (n=48). Among SRS alone, 63.5% of patients experienced cognitive decline compared to 91.7% in the WBRT group, with a difference of -28.2% (90% CI, -41.9% to -14.4%; P=.001). This is a reversal of WBRT for patients with 1 to 3 brain metastases. | 2017. A SR/MA that included this study found that overall tumor control rate was better among those receiving SRS, compared to SRS+WBRT, but overall survival was no better. This MA did not look at cognitive deterioration.^88^ | GoogleScholar - MA cited RCT |
| 272 | Effect of Topical Intranasal Therapy on Epistaxis Frequency in Patients With Hereditary Hemorrhagic Telangiectasia A Randomized Clinical Trial  Whitehead et al. | Public health/Preventive medicine | 9/6/2017  JAMA | Hereditary hemorrhagic telangiectasia (HHT) is a genetic condition that causes abnormal blood vessel formation in mucus membranes and commonly leads to recurrent and spontaneous epistaxis. Treatment for epistaxis in this population can be serious, requiring cautery and surgery, but other less invasive methods, such as systemic oestrogens and nasal sprays containing bevacizumab or tranexamic acid have also been used to prevent subsequent bleeding.^89 90^ In this randomized controlled trial, twice daily nose spray treatments of bevacizumab (n=24), estriol (n=25), tranexamic acid (n=30), or placebo (n=27) were given to patients with HHT-related epistaxis. None of the interventions tested were superior to placebo in reducing epistaxis frequency (P=.97). This is a reversal of topical intranasal therapy for patients with hereditary hemorrhagic telangiectasia. | None found | hemorrhagic telangiectasia and intranasal (with or without tranexamic acid or bevacizumab) therapy |
| 273 | Effect of Wearable Technology Combined With a Lifestyle Intervention on Long-term Weight Loss The IDEA Randomized Clinical Trial  Jakicic et al. | Public health/Preventive medicine | 9/20/2017  JAMA | Wearable technologies have become increasingly popular as tools to assist in weight loss since they help track physical activity and estimate calorie burn.^91^ This clinical trial randomized adults who were participating in a weight-loss program (including a low-calorie diet, increases in physical activity, group counseling sessions, telephone counseling sessions, text message prompts, and access to study materials on a website) to use a wearable device and accompanying web interface (enhanced intervention group, n=237) or to a self-monitoring website (standard intervention group, n=233). The study found that the standard intervention group experienced significantly more weight loss than the enhanced intervention group after 24 months (5.9 kg vs 3.5 kg; difference 2.4 kg; 95% CI, 1.0-3.7; P=.002). This is a reversal of wearable technology for long-term weight loss. | 2017.While this review concluded that wearable technology reduces sedentary behavior, there were no SR/MA on whether these devices reduce weight.^92^ This review did not include the RCT. | wearable technology and weight loss |
| 274 | Effect of Cranberry Capsules on Bacteriuria Plus Pyuria Among Older Women in Nursing Homes A Randomized Clinical Trial  Juthani-Mehta et al. | Urology | 11/8/2016  JAMA | Bacteriuria plus pyuria is prevalent among female residents at nursing homes, and it is often recommended that women take cranberry capsules to prevent such infections from occurring in lieu of treatment with antibiotics. This randomized control trial found that, compared to a placebo (n=93), prophylactic treatment with cranberry capsules (n=92) for women in nursing homes did not alter the presence of bacteriuria plus pyuria over the course of a year (29.1% in the cranberry group vs 29.0% in the placebo group; OR, 1.01; 95% CI, 0.61-1.66; P=.98). This is a reversal of cranberry capsules for preventing bacteriuria plus pyuria in older women in nursing homes. | None found | cranberry for urinary tract infections in nursing homes |
| 275 | Chlorhexidine Bathing and Health Care–Associated Infections A Randomized Clinical Trial  Noto et al. | Public health/Preventive medicine | 1/27/2015  JAMA | In order to prevent infections, it is common practice to bathe hospitalized patients with broad-spectrum antimicrobial agents such as chlorhexidine. This is based on the understanding that skin can host a number of pathogens.^93^ However, this randomized clinical trial found that, compared to non-antimicrobial cloths (n=4852), daily bathing with chlorhexidine (n= 4488) had no effect on rates of a composite of central line–associated bloodstream infections, catheter-associated urinary tract infections, ventilator-associated pneumonia, and *Clostridium difficile* (2.86 per 1000 patient-days in treatment group vs 2.90/1000 in control group; rate difference, -0.04; 95% CI, -1.01 to 1.01; P=.95). This is a reversal of chlorhexidine bathing for hospitalized patients. | 2016. “No relevant studies were identified regarding the clinical effectiveness of CHG wipes for infection prevention in adult patients in acute care, or regarding the cost-effectiveness of the use of CHG wipes in acute or critical care settings; therefore, no summary can be provided for these questions.”^94^ | PubMed suggestion |
| 276 | Effect of Sedative Premedication on Patient Experience After General Anesthesia A Randomized Clinical Trial  Maurice-Szamburski et al. | Anesthesiology | 3/3/2015  JAMA | Premedication with lorazepam for anxiety prior to sedation is a common practice for patients undergoing general anesthesia.^95 96^ A randomized control trial compared pre-medication sedation with lorazepam (n=330), no pre-medication (n=319), and placebo (n=322) in patients undergoing surgery and found that there was no difference among the groups in self-reported improvement (patient satisfaction scores, 72 lorazepam vs 73 no premedication vs 71 placebo; P=.38). In fact, these patients had lower rates of early cognitive recovery (51% vs 71% vs 64%; P<.001) as well as prolonged times to extubation (17 minutes vs 12 min vs 13 min; P<.001). This is a reversal of lorazepam for reducing anxiety prior to sedation in patients undergoing general anesthesia. | None found | sedative premedication after general anesthesia |
| 277 | Surgical vs Nonsurgical Treatment of Adults With Displaced Fractures of the Proximal Humerus The PROFHER Randomized Clinical Trial  Rangan et al. | Orthopedic | 3/10/2015  JAMA | Proximal humeral fractures are common among people older than 65 years of age. Treatment for this type of fracture can be surgical or nonsurgical. Surgical treatment usually involves internal fixation or humeral head replacement.^97^ A randomized control trial compared surgical (n=114) versus nonsurgical (n=117) approaches to treating proximal humeral fractures and found that there were no differences in outcomes at 2 years following the fracture. Average Oxford Shoulder Scores was 39.07 points for the surgical group and 38.32 points for the nonsurgical group (difference, 0.75 points; 95% CI, -1.33 to 2.84 points; P=.48). This is a reversal of surgical treatment for proximal humeral fractures in adults. | 2015. Cochrane review. “There is high or moderate quality evidence that, compared with non-surgical treatment, surgery does not result in a better outcome at one and two years after injury for people with displaced proximal humeral fractures involving the humeral neck and is likely to result in a greater need for subsequent surgery.” ^98^ The review did not include the RCT. | PubMed suggestion |
| 278 | Effect of a Retrievable Inferior Vena Cava Filter Plus Anticoagulation vs Anticoagulation Alone on Risk of Recurrent Pulmonary Embolism A Randomized Clinical Trial  Mismetti et al. | Pulmonary | 4/28/2015  JAMA | For patients who present with acute venous thromboembolism, it is becoming increasingly popular for them to receive a retrievable inferior vena cava filter in addition to anticoagulation.^99 100^ A randomized control trial evaluated the efficacy and safety of this procedure in addition to anticoagulation (n=200) as opposed to anticoagulant therapy alone (n=199), and found that among people with severe acute pulmonary embolism, the addition of a retrievable inferior vena cava filter did not reduce their risk of symptomatic recurrent pulmonary embolism at 3 months (3 patients in both groups; RR with filter, 2.00; 95% CI, 0.51-7.89; p=.50). This is a reversal of the addition of retrievable inferior vena cava filters to anticoagulation therapy for patients with acute venous thromboembolism. | 2016. “Inferior vena cava filter in addition to anticoagulation was not associated with a reduction in the incidence of recurrent pulmonary embolism as compared with anticoagulation alone in patients with deep vein thrombosis in the short term.”^101^ | GoogleScholar - MA cited RCT |
| 279 | Effect of a 24-Month Physical Activity Intervention vs Health Education on Cognitive Outcomes in Sedentary Older Adults The LIFE Randomized Trial  Sink et al. | Public health/Preventive medicine | 8/25/2015  JAMA | Moderate-intensity physical activity that includes activities promoting flexibility and balance is recommended for older adults.^102^ This recommendation is based upon evidence that shows improvements in many health outcomes, including some evidence of improved cognition. In this RCT, a 24-month exercise program (n=735) was no better than a health education program (n=741) in improving cognitive function. At 24 months, mean Digit Symbol Coding scores were 46.26 points in the physical activity group vs 46.28 in the health education group (mean difference, -0.03; 96% CI, -0.20 to 0.24 words, P= .84). This is a reversal of physical activity interventions for improving cognitive functioning in sedentary older adults. | 2017. “Evidence from RCTs is limited and does not support that exercise reduces the risk of developing clinically important cognitive outcomes.”^103^ | GoogleScholar - MA cited RCT |
| 280 | Effect of Omega-3 Fatty Acids, Lutein/Zeaxanthin, or Other Nutrient Supplementation on Cognitive Function The AREDS2 Randomized Clinical Trial  Chew et al. | Public health/Preventive medicine | 8/25/2015  JAMA | Supplemental omega-3 fatty acids are thought to be beneficial for brain health because there is a high concentration of fatty acids in the brain, which helps in the flow of electrical signals.^104^ Researchers and physicians have promoted the intake of omega-3 fatty acids for the prevention of many health outcomes, including cognitive decline.^105^ In the AREDS2 trial, supplementation with omega-3 fatty acids (n= 1521) did not have an effect on cognitive function compared to no supplementation (n=1503) in patients who were at risk for developing late age-related macular degeneration. There was no difference in change in cognitive functioning scores between the supplement and no supplement group (-0.19 vs -0.18; difference in yearly change, -0.03; 995 CI, -0.20 to 0.13; P=.63). This is a reversal of omega 3 fatty acids for protecting cognitive functioning. | 2017. AHRQ report. “ Low-strength evidence suggests omega-3 fatty acids and ginkgo biloba did not reduce CATD incidence or improve cognitive performance in adults with normal cognition.” ^106^ | PubMed suggestion |
| 281 | Rehabilitation After Immobilization for Ankle Fracture The EXACT Randomized Clinical Trial  Moseley et al. | Orthopedic | 10/6/2015  JAMA | It is often recommended that people who have fractured their ankle undergo a supervised exercise program of some kind after the immobilization of the ankle fracture is complete, but the effects of this type of rehabilitation are not clearly established.^107^ This randomized control trial did not find any additional benefits for people who had isolated and uncomplicated ankle fracture who underwent a supervised exercise program (n=106) as compared with those who only received advice (n=108) following the removal of immobilization. At 3 months, mean difference in activity limitation score was 0.4 (95% CI, -3.9 to 3.2) and mean difference in quality of life was -0.04 (95% CI, -0.09 to 0.01). This is a reversal of rehabilitation after immobilization for ankle fracture. | None found | rehabilitation after ankle fracture |
| 282 | Naproxen With Cyclobenzaprine, Oxycodone/Acetaminophen, or Placebo for Treating Acute Low Back Pain A Randomized Clinical Trial  Friedman et al. | Orthopedic | 10/20/2015  JAMA | Low back pain is very common and treatment protocols in emergency departments are heterogeneous, ranging from medication with nonsteroidal anti-inflammatory drugs (NSAIDs), skeletal muscle relaxants, and opioids.^108^ A randomized study found that among patients with acute, nontraumatic, nonradicular low back pain presenting in the emergency room, adding cyclobenzaprine (n=108), oxycodone plus acetaminophen (n=108), or placebo (n=107) to naproxen did nothing to improve functional outcomes or pain for these patients one week later. Between-group difference in mean Roland-Morris Disability Questionnaire improvement for cyclobenzaprine vs placebo was 0.3 (98.3% CI, −2.6 to 3.2; P = .77), for oxycodone/acetaminophen vs placebo, 1.3 (98.3% CI, −1.5 to 4.1; P = .28), and for oxycodone/acetaminophen vs cyclobenzaprine, 0.9 (98.3% CI, −2.1 to 3.9; P = .45). This is a reversal of cyclobenzaprine and oxycodone/acetaminophen for treating acute low back pain in emergency departments in addition to naproxen. | None found | pharmacologic treatment for low back pain |
| 283 | Autologous Hematopoetic Stem Cell Transplantation for Refractory Crohn Disease A Randomized Clinical Trial  Hawkey et al. | Gastroenterology/Hepatology | 12/15/2015  JAMA | Crohn disease is a chronic relapsing inflammatory condition of the gastrointestinal tract that can result in lifelong ill health, impaired quality of life, and reduced life expectancy. Immunosuppressive drugs are standard of care for Crohn disease, but some patients do not respond or become unresponsive to treatment. Hematopoietic stem cell transplantation (HSCT) might have a role to play in some of these treatment-resistant cases. Case reports and series describe long-term treatment-free disease regression with autologous and allogeneic HSCT in some but not all patients with Crohn disease and in other patients with conditions that have autoimmune pathology, such as systemic sclerosis.^109^ This study found that among adult patients with refractory Crohn disease not amenable to surgery who had impaired quality of life, HSCT (n=21), compared with conventional therapy (n=21), did not result in a statistically significant improvement in sustained disease remission at 1 year (remission in 2 patients in the HSCT group vs 1 patient in control group; absolute difference, 4.2%; 95% CI, -14.2% to 22.6%; P=.60). The HSCT group had 76 adverse events compared to 38 in the control group. This is a reversal of autologous HSCT for refractory Crohn disease. | A 2017 SR/MA concluded that hematopoietic stem cell transplantation led to high remission rates. It should be noted that this SR was not limited to RCTs (included case-series), and the information they used for this RCT did not appear to be correct.^110^ | PubMed suggestion |
| 284 | Effect of Prehospital Induction of Mild Hypothermia on Survival and Neurological Status Among Adults With Cardiac Arrest A Randomized Clinical Trial  Kim et al. | Cardiovascular | 1/1/2014  JAMA | Mild therapeutic hypothermia was first used in the treatment of patients with cardiac arrest because people thought this was a way to reduce cerebral oxygen demand, which could improve neurological outcomes in these patients. This practice became more common and was incorporated into several sets of guidelines after several positive randomized controlled trials, some encouraging the cooling as soon as spontaneous circulation returns. ^111-113^ Some hospitals even incorporated pre-hospital cooling into their treatment protocol.^114^ This randomized controlled study compared therapeutic hypothermia with (n=292) and without (n=396) ventricular fibrillation to no hypothermia with (n=291) and without (n=380) ventricular fibrillation and found that the addition of therapeutic hypothermia to standard care did not improve survival or neurological status in cardiac patients. Survival to hospital discharge was similar among the intervention and control groups among patients with VF (62.7% [95% CI, 57.0%-68.0%] vs 64.3% [95% CI, 58.6%-69.5%], respectively; P = .69) and among patients without VF (19.2% [95% CI, 15.6%-23.4%] vs 16.3% [95% CI, 12.9%-20.4%], respectively; P = .30). This is a reversal of mild hypothermia for cardiac arrest with or without ventricular fibrillation. | 2016. Cochrane review. “Currently, there is no convincing evidence to clearly delineate beneficial or harmful effects of pre-hospital induction of cooling in comparison to in-hospital induction of cooling. This conclusion is based on very low quality evidence.”^115^ | PubMed suggestion |
| 285 | Mechanical Chest Compressions and Simultaneous Defibrillation vs Conventional Cardiopulmonary Resuscitation in Out-of-Hospital Cardiac Arrest The LINC Randomized Trial  Rubertsson et al. | Critical care | 01/01/2014  JAMA | Cardiopulmonary resuscitation (CPR) is practiced on cardiac arrest patients to restore spontaneous circulation, but there are drawbacks with the procedure that cause it to be less effective,^116^ such as human limitations of consistency, strength, and duration.^117^ Therefore, mechanical depression devices have been designed to provide more effective means of delivering CPR. LUCAS is a chest compression device that was in use since 2003 for treating patients with cardiac arrest.^118^ This study found that mechanical chest compressions with LUCAS (n=1300) had no significant effect on survival compared to manual CPR (n=1289) in patients with out-of-hospital cardiac arrest. Four-hour survival occurred for 23.6% of patients with mechanical CPR and 23.7% with manual CPR (risk difference, -0.05%, 95% CI, -3.3% to 3.2%; P>.99). This is a reversal of mechanical CPR in out-of-hospital cardiac arrest. | 2016. "The ability to achieve ROSC with mechanical devise was inferior to manual chest compression during resuscitation. The use of mechanical chest compression cannot be recommended as a replacement for manual CPR, but rather a supplemental treatment in an overall strategy for treating CA patients."^119^ | PubMed suggestion |
| 286 | Web-Based Alcohol Screening and Brief Intervention for University Students A Randomized Trial  Kypri et al. | Public health/Preventive medicine | 3/26/2014  JAMA | Alcohol consumption is common among young adults and college students. Unhealthy alcohol consumption can lead to adverse outcomes, and web-based alcohol screenings and brief intervention programs were therefore designed to promote healthy alcohol use. While these programs have not been implemented on a national scale in any country, they have been implemented on smaller, local scales. Several commercial programs are available for college students and many others have been tested in various academic settings.^120-124^ In this study, web-based alcohol screening and a brief intervention program (n=1437) did not lead to better alcohol consumption outcomes compared to screening only (n=1413) for students who screened positive for alcohol abuse. The intervention group did not drink less often compared to control (RR, 0.95; 99.17% CI, 0.88-1.03; P=.08) or less overall (RR, 0.95; 99.17% CI, 0.81-1.10; P.33) and academic problem scores were not lower (RR, 0.91; 99.17% CI, 0.76-1.08; P=.14). Effects on risks of binge drinking (OR, 0.84; 99.17% CI, 0.67-1.05; P=.04) and heavy drinking (OR, 0.77; 99.17% CI, 0.56-1.05; P = .03) were not statistically significant. This is a reversal of web-based screening plus brief interventions for reducing unhealthy alcohol use in university students. | None found | PubMed suggestion |
| 287 | Effect of PET Before Liver Resection on Surgical Management for Colorectal Adenocarcinoma Metastases A Randomized Clinical Trial  Moulton et al. | Oncology | 5/14/2014 | About 50% of people with colorectal cancer will develop liver metastases.^125 126^ Positron emission tomography (PET) and computed tomography (CT) as a combined procedure can detect occult metastases that are not detected during surgical treatment of colorectal cancer. Their use became common after several smaller studies were published.^126-128^ In this study, use of PET in combination with CT (n=263) did not lead to changes in surgical management in patients who had a resectable colorectal liver metastases when compared to patients who received no PET-CT (n=134). After a median follow-up of 36 months, survival rate did not differ between the two groups (hazard ratio, 0.86; 95% CI, 0.60-1.21; P = .38). This is a reversal of the addition of PET to CT before liver resection for colorectal adenocarcinomas. | None found | PET in combination with CT for liver resection in colorectal cancer |
| 288 | Effect of Physical Therapy on Pain and Function in Patients With Hip Osteoarthritis A Randomized Clinical Trial  Bennell et al. | Orthopedic | 5/21/2014  JAMA | Hip osteoarthritis is a common condition, and guideline-recommended treatment advocates the use of physiotherapy.^129^ Patients with hip osteoarthritis in this study who were treated with physical therapy (n=49) did not have improvements in pain or function, as compared to sham treatment (n=53). At week 13, mean difference in Visual Analog pain scores was 6.9 mm, favoring sham (95% CI, -3.9 to 17.7) and function scores were also similar and favoring sham treatment with a mean difference of 1.4 units (95% CI, -3.8 to 6.5). This is a reversal of physical therapy for reducing pain and improving functionality in patients with hip osteoarthritis. | None found | GoogleSchoolar - RCT cited MA |
| 289 | Effect of Endoscopic Sphincterotomy for Suspected Sphincter of Oddi Dysfunction on Pain-Related Disability Following Cholecystectomy The EPISOD Randomized Clinical Trial  Cotton et al. | Gastroenterology/Hepatology | 5/28/2014  JAMA | Sphincter of Oddi manometry is the gold standard for diagnosing sphincter of Oddi dysfunction and is commonly used to determine the etiology of pancreatitis in patients who have undergone endoscopic retrograde cholangiopancreatography (ERCP).^130 131^ Sphincterotomies are commonly used in sphincter of Oddi manometry to remove stones or to relieve scarring or spasms of the sphincter.^132 133^ In this study, patients with abdominal pain after cholecystectomy who had undergone sphincterotomy (n=141) had worse risk of pain compared to sham treatment (n=73). Success treatment (less than 6 days of disability due to pain in the prior 90 days with no narcotic use) occurred in 37% of patients in the sham group and 23% in the sphincterotomy group (adjusted risk difference, -15.6%; 95% CI, -28.0% to -3.3%; P=.01). This is a reversal of sphincterotomy for abdominal pain after cholecystectomy undergoing ERCP with manometry. | There is only one published Cochrane review on this topic and it is from 2001. It concluded that there was insufficient data to make a recommendation (2 studies were included in the review).^134^ There was one SR/MA of RCTs and observational studies that looked at this topic, but did not include this study in its review, even though the study was published 2 years before the SR/MA. It found that there was no improvement in pancreatitis with endoscopic sphincterotomy in patients who received endoscopic retrograde cholangiopancreatography and stent.^135^ | GoogleSchoolar - RCT cited MA |
| 290 | Effect of Postoperative Antibiotic Administration on Postoperative Infection Following Cholecystectomy for Acute Calculous Cholecystitis A Randomized Clinical Trial  Regimbeau et al. | Gastroenterology/Hepatology | 7/9/2014  JAMA | Antibiotics are often given to a patient after cholecystectomy surgery to prevent subsequent infections, but this treatment has not been thoroughly tested as to whether or not it is necessary.^136 137^ In this study of patients with mild or moderate calculous cholecystitis, the continuation of the antibiotic regimen, amoxicillin plus clavulanic acid, after surgery (n=207) did not lead to lower rates of post-operative infection, compared to no antibiotics post-surgery (n=207). At the 4-week follow-up visit, rates of infection were 17% for the no-treatment group and 15% for the antibiotic group (absolute difference, 1.93%; 95% CI, -8.98% to 5.12%). This is a reversal of antibiotic administration to prevent postoperative infection following a cholecystectomy. | None found | GoogleSchoolar - RCT cited MA |
| 291 | Effects of Hydroxychloroquine on Symptomatic Improvement in Primary Sjögren Syndrome The JOQUER Randomized Clinical Trial  Gottenberg et al. | Rheumatology | 7/16/2014  JAMA | Hydroxychloroquine and corticosteroids are commonly prescribed for patients with Sjögren Syndrome, but data on their efficacy are sparse and conflicting.^138-141^ In this trial, patients with Sjögren Syndrome treated with hydroxychloroquine (n=56) did not have improvements in eye dryness, pain, or fatigue, compared to patients who received placebo (n=64). Reduction of symptoms by 30% occurred in 17.9% of patients in the hydochloroquine group and 17.2% in the placebo group (OR, 1.01; 95% CI, 0.37-2.78; P=.98). This is a reversal of hydroxychloroquine for treating symptoms of primary Sjögren syndrome. | 2017. “This systematic review showed that there is no significant difference between HCQ [hydroxychloroquine] and placebo in the treatment of dry mouth and dry eye in pSS [primary Sjögren Syndrome.]”^142^ | PubMed suggestion |
| 292 | Brief Intervention for Problem Drug Use in Safety-Net Primary Care Settings A Randomized Clinical Trial  Roy-Byrne et al. | Public health/Preventive medicine | 8/6/2014  JAMA | Programs with screening, brief intervention, and treatment for drug dependency have been widely adopted in medical settings, even though evidence for their effectiveness has been limited.^143 144^ In this trial, there were no positive improvements in drug use in patients with drug use for patients who received a one-time brief intervention plus booster call (n=435) compared to usual care (n=433). Differences in mean days of drug use at 3 months postintervention were 11.87 for the treatment group and 9.84 for the control group (between group difference, β=0.89; 95% CI, -0.49 to 2.26). This is a reversal of one-time brief interventions with telephone booster in a primary care setting for drug use intervention. | None found | brief intervention for drug use (with our without "safety net”) |
| 293 | Screening and Brief Intervention for Drug Use in Primary Care The ASPIRE Randomized Clinical Trial  Saitz et al. | Public health/Preventive medicine | 8/6/2014  JAMA | Programs with screening, brief intervention, and treatment for drug dependency have been widely adopted in medical settings, even though evidence for their effectiveness has been limited.^143 144^ This study randomized primary care patients identified as drug users by screening to one of 3 groups: brief negotiated interviewing (BNI), motivational interviewing (MOTIV), and no brief intervention. Mean days of drug use over a 30-day period was 11 for the BNI group, 12 for MOTIV group, and 12 for control group (incidence RR, 1.05; 95% CI, 0.84-1.32; P = .81 for both comparisons vs no brief intervention). This is a reversal of brief interventions for drug use in the primary care setting. | None found |  |
| 294 | Acupuncture for Chronic Knee Pain A Randomized Clinical Trial  Hinman et al. | Orthopedic | 10/1/2014  JAMA | Chronic knee pain is common in older adults and as people age. Acupuncture is a popular complementary and alternative medicine treatment for treating pain^145^ and has been shown to be effective in one meta-analysis.^146^ In this trial, patients with chronic knee pain, >50 years of age, were randomized to needle acupuncture (n=70), laser acupuncture (n=71), sham acupuncture (n=70), and no acupuncture (n=71). At 12 weeks, neither needle nor laser acupuncture improved knee pain (mean difference; −0.4 units; 95% CI, −1.2 to 0.4, and −0.1; 95% CI, −0.9 to 0.7, respectively) or physical function (−1.7; 95% CI, −6.1 to 2.6, and 0.5; 95% CI, −3.4 to 4.4, respectively) compared with sham at 12 weeks.  This is a reversal of acupuncture for treating patients with chronic knee pain. | None found |  |
| 295 | Effect of Screening for Coronary Artery Disease Using CT Angiography on Mortality and Cardiac Events in High-Risk Patients With Diabetes The FACTOR-64 Randomized Clinical Trial  Muhlestein et al. | Endocrinology, Diabetes, and Metabolism | 12/3/2014  JAMA | People with diabetes have a high risk of developing severe coronary artery disease (CAD). CAD is the most common cause of death among patients with diabetes.^147^ Screening for CAD may help identify individuals at high risk of CAD who may benefit from management and treatment. Coronary computed tomography angiography (CCTA) was a common form of screening being used for CAD screening.^148^ However, in this trial, there were no differences in a composite of all-cause mortality, nonfatal myocardial infarction, or unstable angina requiring hospitalization between patients with diabetes who were screened using CCTA (n=395) and those who were not screened (n=504). At 4 years follow up, the composite outcome occurred in 6.2% from the CCTA group vs 7.6% from no treatment group (HR, 0.80; 95% CI, 0.49-1.32; P=.38). This is a reversal of using CCTA to screen for CAD in patients with diabetes. | 2017. “Current available data do not support screening [with CCTA] for coronary artery disease in patients with type 2 diabetes for preventing fatal events.”^149^ | GoogleSchoolar - RCT cited MA |
| 296 | Effect of Fenoldopam on Use of Renal Replacement Therapy Among Patients With Acute Kidney Injury After Cardiac Surgery A Randomized Clinical Trial  Bove et al. | Nephrology | 12/3/2014  JAMA | Acute kidney injury (AKI) is common after cardiac surgery and is an independent predictor of higher mortality among patients who received cardiac surgery.^150^ Fenoldopam is a therapy that has been used for treatment of AKI in this setting and is considered part of standard care.^151 152^ In this trial, fenoldopam (n=338) did not reduce the rate of renal replacement therapy compared to placebo (n=329) in patients with acute kidney injury after cardiac surgery. This study ended early for futility. Twenty percent of patients in the fenoldopam and 18% of patients in the placebo group received renal replacement therapy (P=.47). This is a reversal of fenoldopam for treating AKI after cardiac surgery. | 2015. “In this analysis, peri-operative treatment with fenoldopam was associated with a significant reduction in post-operative AKI but it had no impact on renal replacement therapy or hospital mortality.” ^153^ This review did not include the RCT. | GoogleSchoolar - RCT cited MA |
| 297 | Low-Dose Aspirin for Primary Prevention of Cardiovascular Events in Japanese Patients 60 Years or Older With Atherosclerotic Risk Factors A Randomized Clinical Trial  Ikeda et al. | Cardiovascular | 12/17/2014  JAMA | Based on several studies showing a beneficial effect of aspirin, the 2002 United States Preventive Services Task Force found "good" evidence that aspirin decreases coronary heart disease in high-risk adults.^154^ Other organizations, such as the American Diabetes Association, American Heart Association, and the European Society of Cardiology also recommend aspirin prophylaxis for certain high-risk groups.^154^ Recommendations by the Joint Research Committee for Cardiac Disease in Japan advises physicians to “consider the use of aspirin for those with risk factors”.^155^ Although aspirin use for preventing cardiovascular events is not widespread in Japan, US guidelines are often adapted in other countries without considering their appropriateness in non-US populations. This study on low-dose aspirin use in Japanese men, 60 years or older, found that, compared to no aspirin (n=7244), daily low-dose aspirin (n=7220) did not lead to lower rates of a composite of death from cardiovascular causes, nonfatal stroke, or nonfatal myocardial infarction (2.77% in aspirin group vs 2.96% in no-aspirin group; HR, 0.94; 95% CI, 0.77-1.15; P=.54). This is a reversal of low-dose aspirin for primary prevention of cardiovascular events in Japanese patients with atherosclerotic risk factors. | 2017. “Evidence for aspirin in primary prevention is heterogeneous and limited by rare events and few credible subgroup analyses… The beneficial effect of aspirin for the primary prevention of CVD is modest and occurs at doses of 100 mg or less per day.^156^ | GoogleSchoolar - RCT cited MA |
| 298 | Effect of Maintenance Tocolysis With Nifedipine in Threatened Preterm Labor on Perinatal Outcomes A Randomized Controlled Trial  Roos et al. | Obstetrics/Gynecology | 1/2/2013  JAMA | Preterm labor is relatively common occurrence that can result in high health care costs. Tocolytics are often used to prevent uterine contractions but none are recommended above any others, in general.^157^ Nifedipine is one such commonly used tocolytic agent.^158^ This paper showed that In patients with threatened preterm labor, treatment with nifedipine (n=201) did not result in a statistically significant reduction in adverse perinatal outcomes when compared with placebo (n=205). Twenty-four of 201 participants (11.9%) in the nifedipine group vs 28/205 (13.7%) in the placebo group experience a composite of perinatal death, chronic lung disease, neonatal sepsis, intraventricular hemorrhage >grade 2, periventricular leukomalacia >grade 1, or necrotizing enterocolitis (RR, 0.87; 95% CI, 0.53-1.45). This is a reversal of nifedipine for maintaining tocolysis in threatened preterm labor. | 2013. Cochrane review. “Based on the current available evidence, maintenance treatment with a calcium channel blocker after threatened preterm labour does not prevent preterm birth or improve maternal or infant outcomes.” ^159^ | PubMed suggestion |
| 299 | Effect of Corticosteroid Injection, Physiotherapy, or Both on Clinical Outcomes in Patients With Unilateral Lateral Epicondylalgia A Randomized Controlled Trial  Coombes et al. | Orthopedic | 2/6/2013  JAMA | Corticosteroid injection is a common treatment for patients with lateral epicondylalgia,^160^ but because of the high recurrence rates, alternative treatments, such as physiotherapy, are also used. A conservative treatment, consisting of physiotherapy, is the recommended for the management of this condition.^161^ In this randomized controlled trial, patients with unilateral lateral epicondylalgia were randomized to corticosteroid injection (n=43), corticosteroid injection plus physiotherapy (n=39), placebo injection (n=40), or placebo injection plus physiotherapy n=41). Corticosteroid injections had worse rates of complete recovery or much recovery at 83% compared to placebo at 96% (RR, 0.86; 99% CI, 0.75-0.99; P=.01) and greater 1-year recurrence (54% vs 12%; RR, 0.23; 99% CI, 0.10-0.51; P<.001). Physiotherapy rates of recovery were 91% vs 88% for no physiotherapy (RR, 1.04; 99% CI, 0.90-1.19; P=.56) and recurrence was also similar at 29% vs 38% (RR, 1.31; 99% Ci, 0.73-2.35; P=.25). This is a reversal of corticosteroid injection and physiotherapy for patients with unilateral lateral epicondylalgia. | 2015. “Pooled data from RCTs indicate a lack of intermediate- to long-term clinical benefit after nonsurgical treatment of lateral epicondylitis compared with observation only or placebo.” ^162^ | GoogleSchoolar - RCT cited MA |
| 300 | Effect of Early vs Late Tracheostomy Placement on Survival in Patients Receiving Mechanical Ventilation The TracMan Randomized Trial  Young et al. | Critical care | 5/22/2013  JAMA | Tracheostomies are commonly performed on patients who require prolonged ventilation. Tracheostomies appear to increase patient comfort and lead to faster time to weaning from mechanical ventilation.^163^ Survey data indicate that there are wide variations in time to tracheostomy – with 13% of respondents initiating tracheostomy within 2 days and a median time of 10 to 11 days.^164^ Retrospective data suggested that earlier tracheostomy led to faster recovery from mechanical ventilation, shorter hospital stay, and a reduction in hospital mortality. In this trial, earlier tracheostomy would lead to faster recovery from mechanical ventilation, but in this trial earlier tracheostomy (within 4 days, n=451)) did not lead to improvements in 30-day mortality compared to late tracheostomy (after 10 days if still indicated, n=448). All-cause mortality after 30 days was 30.8% for the early group and 31.5% in the late group (absolute RR, 0.7%; 95% CI, -5.4% to 6.7%; P=.89). This is a reversal of early tracheostomy for patients receiving mechanical ventilation. | 2014. “Among the patients requiring prolonged MV [mechanical ventilation], ET [early tracheostomy] showed no significant difference in clinical outcomes compared to that of the LT/PI [late tracheostomy/prolonged intubation] group.”^165^ | PubMed suggestion |
| 301 | Early Parenteral Nutrition in Critically Ill Patients With Short-term Relative Contraindications to Early Enteral Nutrition A Randomized Controlled Trial  Doig et al. | Critical care | 5/22/2013  JAMA | Parenteral nutrition (PN) can provide nutrition for critically ill patients who cannot receive nutrition enterally. However, the timing of when to begin PN has been debated. Guidelines such as the European Society for Clinical Nutrition and Metabolism recommend starting PN within 24 to 48 hours of ICU admission if enteral nutrition is contraindicatedr,^166 167^ rather than waiting at least a week, as recommended by the American Society for Parenteral and Enteral Nutrition .^168^ In this trial, early PN (n=678) did not improve 60-day mortality compared to standard care (n=680). Day-60 mortality was 22.8% for standard care vs 21.5% for early PN (risk difference, -1.26%; 95% CI, -6.6 to 4.1; P=.60). This is a reversal of early PN for critically ill patients with short-term relative contraindications to early enteral nutrition. | 2015. “Overall, this meta-analysis from RCTs indicates that provision of ePN [early parenteral nutrition] within 24-48 hours has no benefit on the survival rate in critically ill patients.” ^169^ | PubMed suggestion |
| 302 | Effect of Soy Protein Isolate Supplementation on Biochemical Recurrence of Prostate Cancer After Radical Prostatectomy A Randomized Trial  Bosland et al. | Oncology | 7/10/2013  JAMA | Observational studies have shown an association between high soy intake and low prevalence of prostate cancer.^170^ The belief that soy is protective in preventing prostate cancer seems biologically plausible in that soy foods contain isoflavones, which contain antioxidant and anticancer properties.^171^ Sales of soy increased from $300 million in 1992 to $4 billion in 2008.^172^ Several companies were marketing soy isoflavones to help prevent prostate and breast cancer.^173^ In this study, soy protein isolate supplements (n=78) did not reduce recurrence of prostate cancer compared to placebo (n=73) in patients after radical prostatectomy. The rate of biochemical recurrence of prostate cancer was 27.3% for participants in the treatment group and 29.5% in the placebo group (HR, 0.96; 95% CI, 0.53-1.72; P=.89). This is a reversal of soy protein isolate supplementation for preventing recurrence of prostate cancer after radical prostatectomy. | None found | "soy protein isolate" and prostate cancer |
| 303 | Menopausal Hormone Therapy and Health Outcomes During the Intervention and Extended Poststopping Phases of the Women’s Health Initiative Randomized Trials  Manson et al. | Obstetrics/Gynecology | 10/2/2013  JAMA | Postmenopausal hormone replacement therapy (HRT) was initially used in the 1940s as a way to delay age-related health outcomes, but in the 1970’s, studies began to emerge showing that the use of HRT, specifically unopposed estrogen, was associated with endometrial cancer. Progesterone was thought to oppose the effects of estrogen and mitigate the excess risk of cancer, so women began to take them again. By the 1990s, HRTs were the most commonly prescribed medications.^174^ The Women’s Health Initiative investigated the effects of HRT in postmenopausal women compared to placebo. This paper is an overview of the many health effects of HRT and found that there is a complex pattern of risks and benefits. The authors concluded that HRT is not an appropriate or recommended intervention for the prevention of chronic disease in postmenopausal women. | 2015. “The current evidence suggests that MHT [menopausal hormone therapy] does not affect the risk of death from all causes, cardiac death and death from stroke or cancer.”^175^ Another SR/MA (2016) did not find any cardiovascular benefit to hormone therapy.^176^ | GoogleSchoolar - RCT cited MA |
| 304 | Universal Glove and Gown Use and Acquisition of Antibiotic-Resistant Bacteria in the ICU A Randomized Trial  Harris et al. | Critical care | 10/16/2013  JAMA | The emergence of antibiotic-resistant bacteria has become a serious public health issue. To help prevent the spread of these organisms, policies recommending contact precautions (e.g. gloves and gowns) were made by the Centers for Disease Control and Prevention.^177^ In this trial, intensive care units (ICUs) were randomized to usual care of ICUs (n of ICUs=10) or a protocol where all health care workers are required to wear gloves and gowns for all patient contact (intervention ICUs; n of ICUs=10). There was no difference in the acquisition of methicillin-resistant *Staphylococcus aureus* or vancomycin-resistant *Enterococcus* between ICUs that had universal glove and gown use and those that did not (difference, −1.71 acquisitions per 1000 person-days, 95% CI, −6.15 to 2.73; P = .57). This is a reversal of requiring that all health care workers in ICUs wear gloves and gowns for all patient contact and when entering a patient room. | 2014. “Contact precautions did not significantly reduce the VRE acquisition rate.” ^178^ This review did not include the RCT. | gloves and gowns for antibiotic resistant bacteria in icu |
| 305 | Effect of Risk-Reduction Counseling With Rapid HIV Testing on Risk of Acquiring Sexually Transmitted Infections The AWARE Randomized Clinical Trial  Metsch et al. | Infectious | 10/23-30/2013  JAMA | In 1999, the CDC issued recommendations that encouraged counseling and screening to prevent human immunodeficiency virus (HIV) incidence and the impact of existing HIV.^179^ In 2006, these recommendations were changed, and the recommendation for counseling at the time of testing were relaxed,^180^ but some states still recommend counseling in addition to HIV testing.^181^ In the AWARE trial, rapid HIV testing with patient-centered risk-reduction counseling (n=2505) did not reduce the incidence of sexually transmitted disease (STD) among patients in STD clinics compared to receiving information with rapid testing (n=2507). Rate of STDs was 12.3% in the counseling group and 11.1% in the information-only group (adjusted RR, 1.12; 95% CI, 0.94=1.33). This is a reversal of implementing risk-reduction counseling compared to receiving information during rapid HIV testing. | None found | risk reduction counseling and sexually transmitted infections |
| 306 | Effects of Fluid Resuscitation With Colloids vs Crystalloids on Mortality in Critically Ill Patients Presenting With Hypovolemic Shock The CRISTAL Randomized Trial  Annane et al. | Critical care | 11/6/2013  JAMA | Colloid solutions, such as albumin, are widely used to increase interstitial fluid volume because of their ability to increase oncotic pressure within the interstitium, but they are more expensive than other options (e.g. hypertonic saline) and may not provide any additional benefits.^182^ In the CRISTAL trial, colloidal solutions (n=1414) given to patients with hypovolemic shock did not improve 28-day mortality, when compared to patients treated with crystalloid solutions (n=1443). Rate of death at 28 days was 25.4% in the colloids group vs 27.0% in the crystalloids group (RR, 0.96; 95% CI, 0.88-1.04; P=.26). This is a reversal of colloids for critically ill patients presenting with hypovolemic shock. | 2014. “The present meta-analysis did not demonstrate significant advantage of using albumin-containing fluids for resuscitation in patients with sepsis of any severity… crystalloids should be the first choice for fluid resuscitation in septic patients.” ^183^ | PubMed suggestion |
| 307 | Effect of Communication Skills Training for Residents and Nurse Practitioners on Quality of Communication With Patients With Serious Illness A Randomized Trial  Curtis et al. | Critical care | 12/4/2013  JAMA | Discussing end-of-life care can be an uncomfortable but sometimes unavoidable component of patient care. Good communication skills are necessary for health care providers in these types of circumstances. Several types of training are available for health care providers, including one by the American Medical Association.^184 185^ In this trial, training medical professionals in simulation-based communication skills (n=78) did not result in improved patient-reported of communication compared to usual education (n=116). Quality of communication questionnaire scores were similar for patients (difference, 0.4 points; 95% CI, -0.1 to 0.9; P=.15) and for families (difference, 0.1; 95% CI, -0.8 to 1.0; P=.81). This is a reversal of simulation-based communication training in place of usual education on end-of-life care for internal medicine and nurse practitioner trainees. | 2017. “Meta-analysis showed no effect on patient outcomes.“^186^ | GoogleSchoolar - RCT cited MA |
| 308 | Effect of Nortriptyline on Symptoms of Idiopathic Gastroparesis The NORIG Randomized Clinical Trial  Parkman et al. | Gastroenterology/Hepatology | 12/25/2013  JAMA | There are few used or approved drugs for increasing gastric emptying in gastroparesis, and administration of these drugs is limited due to serious adverse effects.^187^ Some symptoms of gastroparesis are due to neuropathic events and have led some to theorize that tricyclic antidepressants, a class of neuromodulating drugs, may be effective in treating the condition. Several organizations have recommended tricyclic antidepressants for the antiemetic treatment of gastroparesis.^188 189^ “In clinical practice, tricyclic antidepressants (TCAs) in low doses are used as neuromodulators for treatment of nausea, vomiting, and abdominal pain in patients with gastroparesis.”^190^ In this trial, nortriptyline (n=65), a tricyclic antidepressant, did not improve symptoms of gastroparesis compared to placebo (n=65). Decrease in baseline Gastroparesis Cardinal Symptom Index scores of at least 50% on 2 consecutive assessments was similar between the nortriptyline group (23%; 95% CI, 14%-35%) and placebo group (21%; 95% CI, 12%-34%; P=.86). This is a reversal of nortriptyline for treating symptoms of idiopathic gastroparesis. | None found | There was no SR/MA on this specific topic. |

1. Jaber S, Amraoui J, Lefrant J-Y, et al. Clinical practice and risk factors for immediate complications of endotracheal intubation in the intensive care unit: a prospective, multiple-center study. *Critical care medicine* 2006;34(9):2355-61.

2. Chemsian R, Bhananker S, Ramaiah R. Videolaryngoscopy. *International Journal of Critical Illness and Injury Science* 2014;4(1):35-41. doi: 10.4103/2229-5151.128011

3. Huang H-B, Peng J-M, Xu B, et al. Video laryngoscopy for endotracheal intubation of critically ill adults: a systemic review and meta-analysis. *Chest* 2017;152(3):510-17.

4. Canonica GW, Cox L, Pawankar R, et al. Sublingual immunotherapy: World Allergy Organization position paper 2013 update. *World Allergy Organization Journal* 2014;7(1):1.

5. Kristiansen M, Dhami S, Netuveli G, et al. Allergen immunotherapy for the prevention of allergy: A systematic review and meta‐analysis. *Pediatric Allergy and Immunology* 2017;28(1):18-29.

6. Yaffe K, Lui LY, Zmuda J, et al. Sex hormones and cognitive function in older men. *Journal of the American Geriatrics Society* 2002;50(4):707-12.

7. Cherrier MM, Asthana S, Plymate S, et al. Testosterone supplementation improves spatial and verbal memory in healthy older men. *Neurology* 2001;57(1):80-88.

8. Margo K, Winn R. Testosterone treatments: why, when, and how? *American family physician* 2006;73(9)

9. Bolliger D, Szlam F, Molinaro R, et al. Finding the optimal concentration range for fibrinogen replacement after severe haemodilution: an in vitro model. *British journal of anaesthesia* 2009;102(6):793-99.

10. Solomon C, Pichlmaier U, Schoechl H, et al. Recovery of fibrinogen after administration of fibrinogen concentrate to patients with severe bleeding after cardiopulmonary bypass surgery. *British journal of anaesthesia* 2010;104(5):555-62.

11. Fominskiy E, Nepomniashchikh VA, Lomivorotov VV, et al. Efficacy and safety of fibrinogen concentrate in surgical patients: a meta-analysis of randomized controlled trials. *Journal of cardiothoracic and vascular anesthesia* 2016;30(5):1196-204.

12. Naylor J, Harmer A, Fransen M, et al. Status of physiotherapy rehabilitation after total knee replacement in Australia. *Physiotherapy research international* 2006;11(1):35-47.

13. Pezzin LE, Roberts BA, Miao H, et al. Regulatory policies, the" 75% rule," and post-acute care discharge setting. *American journal of physical medicine & rehabilitation* 2011;90(11):954-58.

14. Li D, Yang Z, Kang P, et al. Home-Based Compared with Hospital-Based Rehabilitation Program for Patients Undergoing Total Knee Arthroplasty for Osteoarthritis: A Systematic Review and Meta-analysis of Randomized Controlled Trials. *American journal of physical medicine & rehabilitation* 2017;96(6):440-47.

15. INDIANA DEPARTMENT OF ENVIRONMENTAL MANAGEMENT: Integrated Pest Management OoCS. IDEM Fact Sheet, 2010.

16. Action MBaLSaHRi. The Role of Pest Control in Effective Asthma Management:

A Business Case. In: Commission eARCoNEftBPH, ed., 2009.

17. Brozek G, Lawson J, Szumilas D, et al. Increasing prevalence of asthma, respiratory symptoms, and allergic diseases: Four repeated surveys from 1993-2014. *Respiratory medicine* 2015;109(8):982-90.

18. Short J. Use of dexmedetomidine for primary sedation in a general intensive care unit. *Critical care nurse* 2010;30(1):29-38.

19. Jüni P, Hari R, Rutjes AW, et al. Intra‐articular corticosteroid for knee osteoarthritis. *The Cochrane Library* 2015

20. Jüni P, Hari R, Rutjes AWS, et al. Intra-articular corticosteroid for knee osteoarthritis. *Cochrane Database of Systematic Reviews* 2015(10) doi: 10.1002/14651858.CD005328.pub3

21. Group SoUNW. Standardization of uveitis nomenclature for reporting clinical data. Results of the First International Workshop. *American journal of ophthalmology* 2005;140(3):509-16.

22. Callanan DG, Jaffe GJ, Martin DF, et al. Treatment of posterior uveitis with a fluocinolone acetonide implant: three-year clinical trial results. *Archives of ophthalmology (Chicago, Ill: 1960)* 2008;126(9):1191-201.

23. Legro RS, Brzyski RG, Diamond MP, et al. Letrozole versus clomiphene for infertility in the polycystic ovary syndrome. *New England Journal of Medicine* 2014;371(2):119-29.

24. Smith JF, Eisenberg ML, Millstein SG, et al. The use of complementary and alternative fertility treatment in couples seeking fertility care: data from a prospective cohort in the United States. *Fertility and sterility* 2010;93(7):2169-74.

25. Kollmann M, Martins W, Lima M, et al. Strategies for improving outcome of assisted reproduction in women with polycystic ovary syndrome: systematic review and meta‐analysis. *Ultrasound in Obstetrics & Gynecology* 2016;48(6):709-18.

26. Hartrick C. Evidence-Based Interventional Pain Practice: According to Clinical Diagnoses: John Wiley & Sons 2011.

27. Poetscher AW, Gentil AF, Lenza M, et al. Radiofrequency denervation for facet joint low back pain: a systematic review. *Spine* 2014;39(14):E842-E49.

28. Cattran DC, Feehally J, Cook HT, et al. Kidney disease: improving global outcomes (KDIGO) glomerulonephritis work group. KDIGO clinical practice guideline for glomerulonephritis. *Kidney International Supplements* 2012;2(2):139-274.

29. Vecchio M, Bonerba B, Palmer SC, et al. Immunosuppressive agents for treating IgA nephropathy. *The Cochrane Library* 2015

30. Lapergue B, Blanc R, Guedin P, et al. A direct aspiration, first pass technique (ADAPT) versus stent retrievers for acute stroke therapy: an observational comparative study. *American Journal of Neuroradiology* 2016;37(10):1860-65.

31. Saber H, Rajah GB, Kherallah RY, et al. Comparison of the efficacy and safety of thrombectomy devices in acute stroke: a network meta-analysis of randomized trials. *Journal of neurointerventional surgery* 2017:neurintsurg-2017-013544.

32. Banbury MK, Kouchoukos NT, Allen KB, et al. Emboli capture using the Embol-X intraaortic filter in cardiac surgery: a multicentered randomized trial of 1,289 patients. *The Annals of thoracic surgery* 2003;76(2):508-15.

33. Giustino G, Mehran R, Veltkamp R, et al. Neurological outcomes with embolic protection devices in patients undergoing transcatheter aortic valve replacement: a systematic review and meta-analysis of randomized controlled trials. *JACC: Cardiovascular Interventions* 2016;9(20):2124-33.

34. Landoni G, Bove T, Crivellari M, et al. Acute renal failure after isolated CABG surgery: six years of experience. *Minerva anestesiologica* 2007;73(11):559-65. [published Online First: 2007/10/24]

35. Egan JR, Clarke AJ, Williams S, et al. Levosimendan for low cardiac output: a pediatric experience. *Journal of intensive care medicine* 2006;21(3):183-87.

36. Kolseth SM, Nordhaug DO, Stenseth R, et al. Prophylactic treatment with levosimendan: a retrospective matched-control study of patients with reduced left ventricular function. *European Journal of Cardio-Thoracic Surgery* 2009;36(6):1024-30. doi: 10.1016/j.ejcts.2009.05.001

37. Putzu A, Clivio S, Belletti A, et al. Perioperative levosimendan in cardiac surgery: A systematic review with meta-analysis and trial sequential analysis. *International journal of cardiology* 2018;251:22-31.

38. Fonarow GC, Albert NM, Curtis AB, et al. Improving evidence-based care for heart failure in outpatient cardiology practices: primary results of the Registry to Improve the Use of Evidence-Based Heart Failure Therapies in the Outpatient Setting (IMPROVE HF). *Circulation* 2010;122(6):585-96.

39. Porapakkham P, Porapakkham P, Zimmet H, et al. B-type natriuretic peptide–guided heart failure therapy: a meta-analysis. *Archives of Internal Medicine* 2010;170(6):507-14.

40. Masson S, Latini R, Anand IS, et al. Prognostic value of changes in N-terminal pro-brain natriuretic peptide in Val-HeFT (Valsartan Heart Failure Trial). *Journal of the American College of Cardiology* 2008;52(12):997-1003.

41. Yancy CW, Jessup M, Bozkurt B, et al. 2013 ACCF/AHA guideline for the management of heart failure: executive summary. *Circulation* 2013;128(16):1810-52.

42. Giuliano AE, Jones RC, Brennan M, et al. Sentinel lymphadenectomy in breast cancer. *Journal of Clinical Oncology* 1997;15(6):2345-50.

43. Lucci A, McCall LM, Beitsch PD, et al. Surgical complications associated with sentinel lymph node dissection (SLND) plus axillary lymph node dissection compared with SLND alone in the American College of Surgeons Oncology Group Trial Z0011. *Journal of Clinical Oncology* 2007;25(24):3657-63.

44. Veronesi U, Paganelli G, Viale G, et al. A randomized comparison of sentinel-node biopsy with routine axillary dissection in breast cancer. *New England Journal of Medicine* 2003;349(6):546-53.

45. Rescigno J, Zampell JC, Axelrod D. Patterns of axillary surgical care for breast cancer in the era of sentinel lymph node biopsy. *Annals of surgical oncology* 2009;16(3):687-96.

46. Joyce D, Manning A, Carter M, et al. Meta-analysis to determine the clinical impact of axillary lymph node dissection in the treatment of invasive breast cancer. *Breast cancer research and treatment* 2015;153(2):235-40.

47. Adams HP, Del Zoppo G, Alberts MJ, et al. Guidelines for the early management of adults with ischemic stroke. *Circulation* 2007;115(20):e478-e534.

48. Rønning OM, Guldvog B. Should stroke victims routinely receive supplemental oxygen? *Stroke* 1999;30(10):2033-37.

49. Kaukonen K, Bailey M, Suzuki S, et al. Mortality related to severe sepsis and septic shock among critically ill patients in australia and new zealand, 2000-2012. *JAMA* 2014;311(13):1308-16. doi: 10.1001/jama.2014.2637

50. Rivers E, Nguyen B, Havstad S, et al. Early Goal-Directed Therapy in the Treatment of Severe Sepsis and Septic Shock. *New England Journal of Medicine* 2001;345(19):1368-77. doi: 10.1056/NEJMoa010307

51. Gauer RL. Early recognition and management of sepsis in adults: the first six hours. *American family physician* 2013;88(1)

52. Andrews B, Muchemwa L, Kelly P, et al. Simplified severe sepsis protocol: a randomized controlled trial of modified early goal-directed therapy in Zambia. *Crit Care Med* 2014;42(11):2315-24. doi: 10.1097/ccm.0000000000000541 [published Online First: 2014/07/30]

53. Jacob ST, Banura P, Baeten JM, et al. The impact of early monitored management on survival in hospitalized adult Ugandan patients with severe sepsis: a prospective intervention study. *Critical care medicine* 2012;40(7):2050.

54. Maitland K, Kiguli S, Opoka RO, et al. Mortality after fluid bolus in African children with severe infection. *New England Journal of Medicine* 2011;364(26):2483-95.

55. Angus D, Barnato A, Bell D, et al. A systematic review and meta-analysis of early goal-directed therapy for septic shock: the ARISE, ProCESS and ProMISe Investigators. *Intensive care medicine* 2015;41(9):1549-60.

56. Brower R, Lanken P, MacIntyre N, et al. National Heart, Lung, and Blood Institute ARDS Clinical Trials Network: Higher versus lower positive end-expiratory pressures in patients with the acute respiratory distress syndrome. *N Engl J Med* 2004;351(4):327-36.

57. Meade MO, Cook DJ, Guyatt GH, et al. Ventilation strategy using low tidal volumes, recruitment maneuvers, and high positive end-expiratory pressure for acute lung injury and acute respiratory distress syndrome: a randomized controlled trial. *Jama* 2008;299(6):637-45.

58. Briel M, Meade M, Mercat A, et al. Higher vs lower positive end-expiratory pressure in patients with acute lung injury and acute respiratory distress syndrome: systematic review and meta-analysis. *Jama* 2010;303(9):865-73.

59. Mercat A, Richard J-CM, Vielle B, et al. Positive end-expiratory pressure setting in adults with acute lung injury and acute respiratory distress syndrome: a randomized controlled trial. *Jama* 2008;299(6):646-55.

60. Zaky A, Lang JD. The use of intraoperative positive end expiratory pressure. *J Anesthe Clinic Res* 2011;4(308):2.

61. Walkey AJ, Del Sorbo L, Hodgson CL, et al. Higher PEEP versus Lower PEEP Strategies for Patients with Acute Respiratory Distress Syndrome. A Systematic Review and Meta-Analysis. *Annals of the American Thoracic Society* 2017;14(Supplement 4):S297-S303.

62. Baik SH. Robotic colorectal surgery. *Yonsei medical journal* 2008;49(6):891-96.

63. Panteleimonitis S, Parvaiz A. Robotic colorectal surgery. *Surgery (Oxford)* 2017

64. Kyriacou DN. Opioid vs Nonopioid Acute Pain Management in the Emergency Department. *Jama* 2017;318(17):1655-56.

65. Derry CJ, Derry S, Moore R. Single dose oral ibuprofen plus paracetamol (acetaminophen) for acute postoperative pain. *The Cochrane database of systematic reviews* 2012;6

66. Hedayati SS, Minhajuddin AT, Toto RD, et al. Prevalence of Major Depressive Episode in CKD. *American Journal of Kidney Diseases* 2009;54(3):424-32. doi: 10.1053/j.ajkd.2009.03.017

67. Unruh M. Chronic Kidney Disease: Depression in Chronic Kidney Disease: Renal&Urology News; 2013 [Available from: <http://www.renalandurologynews.com/nephrology-hypertension/chronic-kidney-disease-depression-in-chronic-kidney-disease/article/616028/> accessed 01/23/2018.

68. Hedayati SS, Yalamanchili V, Finkelstein FO. A practical approach to the treatment of depression in patients with chronic kidney disease and end-stage renal disease. *Kidney international* 2012;81(3):247-55.

69. DuBose J, Teixeira PG, Inaba K, et al. Measurable outcomes of quality improvement using a daily quality rounds checklist: one-year analysis in a trauma intensive care unit with sustained ventilator-associated pneumonia reduction. *Journal of Trauma and Acute Care Surgery* 2010;69(4):855-60.

70. Russ S, Rout S, Sevdalis N, et al. Do safety checklists improve teamwork and communication in the operating room? A systematic review. *Annals of surgery* 2013;258(6):856-71.

71. Weiss CH, Moazed F, McEvoy CA, et al. Prompting physicians to address a daily checklist and process of care and clinical outcomes: a single-site study. *American Journal of Respiratory and CriticalCare Medicine* 2011;184(6):680.

72. Krzyzanowska MK, Weeks JC, Earle CC. Treatment of locally advanced pancreatic cancer in the real world: population-based practices and effectiveness. *Journal of clinical oncology* 2003;21(18):3409-14.

73. Seufferlein T, Bachet JB, Van Cutsem E, et al. Pancreatic adenocarcinoma: ESMO–ESDO Clinical Practice Guidelines for diagnosis, treatment and follow-up†. *Annals of Oncology* 2012;23(suppl_7):vii33-vii40. doi: 10.1093/annonc/mds224

74. Xu C, Xue X, Liang N, et al. Effect of chemoradiotherapy and neoadjuvant chemoradiotherapy in resectable pancreatic cancer: a systematic review and meta-analysis. *Journal of cancer research and clinical oncology* 2014;140(4):549-59.

75. Hare DL, Toukhsati SR, Johansson P, et al. Depression and cardiovascular disease: a clinical review. *European heart journal* 2013;35(21):1365-72.

76. Sullivan M, Simon G, Spertus J, et al. Depression-related costs in heart failure care. *Archives of internal medicine* 2002;162(16):1860-66.

77. Glassman AH, O'connor CM, Califf RM, et al. Sertraline treatment of major depression in patients with acute MI or unstable angina. *Jama* 2002;288(6):701-09.

78. Azoulay E, Pochard F, Kentish-Barnes N, et al. Risk of post-traumatic stress symptoms in family members of intensive care unit patients. *American journal of respiratory and critical care medicine* 2005;171(9):987-94.

79. Nelson JE, Kinjo K, Meier DE, et al. When critical illness becomes chronic: informational needs of patients and families. *Journal of critical care* 2005;20(1):79-89.

80. Curtis JR, Treece PD, Nielsen EL, et al. Randomized trial of communication facilitators to reduce family distress and intensity of end-of-life care. *American journal of respiratory and critical care medicine* 2016;193(2):154-62.

81. Lautrette A, Darmon M, Megarbane B, et al. A communication strategy and brochure for relatives of patients dying in the ICU. *New England Journal of Medicine* 2007;356(5):469-78.

82. Lanken PN, Terry PB, DeLisser HM, et al. An official American Thoracic Society clinical policy statement: palliative care for patients with respiratory diseases and critical illnesses. *American journal of respiratory and critical care medicine* 2008;177(8):912-27.

83. Cahill PJ, Lobb EA, Sanderson C, et al. What is the evidence for conducting palliative care family meetings? A systematic review. *Palliative medicine* 2017;31(3):197-211.

84. Koester KA, Morewitz M, Pearson C, et al. Patient navigation facilitates medical and social services engagement among HIV-infected individuals leaving jail and returning to the community. *AIDS patient care and STDs* 2014;28(2):82-90.

85. Giuffrida A, Torgerson DJ. Should we pay the patient? Review of financial incentives to enhance patient compliance. *Bmj* 1997;315(7110):703-07.

86. Chang EL, Wefel JS, Hess KR, et al. Neurocognition in patients with brain metastases treated with radiosurgery or radiosurgery plus whole-brain irradiation: a randomised controlled trial. *The lancet oncology* 2009;10(11):1037-44.

87. Kocher M, Soffietti R, Abacioglu U, et al. Adjuvant whole-brain radiotherapy versus observation after radiosurgery or surgical resection of one to three cerebral metastases: results of the EORTC 22952-26001 study. *Journal of Clinical Oncology* 2010;29(2):134-41.

88. Khan M, Lin J, Liao G, et al. Comparison of WBRT alone, SRS alone, and their combination in the treatment of one or more brain metastases: Review and meta-analysis. *Tumor Biology* 2017;39(7):1010428317702903.

89. Pau H, Carney A, Walker R, et al. Is oestrogen therapy justified in the treatment of hereditary haemorrhagic telangiectasia: a biochemical evaluation. *Clinical Otolaryngology* 2000;25(6):547-50.

90. McDonald J, Bayrak-Toydemir P, Pyeritz RE. Hereditary hemorrhagic telangiectasia: an overview of diagnosis, management, and pathogenesis. *Genetics in medicine* 2011;13(7):607.

91. Piwek L, Ellis DA, Andrews S, et al. The rise of consumer health wearables: promises and barriers. *PLoS Medicine* 2016;13(2):e1001953.

92. Stephenson A, McDonough SM, Murphy MH, et al. Using computer, mobile and wearable technology enhanced interventions to reduce sedentary behaviour: a systematic review and meta-analysis. *International Journal of Behavioral Nutrition and Physical Activity* 2017;14(1):105.

93. Karki S, Cheng A. Impact of non-rinse skin cleansing with chlorhexidine gluconate on prevention of healthcare-associated infections and colonization with multi-resistant organisms: a systematic review. *Journal of Hospital Infection* 2012;82(2):71-84.

94. CADTH Rapid Response Reports. Chlorhexidine Gluconate Wipes for Infection Prevention in Acute and Critical Care: A Review of Clinical Effectiveness and Cost-Effectiveness. Ottawa (ON): Canadian Agency for Drugs and Technologies in Health

Copyright (c) 2016 Canadian Agency for Drugs and Technologies in Health. 2016.

95. Kiecolt-Glaser JK, Page GG, Marucha PT, et al. Psychological influences on surgical recovery: perspectives from psychoneuroimmunology. *American Psychologist* 1998;53(11):1209.

96. Sjöling M, Nordahl G, Olofsson N, et al. The impact of preoperative information on state anxiety, postoperative pain and satisfaction with pain management. *Patient education and counseling* 2003;51(2):169-76.

97. Handoll H, Brealey S, Rangan A, et al. Protocol for the ProFHER (PROximal Fracture of the Humerus: Evaluation by Randomisation) trial: a pragmatic multi-centre randomised controlled trial of surgical versus non-surgical treatment for proximal fracture of the humerus in adults. *BMC musculoskeletal disorders* 2009;10(1):140.

98. Handoll H, Ollivere BJ, Rollins KE. Interventions for treating proximal humeral fractures in adults. *Cochrane Database Syst Rev* 2012;12

99. Athanasoulis CA, Kaufman JA, Halpern EF, et al. Inferior vena caval filters: review of a 26-year single-center clinical experience. *Radiology* 2000;216(1):54-66.

100. Stein PD, Kayali F, Olson RE. Twenty-one-year trends in the use of inferior vena cava filters. *Archives of internal medicine* 2004;164(14):1541-45.

101. Jiang J, Jiao Y, Zhang X. The short-term efficacy of vena cava filters for the prevention of pulmonary embolism in patients with venous thromboembolism receiving anticoagulation: Meta-analysis of randomized controlled trials. *Phlebology* 2017;32(9):620-27.

102. Nelson ME, Rejeski WJ, Blair SN, et al. Physical activity and public health in older adults: recommendation from the American College of Sports Medicine and the American Heart Association. *Circulation* 2007;116(9):1094.

103. de Souto Barreto P, Demougeot L, Vellas B, et al. Exercise training for preventing dementia, mild cognitive impairment, and clinically meaningful cognitive decline: a systematic review and meta-analysis. *The Journals of Gerontology: Series A* 2017:glx234.

104. The Omega wave: British Broadcastin Company; 2014 [Available from: <http://www.bbc.co.uk/science/humanbody/mind/articles/intelligenceandmemory/omega_three.shtml>.

105. Black A.

Brain health dramatically improved by intake of omega-3 fatty acids and fish oils 2006 [Available from: <https://www.naturalnews.com/016353_omega-3_fatty_acids_mental_health.html>.

106. Kane RL, Butler M, Fink HA, et al. Interventions to prevent age-related cognitive decline, mild cognitive impairment, and clinical alzheimer’s-type dementia. 2017

107. Lin CWC, Donkers NA, Refshauge KM, et al. Rehabilitation for ankle fractures in adults. *The Cochrane Library* 2012

108. Chou R, Qaseem A, Snow V, et al. Diagnosis and Treatment of Low Back Pain: A Joint Clinical Practice Guideline from the American College of Physicians and the American Pain SocietyDiagnosis and Treatment of Low Back Pain. *Annals of internal medicine* 2007;147(7):478-91.

109. Hawkey CJ. Stem cell transplantation for Crohn's disease. *Best Practice & Research Clinical Haematology* 2004;17(2):317-25.

110. Qiu X, Feng J-R, Chen L-P, et al. Efficacy and safety of autologous hematopoietic stem cell therapy for refractory Crohn's disease: A systematic review and meta-analysis. *Medicine* 2017;96(26)

111. Knot J, Moťovská Z. Therapeutic hypothermia after cardiac arrest—Part 1: Mechanism of action, techniques of cooling, and adverse events. *cor et vasa* 2012;54(4):e237-e42.

112. Nolan JP, Morley PT, Vanden Hoek TL, et al. Therapeutic hypothermia after cardiac arrest: an advisory statement by the advanced life support task force of the International Liaison Committee on Resuscitation. *Circulation* 2003;108(1):118-21. doi: 10.1161/01.cir.0000079019.02601.90 [published Online First: 2003/07/09]

113. 2005 American Heart Association Guidelines for Cardiopulmonary Resuscitation and Emergency Cardiovascular Care. *Circulation* 2005;112(24 Suppl):Iv1-203. doi: 10.1161/circulationaha.105.166550 [published Online First: 2005/11/30]

114. Busch M, Soreide E, Lossius HM, et al. Rapid implementation of therapeutic hypothermia in comatose out‐of‐hospital cardiac arrest survivors. *Acta anaesthesiologica Scandinavica* 2006;50(10):1277-83.

115. Arrich J, Holzer M, Havel C, et al. Pre‐hospital versus in‐hospital initiation of cooling for survival and neuroprotection after out‐of‐hospital cardiac arrest. *The Cochrane Library* 2016

116. Steen S, Liao Q, Pierre L, et al. The critical importance of minimal delay between chest compressions and subsequent defibrillation: a haemodynamic explanation. *Resuscitation* 2003;58(3):249-58. doi: <https://doi.org/10.1016/S0300-9572(03)00265-X>

117. Wik L, Kramer-Johansen J, Myklebust H, et al. Quality of cardiopulmonary resuscitation during out-of-hospital cardiac arrest. *Jama* 2005;293(3):299-304.

118. System LCC. About us [Available from: <http://www.lucas-cpr.com/en/about_us/about_us> accessed 04/12/2018.

119. Li H, Wang D, Yu Y, et al. Mechanical versus manual chest compressions for cardiac arrest: a systematic review and meta-analysis. *Scandinavian journal of trauma, resuscitation and emergency medicine* 2016;24(1):10.

120. Walters ST, Miller E, Chiauzzi E. Wired for wellness: e-Interventions for addressing college drinking. *Journal of substance abuse treatment* 2005;29(2):139-45.

121. Nelson TF, Naimi TS, Brewer RD, et al. The state sets the rate: the relationship among state-specific college binge drinking, state binge drinking rates, and selected state alcohol control policies. *American Journal of Public Health* 2005;95(3):441-46.

122. Kypri K, Hallett J, Howat P, et al. Randomized controlled trial of proactive web-based alcohol screening and brief intervention for university students. *Archives of internal medicine* 2009;169(16):1508-14.

123. Bewick BM, Trusler K, Barkham M, et al. The effectiveness of web-based interventions designed to decrease alcohol consumption—a systematic review. *Preventive medicine* 2008;47(1):17-26.

124. Elliott JC, Carey KB, Bolles JR. Computer-based interventions for college drinking: a qualitative review. *Addictive behaviors* 2008;33(8):994-1005.

125. Fedorowicz Z, Lodge M, Al‐asfoor A, et al. Resection versus no intervention or other surgical interventions for colorectal cancer liver metastases. *The Cochrane Library* 2008

126. Arulampalam T, Francis D, Visvikis D, et al. FDG-PET for the pre-operative evaluation of colorectal liver metastases. *European Journal of Surgical Oncology (EJSO)* 2004;30(3):286-91.

127. Kantorová I, Lipská L, Bêlohlávek O, et al. Routine 18F-FDG PET preoperative staging of colorectal cancer: comparison with conventional staging and its impact on treatment decision making. *Journal of Nuclear Medicine* 2003;44(11):1784-88.

128. Desai DC, Zervos EE, Arnold MW, et al. Positron emission tomography affects surgical management in recurrent colorectal cancer patients. *Annals of surgical oncology* 2003;10(1):59-64.

129. Zhang W, Nuki G, Moskowitz R, et al. OARSI recommendations for the management of hip and knee osteoarthritis: part III: Changes in evidence following systematic cumulative update of research published through January 2009. *Osteoarthritis and Cartilage* 2010;18(4):476-99.

130. Cheon YK. How to Interpret a Functional or Motility Test-Sphincter of Oddi Manometry. *Journal of neurogastroenterology and motility* 2012;18(2):211.

131. Coyle WJ, Pineau B, Tarnasky P, et al. Evaluation of unexplained acute and acute recurrent pancreatitis using endoscopic retrograde cholangiopancreatography, sphincter of Oddi manometry and endoscopic ultrasound. *Endoscopy* 2002;34(08):617-23.

132. disorders Ifffg. Sphincter of Oddi Dysfunction 2016 [Available from: <https://www.iffgd.org/upper-gi-disorders/sphincter-of-oddi-dysfunction.html> accessed 1/24/2018.

133. Geenen J, Toouli J, Hogan W, et al. Endoscopic sphincterotomy: follow-up evaluation of effects on the sphincter of Oddi. *Gastroenterology* 1984;87(4):754-58.

134. Craig A, Toouli J. Sphincterotomy for biliary sphincter of Oddi dysfunction. *The Cochrane Library* 2001

135. Sofi AA, Nawras A, Alaradi OH, et al. Does endoscopic sphincterotomy reduce the risk of post‐endoscopic retrograde cholangiopancreatography pancreatitis after biliary stenting? A systematic review and meta‐analysis. *Digestive Endoscopy* 2016;28(4):394-404.

136. Kanafani ZA, Khalifé N, Kanj SS, et al. Antibiotic use in acute cholecystitis: practice patterns in the absence of evidence-based guidelines. *Journal of Infection* 2005;51(2):128-34.

137. Higgins A, London J, Charland S, et al. Prophylactic antibiotics for elective laparoscopic cholecystectomy: are they necessary? *Archives of Surgery* 1999;134(6):611-14.

138. Gottenberg J-E, Seror R, Miceli-Richard C, et al. Serum levels of beta2-microglobulin and free light chains of immunoglobulins are associated with systemic disease activity in primary Sjögren’s syndrome. Data at enrollment in the prospective ASSESS cohort. *PloS one* 2013;8(5):e59868.

139. Costedoat‐Chalumeau N, Amoura Z, Duhaut P, et al. Safety of hydroxychloroquine in pregnant patients with connective tissue diseases: A study of one hundred thirty‐three cases compared with a control group. *Arthritis & Rheumatology* 2003;48(11):3207-11.

140. Fox RI, Chan E, Benton L, et al. Treatment of primary Sjögren's syndrome with hydroxychloroquine. *The American journal of medicine* 1988;85(4):62-67.

141. Tishler M, Yaron I, Shirazi I, et al. Hydroxychloroquine treatment for primary Sjögren’s syndrome: its effect on salivary and serum inflammatory markers. *Annals of the Rheumatic Diseases* 1999;58(4):253-56.

142. Wang S-Q, Zhang L-W, Wei P, et al. Is hydroxychloroquine effective in treating primary Sjogren’s syndrome: a systematic review and meta-analysis. *BMC musculoskeletal disorders* 2017;18(1):186.

143. (SAMHSA) SAaMHSA. Screening, Brief Intervention, and Referral to Treatment (SBIRT) 2017 [Available from: <https://www.samhsa.gov/sbirt> accessed 1/24/2018.

144. Babor TF, McRee BG, Kassebaum PA, et al. Screening, Brief Intervention, and Referral to Treatment (SBIRT) toward a public health approach to the management of substance abuse. *Substance abuse* 2007;28(3):7-30.

145. Barnes PM, Bloom B, Nahin RL. Complementary and alternative medicine use among adults and children; United States, 2007. 2008

146. Vickers AJ, Cronin AM, Maschino AC, et al. Acupuncture for chronic pain: individual patient data meta-analysis. *Archives of internal medicine* 2012;172(19):1444-53.

147. Grundy SM, Benjamin IJ, Burke GL, et al. Diabetes and cardiovascular disease: a statement for healthcare professionals from the American Heart Association. *Circulation* 1999;100(10):1134-46. [published Online First: 1999/09/08]

148. Bax JJ, Young LH, Frye RL, et al. Screening for coronary artery disease in patients with diabetes. *Diabetes care* 2007;30(10):2729-36.

149. Rados DV, Pinto LC, Leitão CB, et al. Screening for coronary artery disease in patients with type 2 diabetes: a meta-analysis and trial sequential analysis. *BMJ open* 2017;7(5):e015089.

150. Chertow GM, Levy EM, Hammermeister KE, et al. Independent association between acute renal failure and mortality following cardiac surgery. *The American journal of medicine* 1998;104(4):343-48.

151. Rosner MH, Okusa MD. Acute kidney injury associated with cardiac surgery. *Clinical journal of the American Society of Nephrology* 2006;1(1):19-32.

152. Landoni G, Biondi-Zoccai GG, Tumlin JA, et al. Beneficial impact of fenoldopam in critically ill patients with or at risk for acute renal failure: a meta-analysis of randomized clinical trials. *American journal of kidney diseases* 2007;49(1):56-68.

153. Gillies MA, Kakar V, Parker RJ, et al. Fenoldopam to prevent acute kidney injury after major surgery—a systematic review and meta-analysis. *Critical Care* 2015;19(1):449.

154. Force UPST. Aspirin for the primary prevention of cardiovascular events: recommendation and rationale. *Annals of Internal Medicine* 2002;136(2):157.

155. Morimoto T, Fukui T, Lee TH, et al. Application of US guidelines in other countries: aspirin for the primary prevention of cardiovascular events in Japan. *The American journal of medicine* 2004;117(7):459-68.

156. Guirguis-Blake JM, Evans CV, Senger CA, et al. Aspirin for the primary prevention of cardiovascular events: A systematic evidence review for the u.s. preventive services task force. *Annals of Internal Medicine* 2016;164(12):804-13. doi: 10.7326/M15-2113

157. Conde-Agudelo A, Romero R, Kusanovic JP. NIFEDIPINE FOR THE MANAGEMENT OF PRETERM LABOR: A SYSTEMATIC REVIEW AND METAANALYSIS. *American journal of obstetrics and gynecology* 2011;204(2):134.e1-34.20. doi: 10.1016/j.ajog.2010.11.038

158. Goldenberg RL. The management of preterm labor. *Obstetrics & Gynecology* 2002;100(5):1020-37.

159. Naik Gaunekar N, Raman P, Bain E, et al. Maintenance therapy with calcium channel blockers for preventing preterm birth after threatened preterm labour. *The Cochrane Library* 2013

160. Tallia AF, Wood UR. Diagnostic and therapeutic injection of the elbow region. *Am Fam Physician* 2002;66:2097-100.

161. Stasinopoulos D, Johnson M. Physiotherapy and tennis elbow/lateral epicondylitis. *BMJ* 2004

162. Sayegh ET, Strauch RJ. Does nonsurgical treatment improve longitudinal outcomes of lateral epicondylitis over no treatment? A meta-analysis. *Clinical Orthopaedics and Related Research®* 2015;473(3):1093-107.

163. Freeman BD, Morris PE. Tracheostomy practice in adults with acute respiratory failure. *Critical care medicine* 2012;40(10):2890-96.

164. Howie J, Mackenzie S, MacKirdy F. Scottish Intensive Care Society Audit Group Annual Report 2002. 2002, 2013.

165. Huang H, Li Y, Ariani F, et al. Timing of tracheostomy in critically ill patients: a meta-analysis. *PloS one* 2014;9(3):e92981.

166. Doig GS, Simpson F, Finfer S, et al. Effect of evidence-based feeding guidelines on mortality of critically ill adults: a cluster randomized controlled trial. *Jama* 2008;300(23):2731-41.

167. Singer P, Berger MM, Van den Berghe G, et al. ESPEN guidelines on parenteral nutrition: intensive care. *Clinical nutrition* 2009;28(4):387-400.

168. McClave SA, Martindale RG, Vanek VW, et al. ASPEN Board of Directors; American College of Critical Care Medicine; Society of Critical Care Medicine. Guidelines for the provision and assessment of nutrition support therapy in the adult critically ill patient: Society of Critical Care Medicine (SCCM) and American Society for Parenteral and Enteral Nutrition (ASPEN). *JPEN J Parenter Enteral Nutr* 2009;33(3):277-316.

169. Wan X, Gao X-J, Tian F, et al. Early parenteral nutrition alone or accompanying enteral nutrition in critically ill patients: a systematic review and meta-analysis. *Asia Pacific journal of clinical nutrition* 2015;24(2):227-33.

170. Yan L, Spitznagel EL. Soy consumption and prostate cancer risk in men: a revisit of a meta-analysis–. *The American journal of clinical nutrition* 2009;89(4):1155-63.

171. Hussain M, Banerjee M, Sarkar FH, et al. Soy isoflavones in the treatment of prostate cancer. *Nutrition and cancer* 2003;47(2):111-17.

172. Konkel L. Could Eating Too Much Soy Be Bad for You? : Scientific American; 2009 [Available from: <https://www.scientificamerican.com/article/soybean-fertility-hormone-isoflavones-genistein/>.

173. Herbs or Natural Products That Protect Against Cancer Growth. Oncology nursing forum; 2004.

174. Brett KM, Madans JH. Use of postmenopausal hormone replacement therapy: estimates from a nationally representative cohort study. *American journal of epidemiology* 1997;145(6):536-45.

175. Benkhadra K, Mohammed K, Al Nofal A, et al. Menopausal hormone therapy and mortality: a systematic review and meta-analysis. *The Journal of Clinical Endocrinology & Metabolism* 2015;100(11):4021-28.

176. Mahmoodi BK, Cushman M, Næss IA, et al. Association of traditional cardiovascular risk factors with venous thromboembolism: an individual participant data meta-analysis of prospective studies. *Circulation* 2016:CIRCULATIONAHA. 116.024507.

177. Manian FA, Ponzillo JJ. Compliance with routine use of gowns by healthcare workers (HCWs) and non-HCW visitors on entry into the rooms of patients under contact precautions. *Infection Control & Hospital Epidemiology* 2007;28(3):337-40.

178. De Angelis G, Cataldo MA, De Waure C, et al. Infection control and prevention measures to reduce the spread of vancomycin-resistant enterococci in hospitalized patients: a systematic review and meta-analysis. *Journal of Antimicrobial Chemotherapy* 2014;69(5):1185-92.

179. Control CfD, Prevention. Revised guidelines for HIV counseling, testing, and referral. *MMWR Recommendations and reports: Morbidity and mortality weekly report Recommendations and reports* 2001;50(RR-19):1.

180. Branson BM, Handsfield HH, Lampe MA, et al. Revised recommendations for HIV testing of adults, adolescents, and pregnant women in health-care settings. *Morbidity and Mortality Weekly Report: Recommendations and Reports* 2006;55(14):1-CE-4.

181. Brown J. The changing landscape of state legislation and expanded HIV testing. *Public Health Reports* 2008;123(3_suppl):16-20.

182. Waikar SS, Chertow GM. Crystalloids versus colloids for resuscitation in shock. *Current opinion in nephrology and hypertension* 2000;9(5):501-4. [published Online First: 2000/09/16]

183. Jiang L, Jiang S, Zhang M, et al. Albumin versus Other Fluids for Fluid Resuscitation in Patients with Sepsis: A Meta-Analysis. *PLoS ONE* 2014;9(12):e114666. doi: 10.1371/journal.pone.0114666

184. Larson DG, Tobin DR. End-of-life conversations: evolving practice and theory. *Jama* 2000;284(12):1573-78.

185. Baile WF, Buckman R, Lenzi R, et al. SPIKES—a six-step protocol for delivering bad news: application to the patient with cancer. *The oncologist* 2000;5(4):302-11.

186. Selman LE, Brighton LJ, Hawkins A, et al. The effect of communication skills training for generalist palliative care providers on patient-reported outcomes and clinician behaviors: A systematic review and meta-analysis. *Journal of pain and symptom management* 2017;54(3):404-16. e5.

187. Parkman HP, Van Natta ML, Abell TL, et al. Effect of nortriptyline on symptoms of idiopathic gastroparesis: the NORIG randomized clinical trial. *Jama* 2013;310(24):2640-49.

188. Abell T, Bernstein VK, Cutts T, et al. Treatment of gastroparesis: a multidisciplinary clinical review. *Neurogastroenterology & Motility* 2006;18(4):263-83.

189. Gumaste V, Baum J. Treatment of gastroparesis: an update. *Digestion* 2008;78(4):173-79.

190. Parkman HP, Van Natta ML, Abell TL, et al. Effect of nortriptyline on symptoms of idiopathic gastroparesis: The norig randomized clinical trial. *JAMA* 2013;310(24):2640-49. doi: 10.1001/jama.2013.282833

| **#** | **Article and Author** | | **Primary Medical Discipline** | | **Date and Journal** | | **Summary** | | **Systematic Review** | | **Systematic Review Search Terms** | |
| --- | --- | --- | --- | --- | --- | --- | --- | --- | --- | --- | --- | --- |
| 82 | Effect of mammographic screening from age 40 years on breast cancer mortality at 10 years' follow-up: a randomized controlled trial Moss et al. | | Public health and general preventive medicine | | 12/9/2006  LANCET | | In the past, the American Cancer Society recommended that women between the ages of 40-49 get mammograms every 1-2 years.^1^ The benefit of mammograms for women under the age of 50 has not been established. 160 921 women aged 39-41 years old were randomly assigned in the ratio of 1:2 to an intervention group of annual mammography to age 48 or to a control group of usual medical care. At a mean follow-up of 10.7 years, there was no significant difference in breast cancer mortality between the intervention and control groups (relative risk 0.83 [95% CI 0.66-1.04], p=0.11). This is a reversal of the recommendation of mammographic screening every 1-2 years for women ages 40-49. | | 2013. Cochrane review. “The chance that a woman will benefit from attending screening is small at best, and - if based on the randomised trials - ten times smaller than the risk that she may experience serious harm in terms of overdiagnosis.”^2^ | | PubMed | |
| 83 | Magnesium sulphate for treatment of severe tetanus: a randomized controlled trial Thwaites et al. | | Clinical care medicine | | 10/21/2006  LANCET | | Tetanus, while rare in the developed world, is still common in other parts of the world, and has high mortality in those regions.^3^ Magnesium sulfate is sometimes used for this indication because it inhibits the release of catecholamines and is a neuromuscular relaxant.^3 4^ This study, conducted in Ho Chi Minh City, Vietnam, compared magnesium sulphate (n=97) to placebo (n=98) in treatment of patients with severe tetanus. They found no difference in need for mechanical ventilation between the two groups (odds ratio 0.71, [95% CI 0.36 – 1.40; p=0.324]), no difference in survival. The magnesium group, however, required less midazolam and pipecuronium to control muscle spasms. This is a reversal of the use of magnesium sulphate for treatment of severe tetanus. | | 2012. Anaesthetists of Great Britain and Ireland. “Magnesium sulphate did not reduce mortality, relative risk (95% CI): vs placebo, 0.80 (0.41–1.58); vs diazepam, 1.11 (0.70–1.75). The data on duration of total intensive care unit stay, total hospital stay and the need for ventilatory support were conflicting and pooling of results could not be done due to methodological differences of individual trials.”^5^ | | PubMed | |
| 84 | 30 day results from the SPACE trial of stent-protected angioplasty versus carotid endarterectomy in symptomatic patients: a randomized non-inferiority trial Ringleb et al. | | Cardiovascular disease | | 10/7/2006  LANCET | | Surgery is often recommended after ischemic events for patients with carotid stenosis.^6^ Carotid endarterectomy has often been used but carotid-artery stenting has been used with increasing frequency.^7^ The SPACE trial randomized 1200 patients within 180 days of transient ischemic attack or moderate stroke to either carotid-artery stenting (n=605) or carotid endarterectomy (n=595). This trial failed to show the non-inferiority of carotid-artery stenting compared to carotid endarterectomy in regards of death 30-day death rate (6.84% vs. 6.34%). This is a reversal of the use of carotid artery stenting for patients within 180 days of transient ischemic attack or moderate stroke. | | 2010. “Carotid endarterectomy was found to be superior to carotid artery stenting for short term outcomes  but the difference was not significant for intermediate term outcomes; this difference was mainly driven by nondisabling stroke.”^8^ | | stent-protected angioplasty, carotid endarterectomy | |
| 85 | Secondary prevention of asthma by the use of Inhaled Fluticasone propionate in Wheezy INfants (IFWIN): double-blind, randomized, controlled study Murray et al. | | Pulmonary disease | | 8/26/2006  LANCET | | Wheeze is common in young children, affecting up to half of children by the time they are six.^9^ Even though children often grow out of their wheeze, about one-third continue with wheezing and asthma symptoms later in life.^10^ Steroids are commonly used for this indication, and, specifically, fluticasone proprionate is recommended by the British National Formulary for adults with asthma.^11^ They followed 1073 children prospectively, of whom 333 were eligible, and 200 began treatment (101 in placebo group and 99 in treatment group). The groups did not differ significantly in proportion of children with current wheeze, physician-diagnosed asthma or use of asthma medication, lung function, or airway reactivity. This is a reversal of the use of inhaled fluticasone propionate for secondary prevention of asthma. | | No SR/MA specifically referencing the natural history of asthma | | Inhaled Fluticasone, asthma, long-term, later childhood | |
| 86 | Continuous venovenous hemodiafiltration versus intermittent hemodialysis for acute renal failure in patients with multiple-organ dysfunction syndrome: a multicenter randomized trial Vinsonneau et al. | | Nephrology | | 7/29/2006  LANCET | | Acute renal failure is a serious condition that has historically had high mortality, but because of dialysis, the mortality rate has decined.^12^ Both continuous renal replacement therapy and intermittent dialysis have been used, and while both have advantages and disadvantages, continuous venovenous hemodiafiltration is more intensive and costly.^12^ This study compared the two methods, using the same polymer membrane and bicarbonate-based buffer, and looked at 60-day survival. 360 patients were randomized to either continuous (n=184) or intermittent (n=176). They found the rate of 60-day survival did not differ between intermittent (32%) and continuous (33%). This is a reversal of the practice of continuous venous hemodiafiltration in patients with acute renal failure and multiple-organ dysfunction syndrome. | | 2007. Cochrane review. “We did not ﬁnd any difference between CRRT and IRRT with respect to mortality, renal recovery, and risk of haemodynamic instability or hypotension episodes.”^13^ | | PubMed | |
| 87 | Intrauterine insemination with controlled ovarian hyper stimulation versus expectant management for couples with unexplained subfertility and an intermediate prognosis: a randomized clinical trial Steures et al. | | Obstetrics and Gynecology | | 7/15/2006  LANCET | | Ovarian stimulation is used to treat infertility because it is thought that the multiple mature follicles with stimulation would lead to higher pregnancy rates. Follicle stimulating hormone and gonadotrophin-releasing hormone are often used in ovarian hyperstimulation, and several formulations were on the market at the time of this study.^14^ In this study, 253 couples were randomized to either intrauterine insemination with controlled ovarian hyperstimulation (n=127) for 6 months, or expectant managements (n=126) for 6 months. They found no significant difference in ongoing pregnancy at 6 months. 42 (33%) women in the intervention group conceived, and 29 (23%) were ongoing. 40 (32%) women in the expectant management group conceived, and 34 (27%) were ongoing. This is a reversal of the use of intrauterine insemination with controlled ovarian hyper stimulation for conception. | | 2016. Cochrane review. “However the trials provided insufficient data to investigate the impact of IUI with or without OH on several important outcomes including live births, multiple pregnancies, miscarriage and risk of ovarian hyperstimulation. There was no evidence of a difference in pregnancy rate for IUI with OH compared with timed intercourse in a natural cycle.”^15^ | | PubMed | |
| 88 | Aminophylline in bradyasystolic cardiac arrest: a randomized placebo-controlled trial Abu-Laban et al. | | Cardiovascular disease | | 5/13/2006  LANCET | | Aminophylline is an adenosine antagonist and stimulates the release of catecholamines.^16^ Interest in aminophylline in the treatment of bradyasystolic cardiac arrest began in the early 1990s after the publication of several anecdotal reports. Since then, aminophylline has been sporadically used, but was included in CPR guidelines.^17 18^ This study randomized 971 subjects with asystole or pulseless electrical activity at fewer than 60 beats per minute, and who were unresponsive to initial treatment with epinephrine and atropine, to receive intravenous aminophylline (250 mg, and additional 250 mg if necessary) (n=486) or placebo (n=485). They found that, while aminophylline increased non-sinus tachyarrhythmias (34.6% vs. 26.2%), there was no difference in patients who survived to hospital discharge in the aminophylline group (2, 0.5%) and the placebo group (3, 0.6%). This is a reversal of the use of aminophylline in bradyasystolic cardiac arrest. | | 2015. Cochrane review. “The prehospital administration of aminophylline in bradyasystolic arrest is not associated with improved return of circulation, survival to admission or survival to hospital discharge.”^16^ | | PubMed | |
| 89 | Effects of routine prophylactic supplementation with iron and folic acid on admission to hospital and mortality in preschool children in a high malaria transmission setting: community-based, randomised, placebo-controlled trial Sazawal et al. | | Public health and general preventive medicine | | 1/14/2006  Lancet | | Children are often at high risk of being iron deficient, and as such, the World Health Organization guidelines recommend iron supplementation for children 2-5 years old.^19^ However, in populations that are also at high risk of malaria, recommendations may need to be reconsidered, as iron deficiency may protect against malaria and malaria-related deaths.^20^ Children aged 1-35 months living in Pemba, Zanzibar were assigned to either daily oral supplementation with iron (12.5 mg) and folic acid (50 μg, n=7950), iron, folic acid and zinc (n=8120), or placebo (n=8006). The iron and folic acid containing groups were stopped early due to safety concerns. Those who received iron and folic acid with or without zinc were 12% (95% CI 1.02 to 1.23, p=0.02) more likely to experience an adverse event and 11% (1.01 to 1.23, p=0.03), more like to be admitted to the hospital, and no more likely to have reduced mortality (RR=1.15; 95% CI=0.93 to 1.41, p=0.19). This is a reversal of the practice of prophylactic iron and folic acid supplementation for reducing hospital admissions and mortality in preschool children in a high malaria transmission locale. | | 2016. Cochrane review. The review concluded that iron plus folic acid supplementation does not have any effect on mortality, and hospitalizations were no different, although this RCT was not included in the meta-analysis on hospitalization.^21^ | | PubMed | |
| 90 | Effect of BCG revaccination on incidence of tuberculosis in school-aged children in Brazil: the BCG-REVAC cluster-randomized trial Rodrigues et al. | | Pulmonary disease | | 10/8/2005  LANCET | | The Bacillus Calmette-Guerin (BCG) vaccine was developed as a prevention method for tuberculosis, a prevalent and sometimes serious bacterial infection. Revaccination for tuberculosis is common in European countries, where at least a dozen of them had a policy for revaccination for children 5-8 years of age and more had policies for people 10-15 years of age.^22^ BCG vaccination is common in Brazil, but revaccination varies from state to state. 386 schools (176 846 children) were assigned BCG revaccination and 365 (171 293) no revaccination. 42 053 in the vaccine group were absent in the vaccine group and 47006 in the control group. The crude incidence of tuberculosis in the intervention group was 29.3 per 100 000 person years, and 30.2 per 100 000 in the control group. The efficacy of BCG revaccination was 9% (95% CI -16 to 29%). This is a reversal of the policy of BCG revaccination for incidence of tuberculosis. | | None for revaccination | | BCG revaccination, tuberculosis, school-aged children, adolescents | |
| 91 | Assessment of the clinical effectiveness of pulmonary artery catheters in management of patients in intensive care (PAC-Man): a randomized controlled trial Harvey et al. | | Clinical care medicine | | 8/6/2005  LANCET | | The pulmonary artery catheter (PAC) was introduced 30 years ago, and it is widely used in critically ill patients, yet there has been no formal assessment of either its clinical effectiveness or cost-effectiveness^23^. Subjects in this study were identified by the treating physician as someone who should be managed using invasive hemodynamic monitoring, and randomized to management with (n=519) or without (n=522) a PAC. This study found no difference in hospital mortality between subjects with or without a PAC (68% [346 of 506] vs. 66% [333 of 507], p=0.39). This is a reversal of the routine use of PAC for patients in intensive care. | | 2013. Cochrane review. “Our review concluded that use of a PAC did not alter the mortality, general ICU or hospital LOS, or cost for adult patients in intensive care.”^24^ | | PubMed | |
| 92 | Combination antibiotic susceptibility testing to treat exacerbations of cystic fibrosis associated with multiresistant bacteria: a randomized, double-blind, controlled clinical trial Aaron et al. | | Pulmonary disease | | 8/6/2005  LANCET | | People with cystic fibrosis often have chronic respiratory infections due to their inability to clear pathogens from their lower respiratory tract.^25^ Chronic treatment of these infections can cause antibiotic resistance. Synergy testing or multiple-combination bactericidal testing has been implemented in several specialized laboratories to help optimize antibiotic therapy.^26^ In this RCT, 251 patients with cystic fibrosis who were chronically infected with multiresistant gram negative bacteria gave sputum at 3-month intervals for conventional culture and sensitivity tests for combination antibiotic susceptibility tests using multiple combination bactericidal antibiotic testing (MCBT). Patients who developed an exacerbation of pulmonary disease were randomized to receive a 14-day course of any two blinded antibiotics based on results from the conventional sputum culture and sensitivity testing (n=68) or results of MCBT (n=64). They found that the treatment based off combination antibiotic susceptibility testing did not lead to fewer pulmonary exacerbations or treatment failures, compared to standard culture and sensitivity results (hazard ratio 0.86 [95% CI 0.60–1.23], p=0.40). This is a reversal of the combination antibiotic susceptibility testing to treat exacerbations of cystic fibrosis. | | 2017. Cochrane review. “The current evidence, limited to one study, shows that there is insufficient evidence to determine effect of choosing antibiotics based on combination antimicrobial susceptibility testing compared to choosing antibiotics based on conventional antimicrobial susceptibility testing in the treatment of acute pulmonary exacerbations in people with cystic fibrosis with chronic Pseudomonas aeruginosa infection.” ^27^ | | PubMed | |
| 93 | Chloramphenicol treatment for acute infective conjunctivitis in children in primary care: a randomized double-blind placebo-controlled trial Rose et al. | | Pediatrics | | 7/2/2005  LANCET | | Conjunctivitis is a very common condition in children. About half of conjunctivitis cases are bacterial in nature and most will resolve in a few days without treatment.^28^ In a UK survey, 95% of physicians reported that they prescribed an antibiotic for acute conjunctivitis, with chloramphenicol being the commonest antibiotic prescribed for this indication.^28^ This study randomized 326 children aged 6 months to 12 years with a clinical diagnosis of conjunctivitis to receive chloramphenicol eye drops (n=163) or placebo (n=163). They found clinical cure by day 7 occurred in 128 (83%) of 155 children in the placebo compared to 140 (86%) of 162 with chloramphenicol (risk difference 3.8%, [95% CI −4.1% to 11.8%]). This is a reversal of the use of chloramphenicol for treatment of acute infective conjunctivitis. | | 2011. British Journal of General Practice. “Acute conjunctivitis seen in primary care can be thought of as a self-limiting condition, with most patients getting better regardless of antibiotic therapy.”^29^  2013: Jama: “The majority of cases in bacterial conjunctivitis are self-limiting and no treatment is necessary in uncomplicated cases. However, conjunctivitis caused by gonorrhea or chlamydia and conjunctivitis in contact lens wearers should be treated with antibiotics.” | | PubMed | |
| 94 | Endovascular aneurysm repair and outcome in patients unfit for open repair of abdominal aortic aneurysm (EVAR trial 2): randomized controlled trial EVAR Trial participants, Greenhalgh et al. | | Cardiovascular disease | | 6/25/2005  LANCET | | Abdominal aortic aneurysms are more common as people age, and larger aneurysms are more likely to rupture and lead to death.^30^ Surgery is often performed on larger aneurysms to prevent adverse outcomes, but because a number of people are not good candidates for traditional surgery, endovascular repair has been offered as an alternative.^31^ In the EVAR II trial, 338 patients aged 60 years or older who had aneurysms of at least 5.5 cm in diameter and who had been referred to one of 31 hospitals in the UK were assigned to receive either EVAR (n=166) or no intervention (n=172). By the end of follow up, they found no significant difference between the two groups in all-cause mortality (hazard ratio 1.21, [95% CI 0.87–1.69], p=0.25). This is a reversal of the use of EVAR in patients unfit for open repair of abdominal aortic aneurysms. | | 2014. Cochrane review. “In individuals considered unfit for open surgery, the results of a single trial found no overall short- or long-term benefits of EVAR over no intervention with regard to all-cause mortality, but individuals may differ and individual preferences should always be taken into account.”^32^ | | PubMed | |
| 95 | Endovascular aneurysm repair versus open repair in patients with abdominal aortic aneurysm (EVAR trial 1): randomized controlled trial EVAR Trial participants, Greenhalgh et al. | | Cardiovascular | | 6/25/2005  LANCET | | Abdominal aortic aneurysms are more common as people age, and larger aneurysms are more likely to rupture and lead to death.^30^ Surgery is often performed on larger aneurysms to prevent adverse outcomes, but because a good number of people are not good candidates for traditional surgery, endovascular repair has been offered as an alternative.^31^ In the EVAR I trial, 1082 patients aged 60 years or older who had aneurysms of at least 5.5 cm in diameter and who had been referred to one of 34 hospitals proficient in the EVAR technique were assigned to EVAR (n=543) or open repair (n=539). There was a reduction in aneurysm related deaths in the EVAR group (4% vs 7%, 0.55 [0.31-0.96], p=0.04), but all-cause mortality was similar in the two groups (hazard ratio 0.90, [95% CI 0.69-1.18], p=0.46). In addition, patients in the EVAR group had a higher proportion of postoperative complications within 4 years of randomization compare to the open group (41% vs 9%, 4.9 [3.5-6.8], p<0.0001). This is a reversal of the practice of endovascular aneurysm repair in patients with abdominal aortic aneurysm. | | 2014. Cochrane review. “In individuals considered ﬁt for conventional surgery, EVAR [endovascular aneurysm repair] was associated with lower short-term mortality than OSR [open surgical repair]. However, this beneﬁt from EVAR did not persist at the intermediate- and long-term follow ups. Individuals undergoing EVAR had a higher reintervention rate than those undergoing OSR.”^32^ | | PubMed | |
| 96 | Introduction of the medical emergency team (MET) system: a cluster-randomized controlled trial Hillman et al. | | Critical care medicine | | 6/18/2005  LANCET | | Medical emergency teams (METs) are trained medical professionals that respond quickly to a change in a patient’s condition based on the premise that early intervention may prevent further deterioration and/or death. A MET can help to manage cardiac arrests, and unplanned ICU admissions that sometimes occur in a hospital.^33^ Hospitals in both the UK and Australia have established these teams into their healthcare.^34 35^ In this trial, after two months of collecting baseline data, hospitals in Australia were randomized to receive MET implementation or control. While there was a significant increase in overall calling incidence for an emergency team (3.1 vs 8.7 per 1000 admissions, p=0.0001), there was no difference in the composite outcome of cardiac arrest, unexpected death, or unplanned intensive care unit admissions between control hospitals and MED hospitals (5.86 vs 5.31 per 1000 admissions, p=0.64). This is a reversal of the use of METs. | | 2010. “Although RRTs (Rapid Response Teams) have broad appeal, robust evidence to support their effectiveness in reducing hospital mortality is lacking.”^36^ | | Google Scholar | |
| 97 | Oral vitamin D3 and calcium for secondary prevention of low-trauma fractures in elderly people (Randomized Evaluation of Calcium Or vitamin D, RECORD): a randomized placebo-controlled trial Grant et al. | | Orthopedic | | 5/7/2005  LANCET | | Vitamin D and calcium are important nutrients for bone health and are part of dietary recommendations, and higher intakes of vitamin D is recommended for older adults who have a harder time synthesizing vitamin D from the sun.^37^ For older adults, the World Health Organization Task-Force for Osteoporosis has recommended that physicians provide vitamin D to people in climates where it would be "appropriate". Canadian guidelines recommend vitamin D supplements for older adults and calcium when dietary sources are inadequate.^38^ Physicians in Canada often prescribe vitamin D and/or calcium for long-term care patients with osteoporosis. ^39^ In the RECORD trial,5292 people aged 70 or older who were mobile before developing a low-trauma fracture were randomly assigned to 800 IU daily oral vitamin D3, 1000 mg calcium, oral vitamin D3 (800 IU per day) combined with calcium (1000 mg per day), or placebo. They found that the incidence of new, low-trauma fractures did not differ between those allocated to calcium or not (331 [12.6%] of 2617 vs 367 [13.7%] of 2675; hazard ratio (HR) 0.94 [95% CI 0.81–1.09]), those allocated to vitamin D3 or not (353 [13.3%] of 2649 vs 345 [13.1%] of 2643; 1.02 [0.88–1.19]) or those allocated to the combination or not(165 [12.6%] of 1306 vs 179 [13.4%] of 1332; HR for interaction term 1.01 [0.75–1.36]). This is a reversal of the use of vitamin D supplementation for prevention of fractures. | | 2014. Cochrane. “Vitamin D alone is unlikely to prevent fractures in the doses and formulations tested so far in older people. Supplements of vitamin D and calcium may prevent hip or any type of fracture.”^40^ | | PubMed | |
| 98 | Effects of N-acetylcysteine on outcomes in chronic obstructive pulmonary disease (Bronchitis Randomized on NAC Cost-Utility Study, BRONCUS): a randomized placebo-controlled trial Decramer et al. | | Pulmonary disease | | 4/30/2005  LANCET | | Chronic obstructive pulmonary disease (COPD) is an inflammatory condition, which may be helped with anti-inflammatory drugs, such as N-acetylcysteine (NAC).^41^ Mucolytics, specifically NAC have been widely prescribed in Europe, but less commonly prescribed in other parts of the world.^42^ In this trial, subjects with COPD were randomized to 600 mg/day of N-acetylcysteine or placebo. They were followed for three years and were measured yearly on their forced expiratory volume (FEV) and number of exacerbations per year. They found no significant difference between the NAC group and the placebo group in FEV (54 mL [SE 6] *vs* 47 mL [SE 6]) nor in number of exacerbations per year (1.25 [SD 1.35] *vs* 1.29 [SD 1.46]). This is a reversal of N- acetylcysteine in patients with COPD. | | 2015. Cochrane review. The reviewers found there to be a small decrease in monthly exacerbations in groups using mucolytics. However, there were only shown in smaller, older studies. Newer, larger trials have not shown the same significant decrease, causing doubt to be raised in their conclusion. ^43^ | | PubMed | |
| 99 | Effect of timing and method of enteral tube feeding for dysphagic stroke patients (FOOD): a multicenter randomized controlled trial Dennis et al. | | Neurology/Neurosurgery | | 2/26/2005  LANCET | | Malnutrition is common in patients who have had stroke because of eating problems and impaired functional capacity, which can then lead to a poorer prognosis for the patient.^44^ Percutaneous endoscopic gastrostomy (PEG) feeding has overcome the difficulty of feeding because food can be delivered directly to the stomach, but feeding practices vary, which has raised questions about what is ethically right to do.^45^ Surveys of feeding practice after stroke have recorded much variation between hospitals in the UK, in particular, when to start enteral tube feeding and whether a nasogastric or PEG tube is used. The FOOD trial consisted of three multicenter controlled trials, two of which included dysphagic stroke patients. In one trial, patients were randomized to either early enteral tube feeding, or no tube feeding for more than 7 days. In the other, patients were randomized to PEG or nasogastric feeding. In both trials, death or poor outcome at 6 months was the primary outcome. They found a non-significant reduction in death (5.8% [95% CI -0.8 – 12.5, p=0.09]) and death or poor outcome (1.2% [-4.2 – 6.6 p=0.7]) in the early vs late group. They also found a non-significant drop in death (1% [-10.0 – 11.9 p=0.9]) and increase of death or poor outcome (7.8 [0.0 – 15.5 p=0.05]) in PEG vs nasogastric feeding. Overall, the data does not support early initiation of PEG feeding in dysphagic stroke patients. | | None found | | PubMed | |
| 100 | Routine oral nutritional supplementation for stroke patients in hospital (FOOD): a multicenter randomized controlled trial Dennis et al. | | Neurology/Neurosurgery | | 2/26/2005  LANCET | | Undernutrition is common in patients with stroke in hospital settings and is associated with poor outcomes^46^. One method to combat undernutrition is through oral nutritional supplementation, a treatment offered in some hospitals without concrete evidence to support it. In the third of three randomized trials as a part of the FOOD trials, this measured the outcomes of stroke patients who could swallow. Patients were randomized to normal hospital diet, or normal diet plus nutritional supplements until discharge. Oral supplements were associated with a non-significant reduction in death (0.7% [95% CI -1.4 – 2.7]) and a non-significant risk of death or poor outcome (0.7% [-2.3 – 3.8]) compared to normal hospital diet. This is a reversal of the practice of routine oral nutritional supplementation for stroke patients. | | 2009. Cochrane review. “Supplementation produces a small but consistent weight gain in older people. Mortality may be reduced in older people who are undernourished. There may also be a beneﬁcial effect on complications which needs to be conﬁrmed. However, this updated review found no evidence of improvement in functional beneﬁt or reduction in length of hospital stay with supplements.”^47^ | | oral nutritional supplementation, stroke, feeding, adverse effects | |
| 101 | Early surgery versus initial conservative treatment in patients with spontaneous supratentorial intracerebral hematomas in the International Surgical Trial in Intracerebral Hemorrhage (STICH): a randomized trial Mendelow et al. | | Neurology/Neurosurgery | | 1/29/2005  LANCET | | Spontaneous supratentorial intracerebral hematomas have high rates of morbidity and mortality and the recommended treatment is controversial. While trials on neurosurgery versus conservative treatment have shown conflicting outcomes^48 49^, surgeons have been practicing early surgery in patients with these hematomas^50^. This study randomized 601 patients to either early surgery (n=307) or initial conservative treatment (n=294). 174/297 (59%) of the patients in the early surgery group had unfavorable outcomes compared to 178/286 (62%) of patients in the initial conservative treatment group (absolute difference 3.7% [95% CI −4.3 to 11.6], odds ratio 0.86 [0.62 to 1.20]; p=0.367). This is a reversal of the practice of routine early surgery in patients with spontaneous supratentorial intracerebral hematomas. | | 2008. Cochrane review. “A comment about the STICH trial is warranted because this is the largest study relevant to the review. Some neurosurgeons interpret the results of the STICH trial as negative and justify denying neurosurgery to all kinds of patients with primary supratentorial intracerebral haemorrhage. This is plainly inaccurate. Nearly a quarter of participants in the medical arm of the STICH trial had surgery, mainly because of their deteriorating clinical condition. A more rational interpretation of the STICH trial would be that there is no statistically significant difference in outcomes between the policy of surgery done early in all eligible patients and that of surgery done only in those who deteriorate. ^51^ | | PubMed | |
| 102 | The United Kingdom Infantile Spasms Study comparing vigabatrin with prednisolone or tetracosactide at 14 days: a multicenter, randomized controlled trial Lux et al. | | Pediatrics | | 11/13/2004  LANCET | | Infantile spasms are a type of seizure disorder, which is often treated with adrenocorticotropic hormones (ACTH), which can have potentially life-threatening side effects.^52^ Since 1958, the usual interventions have been hormonal treatments, with either intramuscular adrenocorticotropic hormone or oral corticosteroids. Vigabatrin was approved for use in Canada in 1995, and some physicians began using this as an option.^53^ This study compared three groups with either: vigabatrin 100 mg/kg per day, oral prednisolone 40 mg per day, or intramuscular tetracosactide depot 0·5 mg (40 IU) on alternate days. They found that cessation of spasms was more likely in infants given hormonal treatments (prednisolone [21/30] or tetracosactide [19/25] than those given vigabatrin(28/52) (difference 19%, 95% CI 1%–36%, p=0.043). This is a reversal of the use of vigabatrin for infantile spasms. | | 2013. Cochrane review. “In the majority, methodology has been poor, hence it is not clear which treatment is optimal in the treatment of this epilepsy syndrome. Hormonal treatment resolves spasms in more infants than vigabatrin, but this may or may not translate into better long-term outcomes.“^54^ | | PubMed | |
| 103 | Effect of intravenous corticosteroids on death within 14 days in 10 008 adults with clinically significant head injury (MRC CRASH trial): randomized placebo-controlled trial Roberts et al. | | Critical care medicine | | 10/9/2004  LANCET | | Corticosteroids are commonly given to head injury patients in an effort to reduce swelling.^55^ In a UK study, about half of the surveyed units responded that they used corticosteroids at least some of the time to treat head injury,^56^ and the use was even higher in a US survey.^55^ 10008 adults with head injury and a Glasgow coma score (GCS) of 14 or less within 8 hours of injury were randomly allocated 48 hour infusion of corticosteroids (methylprednisolone) or placebo. They found that, compared to placebo, the risk of death from all causes within 2 weeks was higher in the corticosteroid group (1052 [21.1%] vs 893 [17.9%] deaths, relative risk 1.18 [95% CI 1.09 – 1.27]). This is a reversal of the use of intravenous corticosteroids on patients with clinically significant head injuries. | | 2005. Cochrane review. “In the absence of a meta-analysis, we feel most weight should be placed on the result of the largest trial. The increase in mortality with steroids in this trial suggest that steroids should no longer be routinely used in people with traumatic head injury.”^57^ | | PubMed | |
| 104 | Oropharyngeal and nasopharyngeal suctioning of meconium-stained neonates before delivery of their shoulders: multicenter, randomized controlled trial Vain et al. | | Obstetrics and Gynecology | | 8/14/2004  LANCET | | Meconium aspiration syndrome (MAS) is a life-threatening respiratory disorder in infants born through meconium-stained amniotic fluid (MSAF). The aspiration of MSAF can be very dangerous for babies being born. During the 1960s, tracheal suctioning was introduced as a way to prevent complications, with oropharyngeal suctioning being added to the practice in the 1970s.^58^ By the 1990s, suctioning practices were introduced into several guidelines.^59^ This trial randomized patients of gestational age of at least 37 weeks and cephalic presentation to either suctioning of the oropharynx and nasopharynx (including the hypopharynx) before delivery of the shoulders (n=1263) or to no suctioning (n=1251). They found that there was no significant difference between the two groups in incidence of MAS (52 [4%] suction *vs* 47 [4%] no suction; relative risk 0.9, 95% CI 0.6–1.3), need for mechanical ventilation for MAS (24 [2%] *vs* 18 [1%]; 0.8, 0.4–1.4), mortality (9 [1%] *vs* 4 [0.3%]; 0.4, 0.1–1.5), or in the duration of ventilation, oxygen treatment, and hospital care. This is a reversal of the use of suctioning practices for incidence of MAS. | | 2017. Cochrane review. “The currently available evidence does not support or refute the benefits or harms of routine oro/nasopharyngeal suction over no suction.”^60^ | | PubMed | |
| 105 | Doubling the dose of inhaled corticosteroid to prevent asthma exacerbations: randomized controlled trial Harrison et al. | | Pulmonary disease | | 1/24/2004  LANCET | | Asthma is treated with steroids because of their anti-inflammatory effect. Patients are often started with inhaled corticosteroids (ICS), but when the recommended dose does not control asthma exacerbations, increasing the dose is a widespread practice, and has even been supported by guidelines.^61^ This study aimed to investigate whether doubling the dose of inhaled corticosteroid when asthma control starts to deteriorate reduces the number of patients needing prednisolone. 390 patients were randomized into an active (n=192) or placebo (n=198) inhaler in addition to their usual corticosteroid for 14 days. They found that doubling the dose of inhaled steroids did not reduce the risk for needing to go on prednisolone treatment over the course of 12 months (Risk Ratio for starting prednisolone was .95 [95% CI 0.55 – 1.64, p=.8]). This is a reversal of the doubling of inhaled corticosteroid to prevent asthma exacerbations. | | 2016. Cochrane review. “Current evidence does not support increasing the dose of ICS as part of a self-initiated action plan to treat exacerbations in adults and children with mild to moderate asthma. Increased ICS dose is not associated with a statistically significant reduction in the odds of requiring rescue oral corticosteroids for the exacerbation, or of having adverse events, compared with a stable ICS dose”.^62^ | | PubMed | |
| 106 | Efficacy of a short course of parent-initiated oral prednisolone for viral wheeze in children aged 1–5 years: randomized controlled trial Oommen et al. | | Pediatrics | | 11/1/2003  LANCET | | Wheeze is common in young children, affecting up to half of children by the time they are six.^9^ Often, the children grow out of it, but it is commonly treated with corticosteroids.^63^ In this study, children aged 1–5 years admitted to hospital with viral wheeze were allocated to either a high-primed or low-primed stratum according to amounts of serum eosinophil cationic protein and eosinophil protein X, and randomized to parent-initiated prednisolone (n=109) or placebo (n=108) for the next episode. There was no significant different in 7-day mean daytime (difference in means -0.01 [-0.22 – 0.20]) and nighttime respiratory symptom scores (0.10 [−0.12 to 0.32]) between the two groups. This is a reversal of the use of short course patient-initiated oral prednisolone for viral wheeze in young children. | | 2007. Cochrane review. “Limited current evidence is available and it is inconclusive regarding the beneﬁt from patient- and parents- initiate oral corticosteroids (PIOCS) therapy in the treatment of intermittent wheezing illnesses in children.”^64^  2016 Cochrane review “Current evidence does not support increasing the dose of inhaled corticosteroids (ICS) as part of a self-initiated action plan to treat exacerbations in adults and children with mild to moderate asthma.”^62^ | | PubMed | |
| 107 | Long-term effect of a watch and wait policy versus immediate systemic treatment for asymptomatic advanced-stage non-Hodgkin lymphoma: a randomised controlled trial Ardeshna KM, Et al | | Oncology | | 8/16/2003  LANCET | | Initial treatment protocols for patients with low-grade non-Hodgkin lymphoma vary but none seems to increase long-term disease-free survival.^65^ Immediate treatment of chemotherapy, combination chemotherapy, and chemotherapy plus radiation are often used, but retrospective studies have found that delaying treatment until disease progression may be equally as effective.^66^ This study looked to compared overall and cause-specific survival between a group given immediate systemic therapy with oral chlorambucil 10 mg per day continuously (n=158) with a group with an initial policy of observation (n=151). They found that overall survival and cause-specific survival did not differ between the two groups (median overall survival for oral chlorambucil 5.9 [range 0–17.8] years and for observation 6.7 [0.5–18.9] years, p=0.84; median cause-specific survival 9 [0–17.8] years and 9.1 [0.67–18.9] years, respectively p=0.44). This is a reversal of the policy of immediate systemic treatment of asymptomatic advanced-stage non-Hodgkin lymphoma. | | 2016. NIH Guidelines. "Follicular lymphoma has a long natural history. The conventional view is that apart from very localised disease which may be ablated by local radiotherapy there is no advantage in terms of survival for immediate treatment compared to a watch and wait approach."^67^ | | PubMed | |
| 108 | Effect of conjugate pneumococcal vaccine followed by polysaccharide pneumococcal vaccine on recurrent acute otitis media: a randomized study Veenhoven et al. | | Pediatrics | | 6/28/2003  LANCET | | The American Academy of Pediatrics has recommended immunization with 7-valent pneumococcal conjugate vaccine (PCV7) for children with recurrent or severe acute otitis media (AOM) and children who have tympanostomy tubes because of recurrent AOM^68^. This double-blind study randomized 383 patients, aged 1-7 years old, who had had two or more episodes of AOM in the year before entry. Subjects received either 7-valent pneumococcal conjugate vaccine follow by 23-valent pneumococcal polysaccharide vaccine, or hepatitis A or B vaccines. No difference was found in reduction of AOM episodes between the pneumococcal vaccine group compared to the control (Rate ratio 1.25 [95% CI 0.99 – 1.57]). This is a reversal of the use of recommendation of PCV7 for children with recurrent AOM. | | 2014. Cochran review. “Administering PCV7 in older children with a history of AOM appears to have no beneficial effect on preventing further AOM episodes..”^69^ | | PubMed | |
| 109 | Laparoscopic adhesiolysis in patients with chronic abdominal pain: a blinded randomized controlled multi-center trial Swank et al | | Gastroenterology/Hepatology | | 4/12/2003  LANCET | | Diagnostic laparoscopy is often used to identify specific intraabdominal pathology as the cause for chronic abdominal and pelvic pain.^70 71^ 100 patients who had diagnostic laparoscopy for chronic abdominal pain attributed to adhesions were randomized to either laparoscopic adhesiolysis (n=52) or no treatment (n=48). Pain was assessed for 1 year by visual analogue score, pain change score, use of analgesics, and quality of life score. Both groups reported substantial pain relief and significantly improved quality of life, with no difference between groups (mean change from baseline of VAS score at 12 months: difference 3 points, p=0.53; 95% CI −7 to 13). This is a reversal of laparoscopic adhesiolysis for patients with chronic abdominal pain. | | Study not included in this SR  2015 Langenbeck’s Archives of Surgery “The identified studies showed promising but preliminary results of laparoscopic adhesiolysis as a treatment of chronic abdominal pain. The evidence for laparoscopic adhesiolysis is not sufficient to make definitive conclusions.” | | Laparoscopic adhesiolysis, chronic abdominal pain | |
| 110 | Syndromic management of sexually-transmitted infections and behavior change interventions on transmission of HIV-1 in rural Uganda: a community randomized trial Kamali et al. | | Infectious disease | | 2/22/2003  LANCET | | The rise in HIV incidence during the 1980s led to different types of behavioral and educational programs being implemented at the institutional, community, and population-level, in an effort to reduce the transmission of HIV.^72^ The main methods of prevention against the pandemic of HIV-1 infection in sub-Saharan Africa are promotion of safer sexual behavior and treatment of sexually transmitted infections (STIs). This study randomized adults in three groups: one receiving behavioral interventions alone (The information, education, and communication intervention was based on the behavioral change for interventions model, n=6918), behavioral interventions and STI interventions(STI intervention was implemented by training health workers in both government and private health units in syndromic management of STIs, n=6856), or routine government health services and community development activities (n=6742). All groups received condoms and HIV counseling and testing. They found no difference in HIV-1 incidence rate between any of the groups. This is a reversal of two practices: 1) the treatment of STIs for prevention of HIV-1, and 2) of the Information, Education, and Communication program (drama and video shows, discussions and meetings), which promotes HIV prevention beyond social marketing of condom use and HIV counseling and testing in rural Uganda. | | 2014. Cochran review. “There is no clear evidence that structural interventions at the community level to increase condom use prevent the transmission of HIV and other STIs.”^73^ | | PubMed | |
| 111 | Admission cardiotocography: a randomized controlled trial Impey et al | | Obstetrics and Gynecology | | 2/8/2003  LANCET | | Cardiotocography was introduced as a screening test during the 1970s as a way to detect fetal abnormalities and reduce perinatal mortality, and became standard practice within several decades.^74^ This study randomized 8580 women admitted to the delivery ward of a Dublin teaching hospital who were at low risk of fetal distress in labor to either admission cardiotocography (20 min) (n=4298) or the unit’s usual care (intermittent auscultation only, with continuous cardiotocography only if clinically indicated) (n=4282). The primary endpoint (moderate to severe neonatal morbidity, or perinatal mortality) occurred in 56 (1.3%) of the women assigned to cardiotocography, and 55 (1.3%) in the usual-care group (relative risk 1.01 [95% CI 0.70 – 1.47]). This study does not support the routine use of cardiotocography as a screening test to detect fetal abnormalities, and as such is a reversal. | | 2017. Cochran review. “Contrary to continued use in some clinical areas, we found no evidence of benefit for the use of the admission CTG [admission cardiotocograph] for low-risk women on admission in labour. Furthermore, the probability is that admission CTG increases the caesarean section rate by approximately 20%.”^75^ | | PubMed | |
| 112 | Comparison of intermittent and continuous palliative chemotherapy for advanced colorectal cancer: a multicenter randomized trial Maughan et al. | | Oncology | | 2/8/2003  LANCET | | Colorectal cancer is a common cancer with a wide array of treatment/management practices. At the time of this study, some physicians reported prescribing chemotherapy for about 3 months, many (47%) reported prescribing chemotherapy for 6 months, and 20% reported prescribing chemotherapy indefinitely to patients with stable or responding disease.^76^ 354 patients, who responded or had stable disease after receiving 12 weeks of the regimens described by de Gramont and Lokich, or raltitrexed chemotherapy, across 42 centers in the UK were randomized to either intermittent (n=178) or continuous (n=176) chemotherapy. Median time on treatment after restarting was 84 days for the intermittent group and 92 days for the continuous groups. The intermittent group had significantly fewer toxic effects and series adverse events compared to the continuous group. Both groups had similar proportions of patients who received second-line therapy, and there was no evidence of a difference in overall survival. This is a reversal of the use of continuous palliative chemotherapy for advanced colorectal cancer. | | 2014. Annals of Oncology. “There were no statistically significant survival differences observed between the continuous and intermittent chemotherapy strategies.”^77^ | | PubMed | |
| 113 | Intrahepatic arterial versus intravenous fluorouracil and folinic acid for colorectal cancer liver metastases: a multicentre randomised trial Kerr et al. | | | Oncology | | 2/1/2003  Lancet | | Colon cancer, one of the most common types of cancer, has a relapse rate, after surgery, of about 50%, with the liver being a common site for metastasis.^78^ Intrahepatic arterial infusion (IHA) has been used as a method of delivering chemotherapy because it is thought that there would be a higher dose of chemotherapy to cancer cells, while lessoning the side-effects of chemotherapy.^79 80^ This trial randomly allocated 290 patients from 16 centers to receive either intravenous chemotherapy (folinic acid 200 mg/m[2](https://www.sciencedirect.com/science/article/pii/S0140673603123884#bib2), fluorouracil bolus 400 mg[2](https://www.sciencedirect.com/science/article/pii/S0140673603123884#bib2) and 22-h infusion 600 mg/m[2](https://www.sciencedirect.com/science/article/pii/S0140673603123884#bib2), day 1 and 2, repeated every 14 days) or IHA chemotherapy designed to be equitoxic (folinic acid 200 mg/m[2](https://www.sciencedirect.com/science/article/pii/S0140673603123884#bib2), fluorouracil 400 mg/m[2](https://www.sciencedirect.com/science/article/pii/S0140673603123884#bib2) over 15 mins and 22-h infusion 1600 mg/m[2](https://www.sciencedirect.com/science/article/pii/S0140673603123884#bib2), day 1 and 2, repeated every 14 days). Median survival in the IHA group was 14.7 months and was 14.8 months in the intravenous group (hazard ratio 1.04 [95% CI 0.80 – 1.33]). This is a reversal of the use of IHA for patients with colorectal cancer liver metastases. | | 2009. Cochrane review. “Currently available evidence does not support the clinical or investigational use of ﬂuoropyrimidine-based HAI alone f or the treatment of patients with unresectable CRC liver metastases: in fact, the greater tumor response rate obtained with this IHA regimen does not translate into a survival advantage over ﬂuoropyrimidine alone SCT.”^81^ | | PubMed | |

1. American Cancer Society. History of ACS Recommendations for the Early Detection of Cancer in People Without Symptoms 2014 [updated 2017].

2. Gotzsche PC, Jorgensen KJ. Screening for breast cancer with mammography. *Cochrane Database Syst Rev* 2013(6):Cd001877. doi: 10.1002/14651858.CD001877.pub5 [published Online First: 2013/06/06]

3. Cook TM, Protheroe RT, Handel JM. Tetanus: a review of the literature. *BJA: British Journal of Anaesthesia* 2001;87(3):477-87. doi: 10.1093/bja/87.3.477

4. Sutton D, Tremlett M, Woodcock T, et al. Management of autonomic dysfunction in severe tetanus: the use of magnesium sulphate and clonidine. *Intensive care medicine* 1990;16(2):75-80.

5. Rodrigo C, Samarakoon L, Fernando SD, et al. A meta-analysis of magnesium for tetanus. *Anaesthesia* 2012;67(12):1370-74. doi: 10.1111/anae.12020

6. Biller J, Feinberg WM, Castaldo JE, et al. Guidelines for carotid endarterectomy: a statement for healthcare professionals from a special writing group of the Stroke Council, American Heart Association. *Circulation* 1998;97(5):501-09.

7. John TA, Gough MJ. A late complication of internal carotid artery stenting. *Journal of Vascular Surgery* 1998;27(4):753-55. doi: <https://doi.org/10.1016/S0741-5214(98)70244-7>

8. Meier P, Knapp G, Tamhane U, et al. Short term and intermediate term comparison of endarterectomy versus stenting for carotid artery stenosis: systematic review and meta-analysis of randomised controlled clinical trials. *BMJ* 2010;340:c467.

9. Martinez FD, Wright AL, Taussig LM, et al. Asthma and wheezing in the first six years of life. *New England Journal of Medicine* 1995;332(3):133-38.

10. Strachan DP, Butland BK, Anderson HR. Incidence and prognosis of asthma and wheezing illness from early childhood to age 33 in a national British cohort. *BMJ : British Medical Journal* 1996;312(7040):1195-99.

11. Holt S, Suder A, Weatherall M, et al. Dose-response relation of inhaled fluticasone propionate in adolescents and adults with asthma: meta-analysis. *BMJ : British Medical Journal* 2001;323(7307):253-53.

12. Kierdorf HP, Sieberth HG. Continuous renal replacement therapies versus intermittent hemodialysis in acute renal failure: What do we know? *American Journal of Kidney Diseases*;28(5):S90-S96. doi: 10.1016/S0272-6386(96)90085-2

13. Rabindranath K, Adams J, Macleod AM, et al. Intermittent versus continuous renal replacement therapy for acute renal failure in adults. *Cochrane Database Syst Rev* 2007(3):Cd003773. doi: 10.1002/14651858.CD003773.pub3 [published Online First: 2007/07/20]

14. Homburg R, Howles CM. Low-dose FSH therapy for anovulatory infertility associated with polycystic ovary syndrome: rational, results, reflections refinements. *Human Reproduction Update* 1999;5(5):493-99. doi: 10.1093/humupd/5.5.493

15. Veltman-Verhulst SM, Hughes E, Ayeleke RO, et al. Intra-uterine insemination for unexplained subfertility. *Cochrane Database Syst Rev* 2016;2:Cd001838. doi: 10.1002/14651858.CD001838.pub5 [published Online First: 2016/02/20]

16. Hurley KF, Magee K, Green R. Aminophylline for bradyasystolic cardiac arrest in adults. *Cochrane Database Syst Rev* 2015(11):Cd006781. doi: 10.1002/14651858.CD006781.pub3 [published Online First: 2015/11/26]

17. Richard A Harrigan and William J Brady. The Clinical Challenge of Bradycardia: Diagnosis, Evaluation, and Intervention in the Emergency Department 2000 [Available from: <https://www.ahcmedia.com/articles/58758-the-clinical-challenge-of-bradycardia-diagnosis-evaluation-and-intervention>.

18. Abu-Laban RB, McIntyre CM, Christenson JM, et al. Aminophylline in bradyasystolic cardiac arrest: a randomised placebo-controlled trial. *The Lancet*;367(9522):1577-84. doi: 10.1016/S0140-6736(06)68694-7

19. Stoltzfus RJ, Dreyfuss ML. Guidelines for the use of iron supplements to prevent and treat iron deficiency anemia: Ilsi Press Washington, DC 1998.

20. Sazawal S, Black RE, Ramsan M, et al. Effects of routine prophylactic supplementation with iron and folic acid on admission to hospital and mortality in preschool children in a high malaria transmission setting: community-based, randomised, placebo-controlled trial. *The Lancet* 2006;367(9505):133-43.

21. Neuberger A, Okebe J, Yahav D, et al. Oral iron supplements for children in malaria‐endemic areas. *The Cochrane Library* 2016.

22. Trnka L, Dankova D, Zitova J, et al. Survey of BCG vaccination policy in Europe: 1994-96. *Bulletin of the World Health Organization* 1998;76(1):85.

23. Robin ED. Death by pulmonary artery flow-directed catheter. Time for a moratorium? *Chest* 1987;92(4):727-31. [published Online First: 1987/10/01]

24. Rajaram SS, Desai NK, Kalra A, et al. Pulmonary artery catheters for adult patients in intensive care. *Cochrane Database Syst Rev* 2013(2):Cd003408. doi: 10.1002/14651858.CD003408.pub3 [published Online First: 2013/03/02]

25. Lyczak JB, Cannon CL, Pier GB. Lung Infections Associated with Cystic Fibrosis. *Clinical Microbiology Reviews* 2002;15(2):194-222. doi: 10.1128/CMR.15.2.194-222.2002

26. Chernish RN, Aaron SD. Approach to resistant gram-negative bacterial pulmonary infections in patients with cystic fibrosis. *Current opinion in pulmonary medicine* 2003;9(6):509-15. [published Online First: 2003/10/10]

27. Waters V, Ratjen F. Combination antimicrobial susceptibility testing for acute exacerbations in chronic infection of Pseudomonas aeruginosa in cystic fibrosis. *Cochrane Database Syst Rev* 2017;6:Cd006961. doi: 10.1002/14651858.CD006961.pub4 [published Online First: 2017/06/20]

28. Everitt H, Little P. How do GPs diagnose and manage acute infective conjunctivitis? A GP survey. *Fam Pract* 2002;19(6):658-60. [published Online First: 2002/11/14]

29. Jefferis J, Perera R, Everitt H, et al. Acute infective conjunctivitis in primary care: who needs antibiotics? An individual patient data meta-analysis. *Br J Gen Pract* 2011;61(590):e542-8. doi: 10.3399/bjgp11X593811 [published Online First: 2011/12/14]

30. Jones A, Cahill D, Gardham R. Outcome in patients with a large abdominal aortic aneurysm considered unfit for surgery. *British Journal of Surgery* 1998;85(10):1382-84. doi: doi:10.1046/j.1365-2168.1998.00947.x

31. Zarins CK, Wolf YG, Lee WA, et al. Will endovascular repair replace open surgery for abdominal aortic aneurysm repair? *Annals of surgery* 2000;232(4):501.

32. Paravastu SC, Jayarajasingam R, Cottam R, et al. Endovascular repair of abdominal aortic aneurysm. *Cochrane Database Syst Rev* 2014(1):Cd004178. doi: 10.1002/14651858.CD004178.pub2 [published Online First: 2014/01/24]

33. Introduction of the medical emergency team (MET) system: a cluster-randomised controlled trial. *The Lancet* 2005;365(9477):2091-97. doi: <https://doi.org/10.1016/S0140-6736(05)66733-5>

34. Kenward G, Castle N, Hodgetts T, et al. Evaluation of a medical emergency team one year after implementation. *Resuscitation* 2004;61(3):257-63.

35. Daly FFS, Sidney KL, Fatovich DM. The Medical Emergency Team (MET): a model for the district general hospital. *Australian and New Zealand Journal of Medicine* 1998;28(6):795-98. doi: doi:10.1111/j.1445-5994.1998.tb01556.x

36. Chan PS, Jain R, Nallmothu BK, et al. Rapid response teams: a systematic review and meta-analysis. *Archives of internal medicine* 2010;170(1):18-26.

37. Intakes IoMSCotSEoDR. Dietary reference intakes for calcium, phosphorus, magnesium, vitamin D, and fluoride: National Academies Press (US) 1997.

38. Murray TM. Prevention and management of osteoporosis: consensus statements from the Scientific Advisory Board of the Osteoporosis Society of Canada. 4. Calcium nutrition and osteoporosis. *CMAJ: Canadian Medical Association Journal* 1996;155(7):935-39.

39. McKercher H, Crilly R, Kloseck M. Osteoporosis management in long-term care. Survey of Ontario physicians. *Canadian Family Physician* 2000;46(11):2228-35.

40. Avenell A, Mak JC, O'Connell D. Vitamin D and vitamin D analogues for preventing fractures in post-menopausal women and older men. *Cochrane Database Syst Rev* 2014(4):Cd000227. doi: 10.1002/14651858.CD000227.pub4 [published Online First: 2014/04/15]

41. REPINE JE, BAST A, LANKHORST I, et al. Oxidative Stress in Chronic Obstructive Pulmonary Disease. *American Journal of Respiratory and Critical Care Medicine* 1997;156(2):341-57. doi: 10.1164/ajrccm.156.2.9611013

42. Stey C, Steurer J, Bachmann S, et al. The effect of oral N-acetylcysteine in chronic bronchitis: a quantitative systematic review. *European Respiratory Journal* 2000;16(2):253-62.

43. Criner GJ, Bourbeau J, Diekemper RL, et al. Prevention of Acute Exacerbations of COPD: American College of Chest Physicians and Canadian Thoracic Society Guideline. *Chest* 2015;147(4):894-942. doi: <https://doi.org/10.1378/chest.14-1676>

44. Dávalos A, Ricart W, Gonzalez-Huix F, et al. Effect of Malnutrition After Acute Stroke on Clinical Outcome. *Stroke* 1996;27(6):1028-32. doi: 10.1161/01.str.27.6.1028

45. Goodhall L. Tube feeding dilemmas: can artificial nutrition and hydration be legally or ethically withheld or withdrawn? *Journal of Advanced Nursing* 1997;25(2):217-22.

46. Sullivan DH, Sun S, Walls RC. Protein-energy undernutrition among elderly hospitalized patients: A prospective study. *JAMA* 1999;281(21):2013-19. doi: 10.1001/jama.281.21.2013

47. Milne AC, Potter J, Vivanti A, et al. Protein and energy supplementation in elderly people at risk from malnutrition. *Cochrane Database Syst Rev* 2009(2):Cd003288. doi: 10.1002/14651858.CD003288.pub3 [published Online First: 2009/04/17]

48. Mckissock W, Richardson A, Taylor J. Primary intracerebral haemorrhage: a controlled trial of surgical and conservative treatment in 180 unselected cases. *The Lancet* 1961;278(7196):221-26.

49. Auer LM, Deinsberger W, Niederkorn K, et al. Endoscopic surgery versus medical treatment for spontaneous intracerebral hematoma: a randomized study. *J Neurosurg* 1989;70(4):530-5. doi: 10.3171/jns.1989.70.4.0530 [published Online First: 1989/04/01]

50. Kaneko M, Tanaka K, Shimada T, et al. Long-term evaluation of ultra-early operation for hypertensive intracerebral hemorrhage in 100 cases. *J Neurosurg* 1983;58(6):838-42. doi: 10.3171/jns.1983.58.6.0838 [published Online First: 1983/06/01]

51. Prasad K, Mendelow AD, Gregson B. Surgery for primary supratentorial intracerebral haemorrhage. *The Cochrane Library* 2008

52. Koo B. Vigabatrin in the treatment of infantile spasms. *Pediatric Neurology* 1999;20(2):106-10. doi: <https://doi.org/10.1016/S0887-8994(98)00116-7>

53. Baharoglu MI, Cordonnier C, Salman RA-S, et al. Platelet transfusion versus standard care after acute stroke due to spontaneous cerebral haemorrhage associated with antiplatelet therapy (PATCH): a randomised, open-label, phase 3 trial. *The Lancet* 2016;387(10038):2605-13.

54. Hancock EC, Osborne JP, Edwards SW. Treatment of infantile spasms. *Cochrane Database Syst Rev* 2013(6):Cd001770. doi: 10.1002/14651858.CD001770.pub3 [published Online First: 2013/06/07]

55. Ghajar J, Hariri RJ, Narayan RK, et al. Survey of critical care management of comatose, head-injured patients in the United States. *Crit Care Med* 1995;23(3):560-7. [published Online First: 1995/03/01]

56. Jeevaratnam DR, Menon DK. Survey of intensive care of severely head injured patients in the United Kingdom. *BMJ : British Medical Journal* 1996;312(7036):944-47.

57. Alderson P, Roberts I. Corticosteroids for acute traumatic brain injury. *Cochrane Database Syst Rev* 2005(1):Cd000196. doi: 10.1002/14651858.CD000196.pub2 [published Online First: 2005/01/28]

58. Wiswell TE, Tuggle JM, Turner BS. Meconium aspiration syndrome: have we made a difference? *Pediatrics* 1990;85(5):715-21.

59. Vain NE, Szyld EG, Prudent LM, et al. Oropharyngeal and nasopharyngeal suctioning of meconium-stained neonates before delivery of their shoulders: multicentre, randomised controlled trial. *The Lancet* 2004;364(9434):597-602. doi: <https://doi.org/10.1016/S0140-6736(04)16852-9>

60. Foster JP, Dawson JA, Davis PG, et al. Routine oro/nasopharyngeal suction versus no suction at birth. *Cochrane Database Syst Rev* 2017;4:Cd010332. doi: 10.1002/14651858.CD010332.pub2 [published Online First: 2017/04/19]

61. Harrison TW, Oborne J, Newton S, et al. Doubling the dose of inhaled corticosteroid to prevent asthma exacerbations: randomised controlled trial. *The Lancet* 2004;363(9405):271-75. doi: <https://doi.org/10.1016/S0140-6736(03)15384-6>

62. Kew KM, Quinn M, Quon BS, et al. Increased versus stable doses of inhaled corticosteroids for exacerbations of chronic asthma in adults and children. *Cochrane Database Syst Rev* 2016(6):Cd007524. doi: 10.1002/14651858.CD007524.pub4 [published Online First: 2016/06/09]

63. McKean MC, Ducharme F. Inhaled steroids for episodic viral wheeze of childhood. *The Cochrane Library* 2000

64. Vuillermin P, South M, Robertson C. Parent-initiated oral corticosteroid therapy for intermittent wheezing illnesses in children. *Cochrane Database Syst Rev* 2006(3):Cd005311. doi: 10.1002/14651858.CD005311.pub2 [published Online First: 2006/07/21]

65. Horning SJ. Follicular lymphoma: have we made any progress? *Annals of oncology : official journal of the European Society for Medical Oncology* 2000;11 Suppl 1:23-7. [published Online First: 2000/03/09]

66. O'Brien ME, Easterbrook P, Powell J, et al. The natural history of low grade non-Hodgkin's lymphoma and the impact of a no initial treatment policy on survival. *The Quarterly journal of medicine* 1991;80(292):651-60. [published Online First: 1991/08/01]

67. National Guideline A. National Institute for Health and Care Excellence: Clinical Guidelines. Non-Hodgkin's Lymphoma: Diagnosis and Management. London: National Institute for Health and Care Excellence (UK)

Copyright (c) National Institute for Health and Care Excellence 2016. 2016.

68. American Academy of Pediatrics. Committee on Infectious Diseases. Policy statement: recommendations for the prevention of pneumococcal infections, including the use of pneumococcal conjugate vaccine (Prevnar), pneumococcal polysaccharide vaccine, and antibiotic prophylaxis. *Pediatrics* 2000;106(2 Pt 1):362-6. [published Online First: 2000/08/02]

69. Fortanier AC, Venekamp RP, Boonacker CW, et al. Pneumococcal conjugate vaccines for preventing otitis media. *Cochrane Database Syst Rev* 2014(4):Cd001480. doi: 10.1002/14651858.CD001480.pub4 [published Online First: 2014/04/04]

70. Freys S, Fuchs K, Heimbucher J, et al. Laparoscopic adhesiolysis. *Surgical endoscopy* 1994;8(10):1202-07.

71. Klingensmith M, Soybel D, Brooks D. Laparoscopy for chronic abdominal pain. *Surgical endoscopy* 1996;10(11):1085-87.

72. Council NR. Preventing and mitigating AIDS in Sub-Saharan Africa: Research and data priorities for the social and behavioral sciences: National Academies Press 1996.

73. Moreno R, Nababan HY, Ota E, et al. Structural and community-level interventions for increasing condom use to prevent the transmission of HIV and other sexually transmitted infections. *Cochrane Database Syst Rev* 2014(7):Cd003363. doi: 10.1002/14651858.CD003363.pub3 [published Online First: 2014/07/30]

74. Goddard R. Electronic fetal monitoring : Is not necessary for low risk labours. *BMJ : British Medical Journal* 2001;322(7300):1436-37.

75. Devane D, Lalor JG, Daly S, et al. Cardiotocography versus intermittent auscultation of fetal heart on admission to labour ward for assessment of fetal wellbeing. *Cochrane Database Syst Rev* 2017;1:Cd005122. doi: 10.1002/14651858.CD005122.pub5 [published Online First: 2017/01/27]

76. Seymour MT, Stenning SP, Cassidy J. Attitudes and practice in the management of metastatic colorectal cancer in Britain. *Clinical Oncology* 1997;9(4):248-51. doi: <https://doi.org/10.1016/S0936-6555(97)80010-6>

77. Berry S, Group CCOsGDS, Cosby R, et al. Continuous versus intermittent chemotherapy strategies in metastatic colorectal cancer: a systematic review and meta-analysis. *Annals of Oncology* 2014;26(3):477-85.

78. Midgley R, Kerr D. Colorectal cancer. *Lancet* 1999;353(9150):391-9. doi: 10.1016/s0140-6736(98)07127-x [published Online First: 1999/02/09]

79. Ansfield FJ, Ramirez G, Skibba JL, et al. Intrahepatic arterial infusion with 5‐fluorouracil. *Cancer* 1971;28(5):1147-51.

80. Fortner JG, Silva JS, Golbey RB, et al. Multivariate analysis of a personal series of 247 consecutive patients with liver metastases from colorectal cancer. I. Treatment by hepatic resection. *Annals of surgery* 1984;199(3):306.

81. Mocellin S, Pasquali S, Nitti D. Fluoropyrimidine‐HAI (hepatic arterial infusion) versus systemic chemotherapy (SCT) for unresectable liver metastases from colorectal cancer. *The Cochrane Library* 2009

| **#** | **Article and**  **Authors** | **Primary Medical Discipline** | **Date and Journal** | **Summary** | **Systematic Review** | **Systematic Review Search Terms** |
| --- | --- | --- | --- | --- | --- | --- |
| 309 | Lansoprazole for Children With Poorly Controlled Asthma A Randomized Controlled Trial  Writing Committee for the American Lung Association Asthma Clinical Research Centers | Pulmonary | 1/25/2012 JAMA | Children with asthma who frequently display symptoms are generally treated with proton pump inhibitors (PPI) for gastroesophageal reflux (GER). The use of PPIs has increased dramatically in the United States with a reported 5% of children in the US being on the medication in 2009.^1^ This study compared asthma symptoms in children without symptoms of GER who were prescribed lansoprazole (n=149), a type of PPI, or placebo (n=157). This trial found that in children with poorly controlled asthma without symptoms of GER, the addition of lansoprazole as compared to placebo did not improve asthma symptoms. The mean difference in change in Asthma Control Questionnaire scores between lansoprazole and placebo groups was 0.2 units (95% CI, -0.0 to 0.3; P=.12). This is a reversal of lansoprazole for improving asthma symptoms in children with poorly controlled asthma. | None found | lansoprazole, ashtma outcomes, GER, children |
| 310 | Amoxicillin for Acute Rhinosinusitis A Randomized Controlled Trial  Garbutt et al. | Public health/ Preventive medicine | 2/15/2012 JAMA | Sinusitis is one of the most common medical diagnoses and has led to 25 million US physician office visits in 1995.^2^ Antibiotics were prescribed in adults 60-70% at the time, and in children 70-80% of the time, even though this condition is often viral.^3^ This study provides further evidence of the ineffectiveness of general antibiotic use for those with acute rhinosinusitis. A 10-day course of amoxicillin (n=81) did not improve disease-specific quality of life compared to placebo (n=74) after 3 days (difference in symptom improvement, 37% for amoxicillin group vs 34% for placebo group; P=.67) and 10 days of treatment (78% vs 80%; P=.71). This is a reversal of amoxicillin for treatment of acute rhinosinusitis. | 2012. Cochrane review. “The potential benefit of antibiotics in the treatment of clinically diagnosed acute rhinosinusitis needs to be seen in the context of a high prevalence of adverse events. Taking into account antibiotic resistance and the very low incidence of serious complications, we conclude that there is no place for antibiotics for the patient with clinically diagnosed, uncomplicated acute rhinosinusitis.”^4^ | Pubmed found systematic review |
| 311 | Intracoronary Abciximab and Aspiration Thrombectomy in Patients With Large Anterior Myocardial Infarction The INFUSE-AMI Randomized Trial  Stone et al. | Cardiovascular | 5/02/2012 JAMA | Percutaneous coronary intervention (PCI) is widely used for patients with ST-segment elevation myocardial infarction (STEMI). Aspiration of a thrombus formed during PCI is recommended as part of primary PCI to prevent thrombus embolization and has become standard practice after several randomized controlled trials showed efficacy in the procedure.^5 6^ This trial investigated with effects of manual aspiration and bolus intracoronary abciximab in patients with STEMI undergoing PCI. They found that while abciximab (n=188) was effective at reducing infarct size at 30 days compared to no abciximab (n=184), aspiration thrombectomy (median, 17.0%; 95% CI, 9.0-22.8; n=174) did not improve infarct size at 30 days compared to no aspiration thrombectomy (median, 17.3%; 95% CI, 7.1-25.5; n=179; P=.51). This is a reversal of aspiration thrombectomy for patients with STEMI undergoing PCI. | 2014. “The present meta-analysis suggested that there was no evidence that using manual thrombus aspiration in patients with STEMI could provide distinct benefits in long-term clinical outcomes.”^7^ | Pubmed found systematic review |
| 312 | Effect of Continuous Positive Airway Pressure on the Incidence of Hypertension and Cardiovascular Events in Nonsleepy Patients With Obstructive Sleep Apnea A Randomized Controlled Trial  Barbe et al. | Pulmonary | 5/23/2012+ 5/30/2012 JAMA | Obstructive sleep apnea (OSA), which is a partial or complete collapse of the airway during sleep, has been estimated to affect between 2-7% of adults.^8^ Continuous positive airway pressure (CPAP) machines were first introduced in 1981 for people with OSA, and are now standard of care.^9^ In the early 2000's there was increased interest in CPAPs being used to prevent cardiovascular disease, even among patients with minimal symptoms.^10^ In this trial, use of CPAP (n=357), compared with usual care (n=366), did not lead to a reduction in the incidence rate of systemic hypertension or cardiovascular events in patients with OSA without daytime sleepiness (9.20 per 100 person-years for CPAP group vs 11.02 per 100 person-years for control group; incidence density ratio, 0.83; 95% CI, 0.63-1.1; P=.20). This is a reversal of CPAP for reducing incidence of hypertension and cardiovascular events in nonsleepy patients with OSA. | “Although CPAP treatment reduces OSA severity and sleepiness, it seems not to have a beneficial effect on BP in patients with minimally symptomatic OSA....”^11^ | Pubmed found systematic review |
| 313 | Effect of Silymarin (Milk Thistle) on Liver Disease in Patients With Chronic Hepatitis C Unsuccessfully Treated With Interferon Therapy A Randomized Controlled Trial  Fried et al. | Gastroenterology/Hepatology | 7/18/2012 JAMA | Silymarin (Silybum marianum), an extract from milk thistle, is often used as an alternative treatment for liver health, and is thought to be hepatoprotective.^12^ With this in mind, silymarin is often used by people with hepatitis C virus (HCV) infection - as many as 33% of these patients.^13^ While the extract has been shown to have hepatoprotective and immunomodulatory effects in vitro,^14^ clinical trials have shown mixed results in its efficacy.^15^ In this randomized controlled trial, 420-mg silymarin (n=50) and 700-mg silymarin (n=52) were found to be no better at reducing the liver enzyme alanine aminotransferase (ALT) than placebo (n=52). Only 2 participants from each group reached a serum level of 45 U/L or less or less than 65 U/L, provided that is was at least a 50% decline from baseline values (P≥.99). This is a reversal of silymarin as a hepatoprotective agent for patients with chronic HCV. Levels of ALT was used as a surrogate outcome for hepatoprotection in this study. | 2014. “Silymarin is well tolerated in chronic HCV-infected patients. However, no evidence of salutary effects of oral silymarin has yet been reported based on intermediate endpoints (ALT and HCV RNA) in this population. Moreover, intravenous administration of silymarin should be further studied.”^16^ | Pubmed found systematic review |
| 314 | Effect of Screening for Partner Violence on Women's Quality of Life A Randomized Controlled Trial  Klevens et al. | Public health/ Preventive medicine | 8/15/2012 JAMA | Screening for intimate partner violence (IPV) in the clinical setting has been recommended by several organizations,^17^ even though the USPSTF determined that there was insufficient evidence for its effectiveness.^18^ Computerized screening has been implemented as a way to increase the number of people who report IPV.^19^ In this trial, computerized screening for IPV plus a list of partner violence resources (n=801) did not increase patients' quality of life one year follow-up compared to partner-violence resource list only (n= 772) or a control of no screening or list (n=791). At 1-year follow-up, there were no significant differences in the QOL physical health component between the screen plus partner violence resource list group (mean score, 46.8; 95% CI, 46.1-47.4), the partner violence resource list only group (mean score, 46.4; 95% CI, 45.8-47.1), and the control group (mean score, 47.2; 95% CI, 46.5-47.8). This is a reversal of screening for partner violence in clinical settings for better quality of life in women. | 2015. Cochrane review. “Thus, while screening increases identification, there is insufficient evidence to justify screening in healthcare settings. Furthermore, there remains a need for studies comparing universal screening to case-finding (with or without advocacy or therapeutic interventions) for women's long-term wellbeing in order to inform IPV identification policies in healthcare settings.”^20^ | Pubmed found systematic review |
| 315 | Multivitamins in the Prevention of Cardiovascular Disease in Men The Physicians' Health Study II Randomized Controlled Trial  Sesso et al. | Public health/ Preventive medicine | 11/07/2012 JAMA | In the 1990s, when this randomized controlled study began, about one out of every 5 adults took a multi-vitamin, partly driven by a belief that multivitamins affected disease outcomes.^21^ And, while the rate of multi-vitamin prescribing practices was low among physicians, about 10% of the prescriptions were related to cardiovascular disease.^22^ In this trial of US male physicians, a daily multivitamin supplement (n=7317) did not reduce a composite of major cardiovascular events, including MI, stroke, and CVD mortality compared to placebo (n=7324). Rate of major cardiovascular events was 11.0 events per 1000 person-years in the multivitamin group vs 10.8 events/1000 person-years in the placebo group (HR, 1.01; 95% CI, 0.91-1.10; P=.91). This is a reversal of multivitamins for preventing cardiovascular disease in men. | 2017. “Taken together, we found insufficient evidence to support the use of dietary supplements in the primary prevention of cause-specific death, incidence of CVD, and incidence of cancer.”^23^ | Pubmed found systematic review |
| 316 | Intraoperative High-Dose Dexamethasone for Cardiac Surgery A Randomized Controlled Trial  Dieleman et al. | Cardiovascular | 11/07/2012 JAMA | Corticosteroids are thought to be beneficial in cardiac surgery because they have shown to decrease inflammatory markers and improve pulmonary gas exchange, potentially reducing inflammation and administration of postoperative inotropic agents .^24^ Corticosteroid use in cardiac surgery is more common in Europe countries than the US, where it is not routinely used.^25^ In this randomized controlled trial, the use of dexamethasone, a long-lasting corticosteroid, (n=2235) in patients undergoing cardiac surgery did not reduce 30-day incidence of major adverse events compared to placebo (n=2247). A composite of death, myocardial infarction, stroke, renal failure, or respiratory failure was seen in 7.0% patients in the dexamethasone group and 8.5% patients in the placebo group (RR, 0.83; 95% CI, 0.67-1.01; absolute RR, -1.5%; 95% CI, -3.0% to 0.1%; P=.07). This is a reversal of high-dose dexamethasone for improving outcomes in patients undergoing cardiac surgery. | 2014. “Evidence does not equivocally support the use of corticosteroids to improve clinical outcomes in cardiac surgery patients.”^26^ | Google search “cardiac surgery corticosteroid systematic review” |
| 317 | Effect of Citicoline on Functional and Cognitive Status Among Patients With Traumatic Brain Injury Citicoline Brain Injury Treatment Trial (COBRIT)  Zafonte et al. | Neurology | 11/21/2012 JAMA | Traumatic brain injury (TBI) is a serious public health problem in the United States, yet no treatment is currently available to improve patient outcomes after TBI. Recognized in 1956 as the intermediate element in the biosynthesis of phosphatidylcholine (a key constituent of neuronal membranes), citicoline may have a pleiotropic range of neuroprotective properties, and is an approved therapy for TBI in 59 countries.^27^ This study found that among patients with TBI, the use of citicoline (n=508) compared with placebo (n=509) did not result in improvement in functional and cognitive status. At 90 days, the citicoline and placebo groups had similar TBI-Clinical Trials Network Core Battery scores (OR, 0.98; 95% CI, 0.83-1.15), with favorable improvement rates at 35.4% and 35.6%, respectively. This is a reversal of citicoline for improving functional and cognitive status in patients with TBI. | 2017. “The available evidence doesn’t support current routine use of Citicoline for acute TBI management. Citicoline use for managing impaired neuro-cognitive conditions in chronic TBI patients is weak and needs further research.”^28^ | Pubmed found systematic review |
| 318 | Fish Oil and Postoperative Atrial Fibrillation The Omega-3 Fatty Acids for Prevention of Post-operative Atrial Fibrillation (OPERA) Randomized Trial  Mozaffarian et al. | Cardiovascular | 11/21/2012 JAMA | Omega-3 fatty acids from fish oil have been shown to be beneficial in treating atrial fibrillation (AF) in animals, and observational studies have produced similar findings.^29^ This benefit may be due to their inhibition of fast, voltage-dependent sodium channels.^30^ Because of previous findings in research (both animal and human studies), some physicians have published recommendations for the supplementation of 800-1000 mg/day of EPA and DHA to help prevent AF, a condition common after cardiac surgery.^30^ In the OPERA trial, there was no improvement in postoperative AF in those supplemented with omega-3 fatty acids (n=758) compared to placebo (n=758). Postoperative AF lasting longer than 30 seconds occurred in 30.0% of patients in the omega-3 group and 30.7% of patients in the placebo group (OR, 0.96; 95% CI, 0.77-1.20; P=.74). This is a reversal of fish oil for preventing post-operative AF. | 2013 “Published clinical trials do not support n-3 PUFAs as agents aimed at preventing either postoperative or recurrent AF.”^31^ | Pubmed found systematic review |
| 319 | Behavioral Therapy With or Without Biofeedback and Pelvic Floor Electrical Stimulation for Persistent Postprostatectomy Incontinence A Randomized Controlled Trial  Goode et al. | Urology | 1/12/2011 JAMA | One in 6 men are at risk of prostate cancer, and prostatectomies are a common treatment for the cancer, although adverse events from these surgical procedures are common.^32^ Urinary incontinence occurs in as many as 65% of men after radical prostatectomy.^33^ Biofeedback and pelvic floor electrical stimulation (PFES) are often used together to enhance the effectiveness of behavioral therapy for the treatment of urinary incontinence in this population.^34^ This study aimed to evaluate the effectiveness of behavioral therapy for reducing persistent postprostatectomy incontinence and to determine whether the technologies of biofeedback and pelvic floor electrical stimulation enhance the effectiveness of behavioral therapy. They found that after 8 weeks, the behavioral therapy group (n=70) and the behavioral therapy plus biofeedback and PFES group (n=70) compared with the delayed-treatment control (n=64) resulted in fewer incontinence episodes in patients with urinary incontinence (55% reduction in behavioral group; 51% reduction in behavioral plus biofeedback and PFES group; P=.001 for both groups). However, the addition of biofeedback and pelvic floor electrical stimulation did not result in greater effectiveness (P=.69). While behavioral therapy showed efficacy, the technological additions of biofeedback and PFES had no effect on incontinence. This is a reversal of biofeedback and PFES for postprostectomy incontinence. | 2015. Cochrane review. “This systematic review found insufficient evidence to state whether or not there were additional effects by adding PFMT to other active treatments when compared with the same active treatment alone for urinary incontinence (SUI, UUI or MUI) in women. These results should be interpreted with caution as most of the comparisons were investigated in small, single trials. None of the trials in this review were large enough to provide reliable evidence. Also, none of the included trials reported data on adverse events associated with the PFMT regimen, thereby making it very difficult to evaluate the safety of PFMT.”^35^ | Pubmed found systematic review |
| 320 | Adjunctive Risperidone Treatment for Antidepressant-Resistant Symptoms of Chronic Military Service–Related PTSD A Randomized Trial  Krystal et al. | Psychiatry | 8/3/2011 JAMA | Several serotonin reuptake-inhibiting (SRI) antidepressants have received FDA approval for the treatment of posttraumatic stress disorder (PTSD), and are first-line treatments. Second-generation antipsychotics (SGAs) practice, such as risperidone, are also sometimes used. This trial compared risperidone (n=123) to placebo (n=124) in patients diagnosed with military –related PTSD whose symptoms persisted after 2 treatments with SRI drugs. The study found that the risperidone group did not have improvements in PTSD symptoms. Changes in Clinician-Administered PTSD Scale scores at 24 weeks were -16.3 in the risperidone group and -12.5 in the placebo group (mean difference, 3.74; 95% CI, -0.86 to 8.35; t=1.4; P=.11). This is a reversal of risperidone for treating military service-related PTSD. | 2015. “Some drugs have a small positive impact on PTSD symptoms and are acceptable. Fluoxetine, paroxetine and venlafaxine may be considered as potential treatments for the disorder. For most drugs there is inadequate evidence regarding  efficacy for PTSD, pointing to the need for more research in this area… Four drugs... showed superiority over placebo in single RCTs [randomized controlled studies], whereas eleven did not: alprazolam, citalopram, desipramine, escitalopram, imipramine, lamotrigine, nefazadone, risperidone, tiagabine and valproate semisodium.”^36^ | Google Scholar Search: “risperidone PTSD systematic review” |
| 321 | Effect of Increasing Doses of Saw Palmetto Extract on Lower Urinary Tract Symptoms A Randomized Trial  Barry et al. | Urology | 9/28/2011 JAMA | Benign prostatic hyperplasia (BPH), an enlargement of the prostate that often occurs as men age, often causes bothersome symptoms such as frequent urination. Saw palmetto, a supplement known to have anti-androgenic effects, has been reportedly used for this condition as far back as the 1800’s, and it is estimated that as many as 30-90% of patients seen by urologists use phytotherapeutic agents such as saw palmetto.^37^ A randomized controlled trial investigated the effects of saw palmetto extract (n=176) vs placebo (n=181) on American Urological Association Symptom Index scores in patients with lower urinary tract symptoms attribute to BPH. In patients with BPH, the use of saw palmetto was no better than placebo in reducing lower urinary tract symptoms (group mean difference, 0.79; P=.91). This is a reversal of saw palmetto extract for reducing symptoms of BPH. | 2012. Cochrane review. “Serenoa repens, at double and triple doses, did not improve urinary flow measures or prostate size in men with lower urinary tract symptoms consistent with BPH.”^38^ | Pubmed found systematic review |
| 322 | Vitamin E and the Risk of Prostate Cancer The Selenium and Vitamin E Cancer Prevention Trial (SELECT)  Klein et al. | Public health/ Preventive medicine | 10/12/2011 JAMA | Due to epidemiological and preclinical evidence that selenium and vitamin E were protective against prostate cancer, the SELECT trial was designed to determine the effect of the supplements on prostate cancer.^39^ Vitamin E was a popular supplement being used by men in the United States at the time.^40^ This follow up study of 6-10 years found that, compared with placebo (n=8696), supplementation with Vitamin E alone (n=8737) significantly increased risk of prostate cancer among healthy men (HR, 1.17; 99% CI, 1.004-1.36, P=.008). There were no significant differences in risks of prostate cancer between the placebo group and the selenium group or the Vitamin plus selenium group. This is a reversal of Vitamin E for reducing risk of prostate cancer. | 2013. US Preventive Services Task Force. “Trials of vitamin E supplementation showed mixed results and altogether had no overall effect on cancer, CVD, or all-cause mortality.”^41^ | Pubmed found systematic review |
| 323 | Enteral Omega-3 Fatty Acid, γ-Linolenic Acid, and Antioxidant Supplementation in Acute Lung Injury  Rice et al. | Pulmonary | 10/12/2011 JAMA | Acute lung injury is a condition where trauma upregulates inflammatory processes, which can disrupt the lung endothelial and epithelial barriers.^42^ Omega-3 fatty acids such as docosahexaenoic acid (DHA) and eicosapentaenoic acid (EPA) have anti-inflammatory effects and guidelines have suggested their use in patients with acute lung injury.^43^ Small trials have shown an association between omega-3 and antioxidant supplementation and improved oxygenation and respiratory physiology.^44^ This study found that, compared to control supplement (n=129), twice daily enteral supplementation of n-3 fatty acids, gamma-linolenic acid, and antioxidants (n=143) did not improve ventilator-free days in patients with acute lung injury. In fact, the study was stopped early due to futility and potential harm. The supplement group had 14.0 ventilator-free days vs 17.2 days in the control group (P=.02). This is a reversal of omega-3 fatty acid, γ-linolenic acid, and antioxidant supplementation for improving outcomes in acute lung injury patients. | 2015. “The pooled results did not show a significant reduction in the risk of all-cause mortality (M-H RR (the overall Mantel-Haenszel relative risk), 0.81 (95% CI, 0.50–1.31); p = 0.38; 6 trials, n = 717) in ALI/ARDS patients treated with the immunomodulatory diet. This treatment also did not extend the ventilator-free days and ICU-free days. However, patients with high mortality might benefit from this treatment. Conclusions: The enteral immunomodulatory diet could not reduce the severity of the patients with ALI/ARDS..”^45^ | Pubmed found systematic review |
| 324 | Platelet-Rich Plasma Injection for Chronic Achilles Tendinopathy A Randomized Controlled Trial  de Vos et al. | Orthopedic | 1/13/2010 JAMA | Achilles tendinopathy is a tendon disorder that affects many athletes and inactive middle-aged individuals. Platelet-rich plasma injections for tendinopathy were thought to provide growth factors to aid in tissue repair processes^46^ and the treatment has been used since the 1980s.^47^ This study determined that platelet-rich plasma injections (n=27) did not improve pain and activity among patients with chronic Achilles tendinopathy who were treated with eccentric exercises compared to a saline injection (n=27). Both groups showed improvement in scores in the Victorian Institute of Sports Assessment-Achilles questionnaire, but there was no significant difference between the two groups (adjusted between-group difference from baseline to 24 weeks, -0.9; 95% CI, -12.4 to 10.6). This is a reversal of platelet-rich plasma injections for chronic Achilles tendinopathy. | 2015. Cochrane review. “There is insufficient evidence from randomised controlled trials to draw conclusions on the use, or to support the routine use, of injection therapies for treating Achilles tendinopathy… Injection therapies include a range of options such as corticosteroids, high-volume saline, prolotherapy, autologous blood, platelet-rich plasma, aprotinin, botulinum toxin, sodium hyaluronate, polysulphated glycosaminoglycan and polidocanol.”^48^ | Pubmed found systematic review |
| 325 | Corticosteroid Treatment and Intensive Insulin Therapy for Septic Shock in Adults: A Randomized Controlled Trial  The COIITSS Study Investigators | Critical care | 1/27/2010 JAMA | Insulin therapy is used alongside corticosteroid therapy in the treatment of septic shock,^49^ to normalize blood glucose levels in ICU patients. Some guidelines suggest that continuous intensive insulin treatment to lower glucose levels adds to survival benefit.^50^ Clinical studies on intensive insulin therapy in patients with septic shock have shown mixed results.^51 52^ This study found that, compared to conventional insulin therapy (n=254), intensive insulin therapy (n=255) did not improve in-hospital mortality in patients who were treated with hydrocortisone for septic shock (45.9% intensive vs 42.9% conventional; RR, 1.07%; 95% CI, 0.88-1.30; P=.50). This is a reversal of intensive insulin therapy for septic shock. | 2014. “For patients with sepsis, IIT [intensive insulin therapy] and conservative glucose management show similar efficacy, but ITT is associated with a higher incidence of hypoglycemia.”^53^ | Pubmed found systematic review |
| 326 | Early vs Late Tracheotomy for Prevention of Pneumonia in Mechanically Ventilated Adult ICU Patients: A Randomized Controlled Trial  Terragni et al. | Critical care | 4/21/2010 JAMA | Physicians in the ICU are often required to decide which patients will require prolonged mechanical intubation and if a tracheotomy will be appropriate in those patients. Tracheotomies are an invasive, costly procedure but potentially prevent ventilator-associated pneumonia (VAP) and reduce the length of respiratory support and sedative use. Consensus guidelines recommend late tracheotomy (after 3 weeks of endotracheal intubation),^54^ and this practice has been widely accepted. In spite of this recommendation, an analysis by the US National Trauma Association reported significant variation in the timing of tracheotomy between ICUs.^55^ The analysis found that hospitals were performing early tracheotomy on patients despite the lack of confirmatory evidence of benefit from the procedure, leading to costly, prolonged hospital stays. The randomized control trial by Terragni et al., sought to determine the effectiveness of early tracheotomy (n=209) compared with a late tracheotomy (n=210) in reducing the incidence of VAP. The trial found that early tracheotomy performed after 6 to 8 days of endotracheal intubation did not result in a reduced incidence of VAP compared with late tracheotomy performed after 13 to 15 days of endotracheal intubation (14% early vs 21% late; P=.07; HR, 0.66; 95% CI, 0.442-1.04). This is a reversal of early tracheotomy for preventing pneumonia in mechanically ventilated ICU patients. | 2015. Cochrane review. “The whole findings of this systematic review are no more than suggestive of the superiority of early over late tracheostomy because no information of high quality is available for specific subgroups with particular characteristics.”^56^ | Pubmed found systematic review |
| 327 | Annual High-Dose Oral Vitamin D and Falls and Fractures in Older Women: A Randomized Controlled Trial  Sanders et al. | Public health/ Preventive medicine | 5/12/2010 JAMA | Research on Vitamin D supplementation on falls and fractures has been inconsistent. Meta-analyses have shown that 700-800 IUs of vitamin D reduce fractures by 13-26%,^57^ while others have found vitamin D supplementation to be ineffective. A Cochrane review reported that there was a non-significant increase in hip fractures associated with D supplementation. Vitamin D supplementation is recommended for adults over 50 years with a fracture.^58 59^ This double-blind, placebo controlled randomized trial found that women above the 70 years taking high-dose cholecalciferol (n=1131) were at an increased risk of falls and fractures compared to women taking placebo (n=1125). The rate of fractures was 15.2% in the supplement group vs 12% in the placebo group. The rate of falls was 83.4 per 100 person-years in the supplement group vs 72.7 per 100 person-years in the placebo group (incidence rate ratio, 1.15l 95% CI, 1.02-1.30; P=.03) The authors suggest that further research on the safety of high-dose vitamin D supplementation is needed. This is a reversal of high-dose oral Vitamin D in preventing falls and fractures in older women. | 2014. Cochrane review. “Vitamin D alone is unlikely to prevent fractures in the doses and formulations tested so far in older people. Supplements of vitamin D and calcium may prevent hip or any type of fracture. There was a small but significant increase in gastrointestinal symptoms and renal disease associated with vitamin D and calcium. This review found that there was no increased risk of death from taking calcium and vitamin D.”^60^ | Pubmed found systematic review |
| 328 | Effect of Glucosamine on Pain-Related Disability in Patients With Chronic Low Back Pain and Degenerative Lumbar Osteoarthritis: A Randomized Controlled Trial  Wilkens et al. | Orthopedic | 7/7/2010 JAMA | Nonspecific chronic low back pain (LBP) is one the most prevalent, expensive, and poorly treated conditions seen by primary care clinicians. Treatment approaches are empiric and based on scant evidence. Glucosamine is supplement that is commonly used to treat peripheral joint osteoarthritis (OA).^61^ A report found that more than 25% of patients with chronic LBP have tried glucosamine supplements, seeking to gain relief for their back pain.^62^ Wilkens et al. found that after 250 patients with chronic LBP and degenerative lumbar OA were randomized to treatment with daily glucosamine (n=125) or placebo (n=125), pain-related disability did not differ at 6 months (P=.72) or at 1-year follow-up (P=.97). This is a reversal of glucosamine for reducing pain-related disability in patients with chronic LBP and OA. | 2013. “On the basis of the current research, any clinical benefit of oral glucosamine for patients with chronic LBP and radiographic changes of spinal OA can neither be demonstrated nor excluded based on insufficient data and the low quality of existing studies.”^63^ | Pubmed found systematic review |
| 329 | Elective Intra-aortic Balloon Counterpulsation During High-Risk Percutaneous Coronary Intervention: A Randomized Controlled Trial  Perera et al. | Cardiovascular | 8/25/2010 | Patients with impaired left ventricular function who undergo percutaneous coronary intervention (PCI) are at high risk of cardiogenic shock or death. In these circumstances, patients can be treated by intra-aortic balloon pump (IABP), which augments coronary blood flow and decreases myocardial oxygen demand. IABP insertion is common practice,^64^ and observational studies have reported fewer intra-procedural complications and reduced major adverse cardiovascular events with prophylactic IABP insertion during high-risk PCI. International guidelines for PCI do not recommend prophylactic IABP, but they suggest that IABP should be used for patients with an extreme hemodynamic compromise.^65^ The BCIS-1 study aimed to determine whether routine IABP insertion before PCI would effectively reduce major adverse cardiac and cardiovascular events (MACCE) in patients with severe left ventricular dysfunction and extensive coronary disease after 28 days. The investigation found that, compared to no IABP insertion (n=150), elective IABP insertion (n=151) did not reduce the incidence of MACCE following PCI (15.2% elective IABP vs 16.0% no IABP; OR, 0.94; 95% CI, 0.51-1.76; P=.85). This is a reversal of IABP for patients with severe left ventricular dysfunction and extensive coronary disease. | 2016. “The present results do not favor the clinical utility of IABP in patients suffering high-risk PCI without CS and AMI [acute myocardial infarction] complicated with CS [cardiogenic shock].”^66^ | Pubmed found systematic review |
| 330 | Transfusion Requirements After Cardiac Surgery The TRACS Randomized Controlled Trial  Hajjar et al. | Cardiovascular | 10/13/2010  JAMA | Cardiac surgery often results in the need for blood transfusions, with rates ranging between 30-100%.^67^ Part of the variation in transfusion practices may be due to the variation in the thresholds in which physicians transfuse. Some have advocated for a more liberal threshold, when hemoglobin concentrations reach 10 g/dL, whereas others have a more restrictive strategy, waiting until hemoglobin concentrations fall as low as 6 g/dL.^68^ In the TRACS trial, patients who underwent cardiac surgery with cardiopulmonary bypass were randomized to a liberal strategy of blood transfusion (n=253) or a restrictive strategy (n=249). A more restrictive strategy resulted in similar rates of a composite of 30-day mortality and severe morbidity as compared to the more liberal strategy (11% restrictive vs 10% liberal; between group difference, 1%; 95% CI, -6% to 4%; P=.85). This is a reversal of liberal blood transfusions over restrictive after cardiac surgery. | 2016. Cochrane. “Transfusing at a restrictive haemoglobin concentration of between 7 g/dL to 8 g/dL decreased the proportion of participants exposed to RBC transfusion by 43% across a broad range of clinical specialties. There was no evidence that a restrictive transfusion strategy impacts 30-day mortality or morbidity (i.e. mortality at other points, cardiac events, myocardial infarction, stroke, pneumonia, thromboembolism, infection) compared with a liberal transfusion strategy.”^69^ | Pubmed |
| 331 | Effect of DHA Supplementation During Pregnancy on Maternal Depression and Neurodevelopment of Young Children: A Randomized Controlled Trial  Makrides et al. | Obstetrics/Gynecology | 10/20/2010 JAMA | Several international guidelines recommend that pregnant women increase their dietary docosahexaenoic acid (DHA) intakes.^70 71^ These recommendations are founded on epidemiological studies from the US and Europe which have associated a higher intake of n-3 long-chain polyunsaturated fatty acids (LCPUFA) from fish and seafood consumption during pregnancy with a reduced risk of postpartum depression and enhanced neurodevelopment for newborns.^72^ In 2007, Martek Biosciences Corporation, a manufacturer of DHA products, reported revenues of approximately $307 million dollars, and was awarded contracts to supply DHA to Mead Johnson and Abbott to use in infant formulas for cognitive development.^73^ The DOMInO trial investigated the effect of DHA supplements (n of women=1197; n of infants= 351) on postpartum depression in new mothers and cognitive function in newborns compared to placebo (n of women=1202; n of infants=375). The study found that high levels of depressive symptoms in mothers during the first 6 months postpartum did not differ between the DHA group and the placebo group (9.67% vs 11.19%; adjusted RR, 0.85; 95% CI, 0.70-1.02; P = .09). Mean cognitive composite scores (adjusted mean difference, 0.01; 95% CI, −1.36 to 1.37; P = .99) and language composite scores (adjusted mean difference, −1.42; 95% CI, −3.07 to 0.22; P = .09) did not differ between children of the DHA and placebo groups as well. This is a reversal of DHA for pregnant women for preventing maternal depression and improving neurodevelopment of young children. | 2013. Cochrane review. “There is insufficient evidence to conclude that selenium, DHA or EPA prevent postnatal depression. There is currently no evidence to recommend any other dietary supplement for prevention of postnatal depression.”^74^  For neurodevelopment of young children: 2017. “The following nine cognition outcomes: attention, behaviour, crystallised intelligence, fluid intelligence, global cognition, memory, motor skills, visual processing, and problem solving were not significantly impacted by nutritional interventions, although 65% of studies conducted post-hoc data analyses and were likely to be underpowered. Although, long chain polyunsaturated fatty acids (LCPUFA) supplementation was associated with a marginal increase in crystallised intelligence (Effect size (ES): 0.25; 95% confidence interval (95% CI): -0.04, 0.53), the effect was not statistically significant (p = 0.09), with significant study heterogeneity (p = 0.00).”^75^ | Both sources were pubmed-found systematic reviews |
| 332 | Docosahexaenoic Acid Supplementation and Cognitive Decline in Alzheimer Disease: A Randomized Trial  Quinn et al. | Neurology | 11/3/2010 JAMA | Docosahexaenoic acid (DHA), a popular supplement, has been suggested by epidemiological studies to be associated with a reduced incidence of Alzheimer disease.^76^ In 2007, Martek Biosciences Corporation, a manufacturer of DHA products, reported revenues of approximately $307 million dollars, with some of their products marketed towards cognitive and Alzheimer’s prevention.^73^ Nutritionists suggested that the consumption of DHA might prevent AD.^77^ Quinn et al., conducted a multi-center, placebo-controlled randomized trial examining if supplementation of DHA to patients with mild to moderate AD slowed the cognitive and functional decline. The study found that after 18 months of treatment, DHA (n=238) had no beneficial effect on the rate of cognitive decline compared to placebo (n=164). The rate of change in the cognitive subscale of the Alzheimer’s Disease Assessment Scale was an increased mean of 7.98 points (95% CI, 6.51-9.45) in the DHA group and 8.27 points (95% CI, 6.72-9.82) in the placebo group (linear mixed-effects mode: P=.41). This is a reversal of PHA for slowing down cognitive and functional decline in patients with AD. | 2016. Cochrane review. “We found no convincing evidence for the efficacy of omega-3 PUFA supplements in the treatment of mild to moderate AD [Alzheimer’s disease]. This result was consistent for all outcomes relevant for people with dementia. Adverse effects of omega-3 PUFAs seemed to be low, but based on the evidence synthesized in this review, we cannot make a final statement on tolerability. The effects on other populations remain unclear.”^78^ | Pubmed found systematic review |
| 333 | Efficacy and Safety of Prescription Omega-3 Fatty Acids for the Prevention of Recurrent Symptomatic Atrial Fibrillation: A Randomized Controlled Trial  Kowey et al. | Cardiovascular | 12/1/2010 JAMA | Atrial fibrillation (AF) is prevalent medical condition and patients would benefit from safe and efficient treatment alternatives when standard therapies fail. Multiple trials have suggested that omega-3 polyunsaturated fatty acids may be an effective treatment for AF.^79 80^ However, evidence supporting the safety and efficacy of omega-3 supplementation has been lacking. As a result of confounding evidence, doctors have different perceptions of what the net clinical benefit may be of omega-3 supplementation consumption to their patients.^81^ Kowey and colleagues evaluated the safety and efficacy of prescribing omega-3 fatty acids for the prevention of recurrent symptomatic AF. After 24 weeks, there was no difference between treatment group (n=258) and placebo group (n=269) for recurrence of symptomatic AF in patients with paroxysmal AF (HR, 1.15; 95% CI, 0.90-1.46; P=.26). This is a reversal of Omego-3 fatty acids for preventing recurrent symptomatic AF. | 2013.. “Published clinical trials do not support n-3 PUFAs as agents aimed at preventing either postoperative or recurrent AF.”^31^ | Pubmed found systematic review |
| 334 | Weight Lifting for Women at Risk for Breast Cancer–Related Lymphedema: A Randomized Trial  Schmitz et al. | Oncology | 12/22/2010 + 12/29/2010 JAMA | Lymphedema is a common complication among breast cancer survivors and can cause swelling, discomfort, impaired arm function, and a lower quality of life. Guidelines recommend avoiding weight lifting among breast cancer survivors to reduce risk of lymphedema.^82 83^ This study found that, compared to no exercise (n=75), a program of slowly progressive weight lifting (n=72) did not increase incidence of lymphedema in breast cancer survivors (intervention vs control, 11% vs 17%; cumulative incidence difference, -6.0%; 95% CI, -17.2% to 5.2%; P for equivalence =.04). Furthermore, weight lifting may have a preventive effect. This is a reversal of avoiding weight lifting for women at risk of breast cancer-related lymphedema. | 2015. Cochrane review. “The evidence suggests that progressive resistance exercise therapy does not increase the risk of developing lymphoedema, provided that symptoms are closely monitored and adequately treated if they occur. Given the degree of heterogeneity encountered, limited precision, and the risk of bias across the included studies, the results of this review should be interpreted with caution.”^84^ | Pubmed found systematic review |
| 335 | Effect of Selenium and Vitamin E on Risk of Prostate Cancer and Other Cancers The Selenium and Vitamin E Prevention Trial (SELECT)  Lippman et al. | Public health/ Preventive medicine | 1/07/2009 JAMA | Through secondary analyses, randomized controlled trials, and epidemiological studies,^85^ it was found that selenium and vitamin E supplementation may be protective against certain cancers, including prostate cancer. Vitamin E was a common supplement taken at the time of this study.^40^ This randomized control study sought to determine if a diet supplementation with Vitamin E and selenium would reduce prostate cancer risk in men. The authors found that selenium (n=8752) or vitamin E (n=8737), alone or in combination (n=8703), did not reduce rates of prostate cancer compared to placebo. The Vitamin E group had a HR of 1.13 (99% CI, 0.95-1.35), selenium had a HR of 1.04 (99% CI, 0.87-1.24), and the combination had a HR of 1.05 (99% CI, 0.88-1.25) compared to placebo. In fact, there were statistically nonsignificant increased risks of prostate cancer (P=.06) in the Vitamin E group and type 2 diabetes mellitus (P=.16) in the Selenium group. This is a reversal of selenium and Vitamin E for reducing risk of prostate in men. | 2014. Cochrane review. “RCTs [randomized controlled trials] assessing the effects of selenium supplementation on cancer risk have yielded inconsistent results, although the most recent studies, characterised by a low risk of bias, found no beneficial effect on cancer risk, more specifically on risk of prostate cancer, as well as little evidence of any influence of baseline selenium status. Rather, some trials suggest harmful effects of selenium exposure.”^86^  2013. US Preventive Services Task Force. “Trials of vitamin E supplementation showed mixed results and altogether had no overall effect on cancer, CVD, or all-cause mortality.”^41^ | Pubmed found systematic review |
| 336 | Vitamins E and C in the Prevention of Prostate and Total Cancer in Men The Physicians' Health Study II Randomized Controlled Trial  Gaziano et al. | Public health/ Preventive medicine | 1/07/2009 JAMA | Many people take antioxidant vitamins in the hopes of strengthening their immune system because they are affordable and easily available.^87^ Vitamin E and C have also been associated with reduced risk of some types of cancer.^88^ However, trials have reported contradicting information. The HOPE-TOO trial showed no reduction in prostate cancer when given Vitamin E,^89^ yet the ATBC trial reported a reduction in prostate cancer. In a continuation of the HOPE and HOPE-TOO studies, the present study set out to evaluate whether long-term supplementation of Vitamin E and Vitamin C decreases the risk of prostate and total cancer in male physicians, ages 50 years and older. Compared with placebo (n=7000), Vitamin E (n=6983) showed no effect on incidence of prostate cancer (active vs placebo Vitamin E, 9.1 vs 9.5 events per 1000 person-years; HR, 0.97; 95% CI, 0.85-1.09; P=.58). The active Vitamin E group (n= 7315) and the control Vitamin E group (n=7326) also had similar incidence of total cancer (17.8 vs 17.3 cases/1000 person-years; HR, 1.04; 95% CI, 0.95-1.13; P=.41). Similarly, there was no significant difference between Vitamin C (n=7006) and placebo (n=6977) on incidence of prostate cancer (9.4 vs 9.2 cases/1000 person-years; HR, 1.02; 95% CI, 0.90-1.15; P=.80) and no difference in active Vitamin C (n=7329) and placebo (n=7312) in total cancer (17.6 vs 17.5 cases/1000 person-years; HR, 1.01; 95% CI, 0.92-1.10; P=.86). This is a reversal on Vitamin E and C supplementation for preventing prostate and total cancer in men. | 2013. US Preventive Services Task Force.  “Trials of vitamin E supplementation showed mixed results and altogether had no overall effect on cancer, CVD, or all-cause mortality…. The few studies addressing folic acid, vitamin C, and vitamin A showed no effect on CVD, cancer, and mortality.”^41^ | Pubmed found systematic review |
| 337 | Cardiac Outcomes After Screening for Asymptomatic Coronary Artery Disease in Patients With Type 2 Diabetes The DIAD Study: A Randomized Controlled Trial  Young et al. | Endocrinology, Diabetes, and Metabolism | 4/15/2009 JAMA | Patients with type 2 diabetes are at a high risk of coronary artery disease (CAD).^90^ In 1998, an expert panel of the American Diabetes Association recommended that patients with type 2 diabetes who have 2 or more risk factors for CAD be screened for asymptomatic CAD.^91-93^ The DIAD study compared patients with type 2 diabetes and no symptoms of CAD to screening (n=561) or no screening (n=562) to compare rates of cardiac death of nonfatal myocardial infarction. The rate of cardiac death or nonfatal myocardial MI in the DIAD study did not differ between the screening and no screening groups (2.7% vs 3.0%; HR, 0.88; 95% CI, 0.44-1.88; P= .73). This is a reversal of screening for asymptomatic CAD in patients with type 2 diabetes. | 2016. “The present analysis shows no evidence for a benefit of screening diabetic patients for the presence of asymptomatic CAD. The proportion of patients who undergo myocardial revascularization as a consequence of screening was low.”^94^ | Pubmed found systematic review |
| 338 | Laparoscopic Uterosacral Nerve Ablation for Alleviating Chronic Pelvic Pain A Randomized Controlled Trial  Daniels et al. | Obstetrics/Gynecology | 9/02/2009 JAMA | Laparoscopy uterosacral nerve ablation (LUNA) is the treatment of chronic pelvic pain and it is increasingly being used.^95 96^ The LUNA trial randomized patients suffering from chronic pelvic pain to laparoscopy without pelvic denervation (n=192) or LUNA (n=187). There was no significant difference between LUNA and no LUNA in worst pain scores assesses by visual analogue scale (mean difference, -0.04cm; 95% CI, -.33 to 0.35 cm; P=.80), dysmenorrhea (−0.09 cm; 95% CI, −0.49 to 0.30 cm; P = .60), or dyspareunia (0.18 cm; 95% CI, −0.22 to 0.62 cm; P = .40). This is a reversal of LUNA for alleviating chronic pelvic pain. | 2011. “Surgical interruption of pelvic nerve pathways is not beneficial in treating dysmenorrhoea, and may be associated with adverse effects including constipation.”^97^ | Google scholar search “dysmenorrhoea surgery nerve systematic review” |
| 339 | Effect of High Perioperative Oxygen Fraction on Surgical Site Infection and Pulmonary Complications After Abdominal Surgery The PROXI Randomized Clinical Trial  Meyhoff et al. | Surgery | 10/14/2009 JAMA | The 2007 Global Guidelines for the Prevention of Surgical Site Infection recommends that adult patients undergoing general anesthesia for surgical procedures should receive an 80% fraction of inspired oxygen, (FIO2), intraoperatively and in the immediate postoperative period for 2-6 hours to reduce the risk of surgical site infections (SSI) , if practical.^98^ A meta-analysis found that perioperative administration of high inspired oxygen (80% concentration) was associated with a 3% absolute reduction and a 25% relative reduction in risk of SSI.^99^ On the contrary, a trial by Pryor et al. was stopped prematurely for futility, after high inspiratory oxygen fraction (FIO_2_) during the perioperative period did not reduce the overall incidence of SSI; Instead, the participants experienced several deleterious effects, because the frequency of wound infection was more than doubled in patients randomized to the high FIO_2_.^100^ The PROXI trial was designed to assess the benefits and harms of administering a high FIO_2_ (n=685) compared to low FIO_2_ (n=701) in a general surgical population of patients undergoing laparotomy. The study found that there was no difference in SSI risk reduction between high and low oxygen groups (19.1% vs 20.1%; OR, 0.94; 95% CI, 0.72-1.22; P=.64). High FIO_2_ was not associated with a significant increase in the frequency of pulmonary complications or other adverse events. Mortality differences were not statistically significant (P=.13). This is a reversal on administering high preoperative oxygen fraction for preventing surgical site infections for patients undergoing laparotomy. | 2015. Cochrane review. “As the risk of adverse events, including mortality, may be increased by a fraction of inspired oxygen of 60% or higher, and as robust evidence is lacking for a beneficial effect of a fraction of inspired oxygen of 60% or higher on surgical site infection, our overall results suggest that evidence is insufficient to support the routine use of a high fraction of inspired oxygen during anaesthesia and surgery. Given the risk of attrition and outcome reporting bias, as well as other weaknesses in the available evidence, further randomized clinical trials with low risk of bias in all bias domains, including a large sample size and long-term follow-up, are warranted.”^101^ | Pubmed found systematic review |
| 340 | Surgical Mask vs N95 Respirator for Preventing Influenza Among Health Care Workers: A Randomized trial  Loeb et al. | Public health/ Preventive medicine | 11/04/2009 | During the H1N1 pandemic in 2009-2010, healthcare workers (HCWs) were among the most affected, with higher rates of influenza-related hospitalizations seen compared to workers in other industries.^102^ The Centers for Disease Control and Prevention (CDC) recommends that HCWs wear respiratory protective devices (RPDs) such as N95 filtering face piece respirators (FFRs) if they must share the same airspace with a potentially infectious patient.^103^ Additionally, the National Institute for Occupational Safety and Health, in their 2009 guideline, recommends that HCWs use N95s instead of surgical masks because HCWs using surgical masks will not be protected against exposure to airborne transmissible diseases.^104^ This randomized control trial randomized nurses to either fit-tested N95 respirators (n=210) or surgical masks (n=212) to use when providing care to patients with a respiratory illness during the 2008-2009 influenza season. The study found no significant difference in rates of laboratory-confirmed influenza among the group of nurses who wore a surgical mask as compared to the group wearing N95 respirators (23.6% vs 22.9%, absolute risk difference, -0.73%; 95% CI, -8.8% to 7.3%; P=.86). This is a reversal of N95 respirators for preventing influenza among health care workers. | 2016. “Although N95 respirators appeared to have a protective advantage over surgical masks in laboratory settings, our meta-analysis showed that there were insufficient data to determine definitively whether N95 respirators are superior to surgical masks in protecting health care workers against transmissible acute respiratory infections in clinical settings.”^105^ | Pubmed found systematic review |
| 341 | Prone Positioning in Patients With Moderate and Severe Acute Respiratory Distress Syndrome: A Randomized Controlled Trial  Taccone et al. | Critical care | 11/11/2009 JAMA | In 2001, the Intensive and Critical Care Nursing Journal released guidelines recommending that patients with acute respiratory distress syndrome (ARDS) be placed in the prone position to improve survival. Additionally, the Surviving Sepsis Campaign Guidelines published in 2008,^106^ recommended prone positioning for patients in whom mechanical ventilation may cause harm. The Prone-Supine II Study (PSII) randomized patients with ARDS to either prone (n=168) or supine (n=174) positioning and assessed death from any cause after 28 days of enrollment. PSII found that prone positioning and supine positioning had similar 28-day survival rates (31.0% vs 32.8%; RR, 0.97; 95% CI, 0.84-1.13; P=.72). This is a reversal of prone positioning for patients with moderate and severe ARDS. | 2015. Cochrane review. “We found no convincing evidence of benefit nor harm from universal application of PP [prone positioning] in adults with hypoxaemia mechanically ventilated in intensive care units (ICUs).”^107^ This Cochrane analysis included the Guerin et al paper, ‘Prone Positioning in Severe Acute Respiratory Distress Syndrome’, which was found to be dissimilar to the 8 other studies reviewed, increasing heterogeneity of the meta-analysis. | Pubmed found systematic review |
| 342 | Intravenous Drug Administration During Out-of-Hospital Cardiac Arrest: A Randomized Trial  Olasveengen et al. | Cardiovascular | 11/25/2009 JAMA | American Heart Association guidelines recommend that out-of-hospital emergency personnel treat patients by providing intravenous access (IV) and drug administration when they are experiencing cardiac arrest.^108^ The Advanced Cardiac Life Support (ACLS) guidelines specify that epinephrine should be administered every 3 to 5 minutes to patients that are not responding to compressions. However, whether the administration of out-of-hospital epinephrine provides patients with improved survival to hospital discharge is unknown. According to Olasveengen et al., the use of epinephrine came into favor based on preclinical evidence of increased cerebral and coronary perfusion by redirected peripheral blood flow and animal studies of the short-term effects of epinephrine. After an extensive retrospective registry study found that epinephrine was an independent predictor of poor outcome^109^ and was associated with increased myocardial dysfunction and disturbed cerebral microcirculation after cardiac arrest,^110^ a need for testing the use of epinephrine became paramount. This randomized controlled trial compared IV epinephrine treatment (n=418) to standard ACLS (n=433) in patients with out-of-hospital nontraumatic cardiac arrest and found that the group receiving IV epinephrine had lower rates of short-term survival. Between the IV and no IV groups, there was no statistically significant difference in survival to hospital discharge (10.5% vs 9.2%; P= .61) or survival at 1 year (10% vs 8%; P=.53). This is a reversal of IV epinephrine administration during out-of-hospital cardiac arrest. | 2014. Effects of prehospital adrenaline administration on out-of-hospital cardiac arrest outcomes: a systematic review and meta-analysis. “Prehospital adrenaline administration may increase prehospital return of spontaneous circulation, but it does not improve overall rates of return of spontaneous circulation, hospital admission and survival to discharge.”^111^ | Pubmed search of article title |
| 343 | Effectiveness of Public Report Cards for Improving the Quality of Cardiac Care The EFFECT Study: A Randomized Trial  Tu et al. | Cardiovascular | 12/02/2009 JAMA | Public report cards for physicians and hospitals are a contentious issue in the medical community.^112^ Supporters of report cards believe that being more transparent with the public by providing performance data on hospitals/clinicians helps hospitals and providers to engage in quality improvement activities and increase the accountability and transparency.^113^ Critics argue that report cards are often misleading and these initiatives may have unintended negative consequences for the public, diverting resources away from other important needs and/or incentivizing hospitals and staff to follow “target rates” protocols that may be inappropriate.^114^ Despite the debate, public release of hospital performance data is increasingly being mandated by policymakers. The EFFECT study evaluated the effect of public report cards on significant hospital improvement for the treatment of acute myocardial infarction (AMI) or congestive heart failure (CHF). EFFECT found that hospitals who received early feedback (n of hospitals=42; n of patients= 8481) from report cards as did not differ in a system-wide improvement in the care of patients with AMI and CHF compared to hospitals with delayed feedback (n of hospitals=39; n of patients= 7516). Both groups had similar results in the composite AMI process-of-care indicator (absolute change, 1.5%; 95% CI, -2.2% to 5.1%; P=.43) and the composite CHF process-of-care indicator (absolute change, 0.6%; 95% CI, -4.5% to 4.9%; P=.81). This is a reversal of public report cards for improving quality of cardiac care in hospitals. | 2011. Cochrane review. “The small body of evidence available provides no consistent evidence that the public release of performance data changes consumer behaviour or improves care. Evidence that the public release of performance data may have an impact on the behaviour of healthcare professionals or organisations is lacking.”^115^ | Pubmed found systematic review |
| 344 | Ginkgo biloba for Preventing Cognitive Decline in Older Adults: A Randomized Trial  Snitz et al. | Public health/ Preventive medicine | 12/23/2009+ 12/30/2009 JAMA | There are currently no medications approved for the prevention of dementia, yet *Ginkgo biloba* is used globally for the preservation of memory. Worldwide sales of *G. biloba* exceed $249 million annually.^116^ Despite a lack of evidence of its efficacy from clinical trials, *G. biloba* is a common natural product for cognitive health. The 2008 National Health Statistics Report found that that *G. biloba* is among the most popular non-vitamin, non-mineral natural products consumed by adults in the U.S.^117^ The GEM study investigated the effects of twice- daily consumption of *G. biloba* on rates of cognitive decline in older adults and found that, compared to placebo (n=1524), *G. biloba* supplementation (n=1545) did not result in less cognitive decline in older adults. Rates of change over time did not differ between groups in Modified Mini-Mental State Examination scores (-0.15 in placebo group vs -0.14 in *G. biloba* group; P=.71) or the cognitive subscale of the Alzheimer Disease Assessment Scale (P=.97). This is a reversal of *Gingko biloba* for preventing cognitive decline in older adults. | 2017. “Low-strength evidence suggests omega-3 fatty acids and ginkgo biloba did not improve clinical Alzheimer’s-type dementia (CATD)* incidence or cognitive performance in adults with normal cognition.”^118^ | Pubmed found systematic review |
| 345 | Effect of Testosterone Supplementation on Functional Mobility, Cognition, and Other Parameters in Older Men A Randomized Controlled Trial  Emmelot-Vonk et al. | Endocrinology, Diabetes, and Metabolism | 1/2/2008 JAMA | Decline in serum levels of testosterone is associated with decreased muscle mass and muscle strength, cognitive decline, decrease in bone mass, and increase in abdominal fat.^119^ Clinical trials have suggested that testosterone supplementation could be beneficial to men, although most trials conclude that males should be carefully monitored while receiving these supplements.^120^ In 2002 alone, there were about 2 million prescriptions for testosterone in the US.^119^ Emmelot-Vonk and colleagues examined whether testosterone supplementation (80 mg) in males, 60-80 years of age with a low testosterone level, would improve cognitive function. The study found that testosterone supplementation (n=113), as compared with placebo (n=110), failed to show an improvement in cognitive function (mean difference in change, 0; 95% CI, -0.07-0.07; P=.86), functional mobility (mean difference in change, 0.01; 95% CI, -0.02-0.04; P=.61), bone mineral density (mean difference in change for total hip, 0; P=.77; for lumbar spine, 0; P=.47), and body mass index (mean difference in change, 0.; 95% CI, -0.2-0.3; P=.76). This is a reversal of testosterone supplementation on improving functional mobility, cognition, and other parameters in older men. | 2016. “The prescription of testosterone supplementation for low-T for cardiovascular health, sexual function, physical function, mood, or cognitive function is without support from randomized clinical trials.”^121^ |  |
| 346 | Omega-3 Free Fatty Acids for the Maintenance of Remission in Crohn Disease: The EPIC Randomized Controlled Trials  Feagan et al. | Gatroenterolgy/Hepatology | 4/09/2008 JAMA | Crohn’s Disease is a type of inflammatory bowel disease (IBD) in which no effective cure has been found. Patients diagnosed with Crohn’s Disease often turn to complementary and alternative medicine, including the use of fish oil. Approximately 10% of patients with IBD report the use of salmon oil.^122 123^ In the EPIC-I and EPIC-II randomized controlled trials, omega-3 fatty acids were no better than placebo in preventing relapse in patients with Crohn’s Disease. EPIC-I found that the omega-3 group (n=183) has a 31.6% relapse rate at 1 year, and the placebo group had a 35.7% rate (HR, 0.82; 95% CI, 0.51-1.19; P=.30). EPIC-II found a 47.8% and 48.8% relapse rate in the omega-3 and placebo groups (HR, 0.90; 95% CI, 0.67-1.21; P=.48). The authors conclude by stating, “we do not endorse this practice since patients with Crohn’s Disease who are at risk for relapse would be better served by taking medications of known efficacy.” This is a reversal of omega=3 fatty acids for the maintenance of remission in Crohn’s Disease. | 2014. Cochrane. Omega 3 fatty acids (fish oil) for maintenance of remission in Crohn's disease: “Evidence from two large high quality studies suggests that omega 3 fatty acids are probably ineffective for maintenance of remission in CD [Crohn’s disease]. Omega 3 fatty acids appear to be safe although they may cause diarrhea and upper gastrointestinal tract symptoms.”^124^ | Pubmed found systematic review |
| 347 | Femoral vs Jugular Venous Catheterization and Risk of Nosocomial Events in Adults Requiring Acute Renal Replacement Therapy: A Randomized Controlled Trial  Parienti et al. | Nephrology | 5/28/2008 JAMA | The US Centers for Disease Control and Prevention recommends that for patients requiring hemodialysis, venous catheterization placement should be in a jugular or femoral vein rather than a subclavian vein to avoid venous stenosis, if catheter access is needed.^125^ This recommendation differs from treatment of critically ill patients, in which the recommendation is to attempt catheterization of the subclavian and jugular sites first, since both of these sites have a lower risk of nosocomial infection over the femoral site.^126^ According to Parienti et al., for patients requiring hemodialysis, the choice between jugular or femoral sites remains a contentious issue, since observational studies report discordant findings on which site yields a higher incidence of complications. Recognizing this, the National Foundation of Kidney Disease Outcome and Quality guideline recommends that dialysis catheters do not exceed 3 weeks for the jugular and 5 days for femoral accesses,^127^ in hopes of decreasing the risk of catheter-related bloodstream infection. The Cathedia Study randomized adults requiring a first catheter insertion for renal replacement therapy to receive either jugular (n=313) or femoral (n=324) vein catheterization and investigated the rates of infection complications, catheter colonization, and removal. The study found that, when comparing the jugular to a femoral site catheterization, the risk of nosocomial complications was not reduced when using either site. The rate of catheter-related bloodstream infection was 2.3 vs 1.5 per 1000 catheter-days in the jugular and femoral groups, respectively (P = .42) This finding is inconsistent with the “widely accepted convention to avoid femoral catheterization.”^125^ This is a reversal of avoiding femoral catheterization for adults requiring renal replacement therapy. | 2012. Cochrane. review “In short-term haemodialysis catheterization, femoral and internal jugular CVA routes have similar risks for catheter-related complications, except internal jugular CVA routes are associated with higher risks of mechanical complications.”^128^ | Pubmed found systematic review |
| 348 | Hypericum perforatum (St John's Wort) for Attention-Deficit/Hyperactivity Disorder in Children and Adolescents A Randomized Controlled Trial  Weber et al. | Public health/ Preventive medicine | 6/11/2008 JAMA | Up to 30% of children who receive pharmaceuticals to treat attention-deficit/hyperactivity disorder (ADHD) experience adverse effects and treatment failure.^46^ As a result, many patients seek complementary and alternative medicine to treat the condition. A US survey examining the use of herbal medicine to treat ADHD found that almost 83% of caregivers gave herbal medicines alone- ginkgo biloba, Echinacea, and Hypericum perforatum (St. John's wort) being the most prevalent.46 Weber et al. examined the efficacy and safety of H. perforatum for the treatment of ADHD in children and adolescents, age 6-17 years. H. perforatum is believed to act similarly to a norepinephrine reuptake inhibitor.47 Children and adolscents diagnosed with ADHD were randomized to receive H. perforatum product (n=27) or placebo (n=27). After 8 weeks of treatment, there were no improvements in either the group of children receiving placebo or the group receiving H. perforatum, as measured by the ADHD Rating Scale-IV, and the Clinical Global Impression-Improvement Scale (P = .59). This is a reversal of H. perforatum for ADHD in children and adolescents. | 2011. “Current data suggest that Ginkgo biloba (ginkgo), and Hypercium perforatum (St. John's wort) are ineffective in treating ADHD.”^129^ | Pubmed found systematic review |
| 349 | Mortality and Cardiovascular Events in Patients Treated With Homocysteine-Lowering B Vitamins After Coronary Angiography: A Randomized Controlled Trial  Ebbing et al. | Cardiovascular | 8/20/2008 JAMA | Elevated plasma homocysteine levels are associated with a risk of developing coronary artery disease (CAD). Total plasma levels of homocysteine are a strong predictor of mortality in patients with CAD.^130^ Oral administration of folic acid has been shown to lower homocysteine levels, and observational studies have found that vitamin B6 intake is associated with an observed reduction in CAD, morbidity, and mortality.^131^ The WENBIT trial enrolled patients undergoing coronary angiography diagnosed with coronary artery disease or aortic valve stenosis to receive; (1) folic acid/vitamin B12/ vitamin B6 (n=771), (2) folic acid/vitamin B12 (n=769), (3) vitamin B6 alone (n=771), or (4) placebo (n=779). WENBIT found that lowering homocysteine levels with B vitamins did not reduce a composite of all-cause mortality or major cardiovascular events. Composite events occurred in 219 participants (14.2%) receiving folic acid/vitamin B12 vs 203 (13.1%) not receiving such treatment (HR, 1.09; 95%  CI, 0.90-1.32; P = .36) and 200 participants (13.0%) receiving vitamin B6 vs 222 (14.3%) not receiving vitamin B6 (HR, 0.90; 95% CI, 0.74-1.09; P = .28). Furthermore, the group of patients receiving folic acid/vitamin B12 saw a nonsignificant increase in the rates of stroke and CVD. This is a reversal on B vitamin supplementation for preventing major cardiovascular events and all-cause mortality. | 2017. Cochrane review. “In this third update of the Cochrane review, there were no differences in effects of homocysteine-lowering interventions in the form of supplements of vitamins B6, B9 or B12 given alone or in combination comparing with placebo on myocardial infarction, death from any cause or adverse events.”^132^ | Pubmed found systematic review |
| 350 | Serial 2-Point Ultrasonography Plus D-Dimer vs Whole-Leg Color-Coded Doppler Ultrasonography for Diagnosing Suspected Symptomatic Deep Vein Thrombosis A Randomized Controlled Trial  Bernardi et al. | Cardiovascular | 10/08/2008 JAMA | Lower extremities can be examined for suspected deep vein thrombosis (DVT) with ultrasonography of either the proximal veins (2-point ultrasonography) or the entire deep vein system (whole-leg ultrasonography).^133^ Whole-leg ultrasonography is thought to be better than 2-point ultrasonography because this approach can detect isolated calf DVT. Bernardi et al. studied whether the 2-point (n=801) and whole-leg ultrasonography (n=763) were equivalent and found that there was no difference in the rates of incidence of symptomatic venous thromboembolism in patients with initially normal diagnostic workup. The incidence of events was 7/801 (0.9%) in the 2-point group vs 9/763 (1.2%) in the whole-leg group (observed difference, 0.3%; 95% CI, -1.4% to 0.8%). This is a reversal of the use of whole-leg ultrasonography over 2-point ultrasonography for diagnosing suspected symptomatic deep vein thrombosis. | None found |  |
| 351 | Effect of Combined Folic Acid, Vitamin B6, and Vitamin B12 on Cancer Risk in Women: A Randomized Trial  Zhang et al. | Public health/ Preventive medicine | 11/05/2008 JAMA | Approximately one-third of US adults took multivitamin supplements containing folic acid, vitamin B6, and vitamin B12 from 1999-2000.^134^ Observational studies, which were conducted mostly before folic acid fortification, found an inverse association between high intake or blood level of folate, vitamin B6, and vitamin B12 and risk of cancer, particularly colorectal neoplasia and breast cancer, and primarily among those individuals consuming alcohol, a known antagonist for these B vitamins.^135 136^ This study found that a combination of folic acid, vitamin B6, and vitamin B12 had no effect on overall risk of total invasive cancer or breast cancer among women during the folic acid fortification era. Compared to the placebo group (n=2721) at 104.3/10000 person-years, the supplement group (n=2721) has a risk of 101.1/10000 person-years of developing invasive cancer (HR, 0.97; 95% CI, 0.79-1.18; P=.75). Risk of breast cancer development was 37.8/10000 person-years in the active group vs 45.6/10000 person-years in the placebo group (HR, 0.83; 95% CI, 0.60-1.14; P=.24). This is a reversal of combined folic acid and Vitamin B6 and B12 on reducing cancer risk in women. | 2013. USPSTF. “Limited evidence supports any benefit from vitamin and mineral supplementation for the prevention of cancer or CVD…The sex-specific subgroup analysis [on overall cancer incidence] showed a protective effect among men (adjusted relative risk, 0.69 [CI, 0.53 to 0.91]) but not women.”^137^ | Pubmed found systematic review |
| 352 | Low-Dose Aspirin for Primary Prevention of Atherosclerotic Events in Patients With Type 2 Diabetes: A Randomized Controlled Trial  Ogawa et al. | Endocrinology, Diabetes, and Metabolism | 11/12/2008 JAMA | Patients with diabetes are at an increased cardiovascular risk. The American Diabetes Association recommends use of aspirin as a primary prevention strategy to reduce cardiovascular events in patients with diabetes (type 1 or 2) at increased risk of CV disease.^138^ The Japanese Primary Prevention of Atherosclerosis with Aspirin for Diabetes trial randomized patients with type 2 diabetes to low-aspirin (n=1262) or placebo (n=1277). The trial found that the rate of atherosclerotic events, including fatal or nonfatal ischemic heart disease, fatal or nonfatal stroke, and peripheral arterial disease did not differ between the two groups (HR, 0.80; 95% CI, 0.58-1.10; log-rank test, P=.16). This is a reversal on low-dose aspirin for primary prevention of atherosclerotic events in patients with type 2 diabetes. | 2009. “When aspirin was compared with placebo there was no statistically significant reduction in the risk of major cardiovascular events… there are insufficient data among patients with diabetes to conclusively show a benefit of aspirin therapy for the primary prevention of cardiovascular events.”^139^ | Pubmed found systematic review |
| 353 | Vitamins E and C in the Prevention of Cardiovascular Disease in Men The Physicians' Health Study II Randomized Controlled Trial  Sesso et al. | Public health/ Preventive medicine | 11/12/2008 JAMA | The National Health and Nutrition Examination Survey reported that approximately 12% of US adults took vitamin E or C supplements.^134^ The PHS II trial examined whether overall rates of cardiovascular events would be lower if men took vitamin E or C supplements. After 8 years of follow up, neither vitamin E nor vitamin C supplementation reduced the risk of major cardiovascular events. Both the placebo Vitamin E group (n=7326) and the active Vitamin E group (n=7315) had an incidence rate of major cardiovascular events of 10.9 events per 1000 person-years (HR. 1.01; 95% CI, 0.90-1.13; P=.86). There was no significant difference in incidence of events in the placebo Vitamin C group (n=7312) and the active Vitamin C group (n=7329), at 10.9 vs 10.8 events per 1000 person-years (HR, 0.99; 95% CI, 0.89-1.11; P=.91). This is a reversal on Vitamins E and C for preventing cardiovascular disease in men. | 2017. Cochrane review. "Currently, there is no evidence to suggest that vitamin C supplementation reduces the risk of CVD in healthy participants and those at increased risk of CVD."^140^  2013. Vitamin, Mineral, and Multivitamin Supplements for the Primary Prevention of Cardiovascular Disease and Cancer. “Trials of vitamin E supplementation showed mixed results and altogether had no overall effect on cancer, CVD, or all-cause mortality.”^141^ | Pubmed found systematic review |
| 354 | Ginkgo biloba for Prevention of Dementia A Randomized Controlled Trial  DeKosky et al. | Neurology | 11/19/2008 JAMA | *Ginkgo biloba* supplement sales exceed $249 million annually.^142^ This herbal product is thought to preserve memory and is used in many medical practices around the world.^116^ The GEM study randomized patients to receive *G. biloba* (n=1545) or placebo (n=1524). GEM concluded that *G. biloba* did not reduce the incidence of dementia (HR, 1.12; 95% CI, 0.94-1.33; P=.21) or Alzheimer’s disease (HR, 1.13; 96% CI, 0.85-1.50; P=.39). This is a reversal of *G. biloba* for prevention of dementia. | 2010. “Many of the early trials [on Ginkgo] used unsatisfactory methods, were small, and publication bias cannot be excluded. Overall, evidence that Ginkgo has predictable and clinically significant benefit for people with dementia or cognitive impairment is inconsistent and unreliable.”^143^ | Pubmed found systematic review |
| 355 | Effect of Evidence-Based Feeding Guidelines on Mortality of Critically Ill Adults A Cluster Randomized Controlled Trial  Doig et al. | Critical care | 12/17/2008 JAMA | Evidence-based guidelines (EBGs) have been successful at reducing evidence-practice gaps by discouraging ineffective care.^144-146^ This randomized controlled trial investigated whether evidence-based guidelines would improve feeding practices and reduce mortality in ICU patients. The trial found that although the ICU developed and implemented an evidence-based nutritional support guideline promoting earlier feeding, the use of these guidelines did not affect clinical outcomes. The guideline group (n=561) and the control group (n=557) did not differ in hospital discharge mortality (28.9% vs 27.4%; difference, 1.4% [95% CI, -6.3%-12.0%], P=.75). This is a reversal of evidence-based feeding guidelines for reducing hospital discharge mortality in critically ill adults. | none |  |

1. Taylor A. Pediatric focused safety review: proton pump inhibitors: esomeprazole, lansoprazole, omeprazole, rabeprazole. *Washington, DC: US Food and Drug Administration* 2010

2. Hickner JM, Bartlett JG, Besser RE, et al. Principles of appropriate antibiotic use for acute rhinosinusitis in adults: background. *Annals of emergency medicine* 2001;37(6):703-10.

3. Steinman MA, Gonzales R, Linder JA, et al. Changing use of antibiotics in community-based outpatient practice, 19911999. *Annals of Internal Medicine* 2003;138(7):525-33.

4. Lemiengre MB, van Driel ML, Merenstein D, et al. Antibiotics for clinically diagnosed acute rhinosinusitis in adults. *Cochrane Database of Systematic Reviews* 2012(10) doi: 10.1002/14651858.CD006089.pub4

5. Kushner FG, Hand M, Smith SC, et al. 2009 Focused Updates: ACC/AHA Guidelines for the Management of Patients With ST‐Elevation Myocardial Infarction (Updating the 2004 Guideline and 2007 Focused Update) and ACC/AHA/SCAI Guidelines on Percutaneous Coronary Intervention (Updating the 2005 Guideline and 2007 Focused Update). *Catheterization and Cardiovascular Interventions* 2009;74(7)

6. Jolly SS, James S, Džavík V, et al. Thrombus Aspiration in ST-Segment–Elevation Myocardial InfarctionClinical Perspective: An Individual Patient Meta-Analysis: Thrombectomy Trialists Collaboration. *Circulation* 2017;135(2):143-52.

7. Deng S-B, Wang J, Xiao J, et al. Adjunctive Manual Thrombus Aspiration during ST-Segment Elevation Myocardial Infarction: A Meta-Analysis of Randomized Controlled Trials. *PLoS ONE* 2014;9(11):e113481. doi: 10.1371/journal.pone.0113481

8. Punjabi NM. The epidemiology of adult obstructive sleep apnea. *Proceedings of the American Thoracic Society* 2008;5(2):136-43.

9. Force AOSAT, Medicine AAoS. Clinical guideline for the evaluation, management and long-term care of obstructive sleep apnea in adults. *Journal of clinical sleep medicine: JCSM: official publication of the American Academy of Sleep Medicine* 2009;5(3):263.

10. Lattimore J-DL, Celermajer DS, Wilcox I. Obstructive sleep apnea and cardiovascular disease. *Journal of the American College of Cardiology* 2003;41(9):1429-37.

11. Bratton DJ, Stradling JR, Barbé F, et al. Effect of CPAP on blood pressure in patients with minimally symptomatic obstructive sleep apnoea: a meta-analysis using individual patient data from four randomised controlled trials. *Thorax* 2014;69(12):1128-35. doi: 10.1136/thoraxjnl-2013-204993

12. Abenavoli L, Capasso R, Milic N, et al. Milk thistle in liver diseases: past, present, future. *Phytotherapy Research* 2010;24(10):1423-32.

13. Seeff LB, Curto TM, Szabo G, et al. Herbal product use by persons enrolled in the hepatitis C Antiviral Long‐Term Treatment Against Cirrhosis (HALT‐C) Trial. *Hepatology* 2008;47(2):605-12.

14. Morishima C, Shuhart MC, Wang CC, et al. Silymarin inhibits in vitro T-cell proliferation and cytokine production in hepatitis C virus infection. *Gastroenterology* 2010;138(2):671-81. e2.

15. Rambaldi A, Jacobs BP, Gluud C. Milk thistle for alcoholic and/or hepatitis B or C virus liver diseases. *Cochrane Database of Systematic Reviews* 2007(4) doi: 10.1002/14651858.CD003620.pub3

16. Yang Z, Zhuang L, Lu Y, et al. Effects and Tolerance of Silymarin (Milk Thistle) in Chronic Hepatitis C Virus Infection Patients: A Meta-Analysis of Randomized Controlled Trials. *BioMed Research International* 2014;2014:941085. doi: 10.1155/2014/941085

17. Waalen J, Goodwin MM, Spitz AM, et al. Screening for intimate partner violence by health care providers: barriers and interventions. *American journal of preventive medicine* 2000;19(4):230-37.

18. Force UPST. Screening for family and intimate partner violence: recommendation statement. *The Annals of Family Medicine* 2004;2(2):156-60.

19. Rhodes KV, Lauderdale DS, He T, et al. “Between me and the computer”: increased detection of intimate partner violence using a computer questionnaire. *Annals of emergency medicine* 2002;40(5):476-84.

20. O'Doherty L, Hegarty K, Ramsay J, et al. Screening women for intimate partner violence in healthcare settings. *Cochrane Database of Systematic Reviews* 2015(7) doi: 10.1002/14651858.CD007007.pub3

21. Alabed S, Sabouni A, Al Dakhoul S, et al. Beta-blockers for congestive heart failure in children. *Cochrane Database of Systematic Reviews* 2016(1) doi: 10.1002/14651858.CD007037.pub3

22. Muncie Jr HL, Koch H. Prescription and recommendation of multivitamins by physicians in office based ambulatory care in the United States. *Nutrition Research* 1988;8(10):1129-41.

23. Schwingshackl L, Boeing H, Stelmach-Mardas M, et al. Dietary Supplements and Risk of Cause-Specific Death, Cardiovascular Disease, and Cancer: A Systematic Review and Meta-Analysis of Primary Prevention Trials. *Advances in Nutrition* 2017;8(1):27-39. doi: 10.3945/an.116.013516

24. Chaney MA. Corticosteroids and cardiopulmonary bypass: a review of clinical investigations. *Chest* 2002;121(3):921-31.

25. Murphy GS, Whitlock RP, Gutsche JT, et al. Steroids for adult cardiac surgery with cardiopulmonary bypass: update on dose and key randomized trials. *Journal of cardiothoracic and vascular anesthesia* 2013;27(5):1053-59.

26. Kristeller JL, Jankowski A, Reinaker T. Role of Corticosteroids During Cardiopulmonary Bypass. *Hospital Pharmacy* 2014;49(3):232-36. doi: 10.1310/hpj4903-232

27. Arenth PM, Russell KC, Ricker JH, et al. CDP-Choline as a Biological Supplement During Neurorecovery: A Focused Review. *PM&R* 2011;3(6, Supplement):S123-S31. doi: <https://doi.org/10.1016/j.pmrj.2011.03.012>

28. Meshkini A, Meshkini M, Sadeghi-Bazargani H. Citicoline for traumatic brain injury: a systematic review & meta-analysis. *Journal of Injury and Violence Research* 2017;9(1):41-50. doi: 10.5249/jivr.v9i1.843

29. Kris-Etherton PM, Harris WS, Appel LJ. Fish Consumption, Fish Oil, Omega-3 Fatty Acids, and Cardiovascular Disease. *Circulation* 2002;106(21):2747-57. doi: 10.1161/01.cir.0000038493.65177.94

30. Anand RG, Alkadri M, Lavie CJ, et al. The role of fish oil in arrhythmia prevention. *Journal of cardiopulmonary rehabilitation and prevention* 2008;28(2):92-98.

31. Mariani J, Doval HC, Nul D, et al. N-3 polyunsaturated fatty acids to prevent atrial fibrillation: updated systematic review and meta-analysis of randomized controlled trials. *Journal of the American Heart Association* 2013;2(1):e005033.

32. Penson DF, McLerran D, Feng Z, et al. 5-year urinary and sexual outcomes after radical prostatectomy: results from the prostate cancer outcomes study. *The Journal of urology* 2005;173(5):1701-5. doi: 10.1097/01.ju.0000154637.38262.3a [published Online First: 2005/04/12]

33. Penson DF, McLerran D, Feng Z, et al. 5-YEAR URINARY AND SEXUAL OUTCOMES AFTER RADICAL PROSTATECTOMY: RESULTS FROM THE PROSTATE CANCER OUTCOMES STUDY. *The Journal of Urology* 2005;173(5):1701-05. doi: <https://doi.org/10.1097/01.ju.0000154637.38262.3a>

34. Wyman JF. Treatment of Urinary Incontinence in Men and Older Women: The evidence shows the efficacy of a variety of techniques. *AJN The American Journal of Nursing* 2003;103:26-35.

35. Ayeleke RO, Hay-Smith EJC, Omar MI. Pelvic floor muscle training added to another active treatment versus the same active treatment alone for urinary incontinence in women. *Cochrane Database of Systematic Reviews* 2015(11) doi: 10.1002/14651858.CD010551.pub3

36. Hoskins M, Pearce J, Bethell A, et al. Pharmacotherapy for post-traumatic stress disorder: systematic review and meta-analysis. *The British Journal of Psychiatry* 2015;206(2):93-100. doi: 10.1192/bjp.bp.114.148551

37. Fagelman E, Lowe FC. Saw Palmetto Berry as a Treatment for BPH. *Reviews in Urology* 2001;3(3):134-38.

38. Tacklind J, MacDonald R, Rutks I, et al. Serenoa repens for benign prostatic hyperplasia. *Cochrane Database of Systematic Reviews* 2012(12) doi: 10.1002/14651858.CD001423.pub3

39. Lippman SM, Klein EA, Goodman PJ, et al. Effect of selenium and vitamin e on risk of prostate cancer and other cancers: The selenium and vitamin e cancer prevention trial (select). *JAMA* 2009;301(1):39-51. doi: 10.1001/jama.2008.864

40. Chan JM, Stampfer MJ, Ma J, et al. Supplemental Vitamin E Intake and Prostate Cancer Risk in a Large Cohort of Men in the United States. *Cancer Epidemiology Biomarkers &amp; Prevention* 1999;8(10):893-99.

41. Fortmann S, Burda B, Senger C, et al. Vitamin, mineral, and multivitamin supplements for the primary prevention of cardiovascular disease and cancer: a systematic evidence review for the US Preventive Services Task Force [Internet]. Rockville (MD): Agency for Healthcare Research and Quality; 2013 [cited 2016 Aug 31], 2013.

42. Johnson ER, Matthay MA. Acute Lung Injury: Epidemiology, Pathogenesis, and Treatment. *Journal of Aerosol Medicine and Pulmonary Drug Delivery* 2010;23(4):243-52. doi: 10.1089/jamp.2009.0775

43. Heyland DK, Dhaliwal R, Drover JW, et al. Canadian clinical practice guidelines for nutrition support in mechanically ventilated, critically ill adult patients. *Journal of Parenteral and Enteral nutrition* 2003;27(5):355-73.

44. Gadek JE, DeMichele SJ, Karlstad MD, et al. Effect of enteral feeding with eicosapentaenoic acid, gamma-linolenic acid, and antioxidants in patients with acute respiratory distress syndrome. *Critical care medicine* 1999;27(8):1409-20.

45. Li C, Bo L, Liu W, et al. Enteral Immunomodulatory Diet (Omega-3 Fatty Acid, γ-Linolenic Acid and Antioxidant Supplementation) for Acute Lung Injury and Acute Respiratory Distress Syndrome: An Updated Systematic Review and Meta-Analysis. *Nutrients* 2015;7(7):5572-85. doi: 10.3390/nu7075239

46. Eppley BL, Woodell JE, Higgins J. Platelet quantification and growth factor analysis from platelet-rich plasma: implications for wound healing. *Plastic and reconstructive surgery* 2004;114(6):1502-08.

47. Sampson S, Gerhardt M, Mandelbaum B. Platelet rich plasma injection grafts for musculoskeletal injuries: a review. *Current Reviews in Musculoskeletal Medicine* 2008;1(3):165-74. doi: 10.1007/s12178-008-9032-5

48. Kearney RS, Parsons N, Metcalfe D, et al. Injection therapies for Achilles tendinopathy. *Cochrane Database of Systematic Reviews* 2015(5) doi: 10.1002/14651858.CD010960.pub2

49. Dellinger RP, Carlet JM, Masur H, et al. Surviving Sepsis Campaign guidelines for management of severe sepsis and septic shock. *Intensive care medicine* 2004;30(4):536-55.

50. Picard KM, O'Donoghue SC, Young-Kershaw DA, et al. Development and implementation of a multidisciplinary sepsis protocol. *Crit Care Nurse* 2006;26(3):43-54. [published Online First: 2006/05/24]

51. Van den Berghe G, Wouters P, Weekers F, et al. Intensive Insulin Therapy in Critically Ill Patients. *New England Journal of Medicine* 2001;345(19):1359-67. doi: 10.1056/NEJMoa011300

52. Brunkhorst FM, Engel C, Bloos F, et al. Intensive Insulin Therapy and Pentastarch Resuscitation in Severe Sepsis. *New England Journal of Medicine* 2008;358(2):125-39. doi: 10.1056/NEJMoa070716

53. Song F, Zhong L-J, Han L, et al. Intensive Insulin Therapy for Septic Patients: A Meta-Analysis of Randomized Controlled Trials. *BioMed Research International* 2014;2014:698265. doi: 10.1155/2014/698265

54. Plummer AL, Gracey DR. Consensus Conference on Artificial Airways in Patients Receiving Mechanical Ventilation. *Chest* 1989;96(1):178-80. doi: <https://doi.org/10.1378/chest.96.1.178>

55. Nathens AB, Rivara FP, Mack CD, et al. Variations in rates of tracheostomy in the critically ill trauma patient. *Critical care medicine* 2006;34(12):2919-24.

56. Andriolo BNG, Andriolo RB, Saconato H, et al. Early versus late tracheostomy for critically ill patients. *Cochrane Database of Systematic Reviews* 2015(1) doi: 10.1002/14651858.CD007271.pub3

57. Bischoff-Ferrari HA, Willett WC, Wong JB, et al. Fracture prevention with vitamin d supplementation: A meta-analysis of randomized controlled trials. *JAMA* 2005;293(18):2257-64. doi: 10.1001/jama.293.18.2257

58. guidelines R. Fracture Prevention Protocol- For all men and women over 50 years with a Fracture

2012 [Available from: <https://www.aci.health.nsw.gov.au/ie/projects/osteoporosis/fracture-protocol.pdf>.

59. Vitamin D- fact sheet for health professionals. Office of Dietary Supplements. February 11 ed: National Institutes of Health

2016.

60. Avenell A, Mak JCS, O'Connell D. Vitamin D and vitamin D analogues for preventing fractures in post-menopausal women and older men. *Cochrane Database of Systematic Reviews* 2014(4) doi: 10.1002/14651858.CD000227.pub4

61. Avins AL. Glucosamine and the ongoing enigma of chronic low back pain. *JAMA* 2010;304(1):93-94. doi: 10.1001/jama.2010.922

62. Santaguida PL, Gross A, Busse J, et al. Complementary and alternative medicine in back pain utilization report. *Evid Rep Technol Assess (Full Rep)* 2009;177(January):1-221.

63. Sodha R, Sivanadarajah N, Alam M. The use of glucosamine for chronic low back pain: a systematic review of randomised control trials. *BMJ Open* 2013;3(6):e001167. doi: 10.1136/bmjopen-2012-001167

64. Cohen M, Urban P, Christenson JT, et al. Intra-aortic balloon counterpulsation in US and non-US centres: results of the Benchmark® Registry. *European Heart Journal* 2003;24(19):1763-70. doi: 10.1016/j.ehj.2003.07.002

65. Smith SC, Feldman TE, Hirshfeld JW, et al. ACC/AHA/SCAI 2005 Guideline Update for Percutaneous Coronary Intervention: A Report of the American College of Cardiology/American Heart Association Task Force on Practice Guidelines (ACC/AHA/SCAI Writing Committee to Update the 2001 Guidelines for Percutaneous Coronary Intervention). *Journal of the American College of Cardiology* 2006;47(1):e1-e121. doi: <https://doi.org/10.1016/j.jacc.2005.12.001>

66. Zheng X-y, Wang Y, Chen Y, et al. The effectiveness of intra-aortic balloon pump for myocardial infarction in patients with or without cardiogenic shock: a meta-analysis and systematic review. *BMC Cardiovascular Disorders* 2016;16:148. doi: 10.1186/s12872-016-0323-2

67. Rogers MA, Blumberg N, Saint S, et al. Hospital variation in transfusion and infection after cardiac surgery: a cohort study. *BMC medicine* 2009;7(1):37.

68. Carson JL, Hill S, Carless P, et al. Transfusion triggers: a systematic review of the literature. *Transfusion medicine reviews* 2002;16(3):187-99.

69. Carson JL, Stanworth SJ, Roubinian N, et al. Transfusion thresholds and other strategies for guiding allogeneic red blood cell transfusion. *Cochrane Database Syst Rev* 2016;10:Cd002042. doi: 10.1002/14651858.CD002042.pub4 [published Online First: 2016/11/02]

70. Koletzko B, Cetin I, Thomas Brenna J. Dietary fat intakes for pregnant and lactating women. *British Journal of Nutrition* 2007;98(5):873-77. doi: 10.1017/S0007114507764747 [published Online First: 11/01]

71. Brenna JT, Lapillonne A. Background Paper on Fat and Fatty Acid Requirements during Pregnancy and Lactation. *Annals of Nutrition and Metabolism* 2009;55(1-3):97-122.

72. Golding J, Steer C, Emmett P, et al. High Levels of Depressive Symptoms in Pregnancy With Low Omega-3 Fatty Acid Intake From Fish. *Epidemiology* 2009;20(4):598-603. doi: 10.1097/EDE.0b013e31819d6a57

73. Martek. Martek Biosciences Corporation Annual Report 2007, 2007.

74. Miller BJ, Murray L, Beckmann MM, et al. Dietary supplements for preventing postnatal depression. *Cochrane Database of Systematic Reviews* 2013(10) doi: 10.1002/14651858.CD009104.pub2

75. Taylor RM, Fealy SM, Bisquera A, et al. Effects of Nutritional Interventions during Pregnancy on Infant and Child Cognitive Outcomes: A Systematic Review and Meta-Analysis. *Nutrients* 2017;9(11):1265. doi: 10.3390/nu9111265

76. Swaminathan A, Jicha GA. Nutrition and prevention of Alzheimer’s dementia. *Frontiers in Aging Neuroscience* 2014;6:282. doi: 10.3389/fnagi.2014.00282

77. Cole GM, Frautschy SA. DHA May Prevent Age-Related Dementia. *The Journal of Nutrition* 2010;140(4):869-74. doi: 10.3945/jn.109.113910

78. Burckhardt M, Herke M, Wustmann T, et al. Omega-3 fatty acids for the treatment of dementia. *Cochrane Database of Systematic Reviews* 2016(4) doi: 10.1002/14651858.CD009002.pub3

79. investigators G-H. Effect of n-3 polyunsaturated fatty acids in patients with chronic heart failure (the GISSI-HF trial): a randomised, double-blind, placebo-controlled trial. *The Lancet* 2008;372(9645):1223-30. doi: <https://doi.org/10.1016/S0140-6736(08)61239-8>

80. Calò L, Bianconi L, Colivicchi F, et al. N-3 Fatty Acids for the Prevention of Atrial Fibrillation After Coronary Artery Bypass Surgery: A Randomized, Controlled Trial. *Journal of the American College of Cardiology* 2005;45(10):1723-28. doi: <https://doi.org/10.1016/j.jacc.2005.02.079>

81. Bays HE. Safety Considerations with Omega-3 Fatty Acid Therapy. *The American Journal of Cardiology* 2007;99(6, Supplement 1):S35-S43. doi: <https://doi.org/10.1016/j.amjcard.2006.11.020>

82. Harris SR, Niesen-Vertommen SL. Challenging the myth of exercise-induced lymphedema following breast cancer: A series of case reports. *Journal of Surgical Oncology* 2000;74(2):95-98. doi: 10.1002/1096-9098(200006)74:2<95::AID-JSO3>3.0.CO;2-Q

83. Ridner SH. Breast Cancer Lymphedema: Pathophysiology and Risk Reduction Guidelines. *Oncology Nursing Forum* 2002;29(9):1285-93.

84. Stuiver MM, ten Tusscher MR, Agasi-Idenburg CS, et al. Conservative interventions for preventing clinically detectable upper-limb lymphoedema in patients who are at risk of developing lymphoedema after breast cancer therapy. *Cochrane Database of Systematic Reviews* 2015(2) doi: 10.1002/14651858.CD009765.pub2

85. Blot WJ, Li J-Y, Taylor PR, et al. Nutrition Intervention Trials in Linxian, China: Supplementation With Specific Vitamin/Mineral Combinations, Cancer Incidence, and Disease-Specific Mortality in the General Population. *JNCI: Journal of the National Cancer Institute* 1993;85(18):1483-91. doi: 10.1093/jnci/85.18.1483

86. Vinceti M, Dennert G, Crespi CM, et al. Selenium for preventing cancer. *Cochrane Database of Systematic Reviews* 2014(3) doi: 10.1002/14651858.CD005195.pub3

87. Kaegi E. Unconventional therapies for cancer: 5. Vitamins A, C and E. *CMAJ: Canadian Medical Association Journal* 1998;158(11):1483.

88. Marmot M, Atinmo T, Byers T, et al. Food, nutrition, physical activity, and the prevention of cancer: a global perspective. 2007

89. The H, Investigators H-TT. Effects of long-term vitamin e supplementation on cardiovascular events and cancer: A randomized controlled trial. *JAMA* 2005;293(11):1338-47. doi: 10.1001/jama.293.11.1338

90. Grundy SM, Benjamin IJ, Burke GL, et al. Diabetes and cardiovascular disease: a statement for healthcare professionals from the American Heart Association. *Circulation* 1999;100(10):1134-46.

91. Rajagopalan N, Miller TD, Hodge DO, et al. Identifying high-risk asymptomatic diabetic patients who are candidates for screening stress single-photon emission computed tomography imaging. *Journal of the American College of Cardiology* 2005;45(1):43-49.

92. Scognamiglio R, Negut C, Ramondo A, et al. Detection of coronary artery disease in asymptomatic patients with type 2 diabetes mellitus. *Journal of the American College of Cardiology* 2006;47(1):65-71.

93. Wackers FJT, Young LH, Inzucchi SE, et al. Detection of Silent Myocardial Ischemia in Asymptomatic Diabetic Subjects. *Diabetes care* 2004;27(8)

94. Bauters C, Lemesle G. Screening for asymptomatic coronary artery disease in patients with diabetes mellitus: A systematic review and meta-analysis of randomized trials. *BMC Cardiovascular Disorders* 2016;16:90. doi: 10.1186/s12872-016-0256-9

95. Latthe P, Powell RJ, Daniels J, et al. Variation in practice of laparoscopic uterosacral nerve ablation: a European survey. *Journal of Obstetrics and Gynaecology* 2004;24(5):547-51.

96. Daniels J, Gray R, Khan KS, et al. Laparoscopic uterine nerve ablation: a survey of gynaecological practice in the UK. *Gynaecological Endoscopy* 2000;9(3):157-59. doi: 10.1046/j.1365-2508.2000.00323.x

97. Latthe PM, Champaneria R. Dysmenorrhoea. *BMJ Clinical Evidence* 2011;2011:0813.

98. Organization WH. Global guidelines for the prevention of surgical site infection: World Health Organization 2016.

99. Qadan M, Akça O, Mahid SS, et al. Perioperative supplemental oxygen therapy and surgical site infection: A meta-analysis of randomized controlled trials. *Archives of Surgery* 2009;144(4):359-66. doi: 10.1001/archsurg.2009.1

100. Pryor KO, Fahey IT, Lien CA, et al. Surgical site infection and the routine use of perioperative hyperoxia in a general surgical population: A randomized controlled trial. *JAMA* 2004;291(1):79-87. doi: 10.1001/jama.291.1.79

101. Wetterslev J, Meyhoff CS, Jørgensen LN, et al. The effects of high perioperative inspiratory oxygen fraction for adult surgical patients. *Cochrane Database of Systematic Reviews* 2015(6) doi: 10.1002/14651858.CD008884.pub2

102. Luckhaupt SE, Sweeney MH, Funk R, et al. Influenza-associated Hospitalizations by Industry, 2009–10 Influenza Season, United States. *Emerging Infectious Diseases* 2012;18(4):556-62. doi: 10.3201/eid1804.110337

103. Siegel JD, Rhinehart E. Health Care Infection Control Practices Advisory Committee. 2007 Guideline for isolation precautions: preventing transmission of infectious agents in health care settings. 2007

104. Krah J, Novak D, Stradtman L. Preparedness through daily practice: the myths of respiratory protection in healthcare. 2016

105. Smith JD, MacDougall CC, Johnstone J, et al. Effectiveness of N95 respirators versus surgical masks in protecting health care workers from acute respiratory infection: a systematic review and meta-analysis. *CMAJ : Canadian Medical Association Journal* 2016;188(8):567-74. doi: 10.1503/cmaj.150835

106. Dellinger RP, Levy MM, Carlet JM, et al. Surviving Sepsis Campaign: international guidelines for management of severe sepsis and septic shock: 2008. *Intensive care medicine* 2008;34(1):17-60.

107. Bloomfield R, Noble DW, Sudlow A. Prone position for acute respiratory failure in adults. *Cochrane Database of Systematic Reviews* 2015(11) doi: 10.1002/14651858.CD008095.pub2

108. Part 7.2: Management of Cardiac Arrest. *Circulation* 2005;112(24 suppl):IV-58-IV-66. doi: 10.1161/circulationaha.105.166557

109. Holmberg M, Holmberg S, Herlitz J. Low chance of survival among patients requiring adrenaline (epinephrine) or intubation after out-of-hospital cardiac arrest in Sweden. *Resuscitation* 2002;54(1):37-45. doi: <https://doi.org/10.1016/S0300-9572(02)00048-5>

110. Olasveengen TM, Sunde K, Brunborg C, et al. Intravenous drug administration during out-of-hospital cardiac arrest: A randomized trial. *JAMA* 2009;302(20):2222-29. doi: 10.1001/jama.2009.1729

111. Atiksawedparit P, Rattanasiri S, McEvoy M, et al. Effects of prehospital adrenaline administration on out-of-hospital cardiac arrest outcomes: a systematic review and meta-analysis. *Critical Care* 2014;18(4):463. doi: 10.1186/s13054-014-0463-7

112. Steinbrook R. Public Report Cards — Cardiac Surgery and Beyond. *New England Journal of Medicine* 2006;355(18):1847-49. doi: 10.1056/NEJMp068222

113. Hibbard JH, Stockard J, Tusler M. Does publicizing hospital performance stimulate quality improvement efforts? *Health Affairs* 2003;22(2):84-94.

114. Werner RM, Asch DA. The unintended consequences of publicly reporting quality information. *JAMA* 2005;293(10):1239-44. doi: 10.1001/jama.293.10.1239

115. Ketelaar NABM, Faber MJ, Flottorp S, et al. Public release of performance data in changing the behaviour of healthcare consumers, professionals or organisations. *Cochrane Database of Systematic Reviews* 2011(11) doi: 10.1002/14651858.CD004538.pub2

116. DeKosky ST, Williamson JD, Fitzpatrick AL, et al. Ginkgo biloba for prevention of dementia: A randomized controlled trial. *JAMA* 2008;300(19):2253-62. doi: 10.1001/jama.2008.683

117. Barnes PM, Bloom B, Nahin RL. Complementary and alternative medicine use among adults and children; United States, 2007. 2008

118. Kane RL, Butler M, Fink HA, et al. Interventions to prevent age-related cognitive decline, mild cognitive impairment, and clinical alzheimer’s-type dementia. 2017

119. Tan RS, Salazar JA. Risks of testosterone replacement therapy in ageing men. *Expert opinion on drug safety* 2004;3(6):599-606. [published Online First: 2004/10/27]

120. Tenover JS. Effects of testosterone supplementation in the aging male. *The Journal of Clinical Endocrinology & Metabolism* 1992;75(4):1092-98.

121. Huo S, Scialli AR, McGarvey S, et al. Treatment of Men for “Low Testosterone”: A Systematic Review. *PLoS ONE* 2016;11(9):e0162480. doi: 10.1371/journal.pone.0162480

122. Li FX, Verhoef MJ, Best A, et al. Why patients with inflammatory bowel disease use or do not use complementary and alternative medicine: a Canadian national survey. *Canadian Journal of Gastroenterology and Hepatology* 2005;19(9):567-73.

123. Langmead L, Rampton D. Complementary and alternative therapies for inflammatory bowel disease. *Alimentary pharmacology & therapeutics* 2006;23(3):341-49.

124. Lev-Tzion R, Griffiths AM, Ledder O, et al. Omega 3 fatty acids (fish oil) for maintenance of remission in Crohn's disease. *Cochrane Database of Systematic Reviews* 2014(2) doi: 10.1002/14651858.CD006320.pub4

125. Parienti J, Thirion M, Mégarbane B, et al. Femoral vs jugular venous catheterization and risk of nosocomial events in adults requiring acute renal replacement therapy: A randomized controlled trial. *JAMA* 2008;299(20):2413-22. doi: 10.1001/jama.299.20.2413

126. Moncrief JA. Femoral Catheters. *Annals of Surgery* 1958;147(2):166-72.

127. III. NKF-K/DOQI Clinical Practice Guidelines for Vascular Access: Update 2000. *American Journal of Kidney Diseases* 2001;37(1, Supplement):S137-S81. doi: <https://doi.org/10.1016/S0272-6386(01)70007-8>

128. Ge X, Cavallazzi R, Li C, et al. Central venous access sites for the prevention of venous thrombosis, stenosis and infection. *Cochrane Database of Systematic Reviews* 2012(3) doi: 10.1002/14651858.CD004084.pub3

129. Sarris J, Kean J, Schweitzer I, et al. Complementary medicines (herbal and nutritional products) in the treatment of Attention Deficit Hyperactivity Disorder (ADHD): A systematic review of the evidence. *Complementary Therapies in Medicine* 2011;19(4):216-27. doi: <https://doi.org/10.1016/j.ctim.2011.06.007>

130. Nygård O, Nordrehaug JE, Refsum H, et al. Plasma Homocysteine Levels and Mortality in Patients with Coronary Artery Disease. *New England Journal of Medicine* 1997;337(4):230-37. doi: 10.1056/nejm199707243370403

131. Ebbing M, Bleie Ø, Ueland P, et al. Mortality and cardiovascular events in patients treated with homocysteine-lowering b vitamins after coronary angiography: A randomized controlled trial. *JAMA* 2008;300(7):795-804. doi: 10.1001/jama.300.7.795

132. Martí-Carvajal AJ, Solà I, Lathyris D, et al. Homocysteine-lowering interventions for preventing cardiovascular events. *Cochrane Database of Systematic Reviews* 2017(8) doi: 10.1002/14651858.CD006612.pub5

133. Bernardi E, Camporese G, Büller HR, et al. Serial 2-point ultrasonography plus d-dimer vs whole-leg color-coded doppler ultrasonography for diagnosing suspected symptomatic deep vein thrombosis: A randomized controlled trial. *JAMA* 2008;300(14):1653-59. doi: 10.1001/jama.300.14.1653

134. Radimer K, Bindewald B, Hughes J, et al. Dietary supplement use by US adults: data from the National Health and Nutrition Examination Survey, 1999–2000. *American journal of epidemiology* 2004;160(4):339-49.

135. Giovannucci E. Epidemiologic Studies of Folate and Colorectal Neoplasia: a Review. *The Journal of Nutrition* 2002;132(8):2350S-55S. doi: 10.1093/jn/132.8.2350S

136. Larsson SC, Giovannucci E, Wolk A. Folate and risk of breast cancer: a meta-analysis. *Journal of the National Cancer Institute* 2007;99(1):64-76.

137. Fortmann SP, Burda BU, Senger CA, et al. Vitamin and mineral supplements in the primary prevention of cardiovascular disease and cancer: An updated systematic evidence review for the u.s. preventive services task force. *Annals of Internal Medicine* 2013;159(12):824-34. doi: 10.7326/0003-4819-159-12-201312170-00729

138. Association AD. Aspirin therapy in diabetes. *Diabetes care* 2004;27(suppl 1):s72-s73.

139. Calvin AD, Aggarwal NR, Murad MH, et al. Aspirin for the Primary Prevention of Cardiovascular Events. *A systematic review and meta-analysis comparing patients with and without diabetes* 2009;32(12):2300-06. doi: 10.2337/dc09-1297

140. Al-Khudairy L, Flowers N, Wheelhouse R, et al. Vitamin C supplementation for the primary prevention of cardiovascular disease. *Cochrane Database of Systematic Reviews* 2017(3) doi: 10.1002/14651858.CD011114.pub2

141. Moyer VA. Vitamin, mineral, and multivitamin supplements for the primary prevention of cardiovascular disease and cancer: US Preventive Services Task Force recommendation statement. *Annals of internal medicine* 2014;160(8):558-64.

142. Nutritional Business Journal 2006 Supplement Business Report. San Diego, CA: Penton Media Inc, 2006.

143. Weinmann S, Roll S, Schwarzbach C, et al. Effects of Ginkgo biloba in dementia: systematic review and meta-analysis. *BMC Geriatrics* 2010;10:14-14. doi: 10.1186/1471-2318-10-14

144. Grimshaw J, Eccles M, Thomas R, et al. Toward evidence‐based quality improvement. *Journal of general internal medicine* 2006;21(S2)

145. Cook DJ, Greengold NL, Ellrodt AG, et al. The relation between systematic reviews and practice guidelines. *Annals of internal medicine* 1997;127(3):210-16.

146. Grol R, Hutchinson A, Eccles M, et al. Clinical guidelines, potential benefits, limitations, and harms of guidelines. *BMJ* 1999;318:527-30.

| **#** | **Article and Author** | **Primary Medical Discipline** | **Date and Journal** | **Summary** | **Systematic Review** | **Systematic Review Search Terms** |
| --- | --- | --- | --- | --- | --- | --- |
| 356 | Cognitive and Cardiac Outcomes 5 Years After Off-Pump vs On-Pump Coronary Artery Bypass Graft Surgery  Dijk et al. | Cardiovascular | 2/21/2007 JAMA | Observational studies in the 1990s showed an association between off-pump coronary-artery bypass and better early clinical outcomes compared to on-pump, and the practice of performing coronary-artery bypass surgery on a beating heart experienced a resurgence.^1^ Yet randomized controlled trials have not been able to show efficacy in off-pump surgeries and have suggested that incomplete revascularization was more frequent with off-pump surgery.^2^ This follow-up study compared the 5-year outcomes of cognitive status and cardiovascular events in low-risk patients receiving either on-pump (n=117) or off-pump (n=123) CABG. The study found that 62/123 (50.4%) of the participants in the off-pump group experienced cognitive decline compared to 59/117 (50.4%) in the on-pump group (absolute difference, 0; 95% CI, -12.7%-12.6%; P>.99). The off-pump group experienced 30 cardiovascular events (21.1%) vs 25 events (18.0%) in the on-pump group (absolute difference, 3.1%; 95% CI, -6.1%-12.4%; P=.55). This is a reversal of the practice of off-pump coronary artery bypass surgery in place of on-pump surgery for reducing cardiovascular events and cognitive decline. | 2012. Cochrane review. "Our systematic review did not demonstrate any significant benefit of off-pump compared with on-pump CABG regarding mortality, stroke, or myocardial infarction. In contrast, we observed better long-term survival in the group of patients undergoing on-pump CABG with the use of cardiopulmonary bypass and cardioplegic arrest. Based on the current evidence, on-pump CABG should continue to be the standard surgical treatment. However, off-pump CABG may be acceptable when there are contraindications for cannulation of the aorta and cardiopulmonary bypass. Further randomised clinical trials should address the optimal treatment in such patients."^3^ | Pubmed found systematic review |
| 357 | Levosimendan vs Dobutamine for Patients With Acute Decompensated Heart Failure The SURVIVE Randomized Trial  Mebazaa et al. | Cardiovascular | 5/02/2007 JAMA | Patients with acute decompensated heart failure (ADHF) who show evidence of peripheral hypoperfusion often receive positive inotropes such as dobutamine. The agents improve hemodynamics and symptoms but have been associated with increased risk of death and other cardiovascular events.^4^ Levosimendan is a pharmacological agent that exerts positive inotropic effects and vasodilatory properties and has been approved and used for ADHF in over 60 countries worldwide.^5^ Levosimendan was not found to increase incidence of ischaemia or hypotension and was associated with a lower risk of death in retrospective analysis.^6^ This study found that compared to dobutamine (n=663), patients with ADHF receiving levosimendan (n=664) had reduced plasma B-type natriuretic peptide levels, but did not reduce all-cause mortality at 180 days (HR, 0.91; 95% CI, 0.74-1.13; P=.40). This is a reversal of levosimendan for lowering risk of all-cause mortality in patients with ADHF. | 2018. Cochrane review. “Apart from low quality of evidence data suggesting a short-term mortality benefit of levosimendan compared with dobutamine, at present there are no robust and convincing data to support a distinct inotropic or vasodilator drug-based therapy as a superior solution to reduce mortality in haemodynamically unstable people with cardiogenic shock or LCOS [low cardiac output syndrome].”^7^ | pubmed found systematic review |
| 358 | Influence of a Diet Very High in Vegetables, Fruit, and Fiber and Low in Fat on Prognosis Following Treatment for Breast Cancer The Women's Healthy Eating and Living (WHEL) Randomized Trial  Pierce et al. | Public Health/ Preventive Medicine | 7/18/2007 JAMA | Observational studies suggest that diets high in vegetables and fruit and low in fat are associated with a lower risk of breast cancer,^8^ and guidelines have been recommending this diet for cancer prevention.^9^ The Women's Intervention Nutrition Study, which investigated diets that reduce fat intake on relapse-free survival in breast cancer patients, found a modest but significant improvement in relapse-free survival among those assigned to a low-fat diet.^10^ This study sought to determine the effect of high vegetable, fruit, and fiber and low fat diet in women with previously treated early stage breast cancer. The study found that the intervention diet (n=1537) compared to standard diet (n=1551) did not reduce breast cancer events (adjusted HR, 0.96; 95% CI, 0.80-1.14; P = .63), or mortality (adjusted HR, 0.91; 95% CI, 0.72-1.15; P = .43) after a 7.3-year follow-up period. This is a reversal of diets high in vegetable, fiber, and fruit and low in fat in reducing risk of breast cancer events following treatment. | 2009. “There is no convincing evidence that changing dietary pattern following breast cancer diagnosis will improve prognosis for most women with early stage breast cancer. However, it would appear to be important for some subgroups. Further investigation of mechanisms for such selective action is needed.”^11^ | Source 1: pubmed found systematic review  Source 2: pubmed search of article title |
| 359 | Efficacy of a Hip Protector to Prevent Hip Fracture in Nursing Home Residents The HIP PRO Randomized Controlled Trial  Kiel et al. | Orthopedic | 7/25/2007 JAMA | Hip fractures caused by falling are common among the older adults in the United States, with the highest incident rates in nursing homes, where 50% of residents fall each year.^12^ Hip protectors were designed to prevent fractures by diverting or absorbing energy of a fall. Although hip protectors were widely used,^13^ their effectiveness in institutional settings is uncertain.^14^ This study found that energy-absorbing/shunting hip protectors (n=1042) did not have a protective effect on the risk of hip fracture compared to no hip protector (n=1042) in patients residing in US nursing homes. The incidence rate of hip fracture for the hip protector group was 3.1% (95% CI, 1.8%-4.4%) vs the rate of the no hip protector at 2.5% (95% CI, 1.3%-3.7%; P=.70). This is a reversal of hip protectors for preventing hip fractures in nursing homes. | 2014. Cochrane review. After excluding studies with high risk of bias, this Cochrane systematic review found that hip protectors did not have a significant effect on risk of hip fractures in institutional settings.^15^ | pubmed found systematic review |
| 360 | Antibiotics and Topical Nasal Steroid for Treatment of Acute Maxillary Sinusitis A Randomized Controlled Trial  Williamson et al. | Public Health/ Preventive Medicine | 12/05/2007 JAMA | Acute sinusitis is generally treated with antibiotics, even though the condition tends to resolve without treatment, and whether the sinusitis is caused by bacteria is often unknown.^16-18^ Steroids are occasionally prescribed as alternative to antibiotics,^19^ although there is a lack of evidence for their effectiveness in relieving sinusitis symptoms. This paper found that antibiotics (n=54), nasal steroids (n=56), and a combination of both (n=46) were no better than placebo (n=51) in clinically curing and lessening duration and severity of acute sinusitis in the primary care setting. Twenty-nine percent of patients in the amoxicillin (antibiotic) group experienced symptoms lasting 10 or more days compared to 33.6% in the no-antibiotic group (adjusted OR, 0.99; 95% CI, 0.57-1.73). The percent of patients experiencing symptoms lasting 10+ days in the topical budesonide (steroid) group was 31.4% compared to 31.4% in the no steroid group (adjusted OR, 0.93; 95% CI, 0.54-1.62). This is a reversal of topical nasal steroid for treatment of acute maxillary sinusitis. | 2014. Cochrane. Systemic corticosteroids for acute sinusitis. “Oral corticosteroids as a monotherapy appear to be ineffective for adult patients with clinically diagnosed acute sinusitis.”^20^ | pubmed search of article title |
| 361 | Low-Fat Dietary Pattern and Risk of Colorectal Cancer The Women's Health Initiative Randomized Controlled Dietary Modification Trial  Beresford et al. | Public Health/ Preventive Medicine | 2/08/2006 | Low fat diets have been suggested since the 1970s to reduce the risk of cancer, and the recommendation for a low-fat diet for all was supported by the American Society of Clinical Nutritionists, the AHA, and the National Cancer Institute.^21^ However, observational studies on low-fat diets and rates of cancer have been inconsistent, some suggesting a positive association between fat intake and cancer, and others show no correlation.^22 23^ The Women's Health Initiative RCT determined that, compared to an unaltered diet (n=29294), a low fat, high vegetable diet (n=19541) did not significantly reduce the risk of colorectal cancer in postmenopausal women (HR, 1.08; 95% CI, 0.90-1.29). This is a reversal of low-fat diet for reducing risk of colorectal cancer in postmenopausal women. | None found | Google scholar search “low fat diet cancer systematic review” |
| 362 | Low-Fat Dietary Pattern and Risk of Cardiovascular Disease The Women's Health Initiative Randomized Controlled Dietary Modification Trial  Howard et al. | Public Health/ Preventive Medicine | 2/08/2006 JAMA | Low fat diets have been suggested since the 1950s to reduce the risk of cardiovascular disease, and the recommendation for a low-fat diet for heart health was supported by the American Heart Association.^21^ The Women's Health Initiative randomized controlled study determined that a low fat, high vegetable diet (n=19541) did not significantly reduce the risk of coronary heart disease (HR, 0.97; 95% CI, 0.90-1.06), stroke (HR, 1.02; 95% CI, 0.90-1.15), or cardiovascular disease (HR, 0.98; 95%, 0.92-1.05) in postmenopausal women compared to usual diet (n=29294). This is a reversal of low-fat diets for reducing risk of cardiovascular disease in postmenopausal women. | 2016. “In people with primary hypertension, weight loss diets reduced body weight and blood pressure, however the magnitude of the effects are uncertain due to the small number of participants and studies included in the analyses. Whether weight loss reduces mortality and morbidity is unknown. No useful information on adverse effects was reported in the relevant trials.”^24^ | pubmed found systematic review |
| 363 | Effect of Policosanol on Lipid Levels Among Patients With Hypercholesterolemia or Combined Hyperlipidemia A Randomized Controlled Trial  Berthold et al. | Cardiovascular | 5/17/2006 JAMA | Policosanol is a supplement derived from sugar cane that is advertised as a lipid-lowering substance.^25^ Policosanol was developed and approved for use in Cuba in 1991 and sold in many South American and Caribbean countries.^26^ Most of the research on policosanol comes from one research group in Cuba and the authors of this study aimed to study the drug outside of Cuba to confirm its efficacy. This study found that sugarcane-derived policosanol had no effect on LDL-C levels, regardless of dosage (n=114), compared to placebo (n=29) in patients with hypercholesterolemia or combined hyperlipidemia (P=.39). This is a reversal of Policosanol for treating hypercholesterolemia or combined hyperlipidemia. | 2009. “Data do not support an LDL-lowering claim for guggulipid, policosanol, or cinnamon.”^27^ | pubmed search of article title |
| 364 | Fluoxetine After Weight Restoration in Anorexia Nervosa A Randomized Controlled Trial  Walsh et al. | Psychiatry | 6/14/2006 JAMA | Anorexia Nervosa is a psychiatric illness with a high rate of relapse.^28^ To prevent relapse, various interventions have been implemented to support patients post-hospitalization. Antidepressants are thought to prevent relapse in patients with anorexia nervosa because these patients often exhibit psychiatric orders such as depression and obsessive compulsive disorder.^29^ Selective serotonin reuptake inhibitors (SSRIs) are commonly prescribed to patients both during and following hospitalization,^30 31^ although there is a lack of evidence to back these treatments. SSRIs may be ineffective at low weight, therefore the authors of this study investigated the effect of fluoxetine, an SSRI, on rehospitalization in patients who have completed a course of intensive treatment and have been restored to a healthy weight. The study found that fluoxetine (n=49) had no added benefit to patients with anorexia nervosa after weight restoration compared to placebo (n=44). There was no significant between groups in time-to-relapse (HR, 1.12; 95% CI, 0.65-2.01; P=.64). This is a reversal of fluoxetine for preventing relapse in patients with anorexia nervosa. | 2014. “We found that anti-depressants and antipsychotics had no significant effect on weight compared to placebo in the treatment of AN [anorexia nervosa].” ^32^ | pubmed found systematic review |
| 365 | Manual Chest Compression vs Use of an Automated Chest Compression Device During Resuscitation Following Out-of-Hospital Cardiac Arrest: A Randomized Trial  Hallstrom et al. | Cardiovascular | 6/14/2006 JAMA | Out-of-hospital cardiac arrest is generally treated by cardiopulmonary resuscitation (CPR) and the quality and order of resuscitation intervention may have an effect on cardiac and neurological outcomes.^33^ Consistent compressions in CPR is difficult while maintaining quality, and paramedics have been shown to provide shallower, slower compressions over time.^34^ Manual chest compression devices were designed to provide ideal chest compressions. The AutoPulse Resuscitation System is a load-distributing band circumferential chest compression device (LDB-CPR) that received marketing clearance by the FDA in 2002.^35^ This study compared the use of an LDB-CPR device with manual CPR in EMS care for patients with cardiac arrest that was presumed to be of cardiac origin and that had occurred prior to the arrival of EMS personnel. Automated LDB-CPR devices (n=394) were associated with worse neurological outcomes and showed a trend toward worse survival compared to manual CPR (n=373). Comparing LDB-CPR to manual CPR, survival to hospital discharge was 5.8% vs 9.9% (P=.06). The 2 best cerebral performance categories at hospital discharge were recorded in 3.1% of LDB-CPR patients compared to 7.5% of manual CPR patients (P=.006). This is a reversal on the use of automated chest compression devices for resuscitation following out-of-hospital cardiac arrest. | 2014. Cochrane review “Evidence from RCTs in humans is insufficient to conclude that mechanical chest compressions during cardiopulmonary resuscitation for cardiac arrest are associated with benefit or harm. Widespread use of mechanical devices for chest compressions during cardiac events is not supported by this review. More RCTs that measure and account for the CPR process in both arms are needed to clarify the potential benefit to be derived from this intervention.”^36^ | pubmed found systematic review |
| 366 | Cognitive Behavioral Therapy vs Zopiclone for Treatment of Chronic Primary Insomnia in Older Adults A Randomized Controlled Trial  Siversten et al. | Public Health/ Preventive Medicine | 6/28/2006 JAMA | Insomnia is a common complaint among individuals age 55 years and older and is associated with reduced quality of life, affective disorders, and increased health service utilization.^37^ Pharmacological interventions are common treatments prescribed by primary care physicians, yet sleep medication has shown to have a small effect size and clinical benefit, and long-term use of the drugs can cause dependency and increased tolerance.^38^ Zopiclone, a non-benzodiazepine sleeping pill that has been on the market since 1986,^39^ is also associated with next-day sleepiness and traffic collisions.^40 41^ Cognitive behavioral therapy (CBT) is the most widely used psychological intervention for insomnia but has limited studies proving its efficacy. This study was the first RCT to compare the effects of nonbenzodiazepine sleep medications with nonpharmacological treatment. The study found that, at 6 months, CBT improved sleep efficiency from 81.4% to 90.1% compared to the zopiclone group, which saw a decrease in efficiency from 82.3% to 81.9%.  CBT (n=18) improved short and long-term sleep outcomes compared to zopiclone and that in most outcomes, zopiclone (n=16) was no more effective than placebo (n=12). Zopiclone was no better than placebo in improving symptoms for patients with insomnia. This is a reversal of zopiclone for improving insomnia symptoms. | 2012. “There is moderate grade evidence suggesting CBT-I is superior to the non-benzodiazepines zopiclone and zolpidem for improving sleep measures in the short term.”^42^ | pubmed found systematic review |
| 367 | Distal Microcirculatory Protection During Percutaneous Coronary Intervention in Acute ST-Segment Elevation Myocardial Infarction A Randomized Controlled Trial  Stone et al. | Cardiology | 3/02/2005 JAMA | Distant microcirculatory protection devices were designed to prevent distal embolization of atheromatous and thrombotic debris after primary percutaneous coronary intervention (PCI) and enhance event-free survival in patients.^43^ Two devices were FDA approved at the time of this trial: the PercuSurge GuardWire (Medtronic Corp, Santa Rosa, Calif) and FilterWire-Ex (Boston Scientific, Santa Clara, Calif)^44^ and their popularity was growing in the US since their approval.^45^ This study found that distal embolic protection did not improve microvascular flow, greater reperfusion success, reduced infarct size, or enhanced event-free survival in patients undergoing primary PCI. The absolute difference in ST-segment resolution 30 minutes after PCI between patients assigned to distal protection (n=252) and patients without distal protection (n=249) was 1.4% (95% CI, -7.7%-10.5%; P=.78). Infract size was 12.0% with distal protection and 9.5% without (p=.15). This is a reversal of distal microcirculatory protection during PCI. | 2011. “In patients with STEMI, for most devices, few RCTs evaluated final health outcomes over a long period of follow-up. Due to insufficient data, the safety of these devices is unclear.”^46^ | pubmed found systematic review |
| 368 | Effects of Long-term Vitamin E Supplementation on Cardiovascular Events and Cancer A Randomized Controlled Trial  HOPE and HOPE-TOO investigators | Public Health/ Preventive Medicine | 3/16/2005 JAMA | Vitamin E supplements have been associated with good cardiovascular health and cancer prevention due to their antioxidant properties.^47^ Vitamin E supplementation has been recommended since the 1940s for treatment of various cardiovascular disorders.^48^ This article sought to evaluate long-term Vitamin E supplementation effects on risk of cancer, cancer death, and major cardiovascular events. They found that there were no preventive effects for all outcomes compared to placebo. The Vitamin E group (n=4761) had 11.6% cancer incidence versus 12.3% in the placebo (n=4780) group (RR, 0.94; 95% CI, 0.84-1.06; P=.30), 3.3% vs 3.7% for cancer deaths (RR, 0.88; 95% CI, 0.71-1.09; P=.24), and 21.5% vs 20.6% for cardiovascular events (RR, 1.04; 95% CI, 0.96-1.14; p=.34). The vitamin E group also had higher risk of heart failure (P=.03) and hospitalization for heart failure (P=.045). This is a reversal of long-term vitamin E supplementation for preventing cardiovascular events and cancer. | 2012. US Preventive Services Task Force. “Trials of vitamin E supplementation showed mixed results and altogether had no overall effect on cancer, CVD, or all-cause mortality.”^49^ | pubmed found systematic review |
| 369 | Information Leaflet and Antibiotic Prescribing Strategies for Acute Lower Respiratory Tract Infection A Randomized Controlled Trial  Little et al. | Pulmonary disease | 06/22/2005 + 06/29/2005 JAMA | Acute lower respiratory tract infections have been commonly treated with antibiotics even though there are conflicting findings on its efficacy. Although there has been a consensus to limit antibiotic use in this population,^50^ a 1993-1994 survey of first-line antibiotic prescription for lower respiratory tract infection conducted in France, Germany, Italy, Spain, and the UK found that 83% of general practitioners treated infections with antibiotics.^51^ This study found that immediate antibiotics had no greater effect on symptom duration or severity compared to delaying treatment in patients with acute uncomplicated lower respiratory tract infection. Participants not offered antibiotics (n=273) did not show a difference in cough duration compared to the delayed antibiotic group (n=272; 0.75 days; 95% CI, -0.37-1.88) or the immediate antibiotic group (n=262; 0.11 days; 95% CI, -1.01-1.24). This is a reversal for prescribing antibiotics for acute lower respiratory tract infection. | 2017. Cochrane review. “The strategy of no antibiotics further reduced antibiotic use compared to delaying prescription for antibiotics (14% versus 28%). Delayed antibiotics for people with acute respiratory infection reduced antibiotic use compared to immediate antibiotics, but was not shown to be different to no antibiotics in terms of symptom control and disease complications. Where clinicians feel it is safe not to prescribe antibiotics immediately for people with respiratory infections, no antibiotics with advice to return if symptoms do not resolve is likely to result in the least antibiotic use while maintaining similar patient satisfaction and clinical outcomes to delaying prescription of antibiotics.”^52^ | pubmed found systematic review |
| 370 | Vitamin E in the Primary Prevention of Cardiovascular Disease and Cancer The Women’s Health Study: A Randomized Controlled Trial  Lee et al. | Public Health/ Preventive Medicine | 7/06/2005 JAMA | Vitamin E supplements have been associated with good cardiovascular health and cancer prevention due to their antioxidant properties.^47^ Vitamin E supplementation has been recommended since the 1940s for treatment of various cardiovascular disorders.^48^ This article sought to evaluate long-term Vitamin E supplementation effects on risk of major cardiovascular events and invasive cancer. They found that participants that ingested 600 IU of natural-source vitamin E (n= 19937) experienced no added benefit to all outcomes compared to the placebo group (n=19939). The vitamin E group experience 482 major cardiovascular events and the placebo group experienced 517 (RR, 0.93; 95% CI, 0.82-1.05; P=.26). For incidences of total cancer, there were 1437 cases in the vitamin E group and 1428 in the placebo group (RR, 1.01; 95% CI, 0.94-1.08; P=.87). This is a reversal of vitamin E for preventing cardiovascular disease and cancer in women. | 2012. US Preventive Services Task Force. “Trials of vitamin E supplementation showed mixed results and altogether had no overall effect on cancer, CVD, or all-cause mortality.”^49^ | pubmed found systematic review |
| 371 | Anesthesia-Assisted vs Buprenorphine- or Clonidine-Assisted Heroin Detoxification and Naltrexone Induction A Randomized Trial  Collins et al. | Public Health/ Preventive Medicine | 08/24/2005+ 08/24/2005 JAMA | An effective, safe opioid detoxification has not been found and medically supervised heroin withdrawal has high patient discomfort and dropout rates.^53^ Anesthesia-assisted heroin opioid detoxification has therefore been used to offer a painless treatment.^54^ This study compared the effects of anesthesia-assisted rapid opioid detoxification with 2 control procedures: buprenorphine-assisted (positive control) and clonidine-assisted (negative control) opioid detoxification with delayed naltrexone induction. The trial found that anesthesia (n=35) was no more effective than buprenorphine (n=37) in treating withdrawal severities in opioid addiction with naltrexone. Rates of naltrexone induction (94% anesthesia vs 97% buprenorphine), treatment retention (20% vs 24%), and proportion of opioid-positive urine specimens (14% vs 14%) did not differ between groups. Additionally, anesthesia is more expensive and can cause life-threatening adverse events. This is a reversal of adding anesthesia to heroin detoxification treatment. | 2010. Cochrane review. “Heavy sedation compared to light sedation does not confer additional benefits in terms of less severe withdrawal or increased rates of commencement on naltrexone maintenance treatment. Given that the adverse events are potentially life-threatening, the value of antagonist-induced withdrawal under heavy sedation or anaesthesia is not supported. The high cost of anaesthesia-based approaches, both in monetary terms and use of scarce intensive care resources, suggest that this form of treatment should not be pursued.”^55^ | pubmed found systematic review |
| 372 | Evaluation Study of Congestive Heart Failure and Pulmonary Artery Catheterization Effectiveness The ESCAPE Trial  ESCAPE investigators | Cardiovascular | 10/05/2005 JAMA | Pulmonary artery catheterization (PAC) was introduced in the 1970s and was adopted nationwide in the ICU and perioperative settings for congestive heart failure.^56^ Although therapies have improved over the years, patients with heart failure still have up to 35-40% one-year mortality rates.^57^ PAC has been questioned for its safety and efficacy. This study investigated the survival rate of patients after PAC (n=206) or clinical assessment alone (n=207). They found that PAC increased adverse events (21.9% PAC vs 11.5% clinical assessment; P=.04) and had no effect on days alive out of the hospital during the first 6 months (133 vs 135 days; HR, 1.00; 95% CI, 0.82-1.21; P=.99), overall mortality (10% vs 9%; OR, 1.26; 95% CI, 0.78-2.03; P=.35), and number of days hospitalized (8.7 vs 8.3; HR, 1.04; 95% CI, 0.86-1.27; P=.67). This is a reversal of PAC for patients with congestive heart failure. | 2013. Cochrane review. “PAC is a diagnostic and haemodynamic monitoring tool but not a therapeutic intervention. Our review concluded that use of a PAC did not alter the mortality, general ICU or hospital LOS, or cost for adult patients in intensive care.”^58^ | pubmed found systematic review |
| 373 | Electrocardiographic and Hemodynamic Effects of a Multicomponent Dietary Supplement Containing Ephedra and Caffeine A Randomized Controlled Trial  McBride et al. | Endocrinology/Diabetes/Metabolism | 1/14/2004 JAMA | Metabolife 356 was a popular dietary supplement containing ephedra and caffeine (DSEC). Ephedra and caffeine are popular “natural” diet pill ingredients, known to aid in weight loss.^59^ This study found that in healthy volunteers (n=15), compared to placebo, DSEC significantly increased mean maximal QTc interval (mean (SD), 419.4 (11.8) vs 396.1 milliseconds; P<.001) and systolic blood pressure (mean (SD), 123.5 (10.98) vs 118.93 (9.62) mm Hg; P = .009), and may increase risk of ventricular and atrial arrhythmias. (Later banned by the FDA in 2004 for serious adverse events.) This is a reversal of Metabolife 356 for weight loss due to serious adverse effects caused by the supplement. | None found | google "metabolife 356" |
| 374 | Prehospital Hypertonic Saline Resuscitation of Patients With Hypotension and Severe Traumatic Brain Injury A Randomized Controlled Trial  Cooper et al. | Neurology | 3/17/2004 JAMA | Severe traumatic brain injury has a high mortality rate and many survivors suffer from severe neurological disability. Intravenous hypertonic saline (HTS) has shown to increase blood pressure and decrease elevated intracranial pressure compared to isotonic resuscitation fluids.^60^ HTS-colloid solutions were approved in 11 European countries and was recommended in the Brain Trauma Foundation’s "Guidelines for Pre-hospital Management of Traumatic Brain Injury", with or without dextran as an option for treatment.^61 62^ This study found, compared to patients who received conventional fluid (n=113), patients with hypotension and severe traumatic brain injury who received prehospital resuscitation with HTS (n=113) had no better improvement in neurological outcomes (RR, 0.99; 95% CI, 0.76-1.30; P=.96). This is a reversal of prehospital HTS solutions for treating patients with hypotension and severe traumatic brain injury. | 2016. "We observed no mortality benefit or effect on the control of intracranial pressure with the use of hypertonic saline when compared to other solutions. Based on the current level of evidence pertaining to mortality or control of intracranial pressure, hypertonic saline could thus not be recommended as a first-line agent for managing patients with severe traumatic brain injury."^63^ | google scholar search within articles citing paper. "review" |
| 375 | Low-Dose Inhaled Nitric Oxide in Patients With Acute Lung Injury A Randomized Controlled Trial  Taylor et al. | Pulmonary | 4/07/2004 JAMA | Inhaled nitric oxide is a selective pulmonary vasodilator and has been shown to improve gas exchange in acute respiratory syndrome.^64 65^ A 1997 survey of members of the European Society of Intensive Care Medicine found that 63.2% of respondents prescribed inhaled NO to treat acute lung injury and acute respiratory distress syndrome.^66^ This multicenter, randomized, placebo-controlled, blinded study found that, compares to placebo (n=193), inhaled NO (n=192) in patients with non-sepsis acute lung injury and without evidence of nonpulmonary organ system dysfunction did not lead to a substantial impact on duration of ventilatory support (10.6 days in placebo vs 10.7 days in inhaled NO; P=.97) or mortality (20% vs 23%; P=.54). This is a reversal of low-dose inhaled NO to treat patients with acute lung injury. | 2016. Cochrane review. "Evidence is insufficient to support INO in any category of critically ill patients with AHRF. Inhaled nitric oxide results in a transient improvement in oxygenation but does not reduce mortality and may be harmful, as it seems to increase renal impairment."^67^ | pubmed found systematic review |
| 376 | Effects of Conjugated Equine Estrogen in Postmenopausal Women With Hysterectomy The Women's Health Initiative Randomized Controlled Trial  WHI committee | Obstetrics/Gynecology | 4/14/2004 JAMA | Estrogen therapy has been offered to postmenopausal women with hysterectomies in order to lower risk of breast cancer and all-cause mortality and to treat menopause symptoms.^68^ It was in common practice to prescribe long-term estrogen to postmenopausal women as a preventive treatment since the mid-1900s.^69^ In this randomized, double-blind, placebo-controlled disease prevention trial, it was found that, compared to placebo (n=5429), the conjugated equine estrogen (n=5310) did not affect coronary heart disease incidence (HR, 0.91; 95% CI, 0.75-1.12), increased risk of stroke, and decreased risk of hip fracture in postmenopausal women with prior hysterectomy. The authors concluded that conjugated equine estrogen “should not be recommended for chronic disease prevention in postmenopausal women.”^70^ This is a reversal of conjugated equine estrogens for preventing coronary heart disease, stroke, and hip fractures in postmenopausal women with hysterectomies. | 2017. Cochrane Review. "Women with intolerable menopausal symptoms may wish to weigh the benefits of symptom relief against the small absolute risk of harm arising from short-term use of low-dose HT, provided they do not have specific contraindications. HT may be unsuitable for some women, including those at increased risk of cardiovascular disease, increased risk of thromboembolic disease (such as those with obesity or a history of venous thrombosis) or increased risk of some types of cancer (such as breast cancer, in women with a uterus).”^71^ | pubmed found systematic review |
| 377 | Conjugated Equine Estrogens and Incidence of Probable Dementia and Mild Cognitive Impairment in Postmenopausal Women Women's Health Initiative Memory Study  Shumaker et al. | Obstetrics/Gynecology | 06/23/2004+ 06/30/2004 JAMA | The Women's Health Initiative studied both estrogen plus progestin therapy and estrogen-alone therapy for dementia and cognitive health in postmenopausal women. Cognitive decline in older women is thought to be due to loss of estrogen^72^ and observational and smaller experimental studies have found promising neuroprotective effects with estrogen treatment in postmenopausal women. Postmenopausal women at the time of this study were regularly recommended hormone therapy,^73^ with as many as one out of every four postmenopausal women taking this therapy during the early 1990s.^74^ This study focused on estrogen-alone therapy (n=1464) for dementia and mild cognitive impairment compared to control (n=1483) and found that estrogen therapy alone did not reduce dementia or MCI incidence and increased the risk for both end points combined (HR, 1.38; 95% CI, 1.01-1.89; P=.04). This is a reversal of conjugated equine estrogens for prevention of dementia and mild cognitive impairment in postmenopausal women. | 2017, Cochrane Review, Long-term hormone therapy for perimenopausal and postmenopausal women: "HT is not indicated for primary or secondary prevention of cardiovascular disease or dementia, nor for prevention of deterioration of cognitive function in postmenopausal women."^71^ | pubmed found systematic review |
| 378 | Conjugated Equine Estrogens and Global Cognitive Function in Postmenopausal Women Women's Health Initiative Memory Study  Espeland et al. | Obstetrics/Gynecology | 06/23 + 06/30/2004 JAMA | The Women's Health Initiative studied both estrogen plus progestin therapy and estrogen-alone therapy for cognitive health in postmenopausal women. Cognitive decline in older women is thought to be due to loss of estrogen^72^ and observational and smaller experimental studies have found promising neuroprotective effects with estrogen treatment in postmenopausal women. Postmenopausal women at the time of this study were regularly recommended hormone therapy,^73^ with as many as one out of every four women taking this therapy during the early 1990s.^74^ This study focused on estrogen-alone therapy for cognitive function and found that it had greater adverse effects on women compared to placebo, especially in women with lower cognitive function at initiation of treatment. At mean follow-up of 5.4 years, compared to women taking placebo (n=1421), women taking hormone replacement therapy (n=1387) scores were 0.26 units lower on the Modified Mini-Mental State Examination (P=.04) and the mean decrease was 0.21 (SE, 0.08; P=.006). This is a reversal of conjugated equine estrogens for cognitive health in postmenopausal women. | 2017. Cochrane Review. "HT is not indicated for primary or secondary prevention of cardiovascular disease or dementia, nor for prevention of deterioration of cognitive function in postmenopausal women."^71^ | pubmed found systematic review |
| 379 | Effects of Systematic Prone Positioning in Hypoxemic Acute Respiratory Failure A Randomized Controlled Trial  Guerin et al. | Critical care | 11/17/2004 JAMA | In 2001, the Intensive and Critical Care Nursing Journal released guidelines regarding prone positioning of patients with acute respiratory distress syndrome (ARDS).^75^ The recommendation was that patients with ARDS should be placed in the prone position to improve survival. A clinical follow-up study found that patients with severe acute lung insufficiency who were treated in the prone position had improved gas exchange.^76^ This study investigated the effects on mortality of prone positioning on patients with hypoxemic ARDS and found that prone position (=413) had no beneficial outcomes and may have some safety concerns compared to supine placement (n=378). The 28-day mortality rate for the prone and supine groups were 32.4% and 31.5%, respectively (p=.77), and pressure sores, selective intubation, and endotracheal tube obstruction incidences were higher in the prone group. This is a reversal of the universal application of prone positioning for hypoxemic acute respiratory failure. | 2015. Cochrane Review. "We found no convincing evidence of benefit nor harm from universal application of PP [prone positioning] in adults with hypoxaemia mechanically ventilated in intensive care units (ICUs). Three subgroups (early implementation of PP, prolonged adoption of PP and severe hypoxaemia at study entry) suggested that prone positioning may confer a statistically significant mortality advantage. "^77^ | pubmed found systematic review |
| 380 | Prevention of Hip Fractures by External Hip Protectors A Randomized Controlled Trial  Schoor et al. | Orthopedic | 4/16/2003 JAMA | Hip fractures affect millions of people annually. External hip protectors were designed to absorb the impact of a fall to prevent fractures. There were a number of RCTs investigating external hip protectors and hip fracture prevention showing mixed results,^78-80^ yet protectors were still regularly prescribed in practices.^81^ This article found that prescribing a hip protector was not effective in preventing hip fractures in elderly persons aged 70 years and older compared to risk and bone health information. There were 18 fractures in the intervention group (n= 276) compared to 20 fractures in the control group (n=285; P=.86). This is a reversal of external hip protectors for preventing fractures in elderly persons in institutional homes. | 2014. Cochrane review. After excluding studies with high risk of bias, this Cochrane systematic review found that hip protectors did not have a significant effect on risk of hip fractures in institutional settings. ^15^ | pubmed found systematic review |
| 381 | Pacemaker Therapy for Prevention of Syncope in Patients With Recurrent Severe Vasovagal Syncope Second Vasovagal Pacemaker Study (VPS II): A Randomized Trial  Connolly et al. | Cardiology | 5/07/2003 JAMA | Pacemaker therapy for vasovagal syncope came about on the theory that the devices may ameliorate symptoms or delay development on syncope during tilt testing. Smaller randomized trials found pacemakers to be beneficial for recurrent vasovagal syncope.^82 83^ Pacemaker therapy for fainting has been in practice since the 1980s as seen in case reports and case series.^84^ This study found that there was no significant difference in risk of recurrent syncope at 6 months between patients assigned to pacemakers with (n=48) or without (n=52) pacing (31% vs 43%; RR 30%; 95% CI, -33%-63%; 1-sided P=.14). The editor suggests that treating syncope with pacemakers should be avoided without further research as it is a permanent, invasive treatment with little physiological proof or theory. This is a reversal of pacemaker therapy for preventing vasovagal syncope. | 2011. "There is insufficient evidence to support the use of any of the pharmacological or pacemaker treatments for vasovagal syncope and carotid sinus syncope. Larger studies using patient relevant outcomes are needed."^85^ | pubmed found systematic review |
| 382 | Estrogen Plus Progestin and the Incidence of Dementia and Mild Cognitive Impairment in Postmenopausal Women The Women's Health Initiative Memory Study: A Randomized Controlled Trial  Shumaker et al. | Obstetrics/Gynecology | 5/28/2003 JAMA | Estrogen plus progestin was a common treatment for menopausal women to prevent coronary heart disease and osteoporosis. Hormone therapies were once only prescribed for short periods to reduce menstrual symptoms, but had become widely prescribed for long-term, indefinite use.^86^ Postmenopausal women have a higher risk of developing Alzheimer disease than men and case control and prospective studies have indicated that estrogen therapy is associated with lower risk of dementia.^87^ Studies on hormone therapy and cognitive function have shown trends of postmenopausal women using this treatment regularly.^88^ The Women's Health Initiative conducted a large RCT to analyze the effects of hormone therapy on dementia and cognitive function and found that, compared to placebo (n=2303), hormone therapy (n=2229) increased risk of dementia in women aged 65 and older (HR, 2.05; 95% CI, 1.21-3.48; 22 vs 45 cases of dementia per 10000 person-years; P=.01). This is a reversal of estrogen plus progestin for dementia and cognitive impairment prevention in postmenopausal women. | 2008. Cochrane review. "There is good evidence that both ERT and HRT do not prevent cognitive decline in older postmenopausal women when given as short term or longer term (up to five years) therapy...In the meantime, based on the available evidence, ERT or HRT cannot be recommended for overall cognitive improvement or maintenance in older postmenopausal women without cognitive impairment.”^89^ | pubmed found systematic review |
| 383 | Effect of Estrogen Plus Progestin on Global Cognitive Function in Postmenopausal Women The Women's Health Initiative Memory Study: A Randomized Controlled Trial  Rapp et al. | Obstetrics/Gynecology | 5/28/2003 JAMA | Estrogen plus progestin was a common treatment for menopausal women to prevent coronary heart disease and osteoporosis. Hormone therapies were once only prescribed for short periods to reduce menstrual symptoms, but had become widely prescribed for long-term, indefinite use.^86^ Postmenopausal women have a higher risk of developing Alzheimer disease than men and case-control and prospective studies have indicated that estrogen therapy is associated with better cognitive function.^90^ Studies on hormone therapy and cognitive function have shown trends of postmenopausal women using this treatment regularly.^88^ The Women's Health Initiative conducted a large RCT to analyze the effects of hormone therapy on cognitive function and found that, compared to placebo use (n=2236), hormone therapy use (n=2145) exhibited no clinically significant improvement. Furthermore, a small increased risk of clinically meaningful cognitive decline occurred in the estrogen plus progestin group (6.7% hormone therapy vs 4.8% placebo; P=.008). This is a reversal of estrogen plus progestin for improved cognitive function in postmenopausal women. | 2008. Cochrane review. "There is good evidence that both ERT and HRT do not prevent cognitive decline in older postmenopausal women when given as short term or longer term (up to five years) therapy...In the meantime, based on the available evidence, ERT or HRT cannot be recommended for overall cognitive improvement or maintenance in older postmenopausal women without cognitive impairment.”^89^ | pubmed found systematic review |
| 384 | Rapid Magnetic Resonance Imaging vs Radiographs for Patients With Low Back Pain A Randomized Controlled Trial  Jarvik et al. | Orthopedic | 06/04/2003 JAMA | MRIs have been suggested in guidelines for patients with low back pain who are considering surgery or whom systemic disease is strongly suspected.^91 92^ Yet primary care providers commonly order MRIs for patients with chronic low back pain regardless of surgical referrals.^93^ A study on rates of MRIs and spine surgery found that higher rates of MRI are associated with higher rates of spine surgery.^94^ This study aimed to assess outcomes and cost-effectiveness of MRIs for low back pain and found that participants with low back pain who received MRIs (n=170) experienced similar outcomes compared to those who received radiographs (n=167). Mean back-related disability modified Roland score was 8.75 in the radiograph group vs 9.34 in the MRI group (mean difference, -0.59; 95% CI, -1.69-0.87). Furthermore, MRIs may lead to increased number of spine operations and therefore cost more than radiography. This is a reversal of MRIs for patients with low back pain. | 2009. "Lumbar imaging for low-back pain without indications of serious underlying conditions does not improve clinical outcomes. Therefore, clinicians should refrain from routine, immediate lumbar imaging in patients with acute or subacute low-back pain and without features suggesting a serious underlying condition." ^95^ There were no systematic reviews comparing effects of MRI vs radiography in this setting. | google scholar search "mri low back pain systematic review" |
| 385 | Effect of Estrogen Plus Progestin on Stroke in Postmenopausal Women The Women's Health Initiative: A Randomized Trial  Smoller et al. | Obstetrics/Gynecology | 5/28/2003 JAMA | Hormone therapy has been frequently administered to postmenopausal women to prevent chronic disease and postmenopausal symptoms.^69^ Hormone therapies were once only prescribed for short periods to reduce menstrual symptoms, but had become widely prescribed for long-term, indefinite use in all postmenopausal women.^86^ The Women's Health Initiative Trial investigated the effects of estrogen plus progestin on multiple health outcomes in postmenopausal women and found that the therapy was more harmful than helpful. This study determined that, compared to placebo (n=8102), estrogen plus progestin (n=8506) increased risk of strokes in generally health postmenopausal women (HR 1.31; CI 1.09-1.90). This is a reversal of estrogen plus progestin for stroke prevention in postmenopausal women. | Cochrane, 2017. “HT is not indicated for primary or secondary prevention of cardiovascular disease or dementia, nor for prevention of deterioration of cognitive function in postmenopausal women.”^96^ | pubmed found systematic review |
| 386 | Influence of Estrogen Plus Progestin on Breast Cancer and Mammography in Healthy Postmenopausal Women The Women's Health Initiative Randomized Trial  Chlebowski et al. | Obstetrics/Gynecology | 6/25/2003 JAMA | Hormone therapy has been frequently administered to postmenopausal women to prevent chronic disease and postmenopausal symptoms.^69^ These therapies were once only prescribed for short periods to reduce menstrual symptoms, but had become widely prescribed for long-term, indefinite use in all postmenopausal women.^86^ The Women's Health Initiative Trial investigated the effects of estrogen plus progestin on multiple health outcomes in postmenopausal women and found that the therapy was more harmful than helpful. This study determined that estrogen plus progestin (n=8506) increased total (HR 1.24; weighted p<.001) and invasive (HR 1.24; weighted p=.003) breast cancer risk compared to placebo (n=8102) and increased incidence of abnormal mammograms (p<.001). This is a reversal of estrogen plus progestin for breast cancer prevention in postmenopausal women. | 2017. “Women with intolerable menopausal symptoms may wish to weigh the benefits of symptom relief against the small absolute risk of harm arising from short-term use of low-dose HT, provided they do not have specific contraindications. HT may be unsuitable for some women, including those at increased risk of cardiovascular disease, increased risk of thromboembolic disease (such as those with obesity or a history of venous thrombosis) or increased risk of some types of cancer (such as breast cancer, in women with a uterus).”^71^ | pubmed found systematic review |
| 387 | Effect of Behavioral Training With or Without Pelvic Floor Electrical Stimulation on Stress Incontinence in Women: A Randomized Controlled Trial  Goode et al. | Obstetrics/Gynecology | 7/16/2003 JAMA | Pelvic floor electrical stimulation (PFES) has been used to treat stress incontinence since 1952, when a study found that adding PFES to pelvic floor muscle exercise cured 7 of 17 women suffering from stress incontinence.^100 101^ Various PFES devices have been approved by the FDA^102^ and were approved for reimbursement by Medicare and other insurance companies.^103^ This study compared comprehensive behavioral therapy with (n=67) and without PFES (n=66) and found that the addition of PFES did not reduce number of incontinent episodes in women with stress incontinence (p=.60). They were both more effective than a self-help booklet (n=67). While pelvic floor therapy was shown to improve incontinence in women, the addition of electric stimulation was not beneficial. This is a reversal of the use of electric stimulation for stress incontinence in women. | 2017. Cochrane review. “The current evidence base indicated that electrical stimulation is probably more effective than no active or sham treatment, but it is not possible to say whether ES is similar to [pelvic floor muscle training] or other active treatments in effectiveness or not. Overall, the quality of the evidence was too low to provide reliable results. Without sufficiently powered trials measuring clinically important outcomes, such as subjective assessment of urinary incontinence, we cannot draw robust conclusions about the overall effectiveness or cost-effectiveness of electrical stimulation for stress urinary incontinence in women.”^104^ | pubmed found systematic review |
| 388 | Incidence of Cancer and Mortality Following α-Tocopherol and β-Carotene Supplementation A Postintervention Follow-up  The ATBC Study Group | Public Health/ Preventive Medicine | 7/23/2003+ 07/30/2003 JAMA | Vitamin supplements such as Vitamin E and A are commonly consumed in the United States,^105^ and there has been epidemiological data that shows an association between low serum concentration of antioxidants and risk of cancer.^106^ After a number of RCTs showed a reduction in cancer rates with vitamin supplementation, the Alpha-Tocopherol, Beta-Carotene Cancer Prevention (ATBC) study was designed to investigate the effects of the two supplements on incidence of lung and other cancers in male smokers aged 50 to 69 years.^107^ They found that beta-carotene increased risk of lung cancer and alpha-tocopherol reduced incidence of prostate cancer. This follow-up to the ATBC study found that the beneficial and adverse effects of alpha-tocopherol and beta-carotene disappeared after 6-8 years post intervention. There were a total of 29133 male smokers included in the analysis. RR for lung cancer was 1.06 (95% CI, 0.94-1.20) among the beta-carotene group compared to the no beta-carotene group. RR for prostate cancer incidence was 0.88 (95% CI, 0.76-1.03) for the alpha-tocopherol group compared no alpha-tocopherol. This is a reversal of vitamin E and A supplementation for cancer prevention. | 2012. Cochrane. Antioxidant supplements for prevention of mortality in healthy participants and patients with various diseases: “We found no evidence to support antioxidant supplements for primary or secondary prevention. Beta-carotene and vitamin E seem to increase mortality, and so may higher doses of vitamin A. Antioxidant supplements need to be considered as medicinal products and should undergo sufficient evaluation before marketing.”^108^ | google scholar search of "cancer mortality beta carotene alpha tocopherol supplements systematic review" |
| 389 | Guggulipid for the Treatment of Hypercholesterolemia A Randomized Controlled Trial  Szapary et al. | Public Health/ Preventive Medicine | 8/13/2003 JAMA | Guggul is a resin extract that has traditionally been used in Asia since 600 BC for obesity, atherosclerosis, and inflammatory conditions.^109^ "Recent research indicates that guggulsterones are antagonists of the farnesoid X receptor and the bile acid receptor, two nuclear hormone receptors involved in bile acid regulation and cholesterol metabolism."^110^ Traditional use and bio mechanistic theory have led to guggul's increased marketing and use in the US.^111^ This study found that guggulipid did not improve levels of serum cholesterol in a population with hypercholesterolemia, and may have raised levels of LDL-C. Levels of LDL-C increased by 4% in both the standard-dose (n=33) and high-dose guggulipid groups (n=34), while LDL-C were lowered in the placebo group by 5% (n=36). This is a reversal of guggulipid for the treatment of hypercholesterolemia. | 2005. "The effects of guggulipid in patients with high cholesterol are not clear, with some studies finding cholesterol-lowering effects, and other research suggesting no benefits. At this time, there is not enough scientific evidence to support the use of guggul for any medical condition."^112^ | pubmed search of article title |
| 390 | Effects of Estrogen Plus Progestin on Gynecologic Cancers and Associated Diagnostic Procedures The Women's Health Initiative Randomized Trial  Anderson et al. | Obstetrics/Gynecology | 10/01/2003  JAMA | Postmenopausal hormone replacement therapy (HRT) was initially used in the 1940s as a way to delay age-related health outcomes, but in the 1970’s studies began to emerge showing that the use of HRT, specifically unopposed estrogen, was associated with endometrial cancer. Progesterone was thought to oppose the effects of estrogen and mitigate the excess risk of cancer, and the combination hormone therapy popularized. By the 1990s, HRTs were the most commonly prescribed medications.^86^ HRTs were once only prescribed for short periods to reduce menstrual symptoms, but had become widely prescribed for long-term, indefinite use. The results of this study show that while the rates of endometrial cancer were no different in women taking estrogen plus progestin (n=8506) compared to placebo (8102), rates of ovarian cancer were higher. Women assigned to estrogen plus progestin had a hazard ratio of 1.58 for invasive ovarian cancer compared to the placebo group (95% CI, 0.77-3.24). This is a reversal of estrogen plus progestin for lowering incidence of gynecologic cancers in women. | 2017. Cochrane. “The apparent reduction in risk of endometrial cancer associated with combined HT is offset by the suggestion of increased risk of ovarian cancer.” ^113^ | Pubmed |
| 391 | Effect of Magnetic vs Sham-Magnetic Insoles on Plantar Heel Pain: A Randomized Controlled Trial  Winemiller et al. | Orthopedic | 9/17/2003 JAMA | Magnetic therapy has a history going back 4000 years. It has been and is still used to heal various pains. The first commercial cranial magnetic stimulator was developed in 1985 and adaptations continued to come onto the market.^114 115^ Various nonrandomized trials on magnets and pain have shown mixed results. Still, the practice continued and magnetic insoles were designed and used to treat plantar heel pain. Physical therapists commonly used magnetic therapy.^116^ This study determined that active bipolar magnetic insoles (n=57) offered no reduction to plantar heel pain when compared to nonmagnetic insoles (n=44). At 8 weeks, 35% of participants in the magnetic insole group reported being mostly or all better compared to 33% in the nonmagnetic group (p=0.78). This is a reversal of magnetic insoles for treatment of plantar heel pain. | 2007. "The evidence does not support the use of static magnets for pain relief, and therefore magnets cannot be recommended as an effective treatment."^117^ | pubmed found systematic review |
| 392 | Treatment of Corticosteroid-Responsive Autoimmune Inner Ear Disease With Methotrexate A Randomized Controlled Trial  Harris et al. | Rheumatology | 10/08/2003  JAMA | Methotrexate has been the gold standard of care for patients with rheumatoid arthritis and is often used to treat other autoimmune diseases.^118^ More specifically, methotrexate is sometimes used to treat patients with autoimmune inner ear disease as an adjunct therapy alongside corticosteroids.^119 120^ In this trial, methotrexate (n=33) was no better than placebo (n=34) in maintaining hearing improvement achieved with prednisone in patients with autoimmune inner ear disease (HR, 1.31; 95% CI, 0.79-2.17; P=.30). This is a reversal of methotrexate for maintaining hearing improvement in patients with autoimmune inner ear disease. | None found |  |
| 393 | Fenoldopam Mesylate for the Prevention of Contrast-Induced Nephropathy A Randomized Controlled Trial  Stone et al. | Nephrology | 11/05/2003 JAMA | Contrast-induced nephropathy is one of the most common causes of hospital-acquired acute renal failure.^121^ Fenoldopam mesylate is a dopamine agonist approved for hypertension that has been shown to increase renal plasma flow in patients with and without chronic renal insufficiency.^122^ Fenoldopam mesylate has been used as a renal protective agent and shown some efficacy.^123^ This study found that selective dopamine-1 agonist fenoldopam mesylate does not prevent further renal function deterioration after contrast administration compared to placebo in patients with chronic renal insufficiency. In the fenoldopam group (n=153), 33.6% of patients experienced contrast-induced nephropathy, compared to 30.1% in the placebo group (n=156). Rehospitalization, dialysis, and 30-day mortality rates were also similar between groups. This is a reversal of fenoldopam mesylate for preventing contrast-induced nephropathy in patients with chronic renal insufficiency. | 2015. “Fenoldopam is no better than Placebo/Saline or NAC [N-acetyl cysteine] in preventing CIN, but more studies are required.”^124^ | pubmed search of article title |
| 394 | Early Use of the Pulmonary Artery Catheter and Outcomes in Patients With Shock and Acute Respiratory Distress Syndrome A Randomized Controlled Trial  Richard et al. | Critical care | 11/26/2003 JAMA | Pulmonary artery catheter (PAC) was commonly used in the diagnosis and treatment for cardiopulmonary disturbances. It is thought to provide necessary information and temporal pacing,^125^ yet an observational study on catheterization in the ICU found that catheters were associated with increased mortality and greater utilization of resources.^126^ This study aimed to determine the effects of early use of a PAC in patients with shock mainly of septic origin, acute respiratory distress syndrome (ARDS), or both. The study found that was no difference in mortality or morbidity in patients who were (n=335) and were not (n=341) treated with PAC (mortality: 59.4% vs 61.0% (RR, 0.97; 95% CI, 0.86-1.10; P = .67). This is a reversal on the early use of PAC in patients with shock and acute respiratory distress syndrome. | 2013. Cochrane review. “Our review concluded that use of a PAC did not alter the mortality, general ICU or hospital LOS, or cost for adult patients in intensive care. The quality of evidence was high for mortality and LOS but low for cost analysis.”^58 127^ | pubmed found systematic review |
| 395 | Efficacy and Safety of Echinacea in Treating Upper Respiratory Tract Infections in Children A Randomized Controlled Trial  Taylor et al. | Pediatrics | 12/03/2003 JAMA | Upper respiratory tract infections (URIs) are a substantial health burden for children. There is little evidence for the efficacy of conventional medications in treating children younger than 12 years, and therefore, parents and clinicians turn to alternative therapies. Echinacea is a coneflower that was used as a medicinal plant by the Plains Indians and has become a popular herbal treatment known for its immune boosting properties.^128 129^. There is some research on Echinacea’s effect on adult URIs, but the studies are flawed and available evidence is incomplete.^130^ This study investigated Echinacea extract's effect on URI symptoms and duration on children ages 2 to 11 years over a 4-month period. The study found that, compared to placebo (n=207), Echinacea extract (n= 200) was not effective in treating URI symptoms in children and induced a rash in some children. There was no difference in duration (P=.89) or severity (P=.69) of URI symptoms. This is a reversal of Echinacea for treating URIs in children. | 2014. Cochrane review. “Echinacea products have not here been shown to provide benefits for treating colds, although, it is possible there is a weak benefit from some Echinacea products: the results of individual prophylaxis trials consistently show positive (if non-significant) trends, although potential effects are of questionable clinical relevance.”^131^ | pubmed found systematic review |
| 396 | Combined Levothyroxine Plus Liothyronine Compared With Levothyroxine Alone in Primary Hypothyroidism A Randomized Controlled Trial  Clyde et al. | Endocrinology, diabetes, and metabolism | 12/10/2003 JAMA | Hypothyroidism is a common endocrine disorder that occurs in up to 5% of the US population. It is treated by normalizing thyroid hormone levels in peripheral tissues with the use of replacement therapy. Levothyroxine is the most common replacement medication today, but liothyronine, a medication with a much shorter half-life, is available and sometimes used in combination with levothyroxine. In the 1970's, the combination of the two drugs was researched and practiced,^132^ although the combination was found to have a higher incidence of thyrotoxic symptoms.^133^ The use of liothyronine declined over the years^134^ but was still prescribed to a small percent of those with hypothyroidism. At the time of this study, reports of patients who still had symptoms after normalizing thyroid function with levothyroxine were concerning. This study aimed to investigate whether combined therapy would improve QOL, neurocognitive functioning, and certain psychological parameters. The study found that, compared to levothyroxine alone (n=22), combination therapy (n=22) had no beneficial changes in any of the mentioned outcome measures. The HRQL questionnaire scores improved in both groups, but these changes were statistically similar (P = .54). This is a reversal of treating hypothyroidism with levothyroxine plus liothyronine compared with levothyroxine alone. | Levothyroxine alone should remain the standard of care for treating hypothyroidism.^135 136^ | google scholar search "levothyroxine liothyronine hypothyroidism systematic review" |

1. Cleveland JC, Shroyer ALW, Chen AY, et al. Off-pump coronary artery bypass grafting decreases risk-adjusted mortality and morbidity. *The Annals of Thoracic Surgery* 2001;72(4):1282-89. doi: <https://doi.org/10.1016/S0003-4975(01)03006-5>

2. Hattler B, Messenger JC, Shroyer AL, et al. Off-Pump Coronary Artery Bypass Surgery Is Associated With Worse Arterial and Saphenous Vein Graft Patency and Less Effective Revascularization: Results From the Veterans Affairs Randomized On/Off Bypass (ROOBY) Trial

*Circulation* 2012;125(23):2827-35. doi: 10.1161/circulationaha.111.069260

3. Møller CH, Penninga L, Wetterslev J, et al. Off-pump versus on-pump coronary artery bypass grafting for ischaemic heart disease. *Cochrane Database of Systematic Reviews* 2012(3) doi: 10.1002/14651858.CD007224.pub2

4. Bayram M, De Luca L, Massie MB, et al. Reassessment of Dobutamine, Dopamine, and Milrinone in the Management of Acute Heart Failure Syndromes. *The American Journal of Cardiology* 2005;96(6, Supplement):47-58. doi: <https://doi.org/10.1016/j.amjcard.2005.07.021>

5. Hirani N. Tenax Therapeutics Is Tackling Critical Care With Levosimendan: Seeking Alpha; 2015 [Available from: <https://seekingalpha.com/article/3534026-tenax-therapeutics-tackling-critical-care-levosimendan> accessed February 2nd 2018.

6. Moiseyev VS, Põder P, Andrejevs N, et al. Safety and efficacy of a novel calcium sensitizer, levosimendan, in patients with left ventricular failure due to an acute myocardial infarction. A randomized, placebo-controlled, double-blind study (RUSSLAN). *European Heart Journal* 2002;23(18):1422-32. doi: 10.1053/euhj.2001.3158

7. Schumann J, Henrich EC, Strobl H, et al. Inotropic agents and vasodilator strategies for the treatment of cardiogenic shock or low cardiac output syndrome. *Cochrane Database of Systematic Reviews* 2018(1) doi: 10.1002/14651858.CD009669.pub3

8. Zhang S, Folsom AR, Sellers TA, et al. Better breast cancer survival for postmenopausal women who are less overweight and eat less fat. The Iowa women's health study. *Cancer* 1995;76(2):275-83. doi: 10.1002/1097-0142(19950715)76:2<275::AID-CNCR2820760218>3.0.CO;2-6

9. Patterson BH, Block G. Food choices and the cancer guidelines. *American Journal of Public Health* 1988;78(3):282-86.

10. Chlebowski RT, Blackburn GL, Thomson CA, et al. Dietary Fat Reduction and Breast Cancer Outcome: Interim Efficacy Results From the Women's Intervention Nutrition Study. *JNCI: Journal of the National Cancer Institute* 2006;98(24):1767-76. doi: 10.1093/jnci/djj494

11. Pierce JP. Diet and breast cancer prognosis: making sense of the WHEL and WINS trials. *Current opinion in obstetrics & gynecology* 2009;21(1):86.

12. Thapa PB, Brockman KG, Gideon P, et al. Injurious Falls in Nonambulatory Nursing Home Residents: A Comparative Study of Circumstances, Incidence, and Risk Factors. *Journal of the American Geriatrics Society* 1996;44(3):273-78. doi: 10.1111/j.1532-5415.1996.tb00913.x

13. Cameron ID. Hip protectors. *Prevent fractures but adherence is a problem* 2002;324(7334):375-76. doi: 10.1136/bmj.324.7334.375

14. Parker MJ, Gillespie LD, Gillespie WJ. Hip protectors for preventing hip fractures in the elderly. *The Cochrane Database of Systematic Reviews* 2003(3) doi: 10.1002/14651858.CD001255

15. Santesso N, Carrasco‐Labra A, Brignardello‐Petersen R. Hip protectors for preventing hip fractures in older people. *The Cochrane Library* 2014

16. Ashworth M, Charlton J, Ballard K, et al. Variations in antibiotic prescribing and consultation rates for acute respiratory infection in UK general practices 1995–2000. *Br J Gen Pract* 2005;55(517):603-08.

17. Low DE, Desrosiers M, James McSherry MB C, et al. A practical guide for the diagnosis and treatment of acute sinusitis. *Canadian Medical Association Journal* 1997;156(6):S1.

18. Goossens H, Ferech M, Vander Stichele R, et al. Outpatient antibiotic use in Europe and association with resistance: a cross-national database study. *The Lancet* 2005;365(9459):579-87.

19. Osguthorpe JD, Hadley JA. RHINOSINUSITIS: Current Concepts in Evaluation and Management. *Medical Clinics of North America* 1999;83(1):27-41. doi: <https://doi.org/10.1016/S0025-7125(05)70085-7>

20. Venekamp RP, Thompson MJ, Hayward G, et al. Systemic corticosteroids for acute sinusitis. *Cochrane Database of Systematic Reviews* 2014(3) doi: 10.1002/14651858.CD008115.pub3

21. La Berge AF. How the Ideology of Low Fat Conquered America. *Journal of the History of Medicine and Allied Sciences* 2008;63(2):139-77. doi: 10.1093/jhmas/jrn001

22. Howe GR, Hirohata T, Hislop TG, et al. Dietary Factors and Risk of Breast Cancer: Combined Analysis of 12 Case—Control Studies. *JNCI: Journal of the National Cancer Institute* 1990;82(7):561-69. doi: 10.1093/jnci/82.7.561

23. Hunter DJ, Spiegelman D, Adami H-O, et al. Cohort Studies of Fat Intake and the Risk of Breast Cancer — A Pooled Analysis. *New England Journal of Medicine* 1996;334(6):356-61. doi: 10.1056/nejm199602083340603

24. Semlitsch T, Jeitler K, Berghold A, et al. Long-term effects of weight-reducing diets in people with hypertension. *Cochrane Database of Systematic Reviews* 2016(3) doi: 10.1002/14651858.CD008274.pub3

25. Gouni-Berthold I, Berthold HK. Policosanol: Clinical pharmacology and therapeutic significance of a new lipid-lowering agent. *American Heart Journal* 2002;143(2):356-65. doi: <https://doi.org/10.1067/mhj.2002.119997>

26. Policosanol: Nature's Food Patch; 2011 [Available from: <http://www.naturesfoodpatch.com/ns/DisplayMonograph.asp?storeID=a5pjlp3me3s92n5800akhlbd34ws3rl1&DocID=policosanol> accessed January 29 2018.

27. McGowan MP, Proulx S. Nutritional supplements and serum lipids: does anything work? *Current atherosclerosis reports* 2009;11(6):470.

28. Pike KM. Long-term course of anorexia nervosa: Response, relapse, remission, and recovery. *Clinical Psychology Review* 1998;18(4):447-75. doi: <https://doi.org/10.1016/S0272-7358(98)00014-2>

29. Halmi KA, Eckert E, Marchi P, et al. Comorbidity of psychiatric diagnoses in anorexia nervosa. *Archives of General Psychiatry* 1991;48(8):712-18. doi: 10.1001/archpsyc.1991.01810320036006

30. Walsh B, Kaplan AS, Attia E, et al. Fluoxetine after weight restoration in anorexia nervosa: A randomized controlled trial. *JAMA* 2006;295(22):2605-12. doi: 10.1001/jama.295.22.2605

31. Strober M, Freeman R, DeAntonio M, et al. Does adjunctive fluoxetine influence the post-hospital course of restrictor-type anorexia nervosa? A 24-month prospective, longitudinal followup and comparison with historical controls. *Psychopharmacology bulletin* 1997;33(3):425.

32. de Vos J, Houtzager L, Katsaragaki G, et al. Meta analysis on the efficacy of pharmacotherapy versus placebo on anorexia nervosa. *Journal of Eating Disorders* 2014;2:27. doi: 10.1186/s40337-014-0027-x

33. Steen S, Liao Q, Pierre L, et al. The critical importance of minimal delay between chest compressions and subsequent defibrillation: a haemodynamic explanation. *Resuscitation* 2003;58(3):249-58. doi: <https://doi.org/10.1016/S0300-9572(03)00265-X>

34. Ochoa FJ, Ramalle-Gómara E, Lisa V, et al. The effect of rescuer fatigue on the quality of chest compressions. *Resuscitation* 1998;37(3):149-52. doi: <https://doi.org/10.1016/S0300-9572(98)00057-4>

35. Administration FaD. SUMMARY FOR AutoPulse® Resuscitation System Model 100. In: Services DoHH, ed., 2012.

36. Brooks SC, Hassan N, Bigham BL, et al. Mechanical versus manual chest compressions for cardiac arrest. *Cochrane Database of Systematic Reviews* 2014(2) doi: 10.1002/14651858.CD007260.pub3

37. Simon GE, VonKorff M. Prevalence, burden, and treatment of insomnia in primary care. *The American journal of psychiatry* 1997;154(10):1417.

38. Glass J, Lanctôt KL, Herrmann N, et al. Sedative hypnotics in older people with insomnia: meta-analysis of risks and benefits. *BMJ* 2005;331(7526):1169. doi: 10.1136/bmj.38623.768588.47

39. Zopiclone: Pharmapedia; 2009 [Available from: <http://pharmapedia.wikidot.com/zopiclone> accessed May 6, 2018 2018.

40. Allain H, Delahaye C, Le Coz F, et al. Postmarketing surveillance of zopiclone in insomnia: analysis of 20,513 cases. *Sleep* 1991;14(5):408-13.

41. Montplaisir J, Hawa R, Moller H, et al. Zopiclone and zaleplon vs benzodiazepines in the treatment of insomnia: Canadian consensus statement. *Human Psychopharmacology: Clinical and Experimental* 2003;18(1):29-38.

42. Mitchell MD, Gehrman P, Perlis M, et al. Comparative effectiveness of cognitive behavioral therapy for insomnia: a systematic review. *BMC Family Practice* 2012;13:40-40. doi: 10.1186/1471-2296-13-40

43. Topol EJ, Yadav JS. Recognition of the importance of embolization in atherosclerotic vascular disease. *Circulation* 2000;101(5):570-80.

44. Fasseas P, Orford JL, Denktas AE, et al. Distal protection devices during percutaneous coronary and carotid interventions. *Current Controlled Trials in Cardiovascular Medicine* 2001;2(6):286-91. doi: 10.1186/cvm-2-6-286

45. Medtronic, Inc. Washington DC: United States Securities and Exchange Commission, 2004.

46. Sobieraj DM, White CM, Kluger J, et al. Systematic review: comparative effectiveness of adjunctive devices in patients with ST-segment elevation myocardial infarction undergoing percutaneous coronary intervention of native vessels. *BMC Cardiovascular Disorders* 2011;11:74-74. doi: 10.1186/1471-2261-11-74

47. Traber MG, Sies H. Vitamin E in humans: demand and delivery. *Annual review of nutrition* 1996;16(1):321-47.

48. Olson RE. Vitamin E and its relation to heart disease. *Circulation* 1973;48(1):179-84.

49. Moyer VA. Vitamin, mineral, and multivitamin supplements for the primary prevention of cardiovascular disease and cancer: US Preventive Services Task Force recommendation statement. *Annals of internal medicine* 2014;160(8):558-64.

50. Hickner JM, Bartlett JG, Besser RE, et al. Principles of appropriate antibiotic use for acute rhinosinusitis in adults: background. *Annals of emergency medicine* 2001;37(6):703-10.

51. Huchon G, Gialdroni-Grassi G, Leophonte P, et al. Initial antibiotic therapy for lower respiratory tract infection in the community: a European survey. *European Respiratory Journal* 1996;9(8):1590-95.

52. Spurling GKP, Del Mar CB, Dooley L, et al. Delayed antibiotic prescriptions for respiratory infections. *Cochrane Database of Systematic Reviews* 2017(9) doi: 10.1002/14651858.CD004417.pub5

53. Amato L, Davoli M, Ferri M, et al. Effectiveness of interventions on opiate withdrawal treatment: an overview of systematic reviews. *Drug and Alcohol Dependence* 2004;73(3):219-26. doi: <https://doi.org/10.1016/j.drugalcdep.2003.11.002>

54. Stephenson J. Experts debate merits of 1-day opiate detoxification under anesthesia. *JAMA* 1997;277(5):363-64. doi: 10.1001/jama.1997.03540290015006

55. Gowing L, Ali R, White JM. Opioid antagonists under heavy sedation or anaesthesia for opioid withdrawal. *Cochrane Database of Systematic Reviews* 2010(1) doi: 10.1002/14651858.CD002022.pub3

56. Gore JM, Goldberg RJ, Spodick DH, et al. A Community-Wide Assessment of the Use of Pulmonary Artery Catheters in Patients with Acute Myocardial Infarction. *Chest* 1987;92(4):721-27. doi: <https://doi.org/10.1378/chest.92.4.721>

57. Lee DS, Austin PC, Rouleau JL, et al. Predicting mortality among patients hospitalized for heart failure: Derivation and validation of a clinical model. *JAMA* 2003;290(19):2581-87. doi: 10.1001/jama.290.19.2581

58. Rajaram SS, Desai NK, Kalra A, et al. Pulmonary artery catheters for adult patients in intensive care. *Cochrane Database of Systematic Reviews* 2013(2) doi: 10.1002/14651858.CD003408.pub3

59. IRI: Overall Supplement Sales Slightly Up 2002 [Available from: [https://www.naturalproductsinsider.com/articles/2002/03/iri-overall-supplement-sales-slightly-up.aspx#](https://www.naturalproductsinsider.com/articles/2002/03/iri-overall-supplement-sales-slightly-up.aspx) accessed January 18 2018.

60. Vassar MJ, Fischer RP, O'Brien PE, et al. A multicenter trial for resuscitation of injured patients with 7.5% sodium chloride: The effect of added dextran 70. *Archives of Surgery* 1993;128(9):1003-13. doi: 10.1001/archsurg.1993.01420210067009

61. Kramer GC. Hypertonic resuscitation: physiologic mechanisms and recommendations for trauma care. *Journal of Trauma and Acute Care Surgery* 2003;54(5):S89-S99.

62. Education L, Magazines T. Guidelines for prehospital management of traumatic brain injury. *Journal of neurotrauma* 2004;19(1):111-74.

63. Berger-Pelleiter E, Émond M, Lauzier F, et al. Hypertonic saline in severe traumatic brain injury: a systematic review and meta-analysis of randomized controlled trials. *CJEM* 2016;18(2):112-20. doi: 10.1017/cem.2016.12 [published Online First: 03/18]

64. Frostell CG, Blomqvist H, Hedenstierna G, et al. Inhaled nitric oxide selectively reverses human hypoxic pulmonary vasoconstriction without causing systemic vasodilation. *Anesthesiology* 1993;78(3):427-35.

65. Rossaint R, Gerlach H, Schmidt-Ruhnke H, et al. Efficacy of Inhaled Nitric Oxide in Patients With Severe ARDS. *Chest* 1995;107(4):1107-15. doi: <https://doi.org/10.1378/chest.107.4.1107>

66. Beloucif S, Payen D. A European survey of the use of inhaled nitric oxide in the ICU. *Intensive care medicine* 1998;24(8):864-77.

67. Gebistorf F, Karam O, Wetterslev J, et al. Inhaled nitric oxide for acute respiratory distress syndrome (ARDS) in children and adults. *Cochrane Database of Systematic Reviews* 2016(6) doi: 10.1002/14651858.CD002787.pub3

68. Byrd Jr BF, Burch JC, Vaughn WK. The impact of long term estrogen support after hysterectomy. A report of 1016 cases. *Annals of surgery* 1977;185(5):574.

69. Barrett-Connor E. The Estrogen Elixir: A history of hormone replacement therapy in America. *New England Journal of Medicine* 2007;357(16):1670-71. doi: 10.1056/NEJMbkrev58569

70. The Women's Health Initiative Steering C. Effects of conjugated equine estrogen in postmenopausal women with hysterectomy: The women&#39;s health initiative randomized controlled trial. *JAMA* 2004;291(14):1701-12. doi: 10.1001/jama.291.14.1701

71. Marjoribanks J, Farquhar C, Roberts H, et al. Long-term hormone therapy for perimenopausal and postmenopausal women. *Cochrane Database of Systematic Reviews* 2017(1) doi: 10.1002/14651858.CD004143.pub5

72. Bethea CL, Lu NZ, Gundlah C, et al. Diverse Actions of Ovarian Steroids in the Serotonin Neural System. *Frontiers in Neuroendocrinology* 2002;23(1):41-100. doi: <https://doi.org/10.1006/frne.2001.0225>

73. Delva MD. Hormone replacement therapy. Risks, benefits, and costs. *Canadian Family Physician* 1993;39:2149-54.

74. Connelly MT, Richardson M, Platt R. Prevalence and Duration of Postmenopausal Hormone Replacement Therapy Use in a Managed Care Organization, 1990–1995. *Journal of General Internal Medicine* 2000;15(8):542-50. doi: doi:10.1046/j.1525-1497.2000.03499.x

75. Ball C, Adams J, Boyce S, et al. Clinical guidelines for the use of the prone position in acute respiratory distress syndrome. *Intensive and Critical Care Nursing* 2001;17(2):94-104. doi: <https://doi.org/10.1054/iccn.2001.1556>

76. Mure M, Martling C-R, Lindahl SG. Dramatic effect on oxygenation in patients with severe acute lung insufficiency treated in the prone position. *Critical care medicine* 1997;25(9):1539-44.

77. Bloomfield R, Noble DW, Sudlow A. Prone position for acute respiratory failure in adults. *Cochrane Database of Systematic Reviews* 2015(11) doi: 10.1002/14651858.CD008095.pub2

78. Parker M, District P, Cambridgeshire P. hip protectors reduce hip fractures after falls in elderly people living in institutions or supported home environments. 2002

79. Lauritzen JB, Petersen MM, Lund B. Effect of external hip protectors on hip fractures. *The Lancet* 1993;341(8836):11-13. doi: <https://doi.org/10.1016/0140-6736(93)92480-H>

80. Chan DK, Hillier G, Coore M, et al. Effectiveness and acceptability of a newly designed hip protector: a pilot study. *Archives of Gerontology and Geriatrics* 2000;30(1):25-34. doi: <https://doi.org/10.1016/S0167-4943(99)00048-5>

81. van Schoor NM, Deville W, Bouter LM, et al. Acceptance and compliance with external hip protectors: a systematic review of the literature. *Osteoporosis International* 2002;13(12):917-24.

82. Connolly S, Sheldon R, Roberts R, et al. The North American vasovagal pacemaker study (VPS) §§A list of members appears in the Appendix.22Editorials published in Journal of the American College of Cardiologyreflect the views of the authors and do not necessarily represent the views of JACCor the American College of Cardiology.: A randomized trial of permanent cardiac pacing for the prevention of vasovagal syncope. *Journal of the American College of Cardiology* 1999;33(1):16-20. doi: <https://doi.org/10.1016/S0735-1097(98)00549-X>

83. Sutton R, Brignole M, Menozzi C, et al. Dual-Chamber Pacing in the Treatment of Neurally Mediated Tilt-Positive Cardioinhibitory Syncope. *Pacemaker Versus No Therapy: A Multicenter Randomized Study* 2000;102(3):294-99. doi: 10.1161/01.cir.102.3.294

84. Benditt DG, Petersen M, Lurie KG, et al. CArdiac pacing for prevention of recurrent vasovagal syncope. *Annals of Internal Medicine* 1995;122(3):204-09. doi: 10.7326/0003-4819-122-3-199502010-00008

85. Romme JJCM, Reitsma JB, Black CN, et al. Drugs and pacemakers for vasovagal, carotid sinus and situational syncope. *Cochrane Database of Systematic Reviews* 2011(10) doi: 10.1002/14651858.CD004194.pub3

86. Brett KM, Madans JH. Use of postmenopausal hormone replacement therapy: estimates from a nationally representative cohort study. *American journal of epidemiology* 1997;145(6):536-45.

87. Kawas C, Resnick S, Morrison A, et al. A Prospective Study of Estrogen Replacement Therapy and the Risk of Developing Alzheimer's Disease1997.

88. Waring SC, Rocca WA, Petersen RC, et al. Postmenopausal estrogen replacement therapy and risk of AD. *A population-based study* 1999;52(5):965-65. doi: 10.1212/wnl.52.5.965

89. Lethaby A, Hogervorst E, Richards M, et al. Hormone replacement therapy for cognitive function in postmenopausal women. *Cochrane Database of Systematic Reviews* 2008(1) doi: 10.1002/14651858.CD003122.pub2

90. Jacobs DM, Tang M-X, Stern Y, et al. Cognitive function in nondemented older women who took estrogen after menopause. *Neurology* 1998;50(2):368-73. doi: 10.1212/wnl.50.2.368

91. Jarvik JG, Deyo RA. DIagnostic evaluation of low back pain with emphasis on imaging. *Annals of Internal Medicine* 2002;137(7):586-97. doi: 10.7326/0003-4819-137-7-200210010-00010

92. Koes BW, van Tulder MW, Ostelo R, et al. Clinical guidelines for the management of low back pain in primary care: an international comparison. *Spine* 2001;26(22):2504-13.

93. Rao JK, Kroenke K, Mihaliak KA, et al. Can guidelines impact the ordering of magnetic resonance imaging studies by primary care providers for low back pain? *American Journal of Managed Care* 2002;8(1):27-36.

94. Lurie JD, Birkmeyer NJ, Weinstein JN. Rates of advanced spinal imaging and spine surgery. *Spine* 2003;28(6):616-20.

95. Chou R, Fu R, Carrino JA, et al. Imaging strategies for low-back pain: systematic review and meta-analysis. *The Lancet* 2009;373(9662):463-72. doi: 10.1016/S0140-6736(09)60172-0

96. Marjoribanks J, Farquhar C, Roberts H, et al. Long‐term hormone therapy for perimenopausal and postmenopausal women. *Cochrane Database of Systematic Reviews* 2017(1) doi: 10.1002/14651858.CD004143.pub5

97. Kuiper GGJM, Carlsson B, Grandien K, et al. Comparison of the Ligand Binding Specificity and Transcript Tissue Distribution of Estrogen Receptors α and β. *Endocrinology* 1997;138(3):863-70. doi: 10.1210/endo.138.3.4979

98. Harris PF, Remington PL, Trentham-Dietz A, et al. Prevalence and treatment of menopausal symptoms among breast cancer survivors. *Journal of pain and symptom management* 2002;23(6):501-09.

99. Lethaby A, Marjoribanks J, Kronenberg F, et al. Phytoestrogens for menopausal vasomotor symptoms. *Cochrane Database of Systematic Reviews* 2013(12) doi: 10.1002/14651858.CD001395.pub4

100. Huffman J, Osborne S, Sokol J. Electrical stimulation in the treatment of intractable stress incontinence; a preliminary report. *Archives of physical medicine and rehabilitation* 1952;33(11):674.

101. Moore T, Schofield PF. Treatment of stress incontinence by maximum perineal electrical stimulation. *British medical journal* 1967;3(5558):150.

102. 510(k) Premarket Notification NEOCONTROL PELVIC FLOOR THERAPY SYSTEM. In: Administration UFD, ed.: FDA.

103. Pelvic Floor Stimulation as a Treatment of Urinary and Fecal Incontinence: Blue Cross of Idaho; 2017 [Available from: <https://www.bcidaho.com/providers/medical_policies/dme/mp_10117.asp> accessed January 1 2017.

104. Stewart F, Berghmans B, Bø K, et al. Electrical stimulation with non-implanted devices for stress urinary incontinence in women. *Cochrane Database of Systematic Reviews* 2017(12) doi: 10.1002/14651858.CD012390.pub2

105. Block G, Cox C, Madans J, et al. VITAMIN SUPPLEMENT USE, BY DEMOGRAPHIC CHARACTERISTICS. *American Journal of Epidemiology* 1988;127(2):297-309. doi: 10.1093/oxfordjournals.aje.a114805

106. Dorgan J, Schatzkin A. Antioxidant micronutrients in cancer prevention. *Hematology/oncology clinics of North America* 1991;5(1):43-68.

107. group TAcps. The alpha-tocopherol, beta-carotene lung cancer prevention study: Design, methods, participant characteristics, and compliance. *Annals of Epidemiology* 1994;4(1):1-10. doi: <https://doi.org/10.1016/1047-2797(94)90036-1>

108. Bjelakovic G, Nikolova D, Gluud LL, et al. Antioxidant supplements for prevention of mortality in healthy participants and patients with various diseases. *Cochrane Database of Systematic Reviews* 2012(3) doi: 10.1002/14651858.CD007176.pub2

109. Dev S. Ethnotherapeutics and modern drug development: the potential of Ayurveda. *Current science* 1997;73(11):909-28.

110. Urizar NL, Liverman AB, Dodds DNT, et al. A Natural Product That Lowers Cholesterol As an Antagonist Ligand for FXR. *Science* 2002;296(5573):1703-06. doi: 10.1126/science.1072891

111. Szapary PO, Wolfe ML, Bloedon LT, et al. Guggulipid for the treatment of hypercholesterolemia: A randomized controlled trial. *JAMA* 2003;290(6):765-72. doi: 10.1001/jama.290.6.765

112. Ulbricht C, Basch E, Szapary P, et al. Guggul for hyperlipidemia: a review by the Natural Standard Research Collaboration. *Complementary therapies in medicine* 2005;13(4):279-90.

113. Marjoribanks J, Farquhar C, Roberts H, et al. Long‐term hormone therapy for perimenopausal and postmenopausal women. *The Cochrane Library* 2017

114. Weintraub MI. Magnetotherapy: historical background with a stimulating future. *Critical Reviews™ in Physical and Rehabilitation Medicine* 2004;16(2)

115. Basford JR. A historical perspective of the popular use of electric and magnetic therapy. *Archives of physical medicine and rehabilitation* 2001;82(9):1261-69.

116. Li LC, Bombardier C. Physical Therapy Management of Low Back Pain: An Exploratory Survey of Therapist Approaches. *Physical Therapy* 2001;81(4):1018-28. doi: 10.1093/ptj/81.4.1018

117. Pittler MH, Brown EM, Ernst E. Static magnets for reducing pain: systematic review and meta-analysis of randomized trials. *Canadian Medical Association Journal* 2007;177(7):736-42.

118. Harris JP, Weisman MH, Derebery JM, et al. Treatment of corticosteroid-responsive autoimmune inner ear disease with methotrexate: a randomized controlled trial. *Jama* 2003;290(14):1875-83.

119. Immune-mediated inner ear disease: 10-year experience. Seminars in arthritis and rheumatism; 2004. Elsevier.

120. Rauch SD. Clinical Management of Immune‐mediated Inner‐ear Disease. *Annals of the New York Academy of Sciences* 1997;830(1):203-10.

121. Lasser EC, Lyon SG, Berry CC. Reports on contrast media reactions: analysis of data from reports to the US Food and Drug Administration. *Radiology* 1997;203(3):605-10.

122. Mathur VS, Swan SK, Lambrecht LJ, et al. The effects of fenoldopam, a selective dopamine receptor agonist, on systemic and renal hemodynamics in normotensive subjects. *Critical care medicine* 1999;27(9):1832-37.

123. Hunter DW, Chamsuddin A, Bjarnason H, et al. Preventing contrast-induced nephropathy with fenoldopam. *Techniques in Vascular and Interventional Radiology* 2001;4(1):53-56. doi: <https://doi.org/10.1053/tvir.2001.21994>

124. Naeem M, McEnteggart GE, Murphy TP, et al. Fenoldopam for the prevention of contrast-induced nephropathy (CIN)—do we need more trials? A meta-analysis. *Clinical Imaging* 2015;39(5):759-64. doi: <https://doi.org/10.1016/j.clinimag.2015.02.003>

125. Bennett D, Boldt J, Brochard L, et al. Expert panel: the use of the pulmonary artery catheter. *Intensive care medicine* 1991;17(3):I-VIII.

126. Connors AF, Jr, Speroff T, et al. The effectiveness of right heart catheterization in the initial care of critically ill patients. *JAMA* 1996;276(11):889-97. doi: 10.1001/jama.1996.03540110043030

127. Saguil A, Fargo M. Acute respiratory distress syndrome: diagnosis and management. *American family physician* 2012;85(4):352-58.

128. Canedy D. Real Medicine or Medicine Show?; Growth of Herbal Remedy Sales Raises Issues About Value. *NY Times* 1998 July 23.

129. Melchart D, Linde K, Worku F, et al. Results of five randomized studies on the immunomodulatory activity of preparations of Echinacea. *The Journal of Alternative and Complementary Medicine* 1995;1(2):145-60.

130. O'Hara M, Kiefer D, Farrell K, et al. A review of 12 commonly used medicinal herbs. *Archives of Family Medicine* 1998;7(6):523.

131. Karsch-Völk M, Barrett B, Kiefer D, et al. Echinacea for preventing and treating the common cold. *Cochrane Database of Systematic Reviews* 2014(2) doi: 10.1002/14651858.CD000530.pub3

132. Taylor S, Kapur M, Adie R. Combined thyroxine and triiodothyronine for thyroid replacement therapy. *Br Med J* 1970;2(5704):270-71.

133. Smith R, Taylor S, Massey J. Controlled clinical trial of combined triiodothyronine and thyroxine in the treatment of hypothyroidism. *Br Med J* 1970;4(5728):145-48.

134. Kaufman SC, Gross TP, Kennedy DL. Thyroid hormone use: trends in the United States from 1960 through 1988. *Thyroid* 1991;1(4):285-91.

135. Jonklaas J, Bianco AC, Bauer AJ, et al. Guidelines for the treatment of hypothyroidism: prepared by the american thyroid association task force on thyroid hormone replacement. *Thyroid* 2014;24(12):1670-751.

136. Escobar-Morreale HcF, Botella-Carretero JI, del Rey FE, et al. Treatment of Hypothyroidism with Combinations of Levothyroxine plus Liothyronine. *The Journal of Clinical Endocrinology & Metabolism* 2005;90(8):4946-54. doi: 10.1210/jc.2005-0184

| **#** | **Article and Author** | **Medical Discipline(s)** | **Date and Journal** | **Summary** | **Systematic review** | **Systematic review search terms** |
| --- | --- | --- | --- | --- | --- | --- |
| 114 | Thromboprophylaxis after Knee Arthroscopy and Lower-Leg Casting Adrichem et al. | Surgery | 2/09/2017 NEJM | After patients undergo arthroscopic knee surgery or are placed in a lower leg cast, it is often recommended that these patients be administered low-molecular-weight heparin (LMWH) to reduce the risk of venous thromboembolism.^1^ However, the Prevention of Thromobosis after Knee Arthroscopy (POT-KAST) (n=1543) and the Prevention of Thrombosis after Lower Leg Plaster Cast (POT-CAST) (n=1519) trials demonstrated that there was no significant difference in terms of venous thromboembolism when comparing patients who were given heparin as compared with placebo (POT-KAST: relative risk, 1.6; 95% confidence interval [CI], 0.4 to 6.8; absolute difference in risk, 0.3 percentage points; 95% CI, −0.6 to 1.2) and (POT-CAST: relative risk, 0.8; 95% CI, 0.3 to 1.7; absolute difference in risk, −0.4 percentage points; 95% CI, −1.8 to 1.0). This is a reversal of administering heparin as thromboprophylaxis after knee arthroscopy and lower-leg casting. | 2017. Cochrane review. "Moderate-quality evidence showed that the use of LMWH in outpatients reduced DVT when immobilization of the lower limb was required, when compared with no prophylaxis or placebo. The quality of the evidence was reduced to moderate because of risk of selection and attrition bias in the included studies. Low-quality evidence showed no clear differences in PE between the LMWH and control groups, but less symptomatic VTE in the LMWH groups. The quality of the evidence was downgraded due to risk of bias and imprecision."^2^  Trial by Adrichem et al. is the only large randomized study (n=1543) in this review. All the other trials combined, Bruntink 2017, Jorgensen 2002, Kock 1995, Lapidus 2007b, Lassen 2002, enrolled a total of n=1484 patients. Therefore, we believe the Adrichem et al. study best represents the effects of administering heparin for the prevention of venous thromboembolism in patients with lower-limb immobilization. | PubMed found systematic review |
| 115 | Tight Glycemic Control in Critically Ill Children Agus et al. | Pediatrics | 2/23/2017 NEJM | A single-center randomized trial found that tight glycemic control to a blood glucose level of 80 to 110 mg/dl reduced morbidity and mortality in critically ill adult patients in 2001,^3^ but since then, results of tight control in this population have not shown benefit. Hyperglycemia in critically ill children is associated with poor outcomes and a single-centered randomized trial in children who had undergone cardiac surgery showed lower mortality and infection rates in lower glucose targets compared to higher targets.^4^ Further trials have had mixed results, but practitioners treat children in critical condition with both low and higher glycemic targets.^5^ This study (n=713) found that tight glycemic control targeted to a blood glucose level of 80 to 110 mg/dl did not reduce rate of ICU-free days in critically ill children with hyperglycemia compared to a level of 150 to 180 mg/dl (19.4 days [interquartile range  , 0 to 24.2] and 19.4 days [IQR, 6.7 to 23.9], respectively; P=0.58). This is a reversal of tight glycemic control in critically ill children. | 2018. “Tight glycemic control [TGC] was not associated with reducing 30-day mortality rates and acquired infections compared with [conventional glucose control] CGC in critically ill children. Significant increase of incidence of hypoglycemia was revealed in TGC group.”^6^  2017. “Network meta-analysis showed no mortality benefit of tight glycemic control in critically ill patients, but fivefold more hypoglycemia versus mild or very mild control.”^7^ (NEJM article not part of analysis) | Google scholar within citing articles |
| 116 | Treatment of Subclinical Hypothyroidism or Hypothyroxinemia in Pregnancy Casey et al. | Obstetrics and Gynecology | 3/02/2017 NEJM | Observational studies have shown an association between subclinical hypothyroidism or hypothyroxinemia in pregnancy and impaired fetal neuropsychological development.^8^ As a result, it is recommended that pregnant women with subclinical hypothyroidism be treated with levothyroxine.^9,10^ This study (n=677) found that treatment with levothyroxine for subclinical hypothyroidism or hypothyroxinemia beginning between 8-20 weeks of gestation did not improve cognitive outcomes in children compared to placebo. The median IQ score of the children was 97 (95% confidence interval [CI], 94 to 99) in the levothyroxine group and 94 (95% CI, 92 to 96) in the placebo group (P=0.71). In the hypothyroxinemia trial, the median IQ score was 94 (95% CI, 91 to 95) in the levothyroxine group and 91 (95% CI, 89 to 93) in the placebo group (P=0.30). This is a reversal of the treatment of subclinical hypothyroidism or hypothyroxinemia during pregnancy in women with either subclinical hypothyroidism or hypothyroxinemia and an ultrasonographically verified singleton pregnancy between 8 to 20 weeks of gestation. | 2018. "Currently, there is no evidence that levothyroxine treatment, when initiated 8- to 20-week gestation (mostly between 12 and 17 weeks), for mild maternal thyroid hormone insufficiency during pregnancy reduces intellectual disability in offspring."^11^ | NEJM found in "citing articles" section |
| 117 | Trial of Pregabalin for Acute and Chronic Sciatica Mathieson et al. | Neurology/Neurosurgery | 3/23/2017 NEJM | There is currently no standard of care for the treatment of sciatica^12,13^ Pregabalin is a FDA-approved drug^14^ generally used for neuropathic pain and is often prescribed as treatment for sciatica, despite the lack of evidence to support its efficacy.^15^ This trial (n=209) found that pregabalin did not improve leg-pain intensity score compared to placebo over an 8-week period in patients with sciatica (adjusted mean difference, 0.5; 95% confidence interval [CI], −0.2 to 1.2; P=0.19). Adverse events were significantly higher in the pregabalin group. This is a reversal on pregabalin use for acute and chronic sciatica in patients who visited a trial clinician as an outpatient in New South Wales, Australia | 2018. “Evidence to date does not support the use of anticonvulsants [gabapentin, pregabalin] for chronic low back pain or lumbar radicular pain. This review found mostly moderate- to high-level quality of evidence suggesting no treatment benefit for pain and disability, and high-level evidence supporting the risk of harms.”^16^ | Google scholar within citing articles |
| 118 | Levosimendan for Hemodynamic Support after Cardiac Surgery Landoni et al. | Cardiovascular disease | 5/25/2017 NEJM | Acute perioperative left ventricular dysfunction occurs in up to 20% of patients undergoing cardiac surgery.^17^ Levosimendan is an inotropic agent commonly used in clinical practices outside of the United States since 2000 for patients with heart failure.^18^ The drug is associated with a higher rate of survival compared to other inotropic drugs for patients undergoing cardiac surgery.^19^ This study (n=506) found that low-dose levosimendan, in addition to standard care, did not lower rates of 30-day mortality compared to placebo in patients requiring perioperative hemodynamic support after cardiac surgery (32 patients [12.9%] and 33 patients [12.8%], respectively; absolute risk difference, 0.1 percentage points; 95% confidence interval [CI], −5.7 to 5.9; P=0.97). This is a reversal of Levosimendan for hemodynamic support after cardiac surgery in patients in whom perioperative hemodynamic support was recommended after cardiac surgery. | 2018.. “There is not enough high-quality evidence to neither support nor discourage the systematic use of [levosimendan](https://www.sciencedirect.com/topics/medicine-and-dentistry/levosimendan) in cardiac surgery.” ^20^  2017. “The available evidence from our updated meta-analysis suggests that levosimendan therapy reduced the risk of death in single-center trials and in trials of inferior quality, but there was no benefit of levosimendan on survival in multicentric and in high-quality trials.”^21^ | Google scholar within citing articles |
| 119 | Levosimendan in Patients with Left Ventricular Dysfunction Undergoing Cardiac Surgery Mehta et al. | Cardiovascular disease | 5/25/2017 NEJM | Low cardiac output syndrome occurs in 3 to 14% of patients who undergo cardiac surgery with the use of cardiopulmonary bypass and the syndrome has high rates of short-term mortality.^22^ Levosimendan is an inotropic agent commonly used in clinical practices outside of the United States since 2000 for patients with heart failure.^18^ Levosimendan is currently used in more than 60 countries for the prevention and treatment of the low cardiac output syndrome.^23,24^ This study (n=882) found that prophylactic levosimendan did not results in a lower rate of the short-term composite end point of death, renal-replacement therapy, perioperative myocardial infarction, or use of a mechanical cardiac assist device compared to placebo in patients with a reduced left ventricular ejection fraction who were undergoing cardiac surgery with the use of cardiopulmonary bypass (adjusted odds ratio, 1.00; 99% confidence interval [CI], 0.66 to 1.54; P=0.98). This is a reversal of administering levosimendan to patients with left ventricular dysfunction undergoing cardiac surgery. | 2018. “There is not enough high-quality evidence to neither support nor discourage the systematic use of [levosimendan](https://www.sciencedirect.com/topics/medicine-and-dentistry/levosimendan) in cardiac surgery.” ^20^ | Pubmed found systematic review |
| 120 | Bioresorbable Scaffolds versus Metallic Stents in Routine PCI Wykrzykowska et al. | Cardiovascular disease | 6/15/2017 NEJM | Bioresorbable vascular scaffolds were designed to leave no permanent implant in place of metal stents. They were approved by the FDA and obtained a Conformite Europeenne mark in 2010.^25,26^ The device has since become part of interventional practice even though there is a lack of evidence for its safety and efficacy. AIDA (n=1845) a single-blind, multicenter, investigator-initiated, noninferiority, randomized clinical trial, found that there was no difference between everolimus-eluting bioresorbable scaffolds and everolimus-eluting metallic stents in rate of target-vessel failure in patients undergoing PCI (2-year cumulative event rates, 11.7% and 10.7%, respectively; hazard ratio, 1.12; 95% confidence interval [CI], 0.85 to 1.48; P=0.43). Furthermore, the bioresorbable scaffold was associated with higher incidence of device thrombosis through 2 years of follow-up. This is a reversal of bioresorbable vascular scaffolds in routine PCI in patients with coronary artery disease who had one or more target lesions that were considered, on the basis of clinical judgment, to be suitable for drug-eluting stent implantation. | 2018. “Compared with [everolimus-eluting metallic stents] EESs, [bioresorbable vascular scaffolds] BVSs were associated with a higher incidence of scaffold thrombosis at all-time courses (early, late, and very late), and relative risks seemed to increase over time. Third, BVSs increased risks for myocardial infarction, TLR, and TLF, with such relative risks also increasing over time. Fourth, risks for all-cause, cardiac, and noncardiac death; TVR; and all revascularization were not statistically significantly different between patients with a BVS and those with an EES”^27^ | Pubmed found systematic review |
| 121 | Thyroid Hormone Therapy for Older Adults with Subclinical Hypothyroidism Stott et al. | Endocrinology, Diabetes, and Metabolism | 6/29/2017 NEJM | Subclinical hypothyroidism is a condition with slightly elevated serum thyrotropin levels and a lack of obvious hypothyroidism symptoms. Subclinical hypothyroidism occurs in 8% to 18% of adults 65 years or older and is associated with coronary heart disease.^28,29^ Levothyroxine is commonly used to treat hypothyroidism and is recommended to treat subclinical hypothyroidism ^30^ even though evidence of its efficacy in this patient subgroup is lacking. This study (n=368) found that levothyroxine did not affect the Hypothyroid Symptoms score (0.2±15.3 in the placebo group and 0.2±14.4 in the levothyroxine group; between-group difference, 0.0; 95% confidence interval [CI], −2.0 to 2.1) or Tiredness score (3.2±17.7 and 3.8±18.4, respectively; between-group difference, 0.4; 95% CI, −2.1 to 2.9) on a thyroid-related quality-of-life questionnaire at 1 year compared to placebo in patients with persistent subclinical hypothyroidism. This is a reversal of thyroid hormone therapy with levothyroxine for older adults with subclinical hypothyroidism. | None found |  |
| 122 | Five-Year Outcomes after On-Pump and Off-Pump Coronary-Artery Bypass Shroyer e. al. | Cardiovascular disease | 8/17/2017 NEJM | Observational studies in the 1990s showed an association between off-pump coronary-artery bypass and better early clinical outcomes compared to on-pump, and the practice of performing coronary-artery bypass surgery on a beating heart repopularized.^31^ Yet randomized controlled trials have not been able to show efficacy in off-pump surgeries and suggested that incomplete revascularization was more frequent with off-pump surgery.^32^ This follow-up study (n=2203) found that 5-year outcomes of death from any cause (relative risk, 1.28; 95% confidence interval [CI], 1.03 to 1.58; P=0.02) and any major adverse cardiovascular events (relative risk, 1.14; 95% CI, 1.00 to 1.30; P=0.046) were worse for patients who underwent coronary-artery bypass surgery off-pump compared to on-pump. This is a reversal of off-pump coronary-artery bypass. | 2018. “This meta-analysis represents a comprehensive  summary of RCTs comparing OPCABG to ONCABG. Our  results showed that OPCABG was associated with no  reduction in operative risk, an excess mortality at follow-up  ≥3 years, and a trend toward higher risk of repeated  revascularization.” ^33^ | in NEJM in cited by articles |
| 123 | Bivalirudin versus Heparin Monotherapy in Myocardial Infarction Erlinge et al. | Cardiovascular disease | 9/21/2017  NEJM | Anticoagulation treatment is common in patients undergoing percutaneous coronary intervention (PCI) to reduce thrombotic complications.^34^ Heparin has been treatment of choice for this intervention, but in 2000, bivalirudin was approved for this indication but at a cost 100X more than heparin.^35,36^ In this RCT (n=6006), patients undergoing PCI who took bivalirudin did not have different rates of death from any cause, myocardial infarction, or major bleeding, compared to those taking heparin(hazard ratio, 0.96; 95% confidence interval [CI], 0.83 to 1.10; P=0.54). This is a reversal of bivalirudin monotherapy in myocardial infarction in patients admitted to the hospital with a diagnosis of STEMI (ST-segment elevation myocardial infarction) or Non-STEMI, prior treatment with ticagrelor, prasugrelor, or cangrelor and that were scheduled for PCI. | None found |  |
| 124 | Oxygen Therapy in Suspected Acute Myocardial Infarction Hofmann et al. | Cardiovascular disease | 9/28/2017 NEJM | Oxygen therapy has routinely been used to treat myocardial infarction (MI) because supplemental oxygen is thought to increase oxygen delivery to the ischemic myocardium and reduce infarct size.^37^ Oxygen therapy is recommended in clinical guidelines, yet there has not been substantial evidence to back up the practice.^38,39^ This study (n=6629) found that supplemental oxygen in patients with suspected MI who did not have hypoxemia did not reduce 1-year all-cause mortality (hazard ratio, 0.97; 95% confidence interval [CI], 0.79 to 1.21; P=0.80). This is a reversal of oxygen therapy in patients with suspected acute MI who were 30 years of age or older, had symptoms suggestive of myocardial infarction (less than 6 hours), an oxygen saturation of 90% or higher, and either electrocardiographic changes indicating ischemia or elevated cardiac troponin T or I levels on admission. | 2016. Cochrane review. “There is no evidence from randomised controlled trials to support the routine use of inhaled oxygen in people with AMI, and we cannot rule out a harmful effect. Given the uncertainty surrounding the effect of oxygen therapy on all-cause mortality and on other outcomes critical for clinical decision, well-conducted, high quality randomised controlled trials are urgently required to inform guidelines in order to give definitive recommendations about the routine use of oxygen in AMI.”^40^ review preceded study | Pubmed “similar article” search |
| 125 | Lomustine and Bevacizumab in Progressive Glioblastoma Wick et al. | Oncology | 11/16/2017 NEJM | Progressive glioblastoma is an aggressive cancer with no standard of care. Bevacizumab is a well-tolerated mAb targeting VEGF (vascular endothelial growth factor) that has been suggested to treat glioblastoma.^41^ Avastin (generically bevacizumab) was granted accelerated approval by the FDA in 2009 for people with glioblastoma with progressive disease following prior therapy.^42^ This study found that bevacizumab in combination with lomustine had no greater effect on survival advantage than lomustine alone in patients with progressive glioblastoma. The median overall survival was 9.1 months (95% confidence interval [CI], 8.1 to 10.1) in the combination group and 8.6 months (95% CI, 7.6 to 10.4) in the monotherapy group (hazard ratio for death, 0.95; 95% CI, 0.74 to 1.21; P=0.65). This is a reversal of bevacizumab in combination with lomustine in patients with progressive glioblastoma. | 2018. “Although treatment with [lomustine] CCNU plus [bevacizumab] BEV prolonged [progression free survival] PFS, it did not confer [overall survival] OS advantage over monotherapies in patients with progressive [glioblastoma] GBM.”^43^ | Google scholar within citing articles. |
| 126 | Pharmacomechanical Catheter-Directed Thrombolysis for Deep-Vein Thrombosis Vedentham et al. | Cardiovascular disease | 12/7/2017 NEJM | Patients with deep-vein thrombosis are at risk of developing post-thrombotic syndrome, even with anticoagulant treatment.^44^ Post-thrombotic syndrome causes pain and swelling and can lead to major disability and leg ulcers.^44^ Pharmacomechanical catheter-directed thrombolysis is a treatment that delivers fibrinolytic drugs to the thrombus along with thrombus aspiration or maceration, potentially lowering risk of post-thrombotic syndrome. Pharmacomechanical thrombolysis to treat patients with deep-vein thrombosis has shown some success in retrospective studies, ^45,46^ but the procedure is not widely accepted due to risks such as major bleeding and hospitalization. This study found that pharmacomechanical thrombolysis did not lower risk of post-thrombotic syndrome in patients with acute proximal deep-vein thrombosis (47% in the pharmacomechanical-thrombolysis group and 48% in the control group; risk ratio, 0.96; 95% confidence interval [CI], 0.82 to 1.11; P=0.56). Thrombolysis increased risk of major bleeding among as well. This is a reversal on pharmacomechanical catheter-directed thrombolysis for deep-vein thrombosis in patients with symptomatic proximal deep-vein thrombosis involving the femoral, common femoral, or iliac vein (with or without other involved ipsilateral veins). | None found |  |
| 127 | Outcomes of a Coaching-Based WHO Safe Childbirth Checklist Program in India Semrau et al. | Obstetrics and Gynecology | 12/14/2017 NEJM | Maternal and neonatal mortality rates are high in low-income and middle-income countries compared to high-income regions, although rates have decreased over the last few decades. Shifting births from home to facilities has not produced the improved outcomes it was expected to.^47^ This is likely due to the lack of adherence to safe birthing practices by facility birth attendants. The World Health Organization created a practical tool called the Safe Childbirth Checklist to aid birth attendants in planning for and performing important birth practices known to reduce risk of mortality.^48^ The Safe Childbirth Checklist has been shown to improve facility-based birth attendants' adherence to evidence-based care in small-scale trials.^49^ This trial found that the WHO Safe Childbirth Checklist program increased adherence to essential birth practices in birthing facilities, but did not decrease risk of maternal and perinatal mortality and maternal morbidity (15.1% in the intervention group and 15.3% in the control group; relative risk, 0.99; 95% confidence interval, 0.83 to 1.18; P=0.90).This trial is a reversal of a coaching-based WHO safe childbirth checklist program in India. | None found |  |
| 128 | PCI Strategies in Patients with Acute Myocardial Infarction and Cardiogenic Shock Thiele et al. | Cardiovascular disease | 12/21/2017 NEJM | Percutaneous coronary intervention (PCI) reduces risk of mortality in patients with cardiogenic shock in acute myocardial infarction by restoring blood flow to the culprit coronary artery.^50^ Yet in patients with cardiogenic shock presenting with multivessel coronary artery disease, it is unclear whether performing immediate PCI for all coronary arteries with clinically important stenosis is more effective than PCI for the culprit lesion. Some believe that the former has the potential to improve overall myocardial perfusion and function, but the procedure has increased risks. While international guidelines do not recommend multivessel PCI, the procedure is routinely used to treat multivessel lesions.^51^ This study found that patients with multivessel coronary artery disease and acute myocardial infarction with cardiogenic shock had a lower risk of death or severe renal failure with PCI of the culprit lesion only compared to immediate multivessel PCI (relative risk, 0.83; 95% confidence interval [CI], 0.71 to 0.96; P=0.01). The relative risk of death in the culprit-lesion-only PCI group as compared with the multivessel PCI group was 0.84 (95% CI, 0.72 to 0.98; P=0.03). This is a reversal of immediate multivessel PCI in patients with acute myocardial infarction and cardiogenic shock. | None found |  |
| 129 | Delayed versus Immediate Cord Clamping in Preterm Infants Tarnow-Mordi et al. | Obstetrics and Gynecology | 12/21/2017  NEJM | The timing of cord clamping in newborns has been debated. Advocates for delayed cord clamping assert that delaying clamping allows for the baby to receive more placental blood, while advocates of early clamping respond that this could lead to an increase in polycythemia or jaundice and is awkward for the person delivering the baby.^52^ While some birthing centers may have protocol for delayed clamping, there is wide variation in actual practice.^53^ In this RCT, it was found that delayed cord clamping was no better at lowering death or major morbidity (relative risk, 1.00; 95% confidence interval, 0.88 to 1.13; P=0.96). This is a reversal on delayed cord clamping in preterm infants (expected to be delivered before 30 weeks of gestation). | 2018. “This systematic review provides high-quality evidence that delayed clamping reduced hospital mortality, which supports current guidelines recommending delayed clamping in preterm infants."^54^  The Tarnow-Mordi et al. paper is the single dominant data point in this entire space. | pubmed "similar articles" search |
| 130 | Routine Amoxicillin for Uncomplicated Severe Acute Malnutrition in Children Isanaka et al. | Pediatrics | 2/4/2016 NEJM | Since 1999, the World Health Organization has recommended broad-spectrum antibiotic treatment for children suffering from severe acute malnutrition due to the possibility that they will contract bacterial infections and worsen their nutritional status.^55^ Evidence to support this recommendation is limited, especially in light of the risk of antibiotic resistance from routine antibiotic use. This double blind, placebo-controlled trial contradicted the WHO recommendation, demonstrating zero benefit with regard to nutritional recovery for severely malnourished children who had taken a broad-spectrum antibiotic (risk ratio for amoxicillin vs. placebo, 1.05; 95% confidence interval [CI], 0.99 to 1.12; P=0.10). This is a reversal of routine amoxicillin for uncomplicated severe acute malnutrition in children. | None found |  |
| 131 | Adjunctive Dexamethasone in HIV-Associated Cryptococcal Meningitis Beardsley et al. | Infectious disease | 2/11/2016 NEJM | Cryptococcal meningitis is often associated with human immunodeficiency virus (HIV) and is highly deadly. Guidelines often recommend glucocorticoids - such as dexamethasone - for treatment of cryptococcal meningitis as a coinfection with HIV.^56^ However, a double blind, randomized, placebo-controlled trial, found that use of dexamethasoze for adjunctive therapy did not improve survival among adults suffering from cryptococcal meningitis associated with HIV. There was no reduction in mortality among this group when compared to placebo by 10 weeks (hazard ratio in the dexamethasone group, 1.11; 95% confidence interval [CI], 0.84 to 1.47; P=0.45). This is a reversal of adjunctive dexamethasone in HIV-associated cryptococcal meningitis. | None found |  |
| 132 | A Randomized Trial of a Cervical Pessary to Prevent Preterm Singleton Birth Nicolaides et al. | Obstetrics and Gynecology | 3/17/2016 NEJM | The transvaginal placement of a silicone pessary is often recommended for pregnant women with a short cervix given their increased risk of spontaneous delivery prior to 34 weeks of gestation. It is believed that this device reduces direct pressure on the cervix and prolongs pregnancy.^57^ This randomized trial compared spontaneous preterm births among women with pessaries with those who underwent expectant management and found that the pessary had no significant effect on the rate of preterm delivery (12.0% and 10.8%, respectively; odds ratio in the pessary group, 1.12; 95% confidence interval, 0.75 to 1.69; P=0.57). This is a reversal on a cervical pessary to prevent preterm singleton birth of women 16 years or older with a cervical length of 25 mm or less. | 2017. J Ultrasound Med. “In singleton pregnancies with a [transvaginal ultrasound cervical length] TVU CL ≤25mm at 200–246 weeks, the Arabin pessary does not reduce the rate of spontaneous preterm delivery or improve perinatal outcome.”^58^ | Pubmed found systematic review |
| 133 | Early versus Late Parenteral Nutrition in Critically Ill Children Fivez et al. | Critical care medicine | 3/24/2016 NEJM | Among children who are critically ill, macro nutrition is vital. Among such children who cannot be fed by mouth or enteral feeding, standard practice is to administer parenteral nutrition as early as possible.^59,60^ This study found that withholding parenteral nutrition was superior to administering parenteral feeding to these children, regardless of the severity of the child's condition. Children with delayed parenteral nutrition had fewer new infections, shorter hospital stays and less dependency on intensive care overall (adjusted odds ratio, 0.48; 95% confidence interval [CI], 0.35 to 0.66). The mean (±SE) duration of ICU stay was 6.5±0.4 days in the group receiving late parenteral nutrition, as compared with 9.2±0.8 days in the group receiving early parenteral nutrition; there was also a higher likelihood of an earlier live discharge from the ICU at any time in the late-parenteral-nutrition group (adjusted hazard ratio, 1.23; 95% CI, 1.11 to 1.37). This is a reversal on early parenteral nutrition in critically ill children needing to stay in the pediatric ICU for 24 hours or more. | None found |  |
| 134 | Randomized Trial of Longer-Term Therapy for Symptoms Attributed to Lyme Disease Berende et al. | Public health and general preventive medicine | 3/31/2016 NEJM | Guidelines for the management of patients with Lyme disease differ - with some recommending prolonged treatment with antibiotics for four weeks or more.^61^ This randomized, double-blind placebo-controlled trial (n=281) allocated participants to a 12-week oral course of doxycycline, clarithromycin plus hydroxychloroquine, or placebo. They found that longer-term treatment with antibiotic did not result in improved quality of life when compared with the shorter course in patients with persistent symptoms attributed to Lyme disease. The SF-36 physical-component summary score did not differ significantly among the three study groups at the end of the treatment period, with mean scores of 35.0 (95% confidence interval [CI], 33.5 to 36.5) in the doxycycline group, 35.6 (95% CI, 34.2 to 37.1) in the clarithromycin–hydroxychloroquine group, and 34.8 (95% CI, 33.4 to 36.2) in the placebo group (P=0.69; a difference of 0.2 [95% CI, –2.4 to 2.8] in the doxycycline group vs. the placebo group and a difference of 0.9 [95% CI, –1.6 to 3.3] in the clarithromycin–hydroxychloroquine group vs. the placebo group); the score also did not differ significantly among the groups at subsequent study visits (P=0.35). This is a reversal of longer-term antibiotic treatment for symptoms attributed to Lyme disease. | None found |  |
| 135 | Effect of Avoidance on Peanut Allergy after Early Peanut Consumption Du Toit et al. | Allergy and Immunology | 4/14/2016 NEJM | Given the high prevalence of peanut allergy, various theories exist to explain its origin. The most sustained perspective at the time of this article's writing is that infants should be exposed to peanuts early, in the first 11 months of life, to prevent the development of peanut allergy as evidenced by the Learning Early about Peanut Allergy (LEAP).^62,63^ However, an extension of the LEAP trial (n=556) found conflicting evidence. When the children who had been exposed to peanuts in the first 11 months of life were compared to those who had not later on at 5 years of age, there was no difference in prevalence of peanut allergy when comparing the two groups (3.6% [10 of 274 participants] at 60 months and 4.8% [13 of 270] at 72 months, P=0.25).This is a reversal on 12 month period of peanut avoidance. | None found |  |
| 136 | A Randomized, Controlled Trial of Fusion Surgery for Lumbar Spinal Stenosis Försth et al. | Orthopedic | 4/14/2016 NEJM | Surgery for lumbar spinal stenosis is becoming more common, specifically decompression by laminectomy with or without spinal fusion.^64,65^ Despite the growing popularity of spinal fusion - especially among patients with degenerative spondylolisthesis - this multicenter, open-label, clinical superiority trial (n=247) found no difference in outcomes at 2 years and 5 years between decompression surgeries coupled with fusion when compared to decompression alone. There was no significant difference between the groups in the mean score on the [Oswestry Disability Index] ODI at 2 years (27 in the fusion group and 24 in the decompression-alone group, P=0.24) or in the results of the 6-minute walk test (397 m in the fusion group and 405 m in the decompression-alone group, P=0.72). This is a reversal of decompression by laminectomy with spinal fusion surgery for lumbar spinal stenosis in patients with degenerative spondylolisthesis (vertebra that had slipped forward at least 3mm relative to the vertebra below it). | 2018. “Currently, there is not enough evidence that concomitant instrumented fusion in patients with symptomatic lumbar spinal canal stenosis and degenerative spondylolisthesis leads to better clinical outcome than decompression alone. Decompression alone is a more cost-efective technique and is presumably more associated with fewer complications compared to decompression with concomitant fusion.”^66^  2016. Cochrane review. "The results of this Cochrane review show a paucity of evidence on the efficacy of surgery for lumbar spinal stenosis, as to date no trials have compared surgery with no treatment, placebo or sham surgery. Placebo-controlled trials in surgery are feasible and needed in the field of lumbar spinal stenosis. Our results demonstrate that at present, decompression plus fusion and interspinous process spacers have not been shown to be superior to conventional decompression alone. More methodologically rigorous studies are needed in this field to confirm our results."^67^ | Pubmed found systematic review |
| 137 | Amiodarone, Lidocaine, or Placebo in Out-of-Hospital Cardiac Arrest Kudenchuk et at. | Cardiovascular disease | 5/05/2016 NEJM | Following a shock-refractory ventricular fibrillation or pulseless ventricular tachycardia event, patients are often given antiarrhythmic drugs such as Amiodarone and Lidocaine to assist in successful defibrillation.^68^ However, the effects these drugs on out-of-hospital survival and neurologic outcomes after their administration is unknown, despite these drugs being widely administered. This randomized, double-blind, placebo-controlled trial (n=3026)found that the rate of survival to hospital discharge or favorable neurologic outcome at discharge after treatment with amiodarone or lidocaine was not significantly different when compared to one another or compared to placebo. The difference in survival rate for amiodarone versus placebo was 3.2 percentage points (95% confidence interval [CI], −0.4 to 7.0; P=0.08); for lidocaine versus placebo, 2.6 percentage points (95% CI, −1.0 to 6.3; P=0.16); and for amiodarone versus lidocaine, 0.7 percentage points (95% CI, −3.2 to 4.7; P=0.70). This is a reversal of administering amiodarone and lidocaine in out-of-hospital cardiac arrest patients. | 2018. “The high level evidence supporting the use of antiarrhythmic drugs during CPR for shock refractory pVT/VF or immediately after [return of spontaneous circulation] ROSC is limited and showed no signiﬁcant beneﬁt for critical outcomes of survival at hospital discharge, survival with favorable neurological function and long-term survival.”^69^ | Pubmed found systematic review |
| 138 | Perioperative Rosuvastatin in Cardiac Surgery Zheng et al. | Cardiovascular disease | 5/05/2016 NEJM | Due to their antinflammatory effects and antioxidant effects, current practice guidelines recommend perioperative-statin therapy for patients who have undergone cardiac surgery to prevent atrial fibrillation and other cardiac-related complications.^70,71^ However, evidence to support this recommendation is limited. A randomized, placebo-controlled trial discovered that perioperative rosuvastatin did not reduce atrial fibrillation following surgery (21.1% and 20.5%, respectively; odds ratio 1.04; 95% confidence interval [CI], 0.84 to 1.30; P=0.72) or myocardial damage. This is a reversal of perioperative statin therapy with rosuvastatin for preventing atrial fibrillation or perioperative myocardial damage in cardiac surgery. | 2018. “Our systematic review and meta-analysis suggests that perioperative statin therapy could be protective against postoperative myocardial infarction in non-cardiac surgery but associated with an increased risk of acute kidney injury in cardiac surgery. Statins were associated with an increase in hospital mortality in cardiac surgery in low risk of bias trials.”^72^ | Pubmed found systematic review |
| 139 | Rate Control versus Rhythm Control for Atrial Fibrillation after Cardiac Surgery Gillinov et al. | Cardiovascular disease | 5/19/2016 NEJM | Postoperative atrial fibrillation is associated with higher morbidity and mortality. As such, 2 treatment strategies - heart-rate control or rhythm control - are widely used, but there is conflicting evidence around which method has more advantages.^73^ This multi-site randomized control trial (n=523) compared the total number of hospital days for patients who underwent either heart-rate control or rhythm control and found that there was no difference in hospital days between the groups (median, 5.1 days and 5.0 days, respectively; P=0.76).This is a reversal of the rhythm control strategies for atrial fibrillation after cardiac surgery in patients in which atrial fibrillation persisted for more than 60 minutes or had recurrent episodes of atrial fibrillation while hospitalized. | 2017. “Rhythm control strategies compared with rate control strategies seem to significantly increase the risk of a serious adverse event in patients with atrial fibrillation. Based on current evidence, it seems that most patients with atrial fibrillation should be treated with a rate control strategy unless there are specific reasons (e.g., patients with unbearable symptoms due to atrial fibrillation or patients who are hemodynamically unstable due to atrial fibrillation) justifying a rhythm control strategy. More randomized trials at low risk of bias and low risk of random errors are needed.”^74^ | Pubmed found systematic review |
| 140 | Two-Year Outcomes of Surgical Treatment of Moderate Ischemic Mitral Regurgitation Michler et al. | Cardiovascular disease | 5/19/2016 NEJM | Given the risk of developing ischemic mitral regurgitation following a myocardial infarction, it is often necessary for patients to undergo coronary-artery bypass grafting (CABG). However, the appropriate surgical procedure to manage ischemic mitral regurgitation during CABG remains a matter of debate - namely whether the addition of mitral valve repair to CABG improves survival. Advocates for this technique state that this type of repair directly reduces the risk of heart failure by directly reducing the degree of mitral regurgitation.^75^ However, this multicenter randomized control trial (n=301) found there to be no significant difference in survival (hazard ratio in the combined-procedure group, 0.90; 95% confidence interval, 0.45 to 1.83; P=0.78) or left ventricular reverse remodeling when comparing those who underwent CABG alone and those who underwent CABG and mitral-valve repair. Given the invasive, open-heart exposure necessary to conduct the mitral-valve repair in combination with CABG, this procedure does not appear to offer any additional benefit for patients. This is a reversal of the addition of mitral-valve repair in patients with moderate ischemic mitral regurgitation undergoing CABG. | 2017. “Addition of mitral valve replacement/repair (MVR/Re) to CABG in patients with moderate ischemic mitral regurgitation (MR) did not result in improvement in early or overall mortality, stroke risk, or intermediate markers of LV function when compared with CABG alone.”^76^ | Pubmed found systematic review |
| 141 | Randomized Trial of a Lifestyle Program in Obese Infertile Women Mutsaerts et al. | Endocrinology, Diabetes, and Metabolism | 5/19/2016 NEJM | Considering the negative impacts of obesity on women's reproductive health, it has generally been assumed that weight loss programs that decrease women's body weight by between 5 and 10% would be helpful improve obese women's fertility.^77,78^ However, despite these recommendations, the evidence base to demonstrate the effectiveness of weight loss on improving fertility was lacking. This multicenter randomized trial (n=577) compared women who participated in a six-month lifestyle intervention weight loss program prior to infertility treatment with women who only underwent infertility treatment. They found that the rate of vaginal births 2 years after randomization were lower in the intervention arm as compared to the control group (rate ratio in the intervention group, 0.77; 95% confidence interval, 0.60 to 0.99). This is a reversal of a lifestyle intervention preceding infertility treatment in obese infertile women. | None found |  |
| 142 | Initiation Strategies for Renal-Replacement Therapy in the Intensive Care Unit Gaudry et al. | Critical care medicine | 7/14/2016 NEJM | Whether or not early renal-replacement therapy decreases, mortality among critically ill patients has been a topic of debate. Advocates of early initiation of renal-replacement therapy (RRT) argue that it "may allow for better control of fluid and electrolyte status, removal of uremic toxins, and prevention of complications such as gastric hemorrhage and metabolic encephalopathy."^79^ While an earlier initiation of RRT may reduce acute kidney injury (AKI) symptoms, such as serum pH homeostasis and improve clearance of toxic solutes, it may also unnecessarily expose patients to potential harms (hemorrhage, thrombosis, bacteremia, intradialytic hypotension, hypersensitivity to the extracorporeal circuit, clearance of trace elements, and antibiotics) along with added resource utilization. There remains no rigorous evidence to guide clinicians on this important issue.^80^ The AKIKI study is an unblinded, prospective, multicenter randomized control trial (n=620) whichcompared the mortality of early versus delayed renal-replacement therapy and found no difference in mortality between groups (48.5%; 95% confidence interval [CI], 42.6 to 53.8), and 153 deaths occurred among 308 patients in the delayed-strategy group (49.7%, 95% CI, 43.8 to 55.0; P=0.79).This is a reversal on early initiation strategy for renal replacement therapy in the intensive care unit. | 2017. “This updated meta-analysis showed no added benefit of early initiation of RRT for patients with AKI. The grade evidence generated was of “low quality” and there was a high heterogeneity in the included trials.”^81^ | Pubmed found systematic review |
| 143 | Fresh versus Frozen Embryos for Infertility in the Polycystic Ovary Syndrome Chen et al. | Obstetrics and Gynecology | 8/11/2016 NEJM | It is generally recommended that women with polycystic ovary syndrome be given fresh embryos rather than frozen embryos during in vitro fertilization.^82^ However, it was believed that despite this being common practice, it may come with some increased risks, negatively affecting the receptivity of the endometrium.^83^  This multicenter randomized control trial (n=1508) found that, among infertile women with the polycystic ovary syndrome, women who underwent frozen-embryo transfer experienced a higher rate of live births when compared to women who underwent fresh-embryo transfer (95% confidence interval [CI], 1.05 to 1.31; P=0.004). This is a reversal on fresh embryo transfer in infertile women with the polycystic ovary syndrome. | 2018. “Our results suggest that the IVF/ICSI with [frozen embryo transfer] FET is more efficient and less risky for [ovarian hyperstimulation syndrome] OHSS compared with [fresh embryo transfer] ET. However, we should comprehensively inform patients with advantages, disadvantages and potential risks related to embryo cryopreservation, and carefully assess their fertility conditions to make the most beneficial clinical decision.” ^84^ | Pubmed found systematic review |
| 144 | CPAP for Prevention of Cardiovascular Events in Obstructive Sleep Apnea McEvoy et al. | Cardiovascular disease | 9/8/2016 NEJM | Obstructive sleep apnea is associated with cardiovascular events.^85^ The use of continuous positive airway pressure (CPAP) as a treatment for sleep apnea and as a preventive measure for cardiovascular disease has become popular with the growing observational evidence of the link between the two conditions.^86^ The 2014 American Heart Association/American Stroke Association (AHA/ASA) guidelines for the use of positive airway pressure (PAP) recommend that treatment should be considered for patients with acute ischemic stroke or transient ischemic attack. ^87^This study (n=2717) found that therapy with CPAP plus usual care did not prevent cardiovascular events in patients with moderate-to-obstructive sleep apnea and established cardiovascular disease compared to usual care alone (hazard ratio with CPAP, 1.10; 95% confidence interval, 0.91 to 1.32; P=0.34). This is a reversal of CPAP for prevention of cardiovascular events in obstructive sleep apnea. | 2017. “The use of positive airway pressure (PAP), compared with no treatment or sham, was not associated with reduced risks of cardiovascular outcomes or death for patients with sleep apnea. Although there are other benefits of treatment with PAP for sleep apnea, these findings do not support treatment with PAP with a goal of prevention of these outcomes.” ^88^ | Found in NEJM in "citing articles" section |
| 145 | A Randomized Trial of Long-Term Oxygen for COPD with Moderate Desaturation The Long-Term Oxygen Treatment Trial Research Group et al. | Pulmonary disease | 10/27/2016 NEJM | It has generally been believed that long-term treatment with supplemental oxygen for patients with COPD and hypoxemia reduces mortality. This idea has led to recommendations that patients with hypoxemia receive supplemental oxygen.^89,90^ This randomized clinical trial comparing long-term supplemental oxygen (LTOT) versus no long-term supplemental oxygen in patients with COPD and moderate resting or exercise-induced desaturation found no difference in mortality or time to first hospitalization when comparing groups (hazard ratio, 0.94; 95% confidence interval [CI], 0.79 to 1.12; P=0.52). This was a reversal of the prescription of long-term supplemental oxygen to patients with stable COPD with moderate desaturation. | 2017. “Despite relatively good evidence for the benefits of LTOT in hypoxemic COPD patients in improving survival, similar benefits have not been demonstrated in the majority of COPD patients with moderate or intermittent hypoxemia, either nocturnal or exercise-induced.” ^91^ | Google scholar within citing articles |
| 146 | Five-Year Outcomes after Off-Pump or On-Pump Coronary-Artery Bypass Grafting Lamy et al. | Cardiovascular disease | 12/15/2016 NEJM | Coronary-artery bypass grafting (CABG) can be performed either with a still heart (on-pump CABG) or without a cardiopulmonary bypass on the beating heart (off-pump). Traditionally, surgeons performed surgery on the arrested heart, on-pump CABG, which allowed for increased surgical precision.^92^ However, surgeons grew concerned that the cross clamping of the aorta, necessary for the on-pump CABG procedure, may be harmful to patients and increase mortality and risk of stroke or other systemic embolic events in these patients. The off-pump method, operating on a beating heart, was developed to decrease the perioperative risks.^93^ However, the clinical literature reported different results about the relative efficacy of off-pump CABG as compared with on-pump CABG.^94^ The CORONARY trial (n=4752) compared on-pump and off-pump CABG surgery in patients with coronary heart disease. This follow up study on the results of the CORONARY trial found the after 4 years, patients who underwent on-pump and off-pump CABG has similar rates of outcomes of death, stroke, myocardial infarction, renal failure, and repeat revascularization (23.1% and 23.6%, respectively; hazard ratio with off-pump CABG, 0.98; 95% confidence interval [CI], 0.87 to 1.10; P=0.72). The costs between the two treatments was similar as well. This is a reversal of off-pump coronary-artery bypass grafting. | 2018. “Off-pump CABG increases long-term (≥5 years) mortality compared with on-pump CABG, based on a meta-analysis of eight medium- to large-size RCTs enrolling a total 8780 patients.” ^95^  2012. Cochrane review. "Our systematic review did not demonstrate any significant benefit of off-pump compared with on-pump CABG regarding mortality, stroke, or myocardial infarction. In contrast, we observed better long-term survival in the group of patients undergoing on-pump CABG with the use of cardiopulmonary bypass and cardioplegic arrest. Based on the current evidence, on-pump CABG should continue to be the standard surgical treatment. However, off-pump CABG may be acceptable when there are contraindications for cannulation of the aorta and cardiopulmonary bypass. Further randomised clinical trials should address the optimal treatment in such patients."^96^ This review precedes study. | NEJM within citing articles |
| 147 | Randomized Trial of Bilateral versus Single Internal-Thoracic-Artery Grafts Taggart et al. | Cardiovascular disease | 12/29/2016 NEJM | Single internal-thoracic-artery graft in CABG is highly successful, with a 10-year rate of angiographic patency over 90%.^97^ Single internal-thoracic-artery grafts are standard of care, but its success has led some to perform bilateral internal-thoracic-artery grafts in hopes to further improve outcomes.^98,99^ Observational studies have shown fewer deaths in bilateral versus single internal-thoracic-artery grafts, although the practice has not been widely practiced due to the difficulty of the procedure and the greater risks of complications. This study (n=3102) found that at 5 years, there were no significant differences in rates of mortality and rates of cardiovascular events between patients receiving bilateral versus single internal-thoracic-artery grafts. At 5 years of follow-up, the rate of death was 8.7% in the bilateral-graft group and 8.4% in the single-graft group (hazard ratio, 1.04; 95% confidence interval [CI], 0.81 to 1.32; P=0.77), and the rate of the composite of death from any cause, myocardial infarction, or stroke was 12.2% and 12.7%, respectively (hazard ratio, 0.96; 95% CI, 0.79 to 1.17; P=0.69). There were more sternal wound complications with the bilateral grafting treatment group. This is a reversal of bilateral internal-thoracic-artery grafting. | 2018. “The present meta‐analysis challenges the benefit traditionally attributed to [bilateral single internal thoracic artery] BITA grafting. The fact that, even in the [propensity‐score–matched] PSM series, BITA patients exhibit a significant survival advantage at 1‐year follow‐up suggests that unmeasured confounders may account for the reported survival benefit of BITA in the observational series.”^100^ | PubMed within cited by articles |
| 148 | Randomized Trial of Peanut Consumption in Infants at Risk for Peanut Allergy Du Toit et al. | Allergy and Immunology | 02/26/2015  NEJM | Peanut allergies are relatively uncommon, affecting less than 1% of the population, but can lead to serious outcomes, such as anaphylaxis.^101^ Unlike other food allergies, most children do not become less sensitive as they grow older.^101^ At the time of this study, the recommendations were to withhold peanut products from children until at least 36 months of age.^102^ In this trial (n=640), early exposure to peanut products led to a decrease in peanut allergies in infants and children at high risk of allergy (the prevalence of peanut allergy was 35.3% in the avoidance group and 10.6% in the consumption group (P=0.004)). This is a reversal of peanut avoidance in infants at risk for peanut allergy. | 2016. “In this systematic review, early egg or peanut introduction to the infant diet was associated with lower risk of developing egg or peanut allergy.”^103^ | Google Scholar |
| 149 | Trial of Early, Goal-Directed Resuscitation for Septic Shock Mouncey et al. | Critical care medicine | 4/2/2015 | International guidelines have been developed to direct management of patients presenting with early septic shock. These guidelines state that hospital stays and mortality will be reduced if patients are treated with 6 hours of early, goal-directed therapy (EGDT).^104^ However, the evidence to support these guidelines was limited. ProMISe a randomized control trial (n=1260) in 56 hospitals in England found that all-cause mortality after 90 days did not improve in patients treated with EGDT compared with usual care (relative risk in the EGDT group, 1.01; 95% confidence interval [CI], 0.85 to 1.20; P=0.90). This is a reversal of early, goal-directed resuscitation for septic shock. | 2017. “This systematic review and meta-analysis involving the available RCTs on the impact of EGDT on hospital or ICU mortality for patients with sepsis and septic shock did not show a significant reduced risk of hospital/ICU all-cause mortality associated with the use of EGDT.” ^105^ | PubMed within citing articles |
| 150 | Randomized Trial of Primary PCI with or without Routine Manual Thrombectomy Jolly et al. | Cardiovascular disease | 4/09/2015 NEJM | Primary percutaneous coronary intervention (PCI) for ST-segment elevation myocardial infarction (STEMI) is an effective procedure, but adds a risk of distal embolization of thrombus and failure to restore flow in the microvasculature.^106^ Manual thrombectomy before stent deployment may prevent distal embolization and improve microvascular reperfusion and small randomized trials showed improved outcomes with the addition of thrombectomy. Therefore, practice guidelines were updated to recommend routine thrombectomy with PCI for patients with STEMI.^107^ Meta analyses found that thrombectomies may increase risk of stroke, although the results were not fully conclusive due to limited data.^108^ The TOTAL trial (n=10,732) investigated the effects of PCI with manual thrombectomy on risk of death and stroke. The study found that, compared to PCI alone, PCI with manual thrombectomy did not reduce rates of cardiovascular death, recurrent myocardial infarction, cardiogenic shock, or NYHA class IV heart failure within 180 days (hazard ratio in the thrombectomy group, 0.99; 95% confidence interval [CI], 0.85 to 1.15; P=0.86). Manual thrombectomy was also associated with an increased rate of stroke within 30 days (hazard ratio, 2.06; 95% CI, 1.13 to 3.75; P=0.02). This is a reversal of routine manual thrombectomy in patients with STEMI who are undergoing primary PCI. | 2016. “Moderate certainty evidence suggests aspiration thrombectomy is associated with a possible small decrease in mortality (4 less deaths/1000 over 6 months) and a small increase in stroke (3 more strokes/1000 over 6 months). Because absolute effects are very small and closely balanced, thrombectomy prior to primary PCI should not be used as a routine strategy.” ^109^ The TOTAL trial is the largest and most recent study that was included in this systematic review and meta-analysis.  2015. “Using the totality of evidence available through 2015, this meta-analysis failed to show that the routine use of aspiration thrombectomy in patients with ST-elevation myocardial infarction significantly reduces all-cause mortality, MACE, recurrent MI, TVR, or stent thrombosis. The role of aspiration thrombectomy in selected patients with angiographic evidence of large thrombus burden requires further clinical investigation.”^110^ | Pubmed found systematic review |
| 151 | Prednisolone or Pentoxifylline for Alcoholic Hepatitis Thursz et al. | Gastroenterology/Hepatology | 4/23/2015 NEJM | Despite the prevalence and severity of alcoholic hepatitis, there is no standard therapy. Only two drugs are recommended by guidelines in the United States and Europe - pentoxifylline and prednisolone,^111,112^ yet their effect on reducing mortality is uncertain. STOPAH is a 2-by-2 factorial designed study (n=1103) found no improvement in 28-day mortality among those who used pentoxifylline compared to placebo. Prednisolone had an association with a reduction in 28-day mortality compared to placebo that did not reach significance. The odds ratio for 28-day mortality with pentoxifylline was 1.07 (95% confidence interval [CI], 0.77 to 1.49; P=0.69), and that with prednisolone was 0.72 (95% CI, 0.52 to 1.01; P=0.06). This is a reversal of pentoxifylline for alcoholic hepatitis. | 2017. Cochrane review. “Because of very low-quality evidence, there is uncertainty in the effectiveness of any pharmacological intervention versus no intervention in people with alcoholic hepatitis or severe alcoholic hepatitis.”^113^ | Pubmed found systematic review |
| 152 | Approaches to Catheter Ablation for Persistent Atrial Fibrillation Verma et al. | Cardiovascular disease | 5/7/2015 NEJM | Catheter ablation has shown to be successful in treating paroxysmal atrial fibrillation, but is less effective at treating persistent atrial fibrillation. To increase efficacy in persistent atrial fibrillation, guidelines suggest that extensive ablations based on linear lesions or complex fractionated electrograms be considered,^114^ although there is a lack of research to support the suggestion. The STAR AF II trial (n=589) found that the addition of either linear ablation or ablation of complex fractionated electrograms to pulmonary-vein ablation did not reduce the rate of recurrent atrial fibrillation compared to pulmonary-vein isolation alone (59% for the group receiving isolation alone, 49% for the group receiving isolation plus electrograms, and 46% for the group receiving isolation plus lines; P=0.15 for between-group differences). This is a reversal of linear ablation or ablation of complex fractionated electrograms in addition to pulmonary-vein isolation among patients with persistent atrial fibrillation | 2017. “The recent and much larger CHASE-AF and STAR AF II randomized trials were pivotal in demonstrating that adjunctive [radiofrequency] [RF ablation](https://www.sciencedirect.com/topics/medicine-and-dentistry/radiofrequency-ablation) strategies (lines,[complex fractionated atrial electrograms] CFAEs) did not result in a higher freedom from [arrhythmia](https://www.sciencedirect.com/topics/medicine-and-dentistry/cardiac-dysrhythmia) than a [pulmonary vein isolation] PVI-only strategy using RF but were associated with higher [fluoroscopy](https://www.sciencedirect.com/topics/medicine-and-dentistry/fluoroscopy) and procedure times. Follow-up meta-analyses, which included these 2 studies, now demonstrated no benefit in arrhythmia-free survival with LA linear ablation or CFAE ablation in the [persistent atrial fibrillation] PeAF population.” ^115^ | NEJM citing articles |
| 153 | Therapeutic Hypothermia after Out-of-Hospital Cardiac Arrest in Children Moler et al. | Pediatrics | 5/14/2015 NEJM | Therapeutic hypothermia is recommended for adults with out-of-hospital cardiac arrest and trials on the treatment have shown improvement in neurological outcomes for patients.^116^ Therapeutic hypothermia is practiced in children with cardiac arrest as well,^117^ although research in this population is lacking. This study (n=295) found that therapeutic hypothermia in comatose children who survived out-of-hospital cardiac arrest did not increase survival with a good functional outcome compared to therapeutic normothermia (20% vs. 12%; relative likelihood, 1.54; 95% confidence interval, 0.86 to 2.76; P=0.14). This is a reversal of therapeutic hypothermia after out-of-hospital cardiac arrest in children. | 2015. “Evidence is insufficient to support the advantage of TH compared with normothermia in pediatric resuscitation. The adverse event profile appears to be different than that reported in adults. Further studies are needed before TH may be considered a standard protocol for children after cardiac arrest.” ^118^The Moler et al. study is not included in this review. | Google search for therapeutic hypothermia in children “systematic review” |
| 154 | A Randomized Trial of Intrapartum Fetal ECG ST-Segment Analysis Belfort et al. | Obstetrics and Gynecology | 8/13/2015 NEJM | Fetal electrocardiographic (ECG) ST-segment analysis is approved by the Food and Drug Administration to be used alongside traditional electronic fetal heart-rate monitors to detect ST events. The approval was based on a few studies that found an association between ECG use and reduced rates of neonatal encephalopathy, acidemia, and operative delivery.^119,120^ This multicenter randomized control trial (n=11,108) determined that the addition of fetal ECG ST-segment analysis to standard electronic fetal heart-rate monitors did not significantly improve perinatal outcomes such as intrapartum death, neonatal death, and low Apgar score, nor did it reduce rates of operative delivery (relative risk, 1.31; 95% confidence interval, 0.87 to 1.98; P=0.20). This is a reversal of intrapartum fetal ECG ST-segment analysis used as an adjunct to conventional intrapartum electronic fetal heart rate monitoring. | 2016. “The use of ST analysis during labor as an adjunct to the standard cardiotocography does not improve perinatal outcomes or decrease cesarean delivery.” ^121^ | Pubmed found systematic review |
| 155 | Screening for Occult Cancer in Unprovoked Venous Thromboembolism Carrier et al. | Public health and general preventive medicine | 8/20/2015 NEJM | Venous thromboembolism (VTE) is often provoked (trauma, surgery, or prolonged immobility), but unprovoked thromboembolism may be an early sign of cancer.154 This has been shown in observational studies that found that, among patients with a cancer diagnosis, a thromboembolism event was significantly higher the year prior to their diagnosis than in the general population.155 There are two general approaches to screening for occult cancer in patients who present with venous thromboembolism: basic blood testing or ultrasonography or computer tomography [CT]. Some clinicians recommend limited occult-cancer screening including basic blood testing, history taking, chest radiography, and a physical examination. Others recommend a more robust procedure including the components of the limited screening as well as ultrasonography or [CT] of the abdomen and pelvis.^122,123^ SOME is a multi-center, open-label, randomized, controlled trial (n=854) in Canada found that the additional screening had no significant clinical benefit when compared with limited occult-cancer screening. There was no significant difference between the two study groups in the mean time to a cancer diagnosis (4.2 months in the limited-screening group and 4.0 months in the limited-screening-plus-CT group, P=0.88) or in cancer-related mortality (1.4% and 0.9%, P=0.75). This is a reversal of routine screening with CT of the abdomen and pelvis for occult cancer in unprovoked venous thromboembolism. | 2017. “Occult cancer is detected in 1 in 20 patients within a year of receiving a diagnosis of unprovoked VTE. Older age is associated with a higher cancer prevalence. Although an extensive screening strategy initially may detect more cancer cases than limited screening, whether this translates into improved patient outcomes remains unclear.” ^124^  2017. Cochrane review. "Testing for cancer in people with unprovoked VTE may lead to earlier diagnosis of cancer at an earlier stage of the disease. However, there is currently insufficient evidence to draw definitive conclusions concerning the effectiveness of testing for undiagnosed cancer in people with a first episode of unprovoked VTE (DVT or PE) in reducing cancer and VTE-related morbidity and mortality. The results could be consistent with either benefit or no benefit. Further good-quality large-scale randomised controlled trials are required before firm conclusions can be made."^125^ | Pubmed found systematic review |
| 156 | Letrozole, Gonadotropin, or Clomiphene for Unexplained Infertility Diamond et al. | Obstetrics and Gynecology | 9/24/2015 NEJM | For women with unexplained infertility, empirical ovarian stimulation is an affordable, less invasive option compared to in vitro fertilization. Clomiphene and gonadotropin are ovarian stimulation drugs thought to promote pregnancy by increasing the number of ova ovulated, and possibly by enhancing implantation and placentation,^126^ yet these drugs can cause ovarian hyperstimulation syndrome and multiple gestations which can increase risk of preterm birth and neonatal morbidity. Letrozole, an aromatase inhibitor, has been used off-label for ovarian stimulation and is associated with monofollicular development in many women and has promise as a treatment for unexplained infertility with low risk of ovarian hyperstimulation and multiple births compared to the common ovarian stimulation treatments. This study (n=900) found that women with unexplained fertility treated with letrozole had a lower frequency of multiple gestation but a lower frequency of live births as well, compared to gonadotropin and clomiphene, gonadotropin alone, but not clomiphene alone. After treatment with gonadotropin, clomiphene, or letrozole, clinical pregnancies occurred in 35.5%, 28.3%, and 22.4% of cycles, and live birth in 32.2%, 23.3%, and 18.7%, respectively; pregnancy rates with letrozole were significantly lower than the rates with standard therapy (gonadotropin or clomiphene) (P=0.003) or gonadotropin alone (P<0.001) but not with clomiphene alone (P=0.10). This is a reversal on the use of letrozole for unexplained infertility. | 2016. “Clomiphene may be more effective than letrozole, and treatment with gonadotropins seems more effective, albeit with significantly higher risk of multiple gestations than either oral agent… Adequately powered, randomized controlled trials that compare all of the available treatments for unexplained infertility are needed. Until such data are available, clinicians should individualize the management of unexplained infertility with appropriate counseling regarding the empiric nature of current treatment options including [in vitro fertilization]IVF.”^127^ | Found in NEJM in "citing articles" section |
| 157 | Randomized Trial of Benznidazole for Chronic Chagas’ Cardiomyopathy Morillo et al. | Infections disease | 10/01/2015 NEJM | Chagas' disease is the third most common parasitic disease in the world, caused by *Trypanosoma cruzi*. In its chronic phase, Chagas' leads to cardiac and digestive complications in approximately one third of patients with the disease.^128^ Acute cases of Chagas' disease are successfully treated with trypanocidal drugs, but it is unclear if chronic Chagas' disease is affected by *T. cruzi* in the same way.^129^ The World Health Organization recommended antiparasitic treatment for all chronic-phase *T. cruzi*-infected individuals, despite the lack of substantial evidence.^130^ This study (n=394) investigated the effect of benznidazole, a trypanocidal therapy, in patients with established Chagas' cardiomyopathy on first event of the composite outcome of death, resuscitated cardiac arrest, sustained ventricular tachycardia, insertion of pacemaker or cardioverter-defibrillator, cardiac transplantation, new heart failure, stroke or ischemic attack, or other thromboembolic event. Compared with placebo, benznidazole significantly reduced parasite detection but had no significant effect on cardiac deterioration (95% CI, 0.78 to 1.04; P=0.16 for interaction). This is a reversal of trypanocidal therapy with benznidazole in patients with established Chagas’ cardiomyopathy. | 2018. “Benznidazole had a poor tolerability profle, with a high incidence of [treatment discontinuations] TDs, especially in adult patients and women.” ^131^ | Google scholar within citing articles |
| 158 | Effect of PCI on Long-Term Survival in Patients with Stable Ischemic Heart Disease Sedlis et al. | Cardiovascular disease | 11/12/2015 NEJM | Percutaneous coronary intervention (PCI) is often used to treat patients with stable ischemic heart disease, although PCI has only been shown to increase survival rates in acute ST-segment elevation myocardial infarction.^132^ However, after a 15-year follow up of participants in a randomized control trial (n=1211), it was found that PCI did not improve long-term survival when compared with medical therapy alone (adjusted hazard ratio, 1.03; 95% confidence interval, 0.83 to 1.21; P=0.76). This is a reversal of an initial strategy of PCI plus medical therapy in patients with stable ischemic heart disease. | None found | Effect of PCI on Long-Term Survival in Patients with Stable Ischemic Heart Disease |
| 159 | Acetaminophen for Fever in Critically Ill Patients with Suspected Infection Young et al. | Critical care medicine | 12/03/2015 NEJM | Acetaminophen is routinely administered to critically ill patients in intensive care units to control fever and help recovery.^133^ There are few studies that measure the benefit of acetaminophen administration. HEAT a randomized control trial (n=700) found that there was no difference when comparing placebo and acetaminophen groups in number of ICU-free days. The number of ICU-free days to day 28 did not differ significantly between the acetaminophen group and the placebo group: 23 days (interquartile range, 13 to 25) among patients assigned to acetaminophen and 22 days (interquartile range, 12 to 25) among patients assigned to placebo (Hodges–Lehmann estimate of absolute difference, 0 days; 96.2% confidence interval [CI], 0 to 1; P=0.07). This is a reversal of early administration of acetaminophen to treat fever due to probable infection in patients 16 years old or older with a temperature of 38°C, or higher within 12 hours. | 2017. “Antipyretic treatment does not significantly improve 28-day/hospital mortality in adult patients with sepsis.”^134^ | PubMed within citing articles |
| 160 | Isosorbide Mononitrate in Heart Failure with Preserved Ejection Fraction Redfield et al. | Cardiovascular disease | 12/10/2015 NEJM | Heart failure with preserved ejection fraction (HFpEF) occurs in approximately 50% of heart failure cases. Long acting nitrates have shown to improve activity tolerance in patients with heart failure with a reduced ejection fraction,^135^ yet the effect of nitrates on patients with HFpEF is unclear. Despite the lack of evidence, nitrates are commonly prescribed to patients with HFpEV.^136^ This study (n=110) compared a 6-week dose escalation regimen of isosorbide mononitrate with placebo in patients with HFpEF and found that the treatment group was less active (−439 accelerometer units; 95% CI, −792 to −86; P=0.02) and did not have better QoL or submaximal exercise capacity compared to the placebo group. This is a reversal of giving isosorbide mononitrate to patients with heart failure with preserved ejection fraction of 50% or more. | 2016. “The review including available randomized studies suggests  that nitrates do not confer a mortality benefit in the management  of [acute decompensated heart failure] ADHF [...] There is insufficient data concerning the use of nitrates in female patients, patients with [acute heart failure syndrome] AHFS who have atrial fibrillation, patients with preserved left ventricular systolic function, or patients with nonischemic etiology of heart failure.”^137^ This review does not include RCT because they only included articles published before August of 2015. | Google search for “isosorbide mononitrate in heart failure” “systematic review” |
| 161 | Hypothermia for Intracranial Hypertension after Traumatic Brain Injury Andrews et al. | Critical care medicine | 12/17/2015 NEJM | When people experience traumatic brain injury, hypothermia is often used to improve intracranial hypertension.^138^ Despite its frequent use, there is limited evidence to support this approach. A randomized control trial (n=387) found that when comparing standard care with standard care plus hypothermia, the patients who were treated with therapeutic hypothermia fared worse than those with standard care alone did. Six months after injury, the distribution of GOS-E scores was shifted in an unfavorable direction in the hypothermia group (adjusted common odds ratio, 1.53; 95% confidence interval [CI], 1.02 to 2.30; P=0.04). This is a reversal of therapeutic hypothermia plus standard care to reduce intracranial pressure after a primary, closed traumatic brain injury, an intracranial pressure of more than 20 mmHg for at least 5 minutes, with no obvious reversible cause and an abnormal computer tomographic scan of the brain. | 2017. Cochrane review. “Despite a large number studies, there remains no high-quality evidence that hypothermia is beneficial in the treatment of people with TBI. Further research, which is methodologically robust, is required in this field to establish the effect of hypothermia for people with [traumatic brain injury] TBI.”^139^ | Google scholar search “Hypothermia for traumatic brain injury. Cochrane” |
| 162 | A Trial of Wound Irrigation in the Initial Management of Open Fracture Wounds The Flow Investigators | Surgery | 12/31/2015 NEJM | Open fracture wounds require proper irrigation and removal of infected, damaged tissue to promote healing and prevent infection. The most effective pressure and solution for open fracture wound irrigation is unknown. While low-pressure irrigation is generally recommended, high pressure irrigation may be more effective in removing particulates and bacteria, although the high pressure may damage bone and delay healing.^140^ Castile soap is an affordable, less toxic irrigation solution than antiseptic and antibiotic solutions, and may have added advantage to saline solution alone.^141^ Among some surgeons, high-pressure irrigation with soap was used to treat open wounds, although low pressure, saline irrigation was standard.^142^ This study (n=2551) found that patients with open wounds had similar rates of reoperation regardless of irrigation pressure, and that reoperation rate was higher among wounds irrigated with soap compared to saline. Hazard ratios for the three pairwise comparisons were as follows: for low versus high pressure, 0.92 (95% confidence interval [CI], 0.70 to 1.20; P=0.53), for high versus very low pressure, 1.02 (95% CI, 0.78 to 1.33; P=0.89), and for low versus very low pressure, 0.93 (95% CI, 0.71 to 1.23; P=0.62). This is a reversal of high pressure for wound irrigation in the initial management of open fracture wounds requiring operative fixation. | 2017. Cochrane review. “The evidence base for intracavity lavage and wound irrigation is generally of low certainty… Clinicians should also consider whether the evidence is relevant to the surgical populations under consideration, the varying reporting of other prophylactic antibiotics, and concerns about antibiotic resistance.”^143^ | Pubmed found systematic review |

1.	Mont MA, Jacobs JJ, Boggio LN, et al. Preventing venous thromboembolic disease in patients undergoing elective hip and knee arthroplasty. *JAAOS-Journal of the American Academy of Orthopaedic Surgeons.* 2011;19(12):768-776.

2.	Zee AAG, van Lieshout K, van der Heide M, Janssen L, Janzing HMJ. Low molecular weight heparin for prevention of venous thromboembolism in patients with lower-limb immobilization. *Cochrane Database of Systematic Reviews.* 2017(8).

3.	Van den Berghe G, Wouters P, Weekers F, et al. Intensive Insulin Therapy in Critically Ill Patients. *New England Journal of Medicine.* 2001;345(19):1359-1367.

4.	Vlasselaers D, Milants I, Desmet L, et al. Intensive insulin therapy for patients in paediatric intensive care: a prospective, randomised controlled study. *The Lancet.* 2009;373(9663):547-556.

5.	Hirshberg EL, Sward KA, Faustino EVS, et al. Clinical equipoise regarding glycemic control: a survey of pediatric intensivist perceptions. *Pediatric Critical Care Medicine.* 2013;14(2):123-129.

6.	Zhao Y, Wu Y, Xiang B. Tight glycemic control in critically ill pediatric patients: a meta-analysis and systematic review of randomized controlled trials. *Pediatric Research.* 2018;83:930.

7.	Yamada T, Shojima N, Noma H, Yamauchi T, Kadowaki T. Glycemic control, mortality, and hypoglycemia in critically ill patients: a systematic review and network meta-analysis of randomized controlled trials. *Intensive Care Medicine.* 2017;43(1):1-15.

8.	Haddow JE, Palomaki GE, Allan WC, et al. Maternal Thyroid Deficiency during Pregnancy and Subsequent Neuropsychological Development of the Child. *New England Journal of Medicine.* 1999;341(8):549-555.

9.	Abalovich M, Amino N, Barbour LA, et al. Management of Thyroid Dysfunction during Pregnancy and Postpartum: An Endocrine Society Clinical Practice Guideline. *The Journal of Clinical Endocrinology & Metabolism.* 2007;92(8_supplement):s1-s7.

10.	The American Thyroid Association Taskforce on Thyroid Disease During P, Postpartum, Stagnaro-Green A, et al. Guidelines of the American Thyroid Association for the Diagnosis and Management of Thyroid Disease During Pregnancy and Postpartum. *Thyroid.* 2011;21(10):1081-1125.

11.	Thompson W, Russell G, Baragwanath G, Matthews J, Vaidya B, Thompson‐Coon J. Maternal thyroid hormone insufficiency during pregnancy and risk of neurodevelopmental disorders in offspring: A systematic review and meta‐analysis. *Clinical endocrinology.* 2018.

12.	Deyo RA, Mirza SK. Herniated Lumbar Intervertebral Disk. *New England Journal of Medicine.* 2016;374(18):1763-1772.

13.	Ropper AH, Zafonte RD. Sciatica. *New England Journal of Medicine.* 2015;372(13):1240-1248.

14.	Administration UFaD. LYRICA (pregabalin) 2004; <https://www.accessdata.fda.gov/drugsatfda_docs/label/2012/021446s028lbl.pdf>. Accessed February 12, 2018.

15.	Goodman CW, Brett AS. Gabapentin and Pregabalin for Pain — Is Increased Prescribing a Cause for Concern? *New England Journal of Medicine.* 2017;377(5):411-414.

16.	Enke O, New HA, New CH, et al. Anticonvulsants in the treatment of low back pain and lumbar radicular pain: a systematic review and meta-analysis. *Canadian Medical Association Journal.* 2018;190(26):E786-E793.

17.	Mebazaa A, Pitsis AA, Rudiger A, et al. Clinical review: Practical recommendations on the management of perioperative heart failure in cardiac surgery. *Critical Care.* 2010;14(2):201-201.

18.	Toller W, Algotsson L, Guarracino F, et al. Perioperative Use of Levosimendan: Best Practice in Operative Settings. *Journal of Cardiothoracic and Vascular Anesthesia.* 2013;27(2):361-366.

19.	Landoni G, Mizzi A, Biondi-Zoccai G, et al. Reducing mortality in cardiac surgery with levosimendan: a meta-analysis of randomized controlled trials. *Journal of cardiothoracic and vascular anesthesia.* 2010;24(1):51-57.

20.	Putzu A, Clivio S, Belletti A, Cassina T. Perioperative levosimendan in cardiac surgery: A systematic review with meta-analysis and trial sequential analysis. *International journal of cardiology.* 2018;251:22-31.

21.	Chen Q-H, Zheng R-Q, Lin H, Shao J, Yu J-q, Wang H-L. Effect of levosimendan on prognosis in adult patients undergoing cardiac surgery: a meta-analysis of randomized controlled trials. *Critical Care.* 2017;21(1):253.

22.	Hillis LD, Smith PK, Anderson JL, et al. 2011 ACCF/AHA Guideline for Coronary Artery Bypass Graft Surgery. *A Report of the American College of Cardiology Foundation/American Heart Association Task Force on Practice Guidelines.* 2011;124(23):e652-e735.

23.	Levin R, Degrange M, Del Mazo C, Tanus E, Porcile R. Preoperative levosimendan decreases mortality and the development of low cardiac output in high-risk patients with severe left ventricular dysfunction undergoing coronary artery bypass grafting with cardiopulmonary bypass. *Experimental & Clinical Cardiology.* 2012;17(3):125-130.

24.	Mehta RH, Leimberger JD, van Diepen S, et al. Levosimendan in Patients with Left Ventricular Dysfunction Undergoing Cardiac Surgery. *New England Journal of Medicine.* 2017;376(21):2032-2042.

25.	Wykrzykowska JJ, Kraak RP, Hofma SH, et al. Bioresorbable Scaffolds versus Metallic Stents in Routine PCI. *New England Journal of Medicine.* 2017;376(24):2319-2328.

26.	Wiebe J, Nef HM, Hamm CW. Current Status of Bioresorbable Scaffolds in the Treatment of Coronary Artery Disease. *Journal of the American College of Cardiology.* 2014;64(23):2541-2551.

27.	Zhang X, Zhu Q, Kang L, Li X, Xu B. Mid- and long-term outcome comparisons of everolimus-eluting bioresorbable scaffolds versus everolimus-eluting metallic stents: A systematic review and meta-analysis. *Annals of Internal Medicine.* 2017;167(9):642-654.

28.	Canaris GJ, Manowitz NR, Mayor G, Ridgway E. The colorado thyroid disease prevalence study. *Archives of Internal Medicine.* 2000;160(4):526-534.

29.	Rodondi N, den Elzen WJ, Bauer DC, et al. Subclinical hypothyroidism and the risk of coronary heart disease and mortality. *JAMA.* 2010;304(12):1365-1374.

30.	Baskin HJ, Cobin RH, Duick DS, et al. American association of clinical endocrinologists medical Guidelines for clinical practice for the evaluation and treatment of hyperthyroidism and hypothyroidism: AACE Thyroid Task Force. *Endocrine practice.* 2002;8(6):457-469.

31.	Cleveland JC, Shroyer ALW, Chen AY, Peterson E, Grover FL. Off-pump coronary artery bypass grafting decreases risk-adjusted mortality and morbidity. *The Annals of Thoracic Surgery.* 2001;72(4):1282-1289.

32.	Hattler B, Messenger JC, Shroyer AL, et al. Off-Pump Coronary Artery Bypass Surgery Is Associated With Worse Arterial and Saphenous Vein Graft Patency and Less Effective Revascularization: Results From the Veterans Affairs Randomized On/Off Bypass (ROOBY) Trial

*Circulation.* 2012;125(23):2827-2835.

33.	Gaudino M, Benedetto U, Bakaeen F, et al. Off‐ Versus On‐Pump Coronary Surgery and the Effect of Follow‐Up Length and Surgeons’ Experience: A Meta‐Analysis. *Journal of the American Heart Association.* 2018;7(21):e010034.

34.	Cavender MA. *Anticoagulants in Patients Undergoing PCI: Have New Data Made Heparin the Treatment of Choice?* 2015.

35.	Busko M. PCI Quality Initiative Cuts Bivalirudin Use, Cost, and Bleeding. *Medscape.* 2016. <https://www.medscape.com/viewarticle/863039>.

36.	Rommelfanger J. FDA approves anticoagulant bivalirudin for use in unstable angina. 2000. <https://www.medscape.com/viewarticle/783392>.

37.	Maroko PR, Radvany P, Braunwald E, Hale SL. Reduction of infarct size by oxygen inhalation following acute coronary occlusion. *Circulation.* 1975;52(3):360-368.

38.	Steg PG, James SK, Atar D, et al. ESC Guidelines for the management of acute myocardial infarction in patients presenting with ST-segment elevationThe Task Force on the management of ST-segment elevation acute myocardial infarction of the European Society of Cardiology (ESC). *European Heart Journal.* 2012;33(20):2569-2619.

39.	Roffi M, Patrono C, Collet J-P, et al. 2015 ESC Guidelines for the management of acute coronary syndromes in patients presenting without persistent ST-segment elevationTask Force for the Management of Acute Coronary Syndromes in Patients Presenting without Persistent ST-Segment Elevation of the European Society of Cardiology (ESC). *European Heart Journal.* 2016;37(3):267-315.

40.	Cabello JB, Burls A, Emparanza JI, Bayliss SE, Quinn T. Oxygen therapy for acute myocardial infarction. *Cochrane Database of Systematic Reviews.* 2016(12).

41.	Friedman HS, Prados MD, Wen PY, et al. Bevacizumab Alone and in Combination With Irinotecan in Recurrent Glioblastoma. *Journal of Clinical Oncology.* 2009;27(28):4733-4740.

42.	Cohen MH, Shen YL, Keegan P, Pazdur R. FDA drug approval summary: bevacizumab (Avastin®) as treatment of recurrent glioblastoma multiforme. *The oncologist.* 2009;14(11):1131-1138.

43.	Song J, Xue Y-Q, Zhao M-M, Xu P. Effectiveness of lomustine and bevacizumab in progressive glioblastoma: a meta-analysis. *OncoTargets and therapy.* 2018;11:3435-3439.

44.	Kahn SR, Shbaklo H, Lamping DL, et al. Determinants of health-related quality of life during the 2 years following deep vein thrombosis. *Journal of Thrombosis and Haemostasis.* 2008;6(7):1105-1112.

45.	Hilleman DE, Razavi MK. Clinical and Economic Evaluation of the Trellis-8 Infusion Catheter for Deep Vein Thrombosis. *Journal of Vascular and Interventional Radiology.* 2008;19(3):377-383.

46.	Vedantham S, Grassi CJ, Ferral H, et al. Reporting Standards for Endovascular Treatment of Lower Extremity Deep Vein Thrombosis. *Journal of Vascular and Interventional Radiology.* 2009;20(7, Supplement):S391-S408.

47.	Miller S, Abalos E, Chamillard M, et al. Beyond too little, too late and too much, too soon: a pathway towards evidence-based, respectful maternity care worldwide. *The Lancet.*388(10056):2176-2192.

48.	Organization WH. WHO Safe Childbirth Checklist Implementation Guide: Improving the quality of facility-based delivery for mothers and newborns. 2015.

49.	Kumar S, Yadav V, Balasubramaniam S, et al. Effectiveness of the WHO SCC on improving adherence to essential practices during childbirth, in resource constrained settings. *BMC Pregnancy and Childbirth.* 2016;16:345.

50.	Hochman JS, Sleeper LA, Webb JG, et al. Early revascularization and long-term survival in cardiogenic shock complicating acute myocardial infarction. *JAMA.* 2006;295(21):2511-2515.

51.	Vlaar PJ, Mahmoud KD, Holmes DR, et al. Culprit Vessel Only Versus Multivessel and Staged Percutaneous Coronary Intervention for Multivessel Disease in Patients Presenting With ST-Segment Elevation Myocardial Infarction: A Pairwise and Network Meta-Analysis. *Journal of the American College of Cardiology.* 2011;58(7):692-703.

52.	Tarnow-Mordi W, Morris J, Kirby A, et al. Delayed versus Immediate Cord Clamping in Preterm Infants. *New England Journal of Medicine.* 2017;377(25):2445-2455.

53.	Hill AL, Fontenot HB. Beliefs and practices of obstetric care providers regarding umbilical cord clamping. *Nursing for women's health.* 2014;18(5):413-419.

54.	Fogarty M, Osborn DA, Askie L, et al. Delayed vs early umbilical cord clamping for preterm infants: a systematic review and meta-analysis. *Am J Obstet Gynecol.* 2018;218(1):1-18.

55.	Organization WH. Management of severe malnutrition: a manual for physicians and other senior health workers. 1999.

56.	Perfect JR, Dismukes WE, Dromer F, et al. Clinical Practice Guidelines for the Management of Cryptococcal Disease: 2010 Update by the Infectious Diseases Society of America. *Clinical Infectious Diseases.* 2010;50(3):291-322.

57.	Arabin B, Halbesma JR, Vork F, Hübener M, Eyck Jv. Is treatment with vaginal pessaries an option in patients with a sonographically detected short cervix? *Journal of Perinatal Medicine.* Vol 312003:122.

58.	Saccone G, Ciardulli A, Xodo S, et al. Cervical Pessary for Preventing Preterm Birth in Singleton Pregnancies With Short Cervical Length: A Systematic Review and Meta-analysis. *Journal of Ultrasound in Medicine.* 2017;36(8):1535-1543.

59.	Stockwell JA, Lihinie deAlmeida M. Pediatric Critical Care Nutrition. *Pediatric Critical Care Medicine.* 2015;16(5):e150.

60.	Mehta N. Optimal nutritional therapy for the critically ill child. *Crit Connect.* 2011;10:13.

61.	Cameron DJ, Johnson LB, Maloney EL. Evidence assessments and guideline recommendations in Lyme disease: the clinical management of known tick bites, erythema migrans rashes and persistent disease. *Expert Review of Anti-Infective Therapy.* 2014;12(9):1103-1135.

62.	Du Toit G, Roberts G, Sayre PH, et al. Randomized Trial of Peanut Consumption in Infants at Risk for Peanut Allergy. *New England Journal of Medicine.* 2015;372(9):803-813.

63.	Du Toit G, Roberts G, Sayre PH, et al. Identifying infants at high risk of peanut allergy: The Learning Early About Peanut Allergy (LEAP) screening study. *Journal of Allergy and Clinical Immunology.* 2013;131(1):135-143.e112.

64.	Du Bois M, Szpalski M, Donceel P. A decade’s experience in lumbar spine surgery in Belgium: sickness fund beneficiaries, 2000–2009. *European Spine Journal.* 2012;21(12):2693-2703.

65.	Deyo RA, Mirza SK, Martin BI, Kreuter W, Goodman DC, Jarvik JG. Trends, major medical complications, and charges associated with surgery for lumbar spinal stenosis in older adults. *JAMA.* 2010;303(13):1259-1265.

66.	Dijkerman ML, Overdevest GM, Moojen WA, Vleggeert-Lankamp CLA. Decompression with or without concomitant fusion in lumbar stenosis due to degenerative spondylolisthesis: a systematic review. *European Spine Journal.* 2018;27(7):1629-1643.

67.	Machado GC, Ferreira PH, Yoo RIJ, et al. Surgical options for lumbar spinal stenosis. *Cochrane Database of Systematic Reviews.* 2016(11).

68.	Kudenchuk PJ, Brown SP, Daya M, et al. Amiodarone, Lidocaine, or Placebo in Out-of-Hospital Cardiac Arrest. *New England Journal of Medicine.* 2016;374(18):1711-1722.

69.	Ali MU, Fitzpatrick-Lewis D, Kenny M, et al. Effectiveness of antiarrhythmic drugs for shockable cardiac arrest: A systematic review. *Resuscitation.* 2018;132:63-72.

70.	Hillis LD, Smith PK, Anderson JL, et al. 2011 ACCF/AHA guideline for coronary artery bypass graft surgery: a report of the American College of Cardiology Foundation/American Heart Association Task Force on Practice Guidelines developed in collaboration with the American Association for Thoracic Surgery, Society of Cardiovascular Anesthesiologists, and Society of Thoracic Surgeons. *Journal of the American College of Cardiology.* 2011;58(24):e123-e210.

71.	Association DwtscotEHR, Surgery EbtEAfC-T, Members ATF, et al. Guidelines for the management of atrial fibrillation: the Task Force for the Management of Atrial Fibrillation of the European Society of Cardiology (ESC). *European heart journal.* 2010;31(19):2369-2429.

72.	Putzu A, de Carvalho e Silva CMPD, de Almeida JP, et al. Perioperative statin therapy in cardiac and non-cardiac surgery: a systematic review and meta-analysis of randomized controlled trials. *Annals of Intensive Care.* 2018;8(1):95.

73.	Mayson SE, Greenspon AJ, Adams S, et al. The changing face of postoperative atrial fibrillation prevention: a review of current medical therapy. *Cardiology in review.* 2007;15(5):231-241.

74.	Sethi NJ, Feinberg J, Nielsen EE, Safi S, Gluud C, Jakobsen JC. The effects of rhythm control strategies versus rate control strategies for atrial fibrillation and atrial flutter: A systematic review with meta-analysis and Trial Sequential Analysis. *PloS one.* 2017;12(10):e0186856.

75.	Fattouch K, Guccione F, Sampognaro R, et al. POINT: Efficacy of adding mitral valve restrictive annuloplasty to coronary artery bypass grafting in patients with moderate ischemic mitral valve regurgitation: a randomized trial. *The Journal of Thoracic and Cardiovascular Surgery.* 2009;138(2):278-285.

76.	Narayanan MA, Aggarwal S, Reddy YN, et al. Surgical repair of moderate ischemic mitral regurgitation—a systematic review and meta-analysis. *The Thoracic and cardiovascular surgeon.* 2017;65(06):447-456.

77.	Medicine PCotASfR. Obesity and reproduction: a committee opinion. *Fertility and sterility.* 2015;104(5):1116-1126.

78.	Women's NCCf, Health Cs. *Fertility: assessment and treatment for people with fertility problems.* RCOG Press; 2004.

79.	Wald R, Bagshaw SM. The timing of renal replacement therapy initiation in acute kidney injury: is earlier truly better? *Critical care medicine.* 2014;42(8):1933-1934.

80.	Wang C, Lv L-S, Huang H, et al. Initiation time of renal replacement therapy on patients with acute kidney injury: A systematic review and meta-analysis of 8179 participants. *Nephrology.* 2017;22(1):7-18.

81.	Bhatt GC, Das RR. Early versus late initiation of renal replacement therapy in patients with acute kidney injury-a systematic review & meta-analysis of randomized controlled trials. *BMC Nephrology.* 2017;18(1):78.

82.	Devroey P, Bourgain C, Macklon NS, Fauser BC. Reproductive biology and IVF: ovarian stimulation and endometrial receptivity. *Trends in Endocrinology & Metabolism.* 2004;15(2):84-90.

83.	Bourgain C, Devroey P. The endometrium in stimulated cycles for IVF. *Human Reproduction Update.* 2003;9(6):515-522.

84.	Zhang W, Xiao X, Zhang J, et al. Clinical outcomes of frozen embryo versus fresh embryo transfer following in vitro fertilization: a meta-analysis of randomized controlled trials. *Archives of gynecology and obstetrics.* 2018.

85.	Loke YK, Brown JWL, Kwok CS, Niruban A, Myint PK. Association of obstructive sleep apnea with risk of serious cardiovascular events: a systematic review and meta-analysis. *Circulation: Cardiovascular Quality and Outcomes.* 2012;5(5):720-728.

86.	Lattimore J-DL, Celermajer DS, Wilcox I. Obstructive sleep apnea and cardiovascular disease. *Journal of the American College of Cardiology.* 2003;41(9):1429-1437.

87.	Kernan WN, Ovbiagele B, Black HR, et al. Guidelines for the prevention of stroke in patients with stroke and transient ischemic attack: a guideline for healthcare professionals from the American Heart Association/American Stroke Association. *Stroke.* 2014;45(7):2160-2236.

88.	Yu J, Zhou Z, McEvoy R, et al. Association of positive airway pressure with cardiovascular events and death in adults with sleep apnea: A systematic review and meta-analysis. *JAMA.* 2017;318(2):156-166.

89.	Qaseem A, Wilt TJ, Weinberger SE, et al. Diagnosis and management of stable chronic obstructive pulmonary disease: A clinical practice guideline update from the american college of physicians, american college of chest physicians, american thoracic society, and european respiratory society. *Annals of Internal Medicine.* 2011;155(3):179-191.

90.	LONG TERM DOMICILIARY OXYGEN THERAPY IN CHRONIC HYPOXIC COR PULMONALE COMPLICATING CHRONIC BRONCHITIS AND EMPHYSEMA: Report of the Medical Research Council Working Party. *The Lancet.* 1981;317(8222):681-686.

91.	Ergan B, Nava S. Long-Term Oxygen Therapy in COPD Patients Who Do Not Meet the Actual Recommendations. *COPD: Journal of Chronic Obstructive Pulmonary Disease.* 2017;14(3):351-366.

92.	Shroyer AL, Grover FL, Hattler B, et al. On-Pump versus Off-Pump Coronary-Artery Bypass Surgery. *New England Journal of Medicine.* 2009;361(19):1827-1837.

93.	Grover FL. Current Status of Off-Pump Coronary-Artery Bypass. *New England Journal of Medicine.* 2012;366(16):1541-1543.

94.	Lamy A, Devereaux PJ, Prabhakaran D, et al. Off-Pump or On-Pump Coronary-Artery Bypass Grafting at 30 Days. *New England Journal of Medicine.* 2012;366(16):1489-1497.

95.	Takagi H, Ando T, Mitta S. Meta-Analysis Comparing ≥10-Year Mortality of Off-Pump Versus On-Pump Coronary Artery Bypass Grafting. *The American Journal of Cardiology.* 2017;120(11):1933-1938.

96.	Moller CH, Penninga L, Wetterslev J, Steinbruchel DA, Gluud C. Off-pump versus on-pump coronary artery bypass grafting for ischaemic heart disease. *The Cochrane database of systematic reviews.* 2012(3):Cd007224.

97.	Loop FD, Lytle BW, Cosgrove DM, et al. Influence of the Internal-Mammary-Artery Graft on 10-Year Survival and Other Cardiac Events. *New England Journal of Medicine.* 1986;314(1):1-6.

98.	Lytle BW, Blackstone EH, Loop FD, et al. Two internal thoracic artery grafts are better than one. *The Journal of Thoracic and Cardiovascular Surgery.* 1999;117(5):855-872.

99.	Lytle BW, Loop FD. Superiority of Bilateral Internal Thoracic Artery Grafting. *It’s Been a Long Time Comin’.* 2001;104(18):2152-2154.

100.	Gaudino M, Di Franco A, Rahouma M, et al. Unmeasured Confounders in Observational Studies Comparing Bilateral Versus Single Internal Thoracic Artery for Coronary Artery Bypass Grafting: A Meta-Analysis. *J Am Heart Assoc.* 2018;7(1).

101.	Chapman JA, Bernstein IL, Lee RE, et al. Food allergy: a practice parameter. *Annals of allergy, asthma & immunology.* 2006;96(3):S1-S68.

102.	Laurie Barclay DL. New Guidelines for Introducing Solid Foods to Avoid Development of Infant Allergies. *Medscape.* 2006.

103.	Ierodiakonou D, Garcia-Larsen V, Logan A, et al. Timing of allergenic food introduction to the infant diet and risk of allergic or autoimmune disease: a systematic review and meta-analysis. *Jama.* 2016;316(11):1181-1192.

104.	Rivers E, Nguyen B, Havstad S, et al. Early Goal-Directed Therapy in the Treatment of Severe Sepsis and Septic Shock. *New England Journal of Medicine.* 2001;345(19):1368-1377.

105.	Chen X, Zhu W, Tan J, et al. Early outcome of early-goal directed therapy for patients with sepsis or septic shock: a systematic review and meta-analysis of randomized controlled trials. *Oncotarget.* 2017;8(16):27510-27519.

106.	Buller CE, Fu Y, Mahaffey KW, et al. ST-Segment Recovery and Outcome After Primary Percutaneous Coronary Intervention for ST-Elevation Myocardial Infarction. *Insights From the Assessment of Pexelizumab in Acute Myocardial Infarction (APEX-AMI) Trial.* 2008;118(13):1335-1346.

107.	Kushner FG, Hand M, Smith SC, et al. 2009 Focused Updates: ACC/AHA Guidelines for the Management of Patients With ST‐Elevation Myocardial Infarction (Updating the 2004 Guideline and 2007 Focused Update) and ACC/AHA/SCAI Guidelines on Percutaneous Coronary Intervention (Updating the 2005 Guideline and 2007 Focused Update). *Catheterization and Cardiovascular Interventions.* 2009;74(7).

108.	Tamhane UU, Chetcuti S, Hameed I, Grossman PM, Moscucci M, Gurm HS. Safety and efficacy of thrombectomy in patients undergoing primary percutaneous coronary intervention for Acute ST elevation MI: A Meta-Analysis of Randomized Controlled Trials. *BMC Cardiovascular Disorders.* 2010;10(1):10.

109.	El Dib R, Spencer FA, Suzumura EA, et al. Aspiration thrombectomy prior to percutaneous coronary intervention in ST-elevation myocardial infarction: a systematic review and meta-analysis. *BMC Cardiovascular Disorders.* 2016;16(1):121.

110.	Dominguez AC, Bittl JA, El-Hayek G, Contreras E, Tamis-Holland JE. Meta-analysis of randomized controlled trials comparing percutaneous coronary intervention with aspiration thrombectomy Vs. Conventional percutaneous coronary intervention during ST-segment elevation myocardial infarction. *Catheterization and Cardiovascular Interventions.* 2016;87(7):1203-1210.

111.	European Association for the Study of the L. EASL Clinical Practical Guidelines: Management of Alcoholic Liver Disease. *Journal of Hepatology.* 2012;57(2):399-420.

112.	O'Shea RS, Dasarathy S, McCullough AJ, Practice Guideline Committee of the American Association for the Study of Liver D, the Practice Parameters Committee of the American College of G. Alcoholic liver disease. *Hepatology.* 2010;51(1):307-328.

113.	Buzzetti E, Kalafateli M, Thorburn D, et al. Pharmacological interventions for alcoholic liver disease (alcohol-related liver disease): an attempted network meta-analysis. *Cochrane Database Syst Rev.* 2017;3:Cd011646.

114.	Calkins H, Kuck KH, Cappato R, et al. 2012 HRS/EHRA/ECAS expert consensus statement on catheter and surgical ablation of atrial fibrillation: recommendations for patient selection, procedural techniques, patient management and follow-up, definitions, endpoints, and research trial design. *Journal of Interventional Cardiac Electrophysiology.* 2012;33(2):171-257.

115.	Voskoboinik A, Moskovitch JT, Harel N, Sanders P, Kistler PM, Kalman JM. Revisiting pulmonary vein isolation alone for persistent atrial fibrillation: A systematic review and meta-analysis. *Heart Rhythm.* 2017;14(5):661-667.

116.	Group THaCAS. Mild Therapeutic Hypothermia to Improve the Neurologic Outcome after Cardiac Arrest. *New England Journal of Medicine.* 2002;346(8):549-556.

117.	Doherty DR, Parshuram CS, Gaboury I, et al. Hypothermia Therapy After Pediatric Cardiac Arrest. *Circulation.* 2009;119(11):1492-1500.

118.	Bistritz JF, Horton LM, Smaldone A. Therapeutic hypothermia in children after cardiac arrest: a systematic review and meta-analysis. *Pediatric emergency care.* 2015;31(4):296-303.

119.	Neilson J. Fetal electrocardiogram (ECG) for fetal monitoring during labour. *Cochrane Database Syst Rev.* 2003;2.

120.	Westgate J, Harris M, Curnow JS, Greene KR. Plymouth randomized trial of cardiotocogram only versus ST waveform plus cardiotocogram for intrapartum monitoring in 2400 cases. *American Journal of Obstetrics & Gynecology.* 1993;169(5):1151-1160.

121.	Saccone G, Schuit E, Amer-Wahlin I, Xodo S, Berghella V. Electrocardiogram ST Analysis During Labor: A Systematic Review and Meta-analysis of Randomized Controlled Trials. *Obstetrics and gynecology.* 2016;127(1):127-135.

122.	Cornuz J, Pearson SD, Creager MA, Cook E, Goldman L. IMportance of findings on the initial evaluation for cancer in patients with symptomatic idiopathic deep venous thrombosis. *Annals of Internal Medicine.* 1996;125(10):785-793.

123.	Nordström M, Lindblad B, Anderson H, Bergqvist D, Kjellström T. Deep venous thrombosis and occult malignancy: an epidemiological study. *BMJ : British Medical Journal.* 1994;308(6933):891-894.

124.	van Es N, Le Gal G, Otten H, et al. Screening for occult cancer in patients with unprovoked venous thromboembolism: A systematic review and meta-analysis of individual patient data. *Annals of Internal Medicine.* 2017;167(6):410-417.

125.	Robertson L, Yeoh SE, Stansby G, Agarwal R. Effect of testing for cancer on cancer- and venous thromboembolism (VTE)-related mortality and morbidity in people with unprovoked VTE. *Cochrane Database of Systematic Reviews.* 2017(8).

126.	Fisch P, Casper RF, Brown SE, et al. Unexplained infertility: evaluation of treatment with clomiphene citrate and human chorionic gonadotropin**Supported by the Medical Research Council of Canada and by grants from Ayerst Pharmaceutical Company, and Pharmascience, Montreal, Quebec, Canada. *Fertility and Sterility.* 1989;51(5):828-833.

127.	Gunn DD, Bates GW. Evidence-based approach to unexplained infertility: a&#xa0;systematic review. *Fertility and Sterility.*105(6):1566-1574.e1561.

128.	Rassi Jr A, Rassi A, Marin-Neto JA. Chagas heart disease: pathophysiologic mechanisms, prognostic factors and risk stratification. *Memórias do Instituto Oswaldo Cruz.* 2009;104:152-158.

129.	Marin-Neto JA, Cunha-Neto E, Maciel BC, Simões MV. Pathogenesis of Chronic Chagas Heart Disease. *Circulation.* 2007;115(9):1109-1123.

130.	Viotti R, Alarcón de Noya B, Araujo-Jorge T, et al. Towards a Paradigm Shift in the Treatment of Chronic Chagas Disease. *Antimicrobial Agents and Chemotherapy.* 2014;58(2):635-639.

131.	Crespillo-Andújar C, Venanzi-Rullo E, López-Vélez R, et al. Safety Profile of Benznidazole in the Treatment of Chronic Chagas Disease: Experience of a Referral Centre and Systematic Literature Review with Meta-Analysis. *Drug Safety.* 2018;41(11):1035-1048.

132.	Feldman DN, Gade CL, Slotwiner AJ, et al. Comparison of Outcomes of Percutaneous Coronary Interventions in Patients of Three Age Groups (< 60, 60 to 80, and> 80 Years)(from the New York State Angioplasty Registry†). *American Journal of Cardiology.* 2006;98(10):1334-1339.

133.	Manthous CA, Hall JB, Olson D, et al. Effect of cooling on oxygen consumption in febrile critically ill patients. *American Journal of Respiratory and Critical Care Medicine.* 1995;151(1):10-14.

134.	Drewry AM, Ablordeppey EA, Murray ET, et al. Antipyretic Therapy in Critically Ill Septic Patients: A Systematic Review and Meta-Analysis. *Critical care medicine.* 2017;45(5):806-813.

135.	Leier CV, Huss P, Magorien RD, Unverferth DV. Improved exercise capacity and differing arterial and venous tolerance during chronic isosorbide dinitrate therapy for congestive heart failure. *Circulation.* 1983;67(4):817-822.

136.	Tribouilloy C, Rusinaru D, Mahjoub H, et al. Prognosis of heart failure with preserved ejection fraction: a 5 year prospective population-based study. *European Heart Journal.* 2008;29(3):339-347.

137.	Farag M, Shoaib A, Gorog DA. Nitrates for the Management of Acute Heart Failure Syndromes, A Systematic Review. *Journal of Cardiovascular Pharmacology and Therapeutics.* 2016;22(1):20-27.

138.	Roberts I, Schierhout G, Alderson P. Absence of evidence for the effectiveness of five interventions routinely used in the intensive care management of severe head injury: a systematic review. *Journal of Neurology, Neurosurgery, and Psychiatry.* 1998;65(5):729-733.

139.	Lewis SR, Evans DJW, Butler AR, Schofield-Robinson OJ, Alderson P. Hypothermia for traumatic brain injury. *Cochrane Database of Systematic Reviews.* 2017(9).

140.	Bhandari M, Schemitsch EH, Adili A, Lachowski RJ, Shaughnessy SG. High and low pressure pulsatile lavage of contaminated tibial fractures: an in vitro study of bacterial adherence and bone damage. *Journal of orthopaedic trauma.* 1999;13(8):526-533.

141.	Bhandari M, Adili A, Schemitsch EH. The efficacy of low-pressure lavage with different irrigating solutions to remove adherent bacteria from bone. *JBJS.* 2001;83(3):412.

142.	Petrisor B, Jeray K, Schemitsch E, et al. Fluid lavage in patients with open fracture wounds (FLOW): an international survey of 984 surgeons. *BMC Musculoskeletal Disorders.* 2008;9(1):7.

143.	Norman G, Atkinson RA, Smith TA, et al. Intracavity lavage and wound irrigation for prevention of surgical site infection. *Cochrane Database Syst Rev.* 2017;10:Cd012234.

Total Reversals: 50

| **#** | **Article and Author** | **Primary Medical Discipline** | **Date and Journal** | **Summary** | **Systematic review** | **Systematic Review Search Terms** |
| --- | --- | --- | --- | --- | --- | --- |
| 163 | Stenting and Medical Therapy for Atherosclerotic Renal-Artery Stenosis Cooper et al. | Nephrology | 1/02/2014 NEJM | Since the 1990s, the recommendation for patients who are atherosclerotic with renal-artery stenosis has been to undergo stenting or angioplasty procedures, in the hopes of significantly reducing systolic blood pressure and stabilizing chronic kidney disease.^1^ Since these recommendations, there was a 62% increase in the number of Medicare patients that underwent this procedure between 1996-2000.^2^ However, multiple randomized controlled trials raised questions about the efficacy of treating kidney disease by using a stenting procedure. The CORAL study, an open-label, randomized, control trial (N=5322), set out to test whether treatment of severe renal-artery stenosis and hypertension by stenting combined with medical management conferred patients with a significant prevention of adverse renal and cardiovascular events, over medical management alone. The CORAL study found that stenting combined with medical therapy offered no significant clinical benefit over treatment with medical therapy alone (35.1% and 35.8%, respectively; hazard ratio with stenting, 0.94; 95% confidence interval [CI], 0.76 to 1.17; P=0.58). This is a reversal of stenting for atherosclerotic renal-artery stenosis in patients who have atherosclerotic renal-artery stenosis and either systolic hypertension while taking two or more antihypertensive drugs or chronic kidney disease. | 2014. Cochrane review. “The available data are insufficient to conclude that revascularisation in the form of balloon angioplasty, with or without stenting, is superior to medical therapy for the treatment of atherosclerotic renal artery stenosis in patients with hypertension.”^3^ | Pubmed found systematic review |
| 164 | A Trial of Mass Isoniazid Preventive Therapy for Tuberculosis Control Churchyard et al. | Public health and general preventive medicine | 1/23/2014 NEJM | Individuals living in areas with a high HIV prevalence are at high risk of contracting tuberculosis, as are individuals who work in mining occupations.^4^ Isoniazid, a recommended treatment for tuberculosis prevention, has been suggested by the South African government for workers in the mining industry.^5^ In this trial (N=78,744), mass screening and treatment of tuberculosis in South African gold mine workers did not interrupt the spread of tuberculosis, (rates of 3.02 per 100 person-years in the intervention clusters and 2.95 per 100 person-years in the control clusters (rate ratio in the intervention clusters, 1.00; 95% confidence interval [CI], 0.75 to 1.34; P=0.98; adjusted rate ratio, 0.96; 95% CI, 0.76 to 1.21; P=0.71) This is a reversal of mass isoniazid preventative therapy for tuberculosis control in individuals who work in the mining industry. | No systematic review of individuals at high-risk of contracting tuberculosis who work in mining occupations and were administered prophylactic isoniazid could be found. | GoogleScholar - MA cited RCT |
| 165 | A Randomized Trial of Bevacizumab for Newly Diagnosed Glioblastoma Gilbert et al. | Oncology | 2/20/2014 NEJM | The current approach for managing glioblastoma is with temozolomide and radiotherapy, but 5-year survival remains low.^6^ Bevacizumab, a monoclonal antibody, was being used off-label for treating this cancer, despite the $100,000 a year price tag.^7,8^ In this trial, (n=978) patients newly diagnosed with a glioblastoma were randomized to treatment with bevacizumab or placebo after 4 weeks of radiotherapy. This study found that bevacizumab did not improve overall survival. (Median, 15.7 in the bevacizumab group and 16.1 months in the placebo group; hazard ratio for death in the bevacizumab group, 1.13). This is a reversal of administering bevacizumab to newly diagnosed patients with glioblastoma. | 2014. Cochrane review. "In patients with newly diagnosed GBM [glioblastoma], the use of antiangiogenic therapy does not improve survival, despite evidence of improved progression-free survival."^9^ | PubMed similar articles |
| 166 | Bevacizumab plus Radiotherapy–Temozolomide for Newly Diagnosed Glioblastoma Chinot et al. | Oncology | 2/20/2014 NEJM | The current approach for managing glioblastoma (GBM) is with temozolomide and radiotherapy, but 5-year survival remains low.^6^ Bevacizumab, a monoclonal antibody, was being use off-label for treating this cancer, despite the $100,000 a year price tag.^7,8^ The AVAglio study of (N=978) patients with newly diagnosed glioblastoma randomized patients to bevacizumab or placebo. This study found that bevacizumab did not improve overall survival (stratified hazard ratio for death, 0.88; 95% CI, 0.76 to 1.02; P=0.10). This is a reversal of administering bevacizumab plus radiotherapy to newly diagnosed patients with glioblastoma. | 2014. Cochrane review. "In patients with newly diagnosed GBM, the use of antiangiogenic therapy [bevacizumab, aflibercept, cediranib, angiostatin, endostatin, thrombospondin, cilengitide] does not improve survival, despite evidence of improved progression-free survival.”^9^ | PubMed similar articles |
| 167 | Albumin Replacement in Patients with Severe Sepsis or Septic Shock Caironi et al. | Critical care medicine | 4/10/2014 NEJM | Albumin has been administered to critically ill patients for decades.^10^ However, questions about the safety of this practice have arisen. Since then, there has been conflicting recommendations on how to treat intravascular volume and pressure loss in critically ill patients. The SAFE study, a large randomized control trial, found that patients with severe sepsis had a non-significantly lower risk of death as compared to those patients receiving only saline solution.^11^ Thus, the ALBIOS study (N=1818) sought to determine whether patients with severe sepsis benefited from the treatment of hypoalbuminemia. This study found that the addition of albumin, although safe, did not provide a survival advantage over crystalloid treatment alone (relative risk in the albumin group, 1.00; 95% confidence interval [CI], 0.87 to 1.14; P=0.94). Given the lack of survival advantage and increased cost associated with albumin, this is a reversal of albumin replacement in patients with severe sepsis or septic shock. | 2014. “The present meta-analysis did not demonstrate significant advantage of using albumin-containing fluids for resuscitation in patients with sepsis of any severity. Given the cost-effectiveness of using albumin, crystalloids should be the first choice for fluid resuscitation in septic patients.”^12^ | Pubmed found systematic review |
| 168 | Aspirin in Patients Undergoing Noncardiac Surgery Devereaux et al. | Surgery | 4/17/2014 NEJM | There is evidence that aspirin prevents thromboembolisms after noncardiac surgery but aspirin can also increase the risk of bleeding.^13^Consequently, there is a lack of consensus on whether to start or stop aspirin before surgery. Hospitals and physicians often recommend aspirin for noncardiac surgery to prevent adverse cardiovascular outcomes in patients at high risk.^14^ POISE-2 (N=4998) evaluated if the use of perioperative aspirin reduced the rate of death or nonfatal MI, as compared to placebo. This trial found that aspirin did not significantly decrease the mortality rate. Furthermore, aspirin increased the rate of major bleeding (230 patients [4.6%] in aspirin group vs. 188 patients [3.8%] in placebo group; hazard ratio, 1.23; 95% CI, 1.01, to 1.49; P=0.04) This is a reversal of administering aspirin to patients undergoing noncardiac surgery. | 2018. “Antiplatelet therapy at the time of noncardiac surgery confers minimal bleeding risk with no difference in thrombotic complications. In many cases, it is safe to continue antiplatelet therapy in patients with important indications for their use.”^15^ | Google Scholar cited by articles |
| 169 | A Randomized Trial of Protocol-Based Care for Early Septic Shock The ProCESS Investigators | Critical care medicine | 5/01/2014 NEJM | As a result of one study, which found that early and aggressive intervention (early goal-directed therapy (EGDT) protocol) significantly decreased mortality as compared to standard therapy,^16^ the management of care for sepsis changed drastically.^17^ The authors of ProCESS (N=1341) sought to determine which of the following treatment strategies conferred sepsis patients with the lowest rate of mortality from any cause at 60-days: protocol-based EGDT, protocol-based standard therapy, or usual care. The trial found that there was no significant difference in mortality between septic patients treated with the EGDT protocol, the standard therapy protocol, or usual care. (relative risk with protocol-based therapy vs. usual care, 1.04; 95% confidence interval [CI], 0.82 to 1.31; P=0.83; relative risk with protocol-based EGDT vs. protocol-based standard therapy, 1.15; 95% CI, 0.88 to 1.51; P=0.31). This is a reversal of a protocol-based care for early septic shock in patients in the emergency department in whom sepsis was suspected according to the treating physician, who were at least 18 years of age, who met two or more criteria for systemic inflammatory response syndrome, and who had refractory hypotension or a serum lactate level of 4 mmol per liter or higher. | 2016. “The current meta-analysis pooled data from five RCTs and found no survival benefit of EGDT in patients with sepsis.”^18^  2015."EGDT is not superior to usual care for ED [emergency department] patients with septic shock but is associated with increased utilisation of ICU [intensive care unit] resources."^19^ | Pubmed found systematic review |
| 170 | Clinical Research Network Randomized Trial of Acetylcysteine in Idiopathic Pulmonary Fibrosis Martinez et al. | Pulmonary disease | 5/29/2014 NEJM | The conventional treatment of the chronic progressive lung disease Idiopathic pulmonary fibrosis (IPF) has been glucocorticoids or immunosuppressive agents.^20^ The American Thoracic Society guideline recommends a two-drug regimen of prednisone with either azathioprine or cyclophosphamide.^21^ A 2008 survey of contemporary practice in therapy of idiopathic pulmonary fibrosis found that 50% of pulmonologists used a regimen of either two drugs (azathioprine plus prednisone) or three drugs (azathioprine, prednisone, and N-acetylcysteine [NAC]) to treat mild idiopathic pulmonary fibrosis.^22^ There is no consensus among international guidelines regarding the superiority of the three-drug regimen over the two-drug regimen.^23^ The PANTHER-IPF trial (N=264) assessed whether patients with IPF benefited from treatment measured as the change in forced vital capacity (FVC) (primary outcome) when administered the three-drug regimen against NAC alone (plus placebos for prednisone and azathioprine), as compared with matched placebos for each of the active therapies. The NHLBI stopped the trial and issued a clinical alert for safety concerns on the three-drug regimen. Henceforth, the trial continued recruiting on the NAC-only and placebo groups. PANTHER-IPF found no significant difference between NAC-only and placebo groups in change of FVC at 60 weeks (−0.18 liters and −0.19 liters, respectively; P=0.77). This trial is a reversal of NAC treatment of patients with well-defined IPF. Additionally, this trial provides “compelling evidence against the use of the combination of azathioprine, prednisone, and NAC for patients with idiopathic pulmonary fibrosis who have mild-to-moderate impairment in pulmonary function.”^20^ | 2016. “N-acetylcysteine showed no beneficial effect on changes in forced vital capacity, changes in predicted carbon monoxide diffusing capacity, rates of adverse events, or death rates.”^24^  A SR/MA of the drug combination of azathioprine, prednisone was not found. | Pubmed found systematic review |
| 171 | A Randomized Trial of Epidural Glucocorticoid Injections for Spinal Stenosis Friedly et al. | Neurology/neurosurgery | 7/03/2014 NEJM | The treatment of symptomatic lumbar stenosis has included epidural glucocorticoid injections^.25^ This treatment is frequently prescribed by physicians to treat lumbar stenosis and other conditions, with an estimated 25% of the Medicare population and 74% of patients at the Veteran’s Administration with symptomatic lumbar stenosis being prescribed this treatment. As the usage of glucocorticoid injections increased to treat various ailments, so did the cost. From 1994 to 2001 there was a 271% growth in usage of the treatment, and the cost went from $24 million to over $175 million.^26^ The LESS trial (N=441) was designed to compare the effectiveness of epidural injections of glucocorticoids plus anesthetic vs. injections of anesthetic alone in patients who had lumbar central spinal stenosis and moderate-to-severe leg pain and disability. At six weeks after randomization, there were no significant differences in RMDQ scores (used to measure functional disability), (adjusted difference in the average treatment effect between the glucocorticoid–lidocaine group and the lidocaine-alone group, −1.0 points; 95% confidence interval [CI], −2.1 to 0.1; P=0.07), or pain intensity, (adjusted difference in the average treatment effect, −0.2 points; 95% CI, −0.8 to 0.4; P=0.48), between the patients treated with glucocorticoids plus lidocaine and those in the lidocaine alone group. This is a reversal of administering epidural glucocorticoid injections in patients who have lumbar central spinal stenosis and moderate-to-severe leg pain and disability.. | 2015. “Evidence was limited for epidural corticosteroid injections versus placebo interventions for spinal stenosis ([strength of evidence] SOE: low to moderate) or nonradicular back pain (SOE: low), but showed no differences in pain, function, or likelihood of surgery.”^27^ | Google Scholar within citing articles |
| 172 | Effects of Extended-Release Niacin with Laropiprant in High-Risk Patients  The HPS2-THRIVE Collaborative Group | Cardiovascular disease | 7/17/2014 NEJM | Lowering the risk of major vascular events in patients with cardiovascular disease has centered around lowering LDL cholesterol levels and increasing HDL cholesterol levels. High-dose niacin decreases LDL cholesterol, increases HDL, and lowers triglycerides, lipoprotein levels, and blood pressure.^28^ Hence, guidelines at the time recommend niacin for patients with high cardiovascular risk, and the use of this therapy is increasing in the US.^29^ In 2008, niacin/laropiprant (Tredaptive, Merck) received a positive opinion from the European Committee for Medicinal Products, a recommendation that led to the European Medical Agency (EMEA) approval.^30^ The HPS2-THRIVE trial (N=25,673) assessed whether the addition of extended release niacin and laropiprant, which has been shown to increase adherence to niacin therapy, to statin -based LDL cholesterol-lowerring treatment significantly reduced the incidence of significant vascular events. (13.2% niacin-laropiprant and 13.7% placebo of participants with an event, respectively; rate ratio, 0.96; 95% confidence interval [CI], 0.90 to 1.03; P=0.29) This study found that the addition of niacin-laropiprant to statin-based therapy did not significantly reduce the risk of major vascular events in patients with vascular disease. Moreover, the study found that the addition of niacin-laropiprant led to a significant increase in adverse effects (e.g. excess bleeding, serious infections, myopathy, and serious complications associated with glucose control). It should be noted that when the EMEA approved niacin in 2008, the US FDA issued a “not approvable” letter for the combination tablet of niacin/laropiprant until the results of this HPS2-THRIVE trial had been published.^30^ The agency wanted hard clinical endpoint data before deciding on whether or not to grant approval to the drug. This is a reversal of adding niacin-laropiprant to statin-based therapy in high-risk patients (Men and women 50 to 80 years of age were eligible if they had a history of myocardial infarction, cerebrovascular disease, peripheral arterial disease, or diabetes mellitus with evidence of symptomatic coronary disease). | 2017. Cochrane review. "Moderate- to high-quality evidence suggests that niacin does not reduce mortality, cardiovascular mortality, non-cardiovascular mortality, the number of fatal or non-fatal myocardial infarctions, nor the number of fatal or non-fatal strokes but is associated with side effects. Benefits from niacin therapy in the prevention of cardiovascular disease events are unlikely."^31^ | Pubmed found systematic review |
| 173 | Prednisolone and *Mycobacterium indicus pranii* in Tuberculous Pericarditis Mayosi et al. | Pulmonary disease | 9/18/2014 NEJM | Tuberculous pericarditis— the most common cause of pericarditis in the world—is caused by *Mycobacterium tuberculosis*.^32^ Corticosteroids were often used to treat tuberculous pericarditis in South Africa because of their anti-inflammatory effect – pericarditis being an inflammatory condition.^33^ In fact, current American and World Health Organization guidelines strongly recommend treatment with glucocorticoids in addition to antituberculosis drugs in patients with tuberculous pericarditis.^34^In the IMPI trial (N=1400) patients with definite or probable tuberculous pericarditis received either prednisolone or placebo for 6 weeks and either *M. indicus pranni* or placebo, administered in five injections over the course of 3 months. IMPI found that prednisolone did not have a significant effect on death, cardiac tamponade requiring pericardiocentesis, or constrictive pericarditis in patients with tuberculous pericarditis (primary outcome measures) as compared to placebo(23.8% and 24.5%, respectively; hazard ratio, 0.95; 95% confidence interval [CI], 0.77 to 1.18; P=0.66). Both prednisolone therapy and *M. indicus pranii* immunotherapy increased incidence of HIV- associated cancer among trial participants. This is a reversal of administering prednisolone to HIV positive patients with tuberculous pericarditis. | 2017. Cochrane review. “For HIV-negative patients, corticosteroids may reduce death. For HIV-positive patients not on antiretroviral drugs, corticosteroids may reduce constriction. For HIV-positive patients with good antiretroviral drug viral suppression, clinicians may consider the results from HIV-negative patients more relevant.”^35^  “In people living with HIV and not on antiretroviral drugs, corticosteroids may reduce constrictive pericarditis and hospitalization (low certainty evidence). However, corticosteroids may make little or no difference to the need for repeat pericardiocentesis (low certainty evidence) and it is uncertain whether the intervention has an effect on deaths or any other outcome in HIV-positive people (very low certainty evidence)”^35^ | PubMed similar articles |
| 174 | Outcomes 1 Year after Thrombus Aspiration for Myocardial Infarction Lagerqvist et al. | Cardiovascular disease | 9/18/2014 NEJM | The therapeutic standard of ST-elevation myocardial infarction (STEMI) has been primary PCI;^36^ however, when STEMI patients have been treated with PCI the problem of reperfusion, an injury which leads to myocardial damage, remains.^37^ Thrombus aspiration is a relatively inexpensive and easy procedure that may improve coronary artery flow.^38^ The procedure began to be adopted by some Swedish centers as routine, while other healthcare centers allowed treatment to be at the discretion of the physician. Furthermore, the American College of Cardiology and the American Heart Association recommended the procedure in some cases.^39^ The TASTE trial (N=7244) found that treating STEMI patients with routine manual thrombus aspiration before PCI did not provide a reduction in all-cause mortality, as compared to treating STEMI patients with PCI alone(hazard ratio, 0.94; 95% confidence interval [CI], 0.78 to 1.15; P=0.57). This is a reversal ofc routine manual thrombus aspiration before PCI in patients with acute STEMI. | 2017. “Routine aspiration thrombectomy prior to primary PCI was not associated with a reduction in long‐term mortality or clinical outcomes.” ^40^  2014. “The present meta-analysis suggested that there was no evidence that using manual thrombus aspiration in patients with STEMI could provide distinct benefits in long-term clinical outcomes.”^41^ | Pubmed found systematic review |
| 175 | Ultrasonography versus Computed Tomography for Suspected Nephrolithiasis Smith-Bindman et al. | Urology | 9/18/2014 NEJM | Nephrolithiasis, more commonly known as having a kidney stone, is a painful condition.^42^ It occurs in about 5% of the US population and about half of those with a previous kidney stone will have a recurrence of nephrolithiasis within 10 years.^43^ Recommendations at the time of this study were to make an initial diagnosis of nephrolithiasis by imaging with computed tomography (CT), except for pregnant women, where CT is contraindicated. .^44^ In this trial (N=2759), initial ultrasound diagnosis of nephrolithiasis led to less radiation exposure (P<0.001) and similar high-risk nephrolithiasis detection rates as CT. This is a reversal of initially using a CT for diagnosing nephrolithiasis versus initially using an ultrasound in patients who presented to the emergency department with suspected nephrolithiasis. | No SR/MA found | Initial diagnosis of nephrolithiasis systematic review, meta-analysis |
| 176 | Introduction of Gluten, HLA Status, and the Risk of Celiac Disease in Children Lionetti et al. | Gastroenterology/hepatology | 10/02/2014 NEJM | Celiac disease (CD) is an autoimmune disease that often develops in childhood and can cause developmental delays if left untreated. Many physicians recommend an early introduction to gluten at 4-6 months of age, combined with breastfeeding, as a way of decreasing a child’s risk of developing this food insensitivities.^45^ The practice became known as the “window of opportunity” and gained prevalence because of a Swedish CD epidemic.^46^ However, for children with a familial risk of CD, the recommendation is to delay the introduction of gluten.^47^ The CELIPREV trial (N=707) wanted to determine whether children who had gluten introduction at 12 months of age (closed window) had a higher risk of developing CD, as compared to children who had an early gluten exposure at 4 to 6 months (open window), while taking into consideration high-risk HLA genotype children. The researchers found that delaying the introduction of gluten did not affect the risk of developing CD in the long term,^48^ in other words, the timing of gluten introduction does not seem to influence the development of CD. At 5 years of age, the between-group differences were no longer significant for autoimmunity (21% in open window group and 20% in control group, P=0.59) or overt disease (16% and 16%, P=0.78 by the log-rank test). This is a reversal of the early introduction of gluten to children that are at risk of developing celiac disease (newborns who had a familial risk of celiac disease (i.e., newborns who had at least one first-degree relative with celiac disease)). | 2016. “Currently, there is no evidence on the optimal breastfeeding duration or the effects of avoiding early (<4 months of age) or late (≥ 6 or even at 12 months) gluten introduction in children at risk of CD. Accordingly, no specific general recommendations about gluten introduction or optimal breastfeeding duration can be presently provided on evidence-based criteria in order to prevent CD.”^49^ | Pubmed found systematic review |
| 177 | Randomized Feeding Intervention in Infants at High Risk for Celiac Disease Vriezinga et al. | Gastroenterology/hepatology | 10/02/2014 NEJM | Similar to the study discussed above, PreventCD set out to test whether exposing high-risk children to small amounts of gluten at 16 to 24 weeks while breastfeeding lowered the frequency of celiac disease (CD) at three years of age. The study (N=963) found that the introduction of gluten did not mitigate the risk of CD in high-risk children (5.9% [95% CI, 3.7 to 8.1] and 4.5% [95% CI, 2.5 to 6.5], respectively; hazard ratio in the gluten group, 1.23; 95% CI, 0.79 to 1.91). Additionally, breastfeeding maintenance during the gluten exposure did not have a significant effect on the frequency of CD. This is a reversal of the early introduction of gluten to children that are at risk of developing celiac disease. | 2016. “Currently, there is no evidence on the optimal breastfeeding duration or the effects of avoiding early (<4 months of age) or late (≥ 6 or even at 12 months) gluten introduction in children at risk of CD. Accordingly, no specific general recommendations about gluten introduction or optimal breastfeeding duration can be presently provided on evidence-based criteria in order to prevent CD.”^49^ | PubMed similar articles |
| 178 | Follow-up of Blood-Pressure Lowering and Glucose Control in Type 2 Diabetes Zoungas et al. | Endocrinology, Diabetes, and Metabolism | 10/09/2014 NEJM | Studies of diabetic patients have shown a long-term benefit of intensive glucose control, but not blood pressure lowering on mortality and macrovascular events.^50^ ^51^ ADVANCE-ON (N=8494) is a follow-up study, which examines whether patients with type 2 diabetes and are at high risk of cardiovascular events, benefit in the long-term from tight blood pressure control and tight glucose control (lowering sugar levels to below current guidelines).^50^ In other words, The ADVANCE-ON trial is a ten-year follow-up to the ADVANCE trial, which included patients with type 2 diabetes and high cardiovascular risk. The original results showed that there were significant benefits—a reduction in microvascular or macrovascular events— from tight blood pressure and glucose control. ADVANCE- ON trial found that tight glucose control did not provide a benefit in reducing death from any cause or reducing major macrovascular events. Hence, there were no differences observed during follow-up between the intensive-glucose-control group and the standard-glucose-control group; the hazard ratios were 1.00 (95% CI, 0.92 to 1.08) and 1.00 (95% CI, 0.92 to 1.08), respectively. This is a reversal of tight glucose control in patients with type 2 diabetes who are 55 years of age or older, and at least one additional risk factor for cardiovascular disease. | 2011. “Intensive glycaemic control does not seem to reduce all cause mortality in patients with type 2 diabetes. Data available from randomised clinical trials remain insufficient to prove or refute a relative risk reduction for cardiovascular mortality, non-fatal myocardial infarction, composite microvascular complications, or retinopathy at a magnitude of 10%.”^52^  Does not cite the RCT.  2011. “Tight glucose control did not change the risk of all‐cause or cardiovascular death in type 2 diabetes patients”^53^  Does not cite the RCT. | Google: ADVANCE- ON effect of blood pressure lowering and glucose control in mortality of type 2 diabetes "systematic review" “meta analysis" |
| 179 | Lower versus Higher Hemoglobin Threshold for Transfusion in Septic Shock Holst et al. | Critical care medicine | 10/09/2014 NEJM | Septic shock patients undergo blood transfusions frequently, whether they are bleeding or nonbleeding, per recommendations from the Surviving Sepsis Campaign, which indicates that patients with septic shock should undergo blood transfusions to maintain a hematocrit of more than 30% in the presence of hypoperfusion in the first 6 hours. Thereafter, the transfusion threshold should be a hemoglobin level of less than 7g/dL..^54^ Despite the guidelines, there is variation in practice among physicians, and new data has pointed at safety concerns regarding this practice. Cohort studies have found that some critically ill patients do not benefit from blood transfusions,^55,56^ and yet other studies indicate that sepsis patients benefit from receiving transfusions.^57^ The TRISS trial (N=998) set out to evaluate the effects on mortality of leukoreduced blood transfusions at a lower versus higher hemoglobin threshold among patients with septic shock. The trial found that patients with septic shock who underwent fewer transfusions with a lower level of hemoglobin (7g/dL), had a similar rate of mortality as patients with a higher hemoglobin threshold (9g/dL)(relative risk, 0.94; 95% confidence interval, 0.78 to 1.09; P=0.44). This is a reversal of frequent transfusions and high hemoglobin targets in patients with septic shock 18 years of age or older who were in the ICU, fulfilled the criteria for septic shock, and had a blood concentration of hemoglobin of 9 g per deciliter or less as measured by means of valid point-of-care testing. | 2016. Cochrane review. “There was no evidence that a restrictive transfusion strategy impacts 30-day mortality or morbidity (i.e. mortality at other points, cardiac events, myocardial infarction, stroke, pneumonia, thromboembolism, infection) compared with a liberal transfusion strategy.”^58^ | PubMed articles cited by |
| 180 | Goal-Directed Resuscitation for Patients with Early Septic Shock The ARISE Investigators and the ANZICS Clinical Trials Group | Critical care medicine | 10/16/2014 NEJM | Early goal-directed therapy (EGDT) was adopted into the Surviving Sepsis Campaign guidelines because a 2001 randomized trial showed that patients with sepsis benefited from the six-hour protocol. However, uncertainty remained about the implementation of EGDT.^59^ The ARISE study (N=1600) found that treating critically ill patients with EGDT led to no significant reduction in all-cause mortality. At 90 days after randomization, 147 deaths had occurred in the EGDT group and 150 had occurred in the usual-care group, for rates of death of 18.6% and 18.8%, respectively (absolute risk difference with EGDT vs. usual care, −0.3 percentage points; 95% confidence interval, −4.1 to 3.6; P=0.90). This is a reversal of goal directed resuscitation for patients presenting to the emergency department with early septic shock. | 2016. “The current meta-analysis pooled data from five RCTs and found no survival benefit of EGDT in patients with sepsis.”^18^  2017. “EGDT is not superior to usual care for ED [Emergency Department] patients with septic shock but is associated with increased utilization of ICU resources.” ^19^ | GoogleScholar - MA cited RCT |
| 181 | Early versus On-Demand Nasoenteric Tube Feeding in Acute Pancreatitis Bakker et al. | Gastroenterology/Hepatology | 11/20/2014 NEJM | Patients with acute pancreatitis (AP) often need nutritional support. The risk of infection and mortality increases in patients with AP. It has been established that enteral tube feeding (EN) is superior to total parenteral nutrition (TPN) since EN is associated with a decrease in infections and complications as compared to TPN.^60^ American and European nutritional societies recommend routine early EN in all patients with severe pancreatitis.^61^ On the other hand, pancreatic and gastroenterology guidelines state that EN should be initiated if patients are not able to tolerate an oral diet for up to 7 days.^62^ To address the disparity in the timing of EN feeding (early or after oral nutritional failure) in patients with severe AP, the DAPSG trial was designed to determine whether early EN significantly reduced infectious complications and mortality as compared to delayed EN in patients with severe AP. The DAPSG trial (N=208) found that contrary to most guidelines, early EN does not significantly reduce the rate of infections or mortality. The primary end point occurred in 30 of 101 patients (30%) in the early group and in 28 of 104 (27%) in the on-demand group (risk ratio, 1.07; 95% confidence interval, 0.79 to 1.44; P=0.76). This is a reversal of early nasoenteric tube feeding of patients with acute pancreatitis who were at high risk for complications on the basis of an Acute Physiology and Chronic Health Evaluation II score of 8 or higher (on a scale of 0 to 71, with higher scores indicating more severe disease), an Imrie or modified Glasgow score of 3 or higher (on a scale of 0 to 8, with higher scores indicating more severe disease), or a serum C-reactive protein level of more than 150 mg per liter. | 2017. “Questions persist about feeding in patients with pancreatitis. First, the implications of these findings for patients with severe pancreatitis remain uncertain. Although none of the 4 studies of such patients found an increase in adverse events with early feeding, only 1 showed benefit. This study found reduced length of stay with early feeding; however, it did not report mortality and had methodological flaws that limit the value of its conclusions. Given these limitations, the utility of early feeding in this population remains to be seen…  Limited data suggest that early feeding in patients with acute pancreatitis does not seem to increase adverse events and, for patients with mild to moderate pancreatitis, may reduce length of hospital stay.”^63^ | PubMed similar articles |
| 182 | Surgical Treatment of Moderate Ischemic Mitral Regurgitation Smith et al. | Cardiovascular disease | 12/04/2014 NEJM | Ischemic mitral regurgitation is a common result of myocardial infarction (MI) and is associated with higher rates of mortality.^64^ Concomitant mitral-valve repair is sometimes added to coronary-artery bypass surgery (CABG) for patients with MI to treat ischemic mitral regurgitation, but the benefits of the procedure are unclear. Those that support the addition of mitral-valve repair alongside CABG believe that the additional procedure will improve cardiac function and reduce the risk of heart failure beyond CABG alone by preventing adverse remodeling in the valve.^65^ However, CABG with concomitant valve repair is a considerably longer procedure that is associated with a higher perioperative risk.^66^ This trial set out to determine whether patients with moderate ischemic mitral regurgitation treated with CABG and mitral-valve repair had a greater degree of left ventricular reverse remodeling after one year, compared to those patients that underwent CABG surgery alone. The trial (N=301) did not show a clinically meaningful advantage of adding mitral-valve repair to CABG after one year. There was no significant difference between the rate of mortality, adverse cardiac or cerebrovascular events, and readmissions to the hospital or improved quality of life between the two groups hazard ratio with mitral-valve repair, 0.90; 95% confidence interval, 0.38 to 2.12; P=0.81). Moreover, the combined procedure resulted in longer stays in the ICU and a higher rate of supraventricular arrhythmias. This is a reversal of CABG with concomitant valve repair in treatment of patients with multivessel coronary artery disease and moderate ischemic mitral regurgitation. | 2016. “There is neither increased operative mortality nor survival benefit associated with concomitant CABG and MV [mitral valve] repair for IMR [ischemic mitral regurgitation] of moderate degree over CABG alone.”^67^ | PubMed similar articles |
| 183 | High-Frequency Oscillation for Acute Respiratory Distress Syndrome Young et al. | Critical care medicine | 2/28/2013 NEJM | High-frequency oscillatory ventilation (HFOV) is commonly used in patients with acute respiratory distress syndrome (ARDS). However, data on the efficacy of this treatment are conflicting.^68^ Similarly to OSCILLATE, the OSCAR study (N=795) wanted to compare HFOV to usual ventilatory care. They found that there was no significant difference in reducing the 30-day all-cause mortality between HFOV and usual ventilatory care (P=0.85). This is a reversal of HFOV in treatment of early acute respiratory distress syndrome. | 2016. Cochrane review. "The findings of this systematic review suggest that HFO does not reduce hospital and 30-day mortality due to ARDS; the quality of evidence was very low. Our findings do not support the use of HFO as a first-line strategy in people undergoing mechanical ventilation for ARDS."^69^ | PubMed similar articles |
| 184 | High-Frequency Oscillation in Early Acute Respiratory Distress Syndrome Ferguson et al. | Critical care medicine | 2/28/2013 NEJM | High-frequency oscillatory ventilation (HFOV) is commonly used in patients with acute respiratory distress syndrome (ARDS).^70^ However, data on the efficacy of this treatment are conflicting.^68^ As a result, OSCILLATE (N=548) wanted to compare HFOV with a conventional ventilation strategy in patients with moderate to severe ARDS. The study was stopped early because of a significantly higher rate of mortality among patients receiving HFOV compared to patients receiving a ventilation strategy that used small tidal volumes and high levels of PEEP. Thus, this study found that not only is HFOV not effective at reducing mortality in patients with ARDS, it may also be harmful. In-hospital mortality was 47% in the HFOV group, as compared with 35% in the control group (relative risk of death with HFOV, 1.33; 95% confidence interval, 1.09 to 1.64; P=0.005). This is a reversal of HFOV in treatment of adults with moderate-to-severe early acute respiratory distress syndrome. | 2016. Cochrane review. "The findings of this systematic review suggest that HFO does not reduce hospital and 30-day mortality due to ARDS; the quality of evidence was very low. Our findings do not support the use of HFO as a first-line strategy in people undergoing mechanical ventilation for ARDS."^69^ | PubMed similar articles |
| 185 | A Trial of Imaging Selection and Endovascular Treatment for Ischemic Stroke Kidwell et al. | Cardiovascular disease | 3/07/2013 NEJM | Intravenous tissue plasminogen activator (tPA) is a medication proven to be effective in restoring blood flow after acute ischemic stroke, although its success is heavily time-dependent.^71^ Unfortunately, fewer than 10% of patients are eligible for t-PA because there is a 4.5 hour time window to receive the medication.^72^ Endovascular therapy (ET) is therefore commonly used in patients ischemic stroke to recanalize occluded arteries.^73^ One endovascular therapy device, the concentric thrombus retriever catheter, has received FDA approval and according to the authors, "The device is currently approved by the FDA for the indication of foreign body retrieval and, at some centers, is being increasingly employed off-label at physician discretion to treat ischemic stroke patients."^74^ Although these therapies became widely used, evidence to assess their efficacy and safety is needed. The MR RESCUE study (N=118) found that penumbral imaging did not identify patients who would benefit from endovascular therapy and, similar to the IMS and SYNTHESIS studies, found no effect on relevant clinical endpoints of treatment with endovascular devices over standard tPA therapy. Among all patients, mean scores on the modified Rankin scale did not differ between embolectomy and standard care (3.9 vs. 3.9, P=0.99). This is a reversal of using penumbral imaging to identify patients between the ages of 18 and 85 years with National Institutes of Health Stroke Scale (NIHSS) scores of 6 to 29 who had a large-vessel, anterior-circulation ischemic stroke 8 hours after the onset of symptoms who would benefit from endovascular treatment for ischemic stroke. | 2013. "We found that ET is not superior to IV tPA in improving mortality or functional outcome at 3 months (level B recommendation."^75^ | GoogleScholar - MA cited RCT |
| 186 | Endovascular Treatment for Acute Ischemic Stroke  Ciccone et al. | Neurology/neurosurgery | 3/07/2013 NEJM | Intravenous tissue plasminogen activator (tPA) is a medication proven to be effective in restoring blood flow after acute ischemic stroke, although its success is heavily time-dependent.^71^ Unfortunately, fewer than 10% of patients are eligible for t-PA because there is a 4.5 hour time window to receive the medication.^72^ Endovascular therapy is therefore commonly used in patients ischemic stroke to recanalize occluded arteries,^73^ although the therapy often delays initial treatment and does not have randomized controlled studies to support its use over tPA. The SYNTHESIS Expansion trial (N=362) wanted to examine the clinical effectiveness of endovascular treatment as compared to intravenous t-PA, in patients with acute stroke in whom intracranial hemorrhage had been ruled out and were evaluated within 4.5 hours after symptom onset. The results of this trial also support the findings of the IMS III trial—endovascular therapy is not superior to treatment with intravenous tPA alone in terms of survival free of disability at 3 months (odds ratio adjusted for age, sex, stroke severity, and atrial fibrillation status at baseline, 0.71; 95% confidence interval, 0.44 to 1.14; P=0.16). This is a reversal of endovascular therapy for treatment of acute ischemic stroke for patients with acute stroke and an age of 18 to 80 years, in whom intracranial hemorrhage had been ruled out, and a clearly defined time of stroke onset. | 2013. “Formal meta-analysis indicates that there are similar safety outcomes and functional independence with endovascular therapy and intravenous thrombolysis for acute ischemic stroke.”^76^ | PuBMed cited by articles |
| 187 | Endovascular Therapy after Intravenous t-PA versus t-PA Alone for Stroke Broderick et al. | Neurology/neurosurgery | 3/07/2013 NEJM | Intravenous tissue plasminogen activator (tPA) is a medication proven to be effective in restoring blood flow after acute ischemic stroke, although its success is heavily time-dependent.^71^ There is a 4.5 hour time window to receive the medication, and so, unfortunately, fewer than 10% of patients are eligible for tPA.^72^ Endovascular therapy is therefore commonly used in patients with ischemic stroke to recanalize occluded arteries,^73^ although the therapy often delays initial treatment and does not have randomized controlled studies to support its use. In the Interventional Management of Stroke (IMS) III trial (N=656), patients were treated with tPA within 3 hours of stroke onset and followed with either more tPA or endovascular treatment. IMS III was terminated early because of futility. It showed similar safety outcomes but no better improvement in functional outcomes among those treated with endovascular therapy in addition to tPA. The proportion of participants with a modified Rankin score of 2 or less at 90 days did not differ significantly according to treatment (40.8% with endovascular therapy and 38.7% with intravenous tPA; absolute adjusted difference, 1.5 percentage points; 95% confidence interval [CI], −6.1 to 9.1, with adjustment for the National Institutes of Health Stroke Scale [NIHSS] score [8–19, indicating moderately severe stroke, or ≥20, indicating severe stroke]). This is a reversal of endovascular therapy after intravenous tPA for stroke for patients who had received intravenous t-PA within 3 hours after symptom onset. | 2013. “Formal meta-analysis indicates that there are similar safety outcomes and functional independence with endovascular therapy and intravenous thrombolysis for acute ischemic stroke.”^76^ | Pubmed suggestion |
| 188 | Percutaneous Closure of Patent Foramen Ovale in Cryptogenic Embolism Meier et al. | Cardiovascular disease | 3/21/2013 NEJM | Observational studies have indicated that a viable treatment to reduce the risk of recurrent adverse events in patients with a paradoxical embolism in the presence of a patent foramen ovale (PFO) can be the percutaneous closure of the PFO, in addition to standard prophylactic therapy.^77^ Conversely, several analyses and guidelines suggest that PFO closure might be inferior to standard treatment.^78,79^ The question of the benefit of PFO closure has been posed several times during the last 20 years and several randomized controlled trials have attempted to shed some clarity on this contentious issue. Shatzel et al. summarize the findings of these studies, Closure I 2012, PC 2013, and Respect 2013 had a composite endpoint that included mortality and they found that PFO closure did not decrease deaths.^80^ On the other hand, recent studies –  Gore REDUCE 2017, CLOSE 2017 and RESPECT extended f/u 2017—  focusing of large interatrial shunts or ASA found that PFO closure decrease rates of stroke.^80^ Here we report on the findings of the PC trial, designed to establish a cause and effect relationship between PFO treatment and clinically significant risk reductions of adverse embolic events in patients less than 60 years old with PFO and ischemic stroke, transient ischemic attack (TIA), or a peripheral thromboembolic event. The PC-trial (N=414) examined whether PFO closure using the Amplatzer PFO Occluder device (AGA Medical Corporation, Golden Valley, MN, USA) along with medical prophylactic therapy, was superior in reducing the risk of death or repeated embolic events. This 4-year multinational trial found that PFO closure with the device was not superior to standard treatment alone. The primary end point, a composite of death, nonfatal stroke, TIA, or peripheral embolism, occurred in 7 of the 204 patients (3.4%) in the closure group and in 11 of the 210 patients (5.2%) in the medical-therapy group (hazard ratio for closure vs. medical therapy, 0.63; 95% confidence interval [CI], 0.24 to 1.62; P=0.34). This is a reversal of percutaneous closure of PFO in patients with cryptogenic embolism less than 60 years old with a documented PFO by transesophageal echocardiography and no other identifiable cause of stroke or peripheral thromboembolism. We believe that this is a reversal because at the time of this study, 2013, the available evidence indicated that PFO closure was not effective. | 2018. “Anticoagulation, compared with either PFO closure plus antiplatelet therapy or antiplatelet therapy alone probably increases the risk of major bleeding by approximately 2% over 5 years and probably reduces the risk of pulmonary embolism by approximately 0.4%. There does not appear to be an important difference in the risk of death or in the risk of systemic emboli between any of the interventions.” ^81^  2018. “Surprisingly, the first 3 RCTs (CLOSURE I, PC, and  RESPECT) in this field failed individually to show that  PFO closure was superior to medical therapy alone… In patients with PFO and cryptogenic stroke, transcatheter device closure decreases risk for recurrent stroke compared with medical therapy alone. Because recurrent stroke rates are low even with medical therapy alone and PFO closure might affect AF risk, shared decision making is crucial for this treatment.” ^82^ | PubMed in cited by articles |
| 189 | Closure of Patent Foramen Ovale versus Medical Therapy after Cryptogenic Stroke Carroll et al. | Cardiovascular disease | 3/21/2013 NEJM | Guidelines from the European Society of Cardiology,^83^ American College of Chest Physicians^84^ and the National Institute for Health and Care Excellence recommend that for patients with cryptogenic stroke and Patent Foramen Ovale (PFO) recommend a consideration of percutaneous closure of the PFO.^85^ The RESPECT trial (N=980), similar to the PC trial, evaluated whether patients between 18-60 years of age with a history of cryptogenic ischemic stroke and a patent foramen ovale (PFO), would benefit from the closure of the PFO with the Amplatzer PFO Occluder device, as compared to medical therapy alone. The trial found PFO closure with the Amplatzer PFO Occluder device was not superior to prophylactic standard medical therapy. In the intention-to-treat cohort, 9 patients in the closure group and 16 in the medical-therapy group had a recurrence of stroke (hazard ratio with closure, 0.49; 95% confidence interval [CI], 0.22 to 1.11; P=0.08). This is a reversal of closure of the PFO with the Amplatzer PFO Occluder device after cryptogenic stroke in patients 8 and 60 years of age, had had a cryptogenic ischemic stroke, and had a patent foramen ovale identified by means of transesophageal echocardiography. . | 2013. “Percutaneous closure of PFO in patients with cryptogenic stroke does not appear superior to medical therapy according to currently available randomised data. Furthermore, it is associated with an increased incidence of atrial fibrillation. “^86^  2018. Ann Intern Med. “Surprisingly, the first 3 RCTs (CLOSURE I, PC, and  RESPECT) in this field failed individually to show that  PFO closure was superior to medical therapy alone…In patients with PFO and cryptogenic stroke, transcatheter device closure decreases risk for recurrent stroke compared with medical therapy alone. Because recurrent stroke rates are low even with medical therapy alone and PFO closure might affect AF risk, shared decision making is crucial for this treatment.” ^82^ | Google Scholar in cited by |
| 190 | Effects of Off-Pump and On-Pump Coronary-Artery Bypass Grafting at 1 Year Lamy et al. | Cardiovascular disease | 3/28/2013 NEJM | Coronary-Artery bypass grafting (CABG) is a procedure that can be performed with the use of either a cardiopulmonary bypass, deemed as on-pump CABG, or on a beating heart with an aortic clamp (off-pump). Off-pump CABG was believed to decrease the risk of perioperative complications.^87^ However, several trials began to question the perceived benefits of off-pump CABG as compared to on-pump CABG.^88^ The CORONARY study set out to compare the two procedures in high-risk patients, whom the authors believed would most likely benefit from the off-pump procedure. CORONARY trial (N=4752) found that there were no significant differences in the rates of non-fatal stroke, renal failure or myocardial infarction, death, or repeat revascularization between the off-pump CABG procedure and on-pump CABG (12.1% and 13.3%, respectively; hazard ratio with off-pump CABG, 0.91; 95% confidence interval, 0.77 to 1.07; P=0.24) . This is a reversal of the off-pump CABG procedure in patients that required isolated CABG with median sternotomy and had one or more of the following risk factors: an age of 70 years or more, peripheral arterial disease, cerebrovascular disease or carotid stenosis of 70% or more of the luminal diameter, or renal insufficiency. | 2017. “Off-pump CABG is associated with an increase in very long-term (≥10 years) all-cause mortality compared with on-pump CABG.” ^89^ | PubMed similar articles |
| 191 | Off-Pump versus On-Pump Coronary-Artery Bypass Grafting in Elderly Patients Diegeler et al. | Cardiovascular disease | 3/28/2013 NEJM | Similar to the CORONARY study, the GOPCABE trial (N=2539) evaluated the clinical outcomes of off-pump vs. on-pump CABG. This trial focused on older adults (75 years or older), a population thought to be most likely to benefit from an off-pump procedure (OPCAB). GOPCABE found that, after 30 days, patients exhibited no difference between the off-pump and on-pump CABG in rates of death, stroke, MI, revascularization, or renal failure (7.8% vs. 8.2%; odds ratio, 0.95; 95% confidence interval, 0.71 to 1.28; P=0.74). The outcomes were the same after one year after surgery. The findings of both the CORONARY study and GOPCABE trial provide evidence against favoring off-pump CABG in a subset of patients, a procedure that US surgeons were doing in 21% of cases in 2002. Additionally, off-pump CABG is a procedure that has significantly higher costs and is a substantially more challenging procedure for surgeons to perform. This is a reversal of the off-pump CABG procedure in elderly patients who are at least 75 years of age. | 2016. “Current retrospective studies with small numbers, examining the impact of OPCAB on early mortality and morbidity in octogenarians, have failed to prove overwhelming superiority of one technique over the other. At present it can be safely concluded that both on-pump and off-pump CABG are reasonable revascularization strategies in octogenarians.”^90^ Study not included in this review. | Google scholar within citing articles |
| 192 | A Randomized Trial of Glutamine and Antioxidants in Critically Ill Patients  Heyland et al. | Critical care medicine | 04/18/2013 NEJM | Critically ill patients suffer from oxidative stress. Canadian guidelines from 2003 recommended that antioxidants and glutamine-enriched formula be considered for ventilated, critically ill adult patients.^91^ This study found that early provision of glutamine or antioxidants (selenium, zinc, beta carotene, vitamin E, and vitamin C) did not improve 28-day mortality in critically ill patients compared to placebo. Furthermore, glutamine was associated with an increase in mortality. This is a reversal of administering glutamine or antioxidants to critically ill patients with multi-organ failure to reduce mortality. | 2016. “The main finding of our meta-analysis is that there was a lack of treatment effect when critically ill patients were treated with IV [selenium] Se as single or combined therapy.”^92^  2015. “The results of the present meta-analysis suggest that [glutamine] GLN supplementation given to a mixed population of critically ill patients does not significantly affect primary outcome measures such as hospital and ICU mortality.” ^93^ | PubMed articles cited by |
| 193 | Surgery versus Physical Therapy for a Meniscal Tear and Osteoarthritis  Katz et al. | Orthopedic | 5/02/2013 NEJM | Clinicians who suspect a tear in the meniscus may refer patients either to a surgeon for arthroscopic partial meniscectomy, if they are experiencing pain, or to physical therapy, if they are not feeling pain. This procedure is frequently done in the United States; one estimate is that more than 465,000 patients receive this procedure annually.^94,95^ Given the frequency and cost of arthroscopic partial meniscectomy and lack of concrete evidence on the clinical benefit of the procedure, the METEOR trial (N=351) was designed to assess the efficacy of arthroscopic partial meniscectomy surgery as compared with a physical-therapy for increasing physical function of patients with a meniscal tear and moderate osteoarthritis.^94^ METEOR found that there was not a significant decrease in the WOMAC score—a measure of physical function in which a higher score means worse physical function—between the patients undergoing surgery and those receiving initial physical therapy. The WOMAC score after 6 months was 20.9 points (95% confidence interval [CI], 17.9 to 23.9) in the surgical group and 18.5 (95% CI, 15.6 to 21.5) in the physical-therapy group (mean difference, 2.4 points; 95% CI, −1.8 to 6.5). The authors conclude that the finding of the METEOR trial advocates for an initial nonoperative strategy for treatment. This is a reversal of surgery for a meniscal tear detected on magnetic resonance imaging (MRI) and osteoarthritis in patients 45 year of age or older. | 2016. “Further evidence is required to determine which patient groups have good outcomes from each intervention. Given the current widespread use of arthroscopic meniscal surgeries, more research is urgently needed to support evidence-based practice in meniscal surgery in order to reduce the numbers of ineffective interventions and support potentially beneficial surgery.”^96^ | PubMed similar articles |
| 194 | n–3 Fatty Acids in Patients with Multiple Cardiovascular Risk Factors The Risk and Prevention Study Collaborative Group | Cardiovascular disease | 5/09/2013 NEJM | Observational studies have shown a reduction in cardiovascular disease, likely stemming from, in part, the anti-inflammatory effects of eicosapentaenoic acid (EPA) and docosahexaenoic acid (DHA).^97^ Several cardiology societies, including the American Heart Association and the European Society for Cardiology recommend 1 g/day of EPA and DHA for the secondary prevention of cardiovascular events, but these recommendations are based primarily off of observational studies.^97^ In this randomized trial (N=12,513), omega-3 fatty acid supplements did not improve cardiovascular mortality or morbidity in people with multiple cardiovascular risk factors (adjusted hazard ratio with n−3 fatty acids, 0.97; 95% confidence interval, 0.88 to 1.08; P=0.58). This is a reversal of secondary prevention of cardiovascular events by consumption of EPA and DHA in patients with multiple cardiovascular risk factors, clinical evidence of atherosclerotic vascular disease, or any other condition putting the patient at high cardiovascular risk in the opinion of the patient's general practitioner. | 2016. “There is currently a lack of evidence to support the routine use of omega-3 PUFAs in the primary and secondary prevention of CVD. Pharmacists are ideally situated to engage patients in the discussion of the lack of benefit and possible risk of omega-3 PUFA supplements.” ^98^ | PubMed in cited by articles |
| 195 | A Randomized Trial of Nighttime Physician Staffing in an Intensive Care Unit Kerlin et al. | Critical care medicine | 6/06/2013 NEJM | One-third of academic ICUs in the United States and nearly three-quarters of ICUs in Europe, use in-hospital intensivist staff (board certified attending physicians) at night.^99^ This practice was put in place without any supporting evidence.^100^ The SUNSET-ICU trial (N=1598) compared whether patients admitted to the ICU from the hours of 7 p.m. to 7 a.m. treated by in-hospital intensivists and medical residents had a shorter length of stay than patients seen by residents alone. SUNSET-ICU found that nighttime in-hospital intensivist staffing did not improve patient outcomes, specifically the duration of stay in the ICU (rate ratio for the time to ICU discharge, 0.98; 95% confidence interval [CI], 0.88 to 1.09; P=0.72). This is a reversal of nighttime in-hospital intensivists. | None found | key words: nighttime physician staffing, intensive care unit, ICU, length of stay |
| 196 | Racemic Adrenaline and Inhalation Strategies in Acute Bronchiolitis Skjerven et al. | Pulmonary disease | 6/13/2013 NEJM | Acute bronchiolitis is a condition in which infants sometimes require ventilatory support. Although existing guidelines do not recommend the use of bronchodilators for treatment of acute bronchiolitis,^101^ it is often the case that for babies with this condition clinicians often prescribe inhaled adrenaline.^102^ The Bronchiolitis All-studySE-Norway (N=404), tested whether inhaled racemic adrenaline in the treatment of acute bronchiolitis is superior to using inhaled saline solution, in infants. This randomized trial found that in patients with acute bronchiolitis, treatment with inhalations of racemic adrenaline did not result in a shorter hospital stay than with the treatment of inhaled saline (Confidence interval [cl], 4.5 (-6.5 to 15.5);(P=0.43). This is a reversal of racemic adrenaline in hospitalized infants with acute bronchiolitis not for infants in the emergency room. | None found | Google Scholar within citing articles |
| 197 | Clopidogrel in Infants with Systemic-to-Pulmonary-Artery Shunts Wessel et al. | Pediatrics | 6/20/2013 NEJM | Congenital heart disease is one of the most common types of congenital disabilities associated with a high mortality during the first year of life. The use of systemic-to-pulmonary-artery shunts to treat a variety of different congenital heart diseases is common. In adults undergoing this procedure, prophylactic antiplatelet therapy using aspirin and clopidogrel is used.^103^ Despite a lack evidence of the efficacy and safety, pediatricians increasingly used clopidogrel as prophylactic therapy for children who had a systemic-to-pulmonary artery shunt. In fact, the use of clopidogrel increased by a factor of 15 from 2001 to 2009 in children's hospitals in the United States,^104^ and pediatric cardiovascular doctors rely on evidence from small reviews to ascertain the safety and efficacy of clopidogrel. The CLARINET trial (N=2839) evaluated whether clopidogrel use, in infants with a systemic-to-pulmonary shunt, significantly reduced mortality and morbidity as compared with placebo. CLARINET found that the addition of clopidogrel to aspirin as prophylactic therapy in infants did not meaningfully lessen the rate of death as compared to placebo (absolute risk difference, 1.4 percentage points; relative risk reduction with clopidogrel, 11.1%; 95% confidence interval, –19.2 to 33.6; P=0.43). This is a reversal of the addition of clopidogrel to treatment of infant patients (92 days of age or younger) needing a systemic-to-pulmonary artery shunt operation. | None found | key words: clopidogrel, infants, systemic-to-pulmonary-artery shunts, death, heart transplant, thrombosis |
| 198 | Rapid Blood-Pressure Lowering in Patients with Acute Intracerebral Hemorrhage Anderson et al. | Neurology/Neurosurgery | 6/20/2013 NEJM | The American Heart Association guidelines at the time of the study recommended a mean arterial pressure lower than 110 mmHg in patients with elevated intracranial pressure.^105^ This recommendation was based on the findings of the INTERACT trial in which patients with intracerebral hemorrhage, a type of stroke, improved when mean arterial pressure was aggressively lowered (systolic pressure of <140 mm Hg).^106,107^ The INTERACT2 trial randomized patients with intracerebral hemorrhage to receive intensive blood pressure lowering of systolic blood pressure (<140 mm Hg within 1 hour) or guideline recommended treatment, (systolic level of <180 mm Hg). Early intensive lowering of blood pressure did not result in a reduction of death or major disability (odds ratio with intensive treatment, 0.87; 95% confidence interval, 0.75 to 1.01; P=0.06). This is a reversal of rapid blood-pressure lowering in patients with spontaneous acute intracerebral hemorrhage who had elevated systolic blood pressure. | 2014. Cochrane review. "There is insufficient evidence that lowering blood pressure during the acute phase of stroke improves functional outcome. It is reasonable to withhold blood pressure-lowering drugs until patients are medically and neurologically stable, and have suitable oral or enteral access, after which drugs can than be reintroduced. In people with acute stroke, CCBs, ACEI, ARA, beta blockers and NO donors each lower blood pressure while phenylephrine probably increases blood pressure. Further trials are needed to identify which people are most likely to benefit from early treatment, in particular whether treatment started very early is beneficial… The effect of lowering blood pressure did not vary by stroke subtype (ischaemic stroke, mixed stroke, ICH) across 14 trials ^“108^ | Pubmed found |
| 199 | Cardiovascular Effects of Intensive Lifestyle Intervention in Type 2 Diabetes The Look AHEAD Research Group | Endocrinology, Diabetes, and Metabolism | 7/11/2013 NEJM | The American Diabetes Association recommends that overweight or obese adults with type 2 diabetes lose weight to reduce the risk of cardiovascular morbidity and mortality.^109^ The AHEAD study (N=5145) asked whether an intensive weight loss intervention would reduce cardiovascular morbidity and mortality among overweight/obese adults with type 2 diabetes. The study followed overweight or obese patients with type 2 diabetes for ten years and concluded that an intensive lifestyle intervention aimed at increasing physical activity and restricting caloric intake did not significantly reduce cardiovascular morbidity and mortality as compared to receiving diabetes support and education (1.83 and 1.92 events per 100 person-years, respectively; hazard ratio in the intervention group, 0.95; 95% confidence interval, 0.83 to 1.09; P=0.51). This is a reversal of an intensive weight intervention in patients with type 2 diabetes, for the prevention of cardiovascular morbidity and mortality. Patients were 45 to 75 years of age, a body-mass index of 25.0 or more; a glycated hemoglobin level of 11% or less; a systolic blood pressure of less than 160 mm Hg; a diastolic blood pressure of less than 100 mm Hg; a triglyceride level of less than 600 mg per deciliter (6.77 mmol per liter); the ability to complete a valid maximal exercise test, suggesting it was safe to exercise; and an established relationship with a primary care provider. | 2013. “For all-cause mortality, the pooled results showed no difference between the intervention and control groups at more than 10 years of follow-up.”^110^ | Google Scholar – RCT cited by MA |
| 200 | Therapies for Active Rheumatoid Arthritis after Methotrexate Failure O'Dell et al. | Rheumatology | 7/25/2013 NEJM | The standard treatment for patients with rheumatoid arthritis is to administer methotrexate.^111^ Around 30% of patients do not show improvement and so they must seek alternative treatment.^112^ The FDA has approved multiple types of drugs for patients where methotrexate has failed.^113^ The most commonly used type of drug is a tumor necrosis factor (TNF) inhibitor. A trial by Odell et al (N=353) examined patients with rheumatoid arthritis and randomized them to treatment with triple therapy (sulfasalazine and hydroxychloroquine added to methotrexate) or to etanercept-methotrexate therapy (etanercept, a TNF inhibitor, added to methotrexate). Simulating clinical practice, only those patients that did not show improvement with the initial treatment after 24 weeks were then switched to the etanercept treatment. After 48 weeks, the study found that there was no significant difference in clinical outcomes between the two groups( −2.1 with triple therapy and −2.3 with etanercept and methotrexate, P=0.26). This finding suggests that the addition of the more expensive TNF-inhibiting agents to methotrexate does not provide better clinical outcomes than cheaper, non-biological agents. This is a reversal of the addition on TNF-inhibitors to methotrexate for active rheumatoid arthritis. | 2016 “We did not find any statistical benefit for methotrexate plus biologic therapy compared with triple therapy. This has important policy implications given the difference in cost between these treatments.” ^114^ | Found in related articles on pubmed |
| 201 | A Trial Comparing Noninvasive Ventilation Strategies in Preterm Infants Kirpalani et al. | Pediatrics | 8/15/2013 NEJM | Bronchopulmonary dysplasia is a leading cause of death in extremely-low-birth-weight infants, as well as a strong predictor of later neurologic impairment and increased rates of rehospitalization.^115^ Tracheal intubation and mechanical ventilation are associated with bronchopulmonary dysplasia, and as such, clinicians avoid prolonged intubation and mechanical respiratory support in hopes of reducing this risk.^116^ Clinicians often avoid intubation altogether and turn to either nasal continuous positive airway pressure (CPAP), the current standard of care, or nasal intermittent positive-pressure ventilation (IPPV) as an alternative method to intubation.^117^ Nasal IPPV is more complicated and costly than nasal CPAP. Despite this, nasal IPPV is commonly used in extremely-low-birth-weight infants.^118^ The NIPPV trial (N=1009) sought to establish whether nasal IPPV was superior to nasal CPAP at reducing the rate of bronchopulmonary dysplasia and improving survival rate of extremely-low-birth-weight infants. The trial found that there was no significant difference in a reduction of infant mortality between the two methods (adjusted odds ratio, 1.09; 95% confidence interval, 0.83 to 1.43; P=0.56). The authors call into question the widespread use of the more invasive and costlier nasal IPPV. This is a reversal of nasal IPPV in preterm infants. | 2017. Cochrane review. Although this Cochrane review concludes that, “NIPPV reduces the incidence of extubation failure and the need for re-intubation within 48 hours to one week more effectively than NCPAP; however, it has no effect on chronic lung disease nor on mortality [in preterm infants]” In this review, the Kirpalani et. al., trial is the only large definitive study on the topic. ^119^ | Found in related articles on pubmed |
| 202 | Pretreatment with Prasugrel in Non–ST-Segment Elevation Acute Coronary Syndromes Montalescot et al. | Cardiovascular disease | 9/12/2013 NEJM | For patients with acute coronary syndrome, complete or partial occlusion of a coronary artery is diagnosed by the presence or absence of an ST-segment elevation. Partial occlusion is deemed to be a non-ST-segment elevation myocardial infarction (NSTEMI).^120^ Upon arrival at the hospital, guidelines recommend pretreatment with clopidogrel, a P2Y12 receptor antagonist, for patients needing a percutaneous coronary intervention (PCI).^121^ However, it is unclear whether pretreatment with clopidogrel is safe and efficient when it is unknown whether the coronary-artery of a patient is occluded. Pretreatment delays a coronary-artery bypass grafting (CABG) procedure, which increases cost and unnecessarily exposes patients to a higher risk of bleeding if they do not need to undergo PCI.^122^ Prasugrel is another P2Y12-receptor antagonist that can be administered to patients with acute coronary syndrome. The ACCOAST trial (N=4033) tested the administration of prasugrel before or after coronary angiography. The researchers found that pretreatment with prasugrel did not reduce the rate of ischemic events in the overall population (hazard ratio with pretreatment, 1.02; 95% confidence interval [CI], 0.84 to 1.25; P=0.81). Additionally, there were significantly more major and life-threatening bleeding complications not related to CABG in the pretreatment group than in the control group — mostly among patients who underwent PCI (hazard ratio, 1.90; 95% CI, 1.19 to 3.02; P=0.006). The ACCOAST findings contradict the commonly used, guideline-recommended practice of pretreatment with aspirin and a P2Y12 antagonist for patients with NSTEMI. This is a reversal of pretreatment with Prasugrel in non-ST-segment elevation acute coronary syndromes. | 2015. "Preoperative exposure to clopidogrel on top of aspirin did not reduce the risk of MACE [major adverse cardiac event] but was associated with increased risk of bleeding and mortality."^123^ (Article not included in analysis) | Google scholar within citing articles |
| 203 | Saxagliptin and Cardiovascular Outcomes in Patients with Type 2 Diabetes Mellitus Scirica et al. | Endocrinology, Diabetes, and Metabolism | 10/03/2013 NEJM | Patients with type 2 diabetes are at higher risk of developing and dying from cardiovascular disease.^124^ As such, medications for type 2 diabetes should be evaluated for whether there are improved cardiovascular outcomes in patients taking these medications. Saxagliptin is one such medication that was approved by the FDA in 2009 for the treatment of type 2 diabetes.^125^ In this trial (N=16,492), rates of overall cardiovascular events were similar to those on placebo, but the rate of heart failure hospitalization was higher in the Saxagliptin group (3.5% vs. 2.8%; hazard ratio, 1.27; 95% CI, 1.07 to 1.51; P=0.007). This is a reversal of saxagliptin for improving cardiovascular outcomes in patients with type 2 diabetes. | 2015. “Compared with standard care, glycaemic lowering by various drugs or strategies might increase the risk of heart failure, with the magnitude of risk dependent on the method of glucose lowering and, potentially, weight gain.”^126^  2014. “Available data from RCTs suggest that DPP4i could be associated with an increased risk of heart failure, without any clear evidence of differences among drugs of the class. Although it is plausible that the risk is greater in some sub-populations of patients, current evidence is not yet sufficient to identify susceptible patients.”^127^ | Google scholar within citing articles |
| 204 | A Randomized Trial of Planned Cesarean or Vaginal Delivery for Twin Pregnancy Barrett et al. | Obstetrics and Gynecology | 10/03/2013 NEJM | Twin pregnancy increases the risk of adverse perinatal outcomes and affects 2-3% of all births.^128^ According to one small randomized controlled trial and several cohort studies,^129-131^ there is a reduced risk of twin delivery mortality and morbidity if women delivered with a planned cesarean section, as compared to a planned vaginal delivery. As a result of this observation and despite the lack of strong evidence to support planned cesarean section for twin delivery, the rates of an elective cesarean section for twins increased dramatically in North America from 53.4% to 75.0% in 2008, and a similar increase was observed worldwide.^132^ The Twin Birth Study compared the risk of fetal/neonatal death or serious neonatal morbidity between two delivery strategies for twin pregnancies between 32 weeks 0 days and 38 weeks six days of gestation. These delivery strategies were either planned cesarean delivery or planned vaginal delivery with cesarean delivery only when necessary. The Twin Birth trial (N=1398) found that there were no reductions in neonatal mortality or serious neonatal morbidity between delivery with a planned cesarean section vs. planned vaginal birth (2.2% and 1.9%, respectively; odds ratio with planned cesarean delivery, 1.16; 95% confidence interval, 0.77 to 1.74; P=0.49).^128^ This is a reversal of planned cesarean delivery for twin pregnancy. | 2015. Cochrane review. "Data mainly from one large, multicentre study found no clear evidence of benefit from planned caesarean section for term twin pregnancies with leading cephalic presentation. Data on long-term infant outcomes are awaited. Women should be informed of possible risks and benefits of labour and vaginal birth pertinent to their specific clinical presentation and the current and long-term effects of caesarean section for both mother and babies. There is insufficient evidence to support the routine use of planned caesarean section for term twin pregnancy with leading cephalic presentation, except in the context of further randomised trials."^133^ | Found in related articles on pubmed |
| 205 | Thrombus Aspiration during ST-Segment Elevation Myocardial Infarction Fröbert et al. | Cardiovascular disease | 10/24/2013 NEJM | For patients with ST-segment elevation myocardial infarction (STEMI), treated with percutaneous coronary intervention (PCI), the establishment of normal coronary blood flow has been challenging. Reduced coronary blood flow has been closely associated with reperfusion injury, which leads to arrhythmias, heart failure, and death, among other adverse effects.^134^ TAPAS, a randomized controlled trial, found that the addition of thrombus aspiration to PCI increased patients’ survival, the study’s secondary endpoint.^135^ A one-year follow up of TAPAS also reported improvement in survival. Because of the observed improvement in survival seen by the TAPAS investigators, the European and US practice guidelines were modified to include a IIa recommendation for the adjunctive use of thrombus aspiration in primary PCI.^136,137^ After these recommendations were implemented a meta-analysis found that routine thrombectomy led to an increased risk of stroke.^138^ The TASTE trial (N=7259) was designed to evaluate patients with STEMI to determine whether thrombus aspiration followed by PCI reduced all-cause mortality at 30 days, as compared to PCI alone. TASTE found that the addition of thrombus aspiration to PCI did not decrease the rate of all-cause mortality in patients with STEMI (hazard ratio, 0.94; 95% confidence interval, 0.72 to 1.22; P=0.63). This is a reversal of the addition of thrombus aspiration during ST-segment elevation myocardial infarction. | 2016. “Using the totality of evidence available through 2015, this meta-analysis failed to show that the routine use of aspiration thrombectomy in patients with ST-elevation myocardial infarction significantly reduces all-cause mortality, MACE, recurrent MI, TVR, or stent thrombosis.”^139^  2014. “The present meta-analysis suggested that there was no evidence that using manual thrombus aspiration in patients with STEMI could provide distinct benefits in long-term clinical outcomes.”^41^ | Found in related articles on pubmed |
| 206 | Targeted Temperature Management at 33°C versus 36°C after Cardiac Arrest Nielsen et al. | Critical care | 12/05/2013 NEJM | Unconscious patients who experience out-of-hospital cardiac arrest are at high-risk for death and neurological decline, and previous studies have shown that hypothermia in these patients increased survival rates and improved neurological outcomes.^140^ In 2010, guidelines recommended that comatose patients should be cooled to 32-34 degrees Celsius after out-of-hospital cardiac arrest.^141^ This study (N=939) found that hypothermia at a targeted temperature of 33 degrees Celsius did not affect rates of all-cause mortality (hazard ratio with a temperature of 33°C, 1.06; 95% confidence interval [CI], 0.89 to 1.28; P=0.51) or neurological function (risk ratio, 1.02; 95% CI, 0.88 to 1.16; P=0.78) compared to a targeted temperature of 36 degrees Celsius in unconscious survivors of out-of-hospital cardiac arrest. This is a reversal of cooling patients to 33 degrees after cardiac arrest. | 2016. Cochrane review. "Currently, there is no convincing evidence to clearly delineate beneficial or harmful effects of pre-hospital induction of cooling in comparison to in-hospital induction of cooling. This conclusion is based on very low quality evidence."^142^ | Found in related articles on pubmed |
| 207 | A Pharmacogenetic versus a Clinical Algorithm for Warfarin Dosing Kimmel et al. | Cardiovascular disease | 12/12/2013 NEJM | Warfarin is the most commonly used anticoagulant in the world, but because of its anti-clotting nature, it can also cause excess bleeding if patients are not monitored closely enough.^143^ Two genes, CYP2C9 and VK0RC1, have been identified as affecting warfarin’s effect.^144^ Based on observational and prospective studies, the FDA labeling was changed in 2007 to include a statement about the potential benefits of dosing warfarin based on genetic testing.^144^ Additionally, there are several CLIA-approved labs that perform these services, and CPT codes exist for this kind of testing.^145^ In the COAG trial (N=1015), genotype-guided dosing of warfarin was no better than clinically based dosing in keeping patients’ values in therapeutic range (adjusted mean difference, [genotype-guided group minus clinically guided group], −0.2; 95% confidence interval, −3.4 to 3.1; P=0.91). This is a reversal of genotype guided dosing of warfarin. | 2014. “In this meta-analysis of randomized clinical trials, a genotype-guided dosing strategy did not result in a greater percentage of time that the INR was within the therapeutic range, fewer patients with an INR greater than 4, or a reduction in major bleeding or thromboembolic events compared with clinical dosing algorithms.”^146^ | PubMed similar articles |
| 208 | A Randomized Trial of Genotype-Guided Dosing of Acenocoumarol and Phenprocoumon Verhoef et al. | Cardiovascular disease | 12/12/2013 NEJM | Warfarin is the most commonly used anticoagulant in the world, but because of its anti-clotting nature, it can also cause excess bleeding if patients are not monitored closely enough.^143^ Two genes, CYP2C9 and VK0RC1, have been identified as affecting warfarin’s effect .^144^ Based on observational and prospective studies, the FDA labeling was changed in 2007 to include a statement about the potential benefits of dosing warfarin based on genetic testing.^145^ Additionally, there are several CLIA-approved labs that perform these services, and CPT codes exist for this kind of testing.^145^ In the EU-PACT trial (N=548), genotype-guided dosing of acenocoumarol or phenprocoumon was no better than clinical-guided dosing in keeping patients’ values in therapeutic range. The percentage of time in the therapeutic INR range was 61.6% for patients receiving genotype-guided dosing and 60.2% for those receiving clinically guided dosing (P=0.52). This is a reversal of genotype guided dosing of acenocoumarol and phenprocoumon. | 2014. “In this meta-analysis of randomized clinical trials, a genotype-guided dosing strategy did not result in a greater percentage of time that the INR was within the therapeutic range, fewer patients with an INR greater than 4, or a reduction in major bleeding or thromboembolic events compared with clinical dosing algorithms.” ^146^ | PubMed similar articles |
| 209 | Arthroscopic Partial Meniscectomy versus Sham Surgery for a Degenerative Meniscal Tear Sihvonen et al. | Orthopedic | 12/26/2013 NEJM | Arthroscopic partial meniscectomy is the most common orthopedic procedure. In fact, arthroscopic partial meniscectomies are performed approximately 700,000 times annually in the United States alone and have an estimated direct medical cost valued at $4 billion. FIDELITY is a large randomized control trial that enrolled patients with a degenerative tear of the medial meniscus without knee osteoarthritis and compared the treatment efficacy of arthroscopic partial meniscectomy as compared to a sham procedure.^147^ The FIDELITY trial (N=146) found that after 12 months of surgery the outcomes of knee pain after exercise, and improvement in the quality of life, were not significantly better in the group undergoing arthroscopic partial meniscectomy. There were no significant differences in outcomes between the treatment group and the group of patients that underwent a sham surgical procedure (between-group difference, −1.6 points; 95% confidence interval [CI], −7.2 to 4.0); WOMET score, 24.6 and 27.1 points, respectively (between-group difference, −2.5 points; 95% CI, −9.2 to 4.1); and score for knee pain after exercise, 3.1 and 3.3 points, respectively (between-group difference, −0.1; 95% CI, −0.9 to 0.7). This is a reversal of arthroscopic partial meniscectomy for a degenerative meniscal tear. | 2014. “There is moderate evidence to suggest that there is no benefit to arthroscopic meniscal débridement for degenerative meniscal tears in comparison with nonoperative or sham treatments in middle-aged patients with mild or no concomitant osteoarthritis.”^148^ | Google scholar within articles cited by |
| 210 | Subclinical Atrial Fibrillation and the Risk of Stroke Healey et al. | Cardiovascular disease | 01/12/2012  NEJM | Atrial fibrillation appears to increase a person’s risk of stroke.^149^ Pacing of the heart with pacemakers may be one way to regulate the heart to prevent adverse outcomes. In fact, more than 400,000 pacemakers and implantable cardioverter-defibrillators are placed each year.^149^ Several types of pacing are used for atrial fibrillation, including the continuous atrial overdrive pacing.^150^ In this trial (N= 2580), patients who had a recently implanted pacemaker were randomized to receive continuous atrial overdrive pacing or not (i.e., feature turned off). The researchers in this study found that continuous atrial overdrive pacing did not prevent the development of a clinical atrial tachyarrhythmia nor did it prevent stroke, systemic embolism, myocardial infarction, death from vascular causes, or hospitalization for heart failure (annual rate 5.22 versus 4.69 ; hazard ratio 1.13 (0.90-1.41) P=0.29)This is a reversal of continuous atrial overdrive pacing of pacemakers in subclinical atrial fibrillation and preventing stroke. | None found |  |
| 211 | ABVD Alone versus Radiation-Based Therapy in Limited-Stage Hodgkin's Lymphoma Meyer et al. | Oncology | 02/02/2012  NEJM | Treatment for Hodgkin’s lymphoma has positively evolved over time, in that the majority of patients can now be cured from this disease.^151^ Treatment strategies began to focus on minimizing the adverse effects of the treatment. In the 1970s and 1980s, ABVD treatment (doxorubicin, bleomycin, vinblastine, and dacarbazine) was developed and used in combination with radiation, but in the 1990s, it was discovered that a lower dose of radiation, in combination with ABVD, could be just as beneficial.^151^ This observation was confirmed in a randomized trial that found that survival was higher in patients with stage I or IIa Hodgkin’s lymphoma who were treated with ABVD only, compared to patients treated with ABVD and radiation (hazard ratio for event, 0.88; 95% CI, 0.54 to 1.43; P=0.60). This is a reversal of treating limited-stage Hodgkin’s lymphoma with a combination of ABVD and radiation as opposed to ABVD alone. | 2017. Cochrane review. “The addition of radiotherapy to chemotherapy has probably little or no difference on OS.”^152^ | Google scholar within cited articles |
| 212 | Antenatal Thyroid Screening and Childhood Cognitive Function Lazarus et al. | Pediatrics | 02/09/2012  NEJM | Hypothyroidism in pregnant women is linked to a range of adverse outcomes, most importantly miscarriage, preterm delivery, and reduced cognitive function in children. For decades physicians have recognized the association between maternal hypothyroxinemia and reduced IQ test performance in children.^153^ Despite the recent recommendation of guidelines published by the American Thyroid Association and the Endocrine Society, which advises testing of thyroid function only in women at increased risk rather than universal screening, physicians routinely perform thyroid-function screening based on the belief that earlier diagnosis and treatment will improve pregnancy.^154^ Indeed, surveys report that almost half the obstetricians in private practices in Maine and the majority of obstetricians in a Boston academic center reported ordering thyroid-function tests for screening in pregnancy.^155,156^ To test whether the antenatal screening of pregnant women reduces the impaired cognitive development of babies, Lazarus, and colleagues randomly assigned women to receive screening or no screening (N=405). Women in the screening arm who were found to be hypothyroid were treated with levothyroxine. The children of both groups underwent IQ testing at three years of age, and no significant difference in IQ between children in the screening (treated) group and those in the control group was observed. Among the children of women with positive results, the mean IQ scores were 99.2 and 100.0 in the screening and control groups, respectively (difference, 0.8; 95% confidence interval [CI], −1.1 to 2.6; P=0.40 by intention-to-treat analysis); the proportions of children with an IQ of less than 85 were 12.1% in the screening group and 14.1% in the control group (difference, 2.1 percentage points; 95% CI, −2.6 to 6.7; P=0.39). This is a reversal of antenatal thyroid screening in asymptomatic pregnant women. These findings provide support for guidelines published in 2011 by the American Thyroid Association and the Endocrine Society, which recommended against universal testing.^157^ | None found | Antenatal Thyroid Screening and Childhood Cognitive Function |
| 213 | A Randomized Trial of Nicotine-Replacement Therapy Patches in Pregnancy Coleman et al. | Obstetrics and Gynecology | 03/01/2012  NEJM | Cigarette smoking during pregnancy increases the risks of pregnancy complications, as well as the chance of delivering a low-birth-weight or preterm baby. Despite these risks, approximately 6 to 22% of pregnant women in high-income countries smoke, making cigarette smoking one of the leading causes of adverse pregnancy outcomes.^158^ Behavioral counseling is recommended for pregnant smokers,^159^ as is nicotine-replacement therapy, which is recommended by several guidelines.^160^ In the SNAP trial (N=1050), pregnant smokers receive behavioral counseling and were randomly assigned to either a standard course of nicotine patches or placebo. In this trial, it was found that a nicotine patch was no more effective than placebo in helping pregnant women to quit smoking(9.4% and 7.6%, respectively; unadjusted odds ratio with nicotine-replacement therapy, 1.26; 95% confidence interval, 0.82 to 1.96). This is a reversal of nicotine replacement therapy patches in pregnancy. | 2015. Cochrane review. "NRT [Nicotine Replacement Therapy] used in pregnancy for smoking cessation increases smoking cessation rates measured in late pregnancy by approximately 40%. There is evidence, suggesting that when potentially-biased, non-placebo RCTs are excluded from analyses, NRT is no more effective than placebo. There is no evidence that NRT used for smoking cessation in pregnancy has either positive or negative impacts on birth outcomes. "^161^ | Google search for nicotine patches in pregnancy systematic review |
| 214 | Closure of Patent Foramen Ovale versus Medical Therapy after Cryptogenic Stroke Carroll et al. | Cardiovascular disease | 03/15/2012  NEJM | Paradoxical embolisms may cause cryptogenic strokes or transient ischemic attacks (TIAs). Some studies have correlated the size of a patient’s patent foramen ovale (PFO), or the presence of an atrial septal aneurysm with an increased risk stroke, while other studies have failed to show that there is such a risk.^162^ As a result of these mixed observations doctors have no clear guideline on prophylactic treatment for patients with PFO who present with a cryptogenic stroke or TIA. However, despite the different findings on the benefits of PFO closure and an FDA classification of the use of a percutaneous transcatheter device for PFO closure as investigational, the off-label use in the US to close PFO with devices approved only for the closure of secundum atrial septal defects is common.^163^ The question of the benefit of PFO closure has been posed several times during the last 20 years and several randomized controlled trials have attempted to shed some clarity on this contentious issue. Shatzel et al. summarize the findings of these studies, Closure I 2012, PC 2013, and Respect 2013 had a composite endpoint that included mortality and they found that PFO closure did not decrease deaths.^80^ On the other hand, recent studies--Gore REDUCE 2017, CLOSE 2017 and RESPECT extended f/u 2017--focusing of large interatrial shunts or ASA found that PFO closure decrease rates of stroke.^80^ Here we report on the findings of the CLOSURE I trial (N=980) which asked whether there is a potential benefit –recurrent stroke prevention— of treating patients with PFO presenting with cryptogenic stroke or TIA, by using a percutaneous device plus antiplatelet medical therapy over the treatment with antiplatelet medical therapy alone. During two years of follow-up, CLOSURE I found no significant differences, between the two treatment groups, in the rate of death from any cause during the first 30 days and death from neurologic causes. In the intention-to-treat cohort, 9 patients in the closure group and 16 in the medical-therapy group had a recurrence of stroke (hazard ratio with closure, 0.49; 95% confidence interval [CI], 0.22 to 1.11; P=0.08). This is a reversal of PFO closure after cryptogenic stroke. We believe that this reversal stands because at the time of this study the available evidence indicated that PFO closure was ineffective. | 2015. Cochrane Review. “We found that, when compared with medical therapy, transcatheter device closure [TDC] failed to show any significant benefit in reducing the risk of recurrent stroke or similar events. However, there was a possible protective effect on recurrent strokes in those participants for whom an Amplatzer device was used compared with medical therapy. We did not find evidence that TDC increased the rate of serious adverse events overall. However, TDC increased the risk of new‐onset atrial fibrillation (where there is a problem with the rate or rhythm of the heartbeat) and may be associated with the type of device used.”^164^ | Closure of Patent Foramen Ovale versus Medical Therapy after Cryptogenic Stroke |
| 215 | Off-Pump or On-Pump Coronary-Artery Bypass Grafting at 30 Days Lamy et al. | Cardiovascular disease | 04/19/2012 | Coronary-artery bypass grafting (CABG) can be performed either with a still heart (on-pump CABG) or without a cardiopulmonary bypass on the beating heart (off-pump). Traditionally, surgeons performed surgery on the arrested heart (on-pump CABG), which allowed for increased surgical precision.^165^ However, surgeons grew concerned that the cross-clamping of the aorta, necessary for the on-pump CABG procedure, might be harmful to patients, which would explain the observed increase in mortality, risk of stroke or other systemic embolic events in these patients. The off-pump method, operating on a beating heart, was developed to decrease the perioperative risks.^166^ However, the clinical literature reported different results about the relative efficacy of off-pump CABG as compared with on-pump CABG.^167^ The 5 year follow-up of the CORONARY trial (N=4752) randomly assigned patients to on-pump CABG or off-pump CABG surgery and found that there were no significant differences in the rate of the death, myocardial infarction, stroke, or renal failure requiring dialysis between the two techniques, 30 days after surgery (9.8% vs. 10.3%; hazard ratio for the off-pump group, 0.95; 95% confidence interval [CI], 0.79 to 1.14; P=0.59). This is a reversal of off-pump CABG with respect to the 30-day rate of death, myocardial infarction, stroke, or renal failure requiring dialysis. | 2018. “Evidence from RCTs showed no differences between the techniques, whereas rigorously adjusted observational studies (with >1.1 million patients) and the combined analysis indicated that off-pump CABG offers lower short-term mortality but poorer long-term survival. These results suggest that, in real-world settings, greater operative safety with off-pump CABG comes at the expense of lasting survival gains.”^168^  2014. “A pooled analysis demonstrated no statistically significant difference in off-pump and on-pump CABG (hazard ratio, 1.10; 95% confidence interval, 0.93–1.29; *P* = 0.27)” ^169^ | PubMed search: off pump vs on pump cabg systematic review |
| 216 | Warfarin and Aspirin in Patients with Heart Failure and Sinus Rhythm Homma et al. | Cardiovascular disease | 05/17/2012  NEJM | Anticoagulation is often prescribed for patients who have heart failure because of the increased risk of stroke and thromboembolic events.^170^ For prevention, patients are often prescribed warfarin or some other antiplatelet agent such as aspirin.^171^ In this trial (N=2305), treatment of heart failure when in sinus rhythm with warfarin led to no better outcomes (composite endpoint of ischemic stroke, intracerebral hemorrhage, or death) than with treatment with aspirin hazard ratio with warfarin, 0.93; 95% confidence interval [CI], 0.79 to 1.10; P=0.40), which is less expensive and does not need monitoring and lab testing. This is a reversal of using warfarin in patients with heart failure who are in sinus rhythm. | 2016. Cochrane review. “There is evidence from RCTs to suggest that neither oral anticoagulation with warfarin or platelet inhibition with aspirin is better for mortality in systolic heart failure with sinus rhythm (high quality of the evidence for all-cause mortality and moderate quality of the evidence for non-fatal cardiovascular events and major bleeding events). Treatment with warfarin was associated with a 20% reduction in non-fatal cardiovascular events but a twofold higher risk of major bleeding complications (high quality of the evidence)”.^172^ | Pubmed |
| 217 | Prednisone, Azathioprine, and N-Acetylcysteine for Pulmonary Fibrosis The Idiopathic Pulmonary Fibrosis Clinical Research Network | Pulmonary disease | 05/24/2012 | Idiopathic pulmonary fibrosis is a lung disease with an unknown cause and poor prognosis—the median survival of patients after diagnosis is approximately 2 to 5 years.^173^ The treatment for this disease has been with the use of glucocorticoids or immunosuppressive agents. However, the recommendations of treatment differ between guidelines. One such guideline recommends treatment of patients with a subset of idiopathic pulmonary fibrosis, with a two-drug regimen (a combination of prednisone and either azathioprine or cyclophosphamide).^21^ Other international guidelines, however, suggest the treatment of idiopathic pulmonary fibrosis with a three-drug regimen (azathioprine, prednisone, and N-acetylcysteine [NAC]).^174^ In summary, combined immunosuppression and NAC was a widely used, conventional approach to the treatment of idiopathic pulmonary fibrosis, despite the different recommendations by international guidelines. In fact, a survey of pulmonologists reported that almost 50% of pulmonologists used a treatment of either two drugs (azathioprine plus prednisone) or three drugs when treating patients with mild idiopathic pulmonary fibrosis.^22^ The PANTHER-IPF trial (N=155), evaluated the three-drug regimen against NAC alone in patients with idiopathic pulmonary fibrosis. The study found that the combined three-drug regimen of prednisone, azathioprine, and NAC resulted in an increase in all-cause mortality (8 vs. 1, P=0.01), all-cause hospitalization (23 vs. 7, P<0.001), and the treatment of severe adverse events, so much, that the study was ended early. This is a reversal of the combined three-drug regimen prednisone, azathioprine and N-acetylcysteine for pulmonary fibrosis. | None found |  |
| 218 | Drotrecogin Alfa (Activated) in Adults with Septic Shock Ranieri et al. | Critical care medicine | 05/31/2012  NEJM | On account of the PROWESS study, recombinant human activated protein C, or drotrecogin alfa (activated) (DrotAA) became approved by the US FDA for the treatment of severe sepsis in 2001.^175^ Additional trials on DrotAA could not replicate the results of PROWESS, specifically in patients with a low risk of death or children with severe sepsis.^176^ Irrespective of only having one positive study, several trials terminated for futility, and concerns about severe bleeding, Eli Lily assembled a task force to promote sepsis-treatment bundles that would include the drug.^177^ Following the controversies regarding the approval by the US FDA of DrotAA, the agency limited the drugs’ use to patients with a ‘high-risk of death’ from severe sepsis and called for more trials.^178^ This decision was based on the subgroup analysis of the PROWESS trial which demonstrated a benefit if the drug was administered to more severely ill patients.^178^  The European Medicines Agency conditionally approved DrotAA for the treatments of patients with severe sepsis and multiple organ failure in 2002. In 2007, it found that additional placebo-controlled trials were necessary.^179^ Hence, the PROWESS-SHOCK study tested the hypothesis that DrotAA, as compared with placebo, would reduce mortality in patients with septic shock and a high risk of death. The researchers found that the administration of DrotAA in patients with septic shock did not significantly reduce all-cause mortality at 28 days (relative risk in the DrotAA group, 1.09; 95% confidence interval [CI], 0.92 to 1.28; P=0.31 or 90 days (relative risk, 1.04; 95% CI, 0.90 to 1.19; P=0.56), as compared with placebo. PROWESS-SHOCK is a reversal of DrotAA administered to adults with septic shock to prevent mortality. Eli Lily announced in 2011 that they were withdrawing the drug from the worldwide market.^180^ This is a reversal of administering DrotAA to patients with systemic infection, systemic inflammation, and shock to reduce mortality. | 2012. Cochrane review. “This updated review found no evidence suggesting that Human recombinant protein C (APC) should be used for treating patients with severe sepsis or septic shock. APC seems to be associated with a higher risk of bleeding. The drug company behind APC, Eli Lilly, has announced the discontinuation of all ongoing clinical trials using this drug for treating patients with severe sepsis or septic shock.” ^181^ | PubMed in cited by articles |
| 219 | Hydroxyethyl Starch 130/0.42 versus Ringer's Acetate in Severe Sepsis Perner et al. | Critical care medicine | 07/12/2012  NEJM | The Surviving Sepsis Campaign guidelines recommend the use of either colloids or crystalloids as intravenous fluids for the treatment of patients with hypovolemia due to severe sepsis.^182^ In practice, colloid solutions are used because of the belief that patients obtain rapid and lasting circulatory stabilization.^183^ Nevertheless, there is a lack of consensus about which therapy might better benefit patients with sepsis. Moreover, some trials reported that the use of high-molecular-weight hydroxyethyl starch (HES), a colloid, might harm patients with severe sepsis by causing acute kidney failure.^184^ The results from the Scandinavian Critical Care Trials Group (N=846) concluded that patients treated with hydroxyethyl starch had an increased risk of death at 90 days (relative risk, 1.35; 95% CI, 1.01 to 1.80; P=0.04) and were more likely to require renal-replacement therapy, compared to Ringer’s acetate. This is a reversal of colloids for treating hypovolemia in severe sepsis. | 2013. “In conventional meta-analyses including recent trial data, hydroxyethyl starch 130/0.38-0.45 versus crystalloid or albumin increased the use of renal replacement therapy and transfusion with red blood cells, and resulted in more serious adverse events in patients with sepsis. It seems unlikely that hydroxyethyl starch 130/0.38-0.45 provides overall clinical benefit for patients with sepsis.”^185^ | PubMed similar articles |
| 220 | n–3 Fatty Acids and Cardiovascular Outcomes in Patients with Dysglycemia The ORIGIN Trial Investigators | Cardiovascular disease | 07/26/2012  NEJM | Observational studies have shown a reduction in cardiovascular disease, likely stemming from, in part, the anti-inflammatory effects of eicosapentaenoic acid (EPA) and docosahexaenoic acid (DHA).^97^ Several cardiology societies, including the American Heart Association and the European Society for Cardiology recommend 1 g/day of EPA and DHA for the secondary prevention of cardiovascular events and for those with hypertriglyceridemia, but these recommendations are based primarily off observational studies.^97,186^ In this randomized trial (N=12,611), omega-3 fatty acid supplements did not reduce the rate of cardiovascular events in patients with recent myocardial infarction or heart failure (574 patients [9.1%] vs. 581 patients [9.3%]; hazard ratio, 0.98; 95% confidence interval [CI], 0.87 to 1.10; P=0.72). This is a reversal of n-3 fatty acid consumption to improve cardiovascular outcomes in patients with dysglycemia. | 2018. “This meta-analysis demonstrated that omega-3 fatty acids had no significant association with fatal or nonfatal coronary heart disease or any major vascular events. It provides no support for current recommendations for the use of such supplements in people with a history of coronary heart disease.”^187^  2014. “For the secondary prevention of CV disease, the evidence showed that there was no clinical benefit in using preparations of omega-3 fatty acids compounds. In addition, there was evidence of increased GI adverse effects.”^188^ | PubMed in articles cited by |
| 221 | Tight Glycemic Control versus Standard Care after Pediatric Cardiac Surgery Agus et al. | Pediatrics | 9/27/2012  NEJM | Postoperative morbidity and mortality are high in critically ill patients, and the use of tight glucose control was viewed as a plausible approach to treatment that could improve survival among patients in the ICU. In fact, some guidelines recommended the use of tight glucose control as a standard of therapy for critical care patients.^189,190^ However, due to the results of one study, which showed that tight glycemic control increased mortality in critically ill patients, most guidelines were revised and the routine use of tight glycemic control treatment was discouraged in critically ill patients.^191^ However, one study of critically ill children found that a tight glucose control regimen improved the rate of survival.^192^ The SPECS trial (N=490) set out to test whether tight glucose control after cardiac surgery was beneficial to pediatric patients. The SPECS trial found that there was no significant difference in the incidence of infection (8.6 vs. 9.9 per 1000 patient-days, P=0.67), mortality, hospital length of stay, or measures of organ failure, between tight glucose control and standard treatment. This is a reversal of tight glycemic control after pediatric cardiac surgery to improve survival. | 2014. “TGC [tight glucose control] with IIT [intensive insulin therapy] does not result in decrease in 30‐day mortality, but appears to reduce acquired infection in critically ill children. However, TGC with IIT is associated with higher incidence of hypoglycemia.”^193^ | Google scholar within citing articles |
| 222 | Intraaortic Balloon Support for Myocardial Infarction with Cardiogenic Shock Thiele et al. | Cardiovascular disease | 10/04/2012  NEJM | Cardiogenic shock (CS) complicating acute myocardial infarction (AMI) is associated with an extremely high mortality. Intraaortic balloon counterpulsation (IABP) is the most widely used form of mechanical hemodynamic support used in the treatment of AMI complicated by CS.^194^ International guidelines endorse the use of IABP for treating post-myocardial infarction shock, with a class I recommendation.^195,196^ Despite the recommendation of various guidelines, there is no strong evidence to support the use of an IABP intervention. The IABP-SHOCK II trial (N=600) examined whether IABP added to early revascularization resulted in lower all-cause 30-day mortality, compared to early revascularization, among patients with AMI complicated by CS. The trial showed that the routine use of an IABP, as compared with standard therapy, does not improve survival after 30-days (relative risk with IABP, 0.96; 95% confidence interval, 0.79 to 1.17; P=0.69). This is a reversal of the routine use of an IABP for AMI complicated by CS. | 2015. Cochrane review. "Available evidence suggests that IABP may have a beneficial effect on some haemodynamic parameters. However, this did not result in survival benefits so there is no convincing randomised data to support the use of IABP in infarct-related cardiogenic shock."^197^ | PubMed similar articles |
| 223 | Radiofrequency Ablation as Initial Therapy in Paroxysmal Atrial Fibrillation Nielsen et al. | Cardiovascular disease | 10/25/2012  NEJM | Atrial fibrillation is the most common heart arrhythmia, which manifests as uncoordinated contractions of the heart.^198^ Radiofrequency was attractive in that it was thought to “cure” atrial fibrillation.^199^ Radiofrequency ablation was first used in humans in the mid-1990s, but became a more commonly used method of treating atrial fibrillation in the 2000s.^199^ Although rare, catheter ablation can lead to risks such as death, pulmonary vein stenosis and phrenic-nerve injury.^200^ In this trial (n=294), time spent in atrial fibrillation (as measured by Holter monitors) did not differ between patients with paroxysmal atrial fibrillation assigned to radiofrequency ablation and those assigned antiarrhythmic drug therapy (90th percentile of arrhythmia burden, 13% and 19%, respectively; P=0.10). This is a reversal of radiofrequency ablation as initial therapy in patients with paroxysmal atrial fibrillation over medical therapy. | 2016. “RFA [Radiofrequency ablation] demonstrates an early but nonsustained superiority over AAD[Antiarrhythmic Drug Therapy ] for the improvement of quality of life.”^201^  2015. “Radiofrequency catheter ablation seems to be more effective than medical therapy as first-line treatment of paroxysmal AF in relatively young and otherwise healthy patients, but may also cause more severe adverse effects. These findings support the use of RFA as first-line therapy in selected patients, who understand the benefits and risks of the procedure. “^202^  RFA is an evolving topic, the first results of the randomized, multicenter, long-term, international CABANA clinical trial were presented at Heart Rhythm 2018, the Heart Rhythm Society’s 39th Annual Scientific Sessions. The trial included 2,204 patients across 126 sites worldwide and tested whether primary catheter ablation for the elimination of atrial fibrillation (AF) was superior to state-of-the-art drug therapy. The primary endpoint was a composite of clinical events consisting of death, disabling stroke, serious bleeding, or cardiac arrest. The primary outcome was seen in 8 % randomized to the ablation arm and 101 patients 9.2 % randomized to the drug arm. Ablation did not produce a significant reduction in the primary endpoint and in all-cause mortality when analyzed as randomized (intent-to-treat).^203^ The lack consensus regarding the potential benefits of RFA over AAD points to a need for a randomized placebo controlled trial. | Google Scholar within citing articles searched for “systematic review” |
| 224 | Hydroxyethyl Starch or Saline for Fluid Resuscitation in Intensive Care Myburgh et al. | Critical care medicine | 11/15/2012  NEJM | The administration of intravenous fluids to increase intravascular volume in the critically ill is common practice in the intensive care unit (ICU). They can be treated with either crystalloids or colloids; the most commonly crystalloid is 0.9% sodium chloride (saline), with hydroxyethyl starch (HES) being the most frequently used colloid.^204^ Although it is one of the most common therapies, studies have indicated that HES use in critically ill patients may increase the risk of acute kidney injury.^184^ The CHEST trial (N=294) was conducted to evaluate the safety and efficacy of administering HES (130/0.4) in 0.9% saline as compared with 0.9% saline alone for fluid resuscitation in adult patients treated in the ICU. The study found that there was no significant difference among the treatment groups in 90-day mortality (relative risk in the HES group, 1.06; 95% confidence interval [CI], 0.96 to 1.18; P=0.26), and actually increased the rate of acute kidney injury (P=0.005) and failure (P=0.12). This is a reversal of HES for fluid resuscitation in intensive care. | 2013. Cochrane review. "There is no evidence from randomised controlled trials that resuscitation with colloids reduces the risk of death, compared to resuscitation with crystalloids, in patients with trauma, burns or following surgery. Furthermore, the use of hydroxyethyl starch might increase mortality. As colloids are not associated with an improvement in survival and are considerably more expensive than crystalloids, it is hard to see how their continued use in clinical practice can be justified."^183^ | PubMed similar articles |
| 225 | Bedside Monitoring to Adjust Antiplatelet Therapy for Coronary Stenting Collet et al. | Cardiovascular disease | 11/29/2012  NEJM | Patients undergoing stent placement (600,000 patients in the US each year) are at increased risk of major adverse cardiac events (MACE).^205^ Antiplatelet therapy is used to minimize these risks, but because there are different responses to this therapy in individuals, platelet function monitoring is sometimes used.^206^ Multiple tests have been developed, including the one used in the ARCTIC trial (VerifyNow).^206^ In this study (N=2440), platelet-function monitoring did not lead to better outcomes (composite of death, myocardial infarction, stent thrombosis, stroke, or urgent revascularization) one year after stent placement than did standard antiplatelet therapy (hazard ratio, 1.13; 95% confidence interval [CI], 0.98 to 1.29; P=0.10). This is a reversal of platelet-function bedside monitoring to adjust antiplatelet therapy for coronary stenting. | None found |  |
| 226 | Ultrafiltration in Decompensated Heart Failure with Cardiorenal Syndrome Bart et al. | Cardiovascular disease | 12/13/2012  NEJM | Patients with acutely decompensated heart failure can experience an acute cardiorenal syndrome (type 1) worsening of renal function. Venovenous ultrafiltration is used to treat this condition in hopes of restoring control over the rate and volume of fluid removal, greater conservation of sodium, and less neurohormonal activation. In fact, current treatment guidelines state that ultrafiltration is a reasonable approach in patients with congestion that are not responding to medical therapy. However, whether ultrafiltration is a safe and efficacious therapy as compared with pharmacologic therapy for patients with acutely decompensated heart failure complicated by acute cardiorenal syndrome and persistent congestion is unknown.^207^ Therefore, CARRESS-HF (N=188) set out to compare the effect of ultrafiltration with that of stepped pharmacologic therapy on renal function and weight loss, in patients with heart failure who have worsening renal function and persistent congestion (a loss of 5.5±5.1 kg [12.1±11.3 lb] and 5.7±3.9 kg [12.6±8.5 lb], respectively; P=0.58). Not only were the main outcomes no better in those treated with ultrafiltration, patients also experienced a higher rate of adverse events (72% vs. 57%, P=0.03).This is a reversal of venovenous ultrafiltration to treat decompensated heart failure with cardiorenal syndrome. | 2017. “There is insufficient evidence supporting routine use of ultrafiltration in acute decompensated heart failure.”^208^ | Ultrafiltration in Decompensated Heart Failure with Cardiorenal Syndrome |
| 227 | Effect of Cinacalcet on Cardiovascular Disease in Patients Undergoing Dialysis The EVOLVE Trial Investigators | Nephrology | 12/27/2012  NEJM | Cardiovascular disease is prevalent among patients with chronic kidney disease (CKD), especially among patients treated with hemodialysis, in whom the risk of death from cardiovascular disease is increased by a factor of 10 or more as compared with the risk in the general population.^209^ Indirect evidence from studies suggests that traditional management of secondary hyperparathyroidism (HPT) could be associated with the development of cardiovascular disease in patients with CKD.^210^ Cinacalcet (Sensipar/Mimpara, Amgen)—a calcimimetic agent—was approved by the FDA and EU in 2004 for clinical use for treatment of secondary HTP in patients with CKD on dialysis.^211^ However, evidence supporting these approvals is lacking. Hence, the EVOLVE trial (N=3883) was designed to test the hypothesis that treatment regimen of secondary HPT with cinacalcet reduced the risks of death and nonfatal cardiovascular among patients with CKD who were undergoing dialysis. The EVOLVE trial found that patients who underwent dialysis did not experience a significant reduction in their risk of death or major cardiovascular events with cinacalcet treatment, as compared to those receiving placebo (relative hazard in the cinacalcet group vs. the placebo group, 0.93; 95% confidence interval, 0.85 to 1.02; P=0.11). This is a reversal of Cinacalcet on cardiovascular disease in patients undergoing dialysis. | 2014. Cochrane review. "Routine cinacalcet therapy reduced the need for parathyroidectomy in adults treated with dialysis and elevated PTH levels but does not improve all-cause or cardiovascular mortality. Cinacalcet increases risks of nausea, vomiting and hypocalcaemia, suggesting harms may outweigh benefits in this population."^212^ | PubMed similar articles |
| 228 | A Trial of Intracranial-Pressure Monitoring in Traumatic Brain Injury Chesnut et al. | Neurology/Neurosurgery | 12/27/2012 | Monitoring of intracranial pressure is standard of care for patients with severe traumatic brain injury. However, its use in guiding therapy has incomplete acceptance. There is insufficient evidence that this therapy is effective. In fact, successive editions of the guidelines for the management of severe traumatic brain injury have documented the inadequate evidence.^213^ The BEST: TRIP trial (N=324) was designed to determine whether the information derived from the monitoring of intracranial pressure in patients with severe traumatic brain injury improved medical practice and patient outcomes. The trial found that patients with a serious traumatic brain injury who received treatment guided by intracranial-pressure monitoring did not experience a clinically significant benefit over patients in which treatment was guided by a neurologic examination and serial CT imaging (score, 56 in the pressure-monitoring group vs. 53 in the imaging–clinical examination group; P=0.49). This is a reversal of intracranial-pressure monitoring in traumatic brain injury. | 2015. Cochrane review. "The data from the single RCT studying the role of routine ICP monitoring in acute traumatic coma fails to provide evidence to support the intervention. Research in this area is complicated by the fact that RCTs necessarily assess the combined impact of measurement of ICP with the clinical management decisions made in light of this data. Future studies will need to assess the added value of ICP data alongside other information from the multimodal monitoring typically performed in intensive care unit settings. Additionally, even within traumatically acquired brain injury (TBI), there is great heterogeneity in mechanisms, distribution, location and magnitude of injury, and studies within more homogeneous subgroups are likely to be more informative."^214^ | PubMed similar articles |

1.	Burket MW, Cooper CJ, Kennedy DJ, et al. Renal artery angioplasty and stent placement: Predictors of a favorable outcome. *American Heart Journal.* 2000;139(1):64-71.

2.	Murphy TP, Soares G, Kim M. Increase in Utilization of Percutaneous Renal Artery Interventions by Medicare Beneficiaries, 1996–2000. *American Journal of Roentgenology.* 2004;183(3):561-568.

3.	Jenks S, Yeoh SE, Conway BR. Balloon angioplasty, with and without stenting, versus medical therapy for hypertensive patients with renal artery stenosis. *Cochrane Database of Systematic Reviews.* 2014(12).

4.	Churchyard GJ, Fielding KL, Lewis JJ, et al. A Trial of Mass Isoniazid Preventive Therapy for Tuberculosis Control. *New England Journal of Medicine.* 2014;370(4):301-310.

5.	Health Do. The South African Tuberculosis Control Programme: Practical guidelines. In: Department of Health Pretoria; 1996.

6.	Gilbert MR, Dignam JJ, Armstrong TS, et al. A Randomized Trial of Bevacizumab for Newly Diagnosed Glioblastoma. *New England Journal of Medicine.* 2014;370(8):699-708.

7.	Kolata GP, A. Costly Cancer Drug Offers Hope, but Also a Dilemma. *NY Times.* July 6, 2008, 2008;Health.

8.	Goonewardene TI, Hall MR, Rustin GJ. Management of asymptomatic patients on follow-up for ovarian cancer with rising CA-125 concentrations. *The lancet oncology.* 2007;8(9):813-821.

9.	Khasraw M, Ameratunga MS, Grant R, Wheeler H, Pavlakis N. Antiangiogenic therapy for high-grade glioma. *Cochrane Database of Systematic Reviews.* 2014(9).

10.	Erstad BL, Gales BJ, Rappaport WD. The use of albumin in clinical practice. *Archives of Internal Medicine.* 1991;151(5):901-911.

11.	Investigators TSS. A Comparison of Albumin and Saline for Fluid Resuscitation in the Intensive Care Unit. *New England Journal of Medicine.* 2004;350(22):2247-2256.

12.	Jiang L, Jiang S, Zhang M, Zheng Z, Ma Y. Albumin versus other fluids for fluid resuscitation in patients with sepsis: a meta-analysis. *PLoS One.* 2014;9(12):e114666.

13.	Plümer L, Seiffert M, Punke MA, et al. Aspirin Before Elective Surgery—Stop or Continue?: A Single-center Cross-sectional Study. *Deutsches Ärzteblatt International.* 2017;114(27-28):473.

14.	Václavík J, Táborský M. Antiplatelet therapy in the perioperative period. *European journal of internal medicine.* 2011;22(1):26-31.

15.	Columbo JA, Lambour AJ, Sundling RA, et al. A meta-analysis of the impact of aspirin, clopidogrel, and dual antiplatelet therapy on bleeding complications in noncardiac surgery. *Annals of surgery.* 2018;267(1):1-10.

16.	Rivers E, Nguyen B, Havstad S, et al. Early Goal-Directed Therapy in the Treatment of Severe Sepsis and Septic Shock. *New England Journal of Medicine.* 2001;345(19):1368-1377.

17.	Jones AE, Shapiro NI, Roshon M. Implementing Early Goal-directed Therapy in the Emergency Setting: The Challenges and Experiences of Translating Research Innovations into Clinical Reality in Academic and Community Settings. *Academic Emergency Medicine.* 2007;14(11):1072-1078.

18.	Yu H, Chi D, Wang S, Liu B. Effect of early goal-directed therapy on mortality in patients with severe sepsis or septic shock: a meta-analysis of randomised controlled trials. *BMJ open.* 2016;6(3):e008330.

19.	Angus DC, Barnato AE, Bell D, et al. A systematic review and meta-analysis of early goal-directed therapy for septic shock: the ARISE, ProCESS and ProMISe Investigators. *Intensive Care Medicine.* 2015;41(9):1549-1560.

20.	Prednisone, Azathioprine, and N-Acetylcysteine for Pulmonary Fibrosis. *New England Journal of Medicine.* 2012;366(21):1968-1977.

21.	Society AT. Idiopathic pulmonary fibrosis: diagnosis and treatment: international consensus statement. *Am J Respir Crit Care Med.* 2000;161:646-664.

22.	Peikert T, Daniels C, Beebe TJ, Meyer K, Ryu JH. Assessment of current practice in the diagnosis and therapy of idiopathic pulmonary fibrosis. *Respiratory medicine.* 2008;102(9):1342-1348.

23.	Bradley B, Branley HM, Egan JJ, et al. Interstitial lung disease guideline: the British Thoracic Society in collaboration with the Thoracic Society of Australia and New Zealand and the Irish Thoracic Society. *Thorax.* 2008;63 Suppl 5:v1-58.

24.	Sun T, Liu J, Zhao DW. Efficacy of N-Acetylcysteine in Idiopathic Pulmonary Fibrosis: A Systematic Review and Meta-Analysis. *Medicine.* 2016;95(19):e3629.

25.	Harrast MA. Epidural steroid injections for lumbar spinal stenosis. *Current Reviews in Musculoskeletal Medicine.* 2008;1(1):32-38.

26.	Friedly J, Chan L, Deyo R. Increases in lumbosacral injections in the Medicare population: 1994 to 2001. *Spine.* 2007;32(16):1754-1760.

27.	Chou R, Hashimoto R, Friedly J, et al. AHRQ Technology Assessments. In: *Pain Management Injection Therapies for Low Back Pain.* Rockville (MD): Agency for Healthcare Research and Quality (US); 2015.

28.	Carlson LA. Nicotinic acid: the broad-spectrum lipid drug. A 50th anniversary review. *Journal of internal medicine.* 2005;258(2):94-114.

29.	Jackevicius CA, Tu JV, Ko DT, de Leon N, Krumholz HM. Use of niacin in the united states and canada. *JAMA Internal Medicine.* 2013;173(14):1379-1381.

30.	O'Riordan M. Niacin/laropiprant combination approved in European Union, Iceland, and Norway. 2008; https://www.medscape.com/viewarticle/790175. Accessed August 06, 2018.

31.	Schandelmaier S, Briel M, Saccilotto R, et al. Niacin for primary and secondary prevention of cardiovascular events. *Cochrane Database of Systematic Reviews.* 2017(6).

32.	Mayosi BM, Burgess LJ, Doubell AF. Tuberculous pericarditis. *Circulation.* 2005;112(23):3608-3616.

33.	Programme TSATC. The South African National Tuberculosis Control Programme: Practical Guidelines. In: Department of Health; 2004.

34.	Chaisson RE, Post WS. Immunotherapy for Tuberculous Pericarditis. *New England Journal of Medicine.* 2014;371(12):1155-1157.

35.	Wiysonge CS, Ntsekhe M, Thabane L, et al. Interventions for treating tuberculous pericarditis. *The Cochrane Library.* 2017.

36.	Keeley EC, Boura JA, Grines CL. Primary angioplasty versus intravenous thrombolytic therapy for acute myocardial infarction: a quantitative review of 23 randomised trials. *The Lancet.* 2003;361(9351):13-20.

37.	Fröbert O, Lagerqvist B, Olivecrona GK, et al. Thrombus aspiration during ST-segment elevation myocardial infarction. *New England journal of medicine.* 2013;369(17):1587-1597.

38.	Beran G, Lang I, Schreiber W, et al. Intracoronary Thrombectomy With the X-Sizer Catheter System Improves Epicardial Flow and Accelerates ST-Segment Resolution in Patients With Acute Coronary Syndrome. *A Prospective, Randomized, Controlled Study.* 2002;105(20):2355-2360.

39.	Kushner FG, Hand M, Smith SC, et al. 2009 Focused Updates: ACC/AHA Guidelines for the Management of Patients With ST‐Elevation Myocardial Infarction (Updating the 2004 Guideline and 2007 Focused Update) and ACC/AHA/SCAI Guidelines on Percutaneous Coronary Intervention (Updating the 2005 Guideline and 2007 Focused Update). *Catheterization and Cardiovascular Interventions.* 2009;74(7).

40.	Elgendy AY, Elgendy IY, Mahmoud AN, Bavry AA. Long-term outcomes with aspiration thrombectomy for patients undergoing primary percutaneous coronary intervention: A meta-analysis of randomized trials. *Clinical Cardiology.* 2017;40(8):534-541.

41.	Deng S-B, Wang J, Xiao J, et al. Adjunctive Manual Thrombus Aspiration during ST-Segment Elevation Myocardial Infarction: A Meta-Analysis of Randomized Controlled Trials. *PLoS ONE.* 2014;9(11):e113481.

42.	Worcester EM, Coe FL. Nephrolithiasis. *Primary care.* 2008;35(2):369-vii.

43.	Uribarri J, Oh MS, Carroll HJ. The first kidney stone. *Annals of internal medicine.* 1989;111(12):1006-1009.

44.	Portis AJ, Sundaram CP. Diagnosis and initial management of kidney stones. *American family physician.* 2001;63(7):1329-1340.

45.	Norris JM, Barriga K, Hoffenberg EJ, et al. Risk of celiac disease autoimmunity and timing of gluten introduction in the diet of infants at increased risk of disease. *JAMA.* 2005;293(19):2343-2351.

46.	Ludvigsson JF, Green PHR. The Missing Environmental Factor in Celiac Disease. *New England Journal of Medicine.* 2014;371(14):1341-1343.

47.	Guandalini S. The influence of gluten: weaning recommendations for healthy children and children at risk for celiac disease. In: *Issues in Complementary Feeding.* Vol 60. Karger Publishers; 2007:139-155.

48.	Lionetti E, Castellaneta S, Francavilla R, et al. Introduction of Gluten, HLA Status, and the Risk of Celiac Disease in Children. *New England Journal of Medicine.* 2014;371(14):1295-1303.

49.	Silano M, Agostoni C, Sanz Y, Guandalini S. Infant feeding and risk of developing celiac disease: a systematic review. *BMJ open.* 2016;6(1).

50.	Zoungas S, Chalmers J, Neal B, et al. Follow-up of Blood-Pressure Lowering and Glucose Control in Type 2 Diabetes. *New England Journal of Medicine.* 2014;371(15):1392-1406.

51.	Holman RR, Paul SK, Bethel MA, Matthews DR, Neil HAW. 10-Year Follow-up of Intensive Glucose Control in Type 2 Diabetes. *New England Journal of Medicine.* 2008;359(15):1577-1589.

52.	Hemmingsen B, Lund SS, Gluud C, et al. Intensive glycaemic control for patients with type 2 diabetes: systematic review with meta-analysis and trial sequential analysis of randomised clinical trials. *BMJ.* 2011;343.

53.	Buehler AM, Cavalcanti AB, Berwanger O, et al. Effect of Tight Blood Glucose Control Versus Conventional Control in Patients with Type 2 Diabetes Mellitus: A Systematic Review with Meta-Analysis of Randomized Controlled Trials. *Cardiovascular Therapeutics.* 2011;31(3):147-160.

54.	Dellinger RP, Levy MM, Rhodes A, et al. Surviving Sepsis Campaign: International Guidelines for Management of Severe Sepsis and Septic Shock, 2012. *Intensive Care Medicine.* 2013;39(2):165-228.

55.	Marik PE, Corwin HL. Efficacy of red blood cell transfusion in the critically ill: A systematic review of the literature*. *Critical Care Medicine.* 2008;36(9):2667-2674.

56.	Chatterjee S, Wetterslev J, Sharma A, Lichstein E, Mukherjee D. Association of blood transfusion with increased mortality in myocardial infarction: A meta-analysis and diversity-adjusted study sequential analysis. *JAMA Internal Medicine.* 2013;173(2):132-139.

57.	Vincent MDPDJ-L, Sakr MBBCPDY, Sprung MDC, Harboe MDS, Damas MDP. Are Blood Transfusions Associated with Greater Mortality Rates?Results of the Sepsis Occurrence in Acutely Ill Patients Study. *Anesthesiology.* 2008;108(1):31-39.

58.	Holst LB, Haase N, Wetterslev J, et al. Lower versus Higher Hemoglobin Threshold for Transfusion in Septic Shock. *New England Journal of Medicine.* 2014;371(15):1381-1391.

59.	Dellinger RP, Carlet JM, Masur H, et al. Surviving Sepsis Campaign guidelines for management of severe sepsis and septic shock. *Intensive care medicine.* 2004;30(4):536-555.

60.	Bakker OJ, van Brunschot S, van Santvoort HC, et al. Early versus On-Demand Nasoenteric Tube Feeding in Acute Pancreatitis. *New England Journal of Medicine.* 2014;371(21):1983-1993.

61.	McClave SA, Martindale RG, Vanek VW, et al. Guidelines for the Provision and Assessment of Nutrition Support Therapy in the Adult Critically Ill Patient::Society of Critical Care Medicine (SCCM) and American Society for Parenteral and Enteral Nutrition (A.S.P.E.N.). *Journal of Parenteral and Enteral Nutrition.* 2009;33(3):277-316.

62.	Banks PA, Freeman ML. Practice Guidelines in Acute Pancreatitis. *The American Journal Of Gastroenterology.* 2006;101:2379.

63.	Vaughn VM, Shuster D, Rogers MM, et al. Early versus delayed feeding in patients with acute pancreatitis: A systematic review. *Annals of Internal Medicine.* 2017;166(12):883-892.

64.	Grigioni F, Detaint D, Avierinos J-F, Scott C, Tajik J, Enriquez-Sarano M. Contribution of ischemic mitral regurgitation to congestive heart failure after myocardial infarction. *Journal of the American College of Cardiology.* 2005;45(2):260-267.

65.	Bax JJ, Braun J, Somer ST, et al. Restrictive Annuloplasty and Coronary Revascularization in Ischemic Mitral Regurgitation Results in Reverse Left Ventricular Remodeling. *Circulation.* 2004;110(11 suppl 1):II-103-II-108.

66.	Lee R, Li S, Rankin JS, et al. Fifteen-Year Outcome Trends for Valve Surgery in North America. *The Annals of Thoracic Surgery.* 2011;91(3):677-684.

67.	Kopjar T, Gasparovic H, Mestres CA, Milicic D, Biocina B. Meta-analysis of concomitant mitral valve repair and coronary artery bypass surgery versus isolated coronary artery bypass surgery in patients with moderate ischaemic mitral regurgitation†. *European Journal of Cardio-Thoracic Surgery.* 2016;50(2):212-222.

68.	Derdak S, Mehta S, Stewart TE, et al. High-Frequency Oscillatory Ventilation for Acute Respiratory Distress Syndrome in Adults. *American Journal of Respiratory and Critical Care Medicine.* 2002;166(6):801-808.

69.	Sud S, Sud M, Friedrich JO, et al. High-frequency oscillatory ventilation versus conventional ventilation for acute respiratory distress syndrome. *Cochrane Database of Systematic Reviews.* 2016(4).

70.	Fessler HE, Hess DR. Does High-Frequency Ventilation Offer Benefits Over Conventional Ventilation in Adult Patients With Acute Respiratory Distress Syndrome? *Respiratory Care.* 2007;52(5):595-608.

71.	Lees KR, Bluhmki E, von Kummer R, et al. Time to treatment with intravenous alteplase and outcome in stroke: an updated pooled analysis of ECASS, ATLANTIS, NINDS, and EPITHET trials. *The Lancet.* 2010;375(9727):1695-1703.

72.	de los Ríos la Rosa F, Khoury J, Kissela BM, et al. Eligibility for IV rt-PA within a Population: The Effect of the ECASS III Trial. *Stroke; a Journal of Cerebral Circulation.* 2012;43(6):1591-1595.

73.	Meyers PM, Schumacher HC, Connolly ES, Heyer EJ, Gray WA, Higashida RT. Current Status of Endovascular Stroke Treatment. *Circulation.* 2011;123(22):2591-2601.

74.	Kidwell CS, Jahan R, Gornbein J, et al. A trial of imaging selection and endovascular treatment for ischemic stroke. *New England Journal of Medicine.* 2013;368(10):914-923.

75.	Singh B, Parsaik AK, Prokop LJ, Mittal MK. Endovascular Therapy for Acute Ischemic Stroke: A Systematic Review and Meta-analysis. *Mayo Clinic proceedings.* 2013;88(10):10.1016/j.mayocp.2013.1007.1015.

76.	Prasad V, Vandross A, Toomey C, et al. A Decade of Reversal: An Analysis of 146 Contradicted Medical Practices. *Mayo Clinic Proceedings.* 2013;88(8):790-798.

77.	Schuchlenz HW, Weihs W, Berghold A, Lechner A, Schmidt R. Secondary prevention after cryptogenic cerebrovascular events in patients with patent foramen ovale. *International Journal of Cardiology.* 2005;101(1):77-82.

78.	Kitsios GD, Dahabreh IJ, Abu Dabrh AM, Thaler DE, Kent DM. Patent Foramen Ovale Closure and Medical Treatments for Secondary Stroke Prevention. *A Systematic Review of Observational and Randomized Evidence.* 2012;43(2):422-431.

79.	Kutty S, Sengupta PP, Khandheria BK. Patent Foramen Ovale: The Known and the To Be Known. *Journal of the American College of Cardiology.* 2012;59(19):1665-1671.

80.	Shatzel JJ, Daughety MM, Prasad V, DeLoughery TG. PFO closure for secondary stroke prevention: is the discussion closed? *Journal of thrombosis and thrombolysis.* 2018;46(1):74-76.

81.	Mir H, Siemieniuk RAC, Ge LC, et al. Patent foramen ovale closure, antiplatelet therapy or anticoagulation in patients with patent foramen ovale and cryptogenic stroke: a systematic review and network meta-analysis incorporating complementary external evidence. *BMJ open.* 2018;8(7).

82.	Shah R, Nayyar M, Jovin IS, et al. Device Closure Versus Medical Therapy Alone for Patent Foramen Ovale in Patients With Cryptogenic Stroke: A Systematic Review and Meta-analysis. *Ann Intern Med.* 2018;168(5):335-342.

83.	Vaduganathan M, Qamar A, Gupta A, et al. Patent Foramen Ovale Closure for Secondary Prevention of Cryptogenic Stroke: Updated Meta-Analysis of Randomized Clinical Trials. *Am J Med.* 2018;131(5):575-577.

84.	O'Gara PT, Messe SR, Tuzcu EM, Catha G, Ring JC. Percutaneous Device Closure of Patent Foramen Ovale for Secondary Stroke Prevention. *<span class="subtitle">A Call for Completion of Randomized Clinical Trials: A Science Advisory From the American Heart Association/American Stroke Association and the American College of Cardiology Foundation <em>The American Academy of Neurology affirms the value of this science advisory</em></span>.* 2009;119(20):2743-2747.

85.	Ahmad Y, Howard JP, Arnold A, et al. Patent foramen ovale closure vs. medical therapy for cryptogenic stroke: a meta-analysis of randomized controlled trials. *European Heart Journal.* 2018;39(18):1638-1649.

86.	Wolfrum M, Froehlich GM, Knapp G, et al. Stroke prevention by percutaneous closure of patent foramen ovale: a systematic review and meta-analysis. *Heart.* 2014;100(5):389-395.

87.	Lamy A, Devereaux PJ, Prabhakaran D, et al. Effects of Off-Pump and On-Pump Coronary-Artery Bypass Grafting at 1 Year. *New England Journal of Medicine.* 2013;368(13):1179-1188.

88.	Novitzky D, Shroyer AL, Collins JF, et al. A study design to assess the safety and efficacy of on-pump versus off-pump coronary bypass grafting: the ROOBY trial. *Clinical Trials.* 2007;4(1):81-91.

89.	Takagi H, Ando T, Mitta S. Meta-Analysis Comparing ≥10-Year Mortality of Off-Pump Versus On-Pump Coronary Artery Bypass Grafting. *The American Journal of Cardiology.* 2017;120(11):1933-1938.

90.	Raja SG. Off-pump coronary artery bypass grafting in octogenarians. *Journal of Thoracic Disease.* 2016:S799-S807.

91.	Heyland D, Dhaliwal R, Drover J, Gramlich L, Dodek P. Canadian clinical practice guidelines for nutrition support in mechanically ventilated, critically ill adult patients. *Journal of Parenteral and Enteral Nutrition.* 2003;27(5):355-373.

92.	Manzanares W, Lemieux M, Elke G, Langlois PL, Bloos F, Heyland DK. High-dose intravenous selenium does not improve clinical outcomes in the critically ill: a systematic review and meta-analysis. *Critical Care.* 2016;20(1):356.

93.	Oldani M, Sandini M, Nespoli L, Coppola S, Bernasconi DP, Gianotti L. Glutamine Supplementation in Intensive Care Patients: A Meta-Analysis of Randomized Clinical Trials. *Medicine (Baltimore).* 2015;94(31):e1319.

94.	Katz JN, Brophy RH, Chaisson CE, et al. Surgery versus Physical Therapy for a Meniscal Tear and Osteoarthritis. *New England Journal of Medicine.* 2013;368(18):1675-1684.

95.	Bozic KJ, Roche M, Agnew SG. Hospital-based employment of orthopaedic surgeons—passing trend or new paradigm?: AOA critical issues. *JBJS.* 2012;94(9):e59.

96.	Monk P, Roberts PG, Palmer AJR, et al. The Urgent Need for Evidence in Arthroscopic Meniscal Surgery: A Systematic Review of the Evidence for Operative Management of Meniscal Tears. *The American Journal of Sports Medicine.* 2017;45(4):965-973.

97.	Von Schacky C, Harris WS. Cardiovascular benefits of omega-3 fatty acids. *Cardiovascular research.* 2007;73(2):310-315.

98.	Walz CP, Barry AR, Koshman SL. Omega-3 polyunsaturated fatty acid supplementation in the prevention of cardiovascular disease. *Canadian pharmacists journal : CPJ = Revue des pharmaciens du Canada : RPC.* 2016;149(3):166-173.

99.	Diaz-Guzman E, Colbert CY, Mannino DM, Davenport DL, Arroliga AC. 24/7 In-house Intensivist Coverage and Fellowship Education: A Cross-sectional Survey of Academic Medical Centers in the United States. *Chest.* 2012;141(4):959-966.

100.	Vincent JL, Suter P, Bihari D, Braining H. Organization of intensive care units in Europe: lessons from the EPIC study. *Intensive Care Medicine.* 1997;23(11):1181-1184.

101.	Lieberthal AS, Bauchner H, Hall CB, et al. Diagnosis and management of bronchiolitis. *Pediatrics.* 2006;118(4):1774-1793.

102.	Newcomb RW. Use of adrenergic bronchodilators by pediatric allergists and pulmonologists. *Am J Dis Child.* 1989;143(4):481-485.

103.	Wessel DL, Berger F, Li JS, et al. Clopidogrel in Infants with Systemic-to-Pulmonary-Artery Shunts. *New England Journal of Medicine.* 2013;368(25):2377-2384.

104.	Gentilomo C, Huang Y-S, Raffini L. Significant Increase in Clopidogrel Use Across U.S. Children’s Hospitals. *Pediatric Cardiology.* 2011;32(2):167-175.

105.	Morgenstern LB, Hemphill JC, Anderson C, et al. Guidelines for the Management of Spontaneous Intracerebral Hemorrhage: A Guideline for Healthcare Professionals From the American Heart Association/American Stroke Association. *Stroke; a journal of cerebral circulation.* 2010;41(9):2108-2129.

106.	Frontera JA. Blood Pressure in Intracerebral Hemorrhage — How Low Should We Go? *New England Journal of Medicine.* 2013;368(25):2426-2427.

107.	Anderson CS, Huang Y, Arima H, et al. Effects of Early Intensive Blood Pressure-Lowering Treatment on the Growth of Hematoma and Perihematomal Edema in Acute Intracerebral Hemorrhage. *The Intensive Blood Pressure Reduction in Acute Cerebral Haemorrhage Trial (INTERACT).* 2010;41(2):307-312.

108.	Bath PM, Krishnan K. Interventions for deliberately altering blood pressure in acute stroke. *The Cochrane Library.* 2014.

109.	Executive Summary. *Obesity Research.* 1998;6(S2):51S-179S.

110.	Schellenberg E, Dryden DM, Vandermeer B, Ha C, Korownyk C. Lifestyle interventions for patients with and at risk for type 2 diabetes: A systematic review and meta-analysis. *Annals of Internal Medicine.* 2013;159(8):543-551.

111.	Singh JA, Furst DE, Bharat A, et al. 2012 Update of the 2008 American College of Rheumatology (ACR) Recommendations for the use of Disease-Modifying Anti-Rheumatic Drugs and Biologics in the treatment of Rheumatoid Arthritis (RA). *Arthritis care & research.* 2012;64(5):625-639.

112.	Moreland LW, O’Dell JR, Paulus HE, et al. A Randomized Comparative Effectiveness Study of Oral Triple Therapy versus Etanercept plus Methotrexate in Early, Aggressive Rheumatoid Arthritis. *Arthritis and rheumatism.* 2012;64(9):2824-2835.

113.	O'Dell JR, Mikuls TR, Taylor TH, et al. Therapies for Active Rheumatoid Arthritis after Methotrexate Failure. *New England Journal of Medicine.* 2013;369(4):307-318.

114.	Hazlewood GS, Barnabe C, Tomlinson G, Marshall D, Devoe D, Bombardier C. Methotrexate monotherapy and methotrexate combination therapy with traditional and biologic disease modifying antirheumatic drugs for rheumatoid arthritis: abridged Cochrane systematic review and network meta-analysis. *BMJ.* 2016;353.

115.	Smith VC, Zupancic JAF, McCormick MC, et al. Rehospitalization in the first year of life among infants with bronchopulmonary dysplasia. *The Journal of Pediatrics.* 2004;144(6):799-803.

116.	Walsh MC, Morris BH, Wrage LA, et al. Extremely Low Birthweight Neonates with Protracted Ventilation: Mortality and 18-Month Neurodevelopmental Outcomes. *The Journal of Pediatrics.* 2005;146(6):798-804.

117.	Gregory GA, Kitterman JA, Phibbs RH, Tooley WH, Hamilton WK. Treatment of the Idiopathic Respiratory-Distress Syndrome with Continuous Positive Airway Pressure. *New England Journal of Medicine.* 1971;284(24):1333-1340.

118.	Owen LS, Morley CJ, Davis PG. Neonatal nasal intermittent positive pressure ventilation: what do we know in 2007? *Archives of Disease in Childhood Fetal and Neonatal Edition.* 2007;92(5):F414-F418.

119.	Lemyre B, Davis PG, De Paoli AG, Kirpalani H. Nasal intermittent positive pressure ventilation (NIPPV) versus nasal continuous positive airway pressure (NCPAP) for preterm neonates after extubation. *The Cochrane Library.* 2017.

120.	Keaney JFJ. P2Y12 Inhibition in Patients with NSTEMI — Can Later Be Better? *New England Journal of Medicine.* 2013;369(11):1056-1057.

121.	Jneid H, Anderson JL, Wright RS, et al. 2012 ACCF/AHA Focused Update of the Guideline for the Management of Patients With Unstable Angina/Non–ST-Elevation Myocardial Infarction (Updating the 2007 Guideline and Replacing the 2011 Focused Update): A Report of the American College of Cardiology Foundation/American Heart Association Task Force on Practice Guidelines. *Journal of the American College of Cardiology.* 2012;60(7):645-681.

122.	Montalescot G, Bolognese L, Dudek D, et al. Pretreatment with Prasugrel in Non–ST-Segment Elevation Acute Coronary Syndromes. *New England Journal of Medicine.* 2013;369(11):999-1010.

123.	Siller-Matula JM, Petre A, Delle-Karth G, et al. Impact of preoperative use of P2Y12 receptor inhibitors on clinical outcomes in cardiac and non-cardiac surgery: A systematic review and meta-analysis. *European Heart Journal: Acute Cardiovascular Care.* 2017;6(8):753-770.

124.	Leon BM, Maddox TM. Diabetes and cardiovascular disease: Epidemiology, biological mechanisms, treatment recommendations and future research. *World journal of diabetes.* 2015;6(13):1246.

125.	Administration UFaD. Approval letter: saxagliptin. In: Department of Health and Human Services; 2009.

126.	Udell JA, Cavender MA, Bhatt DL, Chatterjee S, Farkouh ME, Scirica BM. Glucose-lowering drugs or strategies and cardiovascular outcomes in patients with or at risk for type 2 diabetes: a meta-analysis of randomised controlled trials. *The Lancet Diabetes & Endocrinology.* 2015;3(5):356-366.

127.	Monami M, Dicembrini I, Mannucci E. Dipeptidyl peptidase-4 inhibitors and heart failure: a meta-analysis of randomized clinical trials. *Nutrition, Metabolism and Cardiovascular Diseases.* 2014;24(7):689-697.

128.	Barrett JFR, Hannah ME, Hutton EK, et al. A Randomized Trial of Planned Cesarean or Vaginal Delivery for Twin Pregnancy. *New England Journal of Medicine.* 2013;369(14):1295-1305.

129.	HOFFMANN E, OLDENBURG A, RODE L, TABOR A, RASMUSSEN S, SKIBSTED L. Twin births: cesarean section or vaginal delivery? *Acta Obstetricia et Gynecologica Scandinavica.* 2012;91(4):463-469.

130.	Armson BA, O’Connell C, Persad V, Joseph KS, Young DC, Baskett TF. Determinants of perinatal mortality and serious neonatal morbidity in the second twin. *Obstetrics & Gynecology.* 2006;108(3, Part 1):556-564.

131.	Smith GCS, Shah I, White IR, Pell JP, Dobbie R. Mode of delivery and the risk of delivery‐related perinatal death among twins at term: a retrospective cohort study of 8073 births. *BJOG: An International Journal of Obstetrics & Gynaecology.* 2005;112(8):1139-1144.

132.	Lee HC, Gould JB, Boscardin WJ, El-Sayed YY, Blumenfeld YJ. Trends in Cesarean Delivery for Twin Births in the United States: 1995–2008. *Obstetrics & Gynecology.* 2011;118(5):1095-1101.

133.	Hofmeyr GJ, Barrett JF, Crowther CA. Planned caesarean section for women with a twin pregnancy. *Cochrane Database Syst Rev.* 2011;12.

134.	Opie LH. Reperfusion injury and its pharmacologic modification. *Circulation.* 1989;80(4):1049-1062.

135.	Svilaas T, Vlaar PJ, van der Horst IC, et al. Thrombus Aspiration during Primary Percutaneous Coronary Intervention. *New England Journal of Medicine.* 2008;358(6):557-567.

136.	Steg PG, James SK, Atar D, et al. ESC Guidelines for the management of acute myocardial infarction in patients presenting with ST-segment elevationThe Task Force on the management of ST-segment elevation acute myocardial infarction of the European Society of Cardiology (ESC). *European Heart Journal.* 2012;33(20):2569-2619.

137.	Cannon CP, Brindis RG, Chaitman BR, et al. 2013 ACCF/AHA Key Data Elements and Definitions for Measuring the Clinical Management and Outcomes of Patients With Acute Coronary Syndromes and Coronary Artery Disease. *A Report of the American College of Cardiology Foundation/American Heart Association Task Force on Clinical Data Standards (Writing Committee to Develop Acute Coronary Syndromes and Coronary Artery Disease Clinical Data Standards).* 2013;127(9):1052-1089.

138.	De Luca G, Navarese EP, Suryapranata H. A meta-analytic overview of thrombectomy during primary angioplasty. *International Journal of Cardiology.* 2013;166(3):606-612.

139.	Dominguez AC, Bittl JA, El-Hayek G, Contreras E, Tamis-Holland JE. Meta-analysis of randomized controlled trials comparing percutaneous coronary intervention with aspiration thrombectomy Vs. Conventional percutaneous coronary intervention during ST-segment elevation myocardial infarction. *Catheterization and Cardiovascular Interventions.* 2015;87(7):1203-1210.

140.	Group THaCAS. Mild Therapeutic Hypothermia to Improve the Neurologic Outcome after Cardiac Arrest. *New England Journal of Medicine.* 2002;346(8):549-556.

141.	Peberdy MA, Callaway CW, Neumar RW, et al. Part 9: Post–Cardiac Arrest Care. *2010 American Heart Association Guidelines for Cardiopulmonary Resuscitation and Emergency Cardiovascular Care.* 2010;122(18 suppl 3):S768-S786.

142.	Arrich J, Holzer M, Havel C, Warenits A-M, Herkner H. Pre-hospital versus in-hospital initiation of cooling for survival and neuroprotection after out-of-hospital cardiac arrest. *Cochrane Database of Systematic Reviews.* 2016(3).

143.	Pirmohamed M. Warfarin: almost 60 years old and still causing problems. *British journal of clinical pharmacology.* 2006;62(5):509-511.

144.	Kimmel SE, French B, Kasner SE, et al. A pharmacogenetic versus a clinical algorithm for warfarin dosing. *New England Journal of Medicine.* 2013;369(24):2283-2293.

145.	Michael A Pacanowski RC-D. Genotype-guided warfarin dosing now a reality. 2008. https://www.healio.com/cardiology/news/print/cardiology-today/%7B3fd68bb4-dbed-4d32-b498-8d7db7a762a5%7D/genotype-guided-warfarin-dosing-now-a-reality.

146.	Stergiopoulos K, Brown DL. Genotype-guided vs clinical dosing of warfarin and its analogues: meta-analysis of randomized clinical trials. *JAMA internal medicine.* 2014;174(8):1330-1338.

147.	Sihvonen R, Paavola M, Malmivaara A, et al. Arthroscopic Partial Meniscectomy versus Sham Surgery for a Degenerative Meniscal Tear. *New England Journal of Medicine.* 2013;369(26):2515-2524.

148.	Khan M, Evaniew N, Bedi A, Ayeni OR, Bhandari M. Arthroscopic surgery for degenerative tears of the meniscus: a systematic review and meta-analysis. *CMAJ : Canadian Medical Association Journal.* 2014;186(14):1057-1064.

149.	Healey JS, Connolly SJ, Gold MR, et al. Subclinical atrial fibrillation and the risk of stroke. *New England Journal of Medicine.* 2012;366(2):120-129.

150.	Lau C-P. Pacing for atrial fibrillation. *Heart.* 2003;89(1):106-112.

151.	The Cure of Hodgkin Lymphoma. *50 Years in Hematology: Research that Revolutionized Patient Care.* 2008. <http://www.hematology.org/About/History/50-Years/1528.aspx>.

152.	Blank O, von Tresckow B, Monsef I, Specht L, Engert A, Skoetz N. Chemotherapy alone versus chemotherapy plus radiotherapy for adults with early stage Hodgkin lymphoma. *The Cochrane Library.* 2017.

153.	Man EB, Holden RH, Jones WS. Thyroid function in human pregnancy. VII. Development and retardation of 4-year-old progeny of euthyroid and of hypothyroxinemic women. *Am J Obstet Gynecol.* 1971;109(1):12-19.

154.	Brent GA. The Debate over Thyroid-Function Screening in Pregnancy. *New England Journal of Medicine.* 2012;366(6):562-563.

155.	Haddow JE, McClain MR, Palomaki GE, Kloza EM, Williams J. Screening for thyroid disorders during pregnancy: results of a survey in Maine. *American Journal of Obstetrics & Gynecology.* 2006;194(2):471-474.

156.	Chang DL, Leung AM, Braverman LE, Pearce EN. Thyroid testing during pregnancy at an academic Boston Area Medical Center. *The Journal of Clinical Endocrinology & Metabolism.* 2011;96(9):E1452-E1456.

157.	Brent GA. The debate over thyroid-function screening in pregnancy. In: Mass Medical Soc; 2012.

158.	Cnattingius S. The epidemiology of smoking during pregnancy: smoking prevalence, maternal characteristics, and pregnancy outcomes. *Nicotine & tobacco research.* 2004;6(Suppl_2):S125-S140.

159.	Lumley J, Chamberlain C, Dowswell T, Oliver S, Oakley L, Watson L. Interventions for promoting smoking cessation during pregnancy. *Cochrane Database of Systematic Reviews.* 2009(3).

160.	Coleman T, Cooper S, Thornton JG, et al. A randomized trial of nicotine-replacement therapy patches in pregnancy. *New England Journal of Medicine.* 2012;366(9):808-818.

161.	Coleman T, Chamberlain C, Davey MA, Cooper SE, Leonardi‐Bee J. Pharmacological interventions for promoting smoking cessation during pregnancy. *The Cochrane Library.* 2015.

162.	Furlan AJ, Reisman M, Massaro J, et al. Closure or medical therapy for cryptogenic stroke with patent foramen ovale. *New England Journal of Medicine.* 2012;366(11):991-999.

163.	Maisel WH, Laskey WK. Patent foramen ovale closure devices: moving beyond equipoise. *JAMA.* 2005;294(3):366-369.

164.	Li J, Liu J, Liu M, et al. Closure versus medical therapy for preventing recurrent stroke in patients with patent foramen ovale and a history of cryptogenic stroke or transient ischemic attack. *Cochrane Database Syst Rev.* 2015(9):Cd009938.

165.	Shroyer AL, Grover FL, Hattler B, et al. On-pump versus off-pump coronary-artery bypass surgery. *n engl J med.* 2009;361(19):1827-1837.

166.	Grover FL. Current status of off-pump coronary-artery bypass. *N Engl J Med.* 2012;366(16):1541.

167.	Lamy A, Devereaux P, Prabhakaran D, et al. Off-pump or on-pump coronary-artery bypass grafting at 30 days. *New England Journal of Medicine.* 2012;366(16):1489-1497.

168.	Filardo G, Hamman BL, da Graca B, et al. Efficacy and effectiveness of on- versus off-pump coronary artery bypass grafting: A meta-analysis of mortality and survival. *The Journal of Thoracic and Cardiovascular Surgery.* 2018;155(1):172-179.e175.

169.	Takagi H, Watanabe T, Mizuno Y, Kawai N, Umemoto T, for the AG. A meta-analysis of large randomized trials for mid-term major cardio- and cerebrovascular events following off-pump versus on-pump coronary artery bypass grafting. *Interactive CardioVascular and Thoracic Surgery.* 2014;18(4):522-524.

170.	Tsao AL. Anticoagulation in Heart Failure in Sinus Rhythm. *American College of Cardiology.* 2015.

171.	Isnard R, Komajda M. Thromboembolism in heart failure, old ideas and new challenges. *European journal of heart failure.* 2001;3(3):265-269.

172.	Shantsila E, Lip GY. Antiplatelet versus anticoagulation treatment for patients with heart failure in sinus rhythm. *The Cochrane Library.* 2016.

173.	Raghu G, Collard HR, Egan JJ, et al. An official ATS/ERS/JRS/ALAT statement: idiopathic pulmonary fibrosis: evidence-based guidelines for diagnosis and management. *American journal of respiratory and critical care medicine.* 2011;183(6):788-824.

174.	Barton R. Inoperable brain metastases from non-small cell lung cancer: what part does whole brain radiotherapy play in standard treatment? *Thorax.* 2008;63(1):1-2.

175.	Bernard GR, Vincent J-L, Laterre P-F, et al. Efficacy and safety of recombinant human activated protein C for severe sepsis. *New England Journal of Medicine.* 2001;344(10):699-709.

176.	Wenzel RP, Edmond MB. Septic Shock — Evaluating Another Failed Treatment. *New England Journal of Medicine.* 2012;366(22):2122-2124.

177.	Eichacker PQ, Natanson C, Danner RL. Surviving Sepsis — Practice Guidelines, Marketing Campaigns, and Eli Lilly. *New England Journal of Medicine.* 2006;355(16):1640-1642.

178.	Ranieri VM, Thompson BT, Barie PS, et al. Drotrecogin Alfa (Activated) in Adults with Septic Shock. *New England Journal of Medicine.* 2012;366(22):2055-2064.

179.	EMS. Xigris drotrecogin alfa (activated): EPAR summary for the public. In. [Online]: European Medicines Agency; 2009.

180.	Lowes R. Sepsis Drug Xigris Pulled From Worldwide Market. *Medscape Alerts* 2011; October 25, 2011:<https://www.medscape.com/viewarticle/752169>. Accessed August, 01, 2018.

181.	Marti-Carvajal AJ, Sola I, Gluud C, Lathyris D, Cardona AF. Human recombinant protein C for severe sepsis and septic shock in adult and paediatric patients. *Cochrane Database Syst Rev.* 2012;12:Cd004388.

182.	Dellinger RP, Levy MM, Carlet JM, et al. Surviving Sepsis Campaign: international guidelines for management of severe sepsis and septic shock: 2008. *Intensive care medicine.* 2008;34(1):17-60.

183.	Perel P, Roberts I, Ker K. Colloids versus crystalloids for fluid resuscitation in critically ill patients. *The Cochrane Library.* 2013.

184.	Schortgen F, Lacherade J-C, Bruneel F, et al. Effects of hydroxyethylstarch and gelatin on renal function in severe sepsis: a multicentre randomised study. *The Lancet.* 2001;357(9260):911-916.

185.	Haase N, Perner A, Hennings LI, et al. Hydroxyethyl starch 130/0.38-0.45 versus crystalloid or albumin in patients with sepsis: systematic review with meta-analysis and trial sequential analysis. *Bmj.* 2013;346:f839.

186.	Kris-Etherton PM, Harris WS, Appel LJ. Omega-3 fatty acids and cardiovascular disease: new recommendations from the American Heart Association. In: Am Heart Assoc; 2003.

187.	Aung T, Halsey J, Kromhout D, et al. Associations of Omega-3 Fatty Acid Supplement Use With Cardiovascular Disease Risks: Meta-analysis of 10 Trials Involving 77917 Individuals. *JAMA cardiology.* 2018;3(3):225-234.

188.	National CGCU. Lipid modification: cardiovascular risk assessment and the modification of blood lipids for the primary and secondary prevention of cardiovascular disease. 2014.

189.	Association AD. American College of Endocrinology and American Diabetes Association consensus statement on inpatient diabetes and glycemic control: a call to action. *Diabetes care.* 2006;29(8):1955-1962.

190.	Angus DC, Abraham E. Intensive insulin therapy in critical illness. In: Am Thoracic Soc; 2005.

191.	Kavanagh BP. Glucose in the ICU—evidence, guidelines, and outcomes. In: Mass Medical Soc; 2012.

192.	Vlasselaers D, Milants I, Desmet L, et al. Intensive insulin therapy for patients in paediatric intensive care: a prospective, randomised controlled study. *The Lancet.* 2009;373(9663):547-556.

193.	Srinivasan V, Agus MS. Tight glucose control in critically ill children–a systematic review and meta‐analysis. *Pediatric diabetes.* 2014;15(2):75-83.

194.	Thiele H, Allam B, Chatellier G, Schuler G, Lafont A. Shock in acute myocardial infarction: the Cape Horn for trials? *European heart journal.* 2010;31(15):1828-1835.

195.	Antman EM, Anbe DT, Armstrong PW, et al. ACC/AHA guidelines for the management of patients with ST-elevation myocardial infarction—executive summary: a report of the American College of Cardiology/American Heart Association Task Force on Practice Guidelines (Writing Committee to Revise the 1999 Guidelines for the Management of Patients With Acute Myocardial Infarction). *Circulation.* 2004;110(5):588-636.

196.	Van de Werf F, Bax J, Betriu A, et al. Management of acute myocardial infarction in patients presenting with persistent ST-segment elevation. *European Heart Journal.* 2008;29(23):2909-2945.

197.	Unverzagt S, Machemer M-T, Solms A, et al. Intra-aortic balloon pump counterpulsation (IABP) for myocardial infarction complicated by cardiogenic shock. *Cochrane Database Syst Rev.* 2011;7.

198.	Safaei N, Montazerghaem H, Azarfarin R, Alizadehasl A, Alikhah H. Radiofrequency ablation for treatment of atrial fibrillation. *BioImpacts: BI.* 2011;1(3):171.

199.	Grubb NR, Furniss S. Science, medicine, and the future: radiofrequency ablation for atrial fibrillation. *BMJ: British Medical Journal.* 2001;322(7289):777.

200.	Stevenson WG, Albert CM. Catheter Ablation for Paroxysmal Atrial Fibrillation. *New England Journal of Medicine.* 2012;367(17):1648-1649.

201.	Siontis KC, Ioannidis JPA, Katritsis GD, et al. Radiofrequency Ablation Versus Antiarrhythmic Drug Therapy for Atrial Fibrillation: Meta-Analysis of Quality of Life, Morbidity, and Mortality. *JACC: Clinical Electrophysiology.* 2016;2(2):170-180.

202.	Hakalahti A, Biancari F, Nielsen JC, Raatikainen MP. Radiofrequency ablation vs. antiarrhythmic drug therapy as first line treatment of symptomatic atrial fibrillation: systematic review and meta-analysis. *EP Europace.* 2015;17(3):370-378.

203.	Landmark CABANA Trial Confirms Ablation Equal To or Superior to Drug Therapy. *Atrial Fibrillation* 2018; <https://www.dicardiology.com/article/landmark-cabana-trial-confirms-ablation-equal-or-superior-drug-therapy>. Accessed August 07, 2018.

204.	Finfer S, Liu B, Taylor C, et al. Resuscitation fluid use in critically ill adults: an international cross-sectional study in 391 intensive care units. *Critical care.* 2010;14(5):R185.

205.	Childers CP, Maggard-Gibbons M, Shekelle PG. Antiplatelet therapy in patients with coronary stents undergoing elective noncardiac surgery: continue, stop, or something in between? *Jama.* 2017;318(2):120-121.

206.	Gurbel PA, Becker RC, Mann KG, Steinhubl SR, Michelson AD. Platelet function monitoring in patients with coronary artery disease. *Journal of the American College of Cardiology.* 2007;50(19):1822-1834.

207.	Felker GM, Mentz RJ. Diuretics and ultrafiltration in acute decompensated heart failure. *Journal of the American College of Cardiology.* 2012;59(24):2145-2153.

208.	Jessup M, Abraham WT, Casey DE, et al. 2009 focused update: ACCF/AHA guidelines for the diagnosis and management of heart failure in adults: a report of the American College of Cardiology Foundation/American Heart Association Task Force on Practice Guidelines: developed in collaboration with the International Society for Heart and Lung Transplantation. *Circulation.* 2009;119(14):1977-2016.

209.	Go AS, Chertow GM, Fan D, McCulloch CE, Hsu C-y. Chronic kidney disease and the risks of death, cardiovascular events, and hospitalization. *New England Journal of Medicine.* 2004;351(13):1296-1305.

210.	Block GA, Hulbert-Shearon TE, Levin NW, Port FK. Association of serum phosphorus and calcium x phosphate product with mortality risk in chronic hemodialysis patients: a national study. *American Journal of Kidney Diseases.* 1998;31(4):607-617.

211.	Administration UFaD. Approval letter: Cinacalcet. In. Rockville, MD: Department of Health & Human Services; 2004.

212.	Strippoli G, Tong A, Palmer S, Elder G, Craig J. Calcimimetics for secondary hyperparathyroidism in chronic kidney disease patients. *Cochrane Database Syst Rev.* 2006;4.

213.	Kochanek PM, Berger RP, Margulies SS, Jenkins LW. Inflicted childhood neurotrauma: new insight into the detection, pathobiology, prevention, and treatment of our youngest patients with traumatic brain injury. *Journal of Neurotrauma.* 2007;24(1):1.

214.	Forsyth RJ, Wolny S, Rodrigues B. Routine intracranial pressure monitoring in acute coma. *The Cochrane Library.* 2010.

Total: 67

| **#** | **Article and Author** | **Primary Medical Discipline** | **Date**  **Journal** | **Summary** | **Systematic review** | **Systematic Review Search Terms** |
| --- | --- | --- | --- | --- | --- | --- |
| 229 | Diuretic Strategies in Patients with Acute Decompensated Heart Failure  Felker et al. | Cardiovascular disease | 3/3/2011  NEJM | Continuous diuretics in heart failure is a more resource intensive practice than bolus diuretics but believed to offer clinical benefit due to physiological considerations. Treatment for acute decompensated heart failure includes the use of intravenous loop diuretics, but recommendations regarding mode of administration (continuous infusion as compared to intermittent boluses) and dosage varies widely due to conflicting evidence and underpowered studies.^1^ A prospective, randomized, double-blind controlled trial (N=308) found that there were no differences in self-assessed symptoms or in renal function when comparing patients administered a bolus or with continuous infusion (mean AUC, 4236±1440 with boluses and 4373±1404 with continuous infusion; P=0.47; mean change in creatinine level, 0.05±0.3 mg per deciliter [4.4±26.5 μmol per liter] with boluses and 0.07±0.3 mg per deciliter [6.2±26.5 μmol per liter] with continuous infusion; P=0.45) or at a high dose (mean AUC, 4430±1401 vs. 4171±1436; P=0.06; creatinine level (0.08±0.3 mg per deciliter [7.1±26.5 μmol per liter]) as compared with a low dose (0.04±0.3 mg per deciliter [3.5±26.5 μmol per liter] with the low-dose strategy, P=0.21). This is a reversal of the more resource intensive practice of continuous diuretics in patients with acute decompensated heart failure. | 2014. “Meta-analysis of the existing limited studies did not confirm any significant differences in the safety and efficacy with continuous administration of loop diuretic, compared with bolus injection in patients with acute decompensated heart failure.”^2^ | PubMed related articles |
| 230 | Long-Acting Risperidone and Oral Antipsychotics in Unstable Schizophrenia  Rosenheck et al. | Psychiatry | 3/3/2011  NEJM | Long-acting risperidone is an antipsychotic injectable agent used to treat patients with schizophrenia. ^3^ Risperidone is a drug that was introduced in the early 1990s and is widely prescribed.^4^ This second-generation drug, risperidone, is thought to cause fewer extrapyramidal symptoms and reduce hospital use compared to oral drugs, yet there is a lack of research on risperidone efficacy in patients with unstable disease.^5^ This study (N=1045) found that long-acting injectable risperidone had no greater effect on the rate of hospitalization, (P=0.39 by the log-rank test; hazard ratio, 0.87, 95% confidence interval [CI], 0.63 to 1.20), psychiatric symptoms, quality of life (QoL), and social and neurological functioning in patients with schizophrenia compared to a psychiatrist's choice of oral treatment. Furthermore, it was associated with more extrapyramidal symptoms.^6^ This is a reversal of injecting long-acting risperidone to treat patients with unstable schizophrenia. | 2016. Cochrane review. "Depot risperidone [long-acting] may be more acceptable than placebo injection but it is hard to know if it is any more effective in controlling the symptoms of schizophrenia. The active drug, especially higher doses, may be associated with more movement disorders than placebo." ^7^  2014. Schizophrenia Bulletin. “Pooled [long-acting injectable antipsychotics] LAIs did not reduce relapse compared with [oral antipsychotics] OAPs in schizophrenia patients.” ^8^ | Found in pubmed related article |
| 231 | Intervention to Reduce Transmission of Resistant Bacteria in Intensive Care  Huskins et al. | Critical care medicine | 4/14/2011  NEJM | ICUs are at high risk for MRSA and VRE infection transmission and the standard intervention in many health care settings has been through hand hygiene, barrier precautions in the presence of infected patients, dedicated instruments and equipment for infected patients, and reserved rooms for infected patients.^9^ Health care facilities have also implemented additional interventions such as active surveillance and topical antimicrobial treatments in order to prevent asymptomatic patients from spreading infections. ^10-12^ STAR*ICU was a unmasked, cluster-randomized, controlled trial of more than 9000 patients admitted to 18 ICUs comparing interventions for the prevention of MRSA study found that culture-based active surveillance and expanded use of barrier precautions was not effective in reducing the incidence of MRSA and VRE infections in adult ICUs compared to existing hospital practices.^13^ (40.4±3.3 and 35.6±3.7 in the two groups, respectively; P=0.35). This is a reversal of culture-based active surveillance and expanded use of barrier precautions as an intervention to reduce transmission of resistant bacteria in intensive care. | 2015. Cochrane review. “We found no studies assessing the effects of wearing gloves, gowns or masks for contact with MRSA hospitalised patients, or with their immediate environment, on the transmission of MRSA to patients, hospital staff, patients' caregivers or visitors. This absence of evidence should not be interpreted as evidence of no effect for these interventions. The effects of gloves, gowns and masks in these circumstances have yet to be determined by rigorous experimental studies, such as cluster-randomised trials involving multiple wards or hospitals, or interrupted time series studies.”^14^ | Found in pubmed related article |
| 232 | Decompressive Craniectomy in Diffuse Traumatic Brain Injury  Cooper et al. | Neurology/neurosurgery | 4/21/2011  NEJM | Increased intracranial pressure is a dangerous outcome of severe traumatic brain injury.^15^ Clinical practice guidelines recommend monitoring and using first-tier therapies to control intracranial pressure.^16^ When first-tier therapies fail to control pressure, surgical decompressive craniectomy has been recommended as an option to alleviate pressure.^17^ DECRA was a multicenter, randomized controlled trial in Australia, New Zealand, and Saudi Arabia of N=155 patients under age 60 with severe traumatic brain injury—Glasgow Coma Scale score of 3 to 8. Patients with refractory intracranial hypertension, defined as an intracranial pressure higher than 20 mm Hg for more than 15-minutes in whom first-tier intensive care and neurosurgical therapies had not maintained intracranial pressure below accepted targets, were randomized to either standard of care or early bifrontotemporoparietal decompressive craniectomy plus standard of care. This study showed that decompressive craniectomy, compared to standard care, decreased mean intracranial pressure (14.4 mm Hg vs. 19.1 mm Hg, P<0.001) and duration of ventilatory support and ICU stay, but was associated with significantly worse functional outcomes at 6 months after injury on the basis of proportional odds analysis of the Extended Glasgow Outcome Scale (median score, 3 vs. 4; odds ratio for a worse functional outcome in the craniectomy group, 1.84; 95% confidence interval [CI], 1.05 to 3.24; P=0.03) for patients with severe diffuse traumatic brain injury and increased intracranial pressure that was refractory to first-tier therapies.^18^ This is a reversal of decompressive craniectomy plus standard of care in patients with severe diffuse traumatic brain injury that was refractory to first-tier therapies. | 2017. “If one sets the bar at the level of functional independence, then the RCT data raises questions pertaining to the utility of decompressive craniectomy and barbiturate coma in the setting of sTBI [severe traumatic brain injury].” ^19^  Cooper et. al. is a reversal on early decompressive craniectomy (within the first 72 hours after injury), however, the subsequent RESCUE ICP trial showed an increased survival when decompressive craniectomy was administered to patients as last-tier therapy. ^20^ | Found in citing articles section on NEJM |
| 233 | Body-Weight–Supported Treadmill Rehabilitation after Stroke  Duncan et al. | Neurology/neurosurgery | 05/26/2011 NEJM | Locomotor training using body weight support on treadmills is a type of physical therapy intervention used in patients recovering from stroke. While a Cochrane review found that the use of these specialized locomotor training programs did not have substantial research backing its practice,^21^ the therapy was still being adopted and commercial lifts and robot-assisted stepping were in use in recovering patients.^22^ The LEAPS CT compared two different therapeutic exercise programs, early (2-months after stroke) versus late (6 months after stroke) locomotor training with home physical therapy program in post-stroke patients. Outcomes measured the improvement of functional walking capacity one year after stroke. The study found that locomotor training with body-weight support in stepping on a treadmill was not superior to progressive exercise at home managed by a physical therapist. Thehome-exercise program had fewer risks and may be more feasible.^23^ LEAPS trial is a reversal of locomotor training, which includes the use of body-weight support in stepping on a treadmill as rehabilitation therapy after stroke as compared to progressive exercise at home managed by a physical therapist | 2016. Cochrane review. "Cardiorespiratory training and, to a lesser extent, mixed training reduce disability during or after usual stroke care; this could be mediated by improved mobility and balance. There is sufficient evidence to incorporate cardiorespiratory and mixed training, involving walking, within post-stroke rehabilitation programmes to improve the speed and tolerance of walking; some improvement in balance could also occur. There is insufficient evidence to support the use of resistance training. The effects of training on death and dependence after stroke are still unclear but these outcomes are rarely observed in physical fitness training trials. Cognitive function is under-investigated despite being a key outcome of interest for patients. Further well-designed randomised trials are needed to determine the optimal exercise prescription and identify long-term benefits."^24^ | Found in citing articles section on NEJM |
| 234 | Immediate versus Delayed IUD Insertion after Uterine Aspiration Bednarek et al. | Obstetrics and gynecology | 6/9/2011  NEJM | It is often recommended that women return for a follow-up visit after an abortion procedure for a separate IUD insertion because it is believed that immediate insertion of an intrauterine device (IUD) following an abortion by uterine aspiration will increase the risk of expulsion of the IUD, infection, and uterine perforation. This recommendation often contradicts women's preferences.^25^ However, a randomized trial (N=578) discovered that when IUD insertion is performed immediately after uterine aspiration, the rates of expulsion, as compared with those with delayed insertion, did not differ significantly (difference, 2.3 percentage points; 95% CI, –1.0 to 5.8). Furthermore, immediate insertion also resulted in increased rates of IUD use (92.3%, vs. 76.6% after delayed insertion; P<0.001), and subsequently fewer unintended pregnancies. In addition, there was no difference in after first-trimester uterine aspiration, and the rate of IUD expulsion, although higher than that with delayed insertion, is low and statistically noninferior to the rate with delayed insertion. In addition, no differences were found between groups with regard to infection or other complications. This is a reversal of delayed IUD insertion after uterine aspiration. | 2014. Cochrane review. “Moderate quality evidence shows that insertion of an IUD immediately after abortion is safe and practical. IUD expulsion rates appear higher immediately after abortions compared to delayed insertions. However, at six months postabortion, IUD use is higher following immediate insertion compared to delayed insertion.”^26^ | Found in pubmed related article |
| 235 | Mortality after Fluid Bolus in African Children with Severe Infection  Maitland et al. | Critical care medicine | 6/30/2011  NEJM | Children who experience shock and life-threatening infections are often treated with fluid resuscitation, per the World Health Organization's (WHO) guidelines.^27^ The WHO specifically states fluid resuscitation is intended for children suffering from advanced shock, however the efficacy of this practice has not been firmly established. A two-stratum, multicenter, open, randomized, controlled study (N=3141) in Kenya, Tanzania, and Uganda found that mortality actually significantly increased among critically ill children who received albumin fluid boluses, compared to children who received saline boluses(relative risk for saline bolus vs. control, 1.44; 95% confidence interval [CI], 1.09 to 1.90; P=0.01; relative risk for albumin bolus vs. saline bolus, 1.01; 95% CI, 0.78 to 1.29; P=0.96; and relative risk for any bolus vs. control, 1.45; 95% CI, 1.13 to 1.86; P=0.003). This is a reversal of fluid bolus administration in African children with severe infection. | 2014. Systematic Review. “The global evidence base for bolus fluid therapy in children with severe febrile illness and signs of impaired circulation was of very low quality. This large study provides robust evidence that in low-income settings, fluid boluses increase mortality in children with severe febrile illness and impaired circulation, and this increased risk is consistent across children with severe and less severe circulatory impairment.^28^ | Found in citing articles section on NEJM |
| 236 | Primary Isoniazid Prophylaxis against Tuberculosis in HIV-Exposed Children Madhi et al. | Infectious disease | 7/7/2011  NEJM | Human immunodeficiency virus (HIV) coinfection with *Mycobacterium tuberculosis* (MTB) is highly endemic in sub-Saharan Africa. As such, the World Health Organization (WHO) recommends a tuberculosis-prevention strategy with preexposure isoniazid chemoprophylaxis to be administered to both children and adults who are HIV positive or who have been exposed to HIV.^29^ P1041— a multicenter, randomized, double-blind, placebo controlled trial in Johannesburg, Tygerberg, Durban and Botswana of n=548 HIV-infected and n=806  HIV-uninfected infants (91 to 120 days old)—compared the administration of daily isoniazid or matching placebo with respect to tuberculosis-disease-free survival (DFS) among HIV-infected children and tuberculosis-infection-free survival (infection-free survival) among HIV-uninfected children for 96 weeks. HIV-infected children had access to antiretroviral therapy [ART]. Both HIV-infected and HIV-uninfected children received Calmette-Guerin (BCG) vaccination against tuberculosis within 30 days after birth. This study found no benefit of isoniazid as preexposure prophylaxis in improving DFS among HIV-infected children (tuberculosis or death occurred 19% in the isoniazid group and 19.3% in the placebo group; p=0.93). Amid the HIV-uninfected children, there was no significant difference in the combined incidence of tuberculosis infection, tuberculosis disease, or death between groups (isoniazid group 10% and placebo group 11%; p=0.93).The study reported the rate of tuberculosis was 121 cases per 1000 child-years (95% confidence interval [CI], 95 to 153) among HIV-infected children as compared with 41 per 1000 child-years (95% CI, 31 to 52) among HIV-uninfected children. This is a reversal of the administration of preexposure isoniazid against tuberculosis in children previously administered BCG vaccine who are either HIV-uninfected but HIV exposed or HIV positive children on ART. | 2017. Cochrane review. "Isoniazid prophylaxis given to all children diagnosed with HIV may reduce the risk of active TB and death in HIV-positive children not on antiretroviral therapy [ART] in studies from Africa. For children on ART, no clear benefit was detected."^30^ | Found in pubmed related article |
| 237 | Effect of Nesiritide in Patients with Acute Decompensated Heart Failure  O’ Connor et al. | Cardiovascular disease | 7/7/2011 NEJM | Nesiritide has been an approved medication for people suffering from acute heart failure due to the belief that this drug would reduce pulmonary-capillary wedge pressure and improve dyspnea.^31^ The ASCEND-HF randomized controlled trial of n=7141 patients hospitalized with acute heart failure comparednesiritide against the standard of care it found that there was no difference in rates of self-reported dyspnea at 6 and 24 hours, rehospitalization for heart failure or death from any cause at 30 days, and renal dysfunction. However, due to the significant increase in hypotension among patients who were taking nesiritide, it is recommended that this medication not be prescribed to people suffering from acute heart failure. This is a reversal of administering nesiritide in patients with acute decompensated heart failure. | 2014. “To the best of our knowledge, this is the first time that any study has (using cumulative meta-analysis) demonstrated that there was no reduction in mortality rate, according to either short-term or long-term follow-ups since the first RCT performed on ADHF management with nesiritide in 1999.”^32^ | Google Scholar |
| 238 | Early versus Late Parenteral Nutrition in Critically Ill Adults  Casaer et al. | Critical care | 8/11/2011  NEJM | Guidelines for initiation of parenteral nutrition vary globally, with European guidelines advocating for initiation as early as two days after a person is admitted into the intensive care unit^33^ and USA guidelines encouraging waiting for a week or longer.^34,35^ A randomized multicenter trial (N=4640) compared early initiation of parenteral nutrition with late initiation among adults in the ICU and found that late parenteral nutrition led to a quicker recovery time and decreased complications. Patients in the late-initiation group, as compared with the early-initiation group, had fewer ICU infections (22.8% vs. 26.2%, P=0.008), a lower incidence of cholestasis (P<0.001), a reduction of 9.7% in the proportion of patients requiring more than 2 days of mechanical ventilation (P=0.006), a median reduction of 3 days in the duration of renal-replacement therapy (P=0.008), and a mean reduction in health care costs of €1,110 (about $1,600) (P=0.04). This is a reversal of early initiation of parenteral nutrition in critically ill adults. | 2017. Cochrane review. "There is low-quality evidence for the effects of nutrition support on mortality and serious adverse events. Based on the results of our review, it does not appear to lead to a risk ratio reduction of approximately 10% or more in either all-cause mortality or serious adverse events at short-term and long-term follow-up.There is very low-quality evidence for an increase in weight with nutrition support at the end of treatment in hospitalised adults determined to be at nutritional risk. The effects of nutrition support on all remaining outcomes are unclear. Despite the clinically heterogenous population and the high risk of bias of all included trials, our analyses showed limited signs of statistical heterogeneity. Further trials may be warranted, assessing enteral nutrition (tube-feeding) for different patient groups. Future trials ought to be conducted with low risks of systematic errors and low risks of random errors, and they also ought to assess health-related quality of life."^36^ | Found in pubmed related article |
| 239 | Prevention of Intraoperative Awareness in a High-Risk Surgical Population  Avidan et al. | Anesthesiology | 8/18/2011  NEJM | Unintended intraoperative awareness is rare but occurs, nonetheless, in approximately 1% of patients undergoing surgery.^37^ The electroencephalogram-derived bispectral index (BIS) device has been developed to detect a patient's level of consciousness, assigning a value from 0 to 100, from completely unconscious to fully awake.^38^ BAG-RECALL a prospective, randomized trial of patients at a high-risk of intraoperative awareness N=6041 and compared the measure of incidence of intraoperative awareness among these patients when these patients were randomized to standard monitoring of end-tidal anesthetic-agent concentration (ETAC) or use of a single frontal electroencephalographic signal monitor the BIS Quatro device (Covidien). A widely used monitor, which uses a proprietary algorithm to calculate a dimensionless number intended to indicate the patient’s level of consciousness. A range of 40-60 has been advocated for reducing the dose of anesthetic agent and to prevent patient awareness.^39^ BAG-RECALL found that the BIS protocol (0.24% of patients) was not superior to ETAC (0.07%), meaning the BIS group experiences awareness during surgery more often than the standard monitoring of ETAC group, (a difference of 0.38 percentage points; 95% CI, 0.03 to 0.74; P=0.99) This is a reversal of using the BIS Quatro device (Covidien) and BIS protocol to detect intraoperative awareness in a high-risk surgical population. TheBAG-RECALL trial fail to show that ETAC protocol is associated with an increase in postoperative mortality or in the amount of anesthetic agent administered. | 2016. Cochrane review. "Anesthetic depth monitors may have similar effects to standard clinical and electrical monitoring on the risk of awareness during surgery."^40^ | Found in pubmed related article |
| 240 | Niacin in Patients with Low HDL Cholesterol Levels Receiving Intensive Statin Therapy  The AIM-HIGH Investigators | Cardiovascular disease | 12/15/2011  NEJM | Patients with established cardiovascular disease benefit from lowering LDL cholesterol levels; although there are still residual, cardiovascular risks after reaching targeted LDL levels. Epidemiological studies have shown that low HDL levels are an independent predictor of coronary heart disease and therefore raising HDL levels was hypothesized to decrease risk.^41^ Niacin therapy for cholesterol maintenance has been used since the 1950s. The 2001 National Cholesterol Education Program Adult Treatment Panel guidelines recommend the addition of niacin to LDL therapy in high-risk patients with low HDL levels.^42^ The AIM-HIGH study of patients with established atherosclerotic cardiovascular disease and LDL cholesterol levels of less than 70 mg/dL,(N=3414) received extended-release niacin, 1500 to 2000 mg per day, or matching placebo, found that niacin (Niaspan) added to statin therapy had no clinical benefit—  composite of the first event of death from coronary heart disease, nonfatal myocardial infarction, ischemic stroke, hospitalization (for >23 hours) for an acute coronary syndrome, or symptom-driven coronary or cerebral revascularization—compared to placebo, (hazard ratio, 1.02; 95% confidence interval, 0.87 to 1.21; P=0.79 by the log-rank test), despite significant improvements in HDL and triglyceride levels.^43^ This is a reversal of Niacin in addition to statin therapy in high-risk patients for preventing death from coronary heart disease, non-fatal myocardial infarction, ischemic stroke, hospitalization for an acute coronary syndrome, or symptom- driven coronary or cerebral revascularization, despite significant improvements in HDL cholesterol and triglycerides. | 2017. Cochrane review. "Moderate- to high-quality evidence suggests that niacin does not reduce mortality, cardiovascular mortality, non-cardiovascular mortality, the number of fatal or non-fatal myocardial infarctions, nor the number of fatal or non-fatal strokes but is associated with side effects. Benefits from niacin therapy in the prevention of cardiovascular disease events are unlikely."^44^ | Pubmed-found review |
| 241 | Liberal or Restrictive Transfusion in High-Risk Patients after Hip Surgery  Carson et al. | Surgery | 12/29/2011  NEJM | Both restrictive (a hemoglobin level of less than 8 g/dL or symptoms) and liberal (a hemoglobin level of 10 g/dL) strategies are used when transfusing blood to patients following surgery, yet the effect on recovery or risk of cardiac events from these different approaches is not understood.^45^ The Cardiovascular Patients Undergoing Surgical Hip Fracture Repair (FOCUS) randomized controlled study (N=2016) compared both approaches in patients following hip surgery. The study found that a liberal transfusion strategy was not associated with improved outcomes—reduced risk of death, reduced inability to walk independently on 60-day follow-up or reduced in-hospital morbidityodds ratio in the liberal-strategy group, 1.01; 95% confidence interval [CI], 0.84 to 1.22, for an absolute risk difference of 0.5 percentage points (95% CI, −3.7 to 4.7). The rates of in-hospital acute coronary syndrome or death were 4.3% and 5.2%, respectively (absolute risk difference, −0.9%; 99% CI, −3.3 to 1.6), and rates of death on 60-day follow-up were 7.6% and 6.6%, respectively (absolute risk difference, 1.0%; 99% CI, −1.9 to 4.0). This is a reversal of a liberal transfusion strategy in high-risk patients after hip surgery. | 2017. Cochrane review. "There is low-quality evidence that a restrictive RBC transfusion policy reduces the number of RBC transfusions per participant. There is low-quality evidence that a restrictive RBC transfusion policy has little or no effect on: mortality at 30 to 100 days, bleeding, or hospital stay."^46^ | Pubmed-found review |
| 242 | Low-Molecular-Weight Heparin and Mortality in Acutely Ill Medical Patients  Kakkar et al. | Critical care medicine | 12/29/2011  NEJM | Thromboprophylaxis is a strategy used to prevent deep vein thrombosis (DVT) in both surgical patients and patients that are medically ill.^47,48^ Despite current guidelines recommending the use of thromboprophylaxis for both surgical and acutely ill patients, acutely ill medical patients often do not receive this treatment.^47^ LIFENOX a randomized control trial (N=8307) compared the effect on the rate of death from any cause on acutely ill medical patients that were treated by pharmacologic thromboprophylaxis with enoxaparin, a low molecular weight heparin, as compared to placebo— both groups were administered elastic stockings with graduated compression. This study found that there was no reduction in the rate of death in the experimental group (Risk ratio, 1.0; 95% confidence interval [CI], 0.8 to 1.2; P=0.83). Therefore, thromboprophylaxis among acutely ill medical patients may not have a benefit in terms of reduced mortality. This is a reversal of thromboprophylaxis with enoxaparin plus elastic stockings with graduated compression alone in hospitalized acutely ill medical patients to prevent death from any cause. | 2014. Cochrane review. The LIFENOX trial did not assess the risk of venous thromboembolism or asymptomatic DVT, because their primary outcome was all cause mortality and safety. When this Cochrane review compared heparin versus placebo or no treatment they found that, “there was no clear evidence of a difference in mortality between the two treatment groups, with an OR of 0.97 (95% CI 0.87 to 1.08; P= 0.57) and heparin resulted in an increase in major haemorrhage (OR 1.65, 95% CI 1.01 to 2.71; P = 0.05).”^49^ | Pubmed-found review |

1.	Allen LA, Turer AT, DeWald T, Stough WG, Cotter G, O’Connor CM. Continuous Versus Bolus Dosing of Furosemide for Patients Hospitalized for Heart Failure. *The American journal of cardiology.* 2010;105(12):1794-1797.

2.	Wu M-Y, Chang N-C, Su C-L, et al. Loop diuretic strategies in patients with acute decompensated heart failure: A meta-analysis of randomized controlled trials. *Journal of Critical Care.* 2014;29(1):2-9.

3.	Application numberL 21-346 In: Research CFDEa, ed. U.S. Food & Drug Administration [Online]; 2003.

4.	Li C, Xia J, Wang J. Risperidone dose for schizophrenia. *Cochrane Database of Systematic Reviews.* 2009(4).

5.	Rasmussen S, Eisen J. Neuropsychopharmacology—5th Generation of Progress. 2002.

6.	Rosenheck RA, Krystal JH, Lew R, et al. Long-Acting Risperidone and Oral Antipsychotics in Unstable Schizophrenia. *New England Journal of Medicine.* 2011;364(9):842-851.

7.	Sampson S, Hosalli P, Furtado VA, Davis JM. Risperidone (depot) for schizophrenia. *Cochrane Database of Systematic Reviews.* 2016(4).

8.	Kishimoto T, Robenzadeh A, Leucht C, et al. Long-acting injectable vs oral antipsychotics for relapse prevention in schizophrenia: a meta-analysis of randomized trials. *Schizophrenia bulletin.* 2014;40(1):192-213.

9.	Muto CA, Jernigan JA, Ostrowsky BE, et al. SHEA guideline for preventing nosocomial transmission of multidrug-resistant strains of Staphylococcus aureus and enterococcus. *Infection Control & Hospital Epidemiology.* 2003;24(5):362-386.

10.	Lucet J-C, Paoletti X, Lolom I, et al. Successful long-term program for controlling methicillin-resistant Staphylococcus aureus in intensive care units. *Intensive Care Medicine.* 2005;31(8):1051-1057.

11.	Ostrowsky BE, Trick WE, Sohn AH, et al. Control of Vancomycin-Resistant Enterococcus in Health Care Facilities in a Region. *New England Journal of Medicine.* 2001;344(19):1427-1433.

12.	Recommendations for Preventing the Spread of Vancomycin Resistance Recommendations of the Hospital Infection Control Practices Advisory Committee (HICPAC). 1995; <https://www.cdc.gov/mmwr/preview/mmwrhtml/00039349.htm>. Accessed Feb. 05, 2018.

13.	Huskins WC, Huckabee CM, O'Grady NP, et al. Intervention to Reduce Transmission of Resistant Bacteria in Intensive Care. *New England Journal of Medicine.* 2011;364(15):1407-1418.

14.	López-Alcalde J, Mateos-Mazón M, Guevara M, et al. Gloves, gowns and masks for reducing the transmission of meticillin-resistant Staphylococcus aureus (MRSA) in the hospital setting. *Cochrane Database of Systematic Reviews.* 2015(7).

15.	Sahuquillo J. Decompressive craniectomy for the treatment of refractory high intracranial pressure in traumatic brain injury. *Cochrane Database of Systematic Reviews.* 2006(1).

16.	Brain Trauma F. American Association of Neurological S, Congress of Neurological S, Joint Section on N. *Critical Care AC, Bratton SL, et al Guidelines for the management of severe traumatic brain injury VI Indications for intracranial pressure monitoring J Neurotrauma.* 2007;24.

17.	Lumenta CB, Di Rocco, D., Haase, J., Jakob, J., Mooij, A,. *Neurosurgery* Springer-Verlag Berlin Heidelberg; 2010.

18.	Cooper DJ, Rosenfeld JV, Murray L, et al. Decompressive Craniectomy in Diffuse Traumatic Brain Injury. *New England Journal of Medicine.* 2011;364(16):1493-1502.

19.	Abraham P, Rennert RC, Gabel BC, et al. ICP management in patients suffering from traumatic brain injury: a systematic review of randomized controlled trials. *Acta Neurochirurgica.* 2017;159(12):2279-2287.

20.	Hutchinson PJ, Kolias AG, Timofeev IS, et al. Trial of Decompressive Craniectomy for Traumatic Intracranial Hypertension. *New England Journal of Medicine.* 2016;375(12):1119-1130.

21.	Mehrholz J, Pohl M, Elsner B. Treadmill training and body weight support for walking after stroke. *Cochrane Database of Systematic Reviews.* 2014(1).

22.	Duncan PW, Zorowitz R, Bates B, et al. Management of Adult Stroke Rehabilitation Care: a clinical practice guideline. *Stroke.* 2005;36(9):e100-143.

23.	Duncan PW, Sullivan KJ, Behrman AL, et al. Body-Weight–Supported Treadmill Rehabilitation after Stroke. *New England Journal of Medicine.* 2011;364(21):2026-2036.

24.	Saunders DH, Sanderson M, Hayes S, et al. Physical fitness training for stroke patients. *Cochrane Database Syst Rev.* 2016;3:Cd003316.

25.	Stanek AM, Bednarek PH, Nichols MD, Jensen JT, Edelman AB. Barriers associated with the failure to return for intrauterine device insertion following first-trimester abortion. *Contraception.* 2009;79(3):216-220.

26.	Okusanya BO, Oduwole O, Effa EE. Immediate postabortal insertion of intrauterine devices. *Cochrane Database of Systematic Reviews.* 2014(7).

27.	Hospital care for children: guidelines for the management of common illnesses with limited resources. 2005; <http://apps.who.int/iris/bitstream/10665/81170/1/9789241548373_eng.pdf>.

28.	Opiyo N, Molyneux E, Sinclair D, Garner P, English M. Immediate fluid management of children with severe febrile illness and signs of impaired circulation in low-income settings: a contextualised systematic review. *BMJ Open.* 2014;4(4):e004934.

29.	Granich R, Akolo C, Gunneberg C, Getahun H, Williams P, Williams B. Prevention of Tuberculosis in People Living with HIV. *Clinical Infectious Diseases.* 2010;50(Supplement_3):S215-S222.

30.	Zunza M, Gray DM, Young T, Cotton M, Zar HJ. Isoniazid for preventing tuberculosis in HIV-infected children. *Cochrane Database of Systematic Reviews.* 2017(8).

31.	Colucci WS, Elkayam U, Horton DP, et al. Intravenous nesiritide, a natriuretic peptide, in the treatment of decompensated congestive heart failure. Nesiritide Study Group. *N Engl J Med.* 2000;343(4):246-253.

32.	Yan B, Peng L, Zhao X, et al. Nesiritide fails to reduce the mortality of patients with acute decompensated heart failure: an updated systematic review and cumulative meta-analysis. *Int J Cardiol.* 2014;177(2):505-509.

33.	Singer P, Berger MM, Van den Berghe G, et al. ESPEN guidelines on parenteral nutrition: intensive care. *Clinical nutrition.* 2009;28(4):387-400.

34.	Heyland DK, Dhaliwal R, Drover JW, Gramlich L, Dodek P. Canadian clinical practice guidelines for nutrition support in mechanically ventilated, critically ill adult patients. *JPEN J Parenter Enteral Nutr.* 2003;27(5):355-373.

35.	Martindale RG, McClave SA, Vanek VW, et al. Guidelines for the provision and assessment of nutrition support therapy in the adult critically ill patient: Society of Critical Care Medicine and American Society for Parenteral and Enteral Nutrition: Executive Summary. *Crit Care Med.* 2009;37(5):1757-1761.

36.	Feinberg J, Nielsen EE, Korang SK, et al. Nutrition support in hospitalised adults at nutritional risk. *Cochrane Database Syst Rev.* 2017;5:Cd011598.

37.	Phillips AA, McLean RE, Devitt JH, Harrington EM. Recall of intraoperative events after general anaesthesia and cardiopulmonary bypass. *Canadian Journal of Anaesthesia.* 1993;40(10):922.

38.	Punjasawadwong Y, Boonjeungmonkol N, Phongchiewboon A. Bispectral index for improving anaesthetic delivery and postoperative recovery. *Cochrane Database Syst Rev.* 2007(4):Cd003843.

39.	Chan MTV, Cheng BCP, Lee TMC, Gin T, Group tCT. BIS-guided Anesthesia Decreases Postoperative Delirium and Cognitive Decline. *Journal of Neurosurgical Anesthesiology.* 2013;25(1):33-42.

40.	Messina AG, Wang M, Ward MJ, et al. Anaesthetic interventions for prevention of awareness during surgery. *Cochrane Database Syst Rev.* 2016;10:Cd007272.

41.	Assmann G, Schulte H, von Eckardstein A, Huang Y. High-density lipoprotein cholesterol as a predictor of coronary heart disease risk. The PROCAM experience and pathophysiological implications for reverse cholesterol transport. *Atherosclerosis.* 1996;124:S11-S20.

42.	Giugliano RP. Niacin at 56 Years of Age — Time for an Early Retirement? *New England Journal of Medicine.* 2011;365(24):2318-2320.

43.	Investigators TA-H. Niacin in Patients with Low HDL Cholesterol Levels Receiving Intensive Statin Therapy. *New England Journal of Medicine.* 2011;365(24):2255-2267.

44.	Schandelmaier S, Briel M, Saccilotto R, et al. Niacin for primary and secondary prevention of cardiovascular events. *Cochrane Database of Systematic Reviews.* 2017(6).

45.	Carson JL, Carless PA, Hebert PC. Transfusion thresholds and other strategies for guiding allogeneic red blood cell transfusion. *Cochrane Database Syst Rev.* 2012;4(1).

46.	Estcourt LJ, Malouf R, Trivella M, Fergusson DA, Hopewell S, Murphy MF. Restrictive versus liberal red blood cell transfusion strategies for people with haematological malignancies treated with intensive chemotherapy or radiotherapy, or both, with or without haematopoietic stem cell support. *Cochrane Database of Systematic Reviews.* 2017(1).

47.	Geerts WH, Bergqvist D, Pineo GF, et al. Prevention of venous thromboembolism: American College of Chest Physicians Evidence-Based Clinical Practice Guidelines (8th Edition). *Chest.* 2008;133(6 Suppl):381s-453s.

48.	Samama MM, Cohen AT, Darmon JY, et al. A comparison of enoxaparin with placebo for the prevention of venous thromboembolism in acutely ill medical patients. Prophylaxis in Medical Patients with Enoxaparin Study Group. *N Engl J Med.* 1999;341(11):793-800.

49.	Alikhan R, Forster R, Cohen AT. Heparin for the prevention of venous thromboembolism in acutely ill medical patients (excluding stroke and myocardial infarction). *Cochrane Database of Systematic Reviews.* 2014(5).
